# Supplementary material for: A systematic narrative review of the research evidence of the impact of intersectionality on service engagement and help-seeking across different groups of women, trans women, and non-binary individuals experiencing homelessness and housing exclusion
Source: PLoS One. 2025 Apr 24;20(4):e0321300. doi: 10.1371/journal.pone.0321300 (PMC12021236; doi:10.1371/journal.pone.0321300)
Supplement: S2 Appendix — . (PDF) [file pone.0321300.s002.pdf]

	Population category	Duration of study	Type of publication	Study description	Setting	Study Design	Type of Study	Sample/Recruitment	Sample size	Demographic characteristics of participants (e.g., age, gender, ethnicity)	Intersectionality (+gender)	Primary needs mentioned	Intervention: Interaction with services; help-seeking
**AGENDA (UK)**	Adolescent; Young People	not mentioned	Report	Investigating experiences of young women with multiple needs when accessing services.	10 specialist girls and women's voluntary sector organisations in the UK	Interviews and expert advisory group of girls and young women with lived experience of multiple unmet needs. ; Freedom of Information (FOI) and interview insights were analysed thematically and presented in form of case studies	Qualitative (mixed methods)	Unclear, but it can be assumed that they were drawn from the voluntary services they were working with. Interviews totalled 37 interviews with 36 girls and young women (aged 13 to 26) and 4 semi- structured interviews with practitioners	36 girls and young women	56% white, 22% black, 14% Mixed and White and Black Carribbean, 19% others; aged 13 to 26	Race/Ethnicity; Age, Pregnancy	DIV/IPV, Mental Health, Housing, Legal aid (criminal justice), Substance use, Child welfare	Specialist service (women; voluntary); mentioned engagement with statutory services (social services; police)
**AUSTIN (UK)**	Women (not fitting any other category)	< 1yr	Report	Evaluation and analysis of the experience of women experiencing domestic abuse and violence into "The No Woman Turned Away (NWTA) project", funded by the Ministry of Housing, Communities and Local Government, UK.	NWTA project across the UK; no specific location mentioned (volunutary service)	Service data on women's needs, demographics, outcomes was explored, the barriers they had faced and what happened to them while they were waiting for a refuge space or other safe outcomes. Data was complemented with in-depth qualitative interviews and arts-based methods	Mixed Methods	309 in dataset and 17 additional interview with NWTA specialist practitioners, support workers, and survivors; purposive sampling through the organisation	309 women in service, ranging between 16 and 70 (the majority in their 30s); 148 white, the other 50% from other backgrounds; 76% with a disability.	309 women in service, ranging between 16 and 70 (the majority in their 30s); 148 white, the other 50% from other backgrounds; 76% with a disability.	Race/Ethnicity; Disability, Pregnancy	DIV/IPV, Mental Health, Housing, Social care (disabilities) and child welfare	Voluntary service providing access to domestic Abuse refuge, also looked at acess to statutory services (Probation/Police, Social Service/ Social Care) and informal Support
**Baumann (Canada)**	LGBTQ*	< 1yr	Journal/ Research article	Investigating experiences of violence and head injury among women and transgender women sex workers.	Elizabeth Fry organisation in Toronto, Canada, serving women in conflict or at risk of being in conflict with the criminal justice system through provision of housing and community supports.	In-depth qualitative interviews and validated screening tools for TBI number and classification of head injuries participants suffered). Participatory research design, involving staff of Elizabeth Fry in the study and using trauma informed approaches to research. Thematic analysis of qualitative data	Mixed Methods	Purposive sampling to recruit potential participants. 10 face-to-face interviews with participants at the Elizabeth Fry Toronto WorkSafe program.	10 interviews with women	All self-identified as women, 50% as transgender women. Participants ranged from 43 to 59 years old. The majority of participants reported lower educational training (e.g., high school or less than a grade 10 education); primary source of income among participants were forms of government social assistance. Half of the participants lived in subsidised community housing, with the remaining in market rental housing, rooming houses, or shelters. 60% identified as Aboriginal or Metis, and the rest identified as Caucasian, Canadian, or East Asian. Most participants began sex work between 20 and 27 years of age, some as early as age 13 and others later in life (e.g., 36 years of age). Participants reported being engaged in street and off-street (such as online or indoor) sex work but several participants were not actively engaged in sex work at the time of the interview.	Disability, Gender Reassingment	Mental Health, Physical Health/ Primary Care, Housing, Legal aid, Education	Voluntary organisation, helping with access to criminal Justice, housing, and primary care
**Ben-Porat (Israel)**	Women (not fitting any other category)	< 1yr	Journal/ Research article	Investigating patterns of service utilization among Jewish, Arab, former Soviet Union (FSU)–born, and Ethiopian-born women who are victims of domestic violence in Israel	Domestic Abuse and Violence Shelter across Israel	Latent class analysis of questionnaire: excluding women not fitting criteria, data was collected from 526 women, with a response rate of 68.97%; excluding responded who completed than 42% of the questionnaire items; questionnaire was distributed by social workers at the shelters	Quantitative	Recruited from a samle of women applied for admission to shelters between the dates of September 2009 and April 2014 across Israel; from the sample withdrawn were women with cognitive impairments or various psychopathological conditions, women who withdrew from the shelter within 7 days of their arrival, or having responded to less than 42% of the questionnaire items	sample of 499 women who arrived at 12 shelters in Israel out of 1,409 female victims of domestic violence who applied for admission to shelters between the dates of September 2009 and April 2014.	28.4% were Israeli-born Jewish women, 31.3% were Israeli-born Arab women, 25.0% were FSU-born womenwho, 15.3% were Ethiopian-born women; Average age of the participants was 32.52 (SD = 8.41). They had an average of 10.95 years of education (SD = 3.44), and the average number of children born to each woman was 2.51 (SD = 1.66).	Race/Ethnicity	DIV/IPV, Mental Health	Engagement across all statutory services
**Benbow (Canada) - Until you hit**	Mothers	< 1yr	Journal/ Research article	Findings from of a larger study (Benbow, 2015) on social exclusion and health for mothers experiencing homelessness. The study investigated the sociopolitical context of mothers' experiencing social exclusion and how these experiences shape their health.	Partnerships with two organisations operating three shelters and a variety of programs and services. One organisation was a shelter for pregnant and parenting young mothers. The other organisation was a women's shelter for women and children who have experienced domestic violence. Both were in urban settings but in different cities.	Critical research paradigm, using interviews and focus groups with service provider participants	Qualitative	Participants were sampled purposefully based on self-identified homelessness and motherhood status and were recruited via flyer distribution, meet and greet sessions, and by word-of-mouth. Snowball sampling techniques were used, where women who participated shared research contact information with other mothers they knew who were experiencing homelessness.	41 participants comprising 26 mothers experiencing homelessness and 15 service providers who provided care to mothers experiencing homelessness participated in this study.	Participants were mothers with an average age of 27.4 years, and an average 1.6 children; the majority of children were under five years of age. Most mothers had physical custody of at least one of their children (n=19). Nineteen mothers self-identified as having or having had a mental illness, including depression, anxiety, posttraumatic stress disorder, panic disorder, and addiction. Seventeen mothers were born in Canada. While 10 mothers identified as Caucasian, mothers with a variety of racial and ethno-cultural identities participated in the study. Twenty-one mothers were receiving social assistance in the form of financial support (n=21).	Pregnancy/Maternity	Physical Health/ Primary Care; Mental Health; Housing	Interaction with shelter, housing, social services (children's welfare services), and healthcare
**Benbow (Canada) - Spaces of exclusion**		< 1yr	Journal/ Research article	Exploring women's mothering experiences while residing in a homeless shelter	Same as above	Same as above	Qualitative	Same as above	Same as above	Same as above	Race/Ethnicity; Prenancy	Mental Health, Housing, Child welfare	Same as above
**Benbow (Canada) - mothering without home**		< 1yr	Journal/ Research article	Exploring how mothers' experiences of social exclusion and homelessness are shaped, and the internalised impacts on homeless mothers' lives	Same as above	Same as above	Qualitative	Same as above	Same as above	Same as above	Pregnancy/Maternity	DIV/IPV, Housing, Child welfare	Same as above
**Bimpson (UK)**	Mothers	< 1yr	Journal/ Research article	Exploring how key governing frameworks, e.g. child protection processes, housing allocation policy and temporary accommodation provision in England interact with women's status as mother.	Towns and cities in the midlands and north of England	Exploratory study, using biographical research with homeless women.	Qualitative	Participants were recruited through organisations that provide refuge and other temporary accommodation for homeless people, and women's support services.	6 women out of 32 interviewed, who were mothers at the time of the research between one and six children	Mothers age ranged between 17 and mid- 50's. The majority of participants were White British. They had between 1-6 children. 18 were living apart from at least one of their children, some women's children had been adopted or placed into foster care, and just under half were in kinship care. Nine were living with at least one of their children. All research participants were homeless at the time of their interview, with most living in temporary accommodation (including refuges, hostels and supported housing), some staying with friends or family, and one sleeping rough.	Pregnancy/Maternity	DIV/IPV, Housing, Social care	Investigatng engagement with statutory Services (Housing, Social Services/Social Care)
**Boyd (Canada)**	Women (not fitting any other category)	< 1yr	Journal/ Research article	Examining how gender and race dynamics frame marginalised women's experiences of OPS.	Five mixed-gender OPS in Vancouver, BC's Downtown Eastside neighbourhood.	Rapid ethnographic study of OPS implementation in Vancouver, using observations, written fieldnotes, and interviews.	Qualitative	purposive sampling; recruited by team members, including two Indigenous peer researchers.	35 WWUD recruited from three OPS	35 were women, 2 of which were transgender or two-spirit participants. More than half of women participants were Indigenous, homeless, and had experienced at least one overdose in the past year.	Race/Ethnicity; Gender Reassignment	Substance Use	Substance Use Services

**Brais (Canada)**	Women (not fitting any other category)	1 to <5	Journal/ Research article	Study is part of a larger research project exploring the trajectories of women through transitional housing services within an organisation with an open door policy of inclusionary access to services and how inclusionary service access policy is perceived in an environment with a heterogeneous service user population	Temporary housing community organisation in Montreal, Quebec, Canada. Participants stay from a few weeks to over a year. Services provided on-site include mental health resources and counselling. The service promotes an inclusive access policy, by which women are not refused access because of age, race, ethnicity, sexuality, gender identity, physical or mental disability, or substance dependence.	Case study approach; In-depth description of participants and the service provider space, review of subset of data and thematic analysis through an iterative process. Partipants were interviewed in several waves. First wafe during participant's initial stay, second wave 12 months later by contacting consenting participants	Qualitative	Participants were recruited via promotional material displayed in the service and snowball sampling.	33 semi-structured interviews in Wave I; 12 follow-up interviews in Wave II; all but one participant had moved on from transitional housing and were securely housed.	Participant median age was 43 years; 30% were born outside of Canada; 55% were Francophone; 8% identified as Aboriginal (the term employed by the database used by service provider); 32% reported a homelessness duration of more than one year.	Gender Reassignment; Race/Ethnicity	Mental Health, Housing, Substance use	Shelter policy
**Cardwell (UK)**	Mothers	< 1yr	Report	Exploring the maternity experiences of women facing multiple disadvantage.	Local statutory and voluntary support services in two north-east London boroughs – Hackney and Barking and Dagenham	Participatory presearch, including eleven peer researchers with experiences of multiple disadvantage	Qualitative	purposeful sampling and proactive contact with local statutory and Voluntary Community Sector support services or snowball sampling.	34 women, 20 in Barking and Dagenham, 14 in Hackney	The majority of participants (n = 23) were between 25-39 and all were primary carers for their children. More than 50% had exprienced mental health issues, homelessness and/or housing difficulties, domestic violence and abuse. 50% had financial difficultiues. 15% had difficulties speaking or understanding English and experienced social services involvement. Three women had a learning disability.	Race/Ethnicity, Pregnancy/Maternity	DIV/IPV, Mental Health	Housing; Social Care; volntary and community services; Healthcare (pre- and antenatal)
**Dashora (US)**	Mothers	< 1yr	Journal/ Research article	Understanding the needs and intervention desires of substance-abusing homeless mothers from their own perspectives.	Local emergency shelter for families in Ohio	"Ground up" approach, qualitative content analysis procedure, and grounded theory to analyse the data. Three focus groups were conducted between July and August 2009 at the family shelter and each focus group included 8 to 10 mothers.	Qualitative	Participants were recruited from a local family shelter.	28 women	The average age of the mothers was 29.2 years, ranging 18–40 years. Self-identified ethnicity included African American (60.8%), White (32.1%), and mixed ethnicity/race (7.1%). Of the 28 mothers, 16 (57.2%) were single/never married, seven (25%) were divorced, three (10.7%) were separated but still married, and two (16%) were legally married. On average, mothers had 3.1 children (SD = 1.7) whose ages ranged from newborn to 18 years old. Mothers reported 51.7% (SD = 38.6) days of substance use (alcohol and drug use) in the prior 90 days.	Disability; Pregnancy	Mental Health, Housing, Employment, Substance use, Child welfare, Education	Substance Use Services; Housing; Employment; Social Services
**Deal (US)**	Women (not fitting any other category)	1 to <5	Journal/ Research article	Discussing how race, gender, poverty, and violence specifically and uniquely shape the lives of African American women who experience homelessness.	The study is based in Nia Imani, a holistic long-term transitional living facility in Milwaukee, Wisconsin supports homeless African American women experiencing violence. Length of stay in the program ranges from several months to two years. Nia Imani houses a maximum of 14 female residents.	Descriptive study using community-based participatory research (focus group interviews) to examine the experiences of violence in the lives of women who were graduates of Nia Imani.	Qualitative	Participants were recruited from Nia Imani "graduates", meaning they were assessed as being able to access resources, live independently, and support themselves and their children. Nia Imani contacted 50 program graduates by scripted phone call, inviting them to participate in the focus group interviews.	4 focus group interviews with graduates (N= 40) from a long-term transitional housing program	Women's ages ranged from 19 to 61 years. The majority identified as African American (89.7%). 92% had children. The average length of stay for women at Nia Imani was 17.5 months.	Race/Ethnicity, Pregnancy/Maternity	DIV/IPV, Housing	(DV) Shelter; Coping mechanisms; Community and social network support
**Dudley (UK)**	Immigrants/ Refugee	< 1yr	Journal/ Research article	Exploring the state role, and particularly the immigration rule of 'no recourse to public funds', in making abuse worse for women with insecure immigration status who experience domestic violence.	Bradford, Glasgow and Luton	Interviewing staff in three cities in the UK.	Qualitative	Desk-based research to identify networks of interested parties, including a reference group of practitioners who were based in Belfast.	51 interviews with professionals within these organisations between April 2013 and February 2014	Practioners came from organisations in the three cities, including floating support services, refuge services (both black and minority ethnic [BME] women only and women's domestic violence services), helplines, trusts, police, legal advisors, BME support and advocacy organisations, homeless shelters; and refugee/ asylum-seeker support organisations, community-based women's centres, faith-based organisations assisting destitute people, and rape crisis services.	Race/Ethnicity	DIV/IPV, Legal aid; Housing	(DA) Shelter and women's centre interactions; Criminal Justice; Housing; help-seeking
**Duff (Canada)**	Mothers	not mentioned	Journal/ Research article	Describing the barriers that pregnant and parenting sex workers face and elucidate factors associated with experiencing these barriers.	Metro Vancouver (2010–present)	Epidemiological study; drawing on a cross-sectional analysis drawing on data from An Evaluation of Sex Workers Health Access (AESHA), a prospective cohort study of sex workers in Metro Vancouver (2010–present).	Quantitative	Interviewers/outreach staff were recruited using time–location sampling. Participant were recruited through day and night times outreach at off-street sex work venues (i.e., massage parlors, microbrothels, in-call locations), off-street self-advertising spaces (e.g., online, newspapers) and outdoor venues (i.e., streets, alleys).	399 sex workers who reported a history of pregnancy	38.8% were of Indigenous/Aboriginal ancestry, and 25% were new immigrant/migrant workers. 51.4% had graduated high school or had completed some postsecondary education. The median age of participants reporting barriers while pregnant and mothering was 35 (IQR = 28–42).	Race/Ethnicity/Pregnancy	DIV/IPV, Housing, Substance use, Child welfare	Statutory (Criminal justice, Probation/Police, Social Service/ Social Care); specialist shelters/safe spaces
**England (UK)**	LGBTQ*	not mentioned	Journal/ Research article	Research is part of a larger study, exploring trans people's experiences of homelessness undertaken in collaboration with Shelter Cymru (England 2019). It investigates how homelessness service spaces reincsribe cisnormativity	Homeless Shelter in Wales (no LGBTQ-specific provision existed in Wales at the time of the research)	Interviews with participants from larger study (19 out of 28) who had direct experience of hostel accommodation.	Qualitative	Participants were recruited via social media, through partner organisations and trans and queer community groups	19	Participants ranged in age from late teens to early sixties and identified as trans woman, trans man, non-binary, genderqueer, agender, trans- sexual and woman with a trans past.	Sexual Orientation; Gender Reassignment	not further described	inclusivity of shelter environment
**Glumbikova (Czech Republic)**	Mothers	1 to <5	Journal/ Research article	Investigating barriers and accelerators of reintegration into permanent forms of housing from the perspective of homeless mothers; how motherhood affects the process of reintegration and social work with single mothers from shelters into permanent housing.	Shelter in Czech Republic (no law on social housing in the Czech Republic; loss of housing is mainly managed by shelters)	participatory approach, using interviews and focus groups	Qualitative	purposive sampling through the shelter institution and snowball sampling	33 interviews with three groups of mothers with different length of stays in shelters and six focus groups	Mothers with a long life experience in a shelter in the city of Ostrava; 18 of which resided in the shelter, 8 leaving the shelter, and 5 mothers reintegrated into permanent housing for at least 18 months	Maternity	Housing; Child welfare	resilience and parenthood; engagement with social services (children's services)
**Gordon (UK)**	Pregnant Women	< 1yr	Journal/ Research article	Exploring the perspectives of women who have experienced pregnancy and homelessness to ascertain how to improve perinatal care.	Facilities for individuals experiencing homelessness in South Yorkshire	Qualitative research study using interviews	Qualitative	purposive sample of women who had experienced pregnancy and homelessness, recruited from three community settings. Women were also recruited through convenience and snowboard sampling with the support from the staff in the care settings.	11	Women were aged 18–40 years, nine were white British, and two were of British/Indian origin. They had lived in 15 cities across the UK. All had experienced trauma or abuse in childhood. Four had left home by the age of 16 years, and four had experienced teenage pregnancy. Most had not completed secondary education. Two had never worked and the remainder had worked in short-term, low-skilled jobs. Three women had experienced domestic abuse. The mean age of first pregnancy was 24 years (4 years below the national average), women had a maximum of five children, none of whom were in the mother's custody, and eight of the women identified as single.	Pregnancy	Childhood trauma/abuse, mental health, substance use, unplanned pregnancy	Health care: Primary Care (perinatal)

**Greene (Canada)**	Mothers	not mentioned	Journal/ Research article	Providing an understanding of the housing experiences of racialised mothers living with HIV to help develop an effective strategy that will address the factors associated with housing instability	AIDS service organisations, housing, health and social service agencies serving women, families, newcomers, as well as racialised and Aboriginal communities in Ontario.	Community-based research approach, using peer research assistants. Interviews took place in local community-based agencies and were analysed using thematic analysis.	Qualitative	Participants were recruited by distributing information posters in Ontario AIDS service organizations, and housing, health and social service agencies serving women, families, newcomers, as well as racialised and Aboriginal communities.	30 HIV-positive mothers from African or Caribbean communities living in Ontario, Canada (out of which 17 were presented in the findings)	Parents living with HIV who had children in their care, or who were not in their care due to their current housing situation. The participants included refugees, immigrant newcomers and first-generation Canadian citizens; families with one, two and multiple heads of households; a range of dependents; and racial and linguistic diversity.	Race/Ethnicity,Pregnancy/ Maternity	DIV/IPV, Physical Health (HIV), Housing	Shelter and access to permanent housing
**Greenfield (US)**	Transgender youth	1 to <5	Journal/ Research article	Investigating how TGE youth who are homeless and engaging in survival sex describe and understand the protective factors contributing to their resilience.	large U.S. city	Study extends from a larger one that was conducted in a large Northeastern city in the United States in 2011 and 2012, using in-depth qualitative interviews and secondary analysis of qualitative data using thematic analysis.	Qualitative	Respondent-driven sampling (RDS) was used to recruit participants from the larger study who identified as transgender, non-binary, or gender expansive (e.g., genderqueer, androgynous) and reported active engagement in survival sex. participants, identified by service providers and youth leaders were selected to facilitate the recruitment process.	57 TGE youth	Participants were between 17 and 26 years old (M=19.88; SD=1.55). They identified as transgender woman (53%), transgender man (16%), and other (32%; gender-queer, gender-fluid, androgynous, or non-binary). Participants identified their sexual orientation as heterosexual (33%), bisexual (16%), gay (12%), lesbian (11%), and queer, questioning, or other (28%). The majority of youth in this study identified as racial or ethnic minorities, with 40% identifying as Black, 16% as Latino/a, and 35% as multiracial. The remainder identified as White (5%) or another race (3%). 47% did not complete high school, 26.5% only obtained a high school diploma, GED, or equivalent, 23% attended at least some college, and the remainder did not provide their level of education (3.5%).	Age, Ethnicity/Race, Gender Reassignment	Mental Health, Sexual health, Physical Health, Subsistence needs (food, clothes)	Statutory (Probation/Police, Healthcare Services: Physical and Mental Health Care; Sexual Health); Reslience and relying on oneself and their support network
**Gultekin (US)**	Mothers (Family homelessness)	1 to <5	Journal/ Research article	investigating day-to-day experiences of living in an emergency shelter and the process of rehousing, real and perceived barriers for families attempting to reestablish stable housing, and the impact of homelessness on families' overall health and well-being.	Service agency in Detroit, Michigan providing emergency financial assistance, job training, fiscal planning, life skills classes, and a wide array of social services to homeless families. Between April 1, 2010, and January 31, 2011, the agency enrolled 159 new families with 334 children.	Feminist participatory action research using focus groups to capture the experiences of homeless mothers or caseworkers	Qualitative	Purposive approach through the agency's caseworkers and through a Life Skills Support Group that met every other week. Potential mother participants were invited by a trusted caseworkers who knew the inclusion criteria and the participants' ability to act as key informant.	6 caseworkers and 21 homeless mothers allowing for four groups of 7 participants each.	Mothers were included if they were currently homeless, female, 18 years of age or older, and residing with, and caring for, at least one dependent child. Caseworker participants needed to be currently employed, either part- or full-time, at the study agency and actively engaged in providing supportive services to families experiencing homelessness	Maternity	Housing, Social care, Mental health	Emergency shelter; Informal Support/help-seeking Family
**Gultekin (US)**	Mothers	< 1yr	Journal/ Research article	Investigating the feasibility and acceptability of CENI-TF (clinical ethnographic narrative intervention) in helping mothers experiencing homelessness disclose and make meaning of trauma in their lives and their readiness or desire for help-seeking.	Urban family shelter in Detroit that provides emergency, transitional, and permanent supportive housing services to families.	Clinical ethnographic narrative intervention (CENI-TF). A "lifeline map" was used to identify the "high" and "low" points throughout women's life and key relationships; a body map to identify physical experiences of discomfort or pain, and a card sort to aid in recognizing thoughts and feelings associated with life events.	Qualitative	Participants were recruited from the family shelter, using flyers and visits by the PI visited to talk to potential participants about the study and answer questions. Participants self-selected to the study.	13 mothers in the participating family shelter were interviewed over four time points between April and September 2016. Twelve of the 13 participants attended all four interviews; one participant left the shelter related to a substance abuse disorder relapse prior to her fourth interview.	Women's age ranged from 22 to 59 (average 33.8 years). 12 of the mothers identified as African American or Black, 1 as Caucasian or White. 11 of the participants were single, 2 married and were joined in the shelter by their male partners. 1 participant was a grandmother who had taken custody of her grandchildren, and 2 were pregnant at the time of their interviews. All 13 participants had children, ranging from one to five children (an average of 1.8 children) with an average age of 10.3 years. 3 participants identied as disabled, and five participants reported a history of substance use disorders. One mother had Child Protective Services (CPS) involvement within the past 3 months, and all except one parent (not the mother with CPS involvement) had custody of their dependent children while in the shelter.	Race/Ethnicity,Pregnancy/ Maternity	Mental Health (trauma), Housing	Emergency Shelter; resilience and spirituality
**Hanley (Canada)**	Immigrants/ Refugee		Journal/ Research article	Investigating how health intersects with the experience of housing insecurity and homelessness for migrant women.	Montreal	Exploratory, qualitative study, using semi-structured, open- ended interviews.	Qualitative	Women were recruited in public places and in collaboration with community organisations (women's centers, homeless shelters, crisis centers, domestic violence shelters, immigrant settlement agencies and ethnic associations).	26 adult newcomer women and 5 key informants working in four local women's shelters	Not stated. They self-identified as having experienced housing insecurity and efforts were made to recruit a diversity of immigration statuses, age, physical and mental ability.	Race/Ethnicity	Mental Health, Physical Health, Housing	Healthcare Services and how they relate to housing insecuirty; discussion on self-reliance, faith, and social networks
**Henry (Australia)**	Immigrants/ Refugee	< 1yr		investigating the experiences and impacts of TFDV (technological-facilitated domestic violence) among immigrant and refugee women in Australia, and to understand their help-seeking pathways and sources of support.	Australia	interpretivist epistemology and qualitative research design; using a discussion guide	Qualitative	Participants were recruited on social media (e.g., Facebook, Gumtree, and Twitter), through victim support and advocacy stakeholder groups (e.g., DFV ser- vices), as well as on community and university noticeboards. Stakeholders were recruited through internal networking, online searches, and snowballing techniques	29 interviews with immigrant and refugee women between March–May 2018; 20 stakeholders from 12 different frontline organisations who support women experiencing TFDV, such as domestic violence, legal, and health services.	Women, 18 years or older, from a migrant background, and had experienced threats or abuse through technology, such as social media, phone/text, or sharing of private photos.	Race/Ethnicity	DIV/IPV	Statutory (Social Service/ Social Care, Healthcare Services: Primary Care); (DV) Shelter; Informal Support/help-seeking, Family, Friends, Community, Online
**Kiamanesh (Norway)**	Immigrants/ Refugee		Journal/ Research article	Exploring the needs of women with immigrant backgrounds when escaping violence, what help these women needed when fleeing violence and leaving their home, what service providers they contacted, and what experiences they had with service providers in relation to their need for support.	Shelters located in different municipalities in eastern Norway and in Oslo.	Phenomenological approach using qualitative interviews with women who were temporarily living either alone or with their children at shelters for abused women. Interviews were analysed through several stages defined by Smith et al. (2009).	Qualitative	Participants were recruited in collaboration with the leaders the shelters	28 interviews with men (1), women (23), and children (4) between 2016 and 2017	12 were living in shelters at the time of the interview, 2 had recently moved. The remaining 4 had been in touch with a shelter over several years but were not currently living there. All but two of the women had between one and four children aged between 2 and 21. Their educational backgrounds ranged from having completed primary school and high school to four or more years of study at university. Most women were divorced, legally separated, or in the process of separation at the time of the interview.	Race/Ethnicity	DIV/IPV	Statutory (Police); (DV) Shelter
**Langton (Australia)**	Women (not fitting any other category)	1 to <5	Report	Investigating the safety of Aboriginal and Torres Strait Islander women and their engagement with police, service providers and courts and the barriers to and enablers of access to support services and the justice system when experiencing domestic abuse and violence.	two fieldwork sites (Mildura and Albury–Wodonga)	The study was informed by social anthropology, medical anthropology, cultural epidemiology and Indigenous literature, using participant observation between October 2019 and November 2019, semi-structured interviews, and focus groups. The qualitative data was informed by quantiative background data.	Mixed Methods	Participants were recruited purposively to provide information-rich, in-depth data for analysis	97 participants, including Aboriginal victims and service providers across both field sites: 27 individual interviews and 22 focus groups (with a total of 70 participants); 31 participants in Mildura, 61 in Albury– Wodonga and 5 in other locations.	Participants were across different sectors, including health, justice, Aboriginal community-controlled, family violence, housing and hoemlessness. Further demographics of research participants was not given.	Race/Ethnicity	DIV/IPV, Mental Health, Physical Health	Specialist service (women), Statutory (Police, Social Service/ Social Care, Healthcare services, Criminal Justice), (DA) Shelters; Family networks

**Lopez (US)**	Women (not fitting any other category)	1 to <5	Journal/ Research article	Exploring the dynamic between compassion and punishment for highly stigmatised women's everyday life worlds with respect to a US health care safety and how this population is configured in an assemblage which results in multiple experiences of violence and harm.	Tenderloin and Mission Districts of San Francisco (in residential hotels, at community-based service sites, in community clinics, at the county hospital, on street corners, on buses, in coffee shops or fast food restaurants, while running errands, and while strolling around the neighborhoods)	Ethnographic study; ethnographic participant observation	Qualitative	Participants were recruited in residential hotels, at community-based service sites, in community clinics, at the county hospital, on street corners, on buses, in coffee shops or fast food restaurants, and other public spaces in the city.	30	Women were unstably housed and used drugs. Their ages ranged from 19 to 55. Interviews were followed up as possible between 2013 and 2016.	Race/Ethnicity,Gender Reassignment	Physical Health/ Primary Care, Housing, Substance use	Statutory (Police, Healthcare Services, Legal aid/ Criminal Justice, Benefits)
**Lyons (Canada)**	Women (not fitting any other category)	< 1yr	Journal/ Research article	Investigating experiences of accessing women-specific health and housing services among trans women and two-spirit persons in a downtown neighborhood of Vancouver, Canada.	Health and housing services in Vancouver's Downtown Eastside, which arose in response to soaring HIV rates and overdose related deaths in the 1990s and the continued violence against sex workers. The area includes services that are exclusively for women, including housing shelters, supported housing buildings, and drop-in centers that offer low threshold, minimal barrier services, and harm reduction programs.	Study is part of a larger qualitative project. Interviews which were analysed using a participatory analysis approach with two participants hired as research assistants.	Qualitative	Participants were recruited from three open prospective cohorts of individuals who use drugs (The At-Risk Youth Study, Vancouver Injection Drug Users Study, and AIDS Care Cohort to Evaluate Access to Survival Services) and an open prospective cohort of sex workers (An Evaluation of Sex Workers Health Access).	32 participants who identified as trans women and two-spirit individuals who had accessed women-specific health and/or housing services	All 32 participants had been assigned male sex at birth; not all identified as women and many used more than one category to describe their gender. 16 participants identified as transgender, 8 as women, 7 as transsexual, and 6 as two-spirit. Participants ranged in age from 23 to 52 years of age, with an average age of 40.3 years. Twenty-two (68.8%) participants identified as being of Indigenous ancestry (First Nations or Me´tis), seven (21.9%) identified as White and three identified as Filipino, Asian, and ''other'' visible minority respectively. Fifteen participants (47.9%) identified as heterosexual, seven (21.9%) as gay, five (15.6%) as bisexual, two (6.3%) as two-spirit, two (6.3%) as asexual, and five (15.6%) did not report their sexual orientation.	Race/Ethnicity,Gender Reassignment	Physical Health (HIV), Housing, Substance use, Offending	(DV) Shelters, Women-specific services (housing or health services)
**Magill (UK)**	Immigrants/ Refugee	< 1yr	Journal/ Research article	Investigating the experiences of migrants and minoritisied women during the pandemic and barriers to support services and housing	UK	Electronic survey followed by semi-structured interviews	Mixed Methods	Participant's recruited through the author's network	4 Southall Black Sisters employees were interviewed thus adopting a purposive sampling method	No further demographics given.	Race/Ethnicity	DIV/IPV, housing	Emergency accommodation and housing
**Marti-Castaner (US)**	Mothers	< 1yr	Journal/ Research article	Exploring how mothers living in New York City in or near poverty and using public assistance programs manage to meet (or not) their families' needs while mobilising social supports and navigating public assistance programs during pregnancy and the first year of their child's life	New York City	Qualitative study part of an ongoing randomised clinical evaluation (RCT) of "Room to Grow", a program provisioning parents living in low-income households with social and material support. The RCT followed 317 mothers and babies living in NYC. The qualitative study was conducted as part of a larger evaluation; both control and treatment group participants were included in the qualitative study.	Qualitative	Participants were recruited from RCT sample. All participants who met inclusion criteria were eligible. Participants were approached individually by the study team.	20 semi-structured interviews between July and December of 2018	Participants were mothers of 10.5-month-old infants, aged 17 to 39 years. Roughly 70% of the sample was living in poverty, the remaining participants in near poverty just above the poverty line. Half of the participants were first-time mothers. 14 had completed at least a high school degree or equivalent. Nine out of twenty were living in shelters at the time of the interview. Five out of 20 had been victims of domestic violence since they became pregnant. Only two mothers were living with their partners at the time of the interview. Nine out of twenty had their children attending childcare. All were receiving at least two forms of public assistance supports. Nine had recently received housing assistance, although not all were using it yet. A greater number of participants living in shelters received public assistance, demonstrating the vulnerability of this group. Nearly 90% of the sample was Black or Latinx. All women in the study qualified for Medicaid during their pregnancy and none of them reported healthcare expenses during their pregnancy and birth.	Race/Ethnicity,Pregnancy/ Maternity	Social care (safety net programs), Child welfare; subsistence needs; housing	Statutory Services (Housing Authorities); public assistance/welfare; Food needs; informal support; employment
**Mostowska (Poland)**	Older Women	1 to <5	Journal/ Research article	Presenting Polish research on women's homelessness and its theoretical underpinnings, conceptualisations of women's homelessness, and analysing narratives of older women in shelters	fieldwork in Warsaw, the capital of Poland and its largest city, and in the Podkarpackie region, a south-eastern province characterized by high unemployment and poverty, as well as one of the most traditional and Catholic regions in the country	Survey data, policy documents, interviews with experts and representatives of services, as well as individual interviews with women in homelessness crisis.	Mixed Methods	Participants were recruited through shelter managers and snowball sampling	44 women and 40 experts	local and state officials engaged in making and implementing homelessness and housing policies; lawyers with expertise in human rights (both non-governmental organizations and state institutions); managers and staff working in shelters, single mother homes, crisis centres, training flats; street workers and representatives of women's organisations dealing with issues of domestic violence. Ages ranged from early twenties to 86. The women experiencing domestic violence were over 48 years old and living in homeless shelters in the Podkarpackie province at the time. Most of them had adult children.	Age	DIV/IPV, Housing	mixed- and women- only shelter, night shelters; DA centre; police; social support
**Oliver (Canada)**	Adolescent; Young People 18-25		Journal/ Research article	Investigating the life narratives of young women, how they define their own health, what health issues they are experiencing, what services they use in seeking to care for themselves, and which factors they perceive as having influenced their access to health services, overall health and well-being.	Toronto, Canada	Multiple–case study approach to analyse the life history narratives of eight young women living in exceptional circumstances and participant observation. Coding and analysis of data by using the comparative method outlined by Strauss and Corbin (1990).	Qualitative	Women were recruited from shelters and from street visits to those who were not sheltered	8 women	women represented a range of races, sexualities, and ages. No more demographic data given.	Age	Physical Health/ Primary Care, Sexual health	Statutory (Healthcare Services: Primary Care and Sexual health)
**Quinn (US)**	Mothers	< 1yr	Journal/ Research article	Examining the role of permanent supportive housing in the lives of HIV-positive mothers and their children to improve the case management and supportive services available in supportive housing programs.	project develped with non-profit agency in the Midwest, providing safe and affordable housing for low-income individuals living with HIV/AIDS. Families in these programs are provided with permanent supportive housing, case management services, and wrap-around services as needed, including referrals to substance abuse treatment, counseling services, employment training, benefits acquisition, and health care. The program uses a Housing First and harm reduction model.	In-depth semi structured interviews with housing participants, housing case managers and program managers.	Qualitative	Participants were recruited via a flyer distributed during regular home visits through their case manager	10 in-depth qualitative interviews with female caregivers in permanent family supportive housing, 5 interviews with housing case managers and 1 program manager	Participants were housing residents with their families, HIV-positive and have been in the housing program from 1 to 12 years, with an average of 6 years. 9 of the women were African American and 1 was Latina. All of the women had children residing with them, and 1 participant was also living with her grandchild, ages ranging from 11 months to 26 years old. The 4 supportive housing program staff members were female and had been working as a family housing case managers from 1 to 5 years, although 3 had worked as housing case managers for other programs or with other agencies for longer.	Maternity	Mental Health, Physical Health/ Primary Care, Housing	Statutory (Healthcare Services; Care, Housing Authorities); Case manages at shelter

**Ruttan (US)**	Adolescent; Young People 18-25	1 to <5	Journal/ Research article	Determining the impact of becoming pregnant, assets used by the young women as they survived on the street and to transition out of homelessness.	Edmonton, Alberta	Qualitative approach. An assets model for homeless youth based on the Minnesota-based Search Institute's 40 Youth Assets model (Benson, Leffert, Scales, & Blyth, 1998) was developed from the data and used to explore and support women's growth based in positive experiences. Data included a demographic fact sheet, detailed summaries of each interview, transcripts of specific interviews, and life timelines.	Qualitative	Participants were recruited through contact with service agencies and other study participants. Sampling was purposive to select ''expert knowledge''	18 homeless mothers	Participants' ages ranged between 18 and 26, with an average age of 21. Living on the streets was initiated between ages 12 and 18, with most in the 14 to 16 age group. One half of the 18 participants were Aboriginal and the other one half of Euro-Canadian backgrounds. Self-reported reasons for initiating homeless included family tensions, the role as family scapegoat, a parent's death, abuse by parents and/or other relatives, relationships with boyfriends, and substance abuse. Several young women pointed out that they had actually left home for foster care at a much earlier age. The ''length of time homeless'' separated into two groups; most participants were homeless for a 3- to 5-year period, with a smaller group of approximately one year or less. Twelve of the participants had at least one child, and several had three or four. For those with larger numbers of children, their first child was either placed with a family member or in adoptive homes.	Pregnancy/Maternity; Age	Housing; Substance use; Parenting-related stress	Statutory (Healthcare Services: Prenatal, Housing Authorities; Social agencies); self-reliance
**Schmidt (Canada)**	Women (not fitting any other category)	1 to <5	Journal/ Research article	Exploring the trajectory of northern women's homelessness, their experiences accessing services, and suggestions for improving services	Iqaluit, Yellowknife, Whitehorse	Based on "Repairing the Holes in the Net", a 2-year, multilevel action research project to inform culturally appropriate and gender-specific services for homeless and marginally housed northern women	Qualitative	Women were recruited at community-based services using posters and with assistance from service providers.	61 interviews across services (7 in Iqaluit, 15 in Yellowknife and 19 in Whitehorse)	Age ranged between 19-56, Mean age was 38.4, 22 (36%) identified as Aboriginal/First Nation, 20 *33%) as Inuit, 7 (11% as White), and 12 did not mention their race. 74% of the children had children in their care. Women reported using 3-14 types of services (mean 7.3)	Race/Ethnicity,Pregnancy/ Maternity	Mental Health, Housing, Social exclusion	Statutory (Police, Social Service/Social Care, Healthcare Services: Mental Health, Housing Authorities)
**Slesnick (US)**	Mothers	< 1yr	Journal/ Research article	Assessing domain-specific treatment desires among a particularly at-risk population, substance abusing homeless mothers, as well as the correspondence between treatment desires and problem severity.	Based in a family shelter in a large Midwestern city. The local family shelter provides emergency shelter and critical services for homeless families from several townships adjacent to a major Midwestern city. The shelter director reports that annually, nearly 900 families seek emergency shelter.	The study was part of a multi-stage study funded by the National Institute on Drug Abuse with substance abusing homeless mothers of young children in a large Midwestern city. Data was collected using interviews and self-administered questionnaires, assessing age, race/ethnicity, and childhood sexual and physical abuse experiences.	Quantitative	Homeless mothers were recruited as a sample of convenience through a local family shelter. The shelter staff identified potentially eligible mothers and contacted the project coordinator to schedule an initial assessment interview.	102	Participants lacked a fixed, adequate overnight residence, met DSM-IV criteria 20 for psychoactive substance use or alcohol disorder, had a biological child between the ages of 2 to 6 years in their physical custody, were currently residing at a temporary homeless shelter for families. 71.6% of the homeless mothers in the sample were African Americans. On average, the homeless mothers were 27.01 years old, had 2.82 children, and 11.67 years of education. Childhood physical or sexual abuse was reported by 65.7% and 56.9% of the homeless mothers, respectively. They had been currently homeless for 60 days on average.	Pregnancy/Maternity	Mental Health, Physical Health, Substance use; Parenting	Based in family emergency shelter; looking at treatment desires for substance use, physical health, and childhood sexual abuse; acess too counseling and primary care
**Stylianou (US)**	Women (not fitting any other category)	< 1yr	Journal/ Research article	Exploring informal support for survivors of Domestic Abuse and violence, how social support networks are maintained and/or strained when residing in a DV emergency shelter	The study is part of a longitudinal needs assessment conducted by one large urban nonprofit operating six DV emergency shelter programs in the northeast United States. Data for this study utilised responses from the Time 1 qualitative portion of the interviews.	Three rounds of interviews. In-person baseline interviews within the first 2 weeks of entering the shelter. Around four-and-a-half and nine-and-a-half months after entering shelter, the evaluators conducted the second and third interviews. The interviews were analysed conducting a quantitative content analysis	Qualitative	Participants were recruited through shelter staff, distributing flyers to residents as they entered shelter. Researchers also attended orientation groups at the shelters to explain the needs assessment project.	83 residents completed in-person baseline interviews within the first 2 weeks of entering the shelter.	Of the 83 residents who completed the baseline interview, 76 residents (92%) responded to the questions regarding social support networks. The ages of the participants ranged from 19 years to 54 years, with an average age of 28.6 years. The majority of the participants identified as female (n = 75) and as straight/heterosexual (n = 68). All participants were individuals of color with 30 participants identifying as African American/Black, 31 participants as Hispanic/Latina, nine participants as multiracial, two participants as West Indian, one participant as Caribbean, and one participant as Native American. Two participants did not identify their race/ethnicity. The majority of participants (n = 66) had minor children	Race/Ethnicity	DIV/IPV	(DV) Shelters, Informal Support/help-seeking, Family; Friends
**Szanajder-Murray (US)**	Mothers		Journal/ Research article	Exploring experiences of homeless substance-abusing mothers with service providers and how they hope to be treated by service providers	Homeless family shelter in large Midwestern city	Phenomenological research design, using computerised diagnostic tool to determine formal eligibility of mothers and focus groups. The diagnostic tool (CDIS) is a structured comprehensive psychiatric interview containing 263 items based upon DSM- IV criteria and is administered to women by a computer. Focus groups were semistructured and explored four general topics.	Qualitative	Women were recruited to participate in the focus groups through a local homeless family shelter. After ensuing eligibility criteria and obtaining informed consent, women completed a 3-hour assessment battery with a research assistant.	28 women	Participants had physical custody of a biological child between the ages of 2 and 6 years, met diagnostic and Statistical Manual of Mental Disorders, criteria for a substance use or alcohol use disorder, and were currently residing in a local family emergency shelter. The majority of the mothers were African American (60.8%), 32.1% were White, and 7.1% reported Mixed ethnicity. Their ages ranged from 18 to 40 years old, with an average age of 29.2 years old (SD = 6.4). The mothers had an average of 3.1 children (SD = 1.68), whose ages ranged from newborn to 18 years old; additionally, 3 mothers were currently pregnant. Eight (28.6%) of the mothers reported having children removed from their custody; 4 of these mothers still did not have custody of all of their children.	Pregnancy/Maternity	Substance use; housing	Service providers generally; not further specified
**Tegan (US)**	Pregnant Women	< 1yr	Journal/ Research article	Identifying unmet needs related to maternal and infant health of the women currently residing at a homeless/emergency shelter	Shelter in Milwaukee, Wisconsin, considered the most segregated city in the United States, and one of the top 10 most impoverished big cities in the country	Explorative study, using two 1-hour focus groups to conduct a formal assessment to identify unmet needs related to maternal and infant health of the women currently residing at a homeless/emergency shelter.	Qualitative	Women were recruited through the Milwaukee Women's Center. Flyers inviting shelter residents to attend one of two focus groups were posted in the common living areas. Shelter staff announced the time and location of each focus group and invited residents to attend	13 women participated in each session	No specific personal or demographic information was collected from the focus group participants due to the sensitive nature of working with the homeless population and women who have experienced domestic violence. 90% of participants volunteered that they had been pregnant before.	Pregnancy/Maternity	Physical Health/ Primary Care	needs assessement within shelter (access to physician, education, prenatal support and baby care)
**Teruya (US)**	Women (not fitting any other category)	1 to <5	Journal/ Research article	investigating disparities among homeless women by examining and identifying areas of health and health care needs where racial/ethnic groups may differ.	Sober-living shelters or through street outreach in or near the Southwestern area of Los Angeles, from 12 shelters or through street outreach in 2 additional areas of Los Angeles County: the San Fernando Valley (7 sites), and Pasadena (5 sites)	Data for the ''Decision-making Regarding Drug Use among Homeless Women'' study (also known as the Decision-making Study, DMS) were collected from 1994–1996.	Quantitative	A purposive sample of 1,344 homeless women was recruited (age 18 years or older) from 51 traditional or sober-living shelters or through street outreach in or near the Southwestern area of Los Angeles, and from 12 shelters or through street outreach in 2 additional areas of Los Angeles County. Participants were recruited through presentations provided by research staff to groups, or on an individual basis.	original sample was reduced to 1,331 women who identified themselves as African American, Latina, or white.	Two-thirds of the sample were current substance users (reported using an illegal drug and/or alcohol in the past three months). The sample roughly represented the racial/ethnic distributions of homeless women in the study area shelters. The demographic profile showed that a large majority of the sample was racial/ethnic minority—almost half were African American and 30% were Latina (Table 1). Typically, the women had experienced repeated episodes of homelessness, and had stayed in shelters. Their average age was 33 years and each averaged 2 children. The majority had not completed high school, only 1/10 worked full or part time, and almost 1/2 had been incarcerated. Overall, the sample was predisposed to poor health. Almost 1/3 of the women had been victimized, either physically or sexually, as children.	Race/Ethnicity	Physical Health/ Primary Care, Substance use; Mental Health	Statutory (Healthcare Services)
**Theobald (Australia)**	Pregnant Women	not mentioned	Journal/ Research article	Exploring barriers of homeless pregnant women when seeking access to housing and what support they need	Housing and pregnancy agencies in Metropolitan Melbourne, although one participant moved during pregnancy to a regional centre	Critical interpretation of lived experience accounts of homelessness and those of service providers.	Qualitative	Purposive sampling with participants recruited through housing and pregnancy agencies. Interviews were conducted with 14 cis-gender women who were or had been pregnant while homeless in the previous two years.	19 interviews and 8 focus groups with stakeholders (including policy practitioners and service providers) comprising 41 participants.	27 services participated (18 homelessness services, five community-based health services, two hospital-based health services, one community-based specialist support service, and one government agency). The women were aged between 21 and 36 years. Six were partnered, and eight were single. Twelve were born in Australia, including two Indigenous women and one culturally and linguistically diverse woman. The remaining two women had migrated from Middle Eastern countries	Pregnancy/Maternity	Sustance Use; Mental and Physical Health; Housing; Sexual Trauma	Primary care (prenatal); Healthcare; Housing

**Versey (US)**	Women (not fitting any other category)	< 1yr	Journal/ Research article	Examining how housing insecurity impacts the lives of lower-income Black women	Austin, TX; Baltimore, MD; Seattle, WA; New York, NY; Los Angeles, CA; and states (New York, California, Maryland, Texas, and Ohio)	Fighting Displacement Study (FDS) is a national qualitative study of women tenants and tenant-advocates (N = 80). Data were collected between 2020 and 2021, during the height of the COVID-19 pandemic.	Qualitative	Participants were identified by direct outreach and snowball sampling, based on advertisements and recruitment flyers circulated through word-of-mouth and social media. Requests were also sent to eviction diversion programs, legal aid attorneys, eviction prevention agencies, and mutual aid organizations.	78 but 4 women's narratives were chosen to highlight them themes that emerged	Nearly all of the women in the current study identified as Black women (n = 78). Participants' ages ranged from 18 to 75, and the mean age was 41. All women were renters or former renters (for participants temporarily unhoused).	Race/Ethnicit y	Mental Health, Physical Health/ Primary Care, Housing	Informal Support/help-seeking Family Friends
**Vidales (US)**	Immigrants/ Refugee	1 to <5	Journal/ Research article	Investigating the experiences of predominantly Mexican immigrant women as victims and survivors of domestic violence.	Shelter for battered women in Southern California	Ethnographic study in a shelter for battered women, including semi-structured interviews with professinals and 86 battered women who sought assistance from the shelter. Interviews were conducted in Spanish and a questionnaire eliciting demographic information and women's personal networks from the women was distributed. Each woman's biography and attendant experience was treated as a case, and the cases were then examined and combined using comparative pattern analysis	Qualitative	Participants were recruited through the shelter, with access facilitated for the researcher previous establishment of trust with staff members and some clients	86 women	Participants had predominantly Mexican immigrant backgrounds (86%). Most (73%) of the women reported being married to their abusers. In addition, 81% reported being Catholic and 82% stated they were primarily Spanish speaking. 93% of the women are immigrants; of these, 86% are Mexican immigrants. The remainder of the immigrant sample (14%) were from South America, Central America and the Caribbean. Only two women (7%) were born in the U.S. The women from Mexico averaged 12 years in the U.S., ranging from only six months to 28 years. Twenty-one percent of the respondents did not state a residency status. Of those who did respond, 37% stated they were not a legal resident of the U.S. The women in the study averaged 2.4 children. The mean age at which these women first reported being victimized by violence was 23.	Race/Ethnicit y	DIV/IPV	(DV) Shelters and providers providing support with DV; police; family; criminal justice
**Viergever (Netherlands)**	Immigrants/ Refugee	< 1yr	Journal/ Research article	Investigating how service users in the COSM programme conceptualided and experienced their own process of recovery	Three COSM shelters, a setting with a specific programmatic, social, medical and legal context that was established in the Netherlands to provide social and health services for trafficked people. COSM programme consists of three shelters, two for women and one for men, and at the time of this study had a total of 50 beds	Grounded theory approach following the stage of familiarization, open coding, axial coding and selective coding. In addition, some elements of narrative analysis were incorporated, by writing narratives about participants	Qualitative	Participants were recruited and screened by employees of the shelter; sometimes the primary author suggested participants based on observations in the shelter. Which service users would participate in the study was decided upon in discussion between the primary author and the employees of the shelter	29 interviews were conducted with 14 participants of non-Dutch nationality	The study population consisted of foreign, adults who had been trafficked and who resided in one of the three COSM shelters. Nine female and five male service users were interviewed. Their median age was 26. Nine study participants were from Africa, three were from Eastern Europe, one was from Asia and one from the Middle East. Seven participants had no children, five had children, and three were pregnant (total is not 14 because one had children and was pregnant). The me- dian time that the study participants had spent in the shelter prior to the first interview was 2.4 months.	Race/Ethnicit y	Sex trafficking; housing	Shelter; self-reliance and motivations
**Wagaman (US)**	LGBTQ*		Journal/ Research article	Investigating the service experiences of LGBTQ-identified young people, their experiences with services, and the perceived barriers to accessing LGBTQ-specific and general services.	Community-based organisation for LGBTQ-young people in a large, urban Southwestern city. The organization identified as a social support agency focused on increasing self-acceptance and empowerment among LGBTQ young people ages 14 to 24. The agency ran a youth center in the urban center of the city, as well as several satellite groups in suburban communities surrounding the city.	Descpriptive qualitative approach to knowledge development	Qualitative	Participants were drawn from a database of young people, ages 18 to 24, who attended the LGBTQ youth-serving organisation. 113 young people were identified within the selection criteria. The database was divided into subgroups based on race and then further subdivided by gender identity, leading to the recruitment of 15 participants	15	4 participants were African-American, 3 Latino/a, 2 Nativen American/First Nation, 4 White, 1 Asian, and 1 Multiracial. They defnied their gender identity as 6 Male, 8 Female, 1 Genderqueer, 11 Cisgender, and 4 Transgender	Age; Sexual Orientation	Mental Health, Housing, Employment, Legal aid, Substance use, Subsistence needs (food, clothes)	Basic needs services, school-based serivces, Statutory (Healthcare Services: Mental Health, Primary Care, Housing Authorities); LGBT-specific servixes
**Wilson (US)**	Women (not fitting any other category)		Journal/ Research article	Exploring the narratives of Black mothers to discover their experiences in seeking housing after leaving abusive relationshipswith a focus on housing instability and mental health.	Emergency IPV shelter in Baltimore City	qualitative descriptive narrative research design (pilot study)	Qualitative	Participants were recruited from the shelter. Convenience sampling and snowball sampling was utilised to recruit five study participants and other persons having necessary characteristics making them eligible for the sample.	5 women	Participants ranged in age from 21 to 40 years with a mean age of 28.4 years. Of the five participants, two had less than a high school education, three graduated from high school, and two had at least one semester of college education. All five had at least two children (one pregnant with her second child and one with three children). The children ranged in ages from2weeks to 18 years. Four of the women were currently employed, and one was receiving disability benefits and unable to work.	Race/Ethnicit y	DIV/IPV, Housing	(DV) emergency shelter, Employment Services, Mental Health care, Informal Support/help-seeking, Family and friends
**Wydall (UK)**	Older Women	< 1yr	Journal/ Research article	Sheding light on some of the contextual factors influencing help-seeking in later life and exploring professionals' perceptions as to why domestic abuse victims aged 60 years and over might disengage with statutory agencies	Wales, UK. Wales is perceived as a pioneer in the field of domestic abuse, developing highly innovative initiatives to increase the safety and choices for victim-survivors	interpretivist, discovery-orientated approach	Qualitative	Unclear	50 semi-structured interviews with practitioners	Interviews were conducted with practitioners and managers from 21 out of 22 local authorities (44), four regional police forces (4) and members of the Crown Prosecution Service Cymru (2).	Age	DIV/IPV, Housing	(DV) Shelters, Statutory, (Housing Authorities), Informal Support/help-seeking, Family, Friends, Community
**Yarbrogough (US)**	LGBTQ*	1 to <5	Journal/ Research article	Analysing transgender women's experiences of criminalisation and housing deprivation to conceptualize the co-production of poverty and inequality as the result of policing across institutional sites.	Sex worker, homeless, and transgender service and advocacy organisations in San Francisco.	Life and work history interviews and ethnographic observation. The study is part of a multi-year ethnographic study with 55 unhoused sex workers of all genders	Qualitative	Purposive sampling strategy for racial and gender diversity recruiting participants by word of mouth, posters advertising the study, and fliers distributed during nighttime outreach	23 transgender women	Participants had experienced housing deprivation and done criminalised informal economy work. The sample of transgender women included 8 Black women, 7 white women, 2 Latina women, 2 Native American/Indigenous women, and 4 women who identified as multi-racial.	Race/Ethnicit y,Gender Reassignment	Mental Health, Substance use, primary health (HIV), Housing	Statutory, (Housing Authorities; Police, Mental Health support and primary care) and other homeless service organisations including (DV) shelters, drug rehabilitation, and transitional housing programmes; Informal Support/help-seeking, Family, Friends, Community
**Abramovich (Canada)**	LGBTQ* youth	< 1yr	Journal/ Research article	Aims to understand the mental health and service needs among 2SLGBTQ+ youth experiencing homelessness across York Region, a suburban and semi-rural region in Ontario, Canada.	Situated in nine of York Region's municipalities.	Mixed methods using 1-on-1 interviews and qunatitative surveys; analysed using the Social Ecological Model for the qualitative componened and descriptive statistics for the survey.	Mixed Methods	Snowball and purposive sampling through electronic and hardcopy flyers circulated by study collaborators.	33 (although 5 of which were cisgender men)	13-26 years (mean = 18.84) self-identifying as LGBTQ+; experiening or at risk of homelessness including sofa surfing, living with parents but at risk to be evicted, emergency and/or domestic abuse shelters ; rough sleeping, or transitional or supported housing)	Sexual Orientation; Gender Reassignment	Mental Health; Primary Care	Healthcare Services

**Alessi (US)**	LGBTQ* youth	1 to <5	Journal/ Research article	Investigates how sexual and gender minority (SGM) homeless youth described and understood their victimization experiences occurring before they were homeless and those occurring after they were homeless and engaging in survival sex. And how these youths manifested resilience when living on the street. The article is linked to another included study (Greenfield)	NYC	Using secondary analysis of data obtained in an initial study; more focused on the needs of SGM youths. Content and thematic analyses	Qualitative (secondary)	Respondent-driven sampling (RDS) was used to recruit participants from the larger study who identified as transgender, non-binary, or gender expansive (e.g., genderqueer, androgynous) and reported active engagement in survival sex.	283 racially/ethnically diverse youth between the ages of 15 and 26 years (M = 19.6, SD = 1.28); 36% of which were female, 15% transender, and 3% queer or other	Participants identified their gender as male (47%), female (36%), transgender (15%), or queer and other (3%) and their sexual orientation as bisexual (37%), gay (23%), lesbian (15%), heterosexual (13%), or queer and other (13%). Thirty-seven percent identified as Black, 30% as multiracial, 22% as Latino/a, 5% as White, and 5% as another race.	Sexual Orientation; Gender Reassignment	Housing, Subsistence Needs, Employent	Housing; Employment; Self-care and resilience
**Kirkman (Australia)**	Mothers	< 1yr	Journal/ Research article	Explores women's experiences of being homeless with their children in Victoria, Australia, emphasising their mental health.	Youth family services in Melbourne, Australia	interviews and questionaire including demographic information, housing history and number of schools; thematic analysis	Qualitative	Mothers were recruited via their childred aged 5–12 years, 12 women were recruited who are currently supported or accommodated (with at least one other family member) by youth or family homelessness services but not, for ethical reasons, living in crisis accommodation. The children and their mothers were recruited through gate keepers	12 women (who between them had daily responsibility for 31 children)	Most women identified as Anglo-Australian, two identified as Greek-Australian, one as Aboriginal and one as English. Between them, the women had 38 children (including one grandchild), of whom 31 were living with the women. Most women were living in transitional supported accommodation and had been there for less than a year. During their children's lives women had experienced between 3 and 11 changes of residence, which had included hotels or motels, other varieties of emergency accommodation, refuges, sleeping rough or in cars, rooming or boarding houses, caravan, parks, and the homes of friends. Only one woman was living with a partner (her husband). Government benefits and allowances were the major source of income for almost all women completing the questionnaire. Most women reported limited sources of social support (such as from parents, siblings, or ex-partners) with the majority relying primarily on a social worker.	Motherhood	Housing; Mental health; Substance Use	Youth or family homelessness services; Mental Health Services; Self-help; Social Services
**Smid (US)**	Pregnant Women	< 1yr	Journal/ Research article	Examines how street youth exerience their pregenancy	Telegraph/Haste area in Berkeley. The research was primarily based at the Suitcase Clinic, a weekly, university-affiliated, student-run drop-in center for homeless youth providing a variety of free services, not primarily medical. The service is located in a church, but involves no religious activities. Youth can receive a meal and are offered medical, legal, social work, chiropractic, and acupuncture services. They are not required to engage with any services.	Interviews and participant observations, field work notes; grounded theory approach	Qualitative	Women were recruited through the agency and reported being homeless during a current or past pregnancy. Women were also approached if they appeared pregnant during participant-observation conducted by the first author who visited common "hang out" locations frequented by homeless youth.	21 interviews, 11 of which with the women alone, four with men and five with couples, one interview included two women	Eighteen youth identified themselves as White. One woman identified as herself as Irish-African American. The women reported greater levels of personal trauma than the men and left home at younger ages than the men. On average, the women also had less education. Four of the women were pregnant at the time. the others had been interviewed about a previous pregnancy. Five of the thirteen women had experienced multiple pregnancy, two reported 2 pregnany, one reported 3, and two reported 4 pregnancies. Of these 15 newborns, only three remained in their parents' custody. The remaining 12 newborns were removed from their birth mothers by social services and placed with grandparents, a former partner, or in foster care.	Motherhood/ Pregnancy	Mental illess, substance use, institutional care, pregnancy	Child Protection Services, Housing, Healthcare
