# Supplementary material for: A systematic narrative review of the research evidence of the impact of intersectionality on service engagement and help-seeking across different groups of women, trans women, and non-binary individuals experiencing homelessness and housing exclusion
Source: PLoS One. 2025 Apr 24;20(4):e0321300. doi: 10.1371/journal.pone.0321300 (PMC12021236; doi:10.1371/journal.pone.0321300)
Supplement: S3 Appendix — (PDF) [file pone.0321300.s003.pdf]

First Author, Context, Date	Agenda, UK, 2022*	Austin, UK, 2019	Baumann, US, 2018	Benbow, Canada, all papers*	Bimpson, UK, 2020*
Congruity between philosophical perspective and the methodology	No	No	Unclear	Yes	Yes
Congruity between the research methodology and RQ and objectives	Yes	Yes	Yes	Yes	Yes
Congruity between the research methodology and the methods	Yes	Yes	Yes	Yes	Yes
Congruity between methodology and representation/ analysis?	Yes	Unclear	Yes	Yes	Yes
Congruity between the research methodology and interpretation of	Yes	Yes	Yes	Yes	Yes
Statement locating the researcher culturally and theoretically?	No	No	No	Unclear	No
Influence of the researcher on the research, and vice-versa addressed?	No	No	No	No	No
Participants, and their voices, adequately, represented?	Yes	Yes	Yes	Yes	Yes
Research ethical according to current criteria or ethical approval?	Unclear	Unclear	Yes	Yes	Yes
Conclusions drawn in the research report flow?	Yes	Yes	Yes	Yes	Yes
	75%	60%	75%	85%	80%
Quality Appraisal	Medium	Medium	Medium	High	High
How valuable is the research?	High	High	High	High	High

* appraissed by two research team members independently

Boyd, Canada, 2018	Brais, Canada, 2019	Cardwell, UK, 2018	Dashora, USA, 2012*	Deal, USA, 2023	Dudley, UK, 2017	England, UK, 2021
Unclear	Unclear	No	Unclear	Yes	Yes	Yes
Yes	Yes	Yes	Yes	Yes	Yes	Yes
Yes	Yes	Yes	Yes	Yes	Yes	Yes
Yes	Yes	Yes	Yes	Yes	Unclear	Yes
Yes	Yes	Yes	Yes	Yes	Yes	Yes
No	Yes	No	No	Yes	No	Yes
No	No	No	No	No	No	Yes
Yes	Yes	Yes	Yes	Yes	Yes	Yes
Yes	Yes	Unclear	Yes	Yes	Unclear	Yes
Yes	Yes	Yes	Yes	Yes	Yes	Yes
75%	85%	75%	75%	90%	70%	100%
Medium	High	Medium	Medium	High	Medium	High
High	High	High	High	High	Medium	High

Glumbíková, Czech Republic, 2018	Gordon, UK, 2019	Greene, Canada, 2012	Greenfield, USA, 2021*	Gultekin, US, 2015	Gultekin, US, 2023
Yes	Unclear	Yes	Unclear	Yes	Yes
Yes	Yes	Yes	Yes	Yes	Yes
Yes	Yes	Yes	Yes	Yes	Yes
Yes	Yes	Yes	Yes	Yes	Yes
Yes	Yes	Yes	Yes	Yes	Yes
Yes	No	Yes	No	No	No
Unclear	No	No	No	Yes	No
Yes	Yes	Yes	Yes	Yes	Yes
Yes	Yes	Yes	Yes	Yes	Unclear
Yes	Yes	Yes	Yes	Yes	Yes
95%	75%	90%	75%	90%	75%
High	Medium	High	Medium	High	Medium
High	High	High	High	High	Medium

Hanley, Canada, 2019	Henry, Australia, 2021	Kiamenesh, Norway, 2019	Langton, Australia, 2020	Lopez, US, 2014*	Lyons, Canada, 2016	Magill, UK, 2022*
Yes	Yes	Yes	Yes	Yes	Yes	Yes
Yes	Yes	Yes	Yes	Yes	Yes	Yes
Yes	Yes	Yes	Yes	Unclear	Yes	Yes
Yes	Yes	Yes	Yes	Yes	Yes	Yes
Yes	Yes	Yes	Yes	Unclear	Yes	Yes
No	No	No	Yes	Yes	Yes	No
No	No	No	Unclear	No	No	No
Yes	Yes	Yes	Yes	Unclear	Yes	Yes
Yes	Yes	Yes	Yes	Unclear	Yes	Unclear
Yes	Yes	Yes	Yes	Yes	Yes	Yes
80%	80%	80%	95%	70%	90%	75%
High	High	High	High	Medium	High	Medium
High	High	High	High	Medium	High	High

Marti-Castaner, US, 2022*	Mostowska, Poland, 2020	Oliver, Canada, 2012	Quinn, US, 2015*	Ruttan, Canada, 2012	Schmidt, Canada, 2015
Unclear	Yes	Unclear	Unclear	Yes	Unclear
Yes	Yes	Yes	Yes	Yes	Yes
Yes	Yes	Yes	Yes	Yes	Yes
Yes	Unclear	Yes	Unclear	Yes	Yes
Yes	Yes	Yes	Yes	Yes	Yes
No	No	No	No	No	No
No	No	No	No	No	No
Yes	Yes	Yes	Yes	Yes	Yes
Yes	Yes	Unclear	Yes	Yes	Yes
Yes	Yes	Yes	Yes	Yes	Yes
75%	75%	70%	70%	80%	75%
Medium	Medium	Medium	Medium	High	Medium
High	High	Medium	Medium	High	High

Stylianou, USA, 2021	Sznajder-Murray, USA, 2011	Theobald, Australia, 2023	Versey, US, 2022*	Vidales, US, 2010	Viergever, Netherlands, 2019
Yes	Yes	Yes	Yes	Yes	Unclear
Yes	Yes	Yes	Yes	Yes	Yes
Yes	Yes	Yes	Yes	Yes	Yes
Yes	Yes	Yes	Yes	Yes	Yes
Yes	Yes	Yes	Yes	Yes	Yes
No	No	No	No	Yes	Unclear
No	No	No	No	No	Unclear
Yes	Yes	Yes	Yes	Yes	Yes
Yes	Yes	Yes	Yes	Yes	Yes
Yes	Yes	Yes	Yes	Yes	Yes
80%	80%	80%	80%	90%	85%
High	High	High	High	High	High
Medium	High	High	Medium	High	Medium

Wagaman, US, 2014	Wilson, US, 2015	Wydall, UK, 2017*	Yarbrough, USA, 2023	Abramovich, Canada, 2020	Alessi, US, 2021	Kirkman, Australia, 2015
Yes	No	Yes	Yes	Yes	Yes	Unclear
Yes	Yes	Yes	Yes	Yes	Yes	Yes
Yes	Yes	Yes	Yes	Yes	Yes	Yes
Yes	Yes	Yes	Yes	Yes	Yes	Yes
Yes	Yes	Yes	Yes	Yes	Yes	Yes
Yes	No	No	Yes	No	No	No
Yes	No	No	No	No	Unclear	No
Yes	Yes	Yes	Yes	Yes	Yes	Yes
Yes	Yes	Yes	Yes	Yes	Yes	Yes
Yes	Yes	Yes	Yes	Yes	Yes	Yes
100%	70%	70%	90%	80%	85%	75%
High	Medium	Medium	High	High	High	Medium
High	Medium	High	High	High	High	High

Smid, US, 2010
Unclear
Yes
Yes
Yes
Yes
Yes
No
Yes
Yes
Yes
85%
High
High

Author, Context, Year	Ben-Porat, Israel, 2020*	Duff, Canada, 2015*	Slesnick, US, 2014	Teruya, USA, 2010*
Was the exposured measure in valid and reliable way?	Yes	Yes	Yes	Yes
Were objective, standard criteria used for measurement of the condition?	Yes	Yes	Yes	Yes
Were confounding factors identified?	No	No	No	Yes
Were the study subject and the setting described in detail?	Yes	Yes	Yes	Unclear
Were strategies to deal with confounding factor stated?	No	No	No	Yes
Was the outcome measured in a valid and reliable way?	Yes	Yes	Yes	No
Was appropriate statistical analysis used?	Yes	Yes	Yes	Yes
	70%	70%	70%	78%
Quality Appraisal	Medium	Medium	Medium	High
How valuable is the research?	Medium	Medium	High	High
