# Supplementary material for: A systematic narrative review of the research evidence of the impact of intersectionality on service engagement and help-seeking across different groups of women, trans women, and non-binary individuals experiencing homelessness and housing exclusion
Source: PLoS One. 2025 Apr 24;20(4):e0321300. doi: 10.1371/journal.pone.0321300 (PMC12021236; doi:10.1371/journal.pone.0321300)
Supplement: S4 Appendix — (PDF) [file pone.0321300.s004.pdf]

Screened Studies				
No	Item	Title	Screen on Title & Abstract	Screen on Full Text
1	(2008) (ID:87857041)	Helping unaccompanied asylum-seeking children	-EXCLUDE on date (2010)	
2	"Stuck in Limbo" Experiences... (Shelter) (ID:88019158)	"Stuck in Limbo" Experiences of women in Greater Manchester on surviving domestic abuse, homelessness and a housing system not working for them	-INCLUDE on title & abstract	-EXCLUDE on intervention (intersectionality)
3	"We have a lot of home... (Lusambili) (ID:87851443)	"We have a lot of home deliveries" A qualitative study on the impact of COVID-19 on access to and utilization of reproductive, maternal, newborn and child health care among refugee women in urban Eastleigh, Kenya	-EXCLUDE on country (High-Income)	
4	[Anonymous] (2019) (ID:87853113)	Research on multimorbidity in primary care. Selected abstracts from the EGPRN meeting in Tampere, Finland, 9-12 May 2019 All abstracts of the conference can be found at the EGPRN website: www.egprn.org/page/conference-abstracts	-EXCLUDE on population (no housing precarity)	
5	#HEALTHNOW (2022) (ID:87856924)	#Healthnow literature review update: how has patient experience changed for people who are homeless?	-EXCLUDE on population (no gender focus; women population <50)	
6	5.Q. Workshop: Participatory... (ID:87851594)	5.Q. Workshop: Participatory health research with refugees: Voices, experiences, and methodologies.	-EXCLUDE on population (no gender focus; women population <50)	
7	99 Barriers of Follow... (Tay) (ID:87851693)	99 Barriers of Follow Up within the Burn and Wound Population.	-EXCLUDE on intervention (service engagement/helpseeking behaviour)	
8	A (2021) (ID:87851530)	Right to Health in the International Legal System of Human Rights at the Universal and Regional Levels	-EXCLUDE on intervention (service engagement/helpseeking behaviour)	
9	Aagaard (2014) (ID:87848970)	Predictors of frequent visits to a psychiatric emergency room: a large-scale register study combined with a small-scale interview study.	-EXCLUDE on population (women)	
10	Abbs (2020) (ID:87848330)	Risk Factors for Falls in Older Adults Experiencing Homelessness: Results from the HOPE HOME Cohort Study.	-EXCLUDE on population (women)	
11	Abdel-Baki (2019) (ID:87848405)	Improving mental health services for homeless youth in downtown Montreal, Canada: Partnership between a local network and ACCESS Esprits ouverts (Open Minds), a National Services Transformation Research Initiative.	-EXCLUDE on population (women)	
12	Abdellatif (2021) (ID:87857498)	Breaking the mold: Working through our differences to vocalize the sound of change	-EXCLUDE on population (no housing precarity)	
13	Abdelmoneium (2010) (ID:87851384)	Policy and practice: Non-governmental organisations and the health delivery system for displaced children in Khartoum, Sudan.	-EXCLUDE on country (High-Income)	
14	Abdu (2013) (ID:87849057)	Disease pattern and social needs of street people in the race course area of Kano, Nigeria.	-EXCLUDE on country (High-Income)	
15	Abdulmalik (2019) (ID:87850738)	Boko Haram insurgency and Nigeria's mental health response.	-EXCLUDE on country (High-Income)	
16	Abel (2013) (ID:87848995)	The impact of an oral health program on domestic violence survivors within community shelters.	-INCLUDE on title & abstract	-EXCLUDE on intervention (intersectionality)
17	ABEL (2019) (ID:87857385)	Preventing child sexual abuse: screening for hidden child molesters seeking jobs in organizations that care for children	-EXCLUDE on population (no housing precarity)	
18	Abel (2020) (ID:87858038)	"Difficult Roads Lead to Beautiful Places:" Newcomer Youth and Caregiver Experiences in U.S. Elementary Schools	-EXCLUDE on population (women)	
19	Abji (2021) (ID:87857941)	"HONOUR"- BASED VIOLENCE AND THE POLITICS OF CULTURE IN CANADA: ADVANCING A CULTURAL ANALYSIS OF MULTI-SCALAR VIOLENCE	-EXCLUDE on intervention (service engagement/helpseeking behaviour)	
20	Abok (2015) (ID:87853402)	HIV Prevalence in Vulnerable Children Living in Jos, Plateau State, North-Central Nigeria	-EXCLUDE on country (High-Income)	
21	Abraham (2017) (ID:87848639)	Competitive Employment Outcomes Among Veterans in VHA Therapeutic and Supported Employment Services Programs.	-EXCLUDE on population (women)	
22	Abraham (2020) (ID:87858049)	Resilience, Quality of Life, and Discrimination Among Individuals with a Criminal Record	-EXCLUDE on population (women)	
23	Abramovich (2020) (ID:87963018)	A transgender refugee woman experiencing posttraumatic stress disorder symptoms and homelessness	-INCLUDE on title & abstract	-EXCLUDE on intervention (service engagement/helpseeking behaviour)
24	Abrams (2020) (ID:87963077)	Considerations for employing intersectionality in qualitative health research	-EXCLUDE - but review for literature<br>-EXCLUDE on intervention (service engagement/helpseeking behaviour)	

25	Abstracts from the Society... (2012) (ID:87851958)	Abstracts from the Society for Clinical Trials Annual Meeting, Miami, May 21-23, 2012	-EXCLUDE on intervention (service engagement/helpseeking behaviour)
26	Abstracts, Oral (Listed... (2016) (ID:87858015)	Abstracts, Oral (Listed Alphabetically by Author's Last Name)	-EXCLUDE on evidence and form (evidence not in written form or presented as research output)
27	Abstracts, Oral Presentations... (2018) (ID:87858092)	Abstracts, Oral Presentations for Qualitative Health Research Conference, 2017	-EXCLUDE on evidence and form (evidence not in written form or presented as research output)
28	Abstracts, Oral Presentations... (2019) (ID:87858115)	Abstracts, Oral Presentations for Qualitative Health Research Conference, 2018	-EXCLUDE on population (women)
29	Abstracts, Poster Presentations... (2017) (ID:87858026)	Abstracts, Poster Presentations for Qualitative Health Research Conference, October 2016	-EXCLUDE on evidence and form (evidence not in written form or presented as research output)
30	Abul (2022) (ID:87851568)	Family planning knowledge, attitude and practice among Rohingya women living in refugee camps in Bangladesh: a cross-sectional study	-EXCLUDE on country (High-Income)
31	Aby (2023) (ID:87851669)	Long-term clinical outcomes of patients with COVID-19 and chronic liver disease: US multicenter COLD study.	-EXCLUDE on population (women)
32	Access to Care During... (2020) (ID:87851493)	Access to Care During a Global Health Crisis	-EXCLUDE on evidence and form (evidence not in written form or presented as research output)
33	Achat (2010) (ID:87852901)	General health care service utilisation: where, when and by whom in a socioeconomically disadvantaged population	-EXCLUDE on population (women)
34	Aching (2018) (ID:87850800)	Role of a support network for refugee mothers.	-EXCLUDE on country (High-Income)
35	Ackerley (2017) (ID:87963155)	Applying an intersectional lens to sexual violence research and practice	-EXCLUDE on population (people aged under 18 years)
36	Acquah-Hagan (2022) (ID:87851746)	Availability and Affordability of Primary Health Care Among Vulnerable Populations in Urban Kumasi Metropolis: Family Health Perspective.	-EXCLUDE on country (High-Income)
37	Adair (2016) (ID:87848738)	Housing Quality in a Randomized Controlled Trial of Housing First for Homeless Individuals with Mental Illness: Correlates and Associations with Outcomes.	-EXCLUDE on population (women)
38	Adair (2017) (ID:87848697)	Outcome Trajectories among Homeless Individuals with Mental Disorders in a Multisite Randomised Controlled Trial of Housing First.	-EXCLUDE on population (no gender focus; women population <50)
39	Adams (2011) (ID:87851712)	HIV Risk After Release From Prison: A Qualitative Study of Former Inmates.	-EXCLUDE on population (women)
40	Adams (2013) (ID:87851689)	Ten-year hip fracture incidence rate trends in a large California population, 1997--2006	-EXCLUDE on population (no housing precarity)
41	Adams (2018) (ID:87848583)	Willingness to pay for small-quantity lipid-based nutrient supplements for women and children: Evidence from Ghana and Malawi.	-EXCLUDE on country (High-Income)
42	Adams (2021) (ID:87963320)	"A good mother": Impact of motherhood identity on women's substance use and engagement in treatment across the lifespan	-EXCLUDE on population (no housing precarity)
43	Adams (2022) (ID:87848157)	A Qualitative Study Exploring Access to Mental Health and Substance Use Support among Individuals Experiencing Homelessness during COVID-19.	-EXCLUDE on population (no gender focus; women population <50)
44	Adamson (2015) (ID:87850554)	The dance to death : the aesthetic experience of dying	-EXCLUDE on population (no housing precarity)
45	Adamus (2022) (ID:87848151)	A mirror-image analysis of psychiatric hospitalisations among people with severe mental illness using Independent Supported Housing.	-EXCLUDE on population (no housing precarity)
46	Addison (2022) (ID:87857693)	Mental health outcomes in formerly incarcerated Black men: A systematic mixed studies review	-EXCLUDE on population (women)
47	Adkins (2021) (ID:87851893)	Leadership in the Shadow of Jim Crow: Race, Labor, Gender, and Politics of African American Higher Education in North Carolina, 1860-1931	-EXCLUDE on intervention (service engagement/helpseeking behaviour)
48	Adler (2022) (ID:87848152)	Association of Problematic Anger With Long-term Adjustment Following the Military-to-Civilian Transition.	-EXCLUDE on population (women)
49	Adorno (2013) (ID:87851142)	Predisposing, enabling, and need factors associated with addiction treatment among Massachusetts Puerto Rican drug users.	-EXCLUDE on population (women)
50	Adult Korean Americans:... (2005) (ID:87857157)	Adult Korean Americans: their attitudes toward seeking professional counseling services	-EXCLUDE on population (no housing precarity)

51	ADVISORY (2019) (ID:87856982)	Drug-related harms in homeless populations and how they can be reduced	-EXCLUDE on population (no gender focus; women population <50)	
52	Aersolon (2021) (ID:87857637)	Making the Social Actually Work: A Community-Needs Assessment of Rural Transgender Californians	-EXCLUDE on evidence and form (evidence not in written form or presented as research output)	
53	Affleck (2018) (ID:87858034)	Invisible Wounds: Trauma, Gender, and the Lived Experience of Sri Lankan Tamil Refugee Men	-EXCLUDE on population (women)	
54	Afilalo (2015) (ID:87848836)	Characteristics and Needs of Psychiatric Patients With Prolonged Hospital Stay.	-EXCLUDE on population (no housing precarity)	
55	Afrouz (2023) (ID:87963136)	Domestic and Family Violence for Culturally and Linguistically Diverse Communities in Australia during COVID-19 Pandemic	-EXCLUDE on population (no gender focus; women population <50)	
56	Agarwal (2019) (ID:87848385)	Homelessness and Mortality Among Persons With Tuberculosis in Texas, 2010-2017.	-EXCLUDE on population (women)	
57	AGENDA (2020) (ID:87857053)	Often overlooked: young women, poverty and self-harm	-EXCLUDE on intervention (service engagement/helpseeking behaviour)	
58	AGENDA (2021) (ID:87856968)	Voices from lockdown: one year on: a way forward for women and girls	-EXCLUDE on intervention (service engagement/helpseeking behaviour)	
59	**AGENDA (2022) (ID:87856963)**	**Pushed out left out: Girls Speak: final report**	**-INCLUDE on title & abstract**	**-INCLUDE on full study**
60	Agramont-Justiniano (2018) (ID:87858055)	College Students Experiencing Food Insecurity at San José State University	-EXCLUDE on population (women)	
61	Agu (2016) (ID:87851940)	Migrant Sexual Health Help-Seeking and Experiences of Stigmatization and Discrimination in Perth, Western Australia: Exploring Barriers and Enablers	-EXCLUDE on population (no gender focus; women population <50)	
62	Aguiniga (2015) (ID:87852009)	Over the Edge: What Should We Do When Alcohol and Drug Use Become a Problem to Society?	-EXCLUDE on population (women)	
63	AHMAD (2009) (ID:87857276)	Why doesn't she seek help for partner abuse? An exploratory study with South Asian immigrant women	-EXCLUDE on date (2010)	
64	AHMED (2006) (ID:87857386)	Targeted intervention for the ultra poor in rural Bangladesh: Does it make any difference in their health-seeking behaviour?	-EXCLUDE on country (High-Income)	
65	Ahmed (2015) (ID:87850549)	'Internal Orients' : literary representations of colonial modernity and the Kurdish 'other' in Turkey, Iran and Iraq	-EXCLUDE on country (High-Income)	
66	Ahmed (2016) (ID:87848784)	The Impact of Homelessness and Incarceration on Women's Health.	-INCLUDE on title & abstract	-EXCLUDE on intervention (intersectionality)
67	Ahmed (2022) (ID:87857869)	Community health workers and health equity in low- and middle-income countries: systematic review and recommendations for policy and practice	-EXCLUDE on country (High-Income)	
68	Ailawadhi (2014) (ID:87851721)	Outcome Disparities among Ethnic Subgroups of Waldenström's Macroglobulinemia: A Population-Based Study.	-EXCLUDE on population (no housing precarity)	
69	Ailawadhi (2014) (ID:87853228)	Outcome Disparities among Ethntic Subgroups of Waldenstrom's Macroglobulinemia: A Population-Based Study	-EXCLUDE on population (no gender focus; women population <50)	
70	Akande (2022) (ID:87857756)	'Sometimes, we don't know if we're getting abused': discussions of intimate partner violence and HIV risk among transgender women	-INCLUDE on title & abstract	-EXCLUDE on intervention (service engagement/helpseeking behaviour)
71	Akbar (2017) (ID:87850553)	Risk indicators for dental caries in three Scottish prison populations	-EXCLUDE on population (women)	
72	Ake (2018) (ID:87849327)	Needs Assessment for Creating a Patient-Centered, Community-Engaged Health Program for Homeless Pregnant Women	-INCLUDE on title & abstract	-EXCLUDE on intervention (service engagement/helpseeking behaviour)
73	Akers (2017) (ID:87858061)	Individuals in Same-Gender Couples' Experiences of Outness in Adult Romantic Relationships: The Impact of the "Closet" on Connection	-EXCLUDE on intervention (service engagement/helpseeking behaviour)	
74	AKISTER (2004) (ID:87857354)	The parenting task: parents' concerns and where they would seek help	-EXCLUDE on date (2010)	
75	Akkerman (2014) (ID:87848962)	Perspectives of employees with intellectual disabilities on themes relevant to their job satisfaction. an explorative study using photovoice.	-EXCLUDE on intervention (service engagement/helpseeking behaviour)	
76	Aklin (2012) (ID:87849100)	Risk-taking propensity as a predictor of induction onto naltrexone treatment for opioid dependence.	-EXCLUDE on population (women)	

77	AL-KRENAWI (2009) (ID:87857383)	Cross-national comparison of Middle Eastern university students: help seeking behaviours, attitudes towards helping professionals and cultural beliefs about mental health problems	-EXCLUDE on country (High-Income)
78	Al-Natour (2022) (ID:87851591)	Through Her Eyes: The Impact of War on Syrian Refugee Families.	-EXCLUDE on country (High-Income)
79	Al-Shakarchi (2020) (ID:87848283)	Cardiovascular disease in homeless versus housed individuals: a systematic review of observational and interventional studies.	-EXCLUDE on population (women)
80	Al-Temimi (2015) (ID:87851632)	Rethinking the Legal Comparative Study on Children's Rights: Rights Protection and Institutional Capacities in Romania and Kuwait.	-EXCLUDE on population (people aged under 18 years)
81	Alaazi (2015) (ID:87848812)	Therapeutic landscapes of home: Exploring Indigenous peoples' experiences of a Housing First intervention in Winnipeg.	-EXCLUDE on population (women)<br>-EXCLUDE - but review for literature
82	Alama (2011) (ID:87851678)	Perinatal care at the limit of viability between 22 and 26 completed weeks of gestation in Switzerland	-EXCLUDE on population (no housing precarity)
83	Albarran (2015) (ID:87857863)	Expanding Perspectives and Gaining Leverage: How Migrant Farmworker Women Navigate HIV Risk in Their Close, Long-Term Relationships	-EXCLUDE on population (no housing precarity)
84	Albert (2015) (ID:87848817)	Emergency Department Visits Related to Schizophrenia Among Adults Aged 18-64: United States, 2009-2011.	-EXCLUDE on population (women)
85	ALBERT (2015) (ID:87851431)	LGBT youth homelessness: a UK national scoping of cause, prevalence, response and outcome	-EXCLUDE on population (people aged under 18 years)
86	Albright (2017) (ID:87857670)	Applying a Social Justice Lens to Youth Mentoring: A Review of the Literature and Recommendations for Practice	-EXCLUDE on population (women)
87	Albrite (2020) (ID:87857962)	Awakening the Relational Body: Utilization of the Emotionally Focused Therapy Model with Transgender and Gender Expansive Couple Relationships	-EXCLUDE on population (no housing precarity)
88	Alderete (2018) (ID:87848504)	Emerging themes in food security: environmental justice, extended families and the multiple roles of grandmothers.	-EXCLUDE on country (High-Income)
89	Aldiss (2019) (ID:87851995)	Research priorities for young people with cancer: a UK priority setting partnership with the James Lind Alliance	-EXCLUDE on population (people aged under 18 years)
90	Aldridge (2018) (ID:87850835)	Morbidity and mortality in homeless individuals, prisoners, sex workers, and individuals with substance use disorders in high-income countries: A systematic review and meta-analysis.	-EXCLUDE on intervention (service engagement/helpseeking behaviour)
91	Alemi (2020) (ID:87848265)	Relative accuracy of social and medical determinants of suicide in electronic health records.	-EXCLUDE on population (women)
92	Alex (2021) (ID:87851486)	The COVID-19 pandemic: one year later – an occupational perspective	-EXCLUDE on intervention (service engagement/helpseeking behaviour)
93	Alexa (2011) (ID:87849173)	Self-neglect in the case of the elderly. Where are we now?.	-EXCLUDE on population (no gender focus; women population <50)
94	Aléxa (2019) (ID:87851915)	"UPA é nós aqui mesmo": as redes de apoio social no cuidado à saúde da população em situação de rua em um município de pequeno porte	-EXCLUDE on country (High-Income)
95	Algarin (2019) (ID:87857430)	Age, Sex, Race, Ethnicity, Sexual Orientation: Intersectionality of Marginalized-Group Identities and Enacted HIV-Related Stigma Among People Living with HIV in Florida	-EXCLUDE on population (women)
96	Ali (2012) (ID:87849139)	Assessment of prevalence and determinants of posttraumatic stress disorder in survivors of earthquake in Pakistan using Davidson Trauma Scale.	-EXCLUDE on country (High-Income)
97	Ali (2016) (ID:87848766)	Prisoners with intellectual disabilities and detention status. Findings from a UK cross sectional study of prisons.	-EXCLUDE on intervention (service engagement/helpseeking behaviour)
98	Ali (2020) (ID:87850626)	Comorbid health conditions and treatment utilization among individuals with opioid use disorder experiencing homelessness.	-EXCLUDE on population (no gender focus; women population <50)
99	Ali (2021) (ID:87848227)	Facilitating Sustainable Disaster Risk Reduction in Indigenous Communities: Reviving Indigenous Worldviews, Knowledge, and Practices through Two-Way Partnering.	-EXCLUDE on population (no housing precarity)

100	Alkon (2020) (ID:87851900)	'You Can't Evict Community Power': The connection between food justice and eviction defense in Oakland.	-EXCLUDE on intervention (service engagement/helpseeking behaviour)	
101	ALL-PARTY (2018) (ID:87851422)	Creating a truly transformative Domestic Abuse Bill: recommendations from the All-Party Parliamentary Group on Domestic Violence and Abuse, 2017-18	-EXCLUDE on intervention (service engagement/helpseeking behaviour)	
102	Allaria (2021) (ID:87849295)	"Locked down outside": Perception of hazard and health resources in COVID-19 epidemic context among homeless people	-EXCLUDE on population (women)	
103	ALLCOCK (2018) (ID:87857037)	Impact of women specific interventions	-INCLUDE on title & abstract	-EXCLUDE on intervention (service engagement/helpseeking behaviour)
104	Allemani (2015) (ID:87851610)	Global surveillance of cancer survival 1995-2009: analysis of individual data for 25 676 887 patients from 279 population-based registries in 67 countries (CONCORD-2).	-EXCLUDE on country (High-Income)	
105	ALLEN (1996) (ID:87857017)	Women on the edge	-EXCLUDE on date (2010)	
106	Allen (2014) (ID:87851122)	Bringing life course home: A pilot to reduce pregnancy risk through housing access and family support.	-EXCLUDE on intervention (service engagement/helpseeking behaviour)	
107	Allen (2015) (ID:87857940)	The Association Between Alcohol Consumption and Condom Use: Considering Correlates of HIV Risk Among Black Men Who Have Sex with Men	-EXCLUDE on population (women)	
108	Allen (2016) (ID:87857995)	Alcohol use and HIV risk behavior among Black men who have sex with men: Reconsidering the sexual health consequences of problem drinking	-EXCLUDE on population (women)	
109	Allen (2020) (ID:87851508)	Experiences of homeless women in accessing health care in community-based settings: a qualitative systematic review	-INCLUDE on title & abstract	-EXCLUDE on intervention (intersectionality)<br>-EXCLUDE (systematic review)* only use with other code
110	Allsop (2015) (ID:87857692)	Policy Commissions and Representation of Aboriginal Women Issues: A Case-Study of the New Democratic Party of Canada and the Liberal Party of Canada	-EXCLUDE on intervention (service engagement/helpseeking behaviour)	
111	ALLWOOD (2020) (ID:87857312)	Covid-19: understanding inequalities in mental health during the pandemic	-EXCLUDE on population (women)	
112	Alnababtah (2017) (ID:87853141)	Socio-demographic factors which significantly relate to the prediction of burns severity in children	-EXCLUDE on population (people aged under 18 years)	
113	Alperin (2017) (ID:87851553)	Cervical Cancer Attitudes and Knowledge in Somali Refugees in Nebraska	-EXCLUDE on population (no housing precarity)	
114	Alphs (2014) (ID:87848899)	Design and rationale of the Paliperidone Palmitate Research in Demonstrating Effectiveness (PRIDE) study: a novel comparative trial of once-monthly paliperidone palmitate versus daily oral antipsychotic treatment for delaying time to treatment failure in persons with schizophrenia.	-EXCLUDE on population (women)	
115	Alsaïdi (2021) (ID:87851896)	Factors associated with SARS-CoV2 infection and care pathways among the most vulnerable populations living in Marseille: a case control study	-EXCLUDE on population (no housing precarity)	
116	Alschech (2020) (ID:87853093)	Fatherhood Among Youth Experiencing Homelessness	-EXCLUDE on population (women)	
117	Altena (2018) (ID:87848543)	Subgroups of Dutch homeless young adults based on risk- and protective factors for quality of life: Results of a latent class analysis.	-EXCLUDE on population (no gender focus; women population <50)	
118	Alvarado (2020) (ID:87858019)	The Invisible Wall: A Study of Formerly Incarcerated Community College Students and Stigma	-EXCLUDE on population (women)	
119	Alvarez (2014) (ID:87857696)	Building on "the Edge of Each Other's Battles": A Feminist of Color Multidimensional Lens	-EXCLUDE on population (no housing precarity)	
120	Alvarez (2021) (ID:87858014)	Career Development and Workforce Experiences of Trans People in Their Workplaces	-EXCLUDE on population (no housing precarity)	
121	Alvarez-Hernandez (2022) (ID:87852794)	COVID-19 Pandemic and Intimate Partner Violence: an Analysis of Help-Seeking Messages in the Spanish-Speaking Media	-EXCLUDE on population (no gender focus; women population <50)	
122	Alves (2020) (ID:87857956)	A Qualitative Analysis of Survivors' Experiences with Sex Trafficking	-INCLUDE on title & abstract	-EXCLUDE on intervention (service engagement/helpseeking behaviour)
123	Alves (2021) (ID:87857450)	"Mongering Is a Weird Life Sometimes": Discourse Analysis of a Sex Buyer Online Community	-EXCLUDE on intervention (service engagement/helpseeking behaviour)	

124	Alvi (2017) (ID:87857460)	Invisible Voices: An Intersectional Exploration of Quality of Life for Elderly South Asian Immigrant Women in a Canadian Sample	-EXCLUDE on population (no housing precarity)	
125	Alviar (2022) (ID:87963021)	“You should pray about it”: Exploring mental health and help-seeking in Filipino American Catholics.	-EXCLUDE on population (no housing precarity)	
126	Alyan (2015) (ID:87850984)	Experiences of Arab immigrant and Arab-American survivors of sexual violence: An exploratory study.	-EXCLUDE on population (no gender focus; women population <50)	
127	Amarasekera (2019) (ID:87851527)	Prostate cancer in sexual minorities and the influence of HIV status	-EXCLUDE on population (women)	
128	Amati (2015) (ID:87858033)	Experiences of Opportunity in the Northern Resource Frontier	-EXCLUDE on population (women)	
129	Amato (2011) (ID:87851329)	Examining risk factors for homeless men: Gender role conflict, help-seeking behaviors, substance abuse and violence.	-EXCLUDE on population (women)	
130	Amato (2019) (ID:87848476)	Sick and unsheltered: Homelessness as a major risk factor for emergency care utilization.	-EXCLUDE on population (no gender focus; women population <50)	
131	Amis (2018) (ID:87857853)	Inequality, Institutions and Organizations	-EXCLUDE on intervention (service engagement/helpseeking behaviour)	
132	Amos (2023) (ID:87963358)	Family Violence Within LGBTQ Communities in Australia: Intersectional Experiences and Associations with Mental Health Outcomes	-INCLUDE on title & abstract	-EXCLUDE on intervention (service engagement/helpseeking behaviour)
133	Amram (2022) (ID:87850623)	Changes in methadone take-home dosing before and after COVID-19.	-EXCLUDE on intervention (service engagement/helpseeking behaviour)	
134	Amudha (2014) (ID:87852875)	Micro-insurance: A mechanism for women empowerment through Self Help Group linked LIC	-EXCLUDE on country (High-Income)	
135	An Interim Report on... (Kurata) (ID:87851494)	An Interim Report on the Provision of Prenatal Care for Pregnant Mothers Experiencing Homelessness in Hawai'i.	-INCLUDE on title & abstract<br>-EXCLUDE on intervention (service engagement/helpseeking behaviour)	-EXCLUDE on intervention (intersectionality)
136	Andaya (2013) (ID:87851856)	Race and colon cancer survival in an equal-access health care system.	-EXCLUDE on population (no housing precarity)	
137	ANDERSON (2013) (ID:87857236)	“There are too many steps before you get to where you need to be”: help-seeking by patients with first-episode psychosis	-EXCLUDE on population (women)	
138	Andrade (2021) (ID:87848179)	Chickenpox outbreak among Venezuelan immigrants housed in shelters and occupancies in the state of Roraima, Brazil, 2019: a descriptive study.	-EXCLUDE on country (High-Income)	
139	ANDREWS (2011) (ID:87857265)	Between a rock and a hard place? Locus of control, religious problem-solving and psychological help-seeking	-EXCLUDE on population (no housing precarity)	
140	Anitha (2022) (ID:87857457)	Domestic violence during the pandemic: ‘By and for’ frontline practitioners’ mediation of practice and policies to support racially minoritised women 1	-EXCLUDE on population (no housing precarity)	
141	Anne-Cécile (2019) (ID:87851528)	Représenter la dimension spatiale des contextes d'exercice des professionnels intervenant auprès des migrants : le cas des interprètes	-EXCLUDE on intervention (service engagement/helpseeking behaviour)	
142	Announcement of Funding... (2019) (ID:87851989)	Announcement of Funding Awards	-EXCLUDE on intervention (service engagement/helpseeking behaviour)	
143	Anonymous (2011) (ID:87852040)	SUPPORT GROUPS	-EXCLUDE on evidence and form (evidence not in written form or presented as research output)	
144	Ansara (2010) (ID:87852867)	Formal and informal help-seeking associated with women's and men's experiences of intimate partner violence in Canada	-INCLUDE on title & abstract	-EXCLUDE on intervention (intersectionality)
145	Ansberry (2015) (ID:87852011)	Turning Points: From a Widow, Learning Resilience	-EXCLUDE on evidence and form (evidence not in written form or presented as research output)	
146	Antoine (2015) (ID:87857982)	"Pushing the Edge": Challenging Racism and Sexism in American Stand-up Comedy	-EXCLUDE on intervention (service engagement/helpseeking behaviour)	
147	Antunes (2016) (ID:87853298)	No inequalities in survival from colorectal cancer by education and socioeconomic deprivation - a population-based study in the North Region of Portugal, 2000-2002	-EXCLUDE on intervention (service engagement/helpseeking behaviour)	
148	Anwar (2013) (ID:87852734)	Risk Factors of Posttraumatic Stress Disorder After an Earthquake Disaster	-EXCLUDE on intervention (service engagement/helpseeking behaviour)	
149	Anyikwa (2015) (ID:87850995)	The intersections of race and gender in help-seeking strategies among a battered sample of low-income African American women.	-INCLUDE on title & abstract	-EXCLUDE on target group (no housing precarity)

150	APARICIO (2018) (ID:87856998)	Youth and provider perspectives of Wahine Talk: a holistic sexual health and pregnancy prevention program developed with and for homeless youth	-EXCLUDE on intervention (service engagement/helpseeking behaviour)	
151	Aparicio (2019) (ID:87850743)	Holistic, trauma-informed adolescent pregnancy prevention and sexual health promotion for female youth experiencing homelessness: Initial outcomes of Wahine Talk.	-EXCLUDE on population (people aged under 18 years)	
152	Aparicio (2021) (ID:87850587)	Birth control access and selection among youths experiencing homelessness in the United States: A review.	-INCLUDE on title & abstract	-EXCLUDE on intervention (service engagement/helpseeking behaviour)
153	Appeal for Longford... (2019) (ID:87851991)	Appeal for Longford support to help people of Malawi after devastation of Cyclone Idai	-EXCLUDE on country (High-Income)	
154	Appel (2012) (ID:87849103)	Housing First for severely mentally ill homeless methadone patients.	-EXCLUDE on population (women)	
155	Aragona (2020) (ID:87850694)	Negative impacts of COVID-19 lockdown on mental health service access and follow-up adherence for immigrants and individuals in socio-economic difficulties.	-EXCLUDE on population (women)	
156	Araissia (2019) (ID:87852852)	SYRIAN WOMEN REFUGEES IN TUNISIA: DIFFICULTIES ACCESSING ECONOMIC AND SOCIAL RIGHTS	-EXCLUDE on country (High-Income)	
157	Aranda (2020) (ID:87849296)	Good vagrants for the state. The ambivalent development of social housing assistance (France, 1959)	-EXCLUDE on intervention (service engagement/helpseeking behaviour)	
158	Araten-Bergman (2021) (ID:87848237)	Staying connected during COVID-19: Family engagement with adults with developmental disabilities in supported accommodation.	-EXCLUDE on population (no housing precarity)	
159	Araya (2011) (ID:87849235)	Quality of life after postconflict displacement in Ethiopia: comparing placement in a community setting with that in shelters.	-EXCLUDE on country (High-Income)	
160	Arayasirikul (2022) (ID:87963229)	A global cautionary tale: discrimination and violence against trans women worsen despite investments in public resources and improvements in health insurance access and utilization of health care	-EXCLUDE on population (no housing precarity)<br>-EXCLUDE on intervention (service engagement/helpseeking behaviour)	
161	Archibald (2022) (ID:87857832)	"I'm This Girl Who's Drinking and Getting into Trouble": A Feminist Analysis of Alcohol-Related Arrest Narratives of Undergraduate College Women	-EXCLUDE on population (no housing precarity)	
162	Arezoo (2020) (ID:87851514)	Interpretive Model of Glass Ceiling Consequences for Women with an Emphasis on Hegemonic Layers of Power Acceptance	-EXCLUDE on population (no housing precarity)	
163	Arfaoui (2016) (ID:87857651)	Violence against women and Tunisian feminism: Advocacy, policy, and politics in an Arab context	-EXCLUDE on country (High-Income)	
164	Argintaru (2013) (ID:87849018)	A cross-sectional observational study of unmet health needs among homeless and vulnerably housed adults in three Canadian cities.	-EXCLUDE on population (women)	
165	Arias-Uriona (2018) (ID:87851656)	Factors involved in job insecurity and their relationship with the health of salaried workers and contract workers in Bolivia/Factores de precariedad laboral y su relacion con la salud de trabajadores en Bolivia/ Relacao entre condicoes precarias de trabalho e a saude dos trabalhadores assalariados e contratados na Bolivia	-EXCLUDE on country (High-Income)	
166	Arkow (2020) (ID:87857534)	Human–Animal Relationships and Social Work: Opportunities Beyond the Veterinary Environment	-EXCLUDE on population (women)	
167	Armenta (2015) (ID:87848810)	Correlates of perceived risk of HIV infection among persons who inject drugs in Tijuana, Baja California, Mexico.	-EXCLUDE on country (High-Income)	
168	Arnold (2017) (ID:87848667)	Identifying social and economic barriers to regular care and treatment for Black men who have sex with men and women (BMSMW) and who are living with HIV: a qualitative study from the Bruthas cohort.	-EXCLUDE on population (women)	
169	Arnold (2019) (ID:87853198)	The Stepped Care Intervention to Suppress Viral Load in Youth Living With HIV: Protocol for a Randomized Controlled Trial	-EXCLUDE on population (women)	
170	Arnold (2022) (ID:87852856)	HPV Vaccination among Sexual and Gender Minority Youth Living with or at High-Risk for HIV	-EXCLUDE on population (no gender focus; women population <50)	
171	Arnoso (2023) (ID:87963204)	Meanings of Loneliness for Women Using Social Services in Spain: An Intersectional Analysis	-EXCLUDE on population (no housing precarity)	

172	ARNS (2004) (ID:87857399)	Psychosocial needs of HIV-positive individuals seeking workforce re-entry	-EXCLUDE on population (women)	
173	Aronson (2020) (ID:87857966)	The theory and practice of culturally relevant education: expanding the conversation to include gender and sexuality equity	-EXCLUDE on intervention (service engagement/helpseeking behaviour)	
174	Arriola (2015) (ID:87848894)	Understanding the relationship between social support and physical and mental well-being among jail detainees living with HIV.	-EXCLUDE on population (women)	
175	Artenie (2015) (ID:87848865)	Visits to primary care physicians among persons who inject drugs at high risk of hepatitis C virus infection: room for improvement.	-EXCLUDE on population (women)	
176	Asakura (2018) (ID:87857695)	Attending to Social Justice in Clinical Social Work: Supervision as a Pedagogical Space	-EXCLUDE on intervention (service engagement/helpseeking behaviour)	
177	Asana (2018) (ID:87848547)	Associations of alcohol use disorder, alcohol use, housing, and service use in a homeless sample of 255 individuals followed over 2 years.	-EXCLUDE on population (women)	
178	Asberg (2015) (ID:87850968)	Safer in jail? A comparison of victimization history and psychological adjustment between previously homeless and non-homeless incarcerated women.	-EXCLUDE on intervention (service engagement/helpseeking behaviour)<br>-EXCLUDE - but review for literature	
179	Asefnia (2020) (ID:87963246)	Foster Care and Youth Homelessness: The Impact of Race and Victimization History	-INCLUDE on title & abstract	-EXCLUDE on target group (not focused on women's behaviour/outcomes for women)
180	Asgary (2011) (ID:87849194)	Barriers to health care access among refugee asylum seekers.	-EXCLUDE on population (women)	
181	Asgary (2014) (ID:87852985)	Breast Cancer Screening Among Homeless Women of New York City Shelter-Based Clinics	-EXCLUDE on intervention (service engagement/helpseeking behaviour)	
182	Asgary (2015) (ID:87848821)	Human Papillomavirus Knowledge and Attitude among Homeless Women of New York City Shelters.	-EXCLUDE on intervention (service engagement/helpseeking behaviour)	
183	Asgary (2016) (ID:87848781)	Rates and Predictors of Uncontrolled Hypertension Among Hypertensive Homeless Adults Using New York City Shelter-Based Clinics.	-EXCLUDE on population (women)	
184	Asgary (2016) (ID:87853305)	Cervical Cancer Screening Among Homeless Women of New York City Shelters	-EXCLUDE on intervention (service engagement/helpseeking behaviour)	
185	Asgary (2018) (ID:87852776)	Cancer screening in the homeless population	-EXCLUDE on population (women)	
186	Ashford (2018) (ID:87848495)	Peer-delivered harm reduction and recovery support services: initial evaluation from a hybrid recovery community drop-in center and syringe exchange program.	-EXCLUDE on population (no gender focus; women population <50)	
187	Ashford (2019) (ID:87848399)	Reducing harm and promoting recovery through community-based mutual aid: Characterizing those who engage in a hybrid peer recovery community organization.	-EXCLUDE on population (no gender focus; women population <50)	
188	ASHLEY (2015) (ID:87857301)	The impact of combat status on veterans' attitudes toward help seeking: the hierarchy of combat elitism	-EXCLUDE on population (women)	
189	Asi (2022) (ID:87857829)	Assessing gender responsiveness of COVID-19 response plans for populations in conflict-affected humanitarian emergencies	-EXCLUDE on country (High-Income)	
190	Aslam (2021) (ID:87850644)	Focussing on homeless LGBT+ college students during COVID-19.	-EXCLUDE on evidence and form (evidence not in written form or presented as research output)	
191	ASMOREDJO (2017) (ID:87856964)	Client experiences with shelter and community care services in the Netherlands: quality of services for homeless people, homeless youth, and abused women	-EXCLUDE on population (no gender focus; women population <50)<br>-EXCLUDE - but review for literature	
192	ASSOCIATION (2022) (ID:87857104)	Safeguarding pressures phase 8	-EXCLUDE on population (people aged under 18 years)	
193	Association of physician... (Saab) (ID:87851745)	Association of physician care with mortality in Kidney Early Evaluation Program (KEEP) participants.	-EXCLUDE on population (no housing precarity)	
194	Astroth (2018) (ID:87848520)	A qualitative exploration of nursing students' perceptions of the homeless and their care experiences.	-EXCLUDE on population (no gender focus; women population <50)	
195	Ataiants (2019) (ID:87850750)	Drug overdose among female participants of a harm reduction program in Philadelphia.	-INCLUDE on title & abstract	-EXCLUDE on intervention (intersectionality)
196	Atherley (2023) (ID:87857718)	Longitudinal exploration of students' identity formation during the transition from pre-clinical to clinical training using research poetry	-EXCLUDE on population (women)	

197	Attar-Schwartz (2019) (ID:87852667)	Does a Co-Resident Grandparent Matter? Characteristics of Maltreatment-Related Investigations Involving Lone-Parent Families	-EXCLUDE on population (people aged under 18 years)	
198	AU (2013) (ID:87857340)	Coping strategies and social support-seeking behaviour among Chinese caring for older people with dementia	-EXCLUDE on population (no housing precarity)	
199	Aubry (2016) (ID:87848729)	A Longitudinal Study of Predictors of Housing Stability, Housing Quality, and Mental Health Functioning Among Single Homeless Individuals Staying in Emergency Shelters.	-EXCLUDE on population (no gender focus; women population <50)<br>-EXCLUDE - but review for literature	
200	Aubry (2016) (ID:87848786)	A Multiple-City RCT of Housing First With Assertive Community Treatment for Homeless Canadians With Serious Mental Illness.	-EXCLUDE on population (no gender focus; women population <50)	
201	AUGUSTYN (2022) (ID:87857192)	The contextual influences of police and social service providers on formal help-seeking after incidents of intimate partner violence	-INCLUDE on title & abstract	-EXCLUDE on intervention (intersectionality)
202	Aujla (2021) (ID:87963348)	"It was like sugar-coated words": Revictimization when south Asian immigrant women disclose domestic violence	-INCLUDE on title & abstract	-EXCLUDE (IPV/DVA but little discussion on housing)
203	Ausikaitis (2014) (ID:87857596)	Empowering homeless youth in transitional living programs: A transformative mixed methods approach to understanding their transition to adulthood	-EXCLUDE on population (women)	
204	AUSLANDER (2003) (ID:87857274)	The supportive community; help seeking and service use among elderly people in Jerusalem	-EXCLUDE on population (no housing precarity)	
205	Austin (2011) (ID:87851338)	The influence of co-occurring Axis I disorders on treatment utilization and outcome in homeless patients with substance use disorders.	-EXCLUDE on population (women)	
206	**AUSTIN (2019) (ID:87857058)**	**Nowhere to turn 2019: findings from the third year of the No Woman Turned Away project**	**-INCLUDE on title & abstract**	**-INCLUDE on full study**
207	AVA (2017) (ID:87857004)	Mapping the Maze. The core components of a gender sensitive service for women experiencing multiple disadvantage: a review of the literature	-INCLUDE on title & abstract	-EXCLUDE on intervention (intersectionality)
208	Avanza (2020) (ID:87857452)	Using a Feminist Paradigm (Intersectionality) to Study Conservative Women: The Case of Pro-life Activists in Italy	-EXCLUDE on population (no housing precarity)	
209	Avegno (2023) (ID:87851873)	Evaluating a Telephone and Home Blood Pressure Monitoring Intervention to Improve Blood Pressure Control and Self-Care Behaviors in Adults with Low-Socioeconomic Status	-EXCLUDE on population (women)	
210	Avery (2013) (ID:87849071)	Jails as an opportunity to increase engagement in HIV care: findings from an observational cross-sectional study.	-EXCLUDE on population (women)	
211	Avieli (2016) (ID:87848762)	Aging With Schizophrenia: A Lifelong Experience of Multidimensional Losses and Suffering.	-EXCLUDE on population (no housing precarity)	
212	Avila (2008) (ID:87853692)	A grant proposal: To assist women of intimate partner violence in the city of San Diego	-EXCLUDE on evidence and form (evidence not in written form or presented as research output)	
213	Aviles (2015) (ID:87857544)	Homeless Educational Policy: Exploring a Racialized Discourse Through a Critical Race Theory Lens	-EXCLUDE on population (people aged under 18 years)	
214	Awana (2021) (ID:87857912)	Language Learning, Technology, and International Undergraduate Students at Junior and Senior Levels: A Proverbial Double Jeopardy for Academic Success in the Southwestern Borderlands of the United States	-EXCLUDE on population (no housing precarity)	
215	Awor (2020) (ID:87848308)	Innovations in maternal and child health: case studies from Uganda.	-EXCLUDE on country (High-Income)	
216	Ayangbayi (2017) (ID:87848678)	Characteristics of Hospital Emergency Room Visits for Mental and Substance Use Disorders.	-EXCLUDE on population (women)	
217	Ayoub (2023) (ID:87853193)	Two sides of the same coin: A mixed methods study of Black mothers' experiences with violence, stressors, parenting, and coping during the COVID-19 pandemic	-INCLUDE on title & abstract	-EXCLUDE on target group (no housing precarity)
218	Ayvaci (2018) (ID:87848594)	A naturalistic longitudinal study of the order of service provision with respect to cocaine use and outcomes in an urban homeless sample.	-EXCLUDE on population (women)	
219	Azar (2021) (ID:87848187)	Persistence of Disparities Among Racially/Ethnically Marginalized Groups in the Coronavirus Disease 2019 Pandemic Regardless of Statewide Shelter-in-Place Policies: An Analysis From Northern California.	-EXCLUDE on population (no gender focus; women population <50)	

220	Azhar (2021) (ID:87963108)	Navigating intersectional stigma: Strategies for coping among cisgender women of color	-INCLUDE on title & abstract	-EXCLUDE on target group (no housing precarity)
221	AZZUDIN (2016) (ID:87857333)	Amaan: information about mental health and ways to look after your wellbeing: for asylum seeking and refugee women	-EXCLUDE on evidence and form (evidence not in written form or presented as research output)<br>-EXCLUDE - but review for literature	
222	Babak (2021) (ID:87851484)	Sex and age difference in risk factor distribution, trend, and long-term outcome of patients undergoing isolated coronary artery bypass graft surgery	-EXCLUDE on population (no housing precarity)	
223	Babayan (2021) (ID:87848206)	Advocates Make a Difference in Duration of Homelessness and Quality of Life.	-EXCLUDE on population (no gender focus; women population <50)	
224	Babb (2022) (ID:87858079)	'The real indigenous are higher up': locating race and gender in Andean Peru	-EXCLUDE on country (High-Income)	
225	BACCHUS (2003) (ID:87857304)	Experiences of seeking help from health professionals in a sample of women who experienced domestic violence	-EXCLUDE on date (2010)	
226	Bach (2021) (ID:87963072)	Underserved survivors of sexual assault: A systematic scoping review	-INCLUDE on title & abstract	-EXCLUDE on intervention (service engagement/helpseeking behaviour)<br>-EXCLUDE (systematic review)* only use with other code
227	BACHMANN (2018) (ID:87851419)	LGBT in Britain: trans report	-INCLUDE on title & abstract	-EXCLUDE on target group (no housing precarity)
228	BACKHAUS (1989) (ID:87857166)	Training mental health practitioners to work with adoptive families who seek help	-EXCLUDE on population (no housing precarity)	
229	Badger (2019) (ID:87858052)	Challenging Communication Management: Performative Abolition Communication Education	-EXCLUDE on intervention (service engagement/helpseeking behaviour)	
230	Badri (2013) (ID:87849080)	Experiences and psychosocial adjustment of Darfuri female students affected by war: an exploratory study.	-EXCLUDE on country (High-Income)	
231	Baer (2012) (ID:87857528)	German Feminism in the Age of Neoliberalism: Jana Hensel and Elisabeth Raether's "Neue deutsche Mädchen"	-EXCLUDE on intervention (service engagement/helpseeking behaviour)	
232	Baer (2016) (ID:87857856)	Digital Feminisms and the Impasse: Time, Disappearance, and Delay in Neoliberalism	-EXCLUDE on intervention (service engagement/helpseeking behaviour)	
233	Baggett (2010) (ID:87852827)	Cigarette Smoking and Advice to Quit in a National Sample of Homeless Adults	-EXCLUDE on population (women)	
234	Baggett (2011) (ID:87849213)	Food insufficiency and health services utilization in a national sample of homeless adults.	-EXCLUDE on population (no gender focus; women population <50)<br>-EXCLUDE - but review for literature	
235	Baggett (2013) (ID:87851164)	Homelessness, cigarette smoking and desire to quit: Results from a US national study.	-EXCLUDE on population (women)	
236	Baggett (2015) (ID:87848873)	Tobacco-, alcohol-, and drug-attributable deaths and their contribution to mortality disparities in a cohort of homeless adults in Boston.	-EXCLUDE on population (women)	
237	Baggett (2016) (ID:87848753)	Other tobacco product and electronic cigarette use among homeless cigarette smokers.	-EXCLUDE on population (no gender focus; women population <50)	
238	Baggett (2016) (ID:87848789)	Posttraumatic Stress Symptoms and Their Association With Smoking Outcome Expectancies Among Homeless Smokers in Boston.	-EXCLUDE on population (women)	
239	Baggett (2018) (ID:87848546)	Subsistence difficulties are associated with more barriers to quitting and worse abstinence outcomes among homeless smokers: evidence from two studies in Boston, Massachusetts.	-EXCLUDE on population (women)	
240	Baggett (2022) (ID:87852857)	Patient navigation to promote lung cancer screening in a community health center for people experiencing homelessness: Protocol for a pragmatic randomized controlled trial	-EXCLUDE on population (no gender focus; women population <50)	
241	Bagley (2021) (ID:87848193)	Integrating substance use care into primary care for adolescents and young adults: Lessons learned.	-EXCLUDE on population (women)	
242	Baharoon (2016) (ID:87853288)	Continuous Mandatory Onsite Consultant Intensivists in the ICU: Impacts on Patient Outcomes	-EXCLUDE on population (women)	
243	Baiardi (2015) (ID:87848842)	Using Sociograms to Enhance Power and Voice in Focus Groups.	-EXCLUDE on intervention (service engagement/helpseeking behaviour)	

244	BAILEY (2010) (ID:87857614)	LOST IN TRANSLATION: DOMESTIC VIOLENCE, "THE PERSONAL IS POLITICAL," AND THE CRIMINAL JUSTICE SYSTEM	-EXCLUDE - but review for literature<br>-EXCLUDE on intervention (service engagement/helpseeking behaviour)	
245	Baillargeon (2014) (ID:87963075)	The help seeking behaviors of students of color: Factors influencing the utilization of mental health resources on a college campus	-EXCLUDE on population (no housing precarity)	
246	Bajger (2022) (ID:87850551)	Homeless at home : the figure of the proto-queer woman in cinema of communist Poland (1970s-1980s)	-EXCLUDE on intervention (service engagement/helpseeking behaviour)	
247	Baker (2003) (ID:87853703)	Domestic Violence and Housing Problems: A Contextual Analysis of Women's Help-Seeking, Received Informal Support, and Formal System Response	-EXCLUDE on date (2010)	
248	BAKER (2010) (ID:87856970)	Domestic violence, housing instability, and homelessness: a review of housing policies and program practices for meeting the needs of survivors	-INCLUDE on title & abstract	-EXCLUDE on intervention (intersectionality)
249	Balaj (2021) (ID:87853360)	Parental education and inequalities in child mortality: a global systematic review and meta-analysis	-EXCLUDE on population (no housing precarity)	
250	Balarabe (2014) (ID:87848935)	The Sokoto blind beggars: causes of blindness and barriers to rehabilitation services.	-EXCLUDE on country (High-Income)	
251	Balgiu (2022) (ID:87851969)	Romanian Version of the Oral Health Values Scale: Adaptation and Validation	-EXCLUDE on population (no housing precarity)	
252	Ballentine (2020) (ID:87853107)	From Scarcity to Investment: The Range of Strategies Used by Low-Income Parents With "Good" Low-Wage Jobs	-EXCLUDE on population (no housing precarity)	
253	Ballet (2013) (ID:87851156)	Street boys and girls: A case study in Mauritania.	-EXCLUDE on country (High-Income)	
254	Banasova (2022) (ID:88019133)	Emotional difficulties, coping strategies, and help-seeking patterns among Czech perinatal women	-EXCLUDE on population (no housing precarity)	
255	Banati (2021) (ID:87853327)	Addressing the Mental and Emotional Health Impacts of COVID-19 on Children and Adolescents: Lessons From HIV/AIDS	-EXCLUDE on population (people aged under 18 years)	
256	Bansal (2022) (ID:87963276)	Understanding ethnic inequalities in mental healthcare in the UK: A meta-ethnography	-EXCLUDE on population (no housing precarity)	
257	Baranowski (2021) (ID:87857897)	The Experiences of LGBTQ+ Students with Mental Health Disabilities in Community College	-EXCLUDE on population (no housing precarity)	
258	Barata (2015) (ID:87853263)	Health social inequality of the homeless in the city of Sao Paulo	-EXCLUDE on country (High-Income)	
259	Barbosa-Leiker (2021) (ID:87851638)	Stressors, coping, and resources needed during the COVID-19 pandemic in a sample of perinatal women	-EXCLUDE on population (no housing precarity)	
260	Bardwell (2018) (ID:87848514)	Negotiating space & drug use in emergency shelters with peer witness injection programs within the context of an overdose crisis: A qualitative study.	-EXCLUDE on population (women)	
261	Barennes (2010) (ID:87853091)	Survival and health status of DOTS tuberculosis patients in rural Lao PDR	-EXCLUDE on country (High-Income)	
262	Barfield (2019) (ID:87858112)	The evolution of person-centered encounter: creating egalitarian environments for mattering, meaning & healing	-EXCLUDE on population (women)	
263	Baribeau (2017) (ID:87851648)	An advocacy experience for medical students.	-EXCLUDE on intervention (service engagement/helpseeking behaviour)	
264	Bark (2022) (ID:87851876)	AN INTEGRATED APPROACH TO CARDIOVASCULAR DISEASE IN HOMELESS INDIVIDUALS: A QUALITATIVE STUDY	-EXCLUDE on population (no gender focus; women population <50)	
265	Bark (2023) (ID:87851872)	Integrated approach to cardiovascular disease in people experiencing homelessness: a qualitative study	-EXCLUDE on population (women)	
266	Barker (2015) (ID:87848853)	Barriers to health and social services for street-involved youth in a Canadian setting.	-EXCLUDE on population (women)	
267	BARKET (2012) (ID:87856986)	Social capital, homeless young people and the family	-EXCLUDE on population (women)	
268	Barman-Adhikari (2011) (ID:87851306)	Sexual health information seeking online among runaway and homeless youth.	-EXCLUDE on population (no gender focus; women population <50)	
269	Barman-Adhikari (2014) (ID:87848937)	Social networks as the context for understanding employment services utilization among homeless youth.	-EXCLUDE on population (no gender focus; women population <50)	
270	Barman-Adhikari (2015) (ID:87850977)	Social network correlates of methamphetamine, heroin, and cocaine use in a sociometric network of homeless youth.	-EXCLUDE on population (women)	
271	Barman-Adhikari (2016) (ID:87848744)	Sociometric network structure and its association with methamphetamine use norms among homeless youth.	-EXCLUDE on population (women)	

272	Barman-Adhikari (2017) (ID:87848693)	Descriptive and injunctive network norms associated with nonmedical use of prescription drugs among homeless youth.	-EXCLUDE on population (women)	
273	Barman-Adhikari (2017) (ID:87850896)	Condomless sex among homeless youth: The role of multidimensional social norms and gender.	-EXCLUDE on population (no gender focus; women population <50)	
274	Barman-Adhikari (2019) (ID:87848450)	On the fringes: How youth experiencing homelessness conceptualize social and economic inequality-A Photovoice study.	-EXCLUDE on population (no gender focus; women population <50)	
275	Barmettler (2018) (ID:87851799)	Association of Immunoglobulin Levels, Infectious Risk, and Mortality With Rituximab and Hypogammaglobulinemia.	-EXCLUDE on intervention (service engagement/helpseeking behaviour)	
276	Barnabe (2021) (ID:87857857)	Informing the GRADE evidence to decision process with health equity considerations: demonstration from the Canadian rheumatoid arthritis care context	-EXCLUDE on intervention (service engagement/helpseeking behaviour)	
277	Barnert (2019) (ID:87848424)	Understanding Commercially Sexually Exploited Young Women's Access to, Utilization of, and Engagement in Health Care: "Work Around What I Need".	-INCLUDE on title & abstract	-EXCLUDE on target group (no housing precarity)
278	Barnert (2020) (ID:87963274)	Behavioral health treatment “Buy-in” among adolescent females with histories of commercial sexual exploitation	-INCLUDE on title & abstract	-EXCLUDE on target group (no housing precarity)
279	Barnes (2021) (ID:87857618)	Black Sociologists and Civic Engagement	-EXCLUDE on population (no housing precarity)	
280	Baron (2011) (ID:87851337)	Street youths' fear of violent crime.	-EXCLUDE on population (no gender focus; women population <50)	
281	Barrera (2022) (ID:87848140)	County-Level Associations Between Pregnancy-Related Mortality Ratios and Contextual Sociospatial Indicators.	-EXCLUDE on population (no housing precarity)	
282	Barreras (2019) (ID:87853079)	"We have a stronger survival mode": exploring knowledge gaps and culturally sensitive messaging of PrEP among Latino men who have sex with men and Latina transgender women in Los Angeles, CA	-EXCLUDE on population (no gender focus; women population <50)	
283	Barreto (2017) (ID:87853200)	Food Insecurity Increases HIV Risk Among Young Sex Workers in Metro Vancouver, Canada	-EXCLUDE on intervention (service engagement/helpseeking behaviour)	
284	Barrios (2021) (ID:87963352)	Future directions in intimate partner violence research: An intersectionality framework for analyzing women’s processes of leaving abusive relationships	-INCLUDE on title & abstract	-EXCLUDE (IPV/DVA but little discussion on housing)
285	Barros (2020) (ID:87853292)	Healthcare experiences of homeless pregnant women	-INCLUDE on title & abstract	-EXCLUDE on country (High-income)
286	Barrow (2014) (ID:87848924)	Context and opportunity: multiple perspectives on parenting by women with a severe mental illness.	-INCLUDE on title & abstract	-EXCLUDE on target group (not focused on women's behaviour/outcomes for women)
287	Barrow (2019) (ID:87848400)	The introduction of a homeless healthcare team in hospital improves staff knowledge and attitudes towards homeless patients.	-EXCLUDE on population (women)	
288	Bartels (2010) (ID:87963258)	Emerging issues in domestic/family violence research	-EXCLUDE on intervention (service engagement/helpseeking behaviour)	
289	Bartley (1995) (ID:87850538)	'Seeking and saving' : the reform of prostitutes and the prevention of prostitution in Birmingham, 1860-1914	-EXCLUDE on date (2010)	
290	Basile (2012) (ID:87858070)	Reproductive justice and childbirth reform: Doulas as agents of social change	-EXCLUDE on population (no housing precarity)	
291	Basnet (2015) (ID:87848863)	Characteristics of drug-abusing females with and without children seeking treatment in Helsinki, Finland.	-EXCLUDE on intervention (service engagement/helpseeking behaviour)	
292	Bassel (2017) (ID:87963325)	Minority women and austerity: Survival and resistance in France and Britain	-EXCLUDE on population (no housing precarity)	
293	Bassuk (2014) (ID:87851105)	Depression in homeless mothers: Addressing an unrecognized public health issue.	-INCLUDE on title & abstract	-EXCLUDE on intervention (intersectionality)
294	Batastini (2022) (ID:87963240)	Why correctional service providers and researchers should focus on intersectionality and recommendations to get started	-EXCLUDE on population (women)<br>-EXCLUDE - but review for literature	
295	Bateman (2015) (ID:87851428)	LGBT youth homelessness: UK national scoping exercise	-INCLUDE on title & abstract	-EXCLUDE on intervention (service engagement/helpseeking behaviour)
296	Bath (2021) (ID:87963109)	Perspectives of Girls and Young Women Affected by Commercial Sexual Exploitation: mHealth as a Tool to Increase Engagement in Care	-EXCLUDE on intervention (service engagement/helpseeking behaviour)	

297	Batra (2020) (ID:87853420)	Understanding the multidimensional trajectory of psychosocial maternal risk factors causing preterm birth: A systematic review	-EXCLUDE on intervention (service engagement/helpseeking behaviour)<br>-EXCLUDE - but review for literature	
298	Batty (2021) (ID:87851888)	Adverse childhood experiences and adult health: the need for stronger study designs to evaluate impact	-EXCLUDE on population (women)	
299	Bauder (2022) (ID:87849313)	From Sovereignty to Solidarity: Rethinking Human Migration	-EXCLUDE on intervention (service engagement/helpseeking behaviour)	
300	Bauer (2021) (ID:87848192)	Census Tract Patterns and Contextual Social Determinants of Health Associated With COVID-19 in a Hispanic Population From South Texas: A Spatiotemporal Perspective.	-EXCLUDE on intervention (service engagement/helpseeking behaviour)	
301	**Baumann (2019) (ID:87850778)**	**Experiences of violence and head injury among women and transgender women sex workers.**	**-INCLUDE on title & abstract**	**-INCLUDE on full study**
302	Baumgartner (2012) (ID:87849118)	Community integration of formerly homeless men and women with severe mental illness after hospital discharge.	-EXCLUDE on population (no gender focus; women population <50)	
303	Bawah (2019) (ID:87848387)	Prevalence of diabetes among homeless and slum dwellers in Accra, Ghana: a survey study.	-EXCLUDE on country (High-Income)	
304	Bean (2013) (ID:87849045)	The impact of housing first and peer support on people who are medically vulnerable and homeless.	-EXCLUDE on population (women)	
305	BEAR (2019) (ID:87857160)	A sense of safety: trauma-informed approaches for women	-INCLUDE on title & abstract	-EXCLUDE on intervention (intersectionality)
306	BEAULAURIER (2005) (ID:87857316)	Internal barriers to help seeking for middle-aged and older women who experience intimate partner violence	-EXCLUDE on date (2010)	
307	Beaulaurier (2008) (ID:87853690)	Barriers to Help-Seeking for Older Women Who Experience Intimate Partner Violence: A Descriptive Model	-EXCLUDE on date (2010)	
308	Bedell (2019) (ID:87849349)	Mixed-methods evaluation of a New York City resource guide for low-income residents	-EXCLUDE on intervention (service engagement/helpseeking behaviour)	
309	Beder (2017) (ID:87850851)	Caring for the military: A guide for helping professionals.	-EXCLUDE on population (no housing precarity)	
310	Bege (2019) (ID:87853334)	Epidemiology of trauma in France: mortality and risk factors based on a national medico-administrative database	-EXCLUDE on population (no housing precarity)	
311	Begun (2018) (ID:87850841)	A mixed methods examination of pregnancy attitudes and HIV risk behaviors among homeless youth: The role of social network norms and social support.	-EXCLUDE on population (no gender focus; women population <50)<br>-EXCLUDE - but review for literature	
312	Begun (2019) (ID:87848468)	"It seems kinda like a different language to us": Homeless youths' attitudes and experiences pertaining to condoms and contraceptives.	-EXCLUDE on population (no gender focus; women population <50)	
313	Begun (2019) (ID:87850735)	"Getting pregnant might make me seem more normal to them": Attitudes, experiences, and gendered nuances regarding pregnancy and parenting among youths experiencing homelessness.	-INCLUDE on title & abstract	-EXCLUDE on intervention (service engagement/helpseeking behaviour)
314	Begun (2020) (ID:87850707)	Social support and pregnancy attitudes among youth experiencing homelessness.	-INCLUDE on title & abstract	-EXCLUDE on target group (not focused on women's behaviour/outcomes for women)
315	Beharie (2015) (ID:87850972)	Assessing the relationship between the perceived shelter environment and mental health among homeless caregivers.	-EXCLUDE on population (women)	
316	Behl-Chadha (2017) (ID:87848618)	Comparison of Patient Experience between a Practice for Homeless Patients and Other Practices Engaged in a Patient-Centered Medical Home Initiative.	-EXCLUDE on population (women)	
317	Behr (2016) (ID:87852697)	Emergency Department Frequent Utilization for Non-Emergent Presentments: Results from a Regional Urban Trauma Center Study	-EXCLUDE on population (women)	
318	BEIER (2009) (ID:87857282)	Encouraging self-identified pedophiles and hebephiles to seek professional help: first results of the Prevention Project Dunkelfeld (PPD)	-EXCLUDE on population (women)	
319	Beigulenko (2002) (ID:87850521)	Women's experience of home, homelessness and home ownership in Moscow after the Soviet Union	-EXCLUDE on date (2010)	
320	Beijer (2012) (ID:87849311)	Prevalence of tuberculosis, hepatitis C virus, and HIV in homeless people: A systematic review and meta-analysis	-EXCLUDE on intervention (service engagement/helpseeking behaviour)	
321	Beijer (2016) (ID:87853220)	Changes over time in the risk of hospitalization for physical diseases among homeless men and women in Stockholm: A comparison of two cohorts	-EXCLUDE on population (women)	
322	Beijersbergen (2015) (ID:87852844)	Psychometric properties of the consumer quality index to assess shelter and community care services	-EXCLUDE on population (women)	

323	Beima-Sofie (2020) (ID:87848294)	"It's Me as a Person, Not Me the Disease": Patient Perceptions of an HIV Care Model Designed to Engage Persons with Complex Needs.	-EXCLUDE on population (women)	
324	Beiser (2017) (ID:87848661)	Needs Assessment of HCV-Infected Individuals Experiencing Homelessness and Implications.	-EXCLUDE on population (women)	
325	Beiser (2019) (ID:87848425)	Hepatitis C treatment outcomes among homeless-experienced individuals at a community health centre in Boston.	-EXCLUDE on population (women)	
326	Beiser (2023) (ID:87848128)	Hepatitis C Virus Reinfection in a Real-World Cohort of Homeless-Experienced Individuals in Boston.	-EXCLUDE on population (women)	
327	Bekhet (2011) (ID:87849218)	The effects of positive cognitions on the relationship between alienation and resourcefulness in nursing students in Egypt.	-EXCLUDE on country (High-Income)	
328	Bell (2017) (ID:87848651)	Factors associated with high-utilization in a safety net setting.	-EXCLUDE on population (women)<br>-EXCLUDE - but review for literature	
329	Bell (2017) (ID:87857622)	Medicaid Reform, Responsiblization Policies, and the Synergism of Barriers to Low-income Health Seeking	-EXCLUDE on population (women)	
330	Bell (2020) (ID:87850708)	Military sexual trauma, betrayal trauma, and spiritual coping: Investigating what impacts reintegration among homeless female veterans.	-INCLUDE on title & abstract	-EXCLUDE on intervention (service engagement/helpseeking behaviour)
331	Bello (2022) (ID:87853038)	Normalizing the exception: prejudice and discriminations in detention and extraordinary reception centres in Italy	-EXCLUDE on intervention (service engagement/helpseeking behaviour)	
332	Beltran (2019) (ID:87857415)	Intersectional discrimination is associated with housing instability among trans women living in the San Francisco Bay Area	-INCLUDE on title & abstract	-EXCLUDE on intervention (service engagement/helpseeking behaviour)
333	**Ben-Porat (2020) (ID:87850693)**	**Patterns of service utilization among women who are victims of domestic violence: The contribution of cultural background, characteristics of violence, and psychological distress.**	**-INCLUDE on title & abstract**	**-INCLUDE on full study**
334	Ben-Porat (2021) (ID:87852969)	Length of Women's Stays in Domestic Violence Shelters: Examining the Contribution of Background Variables, Level of Violence, Reasons for Entering Shelters, and Expectations	-INCLUDE on title & abstract	-EXCLUDE on intervention (service engagement/helpseeking behaviour)
335	Ben-Porat (2023) (ID:87850582)	Stay-leave decision-making among women victims of domestic violence in Israel: Background, interactional, and environmental factors.	-INCLUDE on title & abstract	-EXCLUDE on intervention (service engagement/helpseeking behaviour)
336	Benbow (2011) (ID:87963060)	Mothers with mental illness experiencing homelessness: A critical analysis	-INCLUDE on title & abstract	-EXCLUDE on intervention (service engagement/helpseeking behaviour)
337	Benbow (2015) (ID:87857500)	Mothers Experiencing Homelessness: Social Exclusion, Resistance, and Health	-INCLUDE on title & abstract	-EXCLUDE on intervention (service engagement/helpseeking behaviour)
338	**Benbow (2019) (ID:87857419)**	**Spaces of Exclusion: Safety, Stigma, and Surveillance of Mothers Experiencing Homelessness**	**-INCLUDE on title & abstract**	**-INCLUDE on full study**
339	**Benbow (2019) (ID:87857504)**	**Mothering Without a Home: Internalized Impacts of Social Exclusion**	**-INCLUDE on title & abstract**	**-INCLUDE on full study**
340	**Benbow (2019) (ID:87963310)**	**“Until You Hit Rock Bottom There’s No Support”: Contradictory Sources and Systems of Support for Mothers Experiencing Homelessness in Southwestern Ontario**	**-INCLUDE on title & abstract**	**-INCLUDE on full study**
341	BENDA (2005) (ID:87856985)	A study of substance abuse, traumata, and social support systems among homeless veterans	-EXCLUDE on population (no gender focus; women population <50)	
342	Bender (2016) (ID:87852818)	Health Care Experiences of Rural Women Experiencing Intimate Partner Violence and Substance Abuse	-INCLUDE on title & abstract	-EXCLUDE on intervention (service engagement/helpseeking behaviour)
343	Benitez (2020) (ID:87848328)	Geographically Focused Collocated Hepatitis C Screening and Treatment in Los Angeles's Skid Row.	-EXCLUDE on population (women)	
344	Benjenk (2021) (ID:87848177)	Impact of COVID-19 Mitigation Efforts on Adults With Serious Mental Illness: A Patient-Centered Perspective.	-EXCLUDE on population (women)	
345	Benoit (2023) (ID:87858010)	Decriminalization and What Else? Alternative Structural Interventions to Promote the Health, Safety, and Rights of Sex Workers	-INCLUDE on title & abstract	-EXCLUDE on intervention (service engagement/helpseeking behaviour)
346	Benson (2018) (ID:87849325)	Keyworkers’ experiences and perceptions of using psychological approaches with people experiencing homelessness	-EXCLUDE on population (women)	

347	Bent-Goodley (2014) (ID:87963112)	An exploration of African American women's perceptions of the intersection of domestic violence and HIV/AIDS	-INCLUDE on title & abstract	-EXCLUDE on target group (no housing precarity)
348	Benz (2022) (ID:87857721)	Consideration of Contextual Factors in Understanding Treatment-Seeking for Problematic Substance Use	-EXCLUDE on population (women)	
349	Benziger (2015) (ID:87853390)	Projected growth of the adult congenital heart disease population in the United States to 2050: an integrative systems modeling approach	-EXCLUDE on population (no housing precarity)	
350	Berends (2016) (ID:87850953)	Social disadvantage and past treatment among clients entering public alcohol and drug services in two Australian States.	-EXCLUDE on population (women)	
351	Berg (2018) (ID:87848525)	Medical Records Flag for Suicide Risk: Predictors and Subsequent Use of Care Among Veterans With Substance Use Disorders.	-EXCLUDE on population (women)	
352	Berke (2022) (ID:87963216)	Men's psychiatric distress in context: Understanding the impact of masculine discrepancy stress, race, and barriers to help-seeking	-EXCLUDE on population (women)	
353	Berman (2015) (ID:87851040)	Parental loss and residential instability: The impact on young women from low-income households in Detroit.	-INCLUDE on title & abstract	-EXCLUDE on intervention (service engagement/helpseeking behaviour)
354	Bermudez (2017) (ID:87852004)	When weary L.A. activists need relief from Trump, this is where they go	-EXCLUDE on evidence and form (evidence not in written form or presented as research output)	
355	Bernard (2021) (ID:87963124)	Intersectionality for social workers: A practical introduction to theory and practice	-EXCLUDE on intervention (service engagement/helpseeking behaviour)<br>-EXCLUDE - but review for literature	
356	Bernardelli (2021) (ID:87851501)	Distribuição espacial e espaço temporal de óbitos de mulheres vivendo com HIV em Porto Alegre de 2007 a 2017. (Portuguese)	-EXCLUDE on country (High-Income)	
357	Bernardelli (2021) (ID:87853213)	Spatial and spatio-temporal distribution o women living with HIV mortality in Porto Alegre, Brazil, from 2007 to 2017	-EXCLUDE on country (High-Income)	
358	Berry (2015) (ID:87852016)	Development of Existential-Humanistic multiculturally- and class-informed recommendations for working with persons experiencing homelessness and/or poverty	-EXCLUDE on population (women)	
359	Berry (2016) (ID:87850950)	Development of existential-humanistic multiculturally- and class-informed recommendations for working with persons experiencing homelessness and/or poverty.	-EXCLUDE on intervention (service engagement/helpseeking behaviour)	
360	Berry (2021) (ID:87848178)	Association between Participation in Counseling and Retention in a Buprenorphine-Assisted Treatment Program for People Experiencing Homelessness with Opioid Use Disorder.	-EXCLUDE on population (women)	
361	BEST (2016) (ID:87857365)	Social work and social media: online help-seeking and the mental well-being of adolescent males	-EXCLUDE on population (women)	
362	Betancourt (2022) (ID:87850599)	Homelessness and race as predictors of stigma against men with serious mental illness.	-EXCLUDE on population (women)	
363	Betron (2018) (ID:87857678)	Expanding the agenda for addressing mistreatment in maternity care: a mapping review and gender analysis	-EXCLUDE on population (no housing precarity)	
364	Bhalla (2020) (ID:87848267)	Social determinants of mental health care systems: intensive community based Care in the Veterans Health Administration.	-EXCLUDE on population (women)	
365	Bhalla (2021) (ID:87848242)	Psychiatric Multimorbidity in a Specialized Program for Severely Mentally Ill Veterans.	-EXCLUDE on population (women)	
366	Bhandari (2020) (ID:87963224)	Identification of abuse experiences of South Asian women in the US and women in Mumbai, India	-EXCLUDE on country (High-Income)	
367	Bharel (2013) (ID:87849003)	Health care utilization patterns of homeless individuals in Boston: preparing for Medicaid expansion under the Affordable Care Act.	-EXCLUDE on population (women)	
368	Bhat (2021) (ID:87851884)	Fear, discrimination, and healthcare access during the COVID-19 pandemic: Exploring women domestic workers' lives in India	-EXCLUDE on country (High-Income)	
369	BHATTACHARYA (2004) (ID:87857327)	Health care seeking for HIV/AIDS among South Asian in the United States	-EXCLUDE on date (2010)	
370	Bhattacharya (2019) (ID:87857724)	Women's Narratives on Illness and Institutionalization in India: A Feminist Inquiry	-EXCLUDE on country (High-Income)	

371	Bhattacharya (2022) (ID:87851587)	Stakeholders facilitating hope and empowerment amidst social suffering: A qualitative documentary analysis exploring lives of homeless women with mental illness.	-INCLUDE on title & abstract	-EXCLUDE on country (High-income)	
372	Bhugra (2015) (ID:87851019)	Home is where hearth is.	-EXCLUDE on intervention (service engagement/helpseeking behaviour)		
373	Biagioli (2021) (ID:87848234)	Living with cancer in the COVID-19 pandemic: An Italian survey on self-isolation at home.	-EXCLUDE on population (no housing precarity)		
374	BIDDLE (2004) (ID:87857244)	Factors influencing help seeking in mentally distressed young adults: a cross-sectional survey	-EXCLUDE on date (2010)		
375	Biddyr (2015) (ID:87848858)	Preventing sight loss in older people. A qualitative study exploring barriers to the uptake of regular sight tests of older people living in socially deprived communities in South Wales.	-EXCLUDE on population (no gender focus; women population <50)		
376	Biederman (2012) (ID:87852036)	Understanding the experience of interacting with service providers from the perspective of homeless women: A phenomenological study	-EXCLUDE Duplicate	-EXCLUDE on intervention (intersectionality)	
377	Biederman (2013) (ID:87851168)	Homeless women's experiences of social support from service providers.	-INCLUDE on title & abstract	-EXCLUDE on intervention (intersectionality)	
378	Biederman (2013) (ID:87852026)	Understanding the experience of interacting with service providers from the perspective of homeless women: A phenomenological study	-EXCLUDE Duplicate		
379	Biederman (2019) (ID:87848443)	Health care utilization following a homeless medical respite pilot program.	-EXCLUDE on population (no gender focus; women population <50)		
380	Biello (2018) (ID:87848492)	Perspectives on HIV pre-exposure prophylaxis (PrEP) utilization and related intervention needs among people who inject drugs.	-EXCLUDE on population (no gender focus; women population <50)		
381	Biello (2019) (ID:87848437)	A missing perspective: injectable pre-exposure prophylaxis for people who inject drugs.	-EXCLUDE on population (women)		
382	Biello (2021) (ID:87848196)	Delivering Preexposure Prophylaxis to People Who Use Drugs and Experience Homelessness, Boston, MA, 2018-2020.	-EXCLUDE on population (women)		
383	Bige (2015) (ID:87848855)	Homeless Patients in the ICU: An Observational Propensity-Matched Cohort Study.	-EXCLUDE on population (no gender focus; women population <50)		
384	Bilotta (2023) (ID:87857953)	‘Respect’ and ‘justice’ for whom? Culturally irresponsive ethical practices with refugee communities	-EXCLUDE on country (High-Income)		
385	**BIMPSON (2020) (ID:87856932)**	**Homeless mothers: key research findings**	**-INCLUDE on title & abstract**	**-INCLUDE on full study**	**--> study included (Bimpson, Emma, Sadie Parr, and Kesia Reeve. "Governing homeless mothers: the unmaking of home and family." Housing Studies 37.2 (2022): 272-291.)**
386	BIMPSON (2021) (ID:87856910)	Women, homelessness and violence: what works?	-INCLUDE on title & abstract	-EXCLUDE on intervention (intersectionality)	
387	Bindley (2021) (ID:87857862)	Social welfare needs of bereaved Australian carers: Implications of insights from palliative care and welfare workers	-EXCLUDE on population (no housing precarity)		
388	Bingham (2019) (ID:87850739)	Gender differences among Indigenous Canadians experiencing homelessness and mental illness.	-INCLUDE on title & abstract	-EXCLUDE on intervention (service engagement/helpseeking behaviour)	
389	Birchall (2021) (ID:87963160)	Intersectionality and responses to Covid-19	-EXCLUDE - but review for literature<br>-EXCLUDE on intervention (service engagement/helpseeking behaviour)		
390	Bird (2021) (ID:87857797)	“I couldn’t move forward if I didn’t look back”: Visual Expression and Transitional Stories of Domestic Violence	-INCLUDE on title & abstract	-EXCLUDE on intervention (service engagement/helpseeking behaviour)	
391	Bitter (2017) (ID:87850856)	How effective is the comprehensive approach to rehabilitation (CARe) methodology? A cluster randomized controlled trial.	-EXCLUDE on population (women)		
392	Bitter (2019) (ID:87848456)	Training professionals in a recovery-oriented methodology: a mixed method evaluation.	-EXCLUDE on intervention (service engagement/helpseeking behaviour)		
393	Bitton (2018) (ID:87853258)	Battered women in Orthodox communities in Israel: Social support, posttraumatic stress symptoms, and coping styles	-INCLUDE on title & abstract	-EXCLUDE on intervention (service engagement/helpseeking behaviour)	
394	Blackstock (2012) (ID:87851280)	Gender and the use of veterans health administration homeless services programs among Iraq/Afghanistan veterans.	-EXCLUDE on population (no gender focus; women population <50)		

395	Blagg (2018) (ID:87963197)	Innovative models in addressing violence against Indigenous women	-INCLUDE on title & abstract	-EXCLUDE on target group (not focused on women's behaviour/outcomes for women)
396	Blais (2012) (ID:87849308)	Love without a home: A portrait of romantic and couple relationships among street-involved young adults in Montreal	-EXCLUDE on intervention (service engagement/helpseeking behaviour)	
397	Blanch (2011) (ID:87851299)	Innovative program approaches to refugee trauma.	-EXCLUDE on population (no gender focus; women population <50)<br>-EXCLUDE - but review for literature	
398	BLAY (2008) (ID:87857220)	Knowledge and beliefs about help-seeking behavior and helpfulness of interventions for Alzheimer's disease	-EXCLUDE on population (no housing precarity)	
399	Blewer (2018) (ID:87853237)	Gender Disparities Among Adult Recipients of Bystander Cardiopulmonary Resuscitation in the Public	-EXCLUDE on intervention (service engagement/helpseeking behaviour)	
400	Block (2022) (ID:87857472)	"It's about Building a Network of Support": Australian Service Provider Experiences Supporting Refugee Survivors of Sexual and Gender-Based Violence	-INCLUDE on title & abstract	-EXCLUDE (IPV/DVA but little discussion on housing)
401	BLOOD (2004) (ID:87857046)	Older women and domestic violence: a report for Help the Aged/hact	-EXCLUDE on date (2010)	
402	Bloomquist (2017) (ID:87857983)	Doin' Meth or Doin' Math: What Client Constructions of Social Class Mean for Social Work Practice	-EXCLUDE on population (women)	
403	Blundo (2015) (ID:87852010)	Solution-Focused Case Management	-EXCLUDE on evidence and form (evidence not in written form or presented as research output)	
404	Bo (2010) (ID:87849279)	An interactional perspective on the relationship of immigration to intimate partner violence in a representative sample of help-seeking women.	-INCLUDE on title & abstract	-EXCLUDE on intervention (service engagement/helpseeking behaviour)
405	Bockey (2020) (ID:87848276)	Patient satisfaction & use of health care: a cross-sectional study of asylum seekers in the Freiburg initial reception centre.	-EXCLUDE on population (women)	
406	Bockting (2020) (ID:87963275)	Engagement and retention in HIV care for transgender women: perspectives of medical and social service providers in New York City	-INCLUDE on title & abstract	-EXCLUDE on target group (no housing precarity)
407	Boddington (2021) (ID:87848208)	Epidemiological and clinical characteristics of early COVID-19 cases, United Kingdom of Great Britain and Northern Ireland.	-EXCLUDE on population (no housing precarity)	
408	Boden (2021) (ID:88019130)	Exploring the barriers and facilitators to menstrual hygiene management for women experiencing homelessness	-INCLUDE on title & abstract	-EXCLUDE on intervention (intersectionality)
409	Boden (2023) (ID:87963091)	Exploring the barriers and facilitators to menstrual hygiene management for women experiencing homelessness	-EXCLUDE Duplicate	
410	Bøg (2017) (ID:87851934)	12-step programs for reducing illicit drug use	-EXCLUDE on population (women)	
411	Boggiano (2017) (ID:87851935)	PROTECTING, BALANCING, AND CONFRONTING: HEALTH-SEEKING AMONG HOMELESS YOUTH IN HO CHI MINH CITY, VIETNAM	-EXCLUDE on country (High-Income)	
412	Bohane (2013) (ID:87850539)	Mental health and homelessness : the role of self control	-EXCLUDE on population (women)	
413	Bohnert (2010) (ID:87849283)	Friendship networks of inner-city adults: a latent class analysis and multi-level regression of supporter types and the association of supporter latent class membership with supporter and recipient drug use.	-EXCLUDE on population (no gender focus; women population <50)	
414	Bojorquez-Chapela (2022) (ID:87848164)	The impact of the COVID-19 pandemic among migrants in shelters in Tijuana, Baja California, Mexico.	-EXCLUDE on country (High-Income)	
415	BOLDERO (1995) (ID:87857147)	Adolescent help-seeking: what do they get help for and from whom?	-EXCLUDE on date (2010)	
416	Bole (2021) (ID:87849372)	Characterization of patients seeking care at a sexual health clinic who report engaging in exchange sex	-EXCLUDE on population (no gender focus; women population <50)	
417	Bonevski (2011) (ID:87849212)	RCT of a client-centred, caseworker-delivered smoking cessation intervention for a socially disadvantaged population.	-EXCLUDE on population (women)	
418	Bonevski (2012) (ID:87849123)	Addressing smoking and other health risk behaviours using a novel telephone-delivered intervention for homeless people: a proof-of-concept study.	-EXCLUDE on population (women)	
419	BONFIELD (2010) (ID:87857238)	Help-seeking by foster-carers for their 'looked after' children: the role of mental health literacy and treatment attitudes	-EXCLUDE on population (no housing precarity)	

420	Bongue (2016) (ID:87852920)	ASSESSMENT OF HEALTH INEQUALITIES AMONG OLDER PEOPLE USING THE EPICES SCORE: A COMPOSITE INDEX OF SOCIAL DEPRIVATION	-EXCLUDE on population (no housing precarity)	
421	Boodhoo (2016) (ID:87850533)	Homeless young mothers' experiences of their relationship with their children : an interpretative phenomenological study	-INCLUDE on title & abstract	-EXCLUDE on intervention (service engagement/helpseeking behaviour)
422	Boodram (2015) (ID:87848837)	The role of social networks and geography on risky injection behaviors of young persons who inject drugs.	-EXCLUDE on population (women)	
423	Boreham (2019) (ID:87963122)	".. that warm feeling that [alcohol] gave me was what I interpreted love would feel like.." Lived experience of excessive alcohol use and care proceedings by mothers in the family justice system in the UK	-EXCLUDE on population (no housing precarity)	
424	Borges (2022) (ID:87963094)	The Role of Social Determinants of Health in Moral Injury: Implications and Future Directions	-EXCLUDE on population (no housing precarity)	
425	Borghi (2023) (ID:87853179)	The perceived impact of homelessness on health during pregnancy and the postpartum period: A qualitative study carried out in the metropolitan area of Nantes, France	-INCLUDE on title & abstract	-EXCLUDE on intervention (service engagement/helpseeking behaviour)
426	Borgschulte (2018) (ID:87848521)	Health care provision for refugees in Germany - one-year evaluation of an outpatient clinic in an urban emergency accommodation.	-EXCLUDE on population (no gender focus; women population <50)<br>-EXCLUDE - but review for literature	
427	Boscaljon (2021) (ID:87857947)	Beyond Idolatrous Whiteness II: Beholding Beauty	-EXCLUDE on intervention (service engagement/helpseeking behaviour)	
428	Boscarino (2013) (ID:87848993)	Mental health outcomes at the Jersey Shore after Hurricane Sandy.	-EXCLUDE on population (women)	
429	Bosch (2019) (ID:87858002)	Paving the Way: Advice from the Career Journeys of LGBTQ Professionals in Higher Education	-EXCLUDE on population (no housing precarity)	
430	Bosley-Smith (2023) (ID:87852870)	Anticipatory Economic Stressors: Perceived and Potential Sources of Economic Disadvantage for LGBTQ Adults	-EXCLUDE on population (no housing precarity)	
431	Bottino (2019) (ID:87848420)	The Relationship Between Housing Instability and Poor Diet Quality Among Urban Families.	-EXCLUDE on population (women)	
432	Bottino (2019) (ID:87848426)	Child Life Specialists' Experiences Addressing Social Determinants of Health: A Web-Based Survey.	-INCLUDE on title & abstract	-EXCLUDE on target group (not focused on women's behaviour/outcomes for women)
433	Boucher (2005) (ID:87850522)	Housing's insignificant others : the experience of homelessness for lesbians and bisexual women	-EXCLUDE on date (2010)	
434	Bould (2019) (ID:87848390)	Factors associated with increases over time in the quality of Active Support in supported accommodation services for people with intellectual disabilities: A multi-level model.	-EXCLUDE on population (women)	
435	Boulos (2023) (ID:87857535)	Obstacles to and opportunities for protecting human rights at the city level: The case of Madrid City Council Human Rights Plan (2017-2019)	-EXCLUDE on intervention (service engagement/helpseeking behaviour)	
436	Bounds (2020) (ID:87963069)	Adapting a family intervention to reduce risk factors for sexual exploitation	-INCLUDE on title & abstract	-EXCLUDE on target group (not focused on women's behaviour/outcomes for women)
437	Bouvier (2017) (ID:87848662)	Willingness to use a supervised injection facility among young adults who use prescription opioids non-medically: a cross-sectional study.	-EXCLUDE on population (women)	
438	Bowe (2015) (ID:87851024)	PTSD and substance use disorder among veterans: Characteristics, service utilization and pharmacotherapy.	-EXCLUDE on population (women)	
439	Bowe (2022) (ID:87858045)	Running with Dogs and Sitting with Vultures: A Heuristic Self-Search Inquiry on the Experience of Intentionally Healing Body Image Dissatisfaction from a Spiritual Ecofeminist, Weight-Inclusive Perspective	-EXCLUDE on population (no housing precarity)	
440	Bowen (2015) (ID:87850987)	Predictors of stable housing for homeless women leaving a sex work-exiting program.	-EXCLUDE on intervention (service engagement/helpseeking behaviour)	
441	Bowen (2016) (ID:87853294)	Prevalence and covariates of food insecurity among residents of single-room occupancy housing in Chicago, IL, USA	-EXCLUDE on population (women)	
442	Bowen (2019) (ID:87848440)	Food Insecurity Among Formerly Homeless Individuals Living in Permanent Supportive Housing.	-EXCLUDE on population (women)	
443	Bowen (2021) (ID:87963332)	Toward many emerging adulthoods: A theory-based examination of the features of emerging adulthood for cross-systems youth	-EXCLUDE on population (no housing precarity)	

444	Bowen (2022) (ID:87853041)	Rural Food Insecurity: A Longitudinal Analysis of Low-Income Rural Households with Children in the South	-EXCLUDE on population (women)	
445	Bower (2018) (ID:87850783)	Australian homeless persons' experiences of social connectedness, isolation and loneliness.	-EXCLUDE on population (women)	
446	BOWLER (2010) (ID:87857326)	A clear view ahead...	-EXCLUDE on population (no gender focus; women population <50)<br>-EXCLUDE - but review for literature	
447	Bowling (2020) (ID:87857932)	"It's the nature of the beast": Community resilience among gender diverse individuals	-EXCLUDE on population (no housing precarity)	
448	Bowman (2023) (ID:87858122)	Be a Good Girl or Else: The Racialization of Black Girls as a Problem and the Trigger Response to Punish or Subdue Them Into Compliance	-EXCLUDE on population (no housing precarity)	
449	Box (2022) (ID:88019142)	Women sleeping rough: The health, social and economic costs of homelessness	-INCLUDE on title & abstract	-EXCLUDE on target group (not focused on women's behaviour/outcomes for women)
450	BOYCOTT (2015) (ID:87857406)	"Work is good for me": views of mental health service users seeking work during the UK recession, a qualitative analysis	-EXCLUDE on population (women)	
451	Boyd (2015) (ID:87848882)	Emergency department use among HIV-infected released jail detainees.	-EXCLUDE on population (no gender focus; women population <50)	
452	Boyd (2017) (ID:87848634)	Social-structural factors influencing periods of injection cessation among marginalized youth who inject drugs in Vancouver, Canada: an ethno-epidemiological study.	-EXCLUDE on population (women)<br>-EXCLUDE - but review for literature	
453	**Boyd (2018) (ID:87857436)**	**Gendered violence and overdose prevention sites: a rapid ethnographic study during an overdose epidemic in Vancouver, Canada**	**-INCLUDE on title & abstract**	**-INCLUDE on full study**
454	Brabeck (2021) (ID:87857811)	Open Science and Feminist Ethics: Promises and Challenges of Open Access	-EXCLUDE on population (no housing precarity)	
455	BRADLEY (2012) (ID:87857210)	Adolescent help-seeking for psychological distress, depression, and anxiety using an Internet program	-EXCLUDE on population (people aged under 18 years)	
456	BRAFIELD (2008) (ID:87857232)	Service user involvement: reaching the hard to reach in supported housing	-EXCLUDE on date (2010)	
457	**Brais (2022) (ID:87849370)**	**Inside the Open Door: Considerations of Inclusivity Among Women Accessing an Open Door Housing Service in Canada**	**-INCLUDE on title & abstract**	**-INCLUDE on full study**
458	Bralock (2011) (ID:87851852)	Issues in community-based care among homeless minorities.	-EXCLUDE on population (no gender focus; women population <50)<br>-EXCLUDE - but review for literature	
459	Brankovic (2013) (ID:87853257)	Applying a gender lens on human papillomavirus infection: cervical cancer screening, HPV DNA testing, and HPV vaccination	-EXCLUDE on population (no housing precarity)	
460	BRANNEN (1980) (ID:87857032)	Seeking help for management problems	-EXCLUDE on date (2010)	
461	Brassard (2015) (ID:87963270)	Application of intersectional analysis to data on domestic violence against aboriginal women living in remote communities in the province of Quebec	-INCLUDE on title & abstract	-EXCLUDE on intervention (service engagement/helpseeking behaviour)
462	Brauer (2011) (ID:87852042)	A Review of Psychotherapy for Obsessive-Compulsive Disorder: The Journal of Psychiatry	-EXCLUDE on population (no housing precarity)	
463	BRAUN (1998) (ID:87857395)	Perceptions of dementia, caregiving and help seeking among Asian and Pacific Islander Americans	-EXCLUDE on population (no housing precarity)	
464	Braveman (2010) (ID:87849289)	Poverty, near-poverty, and hardship around the time of pregnancy.	-INCLUDE on title & abstract	-EXCLUDE on target group (no housing precarity)
465	Bravo (2014) (ID:87851687)	Superviviencia del cancer en Cali: Estudio poblacional, 1995-2004	-EXCLUDE on country (High-Income)	
466	Breland (2015) (ID:87850975)	Racial differences in chronic conditions and sociodemographic characteristics among high-utilizing veterans.	-EXCLUDE on intervention (service engagement/helpseeking behaviour)	
467	Brenisin (2021) (ID:87857545)	The impact of inequality on mental illness: thematic analysis on clinical notes	-EXCLUDE on population (women)<br>-EXCLUDE - but review for literature	
468	Bretherton (2016) (ID:88019122)	Women's Homelessness and Welfare States	-INCLUDE on title & abstract	-EXCLUDE on intervention (intersectionality)
469	Bretherton (2017) (ID:87850517)	Gender dimensions in conceptualisations of homelessness : theoretical and operational (in)visibility	-INCLUDE on title & abstract	-EXCLUDE on intervention (service engagement/helpseeking behaviour)
470	BRETHERTON (2018) (ID:87856937)	Women and rough sleeping: a critical review of current research and methodology	-INCLUDE on title & abstract	-EXCLUDE on intervention (intersectionality)

471	Bretherton (2020) (ID:88019116)	Women's Experiences of Homelessness: A Longitudinal Study	-INCLUDE on title & abstract	-EXCLUDE on intervention (intersectionality)
472	Bretherton (2021) (ID:88019118)	Women's Homelessness: European Evidence Review	-INCLUDE on title & abstract	-EXCLUDE on evidence and form (literature review)
473	Bretherton (2021) (ID:88019154)	Women's Homelessness in Camden: Improving Data, Strategy and Outcomes	-INCLUDE on title & abstract	-EXCLUDE on intervention (service engagement/helpseeking behaviour)
474	Brett (2014) (ID:87848914)	Multimorbidity in a marginalised, street-health Australian population: a retrospective cohort study.	-EXCLUDE on intervention (service engagement/helpseeking behaviour)	
475	Brettel (2022) (ID:87851743)	[Health Service Utilization by Homeless Persons: Analysis of the Role of Enabling Factors, Pain and Gender using the Gelberg-Andersen Model].	-EXCLUDE on population (no gender focus; women population <50)	
476	Brickell (2019) (ID:87857978)	Feminist geolegality	-EXCLUDE on population (no housing precarity)	
477	BRIDGEMAN (2020) (ID:87856993)	Don't let me fall through the cracks: homelessness amongst care-experienced young people in Wales	-EXCLUDE on population (people aged under 18 years)	
478	Briggs (2015) (ID:87857964)	Racial Respect: Initial Testing and Validation of the Racial Respect Scale	-EXCLUDE on population (no housing precarity)	
479	Brinkley-Rubinstein (2013) (ID:87853451)	Health Impact of Incarceration on HIV-Positive African American Males: A Qualitative Exploration	-EXCLUDE on population (women)	
480	Brinkley-Rubinstein (2013) (ID:87858044)	Incarceration as a catalyst for worsening health	-EXCLUDE on population (women)	
481	Brinkley-Rubinstein (2015) (ID:87857734)	Understanding the effects of multiple stigmas among formerly incarcerated HIV-positive african american men	-EXCLUDE on population (women)	
482	Broadus (2014) (ID:87851046)	Legal issues.	-EXCLUDE on population (no housing precarity)	
483	Brody (2021) (ID:87848203)	Enhanced Telehealth Case Management Plus Emergency Financial Assistance for Homeless-Experienced People Living With HIV During the COVID-19 Pandemic.	-EXCLUDE on population (women)	
484	Bromfield (2021) (ID:87853005)	At the Intersection of COVID-19 and Sex Work in the United States: A Call for Social Work Action	-INCLUDE on title & abstract	-EXCLUDE on intervention (service engagement/helpseeking behaviour)
485	Brooks (2017) (ID:87848624)	The association between discharge status, mental health, and substance misuse among young adult veterans.	-EXCLUDE on population (women)	
486	Brothers (2020) (ID:87848339)	Food insecurity among formerly homeless youth in supportive housing: A social-ecological analysis of a structural intervention.	-EXCLUDE on intervention (service engagement/helpseeking behaviour)	
487	Broto (2021) (ID:87857681)	Queering participatory planning	-EXCLUDE on intervention (service engagement/helpseeking behaviour)	
488	Brott (2023) (ID:87852863)	Reproductive justice for unhoused women: An integrative review of the literature	-INCLUDE on title & abstract	-EXCLUDE on target group (not focused on women's behaviour/outcomes for women)<br>-EXCLUDE (systematic review)* only use with other code
489	Brown (2012) (ID:87849087)	Associations between human rights environments and healthy longevity: the case of older persons in China.	-EXCLUDE on country (High-Income)	
490	Brown (2013) (ID:87849024)	Factors associated with geriatric syndromes in older homeless adults.	-EXCLUDE on population (women)	
491	Brown (2013) (ID:87849038)	Characteristics of emergency department visits by older versus younger homeless adults in the United States.	-EXCLUDE on population (women)	
492	Brown (2013) (ID:87849052)	Use of outpatient mental health services by homeless veterans after hurricanes.	-EXCLUDE on population (women)	
493	Brown (2014) (ID:87851051)	Racial health disparities in a cohort of 5,135 transgender veterans.	-EXCLUDE on population (women)	
494	Brown (2014) (ID:87851098)	Predictors of employment among sheltered homeless women.	-EXCLUDE on intervention (service engagement/helpseeking behaviour)	
495	Brown (2015) (ID:87848804)	Health Correlates of Criminal Justice Involvement in 4,793 Transgender Veterans.	-EXCLUDE on intervention (service engagement/helpseeking behaviour)	
496	Brown (2016) (ID:87848795)	Systematic review of barriers and facilitators to accessing and engaging with mental health care among at-risk young people.	-EXCLUDE on population (no gender focus; women population <50)<br>-EXCLUDE - but review for literature	
497	Brown (2016) (ID:87850949)	Mental health and medical health disparities in 5135 transgender veterans receiving healthcare in the veterans health administration: A case-control study.	-EXCLUDE on population (no housing precarity)	
498	Brown (2017) (ID:87848645)	Applying a Time-Patterned Typology of Homelessness Among Individuals with Mental Illness.	-EXCLUDE - but review for literature<br>-EXCLUDE on population (no gender focus; women population <50)	

499	Brown (2017) (ID:87963208)	An analysis of factors that affect African American women with mental illness	-EXCLUDE on intervention (service engagement/helpseeking behaviour)	
500	BROWN (2017) (ID:87851423)	A critical evaluation of the "short stay project" - service users' perspectives	-EXCLUDE on population (women)	
501	Brown (2019) (ID:87853397)	Association of Functional Impairment in Middle Age With Hospitalization, Nursing Home Admission, and Death	-EXCLUDE on population (no housing precarity)	
502	Brown (2020) (ID:87848254)	Family Relationships and the Health and Well-Being of Transgender and Gender-Diverse Youth: A Critical Review.	-EXCLUDE on intervention (service engagement/helpseeking behaviour)	
503	Brown (2020) (ID:87848347)	Sociodemographic and substance use characteristics associated with typologies and composition of social support networks among youth experiencing homelessness in Los Angeles, USA.	-EXCLUDE on population (no gender focus; women population <50)	
504	Brown (2021) (ID:87857656)	The Personal Accounts of Trauma, Stress, and Patient-Provider Relationships of African American Women with Fibroids	-EXCLUDE on population (no housing precarity)	
505	Brown (2022) (ID:87848148)	Factors Associated With Mortality Among Homeless Older Adults in California: The HOPE HOME Study.	-EXCLUDE on population (women)	
506	Brown (2022) (ID:87851967)	Examining Factors of Rehabilitation for Survivors of Sexual Trafficking: Qualitatively Studying the Perspective of Professional Advocates	-INCLUDE on title & abstract	-EXCLUDE on intervention (service engagement/helpseeking behaviour)
507	Browne (2012) (ID:87963254)	Closing the health equity gap: evidence-based strategies for primary health care organizations	-EXCLUDE on population (women)	
508	Browne-Miller (2012) (ID:87851215)	Violence and abuse in society: Understanding a global crisis, Vol 1: Fundamentals, effects, and extremes, Vol 2: Setting, age, gender, and other key elements, Vol 3: Psychological, ritual, sexual, and trafficking issues, Vol 4: Faces of intimate partner violence.	-EXCLUDE on intervention (service engagement/helpseeking behaviour)	
509	Brownstein (2017) (ID:87853004)	Evidence-based Advocacy in Action: Improving Access to Public Housing for Single Parents and their Children in Singapore	-EXCLUDE on intervention (service engagement/helpseeking behaviour)	
510	Brucker (2017) (ID:87848674)	The association of food insecurity with health outcomes for adults with disabilities.	-EXCLUDE on population (women)	
511	BRUCKNER (2004) (ID:87857162)	Changing Europe and the relevance of care and the caring professions	-EXCLUDE on date (2010)	
512	Bruffell (2006) (ID:87850543)	Baby love : self-evaluation processes of young mothers	-EXCLUDE on population (no housing precarity)	
513	BRUNETTE (1998) (ID:87857020)	Gender differences in homeless persons with schizophrenia and substance abuse	-EXCLUDE on date (2010)	
514	Brunson (2017) (ID:87853348)	Maternal, Newborn, and Child Health After the 2015 Nepal Earthquakes: An Investigation of the Long-term Gendered Impacts of Disasters	-EXCLUDE on country (High-Income)	
515	Brush (2018) (ID:87851919)	Understanding Trauma Normativeness, Normalization, and Help Seeking in Homeless Mothers	-INCLUDE on title & abstract	-EXCLUDE on target group (not focused on women's behaviour/outcomes for women)<br>-EXCLUDE (systematic review)* only use with other code
516	Brusilovskiy (2012) (ID:87849124)	A study of environmental influences on the well-being of individuals with psychiatric disabilities in Philadelphia, PA.	-EXCLUDE on population (women)	
517	Bryan (2023) (ID:87851453)	Epidemiological Features and Outcomes of HTLV-1 Carriers Diagnosed With Cancer: A Retrospective Cohort Study in an Endemic Country	-EXCLUDE on country (High-Income)	
518	Bryant (2011) (ID:87851301)	A systematic review and meta-analysis of the effectiveness of behavioural smoking cessation interventions in selected disadvantaged groups.	-EXCLUDE on population (no gender focus; women population <50)	
519	Bryant-Davis (2017) (ID:87857783)	Cultural Oppression and Human Trafficking: Exploring the Role of Racism and Ethnic Bias	-EXCLUDE on population (no gender focus; women population <50)	
520	Bryant-Davis (2021) (ID:87857685)	Resist and Rise: A Trauma-Informed Womanist Model for Group Therapy	-EXCLUDE on population (no housing precarity)	
521	Bryden-Currie (2021) (ID:87850655)	Homelessness and HIV risk: Experiences, perceptions, and beliefs of transgender youth.	-INCLUDE on title & abstract	-EXCLUDE on intervention (service engagement/helpseeking behaviour)
522	Bryere (2014) (ID:87853268)	Socioeconomic environment and cancer incidence: a French population-based study in Normandy	-EXCLUDE on intervention (service engagement/helpseeking behaviour)	

523	Bucciferro (2021) (ID:87857575)	Representations of gender and race in Ryan Coogler's film Black Panther: disrupting Hollywood tropes	-EXCLUDE on intervention (service engagement/helpseeking behaviour)	
524	Buchholz (2010) (ID:87851377)	Associations of housing status with substance abuse treatment and service use outcomes among veterans.	-EXCLUDE on population (women)	
525	BUCK (2002) (ID:87856994)	Repeat homelessness and domestic abuse	-EXCLUDE on date (2010)	
526	Buck (2012) (ID:87851235)	Comparing homeless and domiciled patients' utilization of the Harris County, Texas public hospital system.	-EXCLUDE on population (women)	
527	BUCK (2015) (ID:87857202)	The importance of family and friends in advice-seeking for rights problems	-EXCLUDE on population (no housing precarity)	
528	Buckingham (2018) (ID:87857496)	Shared Communities: A Multinational Qualitative Study of Immigrant and Receiving Community Members	-EXCLUDE on intervention (service engagement/helpseeking behaviour)	
529	Buenrostro (2016) (ID:87857950)	A prevention program for Latina teens at risk for sexual assault: A grant application project	-EXCLUDE on evidence and form (evidence not in written form or presented as research output)	
530	Buente (2020) (ID:87852740)	Examining Factors Associated With Facebook Use Among Sheltered Homeless in Hawai'i	-EXCLUDE on population (no gender focus; women population <50)	
531	BUHRICH (2003) (ID:87857031)	Schizophrenia among homeless people in inner-Sydney: current prevalence and historical trends	-EXCLUDE on date (2010)	
532	Bukowski (2011) (ID:87851353)	Making the invisible visible: A photovoice exploration of homeless women's health and lives in central Auckland.	-INCLUDE on title & abstract	-EXCLUDE on intervention (intersectionality)
533	Bukowski (2018) (ID:87853242)	Characterizing the HIV Care Continuum and Identifying Barriers and Facilitators to HIV Diagnosis and Viral Suppression Among Black Transgender Women in the United States	-INCLUDE on title & abstract	-EXCLUDE on target group (not focused on women's behaviour/outcomes for women)
534	BULLETIN BOARD (2012) (ID:87852030)	BULLETIN BOARD	-EXCLUDE on evidence and form (evidence not in written form or presented as research output)	
535	BULLETIN BOARD (2013) (ID:87852024)	BULLETIN BOARD	-EXCLUDE on evidence and form (evidence not in written form or presented as research output)	
536	Bullock (2020) (ID:87857414)	An intersectional analysis of the feminization of homelessness and mothers' housing precarity	-INCLUDE on title & abstract	-EXCLUDE on intervention (service engagement/helpseeking behaviour)
537	Bungay (2010) (ID:87849277)	Women's health and use of crack cocaine in context: structural and 'everyday' violence.	-INCLUDE on title & abstract	-EXCLUDE on target group (no housing precarity)
538	Bungay (2013) (ID:87852742)	Health Care Among Street-Involved Women: The Perpetuation of Health Inequity	-INCLUDE on title & abstract	-EXCLUDE on intervention (intersectionality)
539	Bunn (2019) (ID:87963121)	Intersectional needs and reentry: Re-conceptualizing 'multiple and complex needs' post-release	-EXCLUDE on population (women)<br>-EXCLUDE - but review for literature	
540	Buot (2014) (ID:87853082)	Beyond Race and Place: Distal Sociological Determinants of HIV Disparities	-EXCLUDE on population (women)	
541	Burak (2022) (ID:87852876)	PROFILE OF HOMELESS PEOPLE IN EMERGENCY DEPARTMENTS	-EXCLUDE on intervention (service engagement/helpseeking behaviour)	
542	Burczycka (2018) (ID:87857610)	Violent victimization of Canadians with mental health-related disabilities, 2014	-EXCLUDE on population (no housing precarity)	
543	Burke (2013) (ID:87849009)	Losing work: regional unemployment and its effect on homeless demographic characteristics, needs, and health care.	-EXCLUDE on population (women)	
544	Burkey (2011) (ID:87851355)	The role of social ties in recovery in a population of homeless substance abusers.	-EXCLUDE on population (women)	
545	BURLS (2008) (ID:87857203)	Seeking nature: a contemporary therapeutic environment	-EXCLUDE on date (2010)	
546	Burman (2016) (ID:87857786)	Multicultural Feeling, Feminist Rage, Indigenous Refusal	-EXCLUDE on population (no housing precarity)	
547	Burnett (2018) (ID:87963149)	Structural Justice: A critical feminist framework exploring the intersection between justice, equity and structural reconciliation.	-INCLUDE on title & abstract	-EXCLUDE on intervention (service engagement/helpseeking behaviour)
548	BURNS (2006) (ID:87857325)	Adolescent mental health literacy: young people's knowledge of depression and help seeking	-EXCLUDE on population (people aged under 18 years)	
549	Burns (2019) (ID:87857652)	Creating Conditions for Literate Engagement: Teaching, Learning, and Acting in the World	-EXCLUDE on population (no housing precarity)	
550	Burra (2012) (ID:87851240)	Homeless and housed inpatients with schizophrenia: Disparities in service access upon discharge from hospital.	-EXCLUDE on population (no gender focus; women population <50)	
551	Burrage (2021) (ID:87850564)	Trauma history and social support among American Indian/Alaska Native and non-Native survivors of intimate partner violence.	-EXCLUDE on population (no gender focus; women population <50)<br>-EXCLUDE - but review for literature	

552	Burruss-Cousins (2022) (ID:87857804)	The Interplay of Home Visitors' Personal and Professional Identities in Effectively Screening and Supporting Women Around Sensitive Topics	-EXCLUDE on intervention (service engagement/helpseeking behaviour)	
553	Burse (2022) (ID:87853197)	Domestic Violence Survivorship Among a Sample of Older African American Women: An Interpretative Phenomenological Analysis	-INCLUDE on title & abstract	-EXCLUDE (IPV/DVA but little discussion on housing)
554	Burstrom (2021) (ID:87853206)	EQ-5D-3L Health Status Among Homeless People in Stockholm, Sweden, 2006 and 2018	-EXCLUDE on intervention (service engagement/helpseeking behaviour)	
555	Burt (2012) (ID:87849134)	Impact of housing and work supports on outcomes for chronically homeless adults with mental illness: LA's HOPE.	-EXCLUDE on population (women)	
556	Burt (2017) (ID:87848630)	A Decline in HIV Testing Among Persons Who Inject Drugs in the Seattle Area, 2004-2015.	-EXCLUDE on intervention (service engagement/helpseeking behaviour)	
557	Burton (2018) (ID:87848597)	Outreach to veterans with serious mental illness who are lost to care: Predictors of outreach contact.	-EXCLUDE on population (women)	
558	Burtscher (2016) (ID:87848761)	"My Favourite Day Is Sunday": Community Perceptions of (Drug-Resistant) Tuberculosis and Ambulatory Tuberculosis Care in Kara Suu District, Osh Province, Kyrgyzstan.	-EXCLUDE on country (High-Income)	
559	Businelle (2013) (ID:87848999)	Comparing homeless smokers to economically disadvantaged domiciled smokers.	-EXCLUDE on population (women)	
560	Businelle (2015) (ID:87851036)	Smoking policy change at a homeless shelter: Attitudes and effects.	-EXCLUDE on population (women)	
561	Buster (2014) (ID:87848926)	Detainees in Amsterdam, a target population of the Public Mental Health System?.	-EXCLUDE on population (women)	
562	Buttram (2014) (ID:87851089)	Resilience and syndemic risk factors among African-American female sex workers.	-INCLUDE on title & abstract	-EXCLUDE on intervention (service engagement/helpseeking behaviour)
563	Butz (2022) (ID:87857428)	Intersectionality and Social Welfare: Avoidance and Unequal Treatment among Transgender Women of Color	-INCLUDE on title & abstract	-EXCLUDE on target group (no housing precarity)
564	Bwambale (2021) (ID:87848221)	Demographic and behavioural drivers of intra-urban mobility of migrant street children and youth in Kampala, Uganda.	-EXCLUDE on country (High-Income)	
565	Bwambale (2022) (ID:87853448)	Rural-Urban Migration, Childbearing Decision-Making, Fertility and Contraceptive Perspectives of Street Adolescents and Youth in Kampala, Uganda	-EXCLUDE on country (High-Income)	
566	Byrne (2016) (ID:87850960)	Unsheltered homelessness among Veterans: Correlates and profiles.	-EXCLUDE on population (women)	
567	Byrne (2019) (ID:87848451)	A Novel Measure to Assess Variation in Hepatitis C Prevalence Among Homeless and Unstably Housed Veterans, 2011-2016.	-EXCLUDE on intervention (service engagement/helpseeking behaviour)	
568	Byrne (2020) (ID:87848272)	A classification model of homelessness using integrated administrative data: Implications for targeting interventions to improve the housing status, health and well-being of a highly vulnerable population.	-EXCLUDE on population (women)	
569	Byrne (2022) (ID:87848150)	Performance of 2 Single-Item Screening Questions to Identify Future Homelessness Among Emergency Department Patients.	-EXCLUDE on intervention (service engagement/helpseeking behaviour)	
570	Caban (2013) (ID:87852029)	Use of mental health services among homeless African American men with HIV/AIDS and mental health needs	-EXCLUDE on population (women)	
571	Cabanas (2000) (ID:87853707)	El Mezquital: A Community's Struggle for Development	-EXCLUDE on date (2010)	
572	Cabassa (2013) (ID:87849021)	Picturing recovery: a photovoice exploration of recovery dimensions among people with serious mental illness.	-EXCLUDE on population (no gender focus; women population <50)<br>-EXCLUDE - but review for literature	
573	Cabrera (2023) (ID:87853330)	Determining factors in the overall happiness and outlook for the future of women living homeless: Evidence from Madrid, Spain	-EXCLUDE on intervention (service engagement/helpseeking behaviour)	
574	Cadet (2020) (ID:87857933)	Institutional thoughtlessness and the needs of older probation clients	-EXCLUDE on population (women)	
575	Caetano (2017) (ID:87853095)	Social Network of People with Addictive Behaviors and Dependencies	-EXCLUDE on population (women)	
576	Cahill (2018) (ID:87848592)	High Rates of Access to Health Care, Disclosure of Sexuality and Gender Identity to Providers Among House and Ball Community Members in New York City.	-EXCLUDE on population (no gender focus; women population <50)	
577	Calcaterra (2014) (ID:87848949)	The association between social stressors and drug use/hazardous drinking among former prison inmates.	-EXCLUDE on population (women)	

578	Call (2022) (ID:87853178)	Facilitators and barriers to PrEP use among straight and LGB young adults experiencing homelessness	-EXCLUDE on population (no gender focus; women population <50)	
579	Callahan (2015) (ID:87852934)	Introducing Sexual Orientation and Gender Identity Into the Electronic Health Record: One Academic Health Center's Experience	-EXCLUDE on population (no housing precarity)	
580	Callegari (2018) (ID:87848483)	Preconception Care in the Veterans Health Administration.	-INCLUDE on title & abstract	-EXCLUDE on target group (not focused on women's behaviour/outcomes for women)
581	Callejas (2021) (ID:87963207)	I Would Never Want to Live That Again	-INCLUDE on title & abstract	-EXCLUDE (IPV/DVA but little discussion on housing)
582	Callejo-Black (2021) (ID:87853002)	Eviction as a Disruptive Factor in Health Care Utilization: Impact on Hospital Readmissions and No-show Rates	-EXCLUDE on intervention (service engagement/helpseeking behaviour)	
583	Calvo (2017) (ID:87852784)	Using WhatsApp for a homeless count	-EXCLUDE on intervention (service engagement/helpseeking behaviour)	
584	Camacho (2012) (ID:87851279)	Crisis visits and psychiatric hospitalizations among patients attending a community clinic in rural southern California.	-EXCLUDE on population (women)	
585	Camacho (2023) (ID:87857902)	The Experience of Parental Self-Efficacy in First-Generation Mexican American Adult Catholic Mothers	-EXCLUDE on population (no housing precarity)	
586	CAMERON (2016) (ID:87856935)	From pillar to post: homeless women's experiences of social care	-INCLUDE on title & abstract	-EXCLUDE on intervention (intersectionality)
587	Cameron (2020) (ID:87963043)	Gender-based analysis plus (GBA+) and intersectionality: Overview, an enhanced framework, and a British Columbia case study	-EXCLUDE on population (no housing precarity)	
588	Camille (2020) (ID:87851518)	Non-COVID-19 Patients Left out in the Cold	-EXCLUDE on population (no housing precarity)	
589	CAMPBEL (2018) (ID:87857387)	A preliminary understanding of search words used by children, teenagers and young adults in seeking information about depression and anxiety online	-EXCLUDE on population (no housing precarity)	
590	Campbell (2021) (ID:87857806)	When the personal is not political: experiences of collective agency amongst participants in the domestic violence response in London, UK	-EXCLUDE on intervention (service engagement/helpseeking behaviour)	
591	Campbell (2023) (ID:87963256)	Creating a university strategic plan to address relationship violence and sexual misconduct (RVSM): An application of principles-focused evaluation at Michigan State University	-EXCLUDE on evidence and form (evidence not in written form or presented as research output)	
592	Campbell (2023) (ID:88019136)	"A Hidden Community": The Experiences of Help- Seeking and Receiving Mental Health Treatment in U.K. Women Veterans. A Qualitative Study	-INCLUDE on title & abstract	-EXCLUDE on intervention (intersectionality)
593	Cancer prevalence by... (Gigli) (ID:87851449)	Cancer prevalence by phase of care: an indicator for assessing health service needs	-EXCLUDE on population (no housing precarity)	
594	Cancer statistics for... (Miller) (ID:87851698)	Cancer statistics for adolescents and young adults, 2020.	-EXCLUDE on intervention (service engagement/helpseeking behaviour)	
595	Cancer survival in Cali,... (Bravo) (ID:87851699)	Cancer survival in Cali, Colombia: A population-based study, 1995-2004.	-EXCLUDE on country (High-Income)	
596	Cancoro (2019) (ID:87857490)	"I didn't think this service was for people like us": improving service response to BME survivors of sexual violence	-INCLUDE on title & abstract	-EXCLUDE on target group (no housing precarity)
597	Canfield (2022) (ID:87848162)	Examining sources of Social Support and Depression Prevention Among Pregnant Youth Experiencing Homelessness: Outcomes of a Seven-City Study.	-INCLUDE on title & abstract	-EXCLUDE on intervention (service engagement/helpseeking behaviour)
598	Canning (2020) (ID:87857815)	Corrosive Control: State-Corporate and Gendered Harm in Bordered Britain	-EXCLUDE on intervention (service engagement/helpseeking behaviour)	
599	Cantor (2020) (ID:87848329)	Medicaid Utilization and Spending among Homeless Adults in New Jersey: Implications for Medicaid-Funded Tenancy Support Services.	-EXCLUDE on population (women)	
600	Capous-Desyllas (2010) (ID:87857662)	Visions & voices: An arts-based qualitative study using photovoice to understand the needs and aspirations of diverse women working in the sex industry	-EXCLUDE on intervention (service engagement/helpseeking behaviour)	
601	Capous-Desyllas (2020) (ID:87963236)	Navigating intersecting identities, self-representation, and relationships: A qualitative study with trans sex workers living and working in Los Angeles, CA	-INCLUDE on title & abstract	-EXCLUDE on target group (no housing precarity)
602	Carbado (2019) (ID:87963324)	INTERSECTIONALITY AT 30	-EXCLUDE on evidence and form (evidence not in written form or presented as research output)	
603	Carbonaro (2013) (ID:87852025)	Gifford Foundation announces $147,000 in grants	-EXCLUDE on evidence and form (evidence not in written form or presented as research output)	

604	Carbonell (2021) (ID:87850541)	Reframing shelter self-recovery : women's experiences of post disaster reconstruction in Machhegaun, Nepal, after the 2015 earthquake	-EXCLUDE on country (High-Income)	
605	CARDOL (2006) (ID:87857268)	Why does it run in families?: explaining family similarity in help-seeking behaviour by shared circumstances, socialisation and selection	-EXCLUDE on population (no housing precarity)	
606	Cardona-Arias (2020) (ID:87848261)	Prevalence of hepatitis B/C viruses and associated factors in key groups attending a health services institution in Colombia, 2019.	-EXCLUDE on country (High-Income)	
607	**CARDWELL (2018) (ID:87857075)**	**Making Better Births a reality for women with multiple disadvantages: a qualitative peer research study exploring perinatal women's experiences of care and services in north-east London**	**-INCLUDE on title & abstract**	**-INCLUDE on full study**
608	Carnie (2011) (ID:87849170)	In their own words: young people's mental health in drought-affected rural and remote NSW.	-EXCLUDE on intervention (service engagement/helpseeking behaviour)	
609	CAROLE (2018) (ID:87857390)	Safeguarding pressures phase 6: research report	-EXCLUDE on population (people aged under 18 years)	
610	CAROLE (2021) (ID:87857367)	Safeguarding pressures phase 7: research report	-EXCLUDE on population (people aged under 18 years)	
611	Caroppo (2014) (ID:87848944)	Health care for immigrant women in Italy: are we really ready? A survey on knowledge about female genital mutilation.	-EXCLUDE on population (no housing precarity)	
612	Carpenter-Song (2012) (ID:87849088)	"Right here is an oasis": how "recovery communities" contribute to recovery for people with serious mental illnesses.	-EXCLUDE on population (women)	
613	Carr (2015) (ID:87963070)	Women and the experience of serious mental illness and sexual objectification: Multicultural feminist theoretical frameworks and therapy recommendations	-EXCLUDE on population (no housing precarity)	
614	Carrington (2015) (ID:87851777)	Perinatal Health Education Intervention for Adolescent and Young Adult Pregnant Homeless Women Living in Transitional Housing, and Best Practice Teaching Intervention Program for Staff Responsible for Care of the Mother-Baby Dyad.	-INCLUDE on title & abstract	-EXCLUDE on target group (not focused on women's behaviour/outcomes for women)
615	Carrington (2017) (ID:87850885)	Perinatal health education intervention for adolescent and young adult pregnant homeless women living in transitional housing, and best practice teaching intervention program for staff responsible for care of the mother-baby dyad.	-EXCLUDE Duplicate	
616	Carrington (2021) (ID:87963128)	The impact of COVID-19 pandemic on Australian domestic and family violence services and their clients	-EXCLUDE on intervention (service engagement/helpseeking behaviour)	
617	Carroll (2018) (ID:87858051)	Cultural Humility and Transgender Clients: A Study Examining the Relationship between Critical Reflection and Attitudes of Nurse Practitioners	-EXCLUDE on population (no housing precarity)	
618	Carter (2012) (ID:87851263)	Nurse-midwives in federally funded health centers: Understanding federal program requirements and benefits.	-EXCLUDE on intervention (service engagement/helpseeking behaviour)	
619	Carter (2017) (ID:87850901)	The impact of psychosocial determinants on the nonmedical use of prescription medication among women with history of intimate partner violence.	-EXCLUDE on population (no housing precarity)	
620	Carter (2019) (ID:87848393)	Housing Instability Characteristics Among Transgender Veterans Cared for in the Veterans Health Administration, 2013-2016.	-EXCLUDE on population (no gender focus; women population <50)	
621	Carter (2019) (ID:87848415)	Low barrier buprenorphine treatment for persons experiencing homelessness and injecting heroin in San Francisco.	-EXCLUDE on population (women)	
622	Carton (2010) (ID:87849287)	Changes in sources and perceived quality of social supports among formerly homeless persons receiving assertive community treatment services.	-EXCLUDE on population (no gender focus; women population <50)	
623	Carvalho (2019) (ID:87850756)	Gender differences regarding opinions on long-term care arrangements: A study of community-dwelling older adults.	-EXCLUDE on intervention (service engagement/helpseeking behaviour)	
624	Carver (2020) (ID:87849351)	What constitutes effective problematic substance use treatment from the perspective of people who are homeless? A systematic review and meta-ethnography	-EXCLUDE on population (no gender focus; women population <50)<br>-EXCLUDE - but review for literature	
625	Cascadia Behavioral... (2020) (ID:87851976)	Cascadia Behavioral Healthcare, Inc.: Self-Care Recovery Tips During COVID-19	-EXCLUDE on evidence and form (evidence not in written form or presented as research output)	

626	Cascalheira (2023) (ID:87852877)	High-risk polysubstance use among LGBTQ plus people who use drugs in the United States: An application of syndemic theory	-EXCLUDE on intervention (service engagement/helpseeking behaviour)	
627	Case (2013) (ID:87849026)	Examining DNA fingerprinting as an epidemiology tool in the tuberculosis program in the Northwest Territories, Canada.	-EXCLUDE on intervention (service engagement/helpseeking behaviour)	
628	Caso (2020) (ID:87858121)	Deconstructing Cis-Heteronormativity and Redefining Atypical Gender Trajectories in the Cultural Borderlands: Experiences of Transgender and Gender-Expansive People	-EXCLUDE on population (no housing precarity)	
629	Castán (2018) (ID:87963142)	Intersectionality challenges for the co-production of urban services: notes for a theoretical and methodological agenda	-EXCLUDE - but review for literature<br>-EXCLUDE on population (no housing precarity)	
630	Castaneda-Guarderas (2016) (ID:87852673)	Shared Decision Making With Vulnerable Populations in the Emergency Department	-EXCLUDE on intervention (service engagement/helpseeking behaviour)	
631	Castellanos (2016) (ID:87848790)	The Role of Institutional Placement, Family Conflict, and Homosexuality in Homelessness Pathways Among Latino LGBT Youth in New York City.	-EXCLUDE on population (women)	
632	Castillo (2018) (ID:87848581)	Improving Depression Care for Adults With Serious Mental Illness in Underresourced Areas: Community Coalitions Versus Technical Support.	-EXCLUDE on population (women)	
633	Cavanagh (2013) (ID:87963033)	Social work and domestic violence: Developing critical and reflective practice	-EXCLUDE on population (women)	
634	Cederbaum (2013) (ID:87849041)	The HIV risk reduction needs of homeless women in Los Angeles.	-INCLUDE on title & abstract	-EXCLUDE on intervention (service engagement/helpseeking behaviour)
635	Cegolon (2020) (ID:87853426)	Birth control knowledge among freshmen of four Italian universities	-EXCLUDE on population (women)	
636	Cerezo (2014) (ID:87857775)	Trans Migrations: Exploring Life at the Intersection of Transgender Identity and Immigration	-EXCLUDE on intervention (service engagement/helpseeking behaviour)	
637	Cerimele (2014) (ID:87848942)	Bipolar disorder in primary care: clinical characteristics of 740 primary care patients with bipolar disorder.	-EXCLUDE on population (women)	
638	Cha (2013) (ID:87849322)	Health of the homeless	-EXCLUDE on population (women)	
639	Chahua (2014) (ID:87852802)	Depression in young regular cocaine users recruited in the community	-EXCLUDE on population (women)	
640	Chakrapani (2014) (ID:87848971)	Barriers to antiretroviral treatment access for injecting drug users living with HIV in Chennai, South India.	-EXCLUDE on country (High-Income)	
641	Chakrapani (2022) (ID:87852915)	Mental health, economic well-being and health care access amid the COVID-19 pandemic: a mixed methods study among urban men who have sex with men in India	-EXCLUDE on country (High-Income)	
642	Chamberlain (2014) (ID:87963237)	Homelessness in Australia	-EXCLUDE on population (women)	
643	Chambers (2013) (ID:87849004)	High utilizers of emergency health services in a population-based cohort of homeless adults.	-EXCLUDE on population (women)	
644	Chambers (2014) (ID:87848991)	Factors associated with poor mental health status among homeless women with and without dependent children.	-INCLUDE on title & abstract	-EXCLUDE on intervention (service engagement/helpseeking behaviour)
645	CHAN (2010) (ID:87857132)	Gender roles and help-seeking behaviour: promoting professional help among Japanese men	-EXCLUDE on population (women)	
646	CHAN (2011) (ID:87857168)	Perceptions of Chinese Australians on seeking help for mental health problems: a qualitative study	-EXCLUDE on population (women)	
647	Chan (2014) (ID:87848903)	Accessibility patterns and community integration among previously homeless adults: a Geographic Information Systems (GIS) approach.	-EXCLUDE on population (women)<br>-EXCLUDE - but review for literature	
648	Chan (2018) (ID:87848487)	The SUMMIT ambulatory-ICU primary care model for medically and socially complex patients in an urban federally qualified health center: study design and rationale.	-EXCLUDE on population (women)	
649	Chan (2019) (ID:87853105)	Socio-economic inequalities in life expectancy of older adults with and without multimorbidity: a record linkage study of 1.1 million people in England	-EXCLUDE on intervention (service engagement/helpseeking behaviour)	
650	Chan (2021) (ID:87851545)	A systematic review: Sexual well-being and perceived barriers to seeking professional help among Chinese adults living with cancer.	-EXCLUDE on population (no housing precarity)	
651	Chaney (2017) (ID:87851938)	Are Homeless Women with Children Accessing Available Resources: If Not, Why?	-INCLUDE on title & abstract	-EXCLUDE on intervention (service engagement/helpseeking behaviour)

652	CHANG (2007) (ID:87857137)	Psychological distress and help-seeking among Taiwanese college students: role of gender and student status	-EXCLUDE on date (2010)	
653	Chang (2014) (ID:87851062)	Predictors of frequent emergency department use among patients with psychiatric illness.	-EXCLUDE on population (women)<br>-EXCLUDE - but review for literature	
654	Chang (2015) (ID:87851016)	Comorbid depression and substance abuse among safety-net clients in Los Angeles: A community participatory study.	-EXCLUDE on population (women)	
655	Chang (2017) (ID:87849379)	The Role of Community Health Centers in Addressing Human Trafficking	-INCLUDE on title & abstract	-EXCLUDE on target group (not focused on women's behaviour/outcomes for women)
656	Chang (2020) (ID:87848291)	Use of General Primary Care, Specialized Primary Care, and Other Veterans Affairs Services Among High-Risk Veterans.	-EXCLUDE on population (no housing precarity)	
657	Chang (2020) (ID:87857533)	Second-Order Devolution Revolution and the Hidden Structural Discrimination? Examining County Welfare-to-Work Service Systems in California	-EXCLUDE on population (no housing precarity)	
658	Chao (2019) (ID:87851765)	Incidence, Risk Factors, and Mortality Associated With Second Malignant Neoplasms Among Survivors of Adolescent and Young Adult Cancer.	-EXCLUDE on population (no housing precarity)	
659	Charani (2021) (ID:87963100)	Navigating sociocultural disparities in relation to infection and antibiotic resistance—the need for an intersectional approach	-EXCLUDE on population (no housing precarity)	
660	Charkhchi (2018) (ID:87848571)	Housing and Food Insecurity, Care Access, and Health Status Among the Chronically Ill: An Analysis of the Behavioral Risk Factor Surveillance System.	-EXCLUDE on population (women)	
661	Chartier (2012) (ID:87851238)	Specific psychiatric correlates of acute care utilization among unstably housed HIV-positive adults.	-EXCLUDE on population (women)	
662	CHASE (2008) (ID:87857097)	Well-being of asylum seeking children	-EXCLUDE on date (2010)	
663	Chassman (2022) (ID:87852973)	Prevalence and Correlates of Illicit Substance Use Among Young Adults Experiencing Homelessness in Seven Cities Across the United States	-EXCLUDE on intervention (service engagement/helpseeking behaviour)	
664	Chattopadhyay (2018) (ID:87853425)	'Safe', yet violent? Women's experiences with obstetric violence during hospital births in rural Northeast India	-EXCLUDE on country (High-Income)	
665	Chattopadhyay (2019) (ID:87850777)	The responses of health systems to marital sexual violence-A perspective from Southern India.	-EXCLUDE on country (High-Income)	
666	Chattopadhyay (2022) (ID:87857468)	Lives in Waiting	-EXCLUDE on population (no housing precarity)	
667	Chaudhry (2011) (ID:87853308)	Participant Characteristics and HIV Risk Behaviors Among Individuals Entering Integrated Buprenorphine/Naloxone and HIV Care	-EXCLUDE on population (no gender focus; women population <50)	
668	Chavez (2023) (ID:87857997)	Reflections on Pre-Kindergarten Pedagogical Practices for Latine Preschoolers: An Autoethnography	-EXCLUDE on intervention (service engagement/helpseeking behaviour)	
669	Chen (2012) (ID:87849111)	Service quality and corporate social responsibility, influence on post-purchase intentions of sheltered employment institutions.	-EXCLUDE on population (no housing precarity)	
670	Chen (2012) (ID:87849147)	A working relationship model that reduces homelessness among people with mental illness.	-EXCLUDE on population (women)	
671	Chen (2012) (ID:87853384)	Prevalence of Malnutrition among Institutionalized Elderly People in Northern Peninsular Malaysia: Gender, Ethnicity and Age-specific	-EXCLUDE on country (High-Income)	
672	Chen (2013) (ID:87851140)	Adherence to HIV treatment and care among previously homeless jail detainees.	-EXCLUDE on population (women)	
673	Chen (2014) (ID:87848984)	Developing community support for homeless people with mental illness in transition.	-EXCLUDE on population (women)<br>-EXCLUDE - but review for literature	
674	CHEN (2014) (ID:87857177)	Associations among the number of mental health problems, stigma, and seeking help from psychological services: a path analysis model among Chinese adolescents	-EXCLUDE on country (High-Income)	
675	Chen (2015) (ID:87848839)	Missing link in community psychiatry: When a patient with schizophrenia was expelled from her home.	-INCLUDE on title & abstract	-EXCLUDE on intervention (intersectionality)
676	Chen (2017) (ID:87850892)	A qualitative analysis of transgender veterans' lived experiences.	-EXCLUDE on population (no housing precarity)	
677	Chen (2018) (ID:87852878)	Psychiatric disorders among children of parents with cancer: A Swedish register-based matched cohort study	-EXCLUDE on population (people aged under 18 years)	

678	Chen (2020) (ID:87963306)	Balancing complex social and technical aspects of design: Exposing engineering students to homelessness issues	-EXCLUDE on intervention (service engagement/helpseeking behaviour)		
679	Chen (2022) (ID:87852831)	Social support and quality of life among chronically homeless patients with schizophrenia	-EXCLUDE on country (High-Income)		
680	Chen (2022) (ID:87963261)	Trauma-informed computing: Towards safer technology experiences for all	-EXCLUDE on population (no housing precarity)		
681	CHENG (2009) (ID:87857309)	Factors related to adolescents' seeking help from social workers in mental health settings	-EXCLUDE - but review for literature<br>-EXCLUDE on date (2010)		
682	CHENG (2015) (ID:87857213)	Racial disparities in intimate partner violence and in seeking help with mental health	-INCLUDE on title & abstract	-EXCLUDE on target group (no housing precarity)	
683	Cherner (2017) (ID:87848650)	Housing First for Adults with Problematic Substance Use.	-EXCLUDE on population (women)		
684	Cherner (2018) (ID:87853118)	An investigation of predictors of mental health in single men and women experiencing homelessness in three Canadian cities	-EXCLUDE on population (no gender focus; women population <50)<br>-EXCLUDE - but review for literature		
685	Cherry (2022) (ID:87857513)	More to come: Perspectives and lived experiences of adults aging with HIV	-EXCLUDE on population (no housing precarity)		
686	Chesney (2021) (ID:87853103)	Immediate and Long-Term Health Care Support Needs of Older Adults Undergoing Cancer Surgery: A Population-Based Analysis of Postoperative Homecare Utilization	-EXCLUDE on population (no housing precarity)		
687	CHEUNG (2011) (ID:87857261)	Depressive symptoms and help-seeking behaviors among Korean Americans	-EXCLUDE on population (no housing precarity)		
688	Cheung (2020) (ID:87848274)	The impact of the Syrian conflict on population well-being.	-EXCLUDE on country (High-Income)		
689	Chew (2013) (ID:87851174)	Impact of social network characteristics on shelter use among street youth in San Francisco.	-EXCLUDE on population (women)		
690	Chhetri (2023) (ID:87853362)	The Body in Crisis: A Health Needs Assessment among Female Survivors of Interpersonal Violence	-INCLUDE on title & abstract	-EXCLUDE on intervention (intersectionality)	
691	Chiaramonte (2020) (ID:87857844)	Gendered Power and Healthcare Access: A Participatory Study with Transgender and Gender Diverse Youth	-EXCLUDE on population (no housing precarity)		
692	Chiaramonte (2022) (ID:87850614)	Examining contextual influences on the service needs of homeless and unstably housed domestic violence survivors.	-INCLUDE on title & abstract	-INCLUDE on full study	-EXCLUDED (Quality Appraisal)
693	Chiba (2010) (ID:87849286)	Reliability and validity of the Japanese version of the Recovery Assessment Scale (RAS) for people with chronic mental illness: scale development.	-EXCLUDE on population (no housing precarity)		
694	Child (2023) (ID:87851865)	Race, homelessness and inner-city policy in 1980s Britain	-EXCLUDE on population (women)		
695	CHILDREN'S (2010) (ID:87857262)	Destitution amongst asylum-seeking and refugee children	-EXCLUDE on population (women)		
696	CHILDREN'S (2019) (ID:87857060)	Child first: a guide to being looked after for unaccompanied asylum seeking children	-EXCLUDE on intervention (service engagement/helpseeking behaviour)		
697	Childress (2022) (ID:87852772)	Barriers to Help-Seeking for Domestic Violence in Kyrgyzstan: Perspectives of Criminal Justice, Social, Health, and Educational Professionals	-EXCLUDE on country (High-Income)		
698	Chinchilla (2019) (ID:87848380)	Comparing Tenant and Neighborhood Characteristics of the VA's Project- vs. Tenant-Based Supportive Housing Program in Los Angeles County.	-EXCLUDE on population (women)		
699	Chinchilla (2020) (ID:87853122)	Exploring Community Integration Among Formerly Homeless Veterans in Project-Based Versus Tenant-Based Supportive Housing	-EXCLUDE on population (women)		
700	Chinman (2001) (ID:87853706)	Chronicity reconsidered: Improving person-environment fit through a consumer-run service	-EXCLUDE on population (no housing precarity)		
701	CHIU (2009) (ID:87857281)	Mental distress and internal stigma in seeking professional help among women in Tin Shui Wai (TSW), Hong Kong	-EXCLUDE on date (2010)		
702	Chiu (2010) (ID:87853328)	A Pilot Study Comparing Two Developmental Screening Tools for Use With Homeless Children	-EXCLUDE on population (people aged under 18 years)		
703	Chiu (2013) (ID:87851186)	Childhood obesity and dental caries in homeless children.	-EXCLUDE on population (people aged under 18 years)		
704	Cho (2013) (ID:87963181)	Toward a field of intersectionality studies: Theory, applications, and praxis	-EXCLUDE on intervention (service engagement/helpseeking behaviour)		
705	Cho (2019) (ID:87853064)	The association of female sex with application of evidence-based practice recommendations for perioperative care in hip fracture surgery	-EXCLUDE on population (no housing precarity)		
706	CHO (2020) (ID:87857069)	Patterns of intimate partner violence victimization and survivors' help-seeking	-INCLUDE on title & abstract	-EXCLUDE (IPV/DVA but little discussion on housing)	

707	Choak (2022) (ID:87857770)	Alternative post-16 transitions: examining the career pathways of young women 'on road'	-EXCLUDE on intervention (service engagement/helpseeking behaviour)	
708	Choi (2021) (ID:87851886)	Life during lockdown: a qualitative study of low-income New Zealanders' experience during the COVID-19 pandemic	-EXCLUDE on population (no housing precarity)	
709	Choi (2023) (ID:87857766)	Understanding the complexity of domestic violence service delivery through the lived experiences of domestic violence advocates	-EXCLUDE on population (women)	
710	Chondraki (2014) (ID:87848978)	Homeless mentally ill in Athens area: a cross-sectional study on unmet needs and help-seeking.	-EXCLUDE on population (women)	
711	Choo (2010) (ID:87963161)	Practicing intersectionality in sociological research: A critical analysis of inclusions, interactions, and institutions in the study of inequalities	-EXCLUDE on intervention (service engagement/helpseeking behaviour)<br>-EXCLUDE - but review for literature	
712	CHOUDHURY (2009) (ID:87857393)	Community alienation and its impact on help-seeking behavior among LGBTIQ South Asians in Southern California	-EXCLUDE on date (2010)<br>-EXCLUDE - but review for literature	
713	Choudhury (2011) (ID:87849207)	Maternal care practices among the ultra poor households in rural Bangladesh: a qualitative exploratory study.	-EXCLUDE on country (High-Income)	
714	Chowbey (2016) (ID:87857505)	Employment, masculinities, and domestic violence in 'fragile' contexts: Pakistani women in Pakistan and the UK	-INCLUDE on title & abstract	-EXCLUDE on target group (no housing precarity)
715	Chowdhury (2018) (ID:87852678)	Household Food Security and Birth Size of Infants: Analysis of the Bangladesh Demographic and Health Survey 2011	-EXCLUDE on country (High-Income)	
716	CHRISTIAN (2010) (ID:87856983)	The role of efficacy and well-being in guiding the use of education and training services by young homeless mothers	-INCLUDE on title & abstract	-EXCLUDE on intervention (service engagement/helpseeking behaviour)
717	Chrystal (2015) (ID:87848862)	Experience of primary care among homeless individuals with mental health conditions.	-EXCLUDE on population (no gender focus; women population <50)	
718	Chum (2016) (ID:87848765)	Construct Validity of the SF-12v2 for the Homeless Population with Mental Illness: An Instrument to Measure Self-Reported Mental and Physical Health.	-EXCLUDE on population (women)	
719	Chun (2016) (ID:87851676)	Organizing across divides: Union challenges to precarious work in Vancouver's privatized health care sector.	-EXCLUDE on population (no housing precarity)	
720	Chung (2011) (ID:87851327)	Psychiatric pharmacist and primary care collaboration at a skid-row safety-net clinic.	-EXCLUDE on population (women)	
721	CHUNG (2013) (ID:87851731)	RESILIENCE AMONG SINGLE ADULT FEMALE REFUGEES IN HAMILTON, ONTARIO.	-INCLUDE on title & abstract	-EXCLUDE on target group (no housing precarity)
722	Chung (2018) (ID:87963148)	Young women from African backgrounds and sexual violence	-INCLUDE on title & abstract	-EXCLUDE (IPV/DVA but little discussion on housing)
723	Chwastiak (2012) (ID:87849092)	Impact of health insurance status and a diagnosis of serious mental illness on whether chronically homeless individuals engage in primary care.	-EXCLUDE on population (no gender focus; women population <50)	
724	Chynoweth (2020) (ID:87851907)	A social ecological approach to understanding service utilization barriers among male survivors of sexual violence in three refugee settings: a qualitative exploratory study	-EXCLUDE on population (women)	
725	CIARROCHI (2002) (ID:87857062)	Adolescents who need help the most are the least likely to seek it: the relationship between low emotional competence and low intention to seek help	-EXCLUDE on date (2010)	
726	Cicero (2018) (ID:87857866)	The Health Status of Transgender and Gender Nonconforming Adults	-EXCLUDE on population (no housing precarity)	
727	Cigrang (2020) (ID:87963140)	Brief motivational interview–based intervention for women in jail with history of drug addiction and sex-trading.	-EXCLUDE on intervention (service engagement/helpseeking behaviour)	
728	Cimino (2017) (ID:87963365)	Childhood maltreatment and child protective services involvement among the commercially sexually exploited: A comparison of women who enter as juveniles or as adults	-EXCLUDE on intervention (service engagement/helpseeking behaviour)<br>-EXCLUDE - but review for literature	
729	City of Maysville Kentucky:... (2019) (ID:87851987)	City of Maysville Kentucky: Commission on Human Rights to Meet Thursday, Sept. 19	-EXCLUDE on evidence and form (evidence not in written form or presented as research output)	
730	Claeys (2010) (ID:87851376)	Beyond despair -- sexual and reproductive health care in Haiti after the earthquake.	-EXCLUDE on country (High-Income)	
731	Clark (2013) (ID:87849032)	Healthcare utilization in medical intensive care unit survivors with alcohol withdrawal.	-EXCLUDE on population (no housing precarity)	
732	Clark (2013) (ID:87851169)	Transitioning through family homelessness and the effect of substance abuse on social support systems.	-INCLUDE on title & abstract	-EXCLUDE on evidence and form (evidence not in written form or presented as research output)
733	Clark (2018) (ID:87848540)	Health Care Utilization and Expenditures of Homeless Family Members Before and After Emergency Housing.	-EXCLUDE on population (no gender focus; women population <50)	

734	Clark (2019) (ID:87848460)	Homelessness Contributes To Pregnancy Complications.	-INCLUDE on title & abstract	-EXCLUDE on intervention (service engagement/helpseeking behaviour)
735	Clark (2019) (ID:87853028)	Infants Exposed To Homelessness: Health, Health Care Use, And Health Spending From Birth To Age Six	-EXCLUDE on population (people aged under 18 years)	
736	CLARKE (2008) (ID:87857323)	Living on the edge of despair: destitution amongst asylum seeking and refugee children	-EXCLUDE on date (2010)<br>-EXCLUDE - but review for literature	
737	Clary (2023) (ID:87963328)	Masculinity and stigma among emerging adult military members and veterans: implications for encouraging help-seeking	-EXCLUDE on population (women)	
738	Claver (2013) (ID:87849077)	Comprehensive care for vulnerable elderly veterans during disasters.	-EXCLUDE on population (women)	
739	Clay (2018) (ID:87848589)	Application of a Theoretical Model Toward Understanding Continued Food Insecurity Post Hurricane Katrina.	-EXCLUDE on intervention (service engagement/helpseeking behaviour)	
740	CLEARY (2017) (ID:87857222)	Help-seeking patterns and attitudes to treatment amongst men who attempted suicide	-EXCLUDE on population (women)	
741	CLEMENT (2018) (ID:87856952)	Couples First? Understanding the needs of rough sleeping couples	-INCLUDE on title & abstract	-EXCLUDE on intervention (intersectionality)
742	Clemenzi-Allen (2019) (ID:87848367)	Evaluating the Impact of Housing Status on Gonorrhea and Chlamydia Screening in an HIV Primary Care Setting.	-EXCLUDE on population (women)	
743	CLOUGH (2014) (ID:87857024)	'Having housing made everything else possible': affordable, safe and stable housing for women survivors of violence	-INCLUDE on title & abstract	-EXCLUDE on intervention (intersectionality)
744	Cloutier (2016) (ID:87848751)	The Economic Burden of Schizophrenia in the United States in 2013.	-EXCLUDE on population (women)	
745	Cocker (2019) (ID:87857972)	Positioning discourse on homophobia in schools: What have lesbian and gay families got to say?	-EXCLUDE on population (no housing precarity)	
746	Cocks (2011) (ID:87849230)	A quality framework for personalised residential supports for adults with developmental disabilities.	-EXCLUDE on population (no housing precarity)	
747	COE (2009) (ID:87857150)	Exploring attitudes of the general public to stress, depression and help seeking	-EXCLUDE on date (2010)	
748	Coe (2015) (ID:87848895)	Medication adherence challenges among patients experiencing homelessness in a behavioral health clinic.	-EXCLUDE on population (no gender focus; women population <50)	
749	Cofield (2022) (ID:87857785)	"You Good, Bruh?" An Exploration of the Influence of Race and Masculinity on Millennial Black Men's Decisions to Seek Mental Health Treatment	-EXCLUDE on population (women)	
750	Cohen (1989) (ID:87853715)	Interaction and Mutual Influence in a Program for Homeless Mentally Ill Women	-EXCLUDE on date (2010)	
751	COHEN (1999) (ID:87857074)	Measuring the willingness to seek help	-EXCLUDE on date (2010)	
752	Cohen (2022) (ID:87853013)	Risk factors for Veteran food insecurity: findings from a National US Department of Veterans Affairs Food Insecurity Screener	-EXCLUDE on population (no housing precarity)	
753	Coker (2010) (ID:87851401)	The health and health care of lesbian, gay, and bisexual adolescents.	-EXCLUDE on intervention (service engagement/helpseeking behaviour)	
754	Cole (2010) (ID:87857604)	Making Coalitions Work: Solidarity across Difference within US Feminism	-EXCLUDE on population (no housing precarity)	
755	Colell (2014) (ID:87852868)	Prevalence of the use of hypnotics and sedatives among the working population and associated work-related stress factors	-EXCLUDE on population (no housing precarity)	
756	Colell (2014) (ID:87853404)	Work-related stress factors associated with problem drinking: A study of the Spanish working population	-EXCLUDE on population (no housing precarity)	
757	Collerton (2014) (ID:87848902)	An exploratory study of the effectiveness of memory AIDS for older people living in supported accommodation.	-EXCLUDE on population (no housing precarity)	
758	Collier (2016) (ID:87857908)	Negotiating Contextually Contingent Agency: Situated Feminist Peacebuilding Strategies in Kenya	-EXCLUDE on country (High-Income)	
759	Collins (2017) (ID:87857426)	On violence, intersectionality and transversal politics	-EXCLUDE on population (no housing precarity)	
760	Collins (2018) (ID:87850801)	Post-hoc analysis of two clinical trials examining Customized Adherence Enhancement plus long acting injectable antipsychotic (CAE-L) in high-risk individuals with serious mental illness.	-EXCLUDE on population (no housing precarity)	

761	Collins (2019) (ID:87848430)	Harm reduction treatment for smoking (HaRT-S): findings from a single-arm pilot study with smokers experiencing chronic homelessness.	-EXCLUDE on population (women)	
762	Collins (2019) (ID:87963071)	The intersectional risk environment of people who use drugs	-EXCLUDE on population (no gender focus; women population <50)<br>-EXCLUDE - but review for literature	
763	Collins (2020) (ID:87851977)	Evaluation of Los Angeles County's 2019 "Spark the True You" Mental Health Campaign	-INCLUDE on title & abstract	-EXCLUDE on intervention (intersectionality)
764	Collins (2020) (ID:87963287)	(Re) shaping the self: an ethnographic study of the embodied and spatial practices of women who use drugs	-INCLUDE on title & abstract	-EXCLUDE on intervention (service engagement/helpseeking behaviour)
765	Collins (2021) (ID:87848205)	Combining behavioral harm-reduction treatment and extended-release naltrexone for people experiencing homelessness and alcohol use disorder in the USA: a randomised clinical trial.	-EXCLUDE on population (women)	
766	Collins-Anderson (2022) (ID:87857470)	Intersectionality and Mental Health Among Emerging Adult Black American Men: a Scoping Review	-EXCLUDE on population (women)	
767	Collinson (2011) (ID:87849353)	The Invisible Men: finding and engaging with the male partners of street sex workers	-EXCLUDE on population (women)	
768	Colon-Lopez (2014) (ID:87851588)	Survival from Anal Cancer Among Hispanics--Puerto Rico, 2000--2007	-EXCLUDE on intervention (service engagement/helpseeking behaviour)	
769	Comassetto (2021) (ID:87848195)	Dental Pain in Homeless Adults in Porto Alegre, Brazil.	-EXCLUDE on country (High-Income)	
770	Comfort (2020) (ID:87857526)	Resource Wars and Resourceful Resistance: Gender Violence and Irreal Oil Environments in Two Global Novels by Women	-EXCLUDE on country (High-Income)	
771	Community calendar:... (2013) (ID:87851950)	Community calendar: Food pantry is in need of food items	-EXCLUDE on evidence and form (evidence not in written form or presented as research output)	
772	Comulada (2020) (ID:87857445)	Predictors of internet health information⇓seeking behaviors among young adults living with HIV across the United States: Longitudinal observational study	-EXCLUDE on population (no housing precarity)	
773	Conard (2017) (ID:87853066)	Deployed Women Veterans: Important Culturally Sensitive Care	-INCLUDE on title & abstract	-EXCLUDE on intervention (service engagement/helpseeking behaviour)
774	CONLON (2021) (ID:87857404)	Stuff you think you can handle as a parent and stuff you can't'. Understanding parental health-seeking behaviour when accessing unscheduled care: a qualitative study	-EXCLUDE on population (no housing precarity)	
775	Conn (2018) (ID:87857796)	"Our lives aren't over": A strengths-based perspective on stigma, discrimination, and coping among young parents	-EXCLUDE on population (no housing precarity)	
776	Conner (2018) (ID:87848593)	Woman to Woman (W2W): Adapting an HIV risk reduction intervention for older women.	-INCLUDE on title & abstract	-EXCLUDE on intervention (intersectionality)
777	CONNOLLY (2017) (ID:87857289)	Cut off from justice: the impact of excluding separated and migrant children from legal aid	-EXCLUDE on population (people aged under 18 years)	
778	Conolly (2011) (ID:87852038)	Staff and ex-service user co-working: a counselling service's enhanced response to multiple exclusion homelessness	-EXCLUDE on population (women)<br>-EXCLUDE - but review for literature	
779	Conteh (2021) (ID:87857441)	Exploring gender, health, and intersectionality in informal settlements in Freetown	-EXCLUDE on country (High-Income)	
780	Conway (2013) (ID:87851673)	Mortality by Race Among Low-Income Adults With Early-Onset Insulin-Treated Diabetes	-EXCLUDE on population (no housing precarity)	
781	Conway (2013) (ID:87963110)	Family secrets and social silence: women with insecure immigration status and domestic abuse policy in Scotland	-INCLUDE on title & abstract	-EXCLUDE (IPV/DVA but little discussion on housing)
782	Cook (2019) (ID:87857798)	Trauma Training: Competencies, Initiatives, and Resources	-EXCLUDE on population (no housing precarity)	
783	Cook (2019) (ID:87857976)	Local turns: Queer histories and Brighton's queer communities	-EXCLUDE on intervention (service engagement/helpseeking behaviour)	
784	Cooke (2016) (ID:87963292)	Information services to diverse populations: Developing culturally competent library professionals	-EXCLUDE on population (no housing precarity)	
785	Coombes (2021) (ID:87857794)	"Is there a Way Back to Me, for Me"? Older Men's Stories of Life, Loss, Connection and Community Development	-EXCLUDE on population (women)	
786	COONEY (2020) (ID:87857040)	Hosting women who have experienced trauma: What is involved? What are the benefits and challenges?	-INCLUDE on title & abstract	-EXCLUDE on intervention (intersectionality)
787	Cooney (2022) (ID:87963302)	Prevention-effective adherence trajectories among transgender women indicated for PrEP in the United States: a prospective cohort study	-EXCLUDE on intervention (service engagement/helpseeking behaviour)	

788	Cooper (2021) (ID:87857747)	Empowering the People: Saint Ambrose Episcopal Church and Twenty-First Century Mission	-EXCLUDE on population (no housing precarity)	
789	Copeland (2020) (ID:87848316)	Gender differences in newly separated veterans' use of healthcare.	-INCLUDE on title & abstract	-EXCLUDE on target group (no housing precarity)
790	Corbett (2020) (ID:87857573)	Jumbled Mosaics: Exploring Intracategorical Complexity in the Memoirs of Jewish Austrian (Youth) Emigrants to the United States	-EXCLUDE on intervention (service engagement/helpseeking behaviour)	
791	Corcorran (2021) (ID:87848229)	Age and gender-specific hepatitis C continuum of care and predictors of direct acting antiviral treatment among persons who inject drugs in Seattle, Washington.	-EXCLUDE on population (no gender focus; women population <50)	
792	CORDIS (2020) (ID:87857225)	Flexible responses during the Coronavirus crisis: rapid evidence gathering	-EXCLUDE on population (women)	
793	Coren (2013) (ID:87849014)	Interventions for promoting reintegration and reducing harmful behaviour and lifestyles in street-connected children and young people.	-EXCLUDE on population (people aged under 18 years)	
794	Coren (2016) (ID:87848780)	Interventions for promoting reintegration and reducing harmful behaviour and lifestyles in street-connected children and young people.	-EXCLUDE on country (High-Income)	
795	Cornelius (2017) (ID:87853231)	Impact of food, housing, and transportation insecurity on ART adherence: a hierarchical resources approach	-EXCLUDE on population (women)	
796	Cornes (2014) (ID:87848977)	Reconciling recovery, personalisation and Housing First: integrating practice and outcome in the field of multiple exclusion homelessness.	-EXCLUDE on intervention (service engagement/helpseeking behaviour)	
797	Corrigan (2017) (ID:87848636)	Peer navigators to promote engagement of homeless African Americans with serious mental illness in primary care.	-EXCLUDE on population (women)	
798	Corrigan (2017) (ID:87848670)	Using Peer Navigators to Address the Integrated Health Care Needs of Homeless African Americans With Serious Mental Illness.	-EXCLUDE on population (women)	
799	Cossyleon (2018) (ID:87857919)	"Hear Us, See Us!": How Mothers of Color Transform Family and Community Relationships through Grassroots Collective Action	-EXCLUDE on population (no housing precarity)	
800	Costa (2020) (ID:87848326)	Intensive Outpatient Treatment (IOP) of Behavioral Health (BH) Problems: Engagement Factors Predicting Subsequent Service Utilization.	-EXCLUDE on population (women)	
801	Costa (2021) (ID:87848230)	Prevalence and Correlates of Nonprescription Hormone Use Among Trans Women in Sao Paulo, Brazil.	-EXCLUDE on country (High-Income)	
802	Coston (2020) (ID:87852692)	Patterns of Post-Traumatic Health Care Service Need and Access among Bisexual and Non-Monosexual Women in the U.S	-INCLUDE on title & abstract	-EXCLUDE on intervention (service engagement/helpseeking behaviour)
803	Côté (2019) (ID:87849323)	Sexual Health Services for Homeless Youth: A Qualitative Analysis of their Experiences	-EXCLUDE on population (no gender focus; women population <50)	
804	Cote (2021) (ID:87853450)	"The least loved, that's what I was": A qualitative analysis of the pathways to homelessness by LGBTQ+youth	-EXCLUDE on intervention (service engagement/helpseeking behaviour)	
805	Cotignola-Pickens (2022) (ID:87849365)	Understanding service engagement among women experiencing housing instability: structural and contextual themes	-EXCLUDE on evidence and form (evidence not in written form or presented as research output)<br>-EXCLUDE - but review for literature	
806	Couch (2012) (ID:87963301)	Young people, old issues: Methodological concerns in research with highly marginalised young people	-EXCLUDE on intervention (service engagement/helpseeking behaviour)<br>-EXCLUDE - but review for literature	
807	Couch (2017) (ID:87850888)	'Neither here nor there': Refugee young people and homelessness in Australia.	-EXCLUDE on population (no gender focus; women population <50)	
808	Couldrey (2010) (ID:87850526)	Violence within the lives of homeless people	-EXCLUDE on population (no gender focus; women population <50)<br>-EXCLUDE - but review for literature	
809	Couto (2022) (ID:87853226)	Meanings of quality of life for sex workers: structure of social representations	-EXCLUDE on country (High-Income)	
810	COVID-19 Pandemia Impact… (Shteinlukht) (ID:87851601)	COVID-19 Pandemia Impact on Mental Health of Older Adults and Possible Treatment Options.	-EXCLUDE on population (no housing precarity)	
811	Covington (2017) (ID:87851937)	Lending Power: How Self-Help Credit Union Turned Small-Time Loans into Big-Time Change	-EXCLUDE on population (no housing precarity)	

812	COWEN (1999) (ID:87857083)	Community care, ideology and social policy	-EXCLUDE on date (2010)	
813	COWLING (2004) (ID:87857293)	Children of adults with severe mental illness: mental health, help seeking and service use	-EXCLUDE on date (2010)	
814	Cox (2011) (ID:87849203)	Does concurrent cocaine use compromise 1-year treatment outcomes for opiate users?.	-EXCLUDE on population (women)	
815	Cox (2017) (ID:87848641)	Characteristics and service utilization of homeless veterans entering VA substance use treatment.	-EXCLUDE on population (women)	
816	Cox (2019) (ID:87857914)	Almost Nowhere: Problematizing the Exclusivity and Coloniality of American Wilderness and Thru-Hiking	-EXCLUDE on population (no housing precarity)	
817	Cox (2022) (ID:87853372)	Risk stratification with explainable machine learning for 30-day procedure-related mortality and 30-day unplanned readmission in patients with peripheral arterial disease	-EXCLUDE on population (no housing precarity)	
818	Crabtree (2018) (ID:87850780)	Perceived harms and harm reduction strategies among people who drink non-beverage alcohol: Community-based qualitative research in Vancouver, Canada.	-EXCLUDE on population (no housing precarity)	
819	Craddock (2016) (ID:87848732)	Are Parental Relationships Always Protective? A Social Network Analysis of Black, Latino, and White Homeless Youth and Sexual Risk-Taking Behaviors.	-EXCLUDE on intervention (service engagement/helpseeking behaviour)	
820	Craddock (2020) (ID:87848353)	Individual and Social Network Correlates of Sexual Health Communication Among Youth Experiencing Homelessness.	-EXCLUDE on population (women)	
821	Craddock (2020) (ID:87852917)	Sexual Health Communication Among Young Black Women and Their Social Network Members	-EXCLUDE on population (no housing precarity)	
822	Craig (2014) (ID:87848925)	'Complex' but coping: experience of symptoms of tuberculosis and health care seeking behaviours--a qualitative interview study of urban risk groups, London, UK.	-EXCLUDE on population (no gender focus; women population <50)<br>-EXCLUDE - but review for literature	
823	Craig (2015) (ID:87852682)	Patching Holes and Integrating Community: A Strengths-Based Continuum of Care for Lesbian, Gay, Bisexual, Transgender and Questioning Youth	-EXCLUDE on population (no housing precarity)	
824	Craine (2010) (ID:87849270)	Characteristics of injecting drug users accessing different types of needle and syringe programme or using secondary distribution.	-EXCLUDE on population (women)	
825	Cramer (2002) (ID:87850513)	Engendering homelessness : an ethnographic study of homeless practices in a post-industrial city	-EXCLUDE on date (2010)	
826	CRAMER (2002) (ID:87856933)	Homelessness: what's gender got to do with it?	-EXCLUDE on date (2010)	
827	CRAMER (2010) (ID:87857350)	Over-the-counter advice seeking about complementary and alternative medicines (CAM) in community pharmacies and health shops: an ethnographic study	-EXCLUDE on population (no housing precarity)	
828	CRANE (2015) (ID:87857306)	Dual diagnosis among veterans in the United States	-EXCLUDE on intervention (service engagement/helpseeking behaviour)	
829	Crawford (2014) (ID:87848915)	Sustaining dignity? food insecurity in homeless young people in urban Australia.	-EXCLUDE on intervention (service engagement/helpseeking behaviour)	
830	Crea (2018) (ID:87857409)	Unaccompanied immigrant children in long term foster care: Identifying needs and best practices from a child welfare perspective	-EXCLUDE on population (people aged under 18 years)	
831	Crenshaw (2011) (ID:87963267)	From private violence to mass incarceration: Thinking intersectionally about women, race, and social control	-EXCLUDE on intervention (service engagement/helpseeking behaviour)<br>-EXCLUDE - but review for literature	
832	Creswell (2014) (ID:87963221)	A critical black feminist ethnography of treatment for women with co-occurring disorders in the psychiatric hospital	-INCLUDE on title & abstract	-EXCLUDE on target group (no housing precarity)
833	Cripps (2019) (ID:87963039)	Improving housing and service responses to domestic and family violence for Indigenous individuals and families	-INCLUDE on title & abstract	-EXCLUDE (IPV/DVA but little discussion on housing)
834	Crisafi (2016) (ID:87848787)	Within the Bounds: The Role of Relocation on Intimate Partner Violence Help-Seeking for Immigrant and Native Women With Histories of Homelessness.	-INCLUDE on title & abstract	-EXCLUDE on intervention (service engagement/helpseeking behaviour)
835	CRISIS (2012) (ID:87857005)	Research briefing: young, hidden and homeless	-EXCLUDE on population (no gender focus; women population <50)	
836	CRISP (2020) (ID:87857099)	West-Yorkshire Finding Independence (WY-FI): effectiveness, outcomes and impact	-EXCLUDE on evidence and form (evidence not in written form or presented as research output)<br>-EXCLUDE - but review for literature	
837	Critelli (2012) (ID:87849108)	Voices of resistance: seeking shelter services in Pakistan.	-EXCLUDE on country (High-Income)	

838	Critical ethics of care... (2019) (ID:87851414)	Critical ethics of care in social work: transforming the politics and practices of caring	-EXCLUDE on intervention (service engagement/helpseeking behaviour)	
839	Crock (2017) (ID:87853020)	Emerging Needs of People Living With HIV Receiving Community-Based Nursing in an Australian Setting	-EXCLUDE on population (no housing precarity)	
840	CROFT-WHITE (1996) (ID:87856972)	Double exposure: addressing the needs of homeless women with a mental illness	-EXCLUDE on date (2010)	
841	CRONLEY (2010) (ID:87857054)	How well does it fit? An organizational culture approach to assessing technology use among homeless service providers	-EXCLUDE on intervention (service engagement/helpseeking behaviour)	
842	CRONLEY (2012) (ID:87856988)	Does the organization matter? A multilevel analysis of organizational effects in homeless service innovations	-EXCLUDE on intervention (service engagement/helpseeking behaviour)	
843	Cronley (2013) (ID:87849023)	Do official hospitalizations predict medical vulnerability among the homeless?: a postdictive validity study of the vulnerability index.	-EXCLUDE on population (no gender focus; women population <50)	
844	CRONLEY (2015) (ID:87856992)	Effects of homelessness and child maltreatment on the likelihood of engaging in property and violent crime during adulthood	-EXCLUDE on intervention (service engagement/helpseeking behaviour)	
845	Cronley (2018) (ID:87852761)	A community of singers: employing exploratory cluster analysis to reveal profiles of psychosocial characteristics among members of a street choir for homeless and formerly homeless individuals	-EXCLUDE on intervention (service engagement/helpseeking behaviour)<br>-EXCLUDE - but review for literature	
846	Cronley (2018) (ID:87963056)	Reproductive health rights and survival: The voices of mothers experiencing homelessness	-INCLUDE on title & abstract	-EXCLUDE on intervention (service engagement/helpseeking behaviour)
847	Cronley (2020) (ID:87852850)	?There?s like no support system?: the life course stories of women with children about growing up, becoming mothers, and becoming homeless	-INCLUDE on title & abstract	-EXCLUDE on intervention (service engagement/helpseeking behaviour)
848	Cronley (2022) (ID:87857412)	Invisible intersectionality in measuring vulnerability among individuals experiencing homelessness - critically appraising the VI-SPDAT	-EXCLUDE on intervention (service engagement/helpseeking behaviour)	
849	CROOK (1999) (ID:87856962)	The new sisters of the road: homeless women and their children	-EXCLUDE on date (2010)	
850	Croom (2017) (ID:87963296)	Exploring undergraduate Black womyn's motivations for engaging in "sister circle" organizations	-EXCLUDE on population (no housing precarity)	
851	CROSBY (2018) (ID:87857221)	Factors that contribute to help-seeking among homeless, trauma-exposed youth: a social-ecological perspective	-EXCLUDE on population (no gender focus; women population <50)<br>-EXCLUDE - but review for literature	
852	Crowder (2023) (ID:87857475)	Doing More than Thanking Black Women: The Influence of Intersectional Solidarity on Public Support for Policies	-EXCLUDE on population (no housing precarity)	
853	Cuca (2014) (ID:87851060)	Women living with HIV: Social stigma and reproductive decisions.	-EXCLUDE on intervention (service engagement/helpseeking behaviour)	
854	Cuddeback (2019) (ID:87848419)	Persons With Severe Mental Illnesses and Sex Offenses: Recidivism After Prison Release.	-EXCLUDE on population (women)	
855	Culbreth (2023) (ID:87853067)	Stressors Associated with Tobacco Use Among Trans Women	-EXCLUDE on intervention (service engagement/helpseeking behaviour)	
856	Cullen (2017) (ID:87857687)	Gendered Mobilizations against Austerity in Ireland	-EXCLUDE on population (no housing precarity)	
857	Cullen (2021) (ID:87857813)	Responses to the COVID-19 crisis in Ireland: From feminized to feminist	-EXCLUDE on population (no housing precarity)	
858	Cullen (2022) (ID:87963076)	Integrating trauma and violence informed care in primary health care settings for First Nations women experiencing violence: a systematic review	-INCLUDE on title & abstract	-EXCLUDE (IPV/DVA but little discussion on housing)
859	Cultural... (2016) (ID:87858041)	Cultural Anthropology	-EXCLUDE on intervention (service engagement/helpseeking behaviour)	
860	Cultural... (2018) (ID:87858028)	Cultural Anthropology	-EXCLUDE on intervention (service engagement/helpseeking behaviour)	
861	Cummings (2018) (ID:87852002)	A Focused Ethnography of Healthcare Transition among Persistent Asthmatic/Chronic Obstructive Pulmonary Disease (COPD) Adult Pennsylvania Medicaid Beneficiaries	-EXCLUDE on population (women)	
862	Currie (2014) (ID:87848904)	Examining the relationship between health-related need and the receipt of care by participants experiencing homelessness and mental illness.	-EXCLUDE on population (women)	

863	Curry (2017) (ID:87853089)	Motor Vehicle Crash Risk Among Adolescents and Young Adults With Attention-Deficit/Hyperactivity Disorder	-EXCLUDE on population (no housing precarity)
864	CURTIS (2010) (ID:87857154)	Youth perceptions of suicide and help-seeking: 'they'd think I was weak or "mental"'	-EXCLUDE on population (no housing precarity)
865	Curtis-Boles (2021) (ID:87963020)	Living in the margins: Intersecting identities and clinical work with black women	-EXCLUDE on intervention (service engagement/helpseeking behaviour)
866	Cusack (2023) (ID:87851869)	Addressing Concurrent Housing Instability, Criminal Justice Involvement, and Unemployment Among Veteran Health Administration Service Users	-EXCLUDE on population (no gender focus; women population <50)
867	Cush (2020) (ID:87848290)	Positive health among older Traveller and older homeless adults: A scoping review of life-course and structural determinants.	-EXCLUDE on population (women)<br>-EXCLUDE - but review for literature
868	Cuthill (2016) (ID:87857877)	Political representation for social justice in nursing: lessons learned from participant research with destitute asylum seekers in the UK	-EXCLUDE on intervention (service engagement/helpseeking behaviour)
869	Cutts (2018) (ID:87853026)	Homelessness During Infancy: Associations With Infant and Maternal Health and Hardship Outcomes	-EXCLUDE on intervention (service engagement/helpseeking behaviour)
870	Cypel (2020) (ID:87848252)	Food Insecurity in US Military Veterans.	-EXCLUDE on population (women)
871	Czarnecki (2017) (ID:87857683)	Virginia Woolf and the Natural World	-EXCLUDE on evidence and form (evidence not in written form or presented as research output)
872	Czechowski (2022) (ID:87853363)	Sexual and Romantic Relationships Among People Experiencing Homelessness: A Scoping Review	-EXCLUDE on intervention (service engagement/helpseeking behaviour)
873	Czeisler (2021) (ID:87857791)	Mental Health During the COVID-19 Pandemic: Challenges, Populations at Risk, Implications, and Opportunities	-EXCLUDE on population (no housing precarity)
874	D'Agostino (2023) (ID:87852919)	Sex- and Gender-Related Aspects in Pulmonary Hypertension	-EXCLUDE on population (no housing precarity)
875	D'Amico (2017) (ID:87848628)	Group motivational interviewing for homeless young adults: Associations of change talk with substance use and sexual risk behavior.	-EXCLUDE on population (women)
876	D'Ath (2016) (ID:87848771)	East London's Homeless: a retrospective review of an eye clinic for homeless people.	-EXCLUDE on intervention (service engagement/helpseeking behaviour)
877	da Silva (2021) (ID:87857788)	Covid-19 vaccine acceptance among an online sample of sexual and gender minority men and transgender women	-EXCLUDE on population (no housing precarity)
878	Dahlburg (2009) (ID:87853688)	Authoritative Guide to Self-Help Resources in Mental Health	-EXCLUDE on date (2010)
879	Dahlman (2017) (ID:87848635)	Physical pain is common and associated with nonmedical prescription opioid use among people who inject drugs.	-EXCLUDE on intervention (service engagement/helpseeking behaviour)
880	Dahya (2019) (ID:87852887)	Social support networks, instant messaging, and gender equity in refugee education	-EXCLUDE on country (High-Income)
881	Dal (2014) (ID:87851670)	Long-term survival, prevalence, and cure of cancer: a population-based estimation for 818 902 Italian patients and 26 cancer types.	-EXCLUDE on population (no housing precarity)
882	Dalla (2022) (ID:87857466)	Family Sex Trafficking Among the Bedia Caste of India: Defying the Dominant Human Trafficking Discourse	-EXCLUDE on country (High-Income)
883	Dallocchio (2021) (ID:87963026)	Women veterans: examining identity through an intersectional lens	-EXCLUDE on population (no housing precarity)
884	Dalton-Locke (2018) (ID:87850805)	Predictors of quality of care in mental health supported accommodation services in England: A multiple regression modelling study.	-EXCLUDE on intervention (service engagement/helpseeking behaviour)
885	Damian (2022) (ID:87848149)	Understanding the Health and Health-Related Social Needs of Youth Experiencing Homelessness: A Photovoice Study.	-EXCLUDE on intervention (service engagement/helpseeking behaviour)
886	Danforth (2020) (ID:87853132)	Disparities in Stage at Diagnosis in an Equal-access Integrated Delivery System: A Retrospective Cohort Study of 7244 Patients With Bladder Cancer	-EXCLUDE on population (no housing precarity)
887	Dang (2013) (ID:87851162)	Characteristics of natural mentoring relationships from the perspectives of homeless youth.	-EXCLUDE on population (people aged under 18 years)
888	Dang (2014) (ID:87848954)	Social connectedness and self-esteem: predictors of resilience in mental health among maltreated homeless youth.	-EXCLUDE on population (people aged under 18 years)
889	Dang (2022) (ID:87857977)	A LGBTQIA+ Creative Arts Program for Transitional-Aged Youth of Color in South Central Los Angeles: A Grant Proposal	-EXCLUDE on intervention (service engagement/helpseeking behaviour)

890	Dankyi (2022) (ID:87850616)	Street children in Ghana's golden triangle cities: Mental health needs and associated risks.	-EXCLUDE on country (High-Income)	
891	Dantas (2020) (ID:87853235)	Mortality from cervical cancer in Brazil: an ecological epidemiologic study of a 22-year analysis	-EXCLUDE on country (High-Income)	
892	Darbyshire (2006) (ID:87849340)	Engagement with health and social care services: perceptions of homeless young people with mental health problems	-EXCLUDE on date (2010)	
893	Darling (2021) (ID:87857682)	Living on the Margins Beyond Gender Binaries: What are the Challenges to Securing Rights	-EXCLUDE on population (no housing precarity)	
894	Darrat (2021) (ID:87853401)	Socioeconomic Disparities in Patient Use of Telehealth During the Coronavirus Disease 2019 Surge	-EXCLUDE on population (no housing precarity)	
895	das (2015) (ID:87858111)	Ethnicity	-EXCLUDE on evidence and form (evidence not in written form or presented as research output)	
896	Dasgupta (2021) (ID:87848201)	HIV-positive persons who inject drugs experience poor health outcomes and unmet needs for care services.	-EXCLUDE on population (no gender focus; women population <50)	
897	**Dashora (2012) (ID:87851242)**	**"Understand my side, my situation, and my story:" Insights into the service needs among substance-abusing homeless mothers.**	**-INCLUDE on title & abstract**	**-INCLUDE on full study**
898	Davey-Rothwell (2011) (ID:87849200)	Sexual networks and housing stability.	-EXCLUDE on population (no gender focus; women population <50)<br>-EXCLUDE - but review for literature	
899	David (2015) (ID:87849344)	Safety, Trust, and Treatment: Mental Health Service Delivery for Women Who Are Homeless	-INCLUDE on title & abstract	-EXCLUDE on intervention (intersectionality)
900	David (2019) (ID:87857875)	Immigrant Perpetrators of Domestic Homicide: Unique Risks and Service Needs	-EXCLUDE on intervention (service engagement/helpseeking behaviour)	
901	David (2021) (ID:87851470)	Mobiliser des partenaires pour lutter contre le VIH/sida : les risques de la démobilisation institutionnelle en cas d'épidémie faible et concentrée. Le cas du programme Amali au Maroc	-EXCLUDE on country (High-Income)	
902	David (2022) (ID:87851454)	Expanding the Duty to Rescue to Climate Migration	-EXCLUDE on intervention (service engagement/helpseeking behaviour)	
903	Davidson (2014) (ID:87851947)	What will It Take for Recovery to Flourish in Hong Kong?	-EXCLUDE on country (High-Income)	
904	Davidson (2018) (ID:87848566)	Monitoring Depression Rates in an Urban Community: Use of Electronic Health Records.	-EXCLUDE on intervention (service engagement/helpseeking behaviour)	
905	Davies (2013) (ID:87849063)	Hospital activity and cost incurred because of unregistered patients in England: considerations for current and new commissioners.	-EXCLUDE on intervention (service engagement/helpseeking behaviour)	
906	Davies (2014) (ID:87850542)	Writing motherhood for contemporary performance : three plays and thesis	-EXCLUDE on intervention (service engagement/helpseeking behaviour)	
907	Davis (2000) (ID:87850528)	An investigation into Housing Association responses towards women who have become homeless because of domestic violence	-EXCLUDE on date (2010)	
908	Davis (2015) (ID:87857581)	White Privilege and Clinical Social Work Practice: Reflections and Recommendations	-EXCLUDE on intervention (service engagement/helpseeking behaviour)	
909	Davis (2018) (ID:87852787)	Care of Incarcerated Patients	-EXCLUDE on population (no gender focus; women population <50)	
910	Davis (2018) (ID:87853109)	Listening to stories from women with heart disease: My experience as a nurse practitioner	-EXCLUDE on population (no housing precarity)	
911	Davis (2020) (ID:87850681)	Fighting isolation and celebrating gender diversity: Art therapy with transgender and gender expansive youth.	-EXCLUDE on population (no housing precarity)	
912	Davis (2022) (ID:87857751)	Trans Theory and Intimate Partner Violence	-EXCLUDE on population (no housing precarity)	
913	DAVIS (2022) (ID:87857164)	When children seek help in non-face-to-face settings: what do we know? A review of the literature	-EXCLUDE on population (people aged under 18 years)	
914	Davison (2023) (ID:87858037)	Influence of Poverty Simulation on Educators' Social Empathy and Educational Practices	-EXCLUDE on population (no housing precarity)	
915	Davy-Mendez (2021) (ID:87848182)	Non-fatal stimulant overdose among homeless and unstably housed women in San Francisco, California.	-EXCLUDE on intervention (service engagement/helpseeking behaviour)	
916	Dawes (2017) (ID:87849356)	Impact of volunteer-led running groups for women affected by homelessness: a qualitative study of the charity, A Mile in Her Shoes	-INCLUDE on title & abstract	-EXCLUDE on intervention (too programme-specific; not generalisable)

917	Dawes (2019) (ID:87851595)	'A Mile in Her Shoes': A qualitative exploration of the perceived benefits of volunteer led running groups for homeless women	-INCLUDE on title & abstract	-EXCLUDE on intervention (intersectionality)
918	Dawkins (2020) (ID:87848258)	A cluster feasibility trial to explore the uptake and use of e-cigarettes versus usual care offered to smokers attending homeless centres in Great Britain.	-EXCLUDE on population (women)	
919	Dawson (2013) (ID:87851196)	Mothering on the margins: Homeless women with an SUD and complex mental health co-morbidities.	-INCLUDE on title & abstract	-EXCLUDE on intervention (service engagement/helpseeking behaviour)
920	Dawson (2021) (ID:87852706)	Beyond polyphony: Open Dialogue in a Women's Shelter in Australia as a possibility for supporting violence-informed practice	-INCLUDE on title & abstract	-EXCLUDE on intervention (intersectionality)
921	Dawson-Rose (2020) (ID:87848314)	Trauma, substance use, and mental health symptoms in transitional age youth experiencing homelessness.	-EXCLUDE on population (women)	
922	Day (2020) (ID:87963025)	Applying intersectionality to partnerships between women's organizations and the criminal justice system in relation to domestic violence	-INCLUDE on title & abstract	-EXCLUDE on intervention (service engagement/helpseeking behaviour)
923	Day (2020) (ID:87963162)	An intersectional approach to improving the efficacy of partnerships between women's organisations and the criminal justice system in relation to domestic violence	-INCLUDE on title & abstract	-EXCLUDE on target group (not focused on women's behaviour/outcomes for women)
924	Dayton (2021) (ID:87848213)	Trends in overdose experiences and prevention behaviors among people who use opioids in Baltimore, MD, 2017-2019.	-EXCLUDE on intervention (service engagement/helpseeking behaviour)	
925	De (2019) (ID:87857172)	Young adolescents' help seeking behaviors and attitudes: an examination of an underserved community	-EXCLUDE on population (no housing precarity)	
926	de Araujo (2015) (ID:87853100)	A life insurance deterrent to risky behavior in Africa	-EXCLUDE on country (High-Income)	
927	de Beer (2020) (ID:87857578)	A reflection on Vuyani Vellem's longing for liberation: A spirituality of life and freedom	-EXCLUDE on intervention (service engagement/helpseeking behaviour)	
928	de Boer (2022) (ID:88019169)	Barriers and facilitators to treatment seeking and engagement amongst women with complex trauma histories	-EXCLUDE on population (no housing precarity)<br>-EXCLUDE - but review for literature	
929	de Bruin (2020) (ID:87851978)	The Urbanization-Household Gender Inequality Nexus: Evidence from Time Allocation in China	-EXCLUDE on country (High-Income)	
930	de Guise (2014) (ID:87848929)	Characteristics of patients with acute traumatic brain injury discharged against medical advice in a level 1 urban trauma centre.	-EXCLUDE on population (women)	
931	de Haan (2017) (ID:87858108)	Rural and urban livelihoods, social exclusion and social protection in sub-Saharan Africa	-EXCLUDE on country (High-Income)	
932	de Jesus (2020) (ID:87850684)	Mental health and challenges of transgender women: A qualitative study in Brazil and India.	-EXCLUDE on country (High-Income)	
933	de Medeiros (2015) (ID:87848893)	The Role of Relevancy and Social Suffering in "Generativity" Among Older Post-Soviet Women Immigrants.	-EXCLUDE on population (no housing precarity)	
934	de Vet (2017) (ID:87848613)	Critical Time Intervention for Homeless People Making the Transition to Community Living: A Randomized Controlled Trial.	-EXCLUDE on population (women)	
935	de Vet (2019) (ID:87853393)	Differences between homeless women and men before and after the transition from shelter to community living: A longitudinal analysis	-INCLUDE on title & abstract	-EXCLUDE on intervention (intersectionality)
936	**Deal (2023) (ID:87857451)**	**Centering Our Voices: Experiences of Violence Among Homeless African American Women**	**-INCLUDE on title & abstract**	**-INCLUDE on full study**
937	Dean (2010) (ID:87851388)	Gender differences in adolescents attending a drug and alcohol withdrawal service.	-INCLUDE on title & abstract	-EXCLUDE on intervention (service engagement/helpseeking behaviour)
938	DEANE (2007) (ID:87857284)	Impact of classroom presentations about health and help-seeking on rural Australian adolescents' intentions to consult health care professionals	-EXCLUDE on date (2010)	
939	Dearth (2015) (ID:87852015)	Relationship between trauma-related stress and coping strategies of mental health providers to the homeless	-EXCLUDE on population (no gender focus; women population <50)	
940	DeBeck (2011) (ID:87849208)	Public crack cocaine smoking and willingness to use a supervised inhalation facility: implications for street disorder.	-EXCLUDE on population (no housing precarity)	
941	DeBeck (2012) (ID:87851286)	The validity of reporting willingness to use a supervised injecting facility on subsequent program use among people who use injection drugs.	-EXCLUDE on population (women)	
942	Debon (2017) (ID:87853325)	Characterization of between-group inequality of longevity in European Union countries	-EXCLUDE on population (no housing precarity)	

943	Decemvirale (2021) (ID:87851975)	Knowing Your Place and Making Do: Radical Arts Activism in Black and Latino Los Angeles, 1968–1984	-EXCLUDE on intervention (service engagement/helpseeking behaviour)	
944	Dechman (2015) (ID:87851012)	Peer helpers' struggles to care for "others" who inject drugs.	-EXCLUDE on intervention (service engagement/helpseeking behaviour)	
945	Decker (2013) (ID:87848997)	Military sexual assault and homeless women veterans: clinical correlates and treatment preferences.	-INCLUDE on title & abstract	-EXCLUDE on intervention (service engagement/helpseeking behaviour)
946	Decker (2014) (ID:87848928)	Participation in a novel treatment component during residential substance use treatment is associated with improved outcome: a pilot study.	-EXCLUDE on intervention (service engagement/helpseeking behaviour)	
947	Decker (2021) (ID:87851891)	Incidence and predictors of violence from clients, intimate partners and police in a prospective US-based cohort of women in sex work	-INCLUDE on title & abstract	-EXCLUDE on intervention (service engagement/helpseeking behaviour)
948	DeFilippis (2017) (ID:87963312)	Embodying margin to center: Intersectional activism among queer liberation organizations	-EXCLUDE on intervention (service engagement/helpseeking behaviour)	
949	Dekel (2019) (ID:87850747)	Posttraumatic stress disorder upon admission to shelters among female victims of domestic violence: An ecological model of trauma.	-INCLUDE on title & abstract	-EXCLUDE on intervention (service engagement/helpseeking behaviour)
950	Dekker (2013) (ID:87849082)	Are long-term psychiatric patients causing more crisis consultations outside office hours in mental health care?.	-EXCLUDE on population (women)	
951	dela (2023) (ID:87857720)	Experiences of Migrants Living with HIV from HIV-Endemic Sub-Saharan African Countries: A Systematic Review of Qualitative Research Studies	-EXCLUDE on country (High-Income)	
952	Delichte (2017) (ID:87857911)	Development and Preliminary Testing of the Intimate Partner Violence, Abuse, and Control Scale (IP-V ACS)	-EXCLUDE on intervention (service engagement/helpseeking behaviour)	
953	Dememew (2020) (ID:87848297)	The yield of community-based tuberculosis and HIV among key populations in hotspot settings of Ethiopia: A cross-sectional implementation study.	-EXCLUDE on country (High-Income)	
954	Dempsey (2019) (ID:87853250)	Special Populations Disaster Care Considerations in Chronically Ill, Pregnant, and Morbidly Obese Patients	-EXCLUDE on population (no housing precarity)	
955	DENNIS (2012) (ID:87857341)	Not a minor offence: unaccompanied children locked up as part of the asylum system	-EXCLUDE on population (people aged under 18 years)	
956	Denson (2017) (ID:87848631)	Health Care Use and HIV-Related Behaviors of Black and Latina Transgender Women in 3 US Metropolitan Areas: Results From the Transgender HIV Behavioral Survey.	-INCLUDE on title & abstract	-EXCLUDE on intervention (service engagement/helpseeking behaviour)
957	Dent (2018) (ID:87858087)	Bridging the Gap of Race and Interfaith Relations: Connecting Humanity with Our Stories	-EXCLUDE on population (no housing precarity)	
958	Dentato (2019) (ID:87858085)	Peer-Based Education and Use of the SBIRT Model in Unique Settings with Transgender Young Adults	-EXCLUDE on population (no housing precarity)	
959	Deren (2010) (ID:87849288)	Migrant drug users: predictors of HIV-related sexual and injection risk behaviors.	-EXCLUDE on population (women)	
960	Dernaika (2022) (ID:87857991)	Reducing Stigmatization Related to Sexual Health Screenings	-EXCLUDE on population (no housing precarity)	
961	Derose (2017) (ID:87857669)	Factors contributing to food insecurity among women living with HIV in the Dominican Republic: A qualitative study	-EXCLUDE on country (High-Income)	
962	Dertadian (2023) (ID:87857865)	Injection drug use in an affluent beachside community in Sydney: An exploratory qualitative study	-EXCLUDE on population (no housing precarity)	
963	Desai (2020) (ID:87852936)	Community interventions with women's groups to improve women's and children's health in India: a mixed-methods systematic review of effects, enablers and barriers	-EXCLUDE on country (High-Income)	
964	Desai (2023) (ID:87850577)	Racial, ethnic, and sex differences in psychiatric diagnosis, mental health sequelae, and VHA service utilization among justice-involved veterans.	-EXCLUDE on intervention (service engagement/helpseeking behaviour)	
965	Desir (2020) (ID:87850703)	Interpersonal factors influencing risk for revictimization in two samples of young adults.	-EXCLUDE on intervention (service engagement/helpseeking behaviour)	
966	Desmennu (2018) (ID:87848516)	Behavioural risk factors for sexually transmitted infections and health seeking behaviour of street youths in Ibadan, Nigeria.	-EXCLUDE on country (High-Income)	
967	Devany (2023) (ID:87850573)	Disengaged youth? Exploring the lives of 'hidden neets' outside the benefits system.	-EXCLUDE on population (women)	
968	DEVECI (2012) (ID:87857388)	Trying to understand: promoting the psychosocial well-being of separated refugee children	-EXCLUDE on population (people aged under 18 years)	

969	DEVENNEY (2020) (ID:87857269)	Social work with unaccompanied asylum-seeking young people: reframing social care professionals as 'co-navigators'	-EXCLUDE on intervention (service engagement/helpseeking behaviour)	
970	Devi (2019) (ID:87857713)	Identity and Self-efficacy among Mathematically Successful African American Single Mothers in Urban Community College Contexts	-EXCLUDE on population (no housing precarity)	
971	Devilly (2019) (ID:87850763)	Crisis support services in night-time entertainment districts: Changes in demand following changes in alcohol legislation.	-EXCLUDE on population (women)	
972	Devine (2021) (ID:87857851)	Australia's disability employment services program: Participant perspectives on factors influencing access to work	-EXCLUDE on population (no housing precarity)	
973	DeVOE (2007) (ID:87857315)	Post-September 11 mental health service help seeking among a group of highly exposed New York city patients	-EXCLUDE on date (2010)	
974	DEVOY (2017) (ID:87857121)	Help-seeking intentions for early dementia diagnosis in a sample of Irish adults	-EXCLUDE on population (no housing precarity)	
975	Dewey (2016) (ID:87963066)	Women of the street: How the criminal justice-social services alliance fails women in prostitution	-EXCLUDE on evidence and form (evidence not in written form or presented as research output)<br>-EXCLUDE - but review for literature	
976	Dewey (2017) (ID:87851708)	Harm Reduction and Help Seeking	-INCLUDE on title & abstract	-EXCLUDE on intervention (intersectionality)
977	Dewey (2017) (ID:87857715)	Transitional Housing Facilities for Women Leaving the Sex Industry: Informed by Evidence or Ideology?	-INCLUDE on title & abstract	-EXCLUDE on intervention (intersectionality)
978	Dewi (2021) (ID:87851520)	Oncologic Head and Neck Reconstructive Microsurgery during the COVID-19 Pandemic in Dharmais Cancer Hospital-National Cancer Center, Jakarta, Indonesia	-EXCLUDE on country (High-Income)	
979	Dhaliwal (2023) (ID:87857661)	How Black Transgender Young Women Find Resiliency While Navigating the Child Welfare System	-INCLUDE on title & abstract	-EXCLUDE on intervention (service engagement/helpseeking behaviour)
980	Dhaske (2014) (ID:87857703)	The lived experience of women affected with matted hair in southwestern India	-EXCLUDE on country (High-Income)	
981	Di Santo (2016) (ID:87850910)	'Mommy that's the exit.': Empowering homeless mothers to support their children's daily literacy experiences.	-EXCLUDE on intervention (service engagement/helpseeking behaviour)	
982	Diamond-Smith (2017) (ID:87852666)	Associations of women's position in the household and food insecurity with family planning use in Nepal	-EXCLUDE on country (High-Income)	
983	Diaz (2016) (ID:87848763)	Medicaid expansion and mental health: A Minnesota case study.	-EXCLUDE on population (women)	
984	Dickerson (2016) (ID:87857630)	Chinese female graduate students on us campuses: Negotiating classroom silence, the leftover woman and the good woman discourses	-EXCLUDE on population (no housing precarity)	
985	Dickins (2021) (ID:87850629)	Physical and behavioral health characteristics of aging homeless women in the United States: An integrative review.	-INCLUDE on title & abstract	-EXCLUDE on intervention (service engagement/helpseeking behaviour)
986	Dickins (2023) (ID:87850559)	Biopsychosocial model of traumatic stress symptoms in women experiencing homelessness: A qualitative descriptive study.	-INCLUDE on title & abstract	-EXCLUDE on intervention (service engagement/helpseeking behaviour)
987	Dierst-Davies (2016) (ID:87850915)	Gaps in ancillary services among hiv-infected substance using ryan-white recipients in los angeles county.	-EXCLUDE on population (women)	
988	Dietrich-Ragon (2015) (ID:87849301)	Family and friends of persons in a situation of residential precarity: The strength and weakness of social ties as they relate to housing deprivation (homelessness)	-EXCLUDE on population (no gender focus; women population <50)<br>-EXCLUDE - but review for literature	
989	DiGuiseppi (2020) (ID:87848269)	Polysubstance use among youth experiencing homelessness: The role of trauma, mental health, and social network composition.	-EXCLUDE on intervention (service engagement/helpseeking behaviour)	
990	DiGuiseppi (2021) (ID:87850641)	Social network correlates of education and employment service use among youth experiencing homelessness: A longitudinal study.	-EXCLUDE on intervention (service engagement/helpseeking behaviour)	
991	Dillon (2023) (ID:87848135)	Implementing universal suicide screening in a large healthcare system's hospitals: rates of screening, suicide risk, and documentation of subsequent psychiatric care.	-EXCLUDE on intervention (service engagement/helpseeking behaviour)	
992	Dillon (2023) (ID:87963371)	The intersectionality of women's substance use.	-EXCLUDE on population (no housing precarity)	
993	DiMarco (2010) (ID:87849259)	Access to and utilization of oral health care by homeless children/families.	-INCLUDE on title & abstract	-EXCLUDE on intervention (service engagement/helpseeking behaviour)
994	Dinh (2012) (ID:87853291)	Sex of Newborns Associated With Place and Mode of Delivery: A Population-Based Study in Northern Vietnam	-EXCLUDE on country (High-Income)	

995	Dionisi (2020) (ID:87848292)	Make Mission Impossible Feasible: The Experience of a Multidisciplinary Team Providing Treatment for Alcohol Use Disorder to Homeless Individuals.	-EXCLUDE on population (women)	
996	Diversity Includes Disability:... (2022) (ID:87857934)	Diversity Includes Disability: Exploring Inequities in Financial Services for Persons with Disabilities, Including Those Newly Disabled Due to Long-Term COVID. Congressional Hearing, 2022-05-24	-EXCLUDE on population (no housing precarity)	
997	Djuric (2020) (ID:87852759)	Primary care services tailored for adult and adolescent homeless persons: a scoping review protocol	-EXCLUDE on evidence and form (evidence not in written form or presented as research output)	
998	do (2019) (ID:87851917)	"We are the UPA ourselves": social support networks on the health care for the homeless in a smalltown	-EXCLUDE on population (women)	
999	Do We Have the Knowledge... (PARSELL) (ID:87849342)	Do We Have the Knowledge to Address Homelessness?	-EXCLUDE on intervention (service engagement/helpseeking behaviour)	
####	Dobransky-Fasiska (2012) (ID:87849157)	A community-academic partnership develops a more responsive model to providing depression care to disadvantaged adults in the US.	-EXCLUDE on population (women)	
####	DOHERTY (2010) (ID:87857349)	Gender and self-reported mental health problems: predictors of help seeking from a general practitioner	-EXCLUDE on population (no housing precarity)	
####	DOIG (2009) (ID:87857396)	'Jumping through hoops': parents' experiences with seeking respite care for children with special needs	-EXCLUDE on date (2010)	
####	Dolamore (2018) (ID:87963249)	Providing solutions to LGBT homeless youth: Lessons from Baltimore's youth empowered society	-EXCLUDE on population (people aged under 18 years)	
####	Dominic (2020) (ID:87853312)	Impact of Social Support on Colorectal Cancer Screening among Adult Hispanics/Latinos: A Randomized Community-based Study in Central Pennsylvania	-EXCLUDE on population (women)	
####	Dominice (2018) (ID:87848600)	Vulnerability in the clinic: case study of a transcultural consultation.	-EXCLUDE on population (no housing precarity)	
####	Donaldson (2016) (ID:87849358)	Wounded veterans: reintegration through adventure-based experience ; a narrative inquiry	-EXCLUDE on population (women)	
####	Donohoe (2013) (ID:87851129)	Public health and social justice: A Jossey-Bass reader.	-EXCLUDE on population (no housing precarity)	
####	DONOVAN (2011) (ID:87857071)	Seeking help from the enemy: help-seeking strategies of those in same-sex relationships who have experienced domestic abuse	-EXCLUDE on population (no gender focus; women population <50)<br>-EXCLUDE - but review for literature	
####	Doorley (2017) (ID:87848684)	Buprenorphine shared medical appointments for the treatment of opioid dependence in a homeless clinic.	-EXCLUDE on population (women)	
####	Dopp (2022) (ID:87852637)	Supportive Housing for Sexual and Gender Minority Individuals With Criminal Justice Histories: Challenges and Opportunities Identified by Providers and Clients	-INCLUDE on title & abstract	-EXCLUDE on target group (youth)
####	DORE (2015) (ID:87857006)	Prison leavers and homelessness	-EXCLUDE on population (no gender focus; women population <50)<br>-EXCLUDE - but review for literature	
####	Dore-Gauthier (2019) (ID:87848446)	How to help homeless youth suffering from first episode psychosis and substance use disorders? The creation of a new intensive outreach intervention team.	-EXCLUDE on population (women)	
####	Dore-Gauthier (2020) (ID:87848357)	Specialized assertive community treatment intervention for homeless youth with first episode psychosis and substance use disorder: A 2-year follow-up study.	-EXCLUDE on population (women)	
####	DORLING (2012) (ID:87857116)	Seeking support: a guide to rights and entitlements of separated children	-EXCLUDE on population (people aged under 18 years)	
####	Douge (2014) (ID:87853255)	Social Support and Employment Status Modify the Effect of Intimate Partner Violence on Depression Symptom Severity in Women: Results from the 2006 Behavioral Risk Factor Surveillance System Survey	-EXCLUDE on intervention (service engagement/helpseeking behaviour)	
####	Douglas (2010) (ID:87857686)	Mothers, Domestic Violence, and Child Protection	-INCLUDE on title & abstract	-EXCLUDE on intervention (service engagement/helpseeking behaviour)
####	Douglass (2018) (ID:87848508)	Health Care for the Homeless Transgender Community: Psychiatric Services and Transition Care at a Student-Run Clinic.	-EXCLUDE on intervention (service engagement/helpseeking behaviour)	

####	Dove (2022) (ID:87857754)	"Data makes the story come to life:" understanding the ethical and legal implications of Big Data research involving ethnic minority healthcare workers in the United Kingdom—a qualitative study	-EXCLUDE on population (no housing precarity)	
####	Dowling (2018) (ID:87858090)	Confronting capital's care fix: care through the lens of democracy	-EXCLUDE on intervention (service engagement/helpseeking behaviour)	
####	Dowshen (2011) (ID:87853216)	Religiosity As a Protective Factor Against HIV Risk Among Young Transgender Women	-EXCLUDE on population (no housing precarity)	
####	Doyle (2002) (ID:87850520)	A woman's space? : meanings of home and homelessness	-EXCLUDE on date (2010)	
####	Dozier (2019) (ID:87857705)	Lived Experiences of Caucasian American Parents and Their Perceptions of the Chinese-Born Adopted Daughters' Ethnic Identity Formation and Quality of Attachment	-EXCLUDE on population (no housing precarity)	
####	Draanen (2013) (ID:87849033)	Reducing service and substance use among frequent service users: a brief report from the Toronto Community Addictions Team.	-EXCLUDE on population (no housing precarity)	
####	Drake (2011) (ID:87851332)	A prospective 2-year study of emergency department patients with early-phase primary psychosis or substance-induced psychosis.	-EXCLUDE on population (women)	
####	Drake (2022) (ID:87857769)	Seeing Our Voices: Using Photovoice with Trauma-Affected Black Women College Students Within a PWI to Explore Success and Persistence	-EXCLUDE on population (no housing precarity)	
####	Dreier (2018) (ID:87851923)	Why America Needs More Social Housing	-EXCLUDE on intervention (service engagement/helpseeking behaviour)	
####	Dressel (2020) (ID:87848298)	Nia Imani Model of Care's Impact on Homeless African-American Women.	-INCLUDE on title & abstract	-EXCLUDE on intervention (intersectionality)
####	Drewnowski (2023) (ID:87848139)	Neighborhood deprivation and residential property values do not affect weight loss at 1 or 3 years after bariatric surgery.	-EXCLUDE on population (no housing precarity)	
####	Du (2013) (ID:87848998)	"She was truly an angel": Women with disabilities' satisfaction with hospital-based sexual assault and domestic violence services.	-INCLUDE on title & abstract	-EXCLUDE on target group (no housing precarity)
####	Du (2020) (ID:87963212)	Marginalized communities, emerging technologies, and social innovation in the digital age: Introduction to the special issue	-EXCLUDE on population (no housing precarity)	
####	Ducre (2018) (ID:87857563)	The Black feminist spatial imagination and an intersectional environmental justice	-EXCLUDE - but review for literature<br>-EXCLUDE on intervention (service engagement/helpseeking behaviour)	
####	**Dudley (2017) (ID:87963157)**	**Domestic abuse and women with 'no recourse to public funds': the state's role in shaping and reinforcing coercive control**	**-INCLUDE on title & abstract**	**-INCLUDE on full study**
####	Dudley (2022) (ID:87857873)	Promoting Success of Military Veteran Nursing Students through Creating Connection and Community	-EXCLUDE on population (no housing precarity)	
####	**Duff (2015) (ID:87851000)**	**Sex work and motherhood: Social and structural barriers to health and social services for pregnant and parenting street and off-street sex workers.**	**-INCLUDE on title & abstract**	**-INCLUDE on full study**
####	Duff (2016) (ID:87853156)	Barriers to Viral Suppression Among Female Sex Workers: Role of Structural and Intimate Partner Dynamics	-INCLUDE on title & abstract	-EXCLUDE on intervention (service engagement/helpseeking behaviour)
####	Duggan (2023) (ID:87857540)	"Our Teaching Is Rocking Their Ontological Security": Exploring the Emotional Labour of Transformative Criminal Justice Pedagogy	-EXCLUDE on population (no housing precarity)	
####	Duncan (2019) (ID:87852654)	Provider Perspectives on Sex Trafficking: Victim Pathways, Service Needs, & Blurred Boundaries	-INCLUDE on title & abstract	-EXCLUDE on target group (not focused on women's behaviour/outcomes for women)
####	Duncan (2019) (ID:87857828)	The social context of HIV prevention and care among black men who have sex with men in three U.S. cities: The neighborhoods and networks (N2) cohort study	-EXCLUDE on population (women)	
####	Dunn (2019) (ID:87848373)	Differential adoption of opioid agonist treatments in detoxification and outpatient settings.	-EXCLUDE on intervention (service engagement/helpseeking behaviour)	
####	Dunne (2012) (ID:87849117)	Mental health services for homeless: patient profile and factors associated with suicide and homicide.	-EXCLUDE on population (women)	
####	Dupont (2004) (ID:87853701)	Help-Seeking Behaviors of Marginalized Battered Women: Theoretical and Policy Implications	-EXCLUDE on date (2010)	

####	Duran (2021) (ID:87963134)	Food and housing insecurity for Latinx/a/o college students: Advancing an intersectional research agenda	-EXCLUDE on population (women)	
####	Durbin (2014) (ID:87848941)	Predictors of unmet need for medical care among justice-involved persons with mental illness.	-EXCLUDE on intervention (service engagement/helpseeking behaviour)	
####	Durbin (2019) (ID:87848404)	Recovery education for people experiencing housing instability: An evaluation protocol.	-EXCLUDE on population (women)	
####	Durbin (2019) (ID:87848445)	Are resilience and perceived stress related to social support and housing stability among homeless adults with mental illness?.	-EXCLUDE on population (women)	
####	Durfee (2012) (ID:87849105)	Characteristics related to protection order use among victims of intimate partner violence.	-INCLUDE on title & abstract	-EXCLUDE on intervention (intersectionality)
####	Dutta (2011) (ID:87853436)	Predictors of Extraordinary Survival in the Iowa Established Populations for Epidemiologic Study of the Elderly: Cohort Follow-Up to "Extinction"	-EXCLUDE on population (no housing precarity)	
####	Dutton (2015) (ID:87848871)	Coordinated public health initiatives to address violence against women and adolescents.	-INCLUDE on title & abstract	-EXCLUDE on intervention (intersectionality)
####	DWIDAR (2022) (ID:87857459)	Coalitional Lobbying and Intersectional Representation in American Rulemaking	-EXCLUDE on population (no housing precarity)	
####	DWORSKY (2012) (ID:87856967)	The parenting experiences of homeless adolescent mothers and mothers-to-be: perspectives from a shelter sample	-INCLUDE on title & abstract	-EXCLUDE on intervention (service engagement/helpseeking behaviour)
####	Dyb (2016) (ID:87848722)	Housing First or no housing? Housing and homelessness at the end of alcohol and drug treatment.	-EXCLUDE on population (women)	
####	Dykema (2017) (ID:87857937)	Relationships among Black Women's Wellness, Gendered-Racial Identity, and Mental Health Symptoms	-EXCLUDE on population (no housing precarity)	
####	Dziewanski (2020) (ID:87857473)	Femme Fatales: Girl Gangsters and Violent Street Culture in Cape Town	-EXCLUDE on country (High-Income)	
####	Dzul-Church (2010) (ID:87849250)	"I'm sitting here by myself ...": experiences of patients with serious illness at an Urban Public Hospital.	-EXCLUDE on population (women)	
####	Eade (2013) (ID:87851159)	Chlamydia screening in young people as an outcome of a HEADSS; Home, education, activities, drug and alcohol use, sexuality and suicide youth psychosocial assessment tool.	-EXCLUDE on intervention (service engagement/helpseeking behaviour)	
####	Earle-Brown (2022) (ID:87963048)	Homeless women Don't wear Prada: The geographies of beauty standards and the bodies of homeless women	-EXCLUDE on intervention (service engagement/helpseeking behaviour)	
####	Earnshaw (2013) (ID:87857613)	Stigma and Racial/Ethnic HIV Disparities: Moving Toward Resilience	-EXCLUDE on population (no housing precarity)	
####	Earnshaw (2015) (ID:87857655)	Stigma and Racial/Ethnic HIV Disparities: Moving Toward Resilience	-EXCLUDE on intervention (service engagement/helpseeking behaviour)	
####	Easteal (2022) (ID:87857714)	Teaching Journalists About Violence Against Women Best Reportage Practices: An Australian Case Study	-EXCLUDE on intervention (service engagement/helpseeking behaviour)	
####	Eastwood (2021) (ID:87850731)	Young transgender women of color: Homelessness, poverty, childhood sexual abuse and implications for HIV care.	-INCLUDE on title & abstract	-EXCLUDE on intervention (service engagement/helpseeking behaviour)
####	Eaton (2017) (ID:87851936)	Interventions to increase use of services; Mental Health Awareness in Nigeria	-EXCLUDE on country (High-Income)	
####	Eckenwiler (2018) (ID:87853137)	Displacement and solidarity: An ethic of place-making	-EXCLUDE on evidence and form (evidence not in written form or presented as research output)	
####	Ecker (2016) (ID:87848735)	Individual, Housing, and Neighborhood Predictors of Psychological Integration Among Vulnerably Housed and Homeless Individuals.	-EXCLUDE on intervention (service engagement/helpseeking behaviour)	
####	Ecker (2018) (ID:87852892)	Sexual Intimacy, Mental Illness, and Homelessness	-EXCLUDE on population (women)	
####	Ecker (2020) (ID:87852820)	Pathways Into Homelessness Among LGBTQ2S Adults	-INCLUDE on title & abstract	-EXCLUDE on intervention (service engagement/helpseeking behaviour)
####	Edens (2011) (ID:87849184)	Chronically homeless women report high rates of substance use problems equivalent to chronically homeless men.	-INCLUDE on title & abstract	-EXCLUDE on intervention (service engagement/helpseeking behaviour)
####	Edens (2011) (ID:87849211)	Does active substance use at housing entry impair outcomes in supported housing for chronically homeless persons?.	-EXCLUDE on population (women)	
####	Edens (2012) (ID:87849158)	Rates and correlates of pathological gambling among VA mental health service users.	-EXCLUDE on population (no gender focus; women population <50)	
####	Edwards (2017) (ID:87848671)	Co-Occurrence of and Recovery from Substance Abuse and Lifespan Victimization: A Qualitative Study of Female Residents in Trauma-Informed Sober Living Homes.	-INCLUDE on title & abstract	-EXCLUDE on intervention (intersectionality)

####	Edwards (2023) (ID:87963265)	Disclosure of sexual assault among sexual and gender minorities: A systematic literature review	-EXCLUDE on population (no housing precarity)<br>-EXCLUDE - but review for literature	
####	Eger (2018) (ID:87857645)	Communicating Organizational and Transgender Intersectional Identities: An Ethnography of a Transgender Outreach Center	-EXCLUDE on population (no housing precarity)	
####	Eghaneyan (2019) (ID:87857592)	Mental Health Help-Seeking Experiences of Hispanic Women in the United States: Results from a Qualitative Interpretive Meta-Synthesis	-EXCLUDE on population (no housing precarity)	
####	Eichler (2017) (ID:87963082)	Add female veterans and stir? A feminist perspective on gendering veterans research	-EXCLUDE on intervention (service engagement/helpseeking behaviour)	
####	Eichler (2021) (ID:87963138)	Mind the gap: Sex, gender, and intersectionality in military-to-civilian transitions	-EXCLUDE on population (no housing precarity)	
####	Eisen (2015) (ID:87848870)	Mental health and quality of life among veterans employed as peer and vocational rehabilitation specialists.	-EXCLUDE on population (women)	
####	Eklund (2020) (ID:87848359)	Perceived occupational balance and well-being among people with mental illness living in two types of supported housing.	-EXCLUDE on intervention (service engagement/helpseeking behaviour)	
####	El Ghaziri (2019) (ID:87848370)	Protocol of a longitudinal study on the specific needs of Syrian refugee families in Switzerland.	-EXCLUDE on population (women)	
####	El-Khani (2018) (ID:87850838)	Syria: Refugee parents' experiences and need for parenting support in camps and humanitarian settings.	-EXCLUDE on country (High-Income)	
####	Elaine (2020) (ID:87963164)	What is intersectionality and why is it important in oral health research?	-EXCLUDE on intervention (service engagement/helpseeking behaviour)	
####	Eliminating Health Disparities:... (Bharel) (ID:87851503)	Eliminating Health Disparities: Innovative Methods to Improve Cervical Cancer Screening in a Medically Underserved Population.	-INCLUDE on title & abstract	-EXCLUDE on intervention (service engagement/helpseeking behaviour)
####	Elkbuli (2020) (ID:87853212)	Gender disparities in lung cancer survival from an enriched Florida population-based cancer registry	-EXCLUDE on population (no housing precarity)	
####	Ellard-Gray (2015) (ID:87857826)	Finding the Hidden Participant: Solutions for Recruiting Hidden, Hard-to-Reach, and Vulnerable Populations	-EXCLUDE on intervention (service engagement/helpseeking behaviour)	
####	Ellen (2015) (ID:87848866)	Evaluation of the effect of human immunodeficiency virus-related structural interventions: the connect to protect project.	-EXCLUDE on intervention (service engagement/helpseeking behaviour)	
####	Ellis (2013) (ID:87853383)	Challenges to Implementation of an Epidermal Growth Factor Receptor Testing Strategy for Non-Small-Cell Lung Cancer in a Publicly Funded Health Care System	-EXCLUDE on population (no housing precarity)	
####	Ellison (2020) (ID:87848333)	Impact of Peer Specialist Services on Residential Stability and Behavioral Health Status Among Formerly Homeless Veterans With Cooccurring Mental Health and Substance Use Conditions.	-EXCLUDE on population (women)	
####	Ely (2017) (ID:87848615)	A trauma-informed examination of the hardships experienced by abortion fund patients in the United States.	-INCLUDE on title & abstract	-EXCLUDE on intervention (service engagement/helpseeking behaviour)
####	Embleton (2013) (ID:87851166)	Lost indeed, but not in translation: A response to Monteiro.	-EXCLUDE on evidence and form (evidence not in written form or presented as research output)	
####	Embleton (2023) (ID:87857418)	Intersectional Stigma and Implementation of HIV Prevention and Treatment Services for Adolescents Living with and at Risk for HIV: Opportunities for Improvement in the HIV Continuum in Sub-Saharan Africa	-EXCLUDE on country (High-Income)	
####	Emerson (2020) (ID:87852967)	Voter Registration and Jail-Incarcerated Women: Are Justice-Involved Women Civically Engaged?	-EXCLUDE on intervention (service engagement/helpseeking behaviour)	
####	**England (2022) (ID:87852631)**	**'This is how it works here': the spatial deprioritisation of trans people within homelessness services in Wales**	**-INCLUDE on title & abstract**	**-INCLUDE on full study**
####	Engle (2022) (ID:87857499)	Sexual Violence, Intangible Harm, and the Promise of Transformative Remedies	-EXCLUDE on intervention (service engagement/helpseeking behaviour)<br>-EXCLUDE - but review for literature	
####	Engstrom (2017) (ID:87848626)	Correlates of HIV Risks Among Women on Probation and Parole.	-EXCLUDE on intervention (service engagement/helpseeking behaviour)	
####	Engström (2022) (ID:87858082)	Vulnerabilisation: Between mainstreaming and human rights overreach	-EXCLUDE on population (no housing precarity)	

####	Enguita-Fernandez (2020) (ID:87850658)	The COVID-19 epidemic through a gender lens: What if a gender approach had been applied to inform public health measures to fight the COVID-19 pandemic?	-EXCLUDE on intervention (service engagement/helpseeking behaviour)	
####	Erb (2017) (ID:87851744)	12 Days Of Charitable Giving: Women's Lunch Place.	-INCLUDE on title & abstract	-EXCLUDE on evidence and form (evidence not in written form or presented as research output)
####	Erdem (2015) (ID:87850983)	Can a house become more than a home? effects of housing assistance and supportive services on promoting capabilities among homeless mothers.	-INCLUDE on title & abstract	-EXCLUDE on intervention (service engagement/helpseeking behaviour)
####	Erez (2018) (ID:87963356)	Intersectionality, immigration, and domestic violence	-EXCLUDE on intervention (service engagement/helpseeking behaviour)<br>-EXCLUDE - but review for literature	
####	Erickson (2020) (ID:87853205)	Violence and other social structural factors linked to incarceration for women living with HIV in Metro Vancouver: need for trauma-informed HIV care in prisons and post-Release	-EXCLUDE on intervention (service engagement/helpseeking behaviour)	
####	Ernst (2020) (ID:87850668)	A guide for schools: Understanding and working with parents of transgender students.	-EXCLUDE on evidence and form (evidence not in written form or presented as research output)	
####	Ervin (2022) (ID:87850583)	Characteristics associated with homeless pregnant women in Columbus, Ohio.	-INCLUDE on title & abstract	-EXCLUDE on intervention (service engagement/helpseeking behaviour)
####	Esen (2017) (ID:87853084)	The homeless pregnant woman	-EXCLUDE on intervention (service engagement/helpseeking behaviour)	
####	ESPLEY (2004) (ID:87857200)	Welcoming children into your neighbourhood	-EXCLUDE on date (2010)	
####	Estrada (2011) (ID:87857495)	Intersectional Dignities: Latino Immigrant Street Vendor Youth in Los Angeles	-EXCLUDE on intervention (service engagement/helpseeking behaviour)	
####	Estrada (2022) (ID:87858094)	Lived Experiences of Transgender Young Adults Transitioning During the COVID-19 Pandemic	-EXCLUDE on population (no housing precarity)	
####	Estrellado (2023) (ID:87857520)	Introduction to the Special Issue on Feminist Therapy with Transgender, Nonbinary, and Gender Expansive People	-EXCLUDE on population (no housing precarity)<br>-EXCLUDE - but review for literature	
####	Estrine (2011) (ID:87851300)	Service delivery for vulnerable populations: New directions in behavioral health.	-EXCLUDE on population (women)<br>-EXCLUDE - but review for literature	
####	Etty (2023) (ID:87848138)	The sociodemographic characteristics and social determinants of visual impairment in a homeless population in the Montreal area.	-EXCLUDE on population (women)	
####	Evangelidou (2023) (ID:87852977)	Health promotion strategies toward improved healthcare access for migrants and refugees in Europe: MyHealth recommendations	-EXCLUDE on population (no gender focus; women population <50)	
####	EVANS (2005) (ID:87857329)	In what ways are adolescents who engage in self-harm or experience thoughts of self-harm different in terms of help-seeking, communication and coping strategies?	-EXCLUDE on date (2010)	
####	Evans (2014) (ID:87851661)	Health Care Utilization in Homeless Youth	-EXCLUDE on population (no gender focus; women population <50)	
####	Evans (2015) (ID:87848822)	Heterogeneity of Mental Health Service Utilization and High Mental Health Service Use Among Women Eight Years After Initiating Substance Use Disorder Treatment.	-INCLUDE on title & abstract	-EXCLUDE on intervention (service engagement/helpseeking behaviour)
####	Evans (2018) (ID:87852993)	Psychosocial Factors that Shape Substance Abuse and Related Mental Health of Women Military Veterans who Use Community-Based Services	-EXCLUDE on population (no housing precarity)	
####	Evans (2020) (ID:87853123)	COVID-19 and violence: a research call to action	-EXCLUDE on country (High-Income)	
####	Evans (2021) (ID:87852906)	Domestic Violence in Atlanta, Georgia Before and During COVID-19	-EXCLUDE on intervention (service engagement/helpseeking behaviour)	
####	Evensen (2017) (ID:87848689)	Employment outcome and predictors of competitive employment at 2-year follow-up of a vocational rehabilitation programme for individuals with schizophrenia in a high-income welfare society.	-EXCLUDE on population (no housing precarity)	
####	Everett (2018) (ID:87858123)	Interfaith Dialogue and Faith-Based Social Activism in a State of Emergency: laïcité and the Crisis of Religion in France	-EXCLUDE on population (no housing precarity)	
####	Evidence from a Longitudinal... (2021) (ID:87851506)	Evidence from a Longitudinal Photovoice and Interview Assessment with Congolese Refugee Women in the Midwestern United States	-INCLUDE on title & abstract	-EXCLUDE on target group (no housing precarity)

####	Evidence of Early Emergence... (Dawson) (ID:87851563)	Evidence of Early Emergence of the Primary Dentition in a Northern Plains American Indian Population	-EXCLUDE on population (no housing precarity)	
####	Ex-custodial homelessness... (Kelly) (ID:87849338)	Ex-custodial homelessness support service South Australia and reduced reoffending	-EXCLUDE on population (women)	
####	Exavery (2020) (ID:87848273)	ART use and associated factors among HIV positive caregivers of orphans and vulnerable children in Tanzania.	-EXCLUDE on country (High-Income)	
####	Eyrich-Garg (2011) (ID:87853099)	Sheltered in cyberspace? Computer use among the unsheltered 'street' homeless	-EXCLUDE on population (women)	
####	Ezard (2018) (ID:87848542)	A managed alcohol program in Sydney, Australia: Acceptability, cost-savings and non-beverage alcohol use.	-EXCLUDE on intervention (service engagement/helpseeking behaviour)	
####	Fabian (2022) (ID:87853341)	Sex, gender, and retinoblastoma: analysis of 4351 patients from 153 countries	-EXCLUDE on population (no housing precarity)	
####	Fabienne (2022) (ID:87851473)	Paediatric refugees from Ukraine: guidance for health care providers	-INCLUDE on title & abstract	-EXCLUDE on intervention (service engagement/helpseeking behaviour)
####	Factores de riesgo para... (Murga-Bendezú) (ID:87851564)	Factores de riesgo para mortalidad en mujeres adultas mayores con síndrome de fragilidad.	-EXCLUDE on country (High-Income)	
####	Factores relacionados... (Gómez) (ID:87851655)	Factores relacionados con conducta suicida en personas hospitalizadas en una unidad psiquiátrica.	-EXCLUDE on population (no housing precarity)	
####	Fajardo-Bullon (2019) (ID:87848395)	Homelessness and self-rated health: evidence from a national survey of homeless people in Spain.	-EXCLUDE on population (women)	
####	Fallin-Bennett (2019) (ID:87848421)	Call to Action to Reduce Tobacco Use During Pregnancy.	-EXCLUDE on population (no housing precarity)	
####	Familiar (2021) (ID:87851556)	Conflict-related violence and mental health among self-settled Democratic Republic of Congo female refugees in Kampala, Uganda – a respondent driven sampling survey.	-EXCLUDE on country (High-Income)	
####	Fang (2018) (ID:87853316)	Excess mortality in children born to opioid-addicted parents: A national register study in Taiwan	-EXCLUDE on population (people aged under 18 years)	
####	Fannin (2015) (ID:87848854)	Using Functional Needs and Personal Care Assistance Rather Than Disability Status During Chronic Care Triage in Community Mass Care.	-EXCLUDE on population (women)	
####	FANSLOW (2010) (ID:87857068)	Help-seeking behaviors and reasons for help seeking reported by a representative sample of women victims of intimate partner violence in New Zealand	-INCLUDE on title & abstract	-EXCLUDE on intervention (intersectionality)
####	FARGAS-MALET (2018) (ID:87857314)	The mental health and help-seeking behaviour of children and young people in care in Northern Ireland: making services accessible and engaging	-EXCLUDE on population (people aged under 18 years)	
####	Fargo (2017) (ID:87848656)	Homelessness following disability-related discharges from active duty military service in Afghanistan and Iraq.	-EXCLUDE on population (women)	
####	Farhoudian (2022) (ID:87851880)	How Substance Use Treatment Services in Iran Survived Despite a Dual Catastrophic Situation	-EXCLUDE on country (High-Income)	
####	FARRAND (2007) (ID:87857271)	Intention of adolescents to seek professional help for emotional and behavioural difficulties	-EXCLUDE on date (2010)	
####	Farvid (2021) (ID:87857523)	The health and wellbeing of transgender and gender non-conforming people of colour in the United States: A systematic literature search and review	-EXCLUDE on population (no housing precarity)	
####	Fast (2014) (ID:87851090)	The multiple truths about crystal meth among young people entrenched in an urban drug scene: A longitudinal ethnographic investigation.	-EXCLUDE on intervention (service engagement/helpseeking behaviour)	
####	Fatiregun (2019) (ID:87853439)	Prevalence and Correlates of Unmet Supportive Needs of Nigerian Patients With Cancer	-EXCLUDE on country (High-Income)	
####	Fauer (2020) (ID:87850691)	Catalyzing a nursing response to healthcare discrimination against transgender and nonbinary individuals.	-EXCLUDE on population (no housing precarity)	
####	FAVER (2009) (ID:87857273)	Seeking our place in the web of life: animals and human spirituality	-EXCLUDE on date (2010)	
####	Fayfman (2020) (ID:87851898)	Barriers to diabetic foot care in a disadvantaged population: A qualitative assessment	-EXCLUDE on population (no housing precarity)	
####	Fazel (2014) (ID:87849297)	The health of homeless people in high-income countries: Descriptive epidemiology, health consequences, and clinical and policy recommendations	-EXCLUDE on intervention (service engagement/helpseeking behaviour)<br>-EXCLUDE - but review for literature	
####	FEANTSA (2022) (ID:88019164)	Housing First & Women - Case studies from across Europe	-INCLUDE on title & abstract	-EXCLUDE on intervention (intersectionality)

####	Federico (2018) (ID:87848545)	United States Pediatricians' Attitudes Regarding Public Policies for Low-Income Children and Their Profession's Advocacy Priorities.	-EXCLUDE on intervention (service engagement/helpseeking behaviour)	
####	Fedock (2013) (ID:87851139)	Service needs for incarcerated adults: Exploring gender differences.	-EXCLUDE on intervention (service engagement/helpseeking behaviour)	
####	Fekadu (2019) (ID:87848382)	The psychosis treatment gap and its consequences in rural Ethiopia.	-EXCLUDE on country (High-Income)	
####	Feldman (2012) (ID:87849096)	The prevalence and correlates of receiving confirmatory HIV test results among newly diagnosed HIV-positive individuals at a community-based testing center.	-EXCLUDE on intervention (service engagement/helpseeking behaviour)	
####	FELDMAN (2017) (ID:87857115)	Explaining the effects of symptom attribution by carers on help-seeking for individuals living with dementia	-EXCLUDE on population (no housing precarity)	
####	Felix (2020) (ID:87853278)	PERCEPTION OF SHELTER UNIT PROFESSIONALS ON SEXUAL AND REPRODUCTIVE HEALTH OF ADOLESCENT INSTITUTIONALIZED	-EXCLUDE on country (High-Income)	
####	Felix (2022) (ID:87851444)	A 5-year multidisciplinary care outcomes in children with wilms' tumour managed at a tertiary centre: A retrospective observational study	-EXCLUDE on population (people aged under 18 years)	
####	Felner (2018) (ID:87850821)	Understanding aging-out of LGBTQ services in Chicago: A youth participatory action research project.	-EXCLUDE on population (people aged under 18 years)<br>-EXCLUDE - but review for literature	
####	Felner (2022) (ID:87850683)	Participatory action research to address aging out of LGBTQ-supportive youth programs in Chicago.	-EXCLUDE on intervention (service engagement/helpseeking behaviour)	
####	Felsher (2021) (ID:87852737)	Predictors of Willingness to Diffuse PrEP Information within Ego-Centric Networks of Women Who Inject Drugs	-EXCLUDE on intervention (service engagement/helpseeking behaviour)	
####	Felsman (2016) (ID:87848707)	Supporting Health and Well-Being for Resettled Refugee Women: The Global Women's Group.	-EXCLUDE on population (no housing precarity)	
####	Felt (2023) (ID:87851665)	Instability in Housing and Medical Care Access: The Inequitable Impacts of the COVID-19 Pandemic on U.S. Transgender Populations.	-EXCLUDE on intervention (service engagement/helpseeking behaviour)	
####	Ferbežar (2023) (ID:87858035)	REZILIENTNOST LGBTQ+ MLADIH V OKVIRU VZGOJE IN IZOBRAŽEVANJA: INTEGRATIVNI PREGLED LITERATURE	-EXCLUDE on evidence and form (evidence not in written form or presented as research output)	
####	Ferguson (2011) (ID:87849181)	Correlates of street-survival behaviors in homeless young adults in four U.S. cities.	-EXCLUDE on population (women)	
####	FERGUSON (2011) (ID:87851440)	Improving engagement in the role of father for homeless, noncustodial fathers: a program evaluation	-EXCLUDE on population (women)	
####	Ferguson (2012) (ID:87849144)	Merging the fields of mental health and social enterprise: lessons from abroad and cumulative findings from research with homeless youths.	-EXCLUDE on intervention (service engagement/helpseeking behaviour)	
####	Ferguson (2016) (ID:87852660)	A Call to Action: A Blueprint for Academic Health Sciences in the Era of Mass Incarceration	-EXCLUDE on population (no housing precarity)	
####	FERGUSON (2016) (ID:87857255)	Social capital and help-seeking behavior among urban, minority parents participating in the CONNECT Program: the role of informal community supports	-EXCLUDE on population (no housing precarity)	
####	Ferguson (2022) (ID:87850621)	Health and healthcare service use: The experiences of runaway trans adolescents compared to their peers.	-EXCLUDE on population (people aged under 18 years)	
####	Ferguson (2023) (ID:87857994)	Examining the Connection Between Missing Persons and Victimization: An Application of Lifestyle Exposure Theory	-EXCLUDE on population (women)	
####	FERNET (2019) (ID:87857331)	Meeting the needs of adolescent and emerging adult victims of sexual violence in their romantic relationships: a mixed methods study exploring barriers to help-seeking	-EXCLUDE on population (people aged under 18 years)	
####	Ferrari (2018) (ID:87848489)	Psychological advocacy towards healing (PATH): A randomized controlled trial of a psychological intervention in a domestic violence service setting.	-EXCLUDE on intervention (service engagement/helpseeking behaviour)	
####	Ferreira (2021) (ID:87857464)	Migrant Women Victims of Intimate Partner Violence and the Criminal Justice System in Portugal	-INCLUDE on title & abstract	-EXCLUDE (IPV/DVA but little discussion on housing)
####	Ferrendelli (2020) (ID:87858126)	Empowering LGBTQ Youth through Mobile Technology	-EXCLUDE on population (no housing precarity)	
####	Festa (2020) (ID:87853314)	Breast Cancer Treatment Delays at an Urban Safety Net Hospital Among Women Experiencing Homelessness	-EXCLUDE on intervention (service engagement/helpseeking behaviour)	
####	Fiani (2018) (ID:87851928)	Beyond the Binary: Gender Identity and Mental Health among Transgender and Gender Non-Conforming Adults	-EXCLUDE on population (no housing precarity)	

####	Fiani (2019) (ID:87850768)	Beyond the binary: Gender identity and mental health among transgender and gender non-conforming adults.	-EXCLUDE on intervention (service engagement/helpseeking behaviour)	
####	Fiedler (2021) (ID:87857706)	'I played by all the rules! Why didn't you tell me there weren't any rules, it's not fair!':contradiction, corporeality, and conformity in Grace and Frankie1	-EXCLUDE on population (no housing precarity)	
####	Fielding-Miller (2022) (ID:87857738)	“We’re already doing this work”: ethical research with community-based organizations	-EXCLUDE on population (no housing precarity)	
####	Filia (2022) (ID:87963375)	Social inclusion, intersectionality, and profiles of vulnerable groups of young people seeking mental health support	-EXCLUDE on population (no housing precarity)	
####	Fillion (2011) (ID:87852039)	(help?)	-EXCLUDE on evidence and form (evidence not in written form or presented as research output)	
####	Fine (2019) (ID:87857507)	Critical Participatory Action Research: A Feminist Project for Validity and Solidarity	-EXCLUDE on population (no housing precarity)	
####	Fine (2021) (ID:87848216)	Office-Based Addiction Treatment Retention and Mortality Among People Experiencing Homelessness.	-EXCLUDE on population (women)	
####	Fine (2021) (ID:87963241)	Critical participatory action research: Methods and praxis for intersectional knowledge production.	-EXCLUDE on intervention (service engagement/helpseeking behaviour)	
####	Fine (2022) (ID:87848170)	Drug Overdose Mortality Among People Experiencing Homelessness, 2003 to 2018.	-EXCLUDE on population (women)	
####	Fine (2023) (ID:87848131)	Health Care Utilization among Homeless-Experienced Adults Who Were Seen by a Mobile Addiction Health Clinic in Boston, Massachusetts: A Quasi-Experimental Study.	-EXCLUDE on population (women)	
####	Finfgeld-Connett (2010) (ID:87849256)	Becoming homeless, being homeless, and resolving homelessness among women.	-INCLUDE on title & abstract	-EXCLUDE on intervention (intersectionality)
####	FINKELHOR (2001) (ID:87857280)	Police reporting and professional help seeking for child crime victims: a review	-EXCLUDE on date (2010)	
####	Finlay (2021) (ID:87848246)	A Critical Approach to Aging in Place: A Case Study Comparison of Personal and Professional Perspectives from the Minneapolis Metropolitan Area.	-EXCLUDE on population (no gender focus; women population <50)	
####	Finn (2011) (ID:87851772)	Looking at the Whole Defendant.	-EXCLUDE on evidence and form (evidence not in written form or presented as research output)	
####	Finnie (2022) (ID:87848144)	Tenant-Based Housing Voucher Programs: A Community Guide Systematic Review.	-EXCLUDE on intervention (service engagement/helpseeking behaviour)	
####	Fiola (2019) (ID:87857848)	TWO-SPIRIT AND QUEER TRANS PEOPLE OF COLOUR: REFLECTING ON THE CALL TO CONVERSATION CONFERENCE (C2C)	-EXCLUDE on population (no housing precarity)	
####	Fiorati (2017) (ID:87848621)	Social ruptures and the everyday life of homeless people: an ethnographic study.	-EXCLUDE on country (High-Income)	
####	Fiore (2017) (ID:87853070)	Underserved populations and bacterial and protozoal sexually transmitted infections: a lost health-care opportunity	-EXCLUDE on population (women)	
####	Fischer (2016) (ID:87858100)	Contingent belonging: Chelsea Manning, transpatriotism, and iterations of empire	-EXCLUDE on population (no housing precarity)	
####	Fisher (2011) (ID:87849192)	Evaluation of an HIV prevention intervention for African Americans and Hispanics: findings from the VOICES/VOCES Community-based Organization Behavioral Outcomes Project.	-EXCLUDE on population (women)	
####	Fisher (2012) (ID:87851217)	Improving emotional and behavioral outcomes for LGBT youth: A guide for professionals.	-EXCLUDE on population (women)	
####	Fisher (2017) (ID:87848675)	Failure to get into substance abuse treatment.	-EXCLUDE on population (no gender focus; women population <50)	
####	Fisk (2013) (ID:87851155)	Vulnerability and health care service use among homeless persons with psychiatric and co-occurring disorders.	-EXCLUDE on population (no gender focus; women population <50)<br>-EXCLUDE - but review for literature	
####	FISSEL (2021) (ID:87857035)	The reporting and help-seeking behaviors of cyberstalking victims	-EXCLUDE on population (no housing precarity)	
####	Fitsch (2020) (ID:87857990)	Coalition-Making and the Practice of Feminist STS in the time of COVID-19	-EXCLUDE on intervention (service engagement/helpseeking behaviour)	
####	Fitzpatrick (2015) (ID:87851038)	Does context matter? Examining the mental health among homeless people.	-EXCLUDE on population (women)	

####	Fitzpatrick (2017) (ID:87850903)	How positive is their future? Assessing the role of optimism and social support in understanding mental health symptomatology among homeless adults.	-EXCLUDE on population (women)	
####	Fledderjohann (2014) (ID:87853124)	Do Girls Have a Nutritional Disadvantage Compared with Boys? Statistical Models of Breastfeeding and Food Consumption Inequalities among Indian Siblings	-EXCLUDE on country (High-Income)	
####	Fleming (2020) (ID:87851984)	Stimulant safe supply: a potential opportunity to respond to the overdose epidemic	-EXCLUDE on population (women)	
####	Fletcher (2014) (ID:87848906)	Housing status and HIV risk behaviors among transgender women in Los Angeles.	-EXCLUDE on intervention (service engagement/helpseeking behaviour)	
####	Fletcher (2022) (ID:87857732)	Culturally Responsive Strategies of a High School STEAM Themed Academy to Engage African American Males	-EXCLUDE on population (women)	
####	Fleury (2013) (ID:87852696)	Typology of persons with severe mental disorders	-EXCLUDE on population (no housing precarity)	
####	Fleury (2023) (ID:87852687)	Profiles of quality of outpatient care use, associated sociodemographic and clinical characteristics, and adverse outcomes among patients with substance-related disorders	-EXCLUDE on intervention (service engagement/helpseeking behaviour)	
####	FLIC and Solace. Housing... (Single) (ID:88019156)	FLIC and Solace. Housing First pilot for homeless women experiencing domestic abuse and multiple disadvantage (2019)	-INCLUDE on title & abstract	-EXCLUDE on intervention (intersectionality)
####	Flicker (2011) (ID:87963368)	Concomitant forms of abuse and help-seeking behavior among White, African American, and Latina women who experience intimate partner violence	-INCLUDE on title & abstract	-EXCLUDE (IPV/DVA but little discussion on housing)
####	Flike (2022) (ID:87853126)	Homelessness and Vulnerably-Housed Defined: A Synthesis of the Literature	-EXCLUDE on intervention (service engagement/helpseeking behaviour)	
####	Flike (2023) (ID:88019141)	Systematic review of access to healthcare and social services among US women Veterans experiencing homelessness	-EXCLUDE - but review for literature<br>-EXCLUDE on intervention (service engagement/helpseeking behaviour)	
####	Flocks (2016) (ID:87963187)	The case for trauma-informed, gender-specific prevention/early intervention programming in reducing female juvenile delinquency in Florida	-EXCLUDE on intervention (service engagement/helpseeking behaviour)	
####	Flora (2012) (ID:87849110)	Pathways to forensic mental health care in Toronto: a comparison of European, African-Caribbean, and other ethnoracial groups in Toronto.	-EXCLUDE on population (no housing precarity)	
####	Flora (2020) (ID:87851906)	A qualitative investigation of lived experiences of long-term health condition management with people who are food insecure	-EXCLUDE on population (women)	
####	Flynn (2018) (ID:87963045)	When structural violences create a context that facilitates sexual assault and intimate partner violence against street-involved young women	-EXCLUDE on intervention (service engagement/helpseeking behaviour)<br>-EXCLUDE - but review for literature	
####	Flynn (2023) (ID:87963062)	How social responses to child sexual abuse and intimate partner violence affect homelessness among women in two rural regions with resource-based economies in eastern Quebec	-EXCLUDE on intervention (service engagement/helpseeking behaviour)	
####	fodders (2018) (ID:88019124)	Better Births for Women with Multiple Disadvantages - Revolving Doors	-EXCLUDE Duplicate	
####	Foley (2013) (ID:87849061)	Functioning and post-school transition outcomes for young people with Down syndrome.	-EXCLUDE on intervention (service engagement/helpseeking behaviour)	
####	Foley (2014) (ID:87851044)	'Their families or the disability services will take care of them': The invisible homeless and how Irish government policy is designed not to help them.	-EXCLUDE on intervention (service engagement/helpseeking behaviour)	
####	Fond (2019) (ID:87848471)	Prescription of potentially inappropriate psychotropic drugs in homeless people with schizophrenia and bipolar disorders. Results from the French Housing First (FHF) program.	-EXCLUDE on population (women)	
####	Fontenot (2016) (ID:87848711)	The Association of Health Seeking Behaviors With Human Papillomavirus Vaccination Status Among High-Risk Urban Youth.	-EXCLUDE on intervention (service engagement/helpseeking behaviour)	
####	Foote (2019) (ID:87853222)	Gender Disparity in Referral for Definitive Care of Malignant Pleural Effusions	-EXCLUDE on population (no housing precarity)	

####	Forchuk (2013) (ID:87849053)	Preventing homelessness after discharge from psychiatric wards: perspectives of consumers and staff.	-EXCLUDE on population (women)	
####	Ford (2011) (ID:87858031)	Political Socialization and Citizenship Education for Queer Youth	-EXCLUDE on population (no housing precarity)	
####	Ford (2012) (ID:87853208)	Ethnoracial and Educational Differences in Victimization History, Trauma-Related Symptoms, and Coping Style	-INCLUDE on title & abstract	-EXCLUDE on target group (no housing precarity)
####	Ford (2013) (ID:87851163)	Intimate partner violence prevention services and resources in Los Angeles: Issues, needs, and challenges for assisting lesbian, gay, bisexual, and transgender clients.	-INCLUDE on title & abstract	-EXCLUDE (IPV/DVA but little discussion on housing)
####	Ford (2019) (ID:87857741)	TransConnect: A Mixed Methods Study of Pathways to Health for Black and Latinx Transgender Women Who Have Survived Violence	-EXCLUDE on population (no housing precarity)	
####	Ford (2021) (ID:87857631)	Experiences of African American Women with Microaggression While Employed in a Human Services Organization	-EXCLUDE on population (no housing precarity)	
####	Fordham (2015) (ID:87848806)	The lived experience of homeless women: insights gained as a specialist practitioner.	-INCLUDE on title & abstract	-EXCLUDE on evidence and form (not empirical)
####	Forecasting life expectancy,... (Foreman) (ID:87851461)	Forecasting life expectancy, years of life lost, and all-cause and cause-specific mortality for 250 causes of death: reference and alternative scenarios for 2016–40 for 195 countries and territories	-EXCLUDE on intervention (service engagement/helpseeking behaviour)	
####	Foreman-Mackey (2019) (ID:87848383)	'It's our safe sanctuary': Experiences of using an unsanctioned overdose prevention site in Toronto, Ontario.	-EXCLUDE on intervention (service engagement/helpseeking behaviour)	
####	Forenza (2017) (ID:87848646)	An Exploratory Analysis of Unhealthy and Abusive Relationships for Adults with Serious Mental Illnesses Living in Supportive Housing.	-EXCLUDE on intervention (service engagement/helpseeking behaviour)	
####	Forge (2013) (ID:87851152)	A longitudinal investigation of risk and resiliency among homeless LGBT youth residing in a transitional living shelter.	-EXCLUDE on intervention (service engagement/helpseeking behaviour)	
####	Fortin (2015) (ID:87848889)	I WAS HERE: young mothers who have experienced homelessness use Photovoice and participatory qualitative analysis to demonstrate strengths and assets.	-EXCLUDE on intervention (service engagement/helpseeking behaviour)<br>-EXCLUDE - but review for literature	
####	Fossion (2014) (ID:87848911)	Psychological and socio-demographic data contributing to the resilience of holocaust survivors.	-EXCLUDE on intervention (service engagement/helpseeking behaviour)	
####	Foster (2011) (ID:87849175)	Personal disaster preparedness of dialysis patients in North Carolina.	-EXCLUDE on intervention (service engagement/helpseeking behaviour)	
####	Foster (2012) (ID:87851259)	Homelessness in schizophrenia.	-EXCLUDE on intervention (service engagement/helpseeking behaviour)	
####	Fouad (2012) (ID:87851211)	APA handbook of counseling psychology, Vol. 1: Theories, research, and methods.	-EXCLUDE on intervention (service engagement/helpseeking behaviour)	
####	Fountoulakis (2012) (ID:87851243)	Development of the Global Disability Scale (Glo.Di.S): Preliminary results.	-EXCLUDE on intervention (service engagement/helpseeking behaviour)	
####	FOX (2001) (ID:87857402)	Barriers to help seeking for mental disorder in a rural impoverished population	-EXCLUDE on date (2010)	
####	Fox (2018) (ID:87963052)	"They said if you come you can't drink. I thought, I can't stop." Exploring the journeys to support among women who experience co-occurring substance use and domestic abuse.	-INCLUDE on title & abstract	-EXCLUDE on intervention (intersectionality)
####	Fraguada (2020) (ID:87850713)	The association between behavior disorders and return visit to the emergency department.	-EXCLUDE on intervention (service engagement/helpseeking behaviour)	
####	Francia (2023) (ID:87857636)	Putting out the welcome mat—A qualitative exploration of service delivery processes and procedures as barriers to treatment-seeking for people who use alcohol and other drugs	-EXCLUDE on population (no housing precarity)	
####	FRANCIS-KELLY (2022) (ID:87857583)	The Wheels on the Bus: The Tourism Industry Development Council and Envisioning Spatial Futures in post-Rodney King Los Angeles	-EXCLUDE on evidence and form (evidence not in written form or presented as research output)	
####	Frasca (2019) (ID:87853275)	A Mixed Methods Evaluation of an Inclusive Sexual History Taking and HIV Prevention Curriculum for Trainees	-EXCLUDE on intervention (service engagement/helpseeking behaviour)	
####	Fraser (2019) (ID:87857413)	LGBTIQ+ homelessness: A review of the literature	-INCLUDE on title & abstract	-EXCLUDE on target group (youth)<br>-EXCLUDE (systematic review)* only use with other code

####	Freeman (2017) (ID:87857434)	Critical race theory as a tool for understanding poor engagement along the HIV care continuum among African American/Black and Hispanic persons living with HIV in the United States: A qualitative exploration	-EXCLUDE on population (no housing precarity)	
####	Freeman (2021) (ID:87857701)	Prevention in Context: An Examination of Factors Associated with Recent HIV Testing Among Men in New York City	-EXCLUDE on population (women)	
####	Fregoso (2015) (ID:87858067)	Through the pipeline: Degree aspirations of African American and Latino males enrolled in California community colleges	-EXCLUDE on population (women)	
####	Freguja (2017) (ID:87852763)	Between deprivation and homelessness: the poverty of women in today's Italy	-INCLUDE on title & abstract	-EXCLUDE on evidence and form (evidence not in written form or presented as research output)
####	Fries (2014) (ID:87851049)	Role of gender, substance use, and serious mental illness in anticipated postjail homelessness.	-INCLUDE on title & abstract	-EXCLUDE on intervention (service engagement/helpseeking behaviour)
####	Frimpong (2015) (ID:87848830)	Correlates of HIV testing and receipt of test results in addiction health services in Los Angeles County.	-EXCLUDE on intervention (service engagement/helpseeking behaviour)	
####	Frisby (2019) (ID:87851994)	An Examination of the Usage of a Psychiatric Readmit Index Tool in Reducing Psychiatric Hospital Recidivism	-EXCLUDE on intervention (service engagement/helpseeking behaviour)	
####	Frisman (2017) (ID:87848611)	Evaluation of CT's ASIST program: Specialized services to divert higher risk defendants.	-EXCLUDE on intervention (service engagement/helpseeking behaviour)	
####	Frisone (2022) (ID:87851791)	Trends in incidence and mortality of lung cancer in Switzerland: Possible explanations and open questions.	-EXCLUDE on intervention (service engagement/helpseeking behaviour)	
####	Frongillo (2017) (ID:87848601)	Food Insecurity Is Associated with Subjective Well-Being among Individuals from 138 Countries in the 2014 Gallup World Poll.	-EXCLUDE on intervention (service engagement/helpseeking behaviour)	
####	Frost (2020) (ID:87857626)	The Qualitative Interview in Psychology and the Study of Social Change: Sexual Identity Development, Minority Stress, and Health in the Generations Study	-EXCLUDE on population (no housing precarity)	
####	Fu (2013) (ID:87849008)	Understanding the revolving door: individual and structural-level predictors of recidivism among individuals with HIV leaving jail.	-EXCLUDE on intervention (service engagement/helpseeking behaviour)	
####	Fulder-Heyd (2010) (ID:87850527)	Women's experience of psychological homelessness and identity management : a portfolio of research and therapeutic practice	-EXCLUDE on intervention (service engagement/helpseeking behaviour)	
####	Fulginiti (2016) (ID:87848758)	Risky Integration.	-EXCLUDE on intervention (service engagement/helpseeking behaviour)	
####	Fulginiti (2016) (ID:87850948)	Risky integration: A social network analysis of network position, exposure, and suicidal ideation among homeless youth.	-EXCLUDE on intervention (service engagement/helpseeking behaviour)	
####	Fulginiti (2021) (ID:87857709)	Sexual Minority Stress, Mental Health Symptoms, and Suicidality among LGBTQ Youth Accessing Crisis Services	-EXCLUDE on population (no housing precarity)	
####	FULLER (2016) (ID:87851427)	The impact of the Health and Social Care Act, 2012 on the health and wellbeing of rough sleepers	-EXCLUDE on population (no gender focus; women population <50)	
####	Funcionalidad familiar,... (Barreto-Zorza) (ID:87851659)	Funcionalidad familiar, una percepción de madres e hijos escolares: programa de atención primaria, Guapí-Cauca, Colombia.	-EXCLUDE on country (High-Income)	
####	Funk (2012) (ID:87851956)	Mental health, poverty and development	-EXCLUDE on intervention (service engagement/helpseeking behaviour)	
####	Funstan (2019) (ID:87963194)	In the business of trauma: an intersectional-materialist feminist analysis of'trauma informed'women's refuges and crisis accommodation services in Sydney and Vancouver	-INCLUDE on title & abstract	-EXCLUDE on intervention (service engagement/helpseeking behaviour)
####	Furl (2018) (ID:87848548)	Determinants of facilitated health insurance enrollment for patients with HIV disease, and impact of insurance enrollment on targeted health outcomes.	-EXCLUDE on intervention (service engagement/helpseeking behaviour)	
####	Furlotte (2017) (ID:87857740)	Mental Health Experiences of Older Adults Living with HIV: Uncertainty, Stigma, and Approaches to Resilience	-EXCLUDE on population (no housing precarity)	
####	Furman (2017) (ID:87850854)	"It's a gap in awareness": Exploring service provision for LGBTQ2S survivors of intimate partner violence in Ontario, Canada.	-INCLUDE on title & abstract	-EXCLUDE (IPV/DVA but little discussion on housing)
####	FURSLAND (2008) (ID:87857065)	Coming to the UK: a guide for children and young people seeking asylum	-EXCLUDE on date (2010)	

####	FURSLAND (2020) (ID:87857047)	Caring for unaccompanied asylum seeking children and young people: Eritrea	-EXCLUDE on country (High-Income)	
####	Fuster (2019) (ID:87848366)	Community Screening, Identification, and Referral to Primary Care, for Hepatitis C, B, and HIV Among Homeless Persons in Los Angeles.	-EXCLUDE on intervention (service engagement/helpseeking behaviour)	
####	Gabbidon (2022) (ID:87857556)	Intersectional stigma and developmental competence among youth living with HIV	-EXCLUDE on population (women)	
####	Gaber (2022) (ID:87853274)	A comparison of perceptions of caring behaviours among women in homelessness, Registered Nurses and nursing students	-EXCLUDE on intervention (service engagement/helpseeking behaviour)	
####	Gabrielian (2014) (ID:87848943)	VA health service utilization for homeless and low-income Veterans: a spotlight on the VA Supportive Housing (VASH) program in greater Los Angeles.	-EXCLUDE on population (women)	
####	Gabrielian (2016) (ID:87848791)	Factors Associated With Premature Exits From Supported Housing.	-EXCLUDE on intervention (service engagement/helpseeking behaviour)	
####	Gabrielian (2017) (ID:87848644)	"They're homeless in a home": Retaining homeless-experienced consumers in supported housing.	-EXCLUDE on population (women)	
####	Gabrielian (2018) (ID:87848599)	Social support and housing transitions among homeless adults with serious mental illness and substance use disorders.	-EXCLUDE on population (women)	
####	Gaddis (2018) (ID:87848503)	Regular MDMA use is associated with decreased risk of drug injection among street-involved youth who use illicit drugs.	-EXCLUDE on intervention (service engagement/helpseeking behaviour)	
####	Gadermann (2020) (ID:87848320)	The Association of Residential Instability and Hospitalizations among Homeless and Vulnerably Housed Individuals: Results from a Prospective Cohort Study.	-EXCLUDE on intervention (service engagement/helpseeking behaviour)	
####	Gadermann (2021) (ID:87850628)	Understanding subjective quality of life in homeless and vulnerably housed individuals: The role of housing, health, substance use, and social support.	-EXCLUDE on population (women)	
####	Gaeta (2023) (ID:88019150)	A National Study of Homelessness, Social Determinants of Health, and Treatment Engagement Among Outpatient Medication for Opioid Use Disorder-Seeking Individuals in the United States	-EXCLUDE on population (women)	
####	Gaetz (2017) (ID:87963219)	A new direction: A framework for homelessness prevention	-EXCLUDE on population (women)	
####	Gait Speed and Dismobility... (Ostir) (ID:87851462)	Gait Speed and Dismobility in Older Adults	-EXCLUDE on intervention (service engagement/helpseeking behaviour)	
####	Galanter (2014) (ID:87848918)	Narcotics anonymous: a comparison of military veterans and non-veterans.	-EXCLUDE on population (no housing precarity)	
####	Gallardo (2020) (ID:87848300)	Human Papillomavirus Vaccination Initiation and Completion among Youth Experiencing Homelessness in Seven U.S. Cities.	-EXCLUDE on population (no gender focus; women population <50)	
####	Gama (2014) (ID:87848951)	Use of medicines by homeless people in Porto, Portugal.	-EXCLUDE on intervention (service engagement/helpseeking behaviour)	
####	Gandy-Guedes (2019) (ID:87857744)	Activism in Southwestern Queer and Trans Young Adults After the Marriage Equality Era	-EXCLUDE on intervention (service engagement/helpseeking behaviour)	
####	Gao (2023) (ID:87851466)	Industry, occupation, and exposure history of mesothelioma patients in the U.S. National Mesothelioma Virtual Bank, 2006–2022.	-EXCLUDE on intervention (service engagement/helpseeking behaviour)	
####	Garakani (2020) (ID:87853186)	Retrospective Chart Review of Voluntary Admissions to an Inpatient Psychiatric Hospital in New York City: A Demographic Breakdown	-EXCLUDE on intervention (service engagement/helpseeking behaviour)	
####	Garcia (2010) (ID:87853019)	Conceptualization and Measurement of Coping During Adolescence: A Review of the Literature	-EXCLUDE on population (people aged under 18 years)	
####	Garcia (2020) (ID:87848285)	"I Felt Safe": The Role of the Rapid Rehousing Program in Supporting the Security of Families Experiencing Homelessness in Salt Lake County, Utah.	-EXCLUDE on intervention (service engagement/helpseeking behaviour)	
####	Garcia (2021) (ID:87857580)	A Transcendental Phenomenology of Homeless Parenting Youth in a Clackamas County Rehousing Program: Examining Their Lived Experiences and Educational Prospects	-INCLUDE on title & abstract	-EXCLUDE on intervention (intersectionality)
####	Garcia-Perez (2020) (ID:87857566)	Lesbian, gay, bisexual, transgender, queer + Latinx youth mental health disparities: A systematic review	-EXCLUDE on population (people aged under 18 years)	

####	Gardy (2023) (ID:87853378)	Cancer Survival and Travel Time to Nearest Reference Care Center for 10 Cancer Sites: An Analysis of 21 French Cancer Registries	-EXCLUDE on population (no housing precarity)	
####	Garey (2015) (ID:87848868)	Subjective social status and readiness to quit among homeless smokers.	-EXCLUDE on intervention (service engagement/helpseeking behaviour)	
####	Garey (2019) (ID:87848478)	Health-Related Quality of Life Among Homeless Smokers: Risk and Protective Factors of Latent Class Membership.	-EXCLUDE on intervention (service engagement/helpseeking behaviour)	
####	Garg (2015) (ID:87848869)	Addressing social determinants of health at well child care visits: a cluster RCT.	-EXCLUDE on population (no housing precarity)	
####	Garnweidner-Holme (2017) (ID:87848648)	Talking about intimate partner violence in multi-cultural antenatal care: a qualitative study of pregnant women's advice for better communication in South-East Norway.	-EXCLUDE on intervention (service engagement/helpseeking behaviour)	
####	Garre-Olmo (2019) (ID:87851535)	Survival, effect measures, and impact numbers after dementia diagnosis: a matched cohort study	-EXCLUDE on population (no housing precarity)	
####	Garrett (2020) (ID:87848263)	Health system resource use among populations with complex social and behavioral needs in an urban, safety-net health system.	-EXCLUDE on population (women)	
####	Gashaw (2021) (ID:87848194)	Expected Impacts of COVID-19: Considering Resource-Limited Countries and Vulnerable Population.	-EXCLUDE on intervention (service engagement/helpseeking behaviour)	
####	Gawron (2017) (ID:87849357)	Long-acting Reversible Contraception Among Homeless Women Veterans With Chronic Health Conditions	-INCLUDE on title & abstract	-EXCLUDE on intervention (service engagement/helpseeking behaviour)
####	Gaynor (2022) (ID:87857561)	Predatory Policing, Intersectional Subjection, and the Experiences of LGBTQ People of Color in New Orleans	-EXCLUDE on intervention (service engagement/helpseeking behaviour)	
####	Gazzola (2022) (ID:87853441)	A cohort study examining the relationship among housing status, patient characteristics, and retention among individuals enrolled in low-barrier-to-treatment-access methadone maintenance treatment	-EXCLUDE on population (women)	
####	Gebreyesus (2019) (ID:87848408)	Experiences of homeless women on maternity health service utilization and associated challenge in Aksum town, Northern Ethiopia.	-EXCLUDE on country (High-Income)	
####	Gebrezgi (2020) (ID:87848306)	Development and Validation of a Risk Prediction Tool to Identify People with HIV Infection Likely Not to Achieve Viral Suppression.	-EXCLUDE on intervention (service engagement/helpseeking behaviour)	
####	Geiger (2022) (ID:87963316)	“I think there’sa lot of intersectionality”: The college experiences of immigrant-origin Latinx young adults with a foster care background.	-EXCLUDE on population (no housing precarity)	
####	Gender differences in... (Shuter) (ID:87849369)	Gender differences in HIV risk behaviors in an adult emergency department in New York City	-EXCLUDE on intervention (service engagement/helpseeking behaviour)	
####	Gennaro (2021) (ID:87848198)	Parental Beliefs, Logistical Challenges, and Improvement Opportunities for Vaccination among Children Ages 19-35 Months Experiencing Homelessness.	-EXCLUDE on population (people aged under 18 years)	
####	Gentil (2020) (ID:87848355)	Satisfaction with health and community services among homeless and formerly homeless individuals in Quebec, Canada.	-EXCLUDE on intervention (service engagement/helpseeking behaviour)	
####	George (2013) (ID:87849079)	A qualitative exploration of the role of social networks in educating urban African American adolescents about sex.	-EXCLUDE on population (no housing precarity)	
####	George (2016) (ID:87848737)	Our Health Is in Our Hands: A Social Marketing Campaign to Combat Obesity and Diabetes.	-EXCLUDE on population (no housing precarity)	
####	George (2021) (ID:87857889)	Pilot Study Examining the Impact of Acculturation on Refugees' Healthcare Satisfaction	-EXCLUDE on intervention (service engagement/helpseeking behaviour)	
####	Gerardin (2017) (ID:87852888)	Evaluation of problematic psychoactive substances use in people placed in police custody	-EXCLUDE on intervention (service engagement/helpseeking behaviour)	
####	Gerassi (2018) (ID:87853238)	Barriers to Accessing Detox Facilities, Substance Use Treatment, and Residential Services among Women Impacted by Commercial Sexual Exploitation and Trafficking	-INCLUDE on title & abstract	-EXCLUDE on intervention (intersectionality)
####	Gerassi (2019) (ID:87857755)	Addressing race, racism, and commercial sexual exploitation in practice through an action-based research partnership	-EXCLUDE on intervention (service engagement/helpseeking behaviour)	
####	Gerassi (2019) (ID:87963188)	How sex trading identities shape experiences of service provision: Insights from adult women with lived experiences and service providers	-INCLUDE on title & abstract	-EXCLUDE on intervention (service engagement/helpseeking behaviour)

####	Gerassi (2020) (ID:87963049)	An intersectional content analysis of inclusive language and imagery among sex trafficking-related services	-INCLUDE on title & abstract	-EXCLUDE on intervention (service engagement/helpseeking behaviour)
####	Gerassi (2021) (ID:87963182)	Disclosing sex trading histories to providers: Barriers and facilitators to navigation of social services among women impacted by commercial sexual exploitation	-INCLUDE on title & abstract	-EXCLUDE on intervention (service engagement/helpseeking behaviour)
####	German (2012) (ID:87853006)	Social Stability and Health: Exploring Multidimensional Social Disadvantage	-EXCLUDE on intervention (service engagement/helpseeking behaviour)	
####	Ghabra (2018) (ID:87857547)	From failure and allyship to feminist solidarities: negotiating our privileges and oppressions across borders	-EXCLUDE on population (no housing precarity)	
####	Ghazi (2021) (ID:87848202)	Neighborhood Socioeconomic Status and Identification of Patients With CKD Using Electronic Health Records.	-EXCLUDE on population (no housing precarity)	
####	Ghose (2013) (ID:87849047)	Hazardous drinking and its association with homelessness among veterans in care.	-EXCLUDE on population (women)	
####	Ghose (2019) (ID:87848412)	Effectiveness of a Viral Load Suppression Intervention for Highly Vulnerable People Living with HIV.	-EXCLUDE on intervention (service engagement/helpseeking behaviour)	
####	Ghose (2019) (ID:87851914)	"It's my Room and my Life": Housing's Influence on Medication Adherence for HIV-positive Women Released from Incarceration	-EXCLUDE on intervention (service engagement/helpseeking behaviour)	
####	Ghoshal (2023) (ID:88019148)	Factors associating with help-seeking by women facing Intimate Partner Violence in India: insights from National Family Health Survey-5	-EXCLUDE on country (High-Income)<br>-EXCLUDE - but review for literature	
####	Ghosn (2017) (ID:87853043)	Associations of cause-specific mortality with area level deprivation and travel time to health care in France from 1990 to 2007, a multilevel analysis	-EXCLUDE on population (no housing precarity)	
####	Giannakeas (2022) (ID:87853194)	Analysis of Platelet Count and New Cancer Diagnosis Over a 10-Year Period	-EXCLUDE on intervention (service engagement/helpseeking behaviour)	
####	Giano (2020) (ID:87848338)	Forty Years of Research on Predictors of Homelessness.	-EXCLUDE on population (women)<br>-EXCLUDE - but review for literature	
####	Gibb (2020) (ID:87857640)	Sexual and gender minority health vulnerabilities during the COVID-19 health crisis	-EXCLUDE on population (no housing precarity)	
####	Gibbs (2015) (ID:87851007)	Services to domestic minor victims of sex trafficking: Opportunities for engagement and support.	-EXCLUDE on population (people aged under 18 years)	
####	Gibbs (2023) (ID:87857988)	Inclusion of Cultural and Linguistic Diversity in COVID-19 Public Health Research: Research Design Adaptations to Seek Different Perspectives in Victoria, Australia	-EXCLUDE on population (no housing precarity)	
####	Giesbrecht (2023) (ID:87963218)	Supporting newcomer women who experience intimate partner violence and their children: Insights from service providers	-INCLUDE on title & abstract	-EXCLUDE (IPV/DVA but little discussion on housing)
####	GILBERT (2011) (ID:87857087)	Service utilization patterns as predictors of response to trauma-informed integrated treatment for women with co-occurring disorders	-EXCLUDE on intervention (service engagement/helpseeking behaviour)	
####	GILCHRIST (2006) (ID:87857318)	Barriers to help-seeking in young people: community beliefs about youth suicide	-EXCLUDE on date (2010)	
####	Gilchrist (2017) (ID:87848602)	Preventing blood-borne virus infection in people who inject drugs in the UK: systematic review, stakeholder interviews, psychosocial intervention development and feasibility randomised controlled trial.	-EXCLUDE on population (no housing precarity)	
####	Gilchrist (2017) (ID:87848659)	The acceptability and feasibility of a brief psychosocial intervention to reduce blood-borne virus risk behaviours among people who inject drugs: a randomised control feasibility trial of a psychosocial intervention (the PROTECT study) versus treatment as usual.	-EXCLUDE on intervention (service engagement/helpseeking behaviour)	
####	GILL (2008) (ID:87857056)	'A specialist refuge space of my own': black, minority ethnic and refugee women, housing and domestic violence	-EXCLUDE on date (2010)	
####	Gillig (2022) (ID:87858096)	Virtual Camp: LGBTQ Youths' Collective Coping During the COVID-19 Pandemic	-EXCLUDE on population (no housing precarity)	
####	GILLON (2007) (ID:87857131)	Gender differences in help seeking	-EXCLUDE on date (2010)	
####	Gillum (2021) (ID:87963067)	African American survivors of intimate partner violence: Lived experience and future directions for research	-EXCLUDE on intervention (service engagement/helpseeking behaviour)	

####	Gilmer (2010) (ID:87849253)	Effect of full-service partnerships on homelessness, use and costs of mental health services, and quality of life among adults with serious mental illness.	-EXCLUDE on intervention (service engagement/helpseeking behaviour)	
####	Gilmer (2014) (ID:87851055)	Fidelity to the Housing First model and effectiveness of permanent supported housing programs in California.	-EXCLUDE on intervention (service engagement/helpseeking behaviour)	
####	Gilmer (2021) (ID:87851895)	Developing trauma resilient communities through community capacity-building	-EXCLUDE on population (no housing precarity)	
####	GILROY (2001) (ID:87857277)	Away from home: alone	-EXCLUDE on date (2010)	
####	Gilroy (2015) (ID:87848888)	Predictors and outcomes of community agency use in abused mothers.	-EXCLUDE on intervention (service engagement/helpseeking behaviour)	
####	Ginn (2020) (ID:87848325)	Engaging From Both Sides: Facilitating a Canadian Two-Generation Prenatal-to-Three Program for Families Experiencing Vulnerability.	-EXCLUDE on intervention (service engagement/helpseeking behaviour)	
####	Giorgio (2016) (ID:87853170)	Social Support, Sexual Violence, and Transactional Sex Among Female Transnational Migrants to South Africa	-EXCLUDE on country (High-Income)	
####	Gipson (2023) (ID:87857519)	Now It's My Time! Black Girls Finding Space and Place in Comic Books	-EXCLUDE on population (no housing precarity)	
####	Girard (2014) (ID:87848939)	Mental health outreach and street policing in the downtown of a large French city.	-EXCLUDE on intervention (service engagement/helpseeking behaviour)<br>-EXCLUDE - but review for literature	
####	Girard (2015) (ID:87848813)	Psychometric properties of the recovery measurement in homeless people with severe mental illness.	-EXCLUDE on intervention (service engagement/helpseeking behaviour)	
####	Giving Back: Helping... (2011) (ID:87852037)	Giving Back: Helping Local Groups Brighten Neighbors' Holidays This Year	-EXCLUDE on intervention (service engagement/helpseeking behaviour)	
####	Gladden (2013) (ID:87851151)	The coping strategies of Sudanese refugee women in Kakuma Refugee Camp, Kenya.	-EXCLUDE on country (High-Income)	
####	Glaser (2019) (ID:87848457)	Medical Students' Perceptions of and Responses to Health Care Disparities During Clinical Clerkships.	-EXCLUDE on population (no housing precarity)	
####	GLASGOW (1985) (ID:87857030)	Re-housing hostel residents: the experience in Glasgow	-EXCLUDE on date (2010)	
####	GLASHEEN (2016) (ID:87857294)	Online counselling in secondary schools: would students seek help by this medium?	-EXCLUDE on population (no housing precarity)	
####	Glasheen (2019) (ID:87848433)	Residential Transience Among Adults: Prevalence, Characteristics, and Association with Mental Illness and Mental Health Service Use.	-EXCLUDE on population (no housing precarity)	
####	Glasheen (2019) (ID:87850740)	Residential transience among US adolescents: Association with depression and mental health treatment.	-EXCLUDE on population (no housing precarity)	
####	Glaspy (2021) (ID:87848215)	Exploring and understanding HCV patient journeys- HEPCARE Europe project.	-EXCLUDE on population (women)	
####	GLASS (2012) (ID:87857257)	Help seeking: perceived risk for African American women	-EXCLUDE on population (no housing precarity)	
####	Gleeson (2022) (ID:87857465)	Exploring gendered differences among polish migrants in the UK in problematic drinking and pathways into and through alcohol treatment	-EXCLUDE on population (no housing precarity)	
####	Glendening (2018) (ID:87848584)	Long-term housing subsidies and SSI/SSDI income: Creating health-promoting contexts for families experiencing housing instability with disabilities.	-EXCLUDE on population (no gender focus; women population <50)<br>-EXCLUDE - but review for literature	
####	Glibicky (2021) (ID:87858113)	Co-designing with the Unhoused Community: Cultivating Trust, Defining Needs, and Making It Happen	-EXCLUDE on population (women)	
####	Glick (2018) (ID:87848536)	Addressing the Homeless Mentally Ill in San Francisco.	-EXCLUDE on population (women)	
####	Glick (2018) (ID:87857820)	"Tiptoeing Around the System": Alternative Healthcare Navigation Among Gender Minorities in New Orleans	-EXCLUDE on population (no housing precarity)	
####	Glick (2019) (ID:87848386)	"Housing Insecurity Seems to Almost Go Hand in Hand with Being Trans": Housing Stress among Transgender and Gender Non-conforming Individuals in New Orleans.	-INCLUDE on title & abstract	-EXCLUDE on intervention (service engagement/helpseeking behaviour)
####	Global, regional, and... (2021) (ID:87851812)	Global, regional, and national progress towards Sustainable Development Goal 3.2 for neonatal and child health: all-cause and cause-specific mortality findings from the Global Burden of Disease Study 2019.	-EXCLUDE on intervention (service engagement/helpseeking behaviour)	
####	Glover (2017) (ID:87857722)	"Black Lesbians—Who Will Fight for Our Lives but Us?": Navigating Power, Belonging, Labor, Resistance, and Graduate Student Survival in the Ivory Tower	-EXCLUDE on population (no housing precarity)	

####	**GLUMBIKOVA (2019) (ID:87857061)**	**Critical reflection of the reintegration process through the lens of gender oppression: the case of social work with mothers in shelters**	**-INCLUDE on title & abstract**	**-INCLUDE on full study**
####	Gocotano (2015) (ID:87848809)	Exposure to cold weather during a mass gathering in the Philippines.	-EXCLUDE on country (High-Income)	
####	Goeury (2021) (ID:87853409)	Mobilizing partners to fight HIV/AIDS: the risks of institutional demobilization in a weak and concentrated epidemic. The case of the Amali program in Morocco	-EXCLUDE on country (High-Income)	
####	Goitia (2022) (ID:87848160)	Factors associated with weight gain during the COVID-19 pandemic.	-EXCLUDE on intervention (service engagement/helpseeking behaviour)	
####	Gold (2016) (ID:87857891)	Not Our Regularly Scheduled Programming: Integrating Feminist Theory, Popular Culture, and Writing Pedagogy	-EXCLUDE on intervention (service engagement/helpseeking behaviour)	
####	Goldblatt (2019) (ID:87857753)	Bloody unfair: Inequality related to menstruation - considering the role of discrimination law	-EXCLUDE on population (no housing precarity)	
####	Goldenberg (2022) (ID:87850593)	Violence, policing, and systemic racism as structural barriers to substance use treatment amongst women sex workers who use drugs: Findings of a community-based cohort in Vancouver, Canada (2010-2019).	-EXCLUDE on intervention (service engagement/helpseeking behaviour)	
####	Goldman-Hasbun (2019) (ID:87852804)	Food insufficiency is associated with depression among street-involved youth in a Canadian setting	-EXCLUDE on intervention (service engagement/helpseeking behaviour)	
####	Goldstein (2021) (ID:87857605)	The Threat of Poverty Without Misery	-EXCLUDE on evidence and form (evidence not in written form or presented as research output)	
####	Goldstraw (2016) (ID:87850540)	Operationalising love within austerity : an analysis of the opportunities and challenges experienced by the voluntary and community sector in Greater Manchester under the Coalition government (2010-2015)	-EXCLUDE on intervention (service engagement/helpseeking behaviour)<br>-EXCLUDE - but review for literature	
####	Golinelli (2016) (ID:87848747)	Racial Differences in Cigarette Smoking Among Homeless Youth.	-EXCLUDE on intervention (service engagement/helpseeking behaviour)	
####	Gomez (2010) (ID:87849262)	Factors associated with substance use among homeless young adults.	-EXCLUDE on intervention (service engagement/helpseeking behaviour)	
####	Gonadotropins (2012) (ID:87851848)	Gonadotropins	-EXCLUDE on population (no housing precarity)	
####	Gondek (2018) (ID:87851926)	Predictors of mental health help-seeking among Polish people living in the United Kingdom	-EXCLUDE on population (women)<br>-EXCLUDE - but review for literature	
####	Gonyea (2017) (ID:87857416)	Older homeless women's identity negotiation: agency, resistance, and the construction of a valued self	-EXCLUDE on intervention (service engagement/helpseeking behaviour)<br>-EXCLUDE - but review for literature	
####	Gonzales (2020) (ID:87857570)	Voices from the Field: Ecological Factors that Promote Employment and Health Among Low-Income Older Adults with Implications for Direct Social Work Practice	-EXCLUDE on intervention (service engagement/helpseeking behaviour)	
####	Gonzales (2021) (ID:87963167)	Variation in provider attitudes and treatment recommendations for individuals with schizophrenia and additional marginalized identities: A mixed-method study.	-EXCLUDE on intervention (service engagement/helpseeking behaviour)	
####	Gonzalez (2022) (ID:87850601)	"A center for trans women where they help you": Resource needs of the immigrant Latinx transgender community.	-INCLUDE on title & abstract	-EXCLUDE on target group (no housing precarity)
####	Gonzalez (2022) (ID:87853306)	Colorectal Cancer Screening in Castilla La Mancha, Spain: The Influence of Social, Economic, Demographic and Geographic Factors	-EXCLUDE on intervention (service engagement/helpseeking behaviour)	
####	Gonzalez (2023) (ID:87857981)	Empowerment Support Group for LGBTQ Students: A Grant Proposal	-EXCLUDE on evidence and form (evidence not in written form or presented as research output)	
####	Gonzalez-Nieto (2023) (ID:87849332)	Intersections between syndemic conditions and stages along the continuum of overdose risk among women who inject drugs in Mexicali, Mexico	-EXCLUDE on country (High-Income)	
####	Goode (2023) (ID:87848134)	Evaluation of a team-based quality improvement initiative for influenza vaccination in an underserved population.	-EXCLUDE on intervention (service engagement/helpseeking behaviour)	
####	Goodhew (2016) (ID:87848715)	Mental health among clients of the Sydney Medically Supervised Injecting Centre (MSIC).	-EXCLUDE on intervention (service engagement/helpseeking behaviour)	
####	Goodling (2020) (ID:87857421)	Intersecting hazards, intersectional identities: A baseline Critical Environmental Justice analysis of US homelessness	-EXCLUDE on intervention (service engagement/helpseeking behaviour)	
####	Goodman (2010) (ID:87852994)	Beyond the 50-Minute Hour: Increasing Control, Choice, and Connections in the Lives of Low-Income Women	-EXCLUDE on population (no housing precarity)	

####	Goodman (2013) (ID:87851187)	Screening for substance abuse in women's health: A public health imperative.	-EXCLUDE on intervention (service engagement/helpseeking behaviour)	
####	Goodman (2016) (ID:87848712)	A Randomized Trial of Dialectical Behavior Therapy in High-Risk Suicidal Veterans.	-EXCLUDE on intervention (service engagement/helpseeking behaviour)	
####	Goodman (2017) (ID:87848681)	Why do Kenyan children live on the streets? Evidence from a cross-section of semi-rural maternal caregivers.	-EXCLUDE on country (High-Income)	
####	Goodsmith (2021) (ID:87850646)	Addressing the urgent housing needs of vulnerable women in the era of COVID-19: The Los Angeles county experience.	-INCLUDE on title & abstract	-EXCLUDE on intervention (intersectionality)
####	Goossen (2017) (ID:87963116)	Service user involvement in UK social service agencies and social work education	-EXCLUDE on population (no housing precarity)	
####	GORCZYNSKI (2020) (ID:87857161)	Evaluating mental health literacy and help-seeking behaviours in UK university students: a country wide study	-EXCLUDE on population (no housing precarity)	
####	GORDE (2004) (ID:87857143)	Trauma symptoms and life skill needs of domestic violence victims	-EXCLUDE on date (2010)	
####	Gordon (2012) (ID:87849121)	Health and social adjustment of homeless older adults with a mental illness.	-EXCLUDE on intervention (service engagement/helpseeking behaviour)	
####	Gordon (2016) (ID:87848706)	Factors Associated With Receiving Rapid HIV Testing Among Individuals on Probation or Parole.	-EXCLUDE on intervention (service engagement/helpseeking behaviour)	
####	Gordon (2018) (ID:87850796)	Wounds of war: How the VA delivers health, healing, and hope to the nation's veterans.	-EXCLUDE on intervention (service engagement/helpseeking behaviour)	
####	**Gordon (2019) (ID:87848389)**	**Influence of past trauma and health interactions on homeless women's views of perinatal care: a qualitative study.**	**-INCLUDE on title & abstract**	**-INCLUDE on full study**
####	Gordon (2022) (ID:87848153)	Appropriate and acceptable health assessments for people experiencing homelessness.	-EXCLUDE on population (no gender focus; women population <50)	
####	Gore (2020) (ID:87858046)	Hyper-Incarceration: An Analysis of Multi-Generational Factors for African American Parents Who Are Repeat Offenders	-EXCLUDE on population (no housing precarity)	
####	Gore (2022) (ID:87848169)	Coronavirus Disease 2019 and Hospital Readmissions: Patient Characteristics and Socioeconomic Factors Associated With Readmissions in an Urban Safety-Net Hospital System.	-EXCLUDE on population (no housing precarity)	
####	GORMAN-MURRAY (2013) (ID:87857723)	Urban Homebodies: Embodiment, Masculinity, and Domesticity in Inner Sydney	-EXCLUDE on evidence and form (evidence not in written form or presented as research output)	
####	GORTON (2000) (ID:87856976)	Homeless young women and pregnancy: pregnancy in hostels for single homeless people	-EXCLUDE on date (2010)	
####	Gosain (2020) (ID:87851609)	Geographic and demographic features of neuroendocrine tumors in the United States of America: A population-based study.	-EXCLUDE on population (no housing precarity)	
####	Goswami (2011) (ID:87849165)	Feasibility and willingness-to-pay for integrated community-based tuberculosis testing.	-EXCLUDE on population (women)	
####	Goto (2019) (ID:87853192)	Sex-specific differences in survival after out-of-hospital cardiac arrest: a nationwide, population-based observational study	-EXCLUDE on population (no housing precarity)	
####	Gough (2020) (ID:87963383)	Mental health, men and culture: how do sociocultural constructions of masculinities relate to men's mental health help-seeking behaviour in the WHO European Region?	-EXCLUDE on population (women)	
####	Gowda (2017) (ID:87848622)	Clinical outcome and rehabilitation of homeless mentally ill patients admitted in mental health institute of South India: "Know the Unknown" project.	-EXCLUDE on country (High-Income)	
####	GOWEN (2013) (ID:87857339)	Online mental health information seeking in young adults with mental health challenges	-EXCLUDE on population (no housing precarity)	
####	Goyal (2021) (ID:87848228)	Impact of COVID-19 on Maternal Mental Health.	-EXCLUDE on population (no housing precarity)	
####	GRACE (2016) (ID:87857003)	Transferability of the youth foyer model for women exiting the criminal justice system	-EXCLUDE on intervention (service engagement/helpseeking behaviour)	
####	Grady (2012) (ID:87858124)	A Critique of Neoliberalism with Fierceness: Queer Youth of Color Creating Dialogues of Resistance	-EXCLUDE on population (no housing precarity)	
####	Graham (2014) (ID:87851054)	"Navigating community institutions: Black transgender women's experiences in schools, the criminal justice system, and churches": Erratum.	-EXCLUDE on evidence and form (evidence not in written form or presented as research output)	

####	Graham (2014) (ID:87852940)	Navigating Community Institutions: Black Transgender Women's Experiences in Schools, the Criminal Justice System, and Churches	-EXCLUDE on population (no housing precarity)	
####	Graham (2018) (ID:87853403)	Racial Disparities in Patient Characteristics and Survival After Acute Myocardial Infarction	-EXCLUDE on population (no housing precarity)	
####	Graham (2022) (ID:87850591)	The Danger Assessment: An instrument for the prevention of intimate partner homicide.	-EXCLUDE on intervention (service engagement/helpseeking behaviour)	
####	Grangeiro (2012) (ID:87853370)	Prevalence and vulnerability of homeless people to HIV infection in Sao Paulo, Brazil	-EXCLUDE on country (High-Income)	
####	Grant (2018) (ID:87852000)	Developing a Volunteer Training Program at Mount Calvary Baptist Church for Mentoring At-Risk Homeless Women	-INCLUDE on title & abstract	-EXCLUDE on intervention (service engagement/helpseeking behaviour)
####	Grauerholz (2016) (ID:87857963)	Teaching Inequalities: Using Public Transportation and Visual Sociology to Make It Real	-EXCLUDE on intervention (service engagement/helpseeking behaviour)	
####	Graves (2015) (ID:87851945)	A Grounded Theory on the Help-Seeking Process of Single African American Mothers Living in Poverty.	-EXCLUDE on population (no housing precarity)	
####	Gray (2016) (ID:87848802)	Changing Social Networks Among Homeless Individuals: A Prospective Evaluation of a Job- and Life-Skills Training Program.	-EXCLUDE on population (women)	
####	Gray (2017) (ID:87848606)	How do homeless adults change their lives after completing an intensive job-skills program? A prospective study.	-EXCLUDE on intervention (service engagement/helpseeking behaviour)	
####	Gray (2019) (ID:87858036)	LGBTQ Adolescent Smoking: A Social Media Approach to Primary Smoking Prevention	-EXCLUDE on population (no housing precarity)	
####	Gray (2022) (ID:87857886)	The Cultural Significance of “We-Ness”: Motivationally Influential Practices Rooted in a Scholarly Agenda on Black Education	-EXCLUDE on population (no housing precarity)	
####	Grazioli (2015) (ID:87848877)	Perceptions of twelve-step mutual-help groups and their associations with motivation, treatment attendance and alcohol outcomes among chronically homeless individuals with alcohol problems.	-EXCLUDE on population (women)	
####	GREAT (2008) (ID:87857155)	Better outcomes: the way forward: improving the care of unaccompanied asylum seeking children	-EXCLUDE on date (2010)	
####	GREAT (2014) (ID:87857195)	Consultation on the draft regulations and statutory guidance for local authorities on the care of unaccompanied asylum seeking and trafficked children	-EXCLUDE on evidence and form (evidence not in written form or presented as research output)	
####	GREAT (2016) (ID:87857010)	Homelessness: third report of session 2016-17: report, together with formal minutes relating to the report	-EXCLUDE on intervention (service engagement/helpseeking behaviour)	
####	GREAT (2017) (ID:87857356)	Future of supported housing: first joint report of the Communities and Local Government and Work and Pensions Committees of Session 2016-17	-EXCLUDE on intervention (service engagement/helpseeking behaviour)	
####	Greco (2015) (ID:87848859)	What is a good life? Selecting capabilities to assess women's quality of life in rural Malawi.	-EXCLUDE on country (High-Income)	
####	Green (2013) (ID:87849037)	Shared risk: who engages in substance use with American homeless youth?.	-EXCLUDE on population (women)	
####	Green (2014) (ID:87858039)	Into the Darkness: A Quare (Re)membering of Los Angeles in a Time of Crises, (1981-Present)	-EXCLUDE on intervention (service engagement/helpseeking behaviour)	
####	GREEN (2016) (ID:87857330)	Whom do migrant home care workers contact in the case of work-related abuse? an exploratory study of help-seeking behaviors	-EXCLUDE on population (no housing precarity)	
####	Green (2018) (ID:87849371)	Exploring the Implications of Shifting HIV Prevention Practice Ideologies on the Work of Community-Based Organizations: A Resource Dependence Perspective	-EXCLUDE on population (no housing precarity)	
####	Green (2018) (ID:87852639)	Interacting With Providers: An Intersectional Exploration of the Experiences of Carers of Aboriginal Children With a Disability	-EXCLUDE on population (no housing precarity)	
####	Green (2021) (ID:87848184)	The social determinants of health and health outcomes among adults during the COVID-19 pandemic: A systematic review.	-EXCLUDE on intervention (service engagement/helpseeking behaviour)	
####	Green (2023) (ID:88019163)	Influence of Cultural Norms on Formal Service Engagement Among Survivors of Intimate Partner Violence: A Qualitative Meta-synthesis	-INCLUDE on title & abstract	-EXCLUDE (systematic review)* only use with other code<br>-EXCLUDE (IPV/DVA but little discussion on housing)

####	Greenbaum (2014) (ID:87853168)	Commercial Sexual Exploitation and Sex Trafficking of Children in the United States	-EXCLUDE on population (people aged under 18 years)	
####	Greenbaum (2017) (ID:87852930)	Global Human Trafficking and Child Victimization	-EXCLUDE on population (people aged under 18 years)<br>-EXCLUDE - but review for literature	
####	**Greene (2013) (ID:87853144)**	**A House is not a Home: The Housing Experiences of African and Caribbean Mothers Living with HIV**	**-INCLUDE on title & abstract**	**-INCLUDE on full study**
####	**Greenfield (2021) (ID:87850688)**	**Learning to endure: A qualitative examination of the protective factors of homeless transgender and gender expansive youth engaged in survival sex.**	**-INCLUDE on title & abstract**	**-INCLUDE on full study**
####	Greenwood (2017) (ID:87848687)	Mastery matters: consumer choice, psychiatric symptoms and problematic substance use among adults with histories of homelessness.	-EXCLUDE on intervention (service engagement/helpseeking behaviour)	
####	Greenwood (2020) (ID:87848342)	Homeless Adults' Recovery Experiences in Housing First and Traditional Services Programs in Seven European Countries.	-EXCLUDE on intervention (service engagement/helpseeking behaviour)	
####	Greer (2019) (ID:87858093)	Fostered Voices: Narratives of U.S. Foster Care	-EXCLUDE on evidence and form (evidence not in written form or presented as research output)	
####	Greeson (2019) (ID:87853421)	Child welfare characteristics in a sample of youth involved in commercial sex: An exploratory study	-EXCLUDE on population (no gender focus; women population <50)	
####	Greeson (2020) (ID:87850715)	Youth Matters: Philly (YMP): Development, usability, usefulness, & accessibility of a mobile web-based app for homeless and unstably housed youth.	-EXCLUDE on intervention (service engagement/helpseeking behaviour)	
####	GREGG (2021) (ID:87857101)	What is the relationship between people with dementia and their caregiver's illness perceptions post-diagnosis and the impact on help-seeking behaviour? A systematic review	-EXCLUDE on population (no housing precarity)	
####	Gregor (2020) (ID:87851489)	The quality of mental health care received by homeless inpatients	-EXCLUDE on intervention (service engagement/helpseeking behaviour)	
####	Greif (2012) (ID:87853199)	Housing, medical, and food deprivation in poor urban contexts: implications for multiple sexual partnerships and transactional sex in Nairobi's slums	-EXCLUDE on country (High-Income)	
####	Greig (2018) (ID:87857878)	Wireless Mesh Networks as Community Hubs: Analysis of Small-Scale Wireless Mesh Networks and Community-Centered Technology Training	-EXCLUDE on intervention (service engagement/helpseeking behaviour)	
####	Grenier (2016) (ID:87848718)	'Growing Old' in Shelters and 'On the Street': Experiences of Older Homeless People.	-EXCLUDE on population (no gender focus; women population <50)	
####	Greponne (2021) (ID:87850643)	Compassion fatigue and intersectionality in human service practitioners: Latina low-wage-earners fighting poverty.	-EXCLUDE on population (no housing precarity)	
####	Greponne (2021) (ID:87851897)	Compassion Fatigue and Intersectionality in Human Service Practitioners: Latina Low-Wage-Earners Fighting Poverty	-EXCLUDE on intervention (service engagement/helpseeking behaviour)	
####	Griffin (2021) (ID:87858101)	The Association between Justice System Contact, Psychological Distress, and Physical Illness: An Examination of Illicit Drug Use	-EXCLUDE on intervention (service engagement/helpseeking behaviour)	
####	Griffin (2022) (ID:87850590)	Resilience in the face of homelessness: Investigating the within-day emotional and academic processes of homeless youth.	-EXCLUDE on intervention (service engagement/helpseeking behaviour)	
####	GRIFFITH (2011) (ID:87857347)	Social and cultural factors influence African American men's medical help seeking	-EXCLUDE on population (women)	
####	Grimes (2020) (ID:87853048)	Perspectives on an earn-a-bike intervention on transportation, health and self- esteem among men experiencing homelessness	-EXCLUDE on population (women)	
####	Groessl (2011) (ID:87849234)	The hepatitis C self-management programme: a randomized controlled trial.	-EXCLUDE on intervention (service engagement/helpseeking behaviour)	
####	Groessl (2013) (ID:87849050)	The hepatitis C self-management program: sustainability of primary outcomes at 1 year.	-EXCLUDE on population (women)	
####	Grooms (2020) (ID:87857493)	No Home and No Acceptance: Exploring the Intersectionality of Sexual/Gender Identities (LGBTQ) and Race in the Foster Care System	-EXCLUDE on population (people aged under 18 years)	
####	Grose (2022) (ID:87853427)	The youth HIV policy advisors programme: Creating change agents and policy leaders	-EXCLUDE on intervention (service engagement/helpseeking behaviour)	
####	Groton (2019) (ID:87848465)	Social networks of unaccompanied women experiencing homelessness.	-INCLUDE on title & abstract	-EXCLUDE on intervention (intersectionality)

####	Groulx (2020) (ID:87851612)	Guideline on screening for esophageal adenocarcinoma in patients with chronic gastroesophageal reflux disease.	-EXCLUDE on population (no housing precarity)	
####	Grove (2022) (ID:87848142)	Permanent Supportive Housing Tenure Among a Heterogeneous Population of Adults with Disabilities.	-EXCLUDE on intervention (service engagement/helpseeking behaviour)	
####	Grunden (2014) (ID:87857735)	The Academic Experiences of Women Post 9/11 Veterans Attending Post-Secondary Educational Institutions	-EXCLUDE on population (no housing precarity)	
####	Gruszczynski (2022) (ID:87851652)	Effects of Social Determinants of Health Care on Pediatric Thyroid Cancer Outcomes in the United States.	-EXCLUDE on population (no housing precarity)	
####	Gryczynski (2020) (ID:87848301)	Leveraging health information exchange for clinical research: Extreme underreporting of hospital service utilization among patients with substance use disorders.	-EXCLUDE on population (no gender focus; women population <50)	
####	Gu (2021) (ID:87851565)	Factors associated with mental health outcomes among patients with COVID-19 treated in the Fangcang shelter hospital in China.	-EXCLUDE on country (High-Income)	
####	Guadalupe-Diaz (2017) (ID:87963382)	“I wasn’ta priority, I wasn’ta victim” challenges in help seeking for transgender survivors of intimate partner violence	-INCLUDE on title & abstract	-EXCLUDE (IPV/DVA but little discussion on housing)
####	Guedes (2021) (ID:87853388)	Diversity and Adults Education: Collective Resistance Strategies in Pandemic Times	-EXCLUDE on country (High-Income)	
####	Guide for developing... (FEANTSA) (ID:88019170)	Guide for developing effective gender-responsive support and solutions for women experiencing homelessness	-INCLUDE on title & abstract	-EXCLUDE on intervention (intersectionality)
####	Guidry-Grimes (2019) (ID:87848391)	Homeless, Ill, and Psychiatrically Complex: The Grueling Carousel of Cassandra Lee.	-EXCLUDE on intervention (service engagement/helpseeking behaviour)	
####	Guilcher (2020) (ID:87848340)	Factors related to screening for problem gambling among healthcare and social service providers in Ontario, Canada: A concept mapping study.	-EXCLUDE on population (no housing precarity)	
####	Guimaraes (2014) (ID:87848983)	Correlates of HIV infection among patients with mental illness in Brazil.	-EXCLUDE on country (High-Income)	
####	**Gultekin (2014) (ID:87848908)**	**Voices from the street: exploring the realities of family homelessness.**	**-INCLUDE on title & abstract**	**-INCLUDE on full study**
####	**Gultekin (2023) (ID:88019138)**	**Trauma-Disclosure, Meaning-Making, and Help-Seeking in Mothers Experiencing Homelessness: Results From a Trauma-Focused, Clinical Ethnographic Narrative Intervention**	**-INCLUDE on title & abstract**	**-INCLUDE on full study**
####	Gummerson (2021) (ID:87852735)	The wealth gradient and the effect of COVID-19 restrictions on income loss, food insecurity and health care access in four sub-Saharan African geographies	-EXCLUDE on country (High-Income)	
####	Gunn (2015) (ID:87857469)	Intra-group stigma: Examining peer relationships among women in recovery for addictions	-EXCLUDE on population (no housing precarity)	
####	Gutman (2018) (ID:87848501)	Feasibility and Satisfaction of an Apartment Living Program for Homeless Adults With Mental Illness and Substance Use Disorder.	-EXCLUDE on population (women)	
####	Gutman (2021) (ID:87850634)	The association between LGBTQIA+ self-identification and factors facilitating homelessness: A scoping review of the occupational therapy peer-reviewed literature.	-INCLUDE on title & abstract	-EXCLUDE on target group (no housing precarity)
####	Guzman (2023) (ID:87963151)	Help-Seeking Behaviors in African American LGBTQ+ Adolescents: A Retrospective Study	-EXCLUDE on population (no housing precarity)	
####	Guzman-Parra (2023) (ID:87848127)	The Impact of Stressful Life Events on Suicidal Ideation in Gender Dysphoria: A Moderator Effect of Perceived Social Support.	-EXCLUDE on intervention (service engagement/helpseeking behaviour)	
####	Gwadz (2018) (ID:87852799)	Do Programs for Runaway and Homeless Youth Work? A Qualitative Exploration From the Perspectives of Youth Clients in Diverse Settings	-EXCLUDE on intervention (service engagement/helpseeking behaviour)	
####	Gyllensten (2017) (ID:87848672)	Computerized physical activity training for persons with severe mental illness - experiences from a communal supported housing project.	-EXCLUDE on intervention (service engagement/helpseeking behaviour)	
####	Ha (2015) (ID:87850994)	Barriers and facilitators to shelter utilization among homeless young adults.	-EXCLUDE on population (no gender focus; women population <50)	
####	Haal (2018) (ID:87853375)	The rise and fall of mortality inequality in South Africa in the HIV era	-EXCLUDE on country (High-Income)	

####	Haas (2020) (ID:87852834)	Excess mortality associated with mental illness in people living with HIV in Cape Town, South Africa: a cohort study using linked electronic health records	-EXCLUDE on country (High-Income)	
####	Haase (2016) (ID:87853252)	Association of socio-demographic factors with the age at death due to cardiovascular diseases	-EXCLUDE on population (no housing precarity)	
####	Habib (2014) (ID:87848923)	Associations between life conditions and multi-morbidity in marginalized populations: the case of Palestinian refugees.	-EXCLUDE on country (High-Income)	
####	Hackett (2015) (ID:87857784)	"Helping women help themselves": An ethnography of carceral empowerment and the neoliberal rehabilitative ideal at a recovery center for criminalized women	-EXCLUDE on population (no housing precarity)	
####	Hadenfeldt (2017) (ID:87848605)	Frailty Assessment in Patients Utilizing a Free Clinic.	-EXCLUDE on intervention (service engagement/helpseeking behaviour)	
####	Hadweh (2019) (ID:87851668)	Knowledge, opportunities, challenges, and the way forward for reproductive health rights: a qualitative study of women in the Bethlehem area of the West Bank	-EXCLUDE on country (High-Income)	
####	Haeri (2015) (ID:87851653)	Emergency Preparedness in Obstetrics	-EXCLUDE on population (no housing precarity)	
####	HAGAN (2021) (ID:87857136)	Doing their damnedest to seek change: how group identity helps people with dementia confront public stigma and maintain purpose	-EXCLUDE on population (no housing precarity)	
####	Hailemariam (2018) (ID:87848515)	Correlates of alcoholics anonymous affiliation among justice-involved women.	-EXCLUDE on intervention (service engagement/helpseeking behaviour)	
####	Hailemariam (2020) (ID:87848327)	Intersectionality, special populations, needs and suggestions: the Flint Women's study.	-INCLUDE on title & abstract	-EXCLUDE on target group (no housing precarity)
####	HAILES (2018) (ID:87856978)	Hand in hand: survivors of multiple disadvantage discuss service and support	-INCLUDE on title & abstract	-EXCLUDE on intervention (intersectionality)
####	HAILSTONE (2017) (ID:87857092)	The development of Attitudes of People from Ethnic Minorities to Help-Seeking for Dementia (APEND): a questionnaire to measure attitudes to help-seeking for dementia in people from South Asian backgrounds in the UK	-EXCLUDE on population (no housing precarity)	
####	Hakanson (2016) (ID:87848709)	Illness narratives of people who are homeless.	-EXCLUDE on intervention (service engagement/helpseeking behaviour)<br>-EXCLUDE - but review for literature	
####	HALE (2010) (ID:87857391)	Male GPs' views on men seeking medical help: a qualitative study	-EXCLUDE on population (women)	
####	Haley (2011) (ID:87851357)	Performance contracting to engage detoxification-only patients into continued rehabilitation.	-EXCLUDE on population (no housing precarity)	
####	Haley (2014) (ID:87857595)	Multilevel challenges to engagement in HIV care after prison release: A theory-informed qualitative study comparing prisoners' perspectives before and after community reentry	-EXCLUDE on population (no housing precarity)	
####	Haley (2017) (ID:87857968)	Intimate constraints: A feminist political economy analysis of biological reproduction and parenting in high-support housing in Ontario	-EXCLUDE on population (no housing precarity)	
####	Haley (2018) (ID:87852745)	Neighborhood Health Care Access and Sexually Transmitted Infections Among Women in the Southern United States: A Cross-Sectional Multilevel Analysis	-EXCLUDE on population (no housing precarity)	
####	Hall (2014) (ID:87848961)	Mobile opioid agonist treatment and public funding expands treatment for disenfranchised opioid-dependent individuals.	-EXCLUDE on intervention (service engagement/helpseeking behaviour)	
####	Hall (2015) (ID:87852013)	Symptom appraisal and healthcare-seeking for symptoms suggestive of colorectal cancer: a qualitative study	-EXCLUDE on population (no housing precarity)	
####	Hall (2020) (ID:87848363)	Housing versus treatment first for supportive housing participants with substance use disorders: A comparison of housing and public service use outcomes.	-EXCLUDE on intervention (service engagement/helpseeking behaviour)	
####	Hall (2022) (ID:87858027)	Reawakening the Ethical Imagination of the Local Congregation Through the Exploration of the Biblical Metanarrative	-EXCLUDE on population (no housing precarity)	
####	Hall-Lipsy (2010) (ID:87851680)	Pharmacotherapeutic disparities: racial, ethnic, and sex variations in medication treatment	-EXCLUDE on population (no housing precarity)	
####	Hamden (2011) (ID:87849201)	Is deinstitutionalization working in our community?.	-EXCLUDE on population (no housing precarity)	
####	Hamdullahpur (2017) (ID:87853097)	A comparison of socioeconomic status and mental health among inner-city Aboriginal and non-Aboriginal women	-EXCLUDE on intervention (service engagement/helpseeking behaviour)	

####	Hameed (2020) (ID:87848289)	Psychological therapies for women who experience intimate partner violence.	-EXCLUDE on intervention (service engagement/helpseeking behaviour)	
####	Hameed (2020) (ID:87852924)	Psychological therapies for women who experience intimate partner violence	-INCLUDE on title & abstract	-EXCLUDE on intervention (service engagement/helpseeking behaviour)
####	Hamer (2016) (ID:87963283)	BDSM and helpseeking: An exploratory qualitative survey	-EXCLUDE on population (no housing precarity)	
####	HAMID (2013) (ID:87857214)	Factors affecting attitude towards seeking professional help for mental illness: a UK Arab perspective	-EXCLUDE on population (no housing precarity)	
####	Hamilton (2011) (ID:87849183)	"Homelessness and trauma go hand-in-hand": pathways to homelessness among women veterans.	-EXCLUDE on intervention (service engagement/helpseeking behaviour)	
####	HAMILTON (2012) (ID:87856916)	Barriers to psychosocial services among homeless women veterans	-INCLUDE on title & abstract	-EXCLUDE on intervention (intersectionality)
####	Hamilton (2018) (ID:87857594)	Forms of Attention: Notes from Harryette Mullen's Tanka Diary	-EXCLUDE on evidence and form (evidence not in written form or presented as research output)	
####	Hamilton (2019) (ID:87850734)	Reflections on poverty, homelessness and problem gambling: Discoveries from a World Cafe.	-EXCLUDE on population (no gender focus; women population <50)<br>-EXCLUDE - but review for literature	
####	Hamilton (2019) (ID:87963211)	Hegemonic femininities and intersectional domination	-EXCLUDE on population (no housing precarity)	
####	Hamilton (2022) (ID:87857647)	Still I Rise: Exploring the Relationships Between the Impostor Phenomenon, the Strong Black Woman Cultural Construct and Attitudes Toward Seeking Professional Psychology Help in African American College Women	-EXCLUDE on population (no housing precarity)	
####	HAMMER (2021) (ID:87857165)	Impact of integrated care and co-location of care on mental help-seeking perceptions	-EXCLUDE on population (no housing precarity)	
####	Hammoud (2023) (ID:87851866)	COVID-19 Testing in a Weekly Cohort Study of Gay and Bisexual Men: The Impact of Health-Seeking Behaviors and Social Connection	-EXCLUDE on population (women)	
####	Hancock (2010) (ID:87851727)	'Reaching the hard to reach' - lessons learned from the VCS (voluntary and community Sector). A qualitative study	-EXCLUDE on population (no gender focus; women population <50)	
####	Hand (2023) (ID:87857898)	Exploring Predictors of Healing from an Expressive Writing Intervention About Heterosexism and Why LGBTQ+ Clients Might Withhold from Their Therapists	-EXCLUDE on population (no housing precarity)	
####	Hanek (2015) (ID:87858057)	Clinical Use of Sexually Explicit Media in the Treatment of Sexual Concerns	-EXCLUDE on intervention (service engagement/helpseeking behaviour)	
####	HANKEL (2016) (ID:87856911)	Characteristics of women seeking services at a transitional housing facility for women leaving street-based sex work: implications for social service providers	-INCLUDE on title & abstract	-EXCLUDE on intervention (intersectionality)
####	Hankela (2017) (ID:87857882)	'There is a Reason': A Call to Re-Consider the Relationship between Charity and Social Justice	-EXCLUDE on country (High-Income)	
####	**Hanley (2019) (ID:87852896)**	**Migrant women's health and housing insecurity: an intersectional analysis**	**-INCLUDE on title & abstract**	**-INCLUDE on full study**
####	Hansen (2019) (ID:87857750)	Time to Kick Some Glass: Understanding the Lived Experiences of African American Aspiring Female Superintendents	-EXCLUDE on population (no housing precarity)	
####	Hanson (2019) (ID:87848409)	Comprehensive oral care improves treatment outcomes in male and female patients with high-severity and chronic substance use disorders.	-EXCLUDE on intervention (service engagement/helpseeking behaviour)	
####	Harawa (2022) (ID:87848158)	Factors predicting incarceration history and incidence among Black and Latino men who have sex with men (MSM) residing in a major urban center.	-EXCLUDE on population (women)	
####	Harcourt (2018) (ID:87852894)	Frequent presentations to emergency departments and the collaborative community and emergency response: A case series	-EXCLUDE on intervention (service engagement/helpseeking behaviour)	
####	Härd (2022) (ID:87857730)	The growth of recovery capital in clients of recovery residences in Florida, USA: a quantitative pilot study of changes in REC-CAP profile scores	-EXCLUDE on population (no gender focus; women population <50)	
####	Hardill (2015) (ID:87850974)	Nursing practice with incarcerated women: A focused comparative review of the nursing and feminist literature.	-EXCLUDE on intervention (service engagement/helpseeking behaviour)	
####	HARDIN (2017) (ID:87856913)	The homeless individual's viewpoint: causes of homelessness and resources needed to leave the sheltered environment	-EXCLUDE on population (no gender focus; women population <50)	

####	HARDING (2005) (ID:87857175)	Maintaining tenancies: gender differences among young people living independently	-EXCLUDE on date (2010)	
####	Harney (2019) (ID:87848423)	Quantitative evaluation of an integrated nurse model of care providing hepatitis C treatment to people attending homeless services in Melbourne, Australia.	-EXCLUDE on intervention (service engagement/helpseeking behaviour)	
####	Harp (2016) (ID:87848716)	Factors associated with two types of child custody loss among a sample of African American mothers: A novel approach.	-EXCLUDE on intervention (service engagement/helpseeking behaviour)	
####	Harp (2020) (ID:87963199)	The racialized nature of child welfare policies and the social control of Black bodies	-EXCLUDE on intervention (service engagement/helpseeking behaviour)	
####	Harpaz-Rotem (2011) (ID:87849164)	Residential treatment for homeless female veterans with psychiatric and substance use disorders: effect on 1-year clinical outcomes.	-EXCLUDE on intervention (service engagement/helpseeking behaviour)	
####	HARRIS (1996) (ID:87856997)	Treating sexual abuse trauma with dually diagnosed women	-EXCLUDE on date (2010)	
####	Harris (2012) (ID:87849153)	Longer length of stay is not associated with better outcomes in VHA's substance abuse residential rehabilitation treatment programs.	-EXCLUDE on intervention (service engagement/helpseeking behaviour)	
####	Harris (2013) (ID:87851182)	Hepatitis C treatment access and uptake for people who inject drugs: A review mapping the role of social factors.	-EXCLUDE on intervention (service engagement/helpseeking behaviour)	
####	Harris (2015) (ID:87851657)	Familial support impacts incarcerated women ' s housing stability.	-EXCLUDE on intervention (service engagement/helpseeking behaviour)	
####	Harris (2019) (ID:87848392)	Mental health change in the transition to permanent supportive housing: The role of housing and social networks.	-EXCLUDE on intervention (service engagement/helpseeking behaviour)	
####	Harris (2019) (ID:87853045)	Los Angeles housing models and neighbourhoods' role in supportive housing residents' social integration	-EXCLUDE on population (women)	
####	Harris (2019) (ID:87963089)	Responding to complexity: improving service provision for survivors of domestic abuse with 'complex needs'	-INCLUDE on title & abstract	-EXCLUDE on intervention (intersectionality)
####	Harris (2020) (ID:87857517)	Intersecting Identities, Intersecting Issues: Exploring the Needs of LGBTQ+ Communities in Wisconsin	-EXCLUDE on population (no housing precarity)	
####	Harris (2020) (ID:87857954)	LGBTQI+ and Christian? Who decides?	-EXCLUDE on population (no housing precarity)	
####	Harris (2021) (ID:87857810)	Veteran Social Network: Peer Support Impact on Mental Health Service Utilization	-EXCLUDE on population (no housing precarity)	
####	Harris-Fry (2017) (ID:87852771)	Determinants of intra-household food allocation between adults in South Asia - a systematic review	-EXCLUDE on country (High-Income)	
####	Harrison (2014) (ID:87852909)	A collaborative outreach clinic for pregnant youth and adolescent mothers: Description of a pilot clinic and its patients	-EXCLUDE on intervention (service engagement/helpseeking behaviour)	
####	Harrison (2016) (ID:87858119)	Embedding Social Justice within Micro Social Work Curricula	-EXCLUDE on intervention (service engagement/helpseeking behaviour)	
####	Harrison (2022) (ID:87857539)	Employment and further study outcomes for care-experienced graduates in the UK	-EXCLUDE on population (no housing precarity)	
####	Hartnell (2021) (ID:87857717)	Messages and Attitudes about Help-Seeking and Mental Health in Black Male College Students	-EXCLUDE on population (women)	
####	HARTNETT (2010) (ID:87857084)	The function of shelters for women: assistance or social control?	-INCLUDE on title & abstract	-EXCLUDE on intervention (intersectionality)
####	Harvey (2012) (ID:87849116)	A place to live: housing needs for people with psychotic disorders identified in the second Australian National Survey of Psychosis.	-EXCLUDE on intervention (service engagement/helpseeking behaviour)	
####	HASHIMOTO (2021) (ID:87857142)	Help-seeking behaviors for intimate partner violence perpetration by men receiving substance use treatment: a mixed-methods secondary analysis	-EXCLUDE on population (women)	
####	Haskett (2017) (ID:87852638)	Feasibility, acceptability, and effects of a peer support group to prevent child maltreatment among parents experiencing homelessness	-EXCLUDE on intervention (service engagement/helpseeking behaviour)	
####	Hassan (2022) (ID:87848155)	A qualitative study of service engagement and unmet needs among unstably housed people who inject drugs in Massachusetts.	-EXCLUDE on population (no gender focus; women population <50)	
####	Hastrup (2019) (ID:87848473)	Does future resource input reflect need in first-episode psychosis: Examining the association between individual characteristics and 5-year costs.	-EXCLUDE on intervention (service engagement/helpseeking behaviour)	

####	HATCHETT (2004) (ID:87857178)	Health-seeking patterns for AIDS in Malawi	-EXCLUDE on country (High-Income)	
####	Hatef (2021) (ID:87848243)	Assessing the Impact of Social Needs and Social Determinants of Health on Health Care Utilization: Using Patient- and Community-Level Data.	-EXCLUDE on intervention (service engagement/helpseeking behaviour)	
####	HATHAZI (2009) (ID:87857015)	Pregnancy and sexual health among homeless young injection drug users	-EXCLUDE on date (2010)	
####	Hawk (2012) (ID:87849140)	The effects of a harm reduction housing program on the viral loads of homeless individuals living with HIV/AIDS.	-EXCLUDE on intervention (service engagement/helpseeking behaviour)	
####	HAWKINS (2007) (ID:87857080)	Disappearing acts: The social networks of formerly homeless individuals with co-occurring disorders	-EXCLUDE on date (2010)	
####	HAWKINS (2010) (ID:87851442)	Fickle families and the kindness of strangers: social capital in the lives of low-income single mothers	-INCLUDE on title & abstract	-EXCLUDE on intervention (intersectionality)
####	Hawkins (2021) (ID:87848245)	Contraceptive usage in homeless women accessing a dedicated primary care service in Scotland, UK: a case note review.	-EXCLUDE on intervention (service engagement/helpseeking behaviour)	
####	Haynes (2019) (ID:87857822)	Community treatment orders and social factors: complex journeys in the mental health system	-EXCLUDE on population (no housing precarity)	
####	Hayward (2018) (ID:87857663)	Perceived barriers to mental health treatment among men enrolled in a responsible fatherhood program	-EXCLUDE on population (women)	
####	Health consumption in... (Norum) (ID:87851710)	Health consumption in Sami-speaking municipalities with regard to cancer and radiotherapy	-EXCLUDE on intervention (service engagement/helpseeking behaviour)	
####	Heard (2020) (ID:87857852)	Using Applied Theater in Primary, Secondary, and Tertiary Prevention of Intimate Partner Violence: A Systematic Review	-EXCLUDE on intervention (service engagement/helpseeking behaviour)	
####	Heavey (2014) (ID:87851658)	Female refugees: sensitive care needed	-INCLUDE on title & abstract	-EXCLUDE on intervention (service engagement/helpseeking behaviour)
####	Heerde (2017) (ID:87850897)	The role of risk and protective factors in the modification of risk for sexual victimization, sexual risk behaviors, and survival sex among homeless youth: A meta-analysis.	-EXCLUDE on intervention (service engagement/helpseeking behaviour)	
####	Heerde (2020) (ID:87857480)	"I'd rather injure somebody else than get injured": An introduction to the study of exposure to physical violence among young people experiencing homelessness	-EXCLUDE on intervention (service engagement/helpseeking behaviour)	
####	Heerde (2021) (ID:87963250)	“I've seen my friend get chopped”: The influence of peer networks on exposure to violence among homeless young adults	-EXCLUDE on intervention (service engagement/helpseeking behaviour)	
####	HEFFERNAN (2018) (ID:87857593)	“As Usual, I'll Have to Take an IOU”: W. E. B. Du Bois, the Gift of Black Music and the Cultural Politics of Obligation	-EXCLUDE on intervention (service engagement/helpseeking behaviour)	
####	Hefner (2019) (ID:87850764)	Multimorbidity among veterans diagnosed with PTSD in the Veterans Health Administration nationally.	-EXCLUDE on intervention (service engagement/helpseeking behaviour)	
####	Hein (2018) (ID:87852889)	Policy brief: Protecting vulnerable LGBTQ youth and advocating for ethical health care	-EXCLUDE on evidence and form (evidence not in written form or presented as research output)	
####	HEINZE (2010) (ID:87856991)	Taking the youth perspective: assessment of program characteristics that promote positive development in homeless and at-risk youth	-EXCLUDE on population (no gender focus; women population <50)	
####	HEINZE (2012) (ID:87851437)	Reasons for homelessness: an empirical typology	-EXCLUDE on intervention (service engagement/helpseeking behaviour)	
####	Helfrich (2011) (ID:87849159)	Trauma symptoms of individuals with mental illness at risk for homelessness participating in a life skills intervention.	-EXCLUDE on intervention (service engagement/helpseeking behaviour)	
####	Helfrich (2012) (ID:87849155)	Readiness-to-change cluster profiles among adults with mental illness who were homeless participating in a life skills intervention.	-EXCLUDE on population (women)	
####	Hemminger (2021) (ID:87851970)	Growing Good: A Beginner's Guide to Cultivating Caring Communities	-EXCLUDE on evidence and form (evidence not in written form or presented as research output)	
####	HENDERSON (2017) (ID:87857189)	Relationships between anti-stigma programme awareness, disclosure comfort and intended help-seeking regarding a mental health problem	-EXCLUDE on population (no housing precarity)	
####	Henderson (2019) (ID:87852929)	Housing and food stress among transgender adults in the United States	-EXCLUDE on intervention (service engagement/helpseeking behaviour)	

####	Henderson (2019) (ID:87857779)	When Stereotypes Speak Louder Than Our Words: Black Female Doctoral Students and Their Experience with Race, Invisibility, and Self-Censorship at a Predominantly White Institution	-EXCLUDE on population (no housing precarity)	
####	Henne (2014) (ID:87858080)	Measure of a Man: A Grounded Theory Approach to Understanding Gay/Queer College Men's Self Identified Masculinity	-EXCLUDE on population (women)	
####	Hennein (2020) (ID:87848257)	A hybrid inductive-abductive analysis of health workers' experiences and wellbeing during the COVID-19 pandemic in the United States.	-EXCLUDE on population (no housing precarity)	
####	Henning (2014) (ID:87848981)	Asymptomatic Mycoplasma genitalium infection amongst marginalised young people accessing a youth health service in Melbourne.	-EXCLUDE on intervention (service engagement/helpseeking behaviour)	
####	Henning (2021) (ID:87848238)	Homelessness, sex and a tale of two sexually transmitted infections.	-EXCLUDE on intervention (service engagement/helpseeking behaviour)	
####	Henriques (2022) (ID:87857474)	Counter-Narratives of Structural Oppressions, Stigma and Resistance, and Reproductive and Sexual Health Among Youth Experiencing Homelessness	-EXCLUDE on population (no gender focus; women population <50)	
####	Henry (2010) (ID:87849273)	Mental disorders among homeless people admitted to a French psychiatric emergency service.	-EXCLUDE on intervention (service engagement/helpseeking behaviour)	
####	**Henry (2022) (ID:87857431)**	**Technology-Facilitated Domestic Violence Against Immigrant and Refugee Women: A Qualitative Study**	**-INCLUDE on title & abstract**	**-INCLUDE on full study**
####	Henwood (2013) (ID:87851952)	Addressing Chronic Disease Within Supportive Housing Programs	-EXCLUDE on intervention (service engagement/helpseeking behaviour)	
####	Henwood (2014) (ID:87848917)	Quality of life after housing first for adults with serious mental illness who have experienced chronic homelessness.	-EXCLUDE on population (women)	
####	Henwood (2014) (ID:87848992)	Provider views of harm reduction versus abstinence policies within homeless services for dually diagnosed adults.	-EXCLUDE on intervention (service engagement/helpseeking behaviour)	
####	Henwood (2015) (ID:87848892)	Maslow and mental health recovery: a comparative study of homeless programs for adults with serious mental illness.	-EXCLUDE on intervention (service engagement/helpseeking behaviour)<br>-EXCLUDE - but review for literature	
####	Henwood (2018) (ID:87848518)	Understanding Risk Environments in Permanent Supportive Housing for Formerly Homeless Adults.	-EXCLUDE on intervention (service engagement/helpseeking behaviour)	
####	Henwood (2021) (ID:87848209)	Investigating Sleep Disturbance and Its Correlates Among Formerly Homeless Adults in Permanent Supportive Housing.	-EXCLUDE on intervention (service engagement/helpseeking behaviour)	
####	Herman (2011) (ID:87849182)	Randomized trial of critical time intervention to prevent homelessness after hospital discharge.	-EXCLUDE on intervention (service engagement/helpseeking behaviour)	
####	Herman (2015) (ID:87848828)	Food Insecurity and Cost-Related Medication Underuse Among Nonelderly Adults in a Nationally Representative Sample.	-EXCLUDE on population (no housing precarity)	
####	Hermann (2021) (ID:87851517)	Monitoring trends in psychosocial and physical working conditions: Challenges and suggestions for the 21st century	-EXCLUDE on population (no housing precarity)	
####	Hermaszewska (2022) (ID:87857925)	Lived experiences of transgender forced migrants and their mental health outcomes: systematic review and meta-ethnography	-EXCLUDE on population (no housing precarity)	
####	Hernandez (2015) (ID:87857598)	An integrated mobile health team for LGBT homeless adults in Los Angeles County: A grant proposal	-EXCLUDE on evidence and form (evidence not in written form or presented as research output)	
####	Hernandez (2020) (ID:87848279)	Urban Stress Indirectly Influences Psychological Symptoms through Its Association with Distress Tolerance and Perceived Social Support among Adults Experiencing Homelessness.	-EXCLUDE on intervention (service engagement/helpseeking behaviour)	
####	Hernández-Saca (2018) (ID:87963308)	Intersectionality dis/ability research: How dis/ability research in education engages intersectionality to uncover the multidimensional construction of dis/abled experiences	-EXCLUDE on population (no housing precarity)	
####	Hersh (2011) (ID:87849174)	Integrating buprenorphine treatment into a public healthcare system: the San Francisco Department of Public Health's office-based Buprenorphine Pilot Program.	-EXCLUDE on intervention (service engagement/helpseeking behaviour)	
####	Heslin (2011) (ID:87849226)	Alternative families in recovery: fictive kin relationships among residents of sober living homes.	-EXCLUDE on population (no housing precarity)	
####	Heslop (2014) (ID:87852851)	The Confidential Inquiry into premature deaths of people with intellectual disabilities in the UK: a population-based study	-EXCLUDE on population (no housing precarity)	

####	Hess (2012) (ID:87963313)	Intersectionality: a systematic review and application to explore the complexity of teen pregnancy involvement	-EXCLUDE on population (no housing precarity)	
####	Hesse-Biber (2011) (ID:87963220)	Handbook of feminist research: Theory and praxis	-EXCLUDE on evidence and form (evidence not in written form or presented as research output)	
####	Hessol (2019) (ID:87848381)	A longitudinal study assessing differences in causes of death among housed and homeless people diagnosed with HIV in San Francisco.	-EXCLUDE on intervention (service engagement/helpseeking behaviour)	
####	Hickling (2011) (ID:87849199)	Deinstitutionalization and attitudes toward mental illness in Jamaica: a qualitative study.	-EXCLUDE on country (High-Income)	
####	Hicks (2014) (ID:87853405)	Association Between Race and Age in Survival After Trauma	-EXCLUDE on population (no housing precarity)	
####	Hill (2020) (ID:87852913)	Work2Prevent, an Employment Intervention Program as HIV Prevention for Young Men Who Have Sex With Men and Transgender Youth of Color (Phase 3): Protocol for a Single-Arm Community-Based Trial to Assess Feasibility and Acceptability in a Real-World Setting	-EXCLUDE on intervention (service engagement/helpseeking behaviour)	
####	Hill (2020) (ID:87853059)	An Employment Intervention Program (Work2Prevent) for Young Men Who Have Sex With Men and Transgender Youth of Color (Phase 1): Protocol for Determining Essential Intervention Components Using Qualitative Interviews and Focus Groups	-EXCLUDE on population (women)	
####	HILL (2021) (ID:87857156)	Seeking an anchor in an unstable world: experiences of low-income families over time	-EXCLUDE on population (no housing precarity)	
####	Hill (2022) (ID:87852779)	An examination of housing interventions among youth experiencing homelessness: an investigation into racial/ethnic and sexual minority status	-EXCLUDE on intervention (service engagement/helpseeking behaviour)	
####	Hill (2023) (ID:87857502)	Illicit drug use among lesbian, gay, bisexual, pansexual, trans and gender diverse, queer and asexual young people in Australia: Intersections and associated outcomes	-EXCLUDE on population (people aged under 18 years)	
####	Hill (2023) (ID:87857763)	Demographic and psychosocial factors associated with recent suicidal ideation and suicide attempts among trans and gender diverse people in Australia	-EXCLUDE on population (no housing precarity)	
####	Hill (2023) (ID:87857789)	Examining the Pathways to Crime in Previously Incarcerated Black Women in the U.S. and Their Perspectives on Mental Health Intervention	-EXCLUDE on intervention (service engagement/helpseeking behaviour)<br>-EXCLUDE - but review for literature	
####	HILLER (2021) (ID:87857382)	Coping and support-seeking in out-of-home care: a qualitative study of the views of young people in care in England	-EXCLUDE on population (no housing precarity)	
####	Himelhoch (2012) (ID:87849133)	Antidepressant prescribing patterns among VA patients with schizophrenia.	-EXCLUDE on intervention (service engagement/helpseeking behaviour)	
####	Hines (2020) (ID:87963326)	My whole world changed: A qualitative exploration of the coping experiences of transgender women after an HIV diagnosis	-EXCLUDE on population (no housing precarity)	
####	Hino (2018) (ID:87848488)	Tuberculosis control from the perspective of health professionals working in street clinics.	-EXCLUDE on intervention (service engagement/helpseeking behaviour)	
####	Hinton (2015) (ID:87857931)	Tri Squared Qualitative and Mixed Methods Analysis of Perceptions of the Effectiveness of the Student Athlete Leadership Academy [SALA]: A Character Development and College Preparatory Program for Young African American Men	-EXCLUDE on population (women)	
####	Hipp (2019) (ID:87857494)	From Conversion Toward Affirmation: Psychology, Civil Rights, and Experiences of Gender-Diverse Communities in Memphis	-EXCLUDE on population (no housing precarity)	
####	Hirani (2019) (ID:87848449)	Maternal and Child Health During Forced Displacement.	-EXCLUDE on intervention (service engagement/helpseeking behaviour)	
####	Hithersay (2019) (ID:87853016)	Association of Dementia With Mortality Among Adults With Down Syndrome Older Than 35 Years	-EXCLUDE on population (no housing precarity)	
####	Hlongwa (2019) (ID:87853284)	Demographic and socioeconomic factors associated with under-5 mortality in KwaZulu-Natal, South Africa	-EXCLUDE on country (High-Income)	
####	Ho (2015) (ID:87848857)	Integrated Care Increases Treatment and Improves Outcomes of Patients With Chronic Hepatitis C Virus Infection and Psychiatric Illness or Substance Abuse.	-EXCLUDE on intervention (service engagement/helpseeking behaviour)	

####	Hodges (2011) (ID:87849160)	Factors that impact help-seeking among battered Black women: application of critical and survivor theories.	-INCLUDE on title & abstract	-EXCLUDE (IPV/DVA but little discussion on housing)
####	Hodges (2015) (ID:87857910)	Contextualizing Multicultural Visions from the Foot of the Mountain	-EXCLUDE on population (no housing precarity)	
####	Hodges (2017) (ID:87963289)	An exploration of decision making by women experiencing multiple and complex needs	-INCLUDE on title & abstract	-EXCLUDE on intervention (intersectionality)
####	Hodgson (2015) (ID:87848864)	Psychopathology among young homeless people: longitudinal mental health outcomes for different subgroups.	-EXCLUDE on population (people aged under 18 years)	
####	Hoefinger (2020) (ID:87852939)	Community-Based Responses to Negative Health Impacts of Sexual Humanitarian Anti-Trafficking Policies and the Criminalization of Sex Work and Migration in the US	-INCLUDE on title & abstract	-EXCLUDE on intervention (service engagement/helpseeking behaviour)
####	Hoffart (2016) (ID:87857824)	Domestic Violence and Intergenerational Trauma Amongst Aboriginal Women in Regina, Saskatchewan	-INCLUDE on title & abstract	-EXCLUDE (IPV/DVA but little discussion on housing)
####	Hoffmann (2017) (ID:87850898)	Mental health of children who work on the streets in Brazil after enrollment in a psychosocial program.	-EXCLUDE on country (High-Income)	
####	Hoffmire (2021) (ID:87963145)	Accelerating research on suicide risk and prevention in women veterans through research-operations partnerships	-EXCLUDE on population (no housing precarity)	
####	Hogan (2012) (ID:87852806)	Barriers to women's participation in inter-conceptional care: a cross-sectional analysis	-EXCLUDE on population (no housing precarity)	
####	HOGAN (2021) (ID:87857114)	Men's experiences of help-seeking for female-perpetrated intimate partner violence: A qualitative exploration	-EXCLUDE on population (women)	
####	HOGG (2015) (ID:87857012)	An unstable start: all babies count: spotlight on homelessness	-EXCLUDE on population (people aged under 18 years)	
####	Hohman (2013) (ID:87851200)	What do female clients want from residential treatment? The relationship between expressed and assessed needs, psychosocial characteristics, and program outcome.	-EXCLUDE on intervention (service engagement/helpseeking behaviour)	
####	Holcomb (2010) (ID:87851371)	A phenomenological study of how homeless single mothers choose social support.	-INCLUDE on title & abstract	-EXCLUDE on target group (no housing precarity)
####	HOLGER-AMBROSE (2013) (ID:87857063)	The illusions and juxtapositions of commercial sexual exploitation among youth: identifying effective street-outreach strategies	-INCLUDE on title & abstract	-EXCLUDE on intervention (intersectionality)
####	Holliday (2020) (ID:87857745)	Racial/Ethnic Disparities in Police Reporting for Partner Violence in the National Crime Victimization Survey and Survivor-Led Interpretation	-EXCLUDE on intervention (service engagement/helpseeking behaviour)	
####	Holliday (2023) (ID:87851584)	Research Letter: Traumatic Brain Injury Among Veterans Accessing VA Justice-Related Services	-EXCLUDE on intervention (service engagement/helpseeking behaviour)	
####	HOLLIDGE (2016) (ID:87857201)	Seeking security in the face of fear: the disorganized dilemma	-EXCLUDE on intervention (service engagement/helpseeking behaviour)	
####	Holloway (2021) (ID:87852801)	Structural Syndemics and Antiretroviral Medication Adherence Among Black Sexual Minority Men Living With HIV	-EXCLUDE on population (women)	
####	Holst (2022) (ID:87857659)	Experiences of menstrual inequity and menstrual health among women and people who menstruate in the Barcelona area (Spain): a qualitative study	-EXCLUDE on population (no housing precarity)	
####	Holt (2015) (ID:87848811)	Using Masculine Capital to Understand the Role of a Sport Program in the Lives of Men From a Western Canadian Inner City.	-EXCLUDE on population (women)	
####	HOLTTUM (2015) (ID:87857237)	Students, inclusion, help-seeking and compassionate caring	-EXCLUDE on population (no housing precarity)	
####	Holtyn (2017) (ID:87850870)	An intensive assessment of alcohol use and emergency department utilization in homeless alcohol-dependent adults.	-EXCLUDE on intervention (service engagement/helpseeking behaviour)	
####	Holzhauer (2019) (ID:87850757)	Profiles of clinical need among homeless individuals with dual diagnoses.	-EXCLUDE on intervention (service engagement/helpseeking behaviour)	
####	Hom (2018) (ID:87848490)	Increased Health and Social Vulnerability Among Hepatitis C Infected Individuals Co-infected with Hepatitis B.	-EXCLUDE on intervention (service engagement/helpseeking behaviour)	
####	Home Wasn't Built in... (Martin) (ID:87849364)	Home Wasn't Built in a Day: A Homeless Health Outreach Team Success Story	-EXCLUDE on date (2010)	
####	HOMELESS (2010) (ID:87856999)	Improving homeless Londoners' mental health	-EXCLUDE on intervention (service engagement/helpseeking behaviour)	
####	HOMELESS (2015) (ID:87857045)	Social networks in hostels: practice briefing for frontline staff	-EXCLUDE on intervention (service engagement/helpseeking behaviour)	

####	Homeless (2018) (ID:88019157)	Promising practice from the frontline - Exploring gendered approaches to supporting women experiencing homelessness and multiple disadvantage	-INCLUDE on title & abstract	-EXCLUDE on intervention (intersectionality)
####	Homeless (2020) (ID:88019167)	Learning from the Ending Women's Homelessness Fund. Case Studies March-July 2020	-INCLUDE on title & abstract	-EXCLUDE on intervention (service engagement/helpseeking behaviour)
####	Homelesslink (2022) (ID:88019115)	Exploring women's homelessness What we know	-INCLUDE on title & abstract	-EXCLUDE on intervention (service engagement/helpseeking behaviour)
####	Homelessness, Unsheltered... (Montgomery) (ID:87851571)	Homelessness, Unsheltered Status, and Risk Factors for Mortality: Findings From the 100 000 Homes Campaign	-EXCLUDE on intervention (service engagement/helpseeking behaviour)	
####	Honer (2017) (ID:87848663)	The Hotel Study-Clinical and Health Service Effectiveness in a Cohort of Homeless or Marginally Housed Persons.	-EXCLUDE on intervention (service engagement/helpseeking behaviour)	
####	HONG (2018) (ID:87857002)	Applications of machine learning methods to predict readmission and length-of-stay for homeless families: the case of win shelters in New York City	-EXCLUDE on intervention (service engagement/helpseeking behaviour)	
####	Hooyman (2021) (ID:87850613)	Living through loss: Interventions across the life span.	-EXCLUDE on intervention (service engagement/helpseeking behaviour)	
####	HOPKINS (2006) (ID:87857185)	Settle down now	-EXCLUDE on date (2010)	
####	Hopkins (2015) (ID:87857825)	Transitions to religious adulthood: relational geographies of youth, religion and international volunteering	-EXCLUDE on population (no housing precarity)	
####	HORDYK (2014) (ID:87857082)	Sometimes you have to go under water to come up: a poetic, critical realist approach to documenting the voices of homeless immigrant women	-EXCLUDE on intervention (service engagement/helpseeking behaviour)	
####	Horowitz (2023) (ID:87857484)	Feminized need and racialized danger: Punitive therapeutics and historical addict tropes in a Midwestern drug court	-EXCLUDE on intervention (service engagement/helpseeking behaviour)	
####	Horvitz-Lennon (2011) (ID:87849188)	Racial and ethnic service use disparities among homeless adults with severe mental illnesses receiving ACT.	-EXCLUDE on intervention (service engagement/helpseeking behaviour)	
####	Hosang (2023) (ID:87963247)	Protocol for secondary data analysis of youth adversity and mental health in the context of intersectionality	-EXCLUDE on intervention (service engagement/helpseeking behaviour)	
####	House Financial Services... (2019) (ID:87851913)	House Financial Services Subcommittee on Housing, Community Development, and Insurance Hearing	-EXCLUDE on evidence and form (evidence not in written form or presented as research output)	
####	House Judiciary Subcommittee... (2021) (ID:87851887)	House Judiciary Subcommittee Issues Testimony From University of Memphis Assistant Professor Mason	-EXCLUDE on evidence and form (evidence not in written form or presented as research output)	
####	Housing: participation... (1998) (ID:87857057)	Housing: participation and exclusion; collected papers from the Socio-Legal Studies Annual Conference 1997, University of Wales, Cardiff	-EXCLUDE on date (2010)	
####	HOWARD (2019) (ID:87857144)	Relationship separation and help-seeking: reporting on an agency study	-EXCLUDE on population (no housing precarity)	
####	Howard (2021) (ID:87853145)	Demographic Factors Associated With Non-Guideline-Based Treatment of Kidney Cancer in the United States	-EXCLUDE on population (no housing precarity)	
####	Howell (2011) (ID:87850530)	From darkness turning to light : a study of spirituality in homeless African American women	-INCLUDE on title & abstract	-EXCLUDE on intervention (service engagement/helpseeking behaviour)
####	HOWERTON (2007) (ID:87857179)	Understanding help seeking behaviour among male offenders: qualitative interview study	-EXCLUDE on date (2010)	
####	Hrabok (2020) (ID:87848278)	Canadian Refugee Women Are at Increased Risk of Postpartum Depression: How Can We Help?.	-EXCLUDE on intervention (service engagement/helpseeking behaviour)	
####	Hsieh (2019) (ID:87852998)	Characteristics of long-term survivors with multiple myeloma: A National Cancer Data Base analysis	-EXCLUDE on population (no housing precarity)	
####	Hsu (2015) (ID:87848850)	Understanding Consistent Condom Use Among Homeless Men Who Have Sex with Women and Engage in Multiple Sexual Partnerships: A Path Analysis.	-EXCLUDE on population (women)	
####	Hsu (2018) (ID:87848534)	But Everyone is Doing It (Sort of)! Perceived Sexual Risks in the Social Environment and the Impact on Homeless Youth Engagement in Concurrent Sexual Relationships.	-EXCLUDE on intervention (service engagement/helpseeking behaviour)	
####	Hsu (2019) (ID:87850733)	Understanding wait times in rapid re-housing among homeless youth: A competing risk survival analysis.	-EXCLUDE on intervention (service engagement/helpseeking behaviour)	
####	Hsu (2020) (ID:87848284)	Race/Ethnicity, Underlying Medical Conditions, Homelessness, and Hospitalization Status of Adult Patients with COVID-19 at an Urban Safety-Net Medical Center - Boston, Massachusetts, 2020.	-EXCLUDE on intervention (service engagement/helpseeking behaviour)	

####	Huang (2013) (ID:87849083)	Employment outcomes of adults with cerebral palsy in Taiwan.	-EXCLUDE on country (High-Income)	
####	Huang (2020) (ID:87963200)	How intersectional are mental health interventions for sexual minority people? A systematic review	-EXCLUDE on population (no housing precarity)	
####	Huang (2023) (ID:87852884)	Risk of delivery complications among pregnant people experiencing housing insecurity	-EXCLUDE on intervention (service engagement/helpseeking behaviour)	
####	Hubberstey (2019) (ID:87851916)	Multi-Service Programs for Pregnant and Parenting Women with Substance Use Concerns: Women's Perspectives on Why They Seek Help and Their Significant Changes	-INCLUDE on title & abstract	-EXCLUDE on target group (no housing precarity)
####	Huber (2019) (ID:87848458)	HIV-positive in the darkness of a correctional facility: more vulnerable and less treated.	-EXCLUDE on population (women)	
####	HUBY (1999) (ID:87857174)	A study of town life: living standards in the City of York 100 years after Rowntree	-EXCLUDE on date (2010)	
####	Hudon (2023) (ID:87857634)	The experience of pregnant women in contexts of vulnerability of prenatal primary nursing care: a descriptive interpretative qualitative study	-EXCLUDE on population (no housing precarity)	
####	Hudson (2010) (ID:87849261)	Health-seeking challenges among homeless youth.	-EXCLUDE on population (people aged under 18 years)	
####	Hudson (2012) (ID:87851222)	Comparisons of substance abuse, high-risk sexual behavior and depressive symptoms among homeless youth with and without a history of foster care placement.	-EXCLUDE on intervention (service engagement/helpseeking behaviour)	
####	Hudson (2018) (ID:87850816)	Identity-conscious services for diverse patients: A descriptive analysis of lesbian, gay, bisexual, and transgender-focused federally qualified community health centers.	-EXCLUDE on intervention (service engagement/helpseeking behaviour)	
####	Hudson (2022) (ID:87857837)	"That is when I changed and my whole life changed": Turning points in health perceptions among LGBTQ adults of color	-EXCLUDE on population (no housing precarity)	
####	Hudson (2023) (ID:87857777)	Nonclinical best practices for creating LGBTQ-inclusive care environments: A scoping review of gray literature	-EXCLUDE on population (no housing precarity)	
####	HUDSON-SHARP (2016) (ID:87851426)	Inequality among lesbian, gay bisexual and transgender groups in the UK: a review of evidence	-EXCLUDE on intervention (service engagement/helpseeking behaviour)	
####	Huey (2010) (ID:87851403)	False security or greater social inclusion? Exploring perceptions of CCTV use in public and private spaces accessed by the homeless.	-EXCLUDE on intervention (service engagement/helpseeking behaviour)	
####	Huey (2012) (ID:87852751)	"I Need Help and I Know I Need Help. Why Won't Nobody Listen to Me?" Trauma and Homeless Women's Experiences with Accessing and Consuming Mental Health Services	-INCLUDE on title & abstract	-EXCLUDE on intervention (intersectionality)
####	HUEY (2013) (ID:87856958)	"If something happened, I will leave it, let it go and move on": resiliency and victimized homeless women's attitudes toward mental health counseling	-INCLUDE on title & abstract	-EXCLUDE on intervention (intersectionality)
####	Huey (2014) (ID:87848897)	"They just asked me why I became homeless": "failure to ask" as a barrier to homeless women's ability to access services post-victimization.	-INCLUDE on title & abstract	-EXCLUDE on intervention (intersectionality)
####	Hufnagel (2021) (ID:87857444)	Rearticulating a New Poor People's Campaign: Fifty Years of Grassroots Anti-Poverty Movement Organizing	-EXCLUDE on intervention (service engagement/helpseeking behaviour)	
####	Huggan (2010) (ID:87853116)	Population-based epidemiology of Staphylococcus aureus bloodstream infection in Canterbury, New Zealand	-EXCLUDE on intervention (service engagement/helpseeking behaviour)	
####	Huggett (2021) (ID:87848176)	Assessment of a Hotel-Based Protective Housing Program for Incidence of SARS-CoV-2 Infection and Management of Chronic Illness Among Persons Experiencing Homelessness.	-EXCLUDE on intervention (service engagement/helpseeking behaviour)	
####	Hulsey (2023) (ID:87858091)	Stigmatizing imagery for substance use disorders: a qualitative exploration	-EXCLUDE on population (no housing precarity)	
####	Hunt (2010) (ID:87849275)	Suicide amongst psychiatric in-patients who abscond from the ward: a national clinical survey.	-EXCLUDE on intervention (service engagement/helpseeking behaviour)	
####	HUNTER (2003) (ID:87857275)	Forgotten act	-EXCLUDE on date (2010)	
####	Hunter (2015) (ID:87848880)	Barriers to Prescription Medication Adherence Among Homeless and Vulnerably Housed Adults in Three Canadian Cities.	-EXCLUDE on population (women)	
####	HUNTLEY (2019) (ID:87857211)	Help-seeking by male victims of domestic violence and abuse (DVA): a systematic review and qualitative evidence synthesis	-EXCLUDE on population (women)	

####	HUNTLEY (2020) (ID:87857259)	Help seeking by male victims of domestic violence and abuse: an example of an integrated mixed methods synthesis of systematic review evidence defining methodological terms	-EXCLUDE on population (women)
####	Huq (2021) (ID:87852893)	DIY Entrepreneurship? - Self-reliance for Women Refugees in Australia	-EXCLUDE on population (no housing precarity)
####	Hurly (2019) (ID:87852903)	'I feel something is still missing': leisure meanings of African refugee women in Canada	-EXCLUDE on intervention (service engagement/helpseeking behaviour)
####	HURT (2012) (ID:87857194)	Why don't older adults with subjective memory complaints seek help?	-EXCLUDE on population (no housing precarity)
####	Hurt (2022) (ID:87848159)	Beyond Disease Intervention: Exploring an Expanded Role for Partner Services in the MATRix-NC Demonstration Project.	-EXCLUDE on population (no housing precarity)
####	Hurtado (2015) (ID:87857921)	Gloria anzaldúa's seven stages of "conocimiento" in redefining latino masculinity: José's story	-EXCLUDE on population (women)
####	Husain (2022) (ID:87857433)	Desperately Seeking Intersectionality in Digital Health Disparity Research: Narrative Review to Inform a Richer Theorization of Multiple Disadvantage	-EXCLUDE on population (women)<br>-EXCLUDE - but review for literature
####	Hussaini (2022) (ID:87852786)	Mental health impacts of earthquake on Afghans amidst humanitarian crisis	-EXCLUDE on country (High-Income)
####	Huynh (2022) (ID:87853008)	Personalised multicomponent interventions for tobacco dependence management in low socioeconomic populations: a systematic review and meta-analysis	-EXCLUDE on intervention (service engagement/helpseeking behaviour)
####	Hwang (2010) (ID:87849251)	Universal health insurance and health care access for homeless persons.	-EXCLUDE on population (no gender focus; women population <50)
####	Hwang (2011) (ID:87849187)	Health status, quality of life, residential stability, substance use, and health care utilization among adults applying to a supportive housing program.	-EXCLUDE on intervention (service engagement/helpseeking behaviour)
####	Hwang (2011) (ID:87851345)	Hospital costs and length of stay among homeless patients admitted to medical, surgical, and psychiatric services.	-EXCLUDE on intervention (service engagement/helpseeking behaviour)
####	Hwang (2013) (ID:87849002)	A comprehensive assessment of health care utilization among homeless adults under a system of universal health insurance.	-EXCLUDE on intervention (service engagement/helpseeking behaviour)
####	Hwang (2016) (ID:87848796)	Accuracy of Self-Reported Health Care Use in a Population-Based Sample of Homeless Adults.	-EXCLUDE on intervention (service engagement/helpseeking behaviour)
####	HYLAND (2015) (ID:87857242)	A test of the inventory of attitudes towards seeking mental health services	-EXCLUDE on population (no housing precarity)
####	Hyman (2011) (ID:87851310)	Exit strategies: Testing ecological prediction models of resilient outcomes in youth with histories of homelessness.	-EXCLUDE on intervention (service engagement/helpseeking behaviour)
####	Hyman (2011) (ID:87851354)	Resilient educational outcomes: Participation in school by youth with histories of homelessness.	-EXCLUDE on evidence and form (evidence not in written form or presented as research output)
####	Hyman (2013) (ID:87851204)	"Resilient educational outcomes: Participation in school by youth with histories of homelessness": Corrigendum.	-EXCLUDE on evidence and form (evidence not in written form or presented as research output)
####	Hyshka (2017) (ID:87848699)	Perceived unmet need and barriers to care amongst street-involved people who use illicit drugs.	-EXCLUDE on population (no gender focus; women population <50)
####	Iacono (2019) (ID:87848475)	An exploration of communication within active support for adults with high and low support needs.	-EXCLUDE on population (no housing precarity)
####	Iacono (2020) (ID:87848318)	A prospective study of hospital episodes of adults with intellectual disability.	-EXCLUDE on population (no housing precarity)
####	Iancu (2014) (ID:87848986)	Mental health recovery on care farms and day centres: a qualitative comparative study of users' perspectives.	-EXCLUDE on population (no housing precarity)
####	Ibabe (2014) (ID:87848972)	Predictors of substance abuse treatment participation among homeless adults.	-EXCLUDE on population (women)
####	Ibañez (2021) (ID:87857859)	Incarceration History and HIV Care Among Individuals Living with HIV in Florida, 2014–2018	-EXCLUDE on population (no housing precarity)
####	Ibarra-Castillo (2018) (ID:87853128)	Survival in relation to multimorbidity patterns in older adults in primary care in Barcelona, Spain (2010-2014): a longitudinal study based on electronic health records	-EXCLUDE on intervention (service engagement/helpseeking behaviour)
####	Idemudia (2013) (ID:87849085)	Migration challenges among Zimbabwean refugees before, during and post arrival in South Africa.	-EXCLUDE on country (High-Income)
####	IFF (2021) (ID:87857366)	Study of children joining family in England under the Dublin III Regulation	-EXCLUDE on population (people aged under 18 years)

####	Ignatia (2017) (ID:87851583)	Refugee children and adolescents in Greece: two case reports	-EXCLUDE on intervention (service engagement/helpseeking behaviour)	
####	Iheanacho (2020) (ID:87848304)	Mobile, Community-Based Buprenorphine Treatment for Veterans Experiencing Homelessness With Opioid Use Disorder: A Pilot, Feasibility Study.	-EXCLUDE on intervention (service engagement/helpseeking behaviour)	
####	Ijadi-Maghsoodi (2018) (ID:87852775)	Commercially sexually exploited youths' health care experiences, barriers, and recommendations: A qualitative analysis	-EXCLUDE on population (women)	
####	Ijadi-Maghsoodi (2021) (ID:87852891)	A Sector Wheel Approach to Understanding the Needs and Barriers to Services among Homeless-Experienced Veteran Families	-EXCLUDE on population (no gender focus; women population <50)	
####	Ijadunola (2020) (ID:87848287)	Reproductive health challenges of male and female street beggars and use of reproductive health services by female street beggars in Ife-Ijesa, Nigeria.	-EXCLUDE on country (High-Income)	
####	Ikkos (2021) (ID:87850608)	Mind, state and society: Social history of psychiatry and mental health in Britain 1960-2010.	-EXCLUDE on intervention (service engagement/helpseeking behaviour)	
####	Ilgen (2010) (ID:87853227)	Psychiatric Diagnoses and Risk of Suicide in Veterans	-EXCLUDE on intervention (service engagement/helpseeking behaviour)	
####	Illman (2013) (ID:87848994)	Exploring the occupations of homeless adults living with mental illnesses in Toronto.	-EXCLUDE on intervention (service engagement/helpseeking behaviour)	
####	Im (2021) (ID:87853412)	Falling Through the Cracks: Stress and Coping in Migration and Resettlement Among Marginalized Hmong Refugee Families in the United States	-EXCLUDE on population (no gender focus; women population <50)	
####	Imam (2017) (ID:87857827)	The devil is in the details: a feminist perspective on development, women's rights, and fundamentalisms	-EXCLUDE on population (no housing precarity)	
####	Impact Evaluation of... (Nelipovich) (ID:87849326)	Impact Evaluation of Patient-Centered, Community-Engaged Health Modules for Homeless Pregnant Women	-EXCLUDE on intervention (service engagement/helpseeking behaviour)	
####	Impact of volunteer-led... (Dawes) (ID:87851465)	Impact of volunteer-led running groups for women affected by homelessness: a qualitative study of the charity, A Mile in Her Shoes	-EXCLUDE on intervention (service engagement/helpseeking behaviour)<br>-EXCLUDE - but review for literature	
####	Improving inclusion... (NIHR) (ID:88019153)	Improving inclusion of under-served groups in clinical research: Guidance from INCLUDE project	-EXCLUDE on evidence and form (evidence not in written form or presented as research output)	
####	In your... (2016) (ID:87852006)	In your neighborhood	-EXCLUDE on evidence and form (evidence not in written form or presented as research output)	
####	Incidence of infant... (Tesema) (ID:87851513)	Incidence of infant mortality and its predictors in East Africa using Gompertz gamma shared frailty model	-EXCLUDE on country (High-Income)	
####	Indu (2018) (ID:87848598)	Determinants of compulsory admissions in a state psychiatric hospital-Case control study.	-EXCLUDE on country (High-Income)	
####	Infante (2012) (ID:87853167)	Violence Committed Against Migrants in Transit: Experiences on the Northern Mexican Border	-EXCLUDE on country (High-Income)	
####	Ingram (2019) (ID:87848469)	The Inner Working of Trauma: A Qualitative Assessment of Experiences of Trauma, Intergenerational Family Dynamics, and Psychological Well-Being in Women With HIV in South Carolina.	-EXCLUDE on intervention (service engagement/helpseeking behaviour)	
####	Insights and Impact... (Homeless) (ID:88019166)	Insights and Impact from the Ending Women's Homelessness Fund Led by Homeless Link	-EXCLUDE on intervention (service engagement/helpseeking behaviour)<br>-EXCLUDE - but review for literature	
####	INTRODUCTION: Thinking... (2016) (ID:87858068)	INTRODUCTION: Thinking beyond Hetero/Homo Normativities	-EXCLUDE on intervention (service engagement/helpseeking behaviour)	
####	Investigating reasons... (McKenzie) (ID:87851467)	Investigating reasons for socioeconomic inequalities in breast cancer survival in New Zealand	-EXCLUDE on population (no housing precarity)	
####	Iparraguirre (2020) (ID:87849305)	Economics and Ageing: Volume IV: Political Economy	-EXCLUDE on evidence and form (evidence not in written form or presented as research output)	
####	Iratzoqui (2020) (ID:87963372)	The reporting and help-seeking behaviors of domestic violence victims with criminal backgrounds	-INCLUDE on title & abstract	-EXCLUDE on intervention (intersectionality)<br>-EXCLUDE (systematic review)* only use with other code
####	Irazábal (2016) (ID:87963042)	Intersectionality and planning at the margins: LGBTQ youth of color in New York	-EXCLUDE on population (no housing precarity)<br>-EXCLUDE - but review for literature	

####	Irenso (2018) (ID:87852733)	Implications of Ethiopian Productive Safety Net Programme on household dietary diversity and women's body mass index: a cross-sectional study	-EXCLUDE on country (High-Income)	
####	Irestig (2010) (ID:87849278)	How are homeless people treated in the healthcare system and other societal institutions? Study of their experiences and trust.	-EXCLUDE on population (no gender focus; women population <50)	
####	Irvine-Collins (2023) (ID:87963266)	Promoting compassionate responses to disclosures of sexual violence in university settings: exploring the impact of a social marketing campaign	-EXCLUDE on population (no housing precarity)	
####	Isaacs (2019) (ID:87848434)	Unmet needs of persons with a severe and persistent mental illness and their relationship to unmet accommodation needs.	-EXCLUDE on intervention (service engagement/helpseeking behaviour)	
####	Isaak (2019) (ID:87848406)	The entrepreneurship of survival among urban adults experiencing homelessness and mental illness.	-EXCLUDE on population (women)	
####	Islami (2013) (ID:87852648)	Disentangling the effects of race/ethnicity and socioeconomic status of neighborhood in cancer stage distribution in New York City	-EXCLUDE on population (no housing precarity)	
####	Israel (2010) (ID:87849255)	Changes in the composition of the homeless population: 1992-2002.	-EXCLUDE on intervention (service engagement/helpseeking behaviour)	
####	ISSUES OF WOMEN HEALTH... (Mahmood) (ID:87851621)	ISSUES OF WOMEN HEALTH IN NON-MUSLIM COMMUNITY OF KARACHI, PAKISTAN: A QUALITATIVE SURVEY.	-EXCLUDE on country (High-Income)	
####	İSTANBUL'DA GÖÇMENLERİN... (Erdoğan) (ID:87851667)	İSTANBUL'DA GÖÇMENLERİN MEKÂNA YERLEŞME VE TUTUNMA DİNAMİKLERİ: BEYOĞLU ÖRNEĞİ. (Turkish)	-EXCLUDE on country (High-Income)	
####	Ito (2014) (ID:87852952)	Factors associated with mental well-being of homeless people in Japan	-EXCLUDE on population (women)	
####	Ito (2014) (ID:87853270)	Socioeconomic inequalities in cancer survival: A population-based study of adult patients diagnosed in Osaka, Japan, during the period 1993-2004	-EXCLUDE on population (no housing precarity)	
####	Ivany (2018) (ID:87857616)	Living in Fear and Prioritizing Safety: Exploring Women's Lives After Traumatic Brain Injury From Intimate Partner Violence	-INCLUDE on title & abstract	-EXCLUDE on target group (no housing precarity)
####	IVERSEN (2010) (ID:87857298)	Help-seeking and receipt of treatment among UK service personnel	-EXCLUDE on population (no housing precarity)	
####	Ives (2018) (ID:87851418)	Burning Britain? Tackling 'burning injustices' that blight Britain	-EXCLUDE on intervention (service engagement/helpseeking behaviour)	
####	Iwundu (2020) (ID:87852636)	Predictors of Overnight and Emergency Treatment among Homeless Adults	-EXCLUDE on population (no gender focus; women population <50)	
####	Jablonka (2017) (ID:87848694)	Tetanus and diphtheria immunity in refugees in Europe in 2015.	-EXCLUDE on intervention (service engagement/helpseeking behaviour)	
####	Jablonka (2017) (ID:87852995)	Hepatitis A immunity in refugees in Germany during the current exodus	-EXCLUDE on intervention (service engagement/helpseeking behaviour)	
####	Jackson (2014) (ID:87852020)	EXAMINING SOCIAL SUPPORT IN A RURAL HOMELESS POPULATION	-EXCLUDE on population (women)	
####	Jackson (2022) (ID:87850532)	Hitting home : exploring housing and home(lessness) in the context of domestic abuse	-INCLUDE on title & abstract	-EXCLUDE on intervention (intersectionality)
####	Jackson (2022) (ID:87851968)	Black, First-Generation, Underresourced College Students: Fighting the Dual Pandemics of COVID-19 and Police Brutality	-EXCLUDE on population (no housing precarity)	
####	Jacobsen (2022) (ID:88019129)	Project Lotus: A really cool community-based initiative assisting women post-homelessness	-INCLUDE on title & abstract	-EXCLUDE on intervention (service engagement/helpseeking behaviour)
####	Jacobsson (2021) (ID:87850636)	Doing human service ethnography.	-EXCLUDE on intervention (service engagement/helpseeking behaviour)	
####	Jacovides (2021) (ID:87858117)	Suicide in American Cities	-EXCLUDE on population (no housing precarity)	
####	Jaffe (2021) (ID:87857742)	Experimental (Re)structuring: The Clinical Trial as Turning Point Among Medical Research Participants	-EXCLUDE on population (no housing precarity)	
####	Jaffiol (2012) (ID:87849290)	Diabetes and social deprivation	-EXCLUDE on population (no housing precarity)	
####	Jafry (2021) (ID:87848181)	Perceived Social Support Attenuates the Association between Stress and Health-Related Quality of Life among Adults Experiencing Homelessness.	-EXCLUDE on intervention (service engagement/helpseeking behaviour)	
####	Jain (2018) (ID:87848538)	Post-Traumatic Stress Disorder, Neighborhood Residency and Satisfaction, and Social Network Characteristics among Underserved Women in Baltimore, Maryland.	-EXCLUDE on intervention (service engagement/helpseeking behaviour)	

####	Jakubovski (2015) (ID:87848808)	Prognostic subgroups for remission, response, and treatment continuation in the Clinical Antipsychotic Trials of Intervention Effectiveness (CATIE) trial.	-EXCLUDE on intervention (service engagement/helpseeking behaviour)
####	James (2019) (ID:87848371)	Creating conditions for a sense of security during the evenings and nights among older persons receiving home health care in ordinary housing: a participatory appreciative action and reflection study.	-EXCLUDE on population (no housing precarity)
####	James (2021) (ID:87851892)	Part III: External Reforms and the Duty to Protect	-EXCLUDE on intervention (service engagement/helpseeking behaviour)
####	Jamwal (2018) (ID:87848587)	Electronic assistive technology use in Australian shared supported accommodation: rates and user characteristics.	-EXCLUDE on intervention (service engagement/helpseeking behaviour)
####	Jamwal (2020) (ID:87848361)	The use of electronic assistive technology for social networking by people with disability living in shared supported accommodation.	-EXCLUDE on population (no housing precarity)
####	Jani (2019) (ID:87853381)	Relationship between multimorbidity, demographic factors and mortality: findings from the UK Biobank cohort	-EXCLUDE on intervention (service engagement/helpseeking behaviour)
####	Janika (2020) (ID:87851981)	"I need to take care of myself": a qualitative study on coping strategies, support and health promotion for social workers serving refugees and homeless individuals	-EXCLUDE on population (no gender focus; women population <50)<br>-EXCLUDE - but review for literature
####	Jannat (2022) (ID:87852949)	Sexual and Reproductive Health Conditions of Women: Insights from Rohingya Refugee Women in Bangladesh	-EXCLUDE on country (High-Income)
####	Janssens (2017) (ID:87848638)	Screening for tuberculosis in an urban shelter for homeless in Switzerland: a prospective study.	-EXCLUDE on intervention (service engagement/helpseeking behaviour)
####	Jantz (2023) (ID:87857771)	Lifting as we climb: Experiences and recommendations from women in neural engineering	-EXCLUDE on population (no housing precarity)
####	Jarrell (2020) (ID:87857728)	The Importance of Play: Identification with Video Game Characters' Intersectional Effects on Bias	-EXCLUDE on population (no housing precarity)
####	Jashnani (2020) (ID:87858102)	Dispossession by Accumulation: The Impacts of Discretionary Arrests in New York City	-EXCLUDE on population (no housing precarity)
####	Jason (2022) (ID:87850611)	Dynamic interdependence of advice seeking, loaning, and recovery characteristics in recovery homes.	-EXCLUDE on intervention (service engagement/helpseeking behaviour)
####	Jasuja (2021) (ID:87848210)	Homelessness and Veteran Status in Relation to Nonfatal and Fatal Opioid Overdose in Massachusetts.	-EXCLUDE on intervention (service engagement/helpseeking behaviour)
####	Javaherian-Dysinger (2011) (ID:87857781)	Occupational Therapy Services for Individuals Who Have Experienced Domestic Violence	-EXCLUDE on intervention (service engagement/helpseeking behaviour)
####	Javaherian-Dysinger (2017) (ID:87857861)	Occupational therapy services for individuals who have experienced domestic violence	-EXCLUDE on evidence and form (evidence not in written form or presented as research output)
####	Jefferson (2021) (ID:87857992)	A scoping review of recommendations in the English language on conducting research with trauma-exposed populations since publication of the Belmont report; thematic review of existing recommendations on research with trauma-exposed populations	-EXCLUDE on population (no housing precarity)
####	Jelinic (2021) (ID:87857664)	Navigating the family law provisions: Migrant women's voices	-EXCLUDE on intervention (service engagement/helpseeking behaviour)
####	Jennings (2023) (ID:87857577)	Mixed-Method Research Approach for Examining the Factors Affecting the Choice of Different Treatment Pathways by Homeless People in Baltimore, Maryland	-EXCLUDE on intervention (service engagement/helpseeking behaviour)
####	Jepsen (2019) (ID:87857874)	A Community Educational Workshop for Individuals Impacted by Domestic Violence in Romania: A Grant Proposal	-EXCLUDE on evidence and form (evidence not in written form or presented as research output)
####	Jesuthasan (2018) (ID:87848561)	Near-death experiences, attacks by family members, and absence of health care in their home countries affect the quality of life of refugee women in Germany: a multi-region, cross-sectional, gender-sensitive study.	-EXCLUDE on intervention (service engagement/helpseeking behaviour)<br>-EXCLUDE - but review for literature
####	Jha (2015) (ID:87850970)	Mother Child Unit (MCU) of a tertiary care institution: A first cry from India.	-EXCLUDE on country (High-Income)
####	Jiang (2020) (ID:87853033)	Prevalence and impact of eye disease in an urban homeless and marginally housed population	-EXCLUDE on intervention (service engagement/helpseeking behaviour)

####	Jiao (2022) (ID:87852972)	Exploring the conceptualization, operationalization, implementation, and measurement of outreach in community settings with hard-to-reach and hidden populations: A scoping review	-EXCLUDE on population (women)	
####	JINNAH (2008) (ID:87857267)	Parents' experiences in seeking child care for school age children with disabilities - where does the system break down	-EXCLUDE on date (2010)	
####	Jivanji (2021) (ID:87853215)	The Association Between Race and 5-year Survival in Patients With Clear Cell Renal Cell Carcinoma: A Cohort Study	-EXCLUDE on intervention (service engagement/helpseeking behaviour)	
####	Jiwatram-Negron (2015) (ID:87848838)	Correlates of Sex Trading among Drug-Involved Women in Committed Intimate Relationships: A Risk Profile.	-EXCLUDE on intervention (service engagement/helpseeking behaviour)	
####	Jiwatram-Negron (2016) (ID:87857689)	Invisible Intersections of Gender-Based Violence among High-Risk, Vulnerable Women in the U.S. and Kazakhstan	-EXCLUDE on intervention (service engagement/helpseeking behaviour)<br>-EXCLUDE - but review for literature	
####	Jiwatram-Negron (2019) (ID:87850769)	Overlapping intimate partner violence and sex trading among high-risk women: Implications for practice.	-INCLUDE on title & abstract	-EXCLUDE on intervention (service engagement/helpseeking behaviour)
####	JOBE (2013) (ID:87857364)	‘If kids don't feel safe they don't do anything’: young people's views on seeking and receiving help from Children's Social Care Services in England	-EXCLUDE on population (people aged under 18 years)	
####	Jobe (2019) (ID:87857890)	Carceral hermeneutics: Discovering the bible in prison and prison in the bible	-EXCLUDE on intervention (service engagement/helpseeking behaviour)	
####	Johns (2017) (ID:87850883)	Sociodemographic factors associated with trans*female youth's access to health care in the San Francisco Bay Area.	-EXCLUDE on population (people aged under 18 years)	
####	JOHNSON (2010) (ID:87856928)	Sexual self-concept in sexually abused homeless adolescents	-EXCLUDE on intervention (service engagement/helpseeking behaviour)	
####	Johnson (2011) (ID:87849178)	Cognitive behavioral treatment of PTSD in residents of battered women's shelters: results of a randomized clinical trial.	-EXCLUDE on intervention (service engagement/helpseeking behaviour)	
####	JOHNSON (2013) (ID:87857337)	Failure and delay in treatment-seeking across anxiety disorders	-EXCLUDE on population (no housing precarity)	
####	Johnson (2014) (ID:87857792)	Transgender college student activists: The intersections of identities	-EXCLUDE on population (no housing precarity)	
####	Johnson (2016) (ID:87848730)	Comparison of Adding Treatment of PTSD During and After Shelter Stay to Standard Care in Residents of Battered Women's Shelters: Results of a Randomized Clinical Trial.	-EXCLUDE on intervention (service engagement/helpseeking behaviour)	
####	Johnson (2017) (ID:87850850)	Religion, disability, and interpersonal violence.	-EXCLUDE on population (no housing precarity)	
####	Johnson (2017) (ID:87963381)	Domestic violence, liminality and precarity in the British Borderlands Polish women’s experiences of abuse and service engagement in Edinburgh	-INCLUDE on title & abstract	-EXCLUDE (IPV/DVA but little discussion on housing)
####	JOHNSON (2017) (ID:87857094)	Help seeking among adolescents in foster care: a qualitative study	-EXCLUDE on population (people aged under 18 years)	
####	Johnson (2020) (ID:87857821)	Is It Because I'm Black or a Woman? Constructing an Intersectional and Trauma-Informed Model of Social Support	-EXCLUDE on population (no housing precarity)	
####	Johnson (2020) (ID:87857917)	An Exploratory Study of Transgender Inmate Populations in Latin America	-EXCLUDE on country (High-Income)	
####	Johnson (2022) (ID:87848163)	Dental Service and Resource Needs during COVID-19 among Underserved Populations.	-EXCLUDE on intervention (service engagement/helpseeking behaviour)	
####	Johnson (2022) (ID:87857543)	Diversifying Indigenous Vulnerability and Adaptation: An Intersectional Reading of Māori Women’s Experiences of Health, Wellbeing, and Climate Change	-EXCLUDE on population (no housing precarity)	
####	Johnson (2022) (ID:87963037)	Trauma, Mental Health Distress, and Infectious Disease Prevention Among Women Recently Released From Incarceration	-EXCLUDE on intervention (service engagement/helpseeking behaviour)	
####	Johnson (2023) (ID:87851768)	Interventions to improve health and the determinants of health among sex workers in high-income countries: a systematic review.	-INCLUDE on title & abstract	-EXCLUDE (IPV/DVA but little discussion on housing)<br>-EXCLUDE (systematic review)* only use with other code
####	Johnson (2023) (ID:87853352)	Lifetime medical costs attributable to sickle cell disease among nonelderly individuals with commercial insurance	-EXCLUDE on population (no housing precarity)	
####	Johnson (2023) (ID:87857808)	Community member perspectives on adapting the cascade of care for opioid use disorder for a tribal nation in the United States	-EXCLUDE on population (no housing precarity)	

####	Johnston (2016) (ID:87852849)	In Their Own Voices: The Reproductive Health Care Experiences of Detained Adolescent Girls	-EXCLUDE on population (people aged under 18 years)	
####	Johnston (2019) (ID:87848379)	A Profile of the Health of Homeless at an Inner City Primary Health Care Clinic in South Africa.	-EXCLUDE on country (High-Income)	
####	JOHNSTON (2020) (ID:87857246)	Preliminary validation of the parental help-seeking stage of change measure for child behavior problems	-EXCLUDE on population (no housing precarity)	
####	Jones (2012) (ID:87857702)	Women Reading for Education, Affinity & Development (WREAD): An evaluation of a semistructured reading discussion group for African American female adult-literacy students with histories of trauma	-EXCLUDE on population (no housing precarity)	
####	Jones (2017) (ID:87848643)	A national evaluation of homeless and nonhomeless veterans' experiences with primary care.	-EXCLUDE on population (women)	
####	JONES (2017) (ID:87857224)	Enablers of help-seeking for deaf and disabled children following abuse and barriers to protection: a qualitative study	-EXCLUDE on population (people aged under 18 years)	
####	Jones (2018) (ID:87848528)	Differences in Experiences With Care Between Homeless and Nonhomeless Patients in Veterans Affairs Facilities With Tailored and Nontailored Primary Care Teams.	-EXCLUDE on intervention (service engagement/helpseeking behaviour)	
####	Jones (2018) (ID:87851627)	Patient predictors and utilization of health services within a medical home for homeless persons.	-EXCLUDE on intervention (service engagement/helpseeking behaviour)	
####	Jones (2018) (ID:87963357)	“Invincible Black women”: Group therapy for Black college women	-EXCLUDE on population (no housing precarity)	
####	Jones (2019) (ID:87848441)	Providing Positive Primary Care Experiences for Homeless Veterans Through Tailored Medical Homes: The Veterans Health Administration's Homeless Patient Aligned Care Teams.	-EXCLUDE on intervention (service engagement/helpseeking behaviour)	
####	Jones (2019) (ID:87857894)	"HIV Lives with Me": An Intersectional Analysis of the Successful Navigation of Health Care Services by Sex Workers Living with HIV	-INCLUDE on title & abstract	-EXCLUDE on target group (no housing precarity)
####	Jones (2020) (ID:87848322)	Resurgent Methamphetamine Use at Treatment Admission in the United States, 2008-2017.	-EXCLUDE on intervention (service engagement/helpseeking behaviour)	
####	Jones (2020) (ID:87853331)	Implementation of the INTERGROWTH-21(st)gestational dating and fetal and newborn growth standards in Nairobi, Kenya: women's experiences with ultrasound and newborn assessment Women's experiences with ultrasound and newborn assessment in peri-urban Kenya	-EXCLUDE on country (High-Income)	
####	Jones (2021) (ID:87850588)	Patterns and predictors of reincarceration among prisoners with serious mental illness: A cohort study.	-EXCLUDE on intervention (service engagement/helpseeking behaviour)	
####	Jones (2021) (ID:87851477)	Patterns and Predictors of Reincarceration among Prisoners with Serious Mental Illness: A Cohort Study: Modèles et prédicteurs de réincarcération chez les prisonniers souffrant de maladie mentale grave : Une étude de cohorte.	-EXCLUDE on intervention (service engagement/helpseeking behaviour)	
####	Jones (2021) (ID:87852757)	Perceptions of Care Coordination Among Homeless Veterans Receiving Medical Care in the Veterans Health Administration and Community Care Settings Results From a National Survey	-EXCLUDE on intervention (service engagement/helpseeking behaviour)	
####	Jones (2023) (ID:87857938)	Black Girl Black Girl Holla and Let Em' Hear You: An Autoethnographic Exploration of My Experiences From Black Girlhood to Black Womanhood	-EXCLUDE on population (no housing precarity)	
####	Jonker (2015) (ID:87848886)	The effectiveness of interventions during and after residence in women's shelters: a meta-analysis.	-EXCLUDE on intervention (service engagement/helpseeking behaviour)	
####	Jonker (2019) (ID:87848474)	Factors Related To Depression and Post-Traumatic Stress Disorder in Shelter-Based Abused Women.	-EXCLUDE on evidence and form (evidence not in written form or presented as research output)	
####	Joomun (2019) (ID:87850529)	Women with dependent children who are homeless and living in temporary accommodation : an interpretative phenomenological analysis of their experiences of loss and the barriers to accessing health services	-INCLUDE on title & abstract	-EXCLUDE on intervention (service engagement/helpseeking behaviour)
####	Jordan (2012) (ID:87851216)	Addressing the needs of LGBT youth and their families: A public health perspective.	-EXCLUDE on population (no housing precarity)	
####	Jordan (2020) (ID:87857476)	Mandating Inclusion: Critical Trans Perspectives on Domestic and Sexual Violence Advocacy	-EXCLUDE on intervention (service engagement/helpseeking behaviour)	

####	Josenhans (2020) (ID:87963143)	Gender, rights and responsibilities: The need for a global analysis of the sexual exploitation of boys	-EXCLUDE on population (women)	
####	JOSEPH (2005) (ID:87857171)	Monitoring poverty and social exclusion in Wales 2005	-EXCLUDE on date (2010)	
####	Joseph (2007) (ID:87853697)	The relevance of involvement in micro-credit self-help groups and empowerment: Findings from a survey of rural women in Tamilnadu (India)	-EXCLUDE on country (High-Income)	
####	Joshi (2012) (ID:87849093)	"I didn't know I could turn colors": Health problems and health care experiences of women strangled by an intimate partner.	-INCLUDE on title & abstract	-EXCLUDE on intervention (service engagement/helpseeking behaviour)
####	Joshi (2019) (ID:87848427)	Burden of household food insecurity in urban slum settings.	-EXCLUDE on country (High-Income)	
####	Joy (2023) (ID:87858022)	The Prognoses of Frontline Workers and Increasing Risk Factors of the Social Work Profession	-EXCLUDE on intervention (service engagement/helpseeking behaviour)	
####	JOYCE (2011) (ID:87857317)	Promoting help-seeking among adolescents and young adults through consideration of the adaptive functions of low mood: a pilot study	-EXCLUDE on population (no housing precarity)	
####	JUDGE (2005) (ID:87857181)	Pathways to care in first episode psychosis: a pilot study on help-seeking precipitants and barriers to care	-EXCLUDE on date (2010)	
####	Judge (2018) (ID:87848506)	Uncharted Waters: Developing Mental Health Services for Survivors of Domestic Human Sex Trafficking.	-INCLUDE on title & abstract	-EXCLUDE (IPV/DVA but little discussion on housing)
####	JUHILA (2008) (ID:87856951)	Forms of advocacy in social welfare work with homeless women	-EXCLUDE on date (2010)	
####	JUHILA (2009) (ID:87856977)	From care to fellowship and back: interpretative repertoires used by social welfare workers when describing their relationship with homeless women	-EXCLUDE on date (2010)	
####	Julia (2021) (ID:87851499)	Creativity, Resilience and Resistance: Black Birthworkers' Responses to the COVID-19 Pandemic	-EXCLUDE on intervention (service engagement/helpseeking behaviour)	
####	Jung (2023) (ID:87857716)	Breaking the Binary: How Clinicians Can Ensure Everyone Receives High Quality Reproductive Health Services	-EXCLUDE on population (no housing precarity)	
####	Jupp (2017) (ID:87857688)	Home space, gender and activism: The visible and the invisible in austere times	-EXCLUDE on intervention (service engagement/helpseeking behaviour)	
####	Justice (2015) (ID:87858071)	Justice	-EXCLUDE on evidence and form (evidence not in written form or presented as research output)	
####	Jutkowitz (2019) (ID:87848407)	Homeless Veterans in Nursing Homes: Care for Complex Medical, Substance Use, and Social Needs.	-EXCLUDE on intervention (service engagement/helpseeking behaviour)	
####	K (2023) (ID:87857842)	Feminist Therapy: Supervision as a Pathway Toward Equitable, Affirming Care for Nonbinary Clients	-EXCLUDE on population (no housing precarity)	
####	Kaberuka (2017) (ID:87853056)	Factors determining child mortality in Uganda	-EXCLUDE on country (High-Income)	
####	Kabir (2016) (ID:87848756)	Climate change and health in Bangladesh: a baseline cross-sectional survey.	-EXCLUDE on country (High-Income)	
####	Kachingwe (2023) (ID:87850574)	'I told him I'm gonna get it': Intimate partner birth control communication among homeless young women.	-EXCLUDE on population (people aged under 18 years)	
####	Kaestle (2012) (ID:87851268)	Selling and buying sex: A longitudinal study of risk and protective factors in adolescence.	-EXCLUDE on population (people aged under 18 years)	
####	Kagal (2020) (ID:87857521)	Towards an intersectional praxis in international development: what can the sector learn from Black feminists located in the global North?	-EXCLUDE on population (no housing precarity)	
####	KAGAN (2016) (ID:87857119)	Factors predicting the likelihood of seeking help from social workers in Israel	-EXCLUDE on population (no housing precarity)	
####	Kahan (2020) (ID:87849361)	Implementing a trauma-informed intervention for homeless female survivors of gender-based violence: Lessons learned in a large Canadian urban centre	-INCLUDE on title & abstract	-EXCLUDE (IPV/DVA but little discussion on housing)
####	Kaine (2017) (ID:87857478)	Women, work and industrial relations in Australia in 2016	-EXCLUDE on population (no housing precarity)	
####	Kainz (2019) (ID:87852984)	Sex-specific analysis of haemodialysis prevalence, practices and mortality over time: the Austrian Dialysis Registry from 1965 to 2014	-EXCLUDE on population (no housing precarity)	
####	Kaiser (2014) (ID:87857501)	I Couldn't Save Myself: Lived Experiences of Female Street Children in Bangladesh	-EXCLUDE on country (High-Income)	
####	Kaiser (2019) (ID:87848442)	Predictors of Diet-Related Health Outcomes in Food-Secure and Food-Insecure Communities.	-EXCLUDE on intervention (service engagement/helpseeking behaviour)	

####	Kaiser (2020) (ID:87850711)	Mental health and psychosocial support needs among people displaced by Boko Haram in Nigeria.	-EXCLUDE on country (High-Income)	
####	Kalichman (2010) (ID:87853398)	Health and Treatment Implications of Food Insufficiency among People Living with HIV/AIDS, Atlanta, Georgia	-EXCLUDE on population (no housing precarity)	
####	Kalliokoski (2013) (ID:87853155)	Physical performance and 25-hydroxyvitamin D: a cross-sectional study of pregnant Swedish and Somali immigrant women and new mothers	-EXCLUDE on population (no housing precarity)	
####	Kaltsidis (2021) (ID:87848199)	Typology of changes in quality of life over 12 months among currently or formerly homeless individuals using different housing services in Quebec, Canada.	-EXCLUDE on intervention (service engagement/helpseeking behaviour)	
####	Kamimura (2013) (ID:87849028)	Health indicators, social support, and intimate partner violence among women utilizing services at a community organization.	-INCLUDE on title & abstract	-EXCLUDE on intervention (service engagement/helpseeking behaviour)
####	Kamimura (2015) (ID:87850982)	Women in a shelter: Victims of intimate partner violence-Their health and safety needs.	-EXCLUDE on intervention (service engagement/helpseeking behaviour)	
####	Kamke (2020) (ID:87850689)	Safer sex communication and sexual health behaviors among a representative statewide sample of homeless adolescents.	-EXCLUDE on population (people aged under 18 years)	
####	Kanai (2020) (ID:87857448)	Between the perfect and the problematic: everyday femininities, popular feminism, and the negotiation of intersectionality	-EXCLUDE on population (no housing precarity)	
####	Kandasamy (2014) (ID:87848934)	Obstetric risks and outcomes of refugee women at a single centre in Toronto.	-EXCLUDE on intervention (service engagement/helpseeking behaviour)	
####	KANE (2010) (ID:87856909)	Perceptions of students about younger and older men and women who may be homeless	-EXCLUDE on intervention (service engagement/helpseeking behaviour)	
####	Kanter (2021) (ID:87848185)	Willingness to use a wearable device capable of detecting and reversing overdose among people who use opioids in Philadelphia.	-EXCLUDE on intervention (service engagement/helpseeking behaviour)	
####	KANUKOLLUA (2011) (ID:87857379)	The idealized cultural identities model on help-seeking and child sexual abuse: a conceptual model for contextualizing perceptions and experiences of South Asian Americans	-EXCLUDE on population (people aged under 18 years)	
####	Kanyeredzi (2014) (ID:87963183)	Knowing what I know now: black women talk about violence inside and outside the home	-EXCLUDE on intervention (service engagement/helpseeking behaviour)<br>-EXCLUDE - but review for literature	
####	Kanyeredzi (2016) (ID:87963374)	Finding a Voice: African and Caribbean Heritage Women Help Seeking 1	-EXCLUDE on population (no housing precarity)<br>-EXCLUDE - but review for literature	
####	Kanzaria (2019) (ID:87848378)	Frequent Emergency Department Users: Focusing Solely On Medical Utilization Misses The Whole Person.	-EXCLUDE on population (no gender focus; women population <50)	
####	Kapilashrami (2018) (ID:87963239)	Examining intersectional inequalities in access to health (enabling) resources in disadvantaged communities in Scotland: advancing the participatory paradigm	-EXCLUDE on population (no housing precarity)	
####	Kar (2018) (ID:87848493)	Prevalence and pattern of mental illnesses in Uttar Pradesh, India: Findings from the National Mental Health Survey 2015-16.	-EXCLUDE on country (High-Income)	
####	Karadzhov (2020) (ID:87853433)	Coping amidst an assemblage of disadvantage: A qualitative metasynthesis of first-person accounts of managing severe mental illness while homeless	-EXCLUDE on intervention (service engagement/helpseeking behaviour)	
####	Karin (2019) (ID:87851911)	Nothing about us, without us: A case study of a consumer-run organization by and for people on the autism spectrum in the Netherlands	-EXCLUDE on population (no housing precarity)	
####	Karlsson (2021) (ID:87850633)	A prospective cohort study of risk behaviours, retention and loss to follow-up over 5 years among women and men in a needle exchange program in Stockholm, Sweden.	-EXCLUDE on intervention (service engagement/helpseeking behaviour)	
####	KARRAS (2021) (ID:87857106)	Veteran perspectives of barriers and facilitators to campaigns promoting help seeking during crisis	-EXCLUDE on population (no housing precarity)	
####	Karunaratne (2023) (ID:87963079)	The influence of family and culture on South Asian student dating violence survivors' college experiences	-EXCLUDE on population (no housing precarity)	
####	Kattari (2017) (ID:87850855)	Social networks and sexual risk factor differences between cisgender heterosexual and cisgender LGBQ homeless youths.	-EXCLUDE on intervention (service engagement/helpseeking behaviour)	

####	Kattari (2017) (ID:87852788)	Racial and Ethnic Differences in Experiences of Discrimination in Accessing Social Services Among Transgender/Gender-Nonconforming People	-EXCLUDE on population (no housing precarity)	
####	Kattari (2017) (ID:87852908)	On the Margins of Marginalized: Transgender Homelessness and Survival Sex	-EXCLUDE - but review for literature<br>-EXCLUDE on intervention (service engagement/helpseeking behaviour)	
####	Kattari (2020) (ID:87850679)	Providing appropriate health-care services to transgender and gender nonconforming survivors of IPV.	-EXCLUDE on intervention (service engagement/helpseeking behaviour)	
####	Katz (2016) (ID:87852714)	'I won't be able to go home being pregnant': sex work and pregnancy in Dhaka, Bangladesh	-EXCLUDE on country (High-Income)	
####	Kaufman (2015) (ID:87851576)	Lesbian, gay, bisexual, and transgender inmates	-INCLUDE on title & abstract	-EXCLUDE on target group (no housing precarity)
####	KAUKINEN (2002) (ID:87857199)	The help-seeking decisions of violent crime victims: an examination of the direct and conditional effects of gender and the victim-offender relationship	-EXCLUDE on date (2010)	
####	KAUKINEN (2004) (ID:87857108)	The help-seeking strategies of female violent-crime victims: the direct and conditional effects of race and the victim-offender relationship	-EXCLUDE on date (2010)	
####	KAUKINEN (2013) (ID:87857290)	Status compatibility and help-seeking behaviors among female intimate partner violence victims	-INCLUDE on title & abstract	-EXCLUDE (IPV/DVA but little discussion on housing)
####	Kawaii-Bogue (2017) (ID:87963322)	Mental health care access and treatment utilization in African American communities: An integrative care framework	-EXCLUDE on population (no housing precarity)	
####	Kawakami (2020) (ID:87848271)	Onset and remission of common mental disorders among adults living in temporary housing for three years after the triple disaster in Northeast Japan: comparisons with the general population.	-EXCLUDE on population (women)	
####	Kawatsu (2018) (ID:87848500)	The profile of prisoners with tuberculosis in Japan.	-EXCLUDE on intervention (service engagement/helpseeking behaviour)	
####	Ke (2010) (ID:87849245)	Social support and Quality of Life: a cross-sectional study on survivors eight months after the 2008 Wenchuan earthquake.	-EXCLUDE on country (High-Income)	
####	Keane (2018) (ID:87848519)	Trajectories of Psychological Distress in Australians Living in Urban Poverty: The Impact of Interpersonal Trauma.	-EXCLUDE on population (women)	
####	KEARNS (2019) (ID:87857295)	The impact of community-based mental health service provision on stigma and attitudes towards professional help-seeking	-EXCLUDE on population (no housing precarity)	
####	Keast (2016) (ID:87848760)	Evaluation of Abuse-Deterrent or Tamper-Resistant Opioid Formulations on Overall Health Care Expenditures in a State Medicaid Program.	-EXCLUDE on intervention (service engagement/helpseeking behaviour)	
####	Keefe (2017) (ID:87857625)	Aftercare Engagement: A Review of the Literature Through the Lens of Disparities	-EXCLUDE on population (no gender focus; women population <50)	
####	Keene (2013) (ID:87851154)	The meaning of homelessness to homeless women veterans.	-EXCLUDE on intervention (service engagement/helpseeking behaviour)<br>-EXCLUDE - but review for literature	
####	Keeshin (2011) (ID:87849185)	Screening homeless youth for histories of abuse: prevalence, enduring effects, and interest in treatment.	-EXCLUDE on population (women)	
####	Kehn (2013) (ID:87849015)	Identifying the health service needs of homeless adults with physical disabilities.	-EXCLUDE on population (no gender focus; women population <50)	
####	Keizur (2020) (ID:87848346)	Safety and Effectiveness of Same-Day Chlamydia trachomatis and Neisseria gonorrhoeae Screening and Treatment Among Gay, Bisexual, Transgender, and Homeless Youth in Los Angeles, California, and New Orleans, Louisiana.	-EXCLUDE on intervention (service engagement/helpseeking behaviour)	
####	KELLER (1997) (ID:87857256)	Cultural diversity and help-seeking behavior: sources of help and obstacles to support for parents	-EXCLUDE on date (2010)	
####	KELLER (2000) (ID:87857253)	Attitudes of low-income parents toward seeking help with parenting: implications for practice	-EXCLUDE on date (2010)	
####	Keller (2019) (ID:87851912)	Urban Partnerships to Address Health Literacy in High Need Populations	-EXCLUDE on intervention (service engagement/helpseeking behaviour)	
####	Keller (2021) (ID:87857691)	Successful Leadership Characteristics in Non-Governmental Organizations	-EXCLUDE on intervention (service engagement/helpseeking behaviour)	
####	Kelly (2017) (ID:87850899)	Diversity in couple and family therapy: Ethnicities, sexualities, and socioeconomics.	-EXCLUDE on population (no housing precarity)	

####	Kelly (2017) (ID:87851593)	2358	-EXCLUDE on intervention (service engagement/helpseeking behaviour)	
####	Kelly (2018) (ID:87848533)	Using Electronic Health Records to Enhance a Peer Health Navigator Intervention: A Randomized Pilot Test for Individuals with Serious Mental Illness and Housing Instability.	-EXCLUDE on population (women)	
####	Kelly (2018) (ID:87850836)	Long-acting intramuscular naltrexone for opioid use disorder: Utilization and association with multi-morbidity nationally in the Veterans Health Administration.	-EXCLUDE on intervention (service engagement/helpseeking behaviour)	
####	Kelly (2021) (ID:87848190)	Association of Social and Behavioral Risk Factors With Mortality Among US Veterans With COVID-19.	-EXCLUDE on intervention (service engagement/helpseeking behaviour)	
####	Kelton (2022) (ID:87851555)	Complementary/integrative healthcare utilization in US Gulf-War era veterans: Descriptive analyses based on deployment history, combat exposure, and Gulf War Illness	-EXCLUDE on intervention (service engagement/helpseeking behaviour)	
####	Kemei (2023) (ID:87857515)	The forms and adverse effects of insecurities among internally displaced children in Ethiopia	-EXCLUDE on country (High-Income)	
####	Kendall (2019) (ID:87857648)	Holistic Conceptualizations of Health by Incarcerated Aboriginal Women in New South Wales, Australia	-INCLUDE on title & abstract	-EXCLUDE on target group (no housing precarity)
####	Kennedy (2010) (ID:87849284)	Unprotected sex of homeless women living in Los Angeles county: an investigation of the multiple levels of risk.	-INCLUDE on title & abstract	-EXCLUDE on intervention (intersectionality)
####	KENNEDY (2010) (ID:87856917)	Risk chains over the life course among homeless urban adolescent mothers: altering their trajectories through formal support	-INCLUDE on title & abstract	-EXCLUDE on intervention (service engagement/helpseeking behaviour)
####	Kennedy (2013) (ID:87849065)	Unprotected sex among heterosexually active homeless men: results from a multi-level dyadic analysis.	-EXCLUDE on population (women)	
####	Kennedy (2018) (ID:87848559)	Social and structural factors associated with greater time with a plasma HIV-1 RNA viral load above log10(1500) copies/ml among illicit drug users.	-EXCLUDE on intervention (service engagement/helpseeking behaviour)	
####	Kennedy (2018) (ID:87848560)	A pilot test of a motivational interviewing social network intervention to reduce substance use among housing first residents.	-EXCLUDE on population (women)	
####	Kennedy (2019) (ID:87848396)	Supervised Injection Facility Utilization Patterns: A Prospective Cohort Study in Vancouver, Canada.	-EXCLUDE on population (women)	
####	Kennedy (2021) (ID:87857627)	Improving Engagement with African American Women Identified with Depression through Cultural Awareness	-EXCLUDE on population (no housing precarity)	
####	Kennedy (2022) (ID:87848168)	Restructuring personal networks with a Motivational Interviewing social network intervention to assist the transition out of homelessness: A randomized control pilot study.	-EXCLUDE on population (women)	
####	Kennedy (2023) (ID:87851552)	Can This Provider Be Trusted? A Review of the Role of Trustworthiness in the Provision of Community-Based Services for Intimate Partner Violence Survivors.	-INCLUDE on title & abstract	-EXCLUDE (IPV/DVA but little discussion on housing)<br>-EXCLUDE (systematic review)* only use with other code
####	Kennelly (2020) (ID:87857518)	Urban masculinity, contested spaces, and classed subcultures: young homeless men navigating downtown Ottawa, Canada	-EXCLUDE on population (women)	
####	Kenney (2019) (ID:87858058)	Solidarity, Not Charity: Mutual Aid in Natural Disaster Relief	-EXCLUDE on intervention (service engagement/helpseeking behaviour)	
####	Kenney (2021) (ID:87848244)	Examining Overdose and Homelessness as Predictors of Willingness to Use Supervised Injection Facilities by Services Provided Among Persons Who Inject Drugs.	-EXCLUDE on intervention (service engagement/helpseeking behaviour)	
####	Kenny (2019) (ID:87852712)	A picture of the older homeless female veteran: A qualitative, case study analysis	-INCLUDE on title & abstract	-EXCLUDE on intervention (service engagement/helpseeking behaviour)
####	Keogh (2015) (ID:87848847)	Health and use of health services of people who are homeless and at risk of homelessness who receive free primary health care in Dublin.	-EXCLUDE on population (women)	
####	Kerman (2019) (ID:87848439)	Perceptions of Service Use Among Currently and Formerly Homeless Adults with Mental Health Problems.	-EXCLUDE on population (women)	
####	Kerman (2019) (ID:87848464)	Predictors of Mental Health Recovery in Homeless Adults with Mental Illness.	-EXCLUDE on population (women)	

####	Kerman (2020) (ID:87848313)	Service use and recovery among currently and formerly homeless adults with mental illness.	-EXCLUDE on population (women)	
####	Kerman (2020) (ID:87848332)	Effectiveness of Housing First for Homeless Adults with Mental Illness Who Frequently Use Emergency Departments in a Multisite Randomized Controlled Trial.	-EXCLUDE on intervention (service engagement/helpseeking behaviour)	
####	Kerman (2020) (ID:87848350)	Surviving versus living life: Capabilities and service use among adults with mental health problems and histories of homelessness.	-EXCLUDE on population (no gender focus; women population <50)<br>-EXCLUDE - but review for literature	
####	Kerouedan (2019) (ID:87852874)	About migrants' health status and migrant's access to health care in France	-EXCLUDE on population (no gender focus; women population <50)	
####	KERSLAKE (2021) (ID:87856943)	When we work together: learning the lessons - interim report	-EXCLUDE on intervention (service engagement/helpseeking behaviour)	
####	KERSLAKE (2021) (ID:87857033)	A new way of working: ending rough sleeping together: final report	-EXCLUDE on intervention (service engagement/helpseeking behaviour)	
####	Kertesz (2013) (ID:87849001)	Comparing homeless persons' care experiences in tailored versus nontailored primary care programs.	-EXCLUDE on intervention (service engagement/helpseeking behaviour)	
####	Kesten (2012) (ID:87849142)	Recidivism rates among mentally ill inmates: impact of the Connecticut Offender Reentry Program.	-EXCLUDE on intervention (service engagement/helpseeking behaviour)	
####	Kestler (2019) (ID:87848477)	Yes, not now, or never: an analysis of reasons for refusing or accepting emergency department-based take-home naloxone.	-EXCLUDE on population (women)	
####	Ketteringham (2022) (ID:87850555)	Architectures of identity : English modernism, domesticity, and imperial decline	-EXCLUDE on intervention (service engagement/helpseeking behaviour)	
####	Keuroghlian (2014) (ID:87851106)	Out on the street: A public health and policy agenda for lesbian, gay, bisexual, and transgender youth who are homeless.	-EXCLUDE on intervention (service engagement/helpseeking behaviour)	
####	Keynejad (2021) (ID:87851889)	Identifying and responding to domestic violence and abuse in healthcare settings	-INCLUDE on title & abstract	-EXCLUDE (IPV/DVA but little discussion on housing)
####	Khademi (2021) (ID:87853453)	Quality of life of HIV-infected individuals: insights from a study of patients in Kermanshah, Iran	-EXCLUDE on country (High-Income)	
####	Khademvatan (2014) (ID:87851860)	Survey of the relationship between metabolic syndrome and myocardial infarction in hospitals of Urmia University of medical sciences.	-EXCLUDE on population (no housing precarity)	
####	Khadra (2022) (ID:87851586)	Risk factors for miscarriage in Syrian refugee women living in non-camp settings in Jordan: results from the Women ASPIRE cross-sectional study	-EXCLUDE on country (High-Income)	
####	Khaja (2021) (ID:87851973)	What It’s Like Working At A Women’s Shelter During The Pandemic	-EXCLUDE on intervention (service engagement/helpseeking behaviour)	
####	Khalil (2019) (ID:87852950)	Does Socioeconomic Status Affect Stage at Presentation for Larynx Cancer in Canada's Universal Health Care System?	-EXCLUDE on population (no housing precarity)	
####	Khalili (2022) (ID:87848171)	Shelter-Based Integrated Model Is Effective in Scaling Up Hepatitis C Testing and Treatment in Persons Experiencing Homelessness.	-EXCLUDE on intervention (service engagement/helpseeking behaviour)	
####	Khan (2021) (ID:87848189)	Does social support moderate between depression and suicidal ideation in low-income African Americans?.	-EXCLUDE on population (no gender focus; women population <50)	
####	Khan (2022) (ID:87963230)	Barriers to and facilitators of accessing HIV services for street-involved youth in Canada and Kenya	-EXCLUDE on population (no housing precarity)	
####	Khandor (2011) (ID:87849171)	Access to primary health care among homeless adults in Toronto, Canada: results from the Street Health survey.	-EXCLUDE on intervention (service engagement/helpseeking behaviour)	
####	Khanlou (2015) (ID:87850967)	Women's mental health: Resistance and resilience in community and society.	-EXCLUDE on population (no housing precarity)	
####	Khattak (2017) (ID:87853223)	The Role of Parents' Literacy in Malnutrition of Children Under the Age of Five Years in a Semi-Urban Community of Pakistan. A Case-Control Study	-EXCLUDE on country (High-Income)	
####	Kia (2020) (ID:87963059)	Poverty in lesbian, gay, bisexual, transgender, queer, and two-spirit (LGBTQ2S+) populations in Canada: An intersectional review of the literature	-EXCLUDE on intervention (service engagement/helpseeking behaviour)	
####	**Kiamanesh (2019) (ID:87851642)**	**"We are not weak, we just experience domestic violence"—Immigrant women's experiences of encounters with service providers as a result of domestic violence.**	**-INCLUDE on title & abstract**	**-INCLUDE on full study**

####	Kibler (2019) (ID:87858003)	Teachers' Experiences in Serving Late-Entering Central American Refugees with Limited or Interrupted Formal Education	-EXCLUDE on intervention (service engagement/helpseeking behaviour)	
####	KIDANE (2004) (ID:87857351)	Fostering unaccompanied asylum seeking and refugee children: a training course for foster carers	-EXCLUDE on date (2010)	
####	KIDANE (2005) (ID:87857130)	Looking after unaccompanied asylum seeking and refugee children: a training course for social care professionals	-EXCLUDE on date (2010)	
####	KIDD (2004) (ID:87856989)	Evaluating child and youth homelessness	-EXCLUDE on date (2010)	
####	Kidd (2021) (ID:87852997)	The Second National Canadian Homeless Youth Survey: Mental Health and Addiction Findings: La Deuxieme Enquete Nationale Aupres des Jeunes Sans Abri : Resultats en Matiere De Sante Mentale et de Toxicomanie	-EXCLUDE on intervention (service engagement/helpseeking behaviour)	
####	KIERNAN (2018) (ID:87857310)	Are veterans different? Understanding veterans' help-seeking behaviour for alcohol problems	-EXCLUDE on population (women)	
####	Kilbourne (2010) (ID:87849239)	Implementing composite quality metrics for bipolar disorder: towards a more comprehensive approach to quality measurement.	-EXCLUDE on intervention (service engagement/helpseeking behaviour)	
####	Killaspy (2016) (ID:87848713)	Quality of life, autonomy, satisfaction, and costs associated with mental health supported accommodation services in England: a national survey.	-EXCLUDE on intervention (service engagement/helpseeking behaviour)	
####	Killaspy (2016) (ID:87848754)	Adaptation of the Quality Indicator for Rehabilitative Care (QuIRC) for use in mental health supported accommodation services (QuIRC-SA).	-EXCLUDE on intervention (service engagement/helpseeking behaviour)	
####	Kim (2011) (ID:87849223)	Factors influencing help-seeking behavior among battered Korean women in intimate relationships.	-INCLUDE on title & abstract	-EXCLUDE (IPV/DVA but little discussion on housing)
####	Kim (2017) (ID:87857437)	The Biopolitical Effect of Cold War Containment in a Coming-of-Age Narrative: On Postcolonial Subjectivity in Hagedorn's Dogeaters 1	-EXCLUDE on evidence and form (evidence not in written form or presented as research output)	
####	Kim (2023) (ID:87851769)	Investigation of the Relationship Between Psychiatry Visit and Suicide After Deliberate Self-harm: Longitudinal National Cohort Study.	-EXCLUDE on population (no housing precarity)	
####	Kimbler (2017) (ID:87848705)	Characteristics of the old and homeless: identifying distinct service needs.	-EXCLUDE on population (no gender focus; women population <50)<br>-EXCLUDE - but review for literature	
####	Kimerling (2016) (ID:87852986)	Prevalence of Intimate Partner Violence among Women Veterans who Utilize Veterans Health Administration Primary Care	-EXCLUDE on population (no housing precarity)	
####	Kimmich (2010) (ID:87963290)	From classroom to community in women's studies	-EXCLUDE on population (no housing precarity)	
####	Kincaid (2019) (ID:87851993)	Methodological Considerations for Researching Hidden-Populations with an Emphasis on Homeless Research Sampling Methods	-EXCLUDE on intervention (service engagement/helpseeking behaviour)	
####	Kindermann (2017) (ID:87848623)	Prevalence of and Risk Factors for Secondary Traumatization in Interpreters for Refugees: A Cross-Sectional Study.	-EXCLUDE on intervention (service engagement/helpseeking behaviour)	
####	King (2020) (ID:87848266)	Community-derived recommendations for healthcare systems and medical students to support people who are houseless in Portland, Oregon: a mixed-methods study.	-EXCLUDE on intervention (service engagement/helpseeking behaviour)	
####	King (2020) (ID:87857564)	Anthropology and #MeToo: Reimagining fieldwork	-EXCLUDE on evidence and form (evidence not in written form or presented as research output)	
####	King (2022) (ID:87858004)	Tobacco-related health inequalities among Black Americans: A narrative review of structural and historical influences	-EXCLUDE on population (women)	
####	King (2023) (ID:87963235)	Structural vulnerability as a conceptual framework for transgender health research: Findings from a community needs assessment of transgender women of colour in Detroit	-EXCLUDE on population (no housing precarity)	
####	KING-OWEN (2020) (ID:87851413)	Service user involvement in social work education: a case study. A discussion paper from the Centre for Welfare Reform	-EXCLUDE on intervention (service engagement/helpseeking behaviour)	
####	Kirkland (2022) (ID:87852951)	Perceptions of Resettled Refugee Congolese Women: Maintaining Cultural Traditions during Resettlement	-INCLUDE on title & abstract	-EXCLUDE on intervention (service engagement/helpseeking behaviour)
####	Kirkpatrick (2010) (ID:87849272)	Assessing the relevance of neighbourhood characteristics to the household food security of low-income Toronto families.	-EXCLUDE on population (no housing precarity)	

####	Kiross (2021) (ID:87853243)	Individual-, household- and community-level determinants of infant mortality in Ethiopia	-EXCLUDE on country (High-Income)	
####	Kirst (2014) (ID:87848955)	The promise of recovery: narratives of hope among homeless individuals with mental illness participating in a Housing First randomised controlled trial in Toronto, Canada.	-EXCLUDE on population (women)	
####	Kirst (2015) (ID:87848878)	The impact of a Housing First randomized controlled trial on substance use problems among homeless individuals with mental illness.	-EXCLUDE on intervention (service engagement/helpseeking behaviour)	
####	Kirton (2018) (ID:87857970)	Neoliberalism, 'race' and child welfare	-EXCLUDE on population (people aged under 18 years)	
####	KISOR (2002) (ID:87856950)	Older homeless women: reframing the stereotype of the bag lady	-EXCLUDE on date (2010)	
####	KISSMAN (1999) (ID:87857013)	Respite from stress and other service needs of homeless families	-EXCLUDE on date (2010)	
####	Kitsantas (2023) (ID:87848133)	Racial/ethnic differences in medication for addiction treatment for opioid use disorders among pregnant women in treatment facilities supported by state funds.	-EXCLUDE on intervention (service engagement/helpseeking behaviour)	
####	Klann (2020) (ID:87857531)	A Pregnancy Decision-Making Model: Psychological, Relational, and Cultural Factors Affecting Unintended Pregnancy	-EXCLUDE on population (no housing precarity)	
####	Klatt (2014) (ID:87848979)	Rationalising predictors of child sexual exploitation and sex-trading.	-EXCLUDE on intervention (service engagement/helpseeking behaviour)	
####	Klein (2010) (ID:87853449)	Self-Esteem and HIV Risk Practices Among Young Adult Ecstasy Users	-EXCLUDE on population (no housing precarity)	
####	Klein (2018) (ID:87850814)	Increasing access to pre-exposure prophylaxis (PrEP) among transgender women and trans feminine non-binary individuals in New York City.	-EXCLUDE on intervention (service engagement/helpseeking behaviour)	
####	Klein (2021) (ID:87963231)	Housing interventions for intimate partner violence survivors: A systematic review	-INCLUDE on title & abstract	-EXCLUDE on intervention (intersectionality)<br>-EXCLUDE (systematic review)* only use with other code
####	Klineberg (2017) (ID:87848632)	Health status of marginalised young people in unstable accommodation.	-EXCLUDE on intervention (service engagement/helpseeking behaviour)	
####	Klooster (2023) (ID:87857872)	Affirmative Sexual Health Care for Transgender & Nonbinary Youth: Toward a Sex-Positive Approach	-EXCLUDE on population (no housing precarity)	
####	Klop (2018) (ID:87848524)	The Views of Homeless People and Health Care Professionals on Palliative Care and the Desirability of Setting Up a Consultation Service: A Focus Group Study.	-EXCLUDE on intervention (service engagement/helpseeking behaviour)	
####	Knight (2014) (ID:87852885)	Single room occupancy (SRO) hotels as mental health risk environments among impoverished women: The intersection of policy, drug use, trauma, and urban space	-EXCLUDE on intervention (service engagement/helpseeking behaviour)	
####	Knowles (2016) (ID:87850961)	"Do you wanna breathe or eat?": Parent perspectives on child health consequences of food insecurity, trade-offs, and toxic stress.	-EXCLUDE on intervention (service engagement/helpseeking behaviour)	
####	Knox (2019) (ID:87851792)	Urban partnerships to address health literacy in low-income and homeless adults...American Academy of Physician Assistants (AAPA) Conference, May 18-20, 2019, Denver, Colorado.	-EXCLUDE on intervention (service engagement/helpseeking behaviour)	
####	Koch (2015) (ID:87848833)	Proxy and self-reported Quality of Life in adults with intellectual disabilities: Impact of psychiatric symptoms, problem behaviour, psychotropic medication and unmet needs.	-EXCLUDE on population (no housing precarity)	
####	Koch (2015) (ID:87857597)	Urban Outcasts, Disposable Bodies, and Embodied Research in a Western Canadian “Arriviste” City	-EXCLUDE on population (women)	
####	Koch (2020) (ID:87858077)	The cost of being transgender: where socio-economic status, global health care systems, and gender identity intersect	-EXCLUDE on population (no housing precarity)	
####	Koci (2012) (ID:87849090)	Informing practice regarding marginalization: the application of the Koci Marginality Index.	-EXCLUDE on intervention (service engagement/helpseeking behaviour)	
####	Koci (2014) (ID:87851066)	Women's functioning following an intervention for partner violence: New knowledge for clinical practice from a 7-year study.	-EXCLUDE on intervention (service engagement/helpseeking behaviour)	

####	KOEHN (2013) (ID:87857467)	Revealing the shape of knowledge using an intersectionality lens: results of a scoping review on the health and health care of ethnocultural minority older adults	-EXCLUDE on intervention (service engagement/helpseeking behaviour)	
####	Koffarnus (2011) (ID:87849189)	A randomized clinical trial of a Therapeutic Workplace for chronically unemployed, homeless, alcohol-dependent adults.	-EXCLUDE on intervention (service engagement/helpseeking behaviour)	
####	Koffarnus (2013) (ID:87849006)	Monetary incentives to reinforce engagement and achievement in a job-skills training program for homeless, unemployed adults.	-EXCLUDE on intervention (service engagement/helpseeking behaviour)	
####	Koh (2012) (ID:87853134)	The Hunger-Obesity Paradox: Obesity in the Homeless	-EXCLUDE on intervention (service engagement/helpseeking behaviour)	
####	KOHLI (2006) (ID:87857127)	The sound of silence: listening to what unaccompanied asylum-seeking children say and do not say	-EXCLUDE on date (2010)	
####	KOHLI (2006) (ID:87857129)	The comfort of strangers: social work practice with unaccompanied asylum-seeking children and young people in the UK	-EXCLUDE on date (2010)	
####	Komaromy (2021) (ID:87848200)	Adaptation of a System of Treatment for Substance Use Disorders During the COVID-19 Pandemic.	-EXCLUDE on intervention (service engagement/helpseeking behaviour)	
####	Kondo (2017) (ID:87853120)	Health Disparities in Veterans A Map of the Evidence	-EXCLUDE on intervention (service engagement/helpseeking behaviour)	
####	Kone (2014) (ID:87857776)	Sex Worker Political Development in Costa Rica: from Informal Solidarities to Formal Organizing	-EXCLUDE on country (High-Income)	
####	Kong (2020) (ID:87852748)	Factors influencing the uptake of influenza vaccine vary among different groups in the hard-to-reach population	-EXCLUDE on intervention (service engagement/helpseeking behaviour)	
####	Kongats (2023) (ID:87848130)	Housing for vulnerable populations-identifying system cost implications: an analytic literature review.	-EXCLUDE on intervention (service engagement/helpseeking behaviour)	
####	Konnoth (2020) (ID:87852846)	Medicalization and the New Civil Rights	-EXCLUDE on intervention (service engagement/helpseeking behaviour)	
####	KOOPMAN (1997) (ID:87857403)	Acute stress disorder symptoms among female sexual abuse survivors seeking treatment	-EXCLUDE on date (2010)	
####	KOOPMANS (2007) (ID:87857377)	Gender and health care utilization: the role of mental distress and help-seeking propensity	-EXCLUDE on date (2010)	
####	Kopet (2013) (ID:87849368)	Experiencing service-learning in prison	-EXCLUDE on intervention (service engagement/helpseeking behaviour)	
####	Kornilova (2017) (ID:87848701)	Decline in HIV seroprevalence in street youth 2006-2012, St. Petersburg, Russia: moving toward an AIDS-free generation.	-EXCLUDE on country (High-Income)	
####	Koss (2017) (ID:87857511)	Victim Voice in Reenvisioning Responses to Sexual and Physical Violence Nationally and Internationally	-EXCLUDE on population (no housing precarity)	
####	KOSYLUK (2018) (ID:87857380)	Examining the impact of this is my brave on mental illness stigma and willingness to seek help: a pilot study	-EXCLUDE on population (no housing precarity)	
####	Kota (2023) (ID:87851534)	Stigma, Discrimination, and Mental Health Outcomes Among Transgender Women With Diagnosed HIV Infection in the United States, 2015-2018	-EXCLUDE on intervention (service engagement/helpseeking behaviour)	
####	Kotiso (2020) (ID:87848251)	Disparities in antenatal care service utilization among food secure and food insecure women in Gombora District, Hadiya zone, south Ethiopia.	-EXCLUDE on country (High-Income)	
####	Kotsatos (2022) (ID:87850602)	The relationship between prior maternal trauma, emotion regulation and maternal sensitivity and hostility among high-risk adolescent mothers.	-EXCLUDE on intervention (service engagement/helpseeking behaviour)	
####	Kotwal (2021) (ID:87848239)	Social Isolation and Loneliness Among San Francisco Bay Area Older Adults During the COVID-19 Shelter-in-Place Orders.	-EXCLUDE on intervention (service engagement/helpseeking behaviour)	
####	Kowalski (2010) (ID:87849280)	Associations between emotional exhaustion, social capital, workload, and latitude in decision-making among professionals working with people with disabilities.	-EXCLUDE on intervention (service engagement/helpseeking behaviour)	
####	Kozloff (2016) (ID:87848743)	The Unique Needs of Homeless Youths With Mental Illness: Baseline Findings From a Housing First Trial.	-EXCLUDE on intervention (service engagement/helpseeking behaviour)	
####	Krabbe (2021) (ID:87852987)	Exploring the operationalisation and implementation of outreach in community settings with hard-to-reach and hidden populations: protocol for a scoping review	-EXCLUDE on evidence and form (evidence not in written form or presented as research output)	

####	Krabbenborg (2013) (ID:87849039)	A strengths based method for homeless youth: effectiveness and fidelity of Houvast.	-EXCLUDE on population (women)	
####	Kraehe (2013) (ID:87857632)	Theoretical Considerations for Art Education Research with and about "Underserved Populations"	-EXCLUDE on population (no housing precarity)	
####	Krahn (2017) (ID:87858118)	Child Welfare Social Workers' Knowledge of Substance Abuse and Self-Efficacy in Working with Substance Abusing Clients	-EXCLUDE on intervention (service engagement/helpseeking behaviour)	
####	Kral (2010) (ID:87849268)	Acceptability of a safer injection facility among injection drug users in San Francisco.	-EXCLUDE on intervention (service engagement/helpseeking behaviour)	
####	KRANKE (2012) (ID:87857389)	What do African American youth with a mental illness think about help-seeking and psychiatric medication?: Origins of stigmatizing attitudes	-EXCLUDE on population (people aged under 18 years)	
####	Krause (2022) (ID:87848161)	Disruptions to School and Home Life Among High School Students During the COVID-19 Pandemic - Adolescent Behaviors and Experiences Survey, United States, January-June 2021.	-EXCLUDE on population (people aged under 18 years)	
####	Krausz (2013) (ID:87851180)	Mental disorder, service use, and barriers to care among 500 homeless people in 3 different urban settings.	-EXCLUDE on population (women)	
####	Kreuter (2021) (ID:87848240)	How Do Social Needs Cluster Among Low-Income Individuals?.	-EXCLUDE on intervention (service engagement/helpseeking behaviour)	
####	Krishna (2020) (ID:87851536)	The Burden of Deciding for Others	-EXCLUDE on evidence and form (evidence not in written form or presented as research output)	
####	Krishnadas (2021) (ID:87852647)	Factors associated with homelessness among women: a cross-sectional survey of outpatient mental health service users at The Banyan, India	-EXCLUDE on country (High-Income)	
####	Krishnamurti (2021) (ID:87848219)	Mobile Remote Monitoring of Intimate Partner Violence Among Pregnant Patients During the COVID-19 Shelter-In-Place Order: Quality Improvement Pilot Study.	-EXCLUDE on intervention (service engagement/helpseeking behaviour)	
####	Kritsotakis (2017) (ID:87848620)	Attitudes towards people with physical or intellectual disabilities among nursing, social work and medical students.	-EXCLUDE on intervention (service engagement/helpseeking behaviour)	
####	Krízová (2012) (ID:87852033)	VOLUNTEERING AND MUTUAL AID IN HEALTH AND SOCIAL CARE IN THE CZECH REPUBLIC AS AN EXAMPLE OF ACTIVE CITIZENSHIP	-EXCLUDE on population (no housing precarity)	
####	Kroll (2020) (ID:87850680)	Non-contact monitoring of agitation and use of a sheltering device in patients with dementia in emergency departments: A feasibility study.	-EXCLUDE on intervention (service engagement/helpseeking behaviour)	
####	Krueger (2022) (ID:87851786)	Successful substance use disorder recovery in transitional housing: Perspectives from African American women.	-INCLUDE on title & abstract	-EXCLUDE on population (housing precarity not discussed)
####	Krupski (2015) (ID:87848820)	Clinical Needs of Patients with Problem Drug Use.	-EXCLUDE on intervention (service engagement/helpseeking behaviour)	
####	Kube (2023) (ID:87963179)	Community-and data-driven homelessness prevention and service delivery: optimizing for equity	-EXCLUDE on intervention (service engagement/helpseeking behaviour)	
####	Kuchai (2022) (ID:87851490)	The role of computational tools in designing healthy housing for the displaced	-EXCLUDE on intervention (service engagement/helpseeking behaviour)	
####	Kucukboyaci (2018) (ID:87848570)	Cluster Analysis of Vulnerable Groups in Acute Traumatic Brain Injury Rehabilitation.	-EXCLUDE on intervention (service engagement/helpseeking behaviour)	
####	Kuffel (2022) (ID:87848156)	Prevalence of dementia and mild cognitive impairment before incarceration.	-EXCLUDE on intervention (service engagement/helpseeking behaviour)	
####	Kulkarni (2019) (ID:87963342)	Intersectional trauma-informed intimate partner violence (IPV) services: Narrowing the gap between IPV service delivery and survivor needs	-INCLUDE on title & abstract	-EXCLUDE on target group (not focused on women's behaviour/outcomes for women)
####	Kumar (2013) (ID:87853296)	Early Neonatal Mortality in India, 1990-2006	-EXCLUDE on country (High-Income)	
####	Kumar (2018) (ID:87848507)	"They don't like us....": Barriers to antiretroviral and opioid substitution therapy among homeless HIV positive people who inject drugs in Delhi: A mixed method study.	-EXCLUDE on country (High-Income)	
####	KUNG (2003) (ID:87857110)	Chinese Americans' help seeking for emotional distress	-EXCLUDE on date (2010)	
####	Kurbatova (2012) (ID:87849109)	Predictors of poor outcomes among patients treated for multidrug-resistant tuberculosis at DOTS-plus projects.	-EXCLUDE on intervention (service engagement/helpseeking behaviour)	
####	Kurdyla (2021) (ID:87848248)	Transgender Intimate Partner Violence and Help-Seeking Patterns.	-INCLUDE on title & abstract	-EXCLUDE (IPV/DVA but little discussion on housing)

####	Kwan (2023) (ID:87857554)	Toward an inclusive digital economy for all: Perspectives from an intersectional feminist social work lens	-EXCLUDE on population (no housing precarity)	
####	KWONG (2012) (ID:87857376)	Disability beliefs and help-seeking behavior of depressed Chinese-American patients in a primary care setting	-EXCLUDE on population (no housing precarity)	
####	Kyronlahti (2020) (ID:87851598)	Childhood cancer mortality and survival in immigrants: A population-based registry study in Finland	-EXCLUDE on population (people aged under 18 years)	
####	La Mar (2021) (ID:87850648)	Mental healthcare barriers and facilitators experienced by homeless women with serious mental illness.	-INCLUDE on title & abstract	-EXCLUDE on intervention (intersectionality)
####	La Motte-Kerr (2020) (ID:87848268)	Exploring the Association of Community Integration in Mental Health among Formerly Homeless Individuals Living in Permanent Supportive Housing.	-EXCLUDE on intervention (service engagement/helpseeking behaviour)	
####	Laban (2015) (ID:87857901)	Gendering surveillance studies: The empirical and normative promise of feminist methodology	-EXCLUDE on intervention (service engagement/helpseeking behaviour)	
####	Labby (2020) (ID:87848336)	Drivers of High-cost Medical Complexity in a Medicaid Population.	-EXCLUDE on intervention (service engagement/helpseeking behaviour)	
####	LaCalle (2013) (ID:87849048)	High-frequency users of emergency department care.	-EXCLUDE on population (no housing precarity)	
####	Lacey (2013) (ID:87963323)	From gender mainstreaming to intersectionality	-EXCLUDE on population (no housing precarity)<br>-EXCLUDE - but review for literature	
####	Lacombe-Duncan (2016) (ID:87857512)	An Intersectional Perspective on Access to HIV-Related Healthcare for Transgender Women	-EXCLUDE on population (no housing precarity)	
####	Lacombe-Duncan (2019) (ID:87848403)	Gender-affirming healthcare experiences and medical transition among transgender women living with HIV: a mixed-methods study.	-EXCLUDE on population (no housing precarity)	
####	Lacombe-Duncan (2022) (ID:87852970)	Context, Types, and Consequences of Violence Across the Life Course: A Qualitative Study of the Lived Experiences of Transgender Women Living With HIV	-EXCLUDE on intervention (service engagement/helpseeking behaviour)	
####	Lacombe-Duncan (2022) (ID:87857449)	"I do the she and her": A qualitative exploration of HIV care providers' considerations of trans women in gender-specific HIV care	-EXCLUDE on population (no housing precarity)	
####	Laconi (2016) (ID:87849294)	Problematic Internet use, time spent online and personality traits	-EXCLUDE on population (no housing precarity)	
####	LaFlamme (2021) (ID:87857665)	The Intersection of Community Engagement and Library Science	-EXCLUDE on population (no housing precarity)	
####	Lahr (2018) (ID:87857843)	Piercing the Cycle of Recidivism: A Self-Study to Inform Social Entrepreneurship Education as a Path to Successful Reentry for Previously Incarcerated African American Women	-EXCLUDE on population (no housing precarity)	
####	Lam (2016) (ID:87848745)	The Impact of Community Engagement on Health, Social, and Utilization Outcomes in Depressed, Impoverished Populations: Secondary Findings from a Randomized Trial.	-EXCLUDE on intervention (service engagement/helpseeking behaviour)	
####	Lamb (2012) (ID:87849156)	Access to mental health in primary care: a qualitative meta-synthesis of evidence from the experience of people from 'hard to reach' groups.	-EXCLUDE on intervention (service engagement/helpseeking behaviour)	
####	Lambdin (2018) (ID:87848513)	Accumulation of Jail Incarceration and Hardship, Health Status, and Unmet Health Care Need Among Women Who Use Drugs.	-EXCLUDE on intervention (service engagement/helpseeking behaviour)	
####	Lambert (2012) (ID:87850546)	Lingering 'on the borderland' : the meanings of home in Elizabeth Gaskell's fiction	-EXCLUDE on intervention (service engagement/helpseeking behaviour)	
####	Lang (2020) (ID:87851905)	Self-Care Management in Homeless Adults with Hypertension	-EXCLUDE on population (no gender focus; women population <50)	
####	Lang (2021) (ID:87850653)	Self-care management in homeless adults with hypertension.	-EXCLUDE on population (no gender focus; women population <50)	
####	**Langton (2020) (ID:87963022)**	**Improving family violence legal and support services for Aboriginal and Torres Strait Islander women**	**-INCLUDE on title & abstract**	**-INCLUDE on full study**
####	Lankenau (2012) (ID:87849114)	Patterns of prescription drug misuse among young injection drug users.	-EXCLUDE on intervention (service engagement/helpseeking behaviour)	
####	Lapiņa (2022) (ID:87857562)	'Diversity tourists'? Tracing whiteness through affective encounters with diversity in a gentrifying district in Copenhagen	-EXCLUDE on population (no housing precarity)	
####	LaPolla (2021) (ID:87858120)	First-generation College Students at a Women's College and Their Sense of Belonging	-EXCLUDE on population (no housing precarity)	

####	Larance (2018) (ID:87857602)	Strategically Stealthy: Women's Agency in Navigating Spousal Violence	-INCLUDE on title & abstract	-EXCLUDE (IPV/DVA but little discussion on housing)
####	Larkin (2018) (ID:87858098)	Shame & Depression in Black Males: Holding Space for Exploring Implications of and Healing from Structural Racism	-EXCLUDE on population (women)	
####	Larkins (2018) (ID:87857551)	Complicating communities: an intersectional approach to women's environmental justice narratives in the Rocky Mountain West	-EXCLUDE on intervention (service engagement/helpseeking behaviour)	
####	LaRosa (2022) (ID:87857979)	Understanding Unwanted Sexual Experiences of Transgender and Gender Diverse Individuals: An Interpretative Phenomenological Study	-EXCLUDE on population (no housing precarity)	
####	Larsen (2011) (ID:87849198)	In-patient treatment for dual diagnoses.	-EXCLUDE on population (no housing precarity)	
####	Latkin (2013) (ID:87849074)	Injection drug users' and their risk networks' experiences of and attitudes towards drug dealer violence in Baltimore, Maryland.	-EXCLUDE on intervention (service engagement/helpseeking behaviour)	
####	Latkin (2013) (ID:87849076)	The relationship between drug user stigma and depression among inner-city drug users in Baltimore, MD.	-EXCLUDE on intervention (service engagement/helpseeking behaviour)	
####	LATZER (2005) (ID:87857216)	Help-seeking characteristics of eating-disordered hotline callers: community based study	-EXCLUDE on date (2010)	
####	Laudet (2015) (ID:87848876)	Characteristics of students participating in collegiate recovery programs: a national survey.	-EXCLUDE on population (no housing precarity)	
####	Lauriks (2014) (ID:87848957)	The use of the Dutch Self-Sufficiency Matrix (SSM-D) to inform allocation decisions to public mental health care for homeless people.	-EXCLUDE on intervention (service engagement/helpseeking behaviour)	
####	LaWall (2019) (ID:87848447)	Living Alone and Homelessness as Predictors of 30-Day Potentially Preventable Hospital Readmission.	-EXCLUDE on intervention (service engagement/helpseeking behaviour)	
####	Lawton (2016) (ID:87850936)	Comparison of demographic factors among homeless youth: New York and California.	-EXCLUDE on population (no gender focus; women population <50)<br>-EXCLUDE - but review for literature	
####	Lazear (2012) (ID:87851219)	The resilience u-turn: Understanding risks and strengths to effectively support LGBT youth and families in systems of care.	-EXCLUDE on population (people aged under 18 years)	
####	le Couteur (2023) (ID:87850519)	Gender and homelessness : a qualitative exploration into the lives of single adult women experiencing homelessness in the North West of England	-INCLUDE on title & abstract	-EXCLUDE on intervention (intersectionality)
####	Lea (2023) (ID:87848132)	Mental health and substance use screening in HIV primary care before and during the early COVID-19 pandemic.	-EXCLUDE on intervention (service engagement/helpseeking behaviour)	
####	Leal (2021) (ID:87857486)	Addressing smoking cessation among women in substance use treatment: A qualitative approach to guiding tailored interventions	-EXCLUDE on population (no housing precarity)	
####	LEARNER (2006) (ID:87857105)	A home from home	-EXCLUDE on population (people aged under 18 years)	
####	Lebrun-Harris (2013) (ID:87849068)	Health status and health care experiences among homeless patients in federally supported health centers: findings from the 2009 patient survey.	-EXCLUDE on intervention (service engagement/helpseeking behaviour)	
####	Leclair (2020) (ID:87850696)	Pathways to recovery among homeless people with mental illness: Is impulsiveness getting in the way?	-EXCLUDE on population (women)	
####	Leclere (2012) (ID:87853054)	The Jornalero: Perceptions of Health Care Resources of Immigrant Day Laborers	-EXCLUDE on intervention (service engagement/helpseeking behaviour)	
####	Leddy (2014) (ID:87848932)	Health and well-being of homeless veterans participating in transitional and supported employment: Six-month outcomes.	-EXCLUDE on intervention (service engagement/helpseeking behaviour)	
####	Leddy (2019) (ID:87853172)	Poverty stigma is associated with suboptimal HIV care and treatment outcomes among women living with HIV in the United States	-INCLUDE on title & abstract	-EXCLUDE on target group (no housing precarity)
####	Ledingham (2022) (ID:87963228)	Perspectives of adults with disabilities and opioid misuse: Qualitative findings illuminating experiences with stigma and substance use treatment	-EXCLUDE on population (no housing precarity)	
####	Ledwaba (2012) (ID:87851960)	Informal Settlements and Organisations in Post-Apartheid South Africa: The Case of Bethlehem, Tshwane	-EXCLUDE on country (High-Income)	
####	LEE (1996) (ID:87857188)	A constructivist approach to the help-seeking process of clients: a response to cultural diversity	-EXCLUDE on date (2010)	

####	LEE (2009) (ID:87857308)	Financial abuse in elderly Korean immigrants: mixed analysis of the role of culture on perception and help-seeking intention	-EXCLUDE on date (2010)	
####	Lee (2010) (ID:87849320)	The new homelessness revisited	-EXCLUDE on intervention (service engagement/helpseeking behaviour)	
####	Lee (2010) (ID:87851391)	Mental health care on the streets: An integrated approach.	-EXCLUDE on intervention (service engagement/helpseeking behaviour)	
####	Lee (2012) (ID:87849126)	Social support and mobility limitation as modifiable predictors of improvement in depressive symptoms in the elderly: results of a national longitudinal study.	-EXCLUDE on population (no housing precarity)	
####	Lee (2012) (ID:87851225)	Family homelessness viewed through the lens of health and human rights.	-EXCLUDE on intervention (service engagement/helpseeking behaviour)	
####	Lee (2012) (ID:87852035)	Influencing positive financial behaviors: the social marketing solution	-EXCLUDE on population (no housing precarity)	
####	Lee (2012) (ID:87853394)	Mortality in diabetes compared with previous cardiovascular disease: A gender-specific meta-analysis	-EXCLUDE on population (no housing precarity)	
####	Lee (2013) (ID:87848996)	A tale of two veterans: homeless vs domiciled veterans presenting to a psychiatric urgent care clinic.	-EXCLUDE on intervention (service engagement/helpseeking behaviour)	
####	Lee (2014) (ID:87848913)	Promoting recovery via an integrated model of care to deliver a bed-based, mental health prevention and recovery centre.	-EXCLUDE on population (women)	
####	LEE (2014) (ID:87857263)	Perception and help-seeking intention of intimate partner violence in later life: an international perspective	-EXCLUDE on intervention (service engagement/helpseeking behaviour)	
####	LEE (2014) (ID:87857342)	Elder mistreatment, culture, and help-seeking: a cross-cultural comparison of older Chinese and Korean immigrants	-EXCLUDE on population (no housing precarity)	
####	LEE (2014) (ID:87857344)	Elder mistreatment among Chinese and Korean immigrants: the roles of sociocultural contexts on perceptions and help-seeking behaviors	-EXCLUDE Duplicate	
####	LEE (2018) (ID:87857270)	Financial abuse in elderly Korean immigrants: mixed analysis of the role of culture on perception and help-seeking intention	-EXCLUDE on population (no housing precarity)	
####	Lee (2021) (ID:87853371)	Bidirectional Relationship Between Food Insecurity and Housing Instability	-EXCLUDE on intervention (service engagement/helpseeking behaviour)	
####	Lee (2023) (ID:87963333)	Race, class, and gender identity: implications for transgender people's police help seeking	-INCLUDE on title & abstract	-EXCLUDE on target group (no housing precarity)
####	Leidel (2017) (ID:87848647)	'We didn't have to dance around it': opt-out HIV testing among homeless and marginalised patients.	-EXCLUDE on population (women)	
####	Lemieux-Cumberlege (2019) (ID:87850723)	An exploratory study on the factors affecting the mental health and well-being of frontline workers in homeless services.	-EXCLUDE on intervention (service engagement/helpseeking behaviour)	
####	Lemoine (2021) (ID:87849318)	Cost-effectiveness analysis of housing first intervention with an independent housing and team support for homeless people with severe mental illness: A Markov model informed by a randomized controlled trial	-EXCLUDE on intervention (service engagement/helpseeking behaviour)	
####	Lenhard (2022) (ID:87849316)	MAKING BETTER LIVES: Hope, Freedom and Home-Making among People Sleeping Rough in Paris	-EXCLUDE on intervention (service engagement/helpseeking behaviour)<br>-EXCLUDE - but review for literature	
####	LENS (2018) (ID:87857125)	Asking for help: a qualitative study of barriers to help seeking in the private sector	-EXCLUDE on population (no housing precarity)	
####	Lenzi (2021) (ID:87849331)	Factors Associated with Providers' Work Engagement and Burnout in Homeless Services: A Cross-national Study	-EXCLUDE on intervention (service engagement/helpseeking behaviour)	
####	Leon (2018) (ID:87850830)	Changes in public order after the opening of an overdose monitoring facility for people who inject drugs.	-EXCLUDE on intervention (service engagement/helpseeking behaviour)	
####	Leonard (2018) (ID:87852823)	Theoretically-Based Emotion Regulation Strategies Using a Mobile App and Wearable Sensor Among Homeless Adolescent Mothers: Acceptability and Feasibility Study	-INCLUDE on title & abstract	-EXCLUDE on intervention (too programme-specific; not generalisable)
####	LEONE (2014) (ID:87857218)	Women's decisions to not seek formal help for partner violence: a comparison of intimate terrorism and situational couple violence	-INCLUDE on title & abstract	-EXCLUDE (IPV/DVA but little discussion on housing)
####	Leslie (2016) (ID:87857557)	Sharing Our Stories With the Future Healthcare Workforce: Perspectives of Low-Income Poverty Simulation Volunteers	-EXCLUDE on population (no housing precarity)	
####	LEUNG (2012) (ID:87857173)	Asian Indians and depressive symptoms: reframing mental health help-seeking behaviour	-EXCLUDE on population (no housing precarity)	

####	Levac (2022) (ID:87963300)	Transforming public policy with engaged scholarship: better together	-EXCLUDE on intervention (service engagement/helpseeking behaviour)	
####	Leven (2020) (ID:87851982)	Invisible Social Identity in the Workplace: Narrative Research Exploring How Experiences of Autistic Adults Influence Their Decision to Disclose	-EXCLUDE on population (no housing precarity)	
####	LEVENSON (2017) (ID:87857241)	Obstacles to help-seeking for sexual offenders: implications for prevention of sexual abuse	-EXCLUDE on intervention (service engagement/helpseeking behaviour)	
####	Levin (2014) (ID:87848901)	Comparing medication attitudes and reasons for medication nonadherence among three disparate groups of individuals with serious mental illness.	-EXCLUDE on intervention (service engagement/helpseeking behaviour)	
####	Levine (2018) (ID:87963061)	Disconnected lives: Women with intellectual disabilities in conflict with the law	-INCLUDE on title & abstract	-EXCLUDE on target group (no housing precarity)
####	Levitt (2012) (ID:87851255)	Impact of chronically street homeless tenants in congregate supportive housing.	-EXCLUDE on intervention (service engagement/helpseeking behaviour)	
####	Lewinson (2014) (ID:87853009)	Traumatic Transitions Homeless Women's Narratives of Abuse, Loss, and Fear	-INCLUDE on title & abstract	-EXCLUDE on intervention (intersectionality)
####	Lewis (2020) (ID:87853092)	Nonresident Fathers' Spending on Children: Child Support Payments and Housing Instability	-EXCLUDE on intervention (service engagement/helpseeking behaviour)	
####	Li (2016) (ID:87857560)	Engaging African-Caribbean, Asian, and Latino community leaders to address HIV stigma in Toronto	-EXCLUDE on intervention (service engagement/helpseeking behaviour)	
####	Li (2016) (ID:87858063)	An exploration of digital media use, community connectedness, and sexual orientation identity development among young men who have sex with men	-EXCLUDE on population (women)	
####	Li (2018) (ID:87851717)	Long-Term Survival Rates of Patients with Stage III–IV Hodgkin Lymphoma According to Age, Sex, Race, and Socioeconomic Status, 1984–2013.	-EXCLUDE on intervention (service engagement/helpseeking behaviour)	
####	Liamputtong (2022) (ID:87850581)	Handbook of social inclusion: Research and practices in health and social sciences.	-EXCLUDE on evidence and form (evidence not in written form or presented as research output)	
####	Lian (2017) (ID:87963073)	Predictors of depression/anxiety, mental health service utilization, and help-seeking for Chinese international students: Role of acculturation, microaggressions, social support, coping self-efficacy, stigma, and college staffs' cultural competence and cultural humility	-EXCLUDE on population (no housing precarity)	
####	Lidia (2022) (ID:87851478)	Primary health care, access to legal abortion and the notion of ideal victim among medical practitioners: The case of Chile	-EXCLUDE on country (High-Income)	
####	Liendo (2011) (ID:87849204)	Victimization and revictimization among women of Mexican descent.	-EXCLUDE on population (no housing precarity)	
####	Lightfoot (2011) (ID:87849167)	Protective factors associated with fewer multiple problem behaviors among homeless/runaway youth.	-EXCLUDE on intervention (service engagement/helpseeking behaviour)	
####	Lim (2018) (ID:87848549)	Impact of a New York City Supportive Housing Program on Housing Stability and Preventable Health Care among Homeless Families.	-EXCLUDE on intervention (service engagement/helpseeking behaviour)	
####	Lim (2019) (ID:87848436)	Supportive Housing and Its Relationship With Diabetes Diagnosis and Management Among Homeless Persons in New York City.	-EXCLUDE on intervention (service engagement/helpseeking behaviour)	
####	Lim (2023) (ID:87848137)	Association between jail-based methadone or buprenorphine treatment for opioid use disorder and overdose mortality after release from New York City jails 2011-17.	-EXCLUDE on intervention (service engagement/helpseeking behaviour)	
####	Lim (2023) (ID:87857749)	Factors Influencing Recovery and Well-Being Among Asian Survivors of International Criminal Sex Trafficking in an Urban U.S. City	-INCLUDE on title & abstract	-EXCLUDE on target group (no housing precarity)
####	Lima (2020) (ID:87850669)	People experiencing homelessness: Their potential exposure to COVID-19.	-EXCLUDE on intervention (service engagement/helpseeking behaviour)	
####	Lin (2015) (ID:87848818)	Frequent Emergency Department Visits and Hospitalizations Among Homeless People With Medicaid: Implications for Medicaid Expansion.	-EXCLUDE on intervention (service engagement/helpseeking behaviour)	
####	Lindan (2019) (ID:87857429)	Beyond a Socially Just Profession: Perspectives of Music Therapists in Canada	-EXCLUDE on population (no housing precarity)	
####	LINDEN (2013) (ID:87857022)	Addiction in maternity: prevalence of mental illness, substance use, and trauma	-EXCLUDE on intervention (service engagement/helpseeking behaviour)	

####	LINDSAY (2010) (ID:87857372)	In a lonely place? Social networks, job seeking and the experience of long-term unemployment	-EXCLUDE on population (no housing precarity)	
####	Lindsay (2019) (ID:87852830)	Gender matters in the transition to employment for young adults with physical disabilities	-EXCLUDE on population (no housing precarity)	
####	LINDSEY (2006) (ID:87857287)	Help-seeking behaviors and depression among African American adolescent boys	-EXCLUDE on date (2010)	
####	Lindsey (2015) (ID:87857704)	Let Me Blow Your Mind: Hip Hop Feminist Futures in Theory and Praxis	-EXCLUDE on intervention (service engagement/helpseeking behaviour)	
####	Lindskog (2022) (ID:87853214)	Acute healthcare utilization in end-of-life among Swedish brain tumor patients - a population based register study	-EXCLUDE on population (no housing precarity)	
####	Linton (2014) (ID:87848975)	Factors associated with the health service utilization of unsheltered, chronically homeless adults.	-EXCLUDE on population (no gender focus; women population <50)	
####	Linton (2016) (ID:87848777)	People and places: Relocating to neighborhoods with better economic and social conditions is associated with less risky drug/alcohol network characteristics among African American adults in Atlanta, GA.	-EXCLUDE on intervention (service engagement/helpseeking behaviour)	
####	Lipkin (2017) (ID:87851788)	The Health of Victims of Human Trafficking Victims in San Diego, California: A Retrospective Study.	-EXCLUDE on intervention (service engagement/helpseeking behaviour)	
####	LIPMANN (2009) (ID:87856981)	Elderly homeless men and women: aged care's forgotten people	-EXCLUDE on date (2010)	
####	Lippert (2015) (ID:87849317)	Stress, Coping, and Mental Health Differences among Homeless People	-EXCLUDE on population (women)	
####	Lippert (2021) (ID:87852989)	Adult and Child Food Insecurity Among Homeless and Precariously-Housed Families at the Close of the Twentieth Century	-EXCLUDE on population (women)	
####	LIPPMAN (2010) (ID:87857352)	The current status and impact of US National Policy on the help-seeking behavior of adolescents	-EXCLUDE on intervention (service engagement/helpseeking behaviour)	
####	Lippy (2020) (ID:87963388)	The impact of mandatory reporting laws on survivors of intimate partner violence: intersectionality, help-seeking and the need for change	-EXCLUDE on intervention (service engagement/helpseeking behaviour)<br>-EXCLUDE - but review for literature	
####	Little (2021) (ID:87857831)	Breast Cancer Screening Practices Among African American Women: A Black Feminist Thought Perspective	-EXCLUDE on population (no housing precarity)	
####	Littler (2022) (ID:87851966)	From the Battlefield to the Classroom: An Exploration of Post-9/11 Female Combat Veterans Who Completed Graduate School After Military Service	-EXCLUDE on intervention (service engagement/helpseeking behaviour)	
####	Liu (2021) (ID:87848226)	Overall and Gender-specific Associations between Dimensions of Adverse Childhood Experiences and Mental Health Outcomes among Homeless Adults: Associations Generales et Sexospecifiques Entre les Dimensions des Experiences Defavorables de L'enfance et les Resultats de Sante Mentale Chez les Adultes Sans Abri.	-EXCLUDE on intervention (service engagement/helpseeking behaviour)	
####	Liu (2023) (ID:87857454)	Intimate Partner Violence Screening for Veterans Accessing Homelessness Services	-EXCLUDE on intervention (service engagement/helpseeking behaviour)	
####	Livingston (2011) (ID:87849209)	'Forensic' labelling: an empirical assessment of its effects on self-stigma for people with severe mental illness.	-EXCLUDE on intervention (service engagement/helpseeking behaviour)	
####	LOCAL (2017) (ID:87857226)	Partnership approaches to improving health outcomes for young people	-EXCLUDE on intervention (service engagement/helpseeking behaviour)	
####	LOCAL (2020) (ID:87857278)	Lessons learnt from councils' response to rough sleeping during the COVID-19 pandemic	-EXCLUDE on intervention (service engagement/helpseeking behaviour)	
####	Lockwood (2022) (ID:87849328)	Homelessness in autistic women: Defining the research agenda	-INCLUDE on title & abstract	-EXCLUDE on intervention (service engagement/helpseeking behaviour)
####	Logan (2016) (ID:87848773)	The Sociospatial Network: Risk and the Role of Place in the Transmission of Infectious Diseases.	-EXCLUDE on intervention (service engagement/helpseeking behaviour)	
####	Logie (2016) (ID:87848708)	'Life under the tent is not safe, especially for young women': understanding intersectional violence among internally displaced youth in Leogane, Haiti.	-EXCLUDE on country (High-Income)	
####	Logie (2016) (ID:87848720)	Prevalence and Correlates of HIV Infection and HIV Testing Among Transgender Women in Jamaica.	-EXCLUDE on country (High-Income)	
####	Logie (2017) (ID:87848652)	Factors associated with sex work involvement among transgender women in Jamaica: a cross-sectional study.	-EXCLUDE on country (High-Income)	

####	Logie (2017) (ID:87851931)	Associations between Police Harassment and HIV Vulnerabilities among Men Who Have Sex with Men and Transgender Women in Jamaica	-EXCLUDE on country (High-Income)		
####	Logie (2018) (ID:87850793)	Factors associated with the separate and concurrent experiences of food and housing insecurity among women living with HIV in Canada.	-EXCLUDE on intervention (service engagement/helpseeking behaviour)		
####	Logie (2018) (ID:87853315)	HIV-related stigma, racial discrimination, and gender discrimination: Pathways to physical and mental health-related quality of life among a national cohort of women living with HIV	-EXCLUDE on population (no housing precarity)		
####	Logie (2018) (ID:87853367)	Social-ecological factors associated with selling sex among men who have sex with men in Jamaica: results from a cross-sectional tablet-based survey	-EXCLUDE on country (High-Income)		
####	Loison-Leruste (2020) (ID:87853182)	Homelessness and social work for women. An analysis of the film Les Invisibles	-EXCLUDE on intervention (service engagement/helpseeking behaviour)		
####	Lokot (2020) (ID:87963102)	Intersectionality as a lens to the COVID-19 pandemic: implications for sexual and reproductive health in development and humanitarian contexts	-EXCLUDE on population (no housing precarity)		
####	Lombardi (2020) (ID:87848349)	Findings of a national dataset analysis on the visits of homeless patients to US emergency departments during 2005-2015.	-EXCLUDE on intervention (service engagement/helpseeking behaviour)		
####	Lombe (2012) (ID:87851959)	Exploring Barriers to Inclusion of Widowed and Abandoned Women through Microcredit Self-Help Groups: The Case of Rural South India	-EXCLUDE on country (High-Income)		
####	Lombe (2016) (ID:87848757)	Examining effects of food insecurity and food choices on health outcomes in households in poverty.	-EXCLUDE on intervention (service engagement/helpseeking behaviour)		
####	Long-acting Reversible... (Gawron) (ID:87849343)	Long-acting Reversible Contraception Among Homeless Women Veterans With Chronic Health Conditions A Retrospective Cohort Study	-EXCLUDE on intervention (service engagement/helpseeking behaviour)		
####	Looijmans (2017) (ID:87848658)	Design of the Lifestyle Interventions for severe mentally ill Outpatients in the Netherlands (LION) trial; a cluster randomised controlled study of a multidimensional web tool intervention to improve cardiometabolic health in patients with severe mental illness.	-EXCLUDE on population (no housing precarity)		
####	Looijmans (2019) (ID:87848377)	Multimodal lifestyle intervention using a web-based tool to improve cardiometabolic health in patients with serious mental illness: results of a cluster randomized controlled trial (LION).	-EXCLUDE on intervention (service engagement/helpseeking behaviour)		
####	**Lopez (2014) (ID:87857807)**	**The paradoxes of poverty: Urban space and ideologies of intervention in the "compassionate city" of San Francisco**	**-INCLUDE on title & abstract**	**-INCLUDE on full study**	**--> published study included (López, Andrea M. "Necropolitics in the “compassionate” city: care/brutality in San Francisco." Medical anthropology 39.8 (2020): 751-764.)**
####	López (2016) (ID:87963166)	Health inequities, social determinants, and intersectionality	-EXCLUDE on intervention (service engagement/helpseeking behaviour)		
####	López (2020) (ID:87857643)	Integrating Latinx/Hispanic Culture, Traditions, and Beliefs into Effective School Psychology Practice	-EXCLUDE on population (no housing precarity)		
####	Loree (2019) (ID:87853413)	Disparity of Race Reporting and Representation in Clinical Trials Leading to Cancer Drug Approvals From 2008 to 2018	-EXCLUDE on population (no housing precarity)		
####	Lorenzetti (2023) (ID:87857876)	Immigrant Men and Racism in Canada: Impacts on Well-being and Family Wellness	-EXCLUDE on population (women)		
####	Lorine (2015) (ID:87848841)	Risk factors associated with psychiatric readmission.	-EXCLUDE on population (no housing precarity)		
####	Lorvick (2018) (ID:87848575)	Exploring Lifetime Accumulation of Criminal Justice Involvement and Associated Health and Social Outcomes in a Community-Based Sample of Women who Use Drugs.	-EXCLUDE on intervention (service engagement/helpseeking behaviour)		
####	Lorvick (2022) (ID:87852768)	Routine and preventive health care use in the community among women sentenced to probation	-EXCLUDE on intervention (service engagement/helpseeking behaviour)		
####	Loubiere (2020) (ID:87848282)	Determinants of healthcare use by homeless people with schizophrenia or bipolar disorder: results from the French Housing First Study.	-EXCLUDE on intervention (service engagement/helpseeking behaviour)		

####	Loubiere (2022) (ID:87848167)	Housing First for homeless people with severe mental illness: extended 4-year follow-up and analysis of recovery and housing stability from the randomized Un Chez Soi d'Abord trial.	-EXCLUDE on intervention (service engagement/helpseeking behaviour)	
####	Loue (2013) (ID:87851134)	Women.	-EXCLUDE on intervention (service engagement/helpseeking behaviour)	
####	Loue (2013) (ID:87851135)	Mental health practitioner's guide to HIV/AIDS.	-EXCLUDE on intervention (service engagement/helpseeking behaviour)	
####	Louie (2021) (ID:87963191)	For better or worse: When technology intersects with domestic violence and the lived experiences of Chinese immigrant women	-EXCLUDE on intervention (service engagement/helpseeking behaviour)	
####	Loutfy (2015) (ID:87857462)	Systematic review of stigma reducing interventions for African/Black diasporic women	-EXCLUDE on population (no housing precarity)	
####	LOVE (2012) (ID:87857042)	Social briefing 2012	-EXCLUDE on evidence and form (evidence not in written form or presented as research output)	
####	Lovett (2016) (ID:87850939)	Barriers to help seeking for lesbian victims of intimate partner violence.	-INCLUDE on title & abstract	-EXCLUDE (IPV/DVA but little discussion on housing)
####	Lovisi (2002) (ID:87853705)	Social disablement among residents of hostels for the homeless in Rio De Janeiro, Brazil	-EXCLUDE on date (2010)	
####	LOW (2011) (ID:87857234)	Help-seeking and service use for dementia in Italian, Greek and Chinese Australians	-EXCLUDE on population (no housing precarity)	
####	Low (2015) (ID:87852014)	Women's interpretation of and responses to potential gynaecological cancer symptoms: a qualitative interview study	-EXCLUDE on population (no housing precarity)	
####	Lowder (2017) (ID:87853166)	SSI/SSDI Outreach, Access, and Recovery (SOAR): Disability Application Outcomes Among Homeless Adults	-EXCLUDE on intervention (service engagement/helpseeking behaviour)	
####	Lowe (2011) (ID:87851342)	Reflections of a homeless population's lived experience with substance abuse.	-EXCLUDE on population (no gender focus; women population <50)	
####	Loweree (2015) (ID:87857601)	One size does not fit all looking beyond homeless housing-first and housing-ready approaches to enhanced models of the U.S.-Mexico border and New York City	-EXCLUDE on intervention (service engagement/helpseeking behaviour)	
####	LOWTHIAN (2000) (ID:87857103)	NACRO: housing needs of women prisoners	-EXCLUDE on date (2010)	
####	Lubman (2016) (ID:87848734)	Characteristics of individuals presenting to treatment for primary alcohol problems versus other drug problems in the Australian patient pathways study.	-EXCLUDE on intervention (service engagement/helpseeking behaviour)	
####	LUCAS (2021) (ID:87856914)	Homelessness among veterans: posttraumatic stress disorder, depression, physical health, and the cumulative trauma of military sexual assault	-EXCLUDE on intervention (service engagement/helpseeking behaviour)	
####	Lucas (2022) (ID:87857989)	LGBTQ+ Loss and Grief in a Cis-Heteronormative Pandemic: A Qualitative Evidence Synthesis of the COVID-19 Literature	-EXCLUDE on population (no housing precarity)	
####	Lucea (2013) (ID:87849060)	Factors influencing resource use by African American and African Caribbean women disclosing intimate partner violence.	-INCLUDE on title & abstract	-EXCLUDE on intervention (service engagement/helpseeking behaviour)
####	Lucenko (2015) (ID:87850993)	Childhood adversity and behavioral health outcomes for youth: An investigation using state administrative data.	-EXCLUDE on population (people aged under 18 years)	
####	Luchenski (2018) (ID:87857525)	What works in inclusion health: overview of effective interventions for marginalised and excluded populations	-EXCLUDE on intervention (service engagement/helpseeking behaviour)	
####	LUDVIGSEN (2004) (ID:87857353)	Searching for siblings: the motivations and experiences of adults seeking contact with adopted siblings	-EXCLUDE on date (2010)	
####	Ludwig (2018) (ID:87857915)	Racially Diverse Students' Perceptions of Caring Teacher-Student Relationships in Private Jewish Schools	-EXCLUDE on population (no housing precarity)	
####	Lund (2021) (ID:87850610)	Violence against LGBTQ+ persons: Research, practice, and advocacy.	-EXCLUDE on population (no housing precarity)	
####	Lund (2021) (ID:87857971)	Engaging Community Partners as Co-Educators in Teacher Education: Reflections from Pre-Service Teachers on a Justice-Based Service-Learning Program	-EXCLUDE on population (no housing precarity)	
####	Lusk (2019) (ID:87848444)	Bicycle Facilities Safest from Crime and Crashes: Perceptions of Residents Familiar with Higher Crime/Lower Income Neighborhoods in Boston.	-EXCLUDE on intervention (service engagement/helpseeking behaviour)	

####	Lutge (2015) (ID:87853335)	Incentives and enablers to improve adherence in tuberculosis	-EXCLUDE on intervention (service engagement/helpseeking behaviour)	
####	Luttges (2016) (ID:87853338)	Implications of chilean legal framework in teen pregnancy prevention: conflict and insecurity in health professionals	-EXCLUDE on country (High-Income)	
####	LUU (2009) (ID:87857228)	Help-seeking attitudes among Vietnamese Americans: the impact of acculturation, cultural barriers and spiritual beliefs	-EXCLUDE on date (2010)	
####	Ly (2014) (ID:87853271)	ETHNICITY AS A PREDICTIVE FACTOR FOR HEPATOCELLULAR CARCINOMA SCREENING AMONG PATIENTS IN HAWAII	-EXCLUDE on population (no housing precarity)	
####	Lynn (2018) (ID:87857731)	Red Dirt Resistance: Oklahoma Educators as Agents of Change	-EXCLUDE on population (no housing precarity)	
####	**Lyons (2016) (ID:87848724)**	**Experiences of Trans Women and Two-Spirit Persons Accessing Women-Specific Health and Housing Services in a Downtown Neighborhood of Vancouver, Canada.**	**-INCLUDE on title & abstract**	**-INCLUDE on full study**
####	Lyons (2016) (ID:87848798)	Women Who Use Drugs and Have Sex with Women in a Canadian Setting: Barriers to Treatment Enrollment and Exposure to Violence and Homelessness.	-INCLUDE on title & abstract	-EXCLUDE on target group (no housing precarity)
####	Mabrey (2020) (ID:87857694)	From Single Mom to Supermom: A Transformative Journey	-EXCLUDE on intervention (service engagement/helpseeking behaviour)	
####	MacDermid (2016) (ID:87850911)	War and family life.	-EXCLUDE on intervention (service engagement/helpseeking behaviour)	
####	MACDONALD (2020) (ID:87857086)	Protecting women with multiple and complex needs from gendered violence: impediments to obtaining and maintaining safe and secure accommodation in a European context	-INCLUDE on title & abstract	-EXCLUDE on intervention (intersectionality)
####	Macdonald-Jarvis (2015) (ID:87858021)	From awareness to action in the elementary classroom: Developing culturally relevant content and pedagogy	-EXCLUDE on population (no housing precarity)	
####	Macedo (2023) (ID:87850557)	Health and self-care from the perspective of institutionalized adolescents.	-EXCLUDE on country (High-Income)	
####	Mackelprang (2014) (ID:87848930)	Housing First is associated with reduced use of emergency medical services.	-EXCLUDE on intervention (service engagement/helpseeking behaviour)	
####	MACKENZIE (2006) (ID:87857204)	Age, gender, and the underutilization of mental health services: the influence of help-seeking attitudes	-EXCLUDE on date (2010)	
####	Mackesy-Amiti (2018) (ID:87853434)	Feasibility of ecological momentary assessment to study mood and risk behavior among young people who inject drugs	-EXCLUDE on intervention (service engagement/helpseeking behaviour)	
####	Mackie (2014) (ID:87851432)	Nations apart? Experiences of single homeless people across Great Britain	-EXCLUDE on intervention (service engagement/helpseeking behaviour)	
####	MacLEAN (2013) (ID:87857378)	Symptoms of mental health problems: children's and adolescents' understandings and implications for gender differences in help seeking	-EXCLUDE on population (women)	
####	Maclean (2015) (ID:87851681)	Primary care characteristics and stage of cancer at diagnosis using data from the national cancer registration service, quality outcomes framework and general practice information	-EXCLUDE on population (no housing precarity)	
####	MacLean (2018) (ID:87848577)	Tobacco and alcohol use disorders: Evaluating multimorbidity.	-EXCLUDE on intervention (service engagement/helpseeking behaviour)	
####	MacLellan (2017) (ID:87848604)	Using peer advocates to improve access to services among hard-to-reach populations with hepatitis C: a qualitative study of client and provider relationships.	-EXCLUDE on population (women)	
####	MacNeil (2011) (ID:87849217)	Needle exchange as a safe haven in an unsafe world.	-EXCLUDE on intervention (service engagement/helpseeking behaviour)	
####	MACY (2005) (ID:87857288)	Battered women's profiles associated with service help-seeking efforts: illuminating opportunities for intervention	-EXCLUDE on date (2010)	
####	Madigan (2021) (ID:87848211)	Health Care Utilization of Individuals Affected by Homelessness: Illinois, 2011-2018.	-EXCLUDE on intervention (service engagement/helpseeking behaviour)	
####	Madsen (2013) (ID:87851844)	Changes in inpatient and postdischarge suicide rates in a nationwide cohort of Danish psychiatric inpatients, 1998-2005.	-EXCLUDE on population (no housing precarity)	

####	**Magill (2023) (ID:87850558)**	**The "epidemic within the pandemic": Meeting the needs of racially minoritised women experiencing domestic abuse during the Covid-19 pandemic.**	**-INCLUDE on title & abstract**	**-INCLUDE on full study**
####	Mago (2018) (ID:87851793)	A Model Pathway to Oral Health Care for Homeless People.	-EXCLUDE on intervention (service engagement/helpseeking behaviour)	
####	Magwood (2019) (ID:87849337)	Common trust and personal safety issues: A systematic review on the acceptability of health and social interventions for persons with lived experience of homelessness	-EXCLUDE on population (women)<br>-EXCLUDE - but review for literature	
####	Magwood (2020) (ID:87848256)	Determinants of Implementation of a Clinical Practice Guideline for Homeless Health.	-EXCLUDE on intervention (service engagement/helpseeking behaviour)	
####	Mahajne (2023) (ID:87852703)	Critical Practices Adopted by Minority Group Social Workers Working in the Third Sector	-EXCLUDE on intervention (service engagement/helpseeking behaviour)	
####	Mahure (2018) (ID:87848550)	Risk of Complications After THA Increases Among Patients Who Are Coinfected With HIV and Hepatitis C.	-EXCLUDE on population (no housing precarity)	
####	Maierhofer (2011) (ID:87849162)	Prevalence and features of ICF-disability in Spain as captured by the 2008 National Disability Survey.	-EXCLUDE on intervention (service engagement/helpseeking behaviour)	
####	Maiorana (2021) (ID:87857565)	“She is Like a Sister to Me.” Gender-Affirming Services and Relationships are Key to the Implementation of HIV Care Engagement Interventions with Transgender Women of Color	-EXCLUDE on population (no housing precarity)	
####	MAITER (2009) (ID:87857394)	The experiences of minority immigrant families receiving child welfare services: seeking to understand how to reduce risk and increase protective factors	-EXCLUDE on date (2010)	
####	Maiton (2021) (ID:87851533)	Spatial and spatio-temporal distribution of women living with HIV mortality in Porto Alegre, Brazil, from 2007 to 2017	-EXCLUDE on country (High-Income)	
####	Máiz-Mazuela (2023) (ID:87857830)	Essential Workers Balancing Life and Work during the COVID-19 Syndemic in Spain: A Qualitative and Gender-Based Study	-EXCLUDE on population (no housing precarity)	
####	Major (2016) (ID:87848750)	Changes in Dental Students' Attitudes About Treating Underserved Populations: A Longitudinal Study.	-EXCLUDE on population (no housing precarity)	
####	Makurumidze (2022) (ID:87850594)	HIV stigma.	-EXCLUDE on intervention (service engagement/helpseeking behaviour)	
####	Malaret (2013) (ID:87858062)	"Sticks and stones...": A Qualitative Exploration of the Resilience Process of Transgender and Gender Fluid Persons: A Research Proposal	-EXCLUDE on evidence and form (evidence not in written form or presented as research output)	
####	Malekinejad (2015) (ID:87848885)	High HIV prevalence in a respondent-driven sampling survey of injection drug users in Tehran, Iran.	-EXCLUDE on country (High-Income)	
####	Malekoff (2014) (ID:87851103)	From the editor.	-EXCLUDE on evidence and form (evidence not in written form or presented as research output)	
####	Malik (2018) (ID:87848588)	Vulnerability of Older Adults in Disasters: Emergency Department Utilization by Geriatric Patients After Hurricane Sandy.	-EXCLUDE on intervention (service engagement/helpseeking behaviour)	
####	Mall (2017) (ID:87848702)	'Restoring the person's life': a qualitative study to inform development of care for people with severe mental disorders in rural Ethiopia.	-EXCLUDE on country (High-Income)	
####	Malla (2019) (ID:87848462)	Canadian response to need for transformation of youth mental health services: ACCESS Open Minds (Esprits ouverts).	-EXCLUDE on intervention (service engagement/helpseeking behaviour)	
####	Mallett (2004) (ID:87849360)	Practising Homelessness: A Typology Approach to Young People's Daily Routines	-EXCLUDE on date (2010)	
####	Mallo (2018) (ID:87857957)	Dying to Be a Man: A Mixed Methods Study Implementing Men's Health Promotion and the Psychology of Masculinity in Graduate Primary Care Curriculum	-EXCLUDE on population (no housing precarity)	
####	Mallon (2014) (ID:87851056)	Child welfare for the twenty-first century: A handbook of practices, policies, and programs.	-EXCLUDE on population (people aged under 18 years)	
####	Malott (2019) (ID:87857955)	Anxieties toward Outgroup Members: Use of an (Elaborated) Imagined Contact Intervention with Undergraduate Students	-EXCLUDE on population (no housing precarity)	
####	Malpas (2022) (ID:87857850)	Family-Based Interventions with Transgender and Gender Expansive Youth: Systematic Review and Best Practice Recommendations	-EXCLUDE on population (no housing precarity)	
####	Malqvist (2011) (ID:87852755)	Neonatal mortality: an invisible and marginalised trauma	-EXCLUDE on country (High-Income)	
####	Malqvist (2012) (ID:87852817)	Causes and determinants of inequity in maternal and child health in Vietnam	-EXCLUDE on country (High-Income)	

####	Malte (2017) (ID:87848640)	Providing intensive addiction/housing case management to homeless veterans enrolled in addictions treatment: A randomized controlled trial.	-EXCLUDE on intervention (service engagement/helpseeking behaviour)	
####	Mama (1989) (ID:87853713)	Violence against Black Women: Gender, Race and State Responses	-EXCLUDE on date (2010)	
####	MAN (2019) (ID:87857152)	Service satisfaction and helpfulness ratings, mental health literacy and help seeking barriers of carers of individuals with dual disabilities	-EXCLUDE on population (no housing precarity)	
####	Mancini (2013) (ID:87849086)	Consumer and practitioner perceptions of the harm reduction approach in a community mental health setting.	-EXCLUDE on intervention (service engagement/helpseeking behaviour)	
####	Mandal (2022) (ID:87851459)	Feminism, Sexuality, Gender, Labour: Invisible Stigma of Sex Work and Menstrual Labour in India.	-EXCLUDE on country (High-Income)	
####	Mandel (2019) (ID:87857900)	Building on the Greenbook: A Perpetrator Pattern-Based Approach to Improve Child Welfare's Response to Domestic Violence	-EXCLUDE on evidence and form (evidence not in written form or presented as research output)	
####	Mandel (2021) (ID:87851761)	Health Care by Age, Gender and Class	-EXCLUDE on intervention (service engagement/helpseeking behaviour)	
####	Maness (2014) (ID:87848936)	Care of the homeless: an overview.	-EXCLUDE on population (no gender focus; women population <50)	
####	Mankowski (2017) (ID:87851596)	Aging LGBT Military Service Members and Veterans.	-EXCLUDE on population (no housing precarity)	
####	Manne (2016) (ID:87857764)	THE LOGIC OF MISOGYNY	-EXCLUDE on population (no housing precarity)	
####	Manning (2018) (ID:87848568)	Microsystems of Recovery in Homeless Services: The Influence of Service Provider Values on Service Users' Recovery Experiences.	-EXCLUDE on intervention (service engagement/helpseeking behaviour)	
####	Manning (2019) (ID:87848448)	Recovery in homelessness: The influence of choice and mastery on physical health, psychiatric symptoms, alcohol and drug use, and community integration.	-EXCLUDE on population (no gender focus; women population <50)	
####	Mansa (2020) (ID:87963268)	Coping strategies of women intimate partner violence survivors: Perspectives of service providers	-INCLUDE on title & abstract	-EXCLUDE (IPV/DVA but little discussion on housing)
####	Mansell (2013) (ID:87849058)	Implementation of active support in Victoria, Australia: an exploratory study.	-EXCLUDE on population (no housing precarity)	
####	MANSFIELD (2008) (ID:87857139)	Understanding and increasing help-seeking in older men	-EXCLUDE on date (2010)	
####	Mansour (2022) (ID:87853326)	Disparities in Social Determinants of Health Among Patients Receiving Liver Transplant: Analysis of the National Inpatient Sample From 2016 to 2019	-EXCLUDE on population (no housing precarity)	
####	MANTHEI (2006) (ID:87857283)	Clients talk about their experience of seeking counselling	-EXCLUDE on date (2010)	
####	Mantler (2017) (ID:87963369)	A rural shelter in Ontario adapting to address the changing needs of women who have experienced intimate partner violence: a qualitative case study	-INCLUDE on title & abstract	-EXCLUDE on target group (not focused on women's behaviour/outcomes for women)
####	Mantler (2022) (ID:87850562)	Sharing personal experiences of accessibility and knowledge of violence: A qualitative study.	-INCLUDE on title & abstract	-EXCLUDE on target group (no housing precarity)
####	Manuel (2012) (ID:87852671)	"I Hope I Can Make it Out There": Perceptions of Women with Severe Mental Illness on the Transition from Hospital to Community	-EXCLUDE on population (no housing precarity)	
####	Manuel-Ebanks (2019) (ID:87857871)	An Exploratory Study of the Impact of Participating in Social Support Groups for Transgender and Gendernonconforming Youth	-EXCLUDE on population (no housing precarity)	
####	Mapping development... (2022) (ID:87851773)	Mapping development and health effects of cooking with solid fuels in low-income and middle-income countries, 2000-18: a geospatial modelling study.	-EXCLUDE on intervention (service engagement/helpseeking behaviour)	
####	Mapping the maze: services... (2017) (ID:87856957)	Mapping the maze: services for women experiencing multiple disadvantage in England and Wales	-EXCLUDE on intervention (service engagement/helpseeking behaviour)<br>-EXCLUDE - but review for literature	
####	Marble (2017) (ID:87852003)	Kevin Michael Key, advocate who found hope and sobriety on skid row, dies at 67	-EXCLUDE on evidence and form (evidence not in written form or presented as research output)	
####	Marcellus (2015) (ID:87857649)	Reenvisioning Success for Programs Supporting Pregnant Women With Problematic Substance Use	-EXCLUDE on population (no housing precarity)	
####	Marcellus (2017) (ID:87857675)	A Grounded Theory of Mothering in the Early Years for Women Recovering From Substance Use	-EXCLUDE on population (no housing precarity)	

####	Mares (2011) (ID:87849214)	A comparison of treatment outcomes among chronically homelessness adults receiving comprehensive housing and health care services versus usual local care.	-EXCLUDE on intervention (service engagement/helpseeking behaviour)	
####	Mares (2016) (ID:87848741)	Czech version of OPQOL-35 questionnaire: the evaluation of the psychometric properties.	-EXCLUDE on intervention (service engagement/helpseeking behaviour)	
####	Maretta (2018) (ID:87850843)	The role of gender, social support, previous trauma, and combat exposure in the development of PTSD.	-EXCLUDE on intervention (service engagement/helpseeking behaviour)	
####	Margolin (2017) (ID:87963271)	Using arts-based methods to create research spaces that encourage meaningful dialogue	-EXCLUDE on population (no housing precarity)	
####	Maria (2021) (ID:87851529)	Reproductive health among Venezuelan migrant women at the north western border of Brazil: A qualitative study	-EXCLUDE on country (High-Income)	
####	Marjadi (2023) (ID:87857524)	Twelve Tips for Inclusive Practice in Healthcare Settings	-EXCLUDE on population (women)	
####	Mark (2014) (ID:87851623)	Migration and the epidemiological transition: insights from the Agincourt sub-district of northeast South Africa	-EXCLUDE on country (High-Income)	
####	Markey (2022) (ID:88019144)	Service User and Service Provider Perceptions of Enablers and Barriers for Refugee and Asylum-Seeking Women Accessing and Engaging with Perinatal Mental Health Care Services in the WHO European Region: A Scoping Review Protocol	-INCLUDE on title & abstract	-EXCLUDE on evidence and form (evidence not in written form or presented as research output)
####	Marpsat (2018) (ID:87849300)	The homeless in Paris: A representative sample survey of users of services for the homeless	-EXCLUDE on intervention (service engagement/helpseeking behaviour)	
####	Marselian (2020) (ID:87857835)	La Maestra’s Circle of Care: Studying the Impact of an Integrated Service Model on the Health and Well-Being of Vulnerable Populations	-EXCLUDE on population (no gender focus; women population <50)	
####	Marshall (2016) (ID:87857768)	Refugee Youth: A Review of Mental Health Counselling Issues and Practices	-EXCLUDE on population (no housing precarity)	
####	Marshall (2022) (ID:87853455)	Experiences of transitioning from homelessness: a systematic review and meta-aggregation of qualitative studies conducted in middle to high income countries	-EXCLUDE on population (no gender focus; women population <50)<br>-EXCLUDE - but review for literature	
####	Marsiglia (2011) (ID:87849202)	Hopelessness, family stress, and depression among Mexican-heritage mothers in the southwest.	-EXCLUDE on population (no housing precarity)	
**####**	**Marti-Castaner (2022) (ID:87853073)**	**Poverty after Birth: How Mothers Experience and Navigate US Safety Net Programs to Address Family Needs**	**-INCLUDE on title & abstract**	**-INCLUDE on full study**
####	Martin (2011) (ID:87853446)	"Now we got lots to eat and they're telling us not to eat it": understanding changes to south-east Labrador Inuit relationships to food	-EXCLUDE on population (no housing precarity)	
####	Martin (2014) (ID:87857743)	"I hate to say it but the system was not designed for us to be successful" The underemployed, unestablished and unforgiven: The uninvited state of America; exploring a new praxis for felon employment through symbolic identification	-EXCLUDE on population (no housing precarity)	
####	Martin (2016) (ID:87848775)	Food insecurity and mental illness: disproportionate impacts in the context of perceived stress and social isolation.	-EXCLUDE on intervention (service engagement/helpseeking behaviour)	
####	Martin (2016) (ID:87850952)	Attitudes toward mental health services among homeless and matched housed youth.	-EXCLUDE on population (no housing precarity)	
####	Martin (2016) (ID:87857522)	Making practice inclusive in gender-based violence work	-EXCLUDE on population (no housing precarity)	
####	Martin (2016) (ID:87857782)	Digital Transformations?: Gendering the End User in Digital Government Policy	-EXCLUDE on population (no housing precarity)	
####	Martin (2019) (ID:87848401)	Adults with Housing Insecurity Have Worse Access to Primary and Preventive Care.	-EXCLUDE on intervention (service engagement/helpseeking behaviour)	
####	Martin-Fernandez (2018) (ID:87849306)	Food insecurity in homeless families in the paris region (France): Results from the ENFAMS survey	-EXCLUDE on population (no gender focus; women population <50)	
####	Martin-West (2019) (ID:87850729)	The role of social support as a moderator of housing instability in single mother and two-parent households.	-INCLUDE on title & abstract	-EXCLUDE on intervention (service engagement/helpseeking behaviour)
####	Martine (2021) (ID:87851502)	Double Jeopardy: Maintaining Livelihoods or Preserving Health? The Tough Choices Sex Workers Faced during the COVID-19 Pandemic	-EXCLUDE on intervention (service engagement/helpseeking behaviour)	
####	Martinelli (2019) (ID:87848416)	Mental health supported accommodation services in England and in Italy: a comparison.	-EXCLUDE on population (no housing precarity)	
####	Martinelli (2022) (ID:87850596)	Quality of residential facilities in Italy: Satisfaction and quality of life of residents with schizophrenia spectrum disorders.	-EXCLUDE on population (no housing precarity)	

####	Martinez (2020) (ID:87963319)	Bios, mythoi and women entrepreneurs: A Wynterian analysis of the intersectional impacts of the COVID-19 pandemic on self-employed women and women-owned businesses	-EXCLUDE on population (no housing precarity)	
####	Martini (2011) (ID:87853160)	Colorectal cancer screening in rural and remote areas: analysis of the National Bowel Cancer Screening Program data for South Australia	-EXCLUDE on population (no housing precarity)	
####	Martini (2019) (ID:87857948)	Migration, Institutions and Intimate Lives: Towards a New Agenda	-EXCLUDE on intervention (service engagement/helpseeking behaviour)	
####	Martino (2011) (ID:87849206)	Increased substance use and risky sexual behavior among migratory homeless youth: exploring the role of social network composition.	-EXCLUDE on population (women)	
####	Martino (2020) (ID:87963099)	Planning with care: Violence prevention policy at the intersection of invisibilities	-INCLUDE on title & abstract	-EXCLUDE on target group (not focused on women's behaviour/outcomes for women)
####	Martorell (2011) (ID:87849186)	Family impact in intellectual disability, severe mental health disorders and mental health disorders in ID. A comparison.	-EXCLUDE on population (no housing precarity)	
####	Marušić (2016) (ID:87963286)	Nowhere at home: Homelessness, non-heterosexuality, and LGBT activism in Croatia	-EXCLUDE on intervention (service engagement/helpseeking behaviour)	
####	Marya (2016) (ID:87851607)	Factors Associated with Recent HIV Testing among Heterosexuals at High-Risk for HIV Infection in New York City	-EXCLUDE on intervention (service engagement/helpseeking behaviour)	
####	Masa (2023) (ID:87848126)	Family Rejection, Socioeconomic Precarity, and Exchanging Sex for Food among Young Transgender Adults: Findings from the U.S. Transgender Survey.	-EXCLUDE on intervention (service engagement/helpseeking behaviour)	
####	Masanotti (2015) (ID:87853319)	Cancer burden trends in Umbria region using a joinpoint regression	-EXCLUDE on intervention (service engagement/helpseeking behaviour)	
####	Mason-Bish (2014) (ID:87963255)	Beyond the silo: Rethinking hate crime and intersectionality	-EXCLUDE on population (no housing precarity)	
####	Mason-Bish (2020) (ID:87857774)	'Some men deeply hate women, and express that hatred freely': Examining victims' experiences and perceptions of gendered hate crime	-EXCLUDE on population (no housing precarity)	
####	Massie (2016) (ID:87857712)	More Than a Shelter: Exploring the Impact of Housing Services among Women with HIV/AIDS in the District of Columbia	-INCLUDE on title & abstract	-EXCLUDE on intervention (intersectionality)
####	Massie (2018) (ID:87850844)	More than a shelter: Exploring the impact of housing services among women with HIV/AIDS in the district of Columbia.	-EXCLUDE Duplicate	
####	Masson (2020) (ID:87848296)	Clients' perceptions of barriers and facilitators to implementing hepatitis C virus care in homeless shelters.	-EXCLUDE on population (no gender focus; women population <50)	
####	Masson (2020) (ID:87849303)	Mental healthcare utilization among homeless people in the greater paris area	-EXCLUDE on population (women)	
####	Mastronardi (2023) (ID:87851574)	Increasing Contraception Options Immediately After Birth: Did Immediate Postpartum Long-Acting Reversible Contraception (IPP LARC) Utilization Increase During the COVID-19 Pandemic? [ID: 1377847].	-EXCLUDE on population (no housing precarity)	
####	Mateo-Rodriguez (2019) (ID:87848388)	Risk of suicide in households threatened with eviction: the role of banks and social support.	-EXCLUDE on intervention (service engagement/helpseeking behaviour)	
####	Matheson (2019) (ID:87851997)	The use of self-management strategies for problem gambling: a scoping review	-EXCLUDE on intervention (service engagement/helpseeking behaviour)	
####	Mathieson (2016) (ID:87857870)	Unnecessary and disproportionate: the outcomes of remand for indigenous young people according to service providers	-EXCLUDE on population (no housing precarity)	
####	MATOLCSI (2020) (ID:87857153)	Unwanted sex with third parties in domestic abuse relationships and its impact on help-seeking and justice	-INCLUDE on title & abstract	-EXCLUDE (IPV/DVA but little discussion on housing)
####	Matos (2023) (ID:87857817)	Utilizing Latinx Cultural Capital for the Retention and Graduation of Latinx Students in Higher Education	-EXCLUDE on population (no housing precarity)	
####	Matson (2015) (ID:87850985)	Mobile voice: A feminist strengths-based interpersonal approach to mobility intervention.	-EXCLUDE on intervention (service engagement/helpseeking behaviour)<br>-EXCLUDE - but review for literature	
####	Matsuzaka (2019) (ID:87857529)	Trans Feminine Sexual Violence Experiences: The Intersection of Transphobia and Misogyny	-EXCLUDE on intervention (service engagement/helpseeking behaviour)	
####	MATTHEW (2015) (ID:87857260)	Participatory action research on help-seeking behaviors of self-defined ritual abuse survivors: a brief report	-EXCLUDE on intervention (service engagement/helpseeking behaviour)	
####	Matthews (2018) (ID:87851415)	Your tenants are gay, get over it! Public services and governance	-EXCLUDE on population (no housing precarity)	

####	Matthews (2018) (ID:87851416)	Homelessness support for LGBT+ people: public services and governance	-EXCLUDE on intervention (service engagement/helpseeking behaviour)	
####	Matthews (2022) (ID:87848141)	Factors influencing the well-being of Asian American LGBT individuals across the lifespan: perspectives from leaders of community-based organizations.	-EXCLUDE on intervention (service engagement/helpseeking behaviour)	
####	Matulic-Domandzic (2019) (ID:87853162)	HOMELESS WOMEN: HIDDEN REALITIES OF RESIDENTIAL EXCLUSION	-INCLUDE on title & abstract	-EXCLUDE on evidence and form (evidence not in written form or presented as research output)
####	Maturi (2023) (ID:87963263)	Revisiting Empowerment Through Critical Praxis: Perspectives of Front-Line Workers Supporting Refugee Women Experiencing Gendered Violence in Australia	-EXCLUDE on intervention (service engagement/helpseeking behaviour)	
####	Maulsby (2018) (ID:87848591)	A Mixed-Methods Exploration of the Needs of People Living with HIV (PLWH) Enrolled in Access to Care, a National HIV Linkage, Retention and Re-Engagement in Medical Care Program.	-EXCLUDE on intervention (service engagement/helpseeking behaviour)	
####	Mawhinney (2020) (ID:87858116)	Teacher Identity Making, Shifting, and Resisting: The Case of Two Former Teach for America Corps Members	-EXCLUDE on population (no housing precarity)	
####	Maxfield (2019) (ID:87857569)	Harmed or Harmful: The Discourse of Trigger Warnings, Trauma, and Shelter	-EXCLUDE on population (no housing precarity)	
####	May (2014) (ID:87848946)	Low uptake of colorectal cancer screening among African Americans in an integrated Veterans Affairs health care network.	-EXCLUDE on intervention (service engagement/helpseeking behaviour)	
####	MAYBERRY (2013) (ID:87857266)	How caregivers make meaning of child mental health problems: toward understanding caregiver strain and help seeking	-EXCLUDE on population (people aged under 18 years)	
####	Mayberry (2019) (ID:87848394)	Text messaging to engage friends/family in diabetes self-management support: acceptability and potential to address disparities.	-EXCLUDE on intervention (service engagement/helpseeking behaviour)	
####	Mayberry (2019) (ID:87851990)	819-P: Personalized Texts Improve Adherence and A1c over 6 Months among High-Risk Adults with Type 2 Diabetes	-EXCLUDE on population (no housing precarity)	
####	Maye-Banbury (2011) (ID:87850524)	Women and homelessness : the relevance of European welfare regimes	-EXCLUDE on intervention (service engagement/helpseeking behaviour)	
####	Mayer (2011) (ID:87853152)	Introduction: Linkage, Engagement, and Retention in HIV Care: Essential for Optimal Individual- and Community-Level Outcomes in the Era of Highly Active Antiretroviral Therapy	-EXCLUDE on population (no housing precarity)	
####	Mayke (2015) (ID:87851575)	Kinderen buiten beeld. De leefsituatie van ongedocumenteerde kinderen in Nederland	-EXCLUDE on population (people aged under 18 years)	
####	Mayock (2015) (ID:88019147)	Women, Homelessness and Service Provision	-INCLUDE on title & abstract	-EXCLUDE on intervention (intersectionality)
####	Mayock (2016) (ID:88019123)	Women's Homelessness and Domestic Violence: (In)visible Interactions	-EXCLUDE on intervention (service engagement/helpseeking behaviour)	
####	Mayock (2017) (ID:88019119)	Women's Responses to Homelessness: FEANTSA 12th European Research Conference on Homelessness	-EXCLUDE on intervention (service engagement/helpseeking behaviour)<br>-EXCLUDE - but review for literature	
####	Mazereeuw (2018) (ID:87851597)	Cancer incidence and survival among Métis adults in Canada: results from the Canadian census follow-up cohort (1992-2009).	-EXCLUDE on population (no housing precarity)	
####	Mazul (2021) (ID:87853142)	Gender and race interact to influence survival disparities in head and neck cancer	-EXCLUDE on population (no housing precarity)	
####	Mazzawi (2022) (ID:87850604)	The relationship of healthiness, stigma, and social support to maternal functioning for postpartum women in treatment to support recovery for opioid use disorder (OUD).	-EXCLUDE on population (no housing precarity)	
####	MAZZER (2009) (ID:87857245)	Community gatekeepers' advice to young people to seek help from mental health professionals: youth workers and sport coaches	-EXCLUDE on date (2010)	
####	Mbonye (2022) (ID:87857666)	The meaning of fatherhood to men in relationships with female sex workers in Kampala, Uganda: The struggle to model the traditional parameters of fatherhood and masculinity	-EXCLUDE on country (High-Income)	
####	Mc Conalogue (2021) (ID:87963260)	Homeless people and health: a qualitative enquiry into their practices and perceptions	-EXCLUDE on intervention (service engagement/helpseeking behaviour)	

####	McCabe (2012) (ID:87849150)	Demographic and substance abuse trends among pregnant and non-pregnant women: eleven years of treatment admission data.	-EXCLUDE on intervention (service engagement/helpseeking behaviour)	
####	McCall (2018) (ID:87852957)	Characteristics and Health Needs of Veterans in Jails and Prisons: What We Know and Do Not Know about Incarcerated Women Veterans	-EXCLUDE on intervention (service engagement/helpseeking behaviour)	
####	McCANN (2014) (ID:87857336)	Who can I talk to? Self-harm and seeking help among 16 year olds: changes between 2008 and 2013	-EXCLUDE on population (people aged under 18 years)	
####	MCCANN (2021) (ID:87851411)	Homeless experiences and support needs of transgender people: a systematic review of the international evidence	-EXCLUDE on intervention (service engagement/helpseeking behaviour)<br>-EXCLUDE - but review for literature	
####	McCarthy (2010) (ID:87849244)	Access-related measures and out-of-system utilization among veterans with bipolar disorder.	-EXCLUDE on population (no housing precarity)	
####	McCarthy (2013) (ID:87963034)	Homelessness and identity: A critical review of the literature and theory	-EXCLUDE on population (no gender focus; women population <50)	
####	Mccarthy (2015) (ID:87850512)	(Re)negotiating the self : homeless women's constructions of home, homelessness and identity	-EXCLUDE on intervention (service engagement/helpseeking behaviour)<br>-EXCLUDE - but review for literature	
####	McCarthy (2017) (ID:87963362)	'I know it was every week, but I can't be sure if it was every day: Domestic violence and women with learning disabilities	-INCLUDE on title & abstract	-EXCLUDE (IPV/DVA but little discussion on housing)
####	Mccarthy (2020) (ID:87963090)	Understanding models of support for people facing multiple disadvantage: A Literature Review	-EXCLUDE - but review for literature<br>-EXCLUDE on population (women)	
####	McCaskill (2022) (ID:87857833)	African American Women and Tenant Management of Public Housing: A Case Study of Stella Wright Homes	-EXCLUDE on intervention (service engagement/helpseeking behaviour)	
####	McCay (2011) (ID:87849166)	A relationship-based intervention to improve social connectedness in street-involved youth: a pilot study.	-EXCLUDE on intervention (service engagement/helpseeking behaviour)	
####	McCLENNEN (2002) (ID:87857335)	Gay men's domestic violence: dynamics, help seeking behaviours and correlates	-EXCLUDE on date (2010)	
####	McClure (2018) (ID:87850784)	Agreement between self and psychiatrist reporting of suicidal ideation at a Veterans Administration psychiatric emergency clinic.	-EXCLUDE on intervention (service engagement/helpseeking behaviour)	
####	McConkey (2010) (ID:87849285)	Using personal goal setting to promote the social inclusion of people with intellectual disability living in supported accommodation.	-EXCLUDE on population (no housing precarity)	
####	McCormack (2015) (ID:87848887)	Voices of homeless alcoholics who frequent Bellevue Hospital: a qualitative study.	-EXCLUDE on intervention (service engagement/helpseeking behaviour)	
####	McCormack (2021) (ID:87853111)	The development and implementation of an advanced practice registered nurse-led prenatal education program for housing insecure women	-EXCLUDE on intervention (service engagement/helpseeking behaviour)	
####	McCormack (2022) (ID:88019155)	Women, homelessness and multiple disadvantage in Stoke-on-Trent: The need for safe places in the context of wider health and social inequalities	-EXCLUDE on intervention (service engagement/helpseeking behaviour)	
####	McCormick-Huhn (2019) (ID:87963169)	What if psychology took intersectionality seriously? Changing how psychologists think about participants	-EXCLUDE on population (no housing precarity)	
####	McCoyd (2016) (ID:87850951)	Social work in health settings: Practice in context.	-EXCLUDE on intervention (service engagement/helpseeking behaviour)	
####	McCrady (2021) (ID:87857980)	The role of the family in alcohol use disorder recovery for adults	-EXCLUDE on population (no gender focus; women population <50)	
####	McCree (2021) (ID:87848175)	Sociodemographic Correlates of Self-reported Discrimination in HIV Health Care Settings Among Persons With Diagnosed HIV in the United States, Medical Monitoring Project, 2018-2019.	-EXCLUDE on intervention (service engagement/helpseeking behaviour)	
####	McCullough (2017) (ID:87857481)	The Counseling Experiences of Transgender and Gender Nonconforming Clients	-EXCLUDE on population (no housing precarity)	
####	McDermott (2007) (ID:87850550)	Old soldiers never die : they adapt their military skills and become successful civilians : what factors contribute to the successful transition of army veterans to civilian life and work?	-EXCLUDE on date (2010)	
####	McDONAGH (2019) (ID:87857348)	"Bury don't discuss": the help-seeking behaviour of family members affected by substance-use disorders	-EXCLUDE on population (no housing precarity)	

####	McDowell (2015) (ID:87850515)	How is women's homelessness governed in contemporary society? : a Foucauldian perspective	-EXCLUDE - but review for literature<br>-EXCLUDE on intervention (service engagement/helpseeking behaviour)	
####	McElroy (2018) (ID:87849324)	Medical anthropology in ecological perspective, sixth edition	-EXCLUDE on evidence and form (evidence not in written form or presented as research output)	
####	McFarlane (2012) (ID:87849089)	Testing two global models to prevent violence against women and children: methods and baseline data analysis of a seven-year prospective study.	-EXCLUDE on intervention (service engagement/helpseeking behaviour)	
####	McFarlane (2014) (ID:87851041)	Predicting abused women with children who return to the abuser: Development of a risk assessment tool.	-EXCLUDE on intervention (service engagement/helpseeking behaviour)	
####	McFarlane (2015) (ID:87851028)	Abused women with children who are first-time users of a shelter or applicants for a protection order: Entry data of a 7-year prospective analysis.	-EXCLUDE on intervention (service engagement/helpseeking behaviour)	
####	McGarvey (2013) (ID:87849046)	Decisions to initiate involuntary commitment: the role of intensive community services and other factors.	-EXCLUDE on population (no gender focus; women population <50)	
####	McGeough (2020) (ID:87849330)	Barriers and facilitators perceived by women while homeless and pregnant in accessing antenatal and or postnatal healthcare: A qualitative evidence synthesis	-INCLUDE on title & abstract	-EXCLUDE on intervention (service engagement/helpseeking behaviour)
####	McGill (2018) (ID:87848529)	Reducing challenging behaviour of adults with intellectual disabilities in supported accommodation: A cluster randomized controlled trial of setting-wide positive behaviour support.	-EXCLUDE on intervention (service engagement/helpseeking behaviour)	
####	McGonigle (2018) (ID:87848573)	Assessing Racial Disparities in HCV Infection and Care Outcomes in a Southern Urban Population.	-EXCLUDE on intervention (service engagement/helpseeking behaviour)	
####	McGrath (2023) (ID:87852760)	Social capital and women's narratives of homelessness and multiple exclusion in northern England	-EXCLUDE on intervention (service engagement/helpseeking behaviour)	
####	McGuffey (2018) (ID:87857491)	INTERSECTIONALITY, COGNITION, DISCLOSURE AND BLACK LGBT VIEWS ON CIVIL RIGHTS AND MARRIAGE EQUALITY Is Gay the New Black?	-EXCLUDE on population (no housing precarity)	
####	McGuire (2011) (ID:87849229)	Patient and program predictors of 12-month outcomes for homeless veterans following discharge from time-limited residential treatment.	-EXCLUDE on intervention (service engagement/helpseeking behaviour)	
####	McIlwaine (2023) (ID:87857482)	Building emotional-political communities to address gendered violence against women and girls during COVID-19 in the favelas of Maré, Rio de Janeiro	-EXCLUDE on country (High-Income)	
####	McInnes (2014) (ID:87851074)	Retaining homeless veterans in outpatient care: A pilot study of mobile phone text message appointment reminders.	-EXCLUDE on intervention (service engagement/helpseeking behaviour)	
####	McInnis (2020) (ID:87852865)	Sex After Prostate Cancer in Gay and Bisexual Men: A Review of the Literature	-EXCLUDE on population (no housing precarity)	
####	McKee (2013) (ID:87858072)	The Transnational Adoption Industrial Complex: An Analysis of Nation, Citizenship, and the Korean Diaspora	-EXCLUDE on population (no housing precarity)	
####	McKenzie (2019) (ID:87848422)	Homelessness-'It will crumble men': The views of staff and service users about facilitating the identification and support of people with an intellectual disability in homeless services.	-EXCLUDE on population (women)	
####	McLachlan (2020) (ID:87848295)	Difficulties in Daily Living Experienced by Adolescents, Transition-Aged Youth, and Adults With Fetal Alcohol Spectrum Disorder.	-EXCLUDE on intervention (service engagement/helpseeking behaviour)	
####	McLaren (2021) (ID:87857834)	A sexual and reproductive health rights approach to menstruation	-EXCLUDE on country (High-Income)	
####	McLean (2012) (ID:87849127)	Needle exchange and the geography of survival in the South Bronx.	-EXCLUDE on intervention (service engagement/helpseeking behaviour)	
####	McLemore (2018) (ID:87963030)	Health care experiences of pregnant, birthing and postnatal women of color at risk for preterm birth	-EXCLUDE on population (no housing precarity)	
####	McLeod (2020) (ID:87857508)	Intimate partner violence: Innovations in theory to inform clinical practice, policy, and research	-EXCLUDE on intervention (service engagement/helpseeking behaviour)	
####	McLuhan (2023) (ID:87851867)	Finding help and hope in a peer-led reentry service hub near a detention centre: A process evaluation	-EXCLUDE on population (women)	
####	McMahon (2011) (ID:87849232)	Poverty, hunger, education, and residential status impact survival in HIV.	-EXCLUDE on intervention (service engagement/helpseeking behaviour)	

####	McMANUS (2016) (ID:87857034)	Joining the dots: the combined burden of violence, abuse and poverty in the lives of women	-INCLUDE on title & abstract	-EXCLUDE on intervention (too programme-specific; not generalisable)
####	McMaster (2017) (ID:87848653)	A Qualitative Study of a Maintenance Support Program for Women at Risk of Homelessness: Part 3: Societal Factors.	-EXCLUDE on intervention (service engagement/helpseeking behaviour)	
####	McMorrow (2021) (ID:87851806)	Evidence from a Longitudinal Photovoice and Interview Assessment with Congolese Refugee Women in the Midwestern United States.	-EXCLUDE on intervention (service engagement/helpseeking behaviour)	
####	McNair (2022) (ID:87963093)	Lesbian, gay and bisexual homelessness in Australia: Risk and resilience factors to consider in policy and practice	-EXCLUDE on intervention (service engagement/helpseeking behaviour)	
####	McNEISH (2014) (ID:87857027)	Women and girls at risk: evidence across the life course	-EXCLUDE on population (no housing precarity)	
####	McPhee (2015) (ID:87850981)	Rehab, respite and recovery: The experiences of methadone users in a modified therapeutic community (MTC) in Scotland.	-EXCLUDE on population (women)	
####	McQuistion (2014) (ID:87848988)	Risk factors associated with recurrent homelessness after a first homeless episode.	-EXCLUDE on population (women)	
####	McShay (2017) (ID:87963338)	Engaging students at the intersections through multicultural centers: An application of the culturally engaging campus environment model	-EXCLUDE on evidence and form (evidence not in written form or presented as research output)	
####	Meade (2020) (ID:87963146)	Embracing diverse women veteran narratives: Intersectionality and women veteran's identity	-EXCLUDE on population (no housing precarity)	
####	Meddings (2019) (ID:87857880)	To what extent does Sussex Recovery College reflect its community? An equalities and diversity audit	-EXCLUDE on intervention (service engagement/helpseeking behaviour)	
####	Medina-Perucha (2019) (ID:87963378)	A qualitative study on intersectional stigma and sexual health among women on opioid substitution treatment in England: implications for research, policy and practice	-EXCLUDE on population (no housing precarity)	
####	Medrano (2018) (ID:87848484)	Three-Year Naturalistic Study On Early Use Of Long-Acting Injectable Antipsychotics In First Episode Psychosis.	-EXCLUDE on intervention (service engagement/helpseeking behaviour)	
####	Meehan (2011) (ID:87849195)	Does supported accommodation improve the clinical and social outcomes for people with severe psychiatric disability? The Project 300 experience.	-EXCLUDE on intervention (service engagement/helpseeking behaviour)	
####	Meek (2020) (ID:87850535)	Participatory book art : establishing connections with dialogue, representation and value	-EXCLUDE on intervention (service engagement/helpseeking behaviour)	
####	Mehtani (2021) (ID:87848214)	COVID-19: A catalyst for change in telehealth service delivery for opioid use disorder management.	-EXCLUDE on population (no housing precarity)	
####	Meichenbaum (2018) (ID:87850795)	Ways to treat victims of human trafficking: Core therapeutic tasks.	-INCLUDE on title & abstract	-EXCLUDE on target group (not focused on women's behaviour/outcomes for women)
####	Meinbresse (2014) (ID:87848947)	Exploring the experiences of violence among individuals who are homeless using a consumer-led approach.	-EXCLUDE on intervention (service engagement/helpseeking behaviour)	
####	Meisler (1997) (ID:87853710)	Impact of assertive community treatment on homeless persons with co-occurring severe psychiatric and substance use disorders	-EXCLUDE on date (2010)	
####	Mejia-Lancheros (2020) (ID:87848312)	Dental problems and chronic diseases in mentally ill homeless adults: a cross-sectional study.	-EXCLUDE on intervention (service engagement/helpseeking behaviour)	
####	Mejia-Lancheros (2020) (ID:87848319)	Trajectories and mental health-related predictors of perceived discrimination and stigma among homeless adults with mental illness.	-EXCLUDE on intervention (service engagement/helpseeking behaviour)	
####	MELZAK (2019) (ID:87857085)	Acting in the best interests of unaccompanied asylum seeking children	-EXCLUDE on population (people aged under 18 years)	
####	MEMON (2018) (ID:87851420)	Walking the talk on diversity: what is holding the charity sector back from putting words into action	-EXCLUDE on intervention (service engagement/helpseeking behaviour)	
####	Mengo (2021) (ID:87850635)	Intimate partner violence and women's mental health: The mediating role of coping strategies among women seeking help from the police.	-INCLUDE on title & abstract	-EXCLUDE on intervention (service engagement/helpseeking behaviour)
####	Menke (2014) (ID:87853234)	Midwives' perceptions of organisational structures and processes influencing their ability to provide caseload care to socially disadvantaged and vulnerable women	-EXCLUDE on intervention (service engagement/helpseeking behaviour)	
####	Menon (2023) (ID:88019149)	Characterization of an extreme phenotype of schizophrenia among women with homelessness	-EXCLUDE on country (High-Income)	
####	Mercier (2011) (ID:87849216)	Intellectual disability and homelessness.	-EXCLUDE on intervention (service engagement/helpseeking behaviour)	

####	Mericle (2017) (ID:87848695)	Quality of life and the complex needs of recovery home residents.	-EXCLUDE on population (women)	
####	Merrick (2012) (ID:87851212)	Child health and human development yearbook, 2010.	-EXCLUDE on population (people aged under 18 years)	
####	MERRY (2012) (ID:87857302)	The effectiveness of SPARX, a computerised self help intervention for adolescents seeking help for depression: randomised controlled non-inferiority trial	-EXCLUDE on population (people aged under 18 years)	
####	Merry (2019) (ID:87858088)	Migrant families with children in Montreal, Canada and transnational family support: a protocol for a focused ethnography	-EXCLUDE on intervention (service engagement/helpseeking behaviour)	
####	MESCHEDE (2010) (ID:87856987)	Accessing housing: exploring the impact of medical and substance abuse services on housing attainment for chronically homeless street dwellers	-EXCLUDE on intervention (service engagement/helpseeking behaviour)	
####	Messing (2021) (ID:87963213)	'Not bullet proof': The complex choice not to seek a civil protection order for intimate partner violence	-INCLUDE on title & abstract	-EXCLUDE (IPV/DVA but little discussion on housing)
####	Messinger (2020) (ID:87850673)	Transgender intimate partner violence: A comprehensive introduction.	-EXCLUDE on intervention (service engagement/helpseeking behaviour)	
####	Messinger (2022) (ID:87851879)	Intimate Partner Violence Help-Seeking in the U.S. Transgender Survey	-EXCLUDE on intervention (service engagement/helpseeking behaviour)	
####	Metcalfe (2014) (ID:87848940)	An academic-community partnership to address the flu vaccination rates of the homeless.	-EXCLUDE on population (women)	
####	Meyer (2014) (ID:87848966)	Gender disparities in HIV treatment outcomes following release from jail: results from a multicenter study.	-EXCLUDE on intervention (service engagement/helpseeking behaviour)	
####	Meyer (2019) (ID:87963097)	Research on women with substance use disorders: Reviewing progress and developing a research and implementation roadmap	-EXCLUDE on population (no housing precarity)	
####	Meyer (2022) (ID:87857905)	Combining Group-based Interventions for Intimate Partner Violence Perpetrators With Comorbid Substance Use: An Australian Study of Cross-sector Practitioner Views	-EXCLUDE on intervention (service engagement/helpseeking behaviour)	
####	Meyer (2022) (ID:87963281)	Mothering in the context of violence: Indigenous and non-Indigenous mothers' experiences in regional settings in Australia	-INCLUDE on title & abstract	-EXCLUDE on intervention (service engagement/helpseeking behaviour)
####	Mezzina (2022) (ID:87963284)	Social vulnerability and mental health inequalities in the "Syndemic": Call for action	-EXCLUDE on intervention (service engagement/helpseeking behaviour)	
####	Mhende (2021) (ID:87853039)	Mobile Delivery of Mindfulness-Based Smoking Cessation Treatment Among Low-Income Adults During the COVID-19 Pandemic: Pilot Randomized Controlled Trial	-EXCLUDE on intervention (service engagement/helpseeking behaviour)	
####	MIDDLETON (2018) (ID:87856966)	Youth experiences survey (YES): exploring the scope and complexity of sex trafficking in a sample of youth experiencing homelessness	-EXCLUDE on intervention (service engagement/helpseeking behaviour)	
####	Miguel (2018) (ID:87848551)	Sociodemographic Characteristics, Patterns of Crack Use, Concomitant Substance Use Disorders, and Psychiatric Symptomatology in Treatment-Seeking Crack-Dependent Individuals in Brazil.	-EXCLUDE on country (High-Income)	
####	Miguel (2022) (ID:88019126)	What factors influence engagement with primary mental health care services? A qualitative study of service user perspectives in rural communities of Mexico	-EXCLUDE on country (High-Income)	
####	Mihelicova (2018) (ID:87857667)	Trauma-Informed Care for Individuals with Serious Mental Illness: An Avenue for Community Psychology's Involvement in Community Mental Health	-EXCLUDE on population (no housing precarity)	
####	Milaney (2018) (ID:87851927)	Falling Through the Cracks: How the Community-Based Approach Has Failed Calgary's Chronically Homeless	-EXCLUDE on intervention (service engagement/helpseeking behaviour)	
####	Milaney (2020) (ID:87848358)	A Portrait of Late Life Homelessness in Calgary, Alberta.	-EXCLUDE on population (women)	
####	Milani (2018) (ID:87963186)	'Beyond Cultural Sensitivity': Service Providers' Perspectives on Muslim Women Experiences of Intimate Partner Violence	-INCLUDE on title & abstract	-EXCLUDE (IPV/DVA but little discussion on housing)
####	MILES (2018) (ID:87857113)	Nowhere to turn, 2018: findings from the second year of the No Woman Turned away project	-INCLUDE on title & abstract	-EXCLUDE on intervention (intersectionality)
####	Millar (2022) (ID:87857620)	Adapted remote cognitive behavioural therapy for comfort eating with a woman with intellectual disabilities: Case report	-EXCLUDE on population (no housing precarity)	
####	Millen (2020) (ID:87848334)	Pilot Study Evaluating Critical Time Intervention for Individuals With Hoarding Disorder at Risk for Eviction.	-EXCLUDE on intervention (service engagement/helpseeking behaviour)	

####	Miller (2017) (ID:87851790)	Transition Readiness, Perceived Health, and Health Services Utilization in Transitional Age Foster Youth Compared to Controls.	-EXCLUDE on population (no gender focus; women population <50)	
####	MILLER (2019) (ID:87857292)	Virtual support groups among adoptive parents: Ideal for information seeking?	-EXCLUDE on population (no housing precarity)	
####	Miller (2020) (ID:87857439)	Intersectionality IN THE LGBTQIA Community	-EXCLUDE on population (no housing precarity)	
####	Miller (2020) (ID:87857582)	Intersectionality IN THE LGBTQIA+ Community: Defining the issues and understanding the history of systems of discrimination	-EXCLUDE on population (no housing precarity)	
####	Miller (2022) (ID:87850592)	An evaluation of a summer camp designed to meet the needs of homeless adolescents: Findings on the impact of camp participation.	-EXCLUDE on population (people aged under 18 years)	
####	Miller (2022) (ID:87858106)	The Physical Hospital Environment and Its Effects on Palliative Patients and Their Families: A Qualitative Meta-Synthesis	-EXCLUDE on population (no housing precarity)	
####	Miller-Archie (2019) (ID:87848435)	Impact of supportive housing on substance use-related health care utilization among homeless persons who are active substance users.	-EXCLUDE on intervention (service engagement/helpseeking behaviour)	
####	Mills (2015) (ID:87851003)	Engaging the citizenship of the homeless-A qualitative study of specialist primary care providers.	-EXCLUDE on intervention (service engagement/helpseeking behaviour)	
####	Mills-Koonce (2018) (ID:87857772)	The Significance of Parenting and Parent–Child Relationships for Sexual and Gender Minority Adolescents	-EXCLUDE on population (people aged under 18 years)	
####	MIND (2010) (ID:87857187)	Welsh youth consultation on suicide and help seeking behaviours	-EXCLUDE on population (no housing precarity)	
####	Minhas (2022) (ID:87851965)	Maharashtra to provide Rs 468Cr for WCD schemes from DPDC	-EXCLUDE on evidence and form (evidence not in written form or presented as research output)	
####	Minoyan (2022) (ID:87848154)	Self-reported impacts of the COVID-19 pandemic among people who use drugs: a rapid assessment study in Montreal, Canada.	-EXCLUDE on population (women)	
####	Mirzazadeh (2020) (ID:87848275)	Barriers and motivators to participation and retention in HIV/HCV cohort studies among people who inject drugs: a community consultation in Iran.	-EXCLUDE on country (High-Income)	
####	MITCHELL (2010) (ID:87857120)	Prisoner perspectives on mental health problems and help-seeking	-EXCLUDE on population (women)	
####	Mitchell (2016) (ID:87850930)	Gender differences in psychosocial complexity for a cohort of adolescents attending youth-specific substance abuse services.	-EXCLUDE on population (no gender focus; women population <50)<br>-EXCLUDE - but review for literature	
####	Mitchell (2017) (ID:87963370)	Teaching community on and off campus: An intersectional approach to community engagement	-EXCLUDE on population (no housing precarity)	
####	Mitchell (2019) (ID:87857975)	Content Analysis of PsychArticles Database: Representation of People Previously Incarcerated	-EXCLUDE on intervention (service engagement/helpseeking behaviour)	
####	Mitchell (2022) (ID:87848146)	Health service use and predictors of high health service use among adults experiencing homelessness: a retrospective cohort study.	-EXCLUDE on intervention (service engagement/helpseeking behaviour)	
####	Mitchell (2023) (ID:87857477)	Figuring out how to participate in the system: Using reflexive feminist autoethnography to explore intersectional experiences in the professional and political spheres of academia	-EXCLUDE on evidence and form (evidence not in written form or presented as research output)	
####	Mitruka (2017) (ID:87848676)	Disparities in Tuberculosis Treatment Completion by Incarceration Status, U.S., 1999-2011.	-EXCLUDE on intervention (service engagement/helpseeking behaviour)	
####	Mitsdarffer (2023) (ID:88019125)	LGBTQ+ Youth Homelessness in Delaware: Building a Case for Targeted Surveillance and Assessment of LGBTQ+ Youth Needs and Experiences	-INCLUDE on title & abstract	-EXCLUDE on intervention (service engagement/helpseeking behaviour)
####	Miyaji (2022) (ID:87848147)	Social Capital and Post-traumatic Stress Disorder among Heavy Rainfall and Flood Victims in Japan.	-EXCLUDE on intervention (service engagement/helpseeking behaviour)	
####	Mizock (2021) (ID:87850654)	Women with serious mental illness: Gender-sensitive and recovery-oriented care.	-EXCLUDE - but review for literature<br>-EXCLUDE on population (no housing precarity)	
####	Mizuno (2015) (ID:87851037)	Syndemic vulnerability, sexual and injection risk behaviors, and HIV continuum of care outcomes in HIV-positive injection drug users.	-EXCLUDE on population (no housing precarity)	

####	Moczygemba (2013) (ID:87853062)	A qualitative analysis of perceptions and barriers to therapeutic lifestyle changes among homeless hypertensive patients	-EXCLUDE on intervention (service engagement/helpseeking behaviour)	
####	Moczygemba (2014) (ID:87848909)	Utilization of behavioral health outpatient therapy and psychiatry services among homeless people recently hospitalized for a psychiatric condition.	-EXCLUDE on population (women)	
####	Moczygemba (2014) (ID:87851043)	Adherence to behavioral therapy and psychiatry visits in a safety-net setting in Virginia, USA.	-EXCLUDE on population (no gender focus; women population <50)	
####	Moczygemba (2017) (ID:87848685)	Homeless patients' perceptions about using cell phones to manage medications and attend appointments.	-EXCLUDE on intervention (service engagement/helpseeking behaviour)	
####	Mody (2021) (ID:87853368)	Gender-Based Differences in Outcomes Among Resuscitated Patients With Out-of-Hospital Cardiac Arrest	-EXCLUDE on population (no housing precarity)	
####	MOJTABAI (2008) (ID:87857145)	Social comparison of distress and mental health help-seeking in the US general population	-EXCLUDE on date (2010)	
####	Molewyk (2020) (ID:87848345)	Factors impacting attrition of vulnerable women from a longitudinal mental health intervention study.	-EXCLUDE on intervention (service engagement/helpseeking behaviour)	
####	Molina (2022) (ID:87848145)	Analysis of Emergency Department Encounters Among High Users of Health Care and Social Service Systems Before and During the COVID-19 Pandemic.	-EXCLUDE on intervention (service engagement/helpseeking behaviour)	
####	Molinari (2013) (ID:87849022)	Perceptions of homelessness in older homeless veterans, VA homeless program staff liaisons, and housing intervention providers.	-EXCLUDE on intervention (service engagement/helpseeking behaviour)	
####	MOLOCZIJ (2008) (ID:87857151)	Help-seeking at the time of stroke: stroke survivors' perspectives on their decisions	-EXCLUDE on date (2010)	
####	Momen (2020) (ID:87963198)	Why Don't You Just Leave?	-INCLUDE on title & abstract	-EXCLUDE (IPV/DVA but little discussion on housing)
####	Momen (2022) (ID:87858114)	Living on the Edge: Trans Exclusion, Survival, and Resistance	-EXCLUDE on intervention (service engagement/helpseeking behaviour)<br>-EXCLUDE - but review for literature	
####	Monaghan (2012) (ID:87858013)	Embodied Heterosexual Masculinities, Part 1: Confluent Intimacies, Emotions and Health	-EXCLUDE on population (women)	
####	Mondal (2013) (ID:87851951)	ROLE OF SELF HELP GROUPS (SHGs) OF TRIBAL INHABITED RURAL AREA ON FOOD SECURITY AT HOUSEHOLD LEVEL	-EXCLUDE on population (no housing precarity)	
####	Monick (2022) (ID:87963133)	Eliminating disparities in youth substance use among Native Hawaiian, Micronesian, and sex and gender minorities: A qualitative needs assessment from interviews with public service leaders.	-EXCLUDE on population (no housing precarity)	
####	Monro (2010) (ID:87963321)	Sexuality, space and intersectionality: the case of lesbian, gay and bisexual equalities initiatives in UK local government	-EXCLUDE on population (no housing precarity)	
####	Montalvo-Liendo (2016) (ID:87850917)	Ethnoracial variation in depression symptoms.	-EXCLUDE on population (no housing precarity)	
####	Montano (2023) (ID:87857503)	Trans death at Rikers Island: sites of (in)visibility and reframing mass incarceration	-EXCLUDE on intervention (service engagement/helpseeking behaviour)	
####	Montesanti (2015) (ID:87963217)	Mapping the role of structural and interpersonal violence in the lives of women: implications for public health interventions and policy	-EXCLUDE on intervention (service engagement/helpseeking behaviour)<br>-EXCLUDE - but review for literature	
####	Montgomery (2011) (ID:87849220)	A comparison of individual and social vulnerabilities, health, and quality of life among Canadian women with mental diagnoses and young children.	-EXCLUDE on intervention (service engagement/helpseeking behaviour)	
####	Montgomery (2014) (ID:87848910)	Development and validation of an instrument to assess imminent risk of homelessness among veterans.	-EXCLUDE on intervention (service engagement/helpseeking behaviour)	
####	Montgomery (2014) (ID:87851077)	Services utilization among recently homeless veterans: A gender-based comparison.	-EXCLUDE on population (no gender focus; women population <50)	
####	Montgomery (2015) (ID:87848874)	Demographic characteristics associated with homelessness and risk among female and male veterans accessing VHA outpatient care.	-EXCLUDE on intervention (service engagement/helpseeking behaviour)	
####	Montgomery (2019) (ID:87848432)	Priorities for Supportive Housing Services: Perspectives of People With Mental Illness in Northeastern Ontario.	-EXCLUDE on intervention (service engagement/helpseeking behaviour)	
####	Montgomery (2020) (ID:87848262)	Correlates of Transitions Into Housing Instability Among Veterans Accessing Veterans Health Administration Health Care.	-EXCLUDE on intervention (service engagement/helpseeking behaviour)	

####	Montgomery (2020) (ID:87853377)	Demographic Correlates of Veterans' Adverse Social Determinants of Health	-EXCLUDE on intervention (service engagement/helpseeking behaviour)	
####	MONTGOMERY (2020) (ID:87857064)	Patients' perspectives on elements of stable housing and threats to housing stability	-EXCLUDE on population (women)	
####	MOON (2010) (ID:87857392)	Bridging pastoral counseling and social work practice: an exploratory study of pastors' perceptions of and responses to intimate partner violence	-EXCLUDE on intervention (service engagement/helpseeking behaviour)	
####	Moore (2010) (ID:87849267)	Male reproductive control of women who have experienced intimate partner violence in the United States.	-EXCLUDE on intervention (service engagement/helpseeking behaviour)	
####	Moore (2016) (ID:87848736)	Factors Affecting Emergency Department Use by a Chronically Homeless Population.	-EXCLUDE on intervention (service engagement/helpseeking behaviour)	
####	Moore (2017) (ID:87857840)	Multiple Minority Identities and Mental Health Service Use: A Mixed-Methods Study of Sexual and Gender Minority Young Adults of Color	-EXCLUDE on population (no housing precarity)	
####	MOORE (2018) (ID:87857285)	Postmigration living difficulties, help-seeking and community resilience in the initial stages of migration: coproducing community practice with recent Irish migrants to London	-EXCLUDE on population (women)	
####	Moore (2020) (ID:87857650)	Identity negotiation processes among Black and Latinx sexual minority young adult mental health service users	-EXCLUDE on population (no housing precarity)	
####	Moore (2020) (ID:87963125)	Risk factors for self-stigma among incarcerated women with alcohol use disorder.	-INCLUDE on title & abstract	-EXCLUDE on target group (no housing precarity)
####	Moore (2021) (ID:87963354)	A mixed-methods study of social identities in mental health care among LGBTQ young adults of color.	-EXCLUDE on population (no housing precarity)	
####	Moorhead (2021) (ID:87857552)	'This is me': Expressions of intersecting identity in an LGBTQ+ ethnic studies course	-EXCLUDE on intervention (service engagement/helpseeking behaviour)	
####	Moraes (2021) (ID:87857926)	Understanding Lived Experiences of Food Insecurity through a Paraliminality Lens	-EXCLUDE on population (no housing precarity)	
####	MORAN (2007) (ID:87857081)	Attachment style, ethnicity and help-seeking attitudes among adolescent pupils	-EXCLUDE on date (2010)	
####	Moran (2022) (ID:87963232)	"It helped me open my eyes": Incorporating lived experience perspectives in social work education	-EXCLUDE on population (no housing precarity)	
####	Morewitz (2016) (ID:87850918)	Runaway and homeless youth: New research and clinical perspectives.	-EXCLUDE on intervention (service engagement/helpseeking behaviour)	
####	MORGAN (2014) (ID:87857305)	Asking men about domestic violence and abuse in a family medicine context: help seeking and views on the general practitioner	-EXCLUDE on population (women)	
####	MORGAN (2016) (ID:87857240)	The role of gender, sexuality and context upon help-seeking for intimate partner violence: a synthesis of data across five studies	-INCLUDE on title & abstract	-EXCLUDE (IPV/DVA but little discussion on housing)
####	Morgan (2021) (ID:87857860)	Like a Chameleon: Resilience among Self-Identified Latinx Mixed Adults/Como un camaleón: la resiliencia entre los adultos mixtos autoidentificados como latinos	-EXCLUDE on population (no housing precarity)	
####	Morlino (2011) (ID:87851340)	Use of psychiatric inpatient services by heavy users: Findings from a national survey in Italy.	-EXCLUDE on population (no housing precarity)	
####	Morrell (1999) (ID:87850534)	Housing and the women's movement, 1860-1914	-EXCLUDE on date (2010)	
####	Morris (2016) (ID:87852007)	Monitor staffJennifer Payson gave birth to her daughter in Manchester's Valley Street Jail. The 31-year-old mother of three has used painkillers and heroin on and off since she was a teen, but landed behind bars in 2014 on a drug-related charge that was later dismissed.... [Derived Headline]	-EXCLUDE on evidence and form (evidence not in written form or presented as research output)	
####	Morris (2021) (ID:87857998)	Working with critical reflective pedagogies at a moment of post-truth populist authoritarianism	-EXCLUDE on intervention (service engagement/helpseeking behaviour)	
####	Morris-Dias (2022) (ID:87857621)	Experiences and Perceptions of Help-Seeking for Psychological Distress among African American Women	-EXCLUDE on population (no housing precarity)<br>-EXCLUDE - but review for literature	
####	MORRISON (2006) (ID:87857286)	Factors influencing help-seeking from informal networks among African American victims of intimate partner violence	-EXCLUDE on date (2010)	
####	Morrison (2008) (ID:87850518)	Homelessness and deprivation in Glasgow : a 5-year retrospective cohort study of hospitalisations and deaths	-EXCLUDE on date (2010)	
####	Morrison (2016) (ID:87853311)	Effect of gender on outcome of out of hospital cardiac arrest in the Resuscitation Outcomes Consortium	-EXCLUDE on intervention (service engagement/helpseeking behaviour)	

####	Morrison (2019) (ID:87857803)	CONTESTED MEANINGS AND LIVED EXPERIENCES OF TWO-SPIRITNESS: A SYSTEMATIC REVIEW OF THE CANADIAN RESEARCH LITERATURE	-EXCLUDE on population (no housing precarity)	
####	Morrison (2022) (ID:87858016)	Measuring the Effectiveness of LGBTQ Training Workshops for Educators	-EXCLUDE on intervention (service engagement/helpseeking behaviour)	
####	Morton (2010) (ID:87849263)	Volunteerism among homeless persons with developmental disabilities.	-EXCLUDE on intervention (service engagement/helpseeking behaviour)	
####	MOSKOWITZ (2006) (ID:87857055)	Students in the community: an interprofessional student-run free clinic	-EXCLUDE on date (2010)	
####	MOSS (2015) (ID:87856955)	Women rough sleepers in Europe: homelessness and victims of domestic abuse	-INCLUDE on title & abstract	-EXCLUDE on evidence and form (evidence not in written form or presented as research output)
####	Moss (2020) (ID:87857841)	Muslim, Jewish, and Christian Women Living with Resilience Amidst Microaggressions and Macroaggressions in King County	-EXCLUDE on population (no housing precarity)	
####	**Mostowska (2020) (ID:87963367)**	**An ambiguous hierarchy of inequalities. The political intersectionality of older women's homelessness in Poland**	**-INCLUDE on title & abstract**	**-INCLUDE on full study**
####	Motta-Ochoa (2017) (ID:87848617)	"I love having benzos after my coke shot": The use of psychotropic medication among cocaine users in downtown Montreal.	-EXCLUDE on population (women)	
####	Moulin (2018) (ID:87848491)	Substance Use, Homelessness, Mental Illness and Medicaid Coverage: A Set-up for High Emergency Department Utilization.	-EXCLUDE on intervention (service engagement/helpseeking behaviour)	
####	Mountz (2018) (ID:87857579)	'Because We're Fighting to Be Ourselves: Voices from Former Foster Youth who are Transgender and Gender Expansive	-EXCLUDE on population (no housing precarity)	
####	Mountz (2019) (ID:87857936)	Speaking Back to the System: Recommendations for Practice and Policy from the Perspectives of Youth Formerly in Foster Care who are LGBTQ	-INCLUDE on title & abstract	-EXCLUDE on intervention (service engagement/helpseeking behaviour)
####	Mountz (2020) (ID:87963242)	Exploring the families of origin of LGBTQ former foster youth and their trajectories throughout care	-EXCLUDE on population (no housing precarity)	
####	Moves to independent... (CRANE) (ID:87857025)	Moves to independent living: single homeless people's experiences and outcomes of resettlement	-EXCLUDE on population (women)	
####	Mowatt (2013) (ID:87857657)	Black/Female/Body Hypervisibility and Invisibility: A Black Feminist Augmentation of Feminist Leisure Research	-EXCLUDE on population (no housing precarity)	
####	MOXLEY (2015) (ID:87857021)	The relevance of four narrative themes for understanding vulnerability among homeless older African-American women	-INCLUDE on title & abstract	-EXCLUDE on intervention (service engagement/helpseeking behaviour)
####	Moya (2017) (ID:87853083)	Adults Experiencing Homelessness in the US-Mexico Border Region: A Photovoice Project	-EXCLUDE on intervention (service engagement/helpseeking behaviour)	
####	Moya (2021) (ID:87963214)	Homelessness and Macro Interventions	-EXCLUDE on intervention (service engagement/helpseeking behaviour)	
####	MUELLER (2019) (ID:87857196)	Developing and testing a web-based intervention to encourage early help-seeking in people with symptoms associated with lung cancer	-EXCLUDE on population (no housing precarity)	
####	Mueller (2023) (ID:87851871)	Sex Trading, Exiting, and Interactions With Police: A Mixed Methods Study of Women Engaged in Street Level Sex Trading	-EXCLUDE on intervention (service engagement/helpseeking behaviour)	
####	Mueller (2023) (ID:87963115)	Sex Trading, Exiting, and Interactions With Police: A Mixed Methods Study of Women Engaged in Street Level Sex Trading	-EXCLUDE Duplicate	
####	Mugambwa (2023) (ID:87850567)	Mental health of people with limited access to health services: A retrospective study of patients attending a humanitarian clinic network in Germany in 2021.	-EXCLUDE on intervention (service engagement/helpseeking behaviour)	
####	Muhajarine (2022) (ID:87857628)	Equity Analysis of Repeated Cross-Sectional Survey Data on Mental Health Outcomes in Saskatchewan, Canada during COVID-19 Pandemic	-EXCLUDE on intervention (service engagement/helpseeking behaviour)	
####	Mukherjee (2022) (ID:87850598)	Barriers to help-seeking for South Asian immigrant survivors of intimate partner violence (IPV): An interpretative phenomenological study.	-INCLUDE on title & abstract	-EXCLUDE (IPV/DVA but little discussion on housing)
####	Mukherjee (2022) (ID:87963014)	Barriers to Help-Seeking for South Asian Immigrant Survivors of Intimate Partner Violence (IPV): An Interpretative Phenomenological Study	-EXCLUDE Duplicate	
####	Mukhopadhyay (2019) (ID:87857487)	Found and Lost in Translation: Exploring the Legal Protection of Women from the Domestic Violence Act 2005 Through the Social Public Space of Kolkata	-EXCLUDE on country (High-Income)	

####	Mulder (2014) (ID:87848989)	The availability and quality across Europe of outpatient care for difficult-to-engage patients with severe mental illness: a survey among experts.	-EXCLUDE on intervention (service engagement/helpseeking behaviour)	
####	Muller (2017) (ID:87853349)	Text message reminders increased colorectal cancer screening in a randomized trial with Alaska Native and American Indian people	-EXCLUDE on population (no housing precarity)	
####	Munawar (2018) (ID:87851551)	BARRIERS TO TRANSGENDER HEALTH CARE IN PAKISTAN	-EXCLUDE on country (High-Income)	
####	Murakami (2017) (ID:87848619)	Association between housing type and gamma-GTP increase after the Great East Japan Earthquake.	-EXCLUDE on intervention (service engagement/helpseeking behaviour)	
####	Murdoch (2011) (ID:87849169)	Long-term outcomes of disability benefits in US veterans with posttraumatic stress disorder.	-EXCLUDE on intervention (service engagement/helpseeking behaviour)	
####	Murdoch (2020) (ID:87853225)	Serial tests of T-cell function predict long-term survival in an elderly cohort from a Scottish general practice	-EXCLUDE on intervention (service engagement/helpseeking behaviour)	
####	Murfree (2018) (ID:87857913)	Faith and Feminist Activism in the Midwest: An Ethnographic Examination	-EXCLUDE on population (no housing precarity)	
####	Mũrĩithi (2020) (ID:87857548)	Fragments Towards an Impossible (Domestic) Genre of the Human in Kenyan Crime Fiction	-EXCLUDE on country (High-Income)	
####	Murphy (2015) (ID:87848856)	Improving cardiovascular health of underserved populations in the community with Life's Simple 7.	-EXCLUDE on intervention (service engagement/helpseeking behaviour)	
####	Murphy (2018) (ID:87858018)	Recognizing a fuller, more expansive God in unassuming places and people	-EXCLUDE on population (no housing precarity)	
####	MURRAY (2011) (ID:87856920)	Violence against homeless women: safety and social policy	-INCLUDE on title & abstract	-EXCLUDE on intervention (intersectionality)
####	Murray (2023) (ID:87857885)	The unexceptional im/mobilities of gender-based violence in the Covid-19 pandemic	-EXCLUDE on intervention (service engagement/helpseeking behaviour)	
####	Murray (2023) (ID:87857918)	"It's called homophobia baby" exploring LGBTQ + substance use and treatment experiences in the UK	-EXCLUDE on population (no housing precarity)	
####	Murshid (2018) (ID:87853112)	Microfinance Participation and Women's Decision-Making Power in the Household in Bangladesh	-EXCLUDE on country (High-Income)	
####	Murshid (2018) (ID:87963150)	A trauma-informed analysis of the violence against women act's provisions for undocumented immigrant women	-EXCLUDE on intervention (service engagement/helpseeking behaviour)	
####	Murugan (2017) (ID:87963329)	Intimate partner violence among South Asian women in the United States: Prevalence and help-seeking behaviors	-INCLUDE on title & abstract	-EXCLUDE (IPV/DVA but little discussion on housing)
####	Musielak (2018) (ID:87857762)	Exploring Dimensions of Vulnerability in Victims of Domestic Homicide	-EXCLUDE on intervention (service engagement/helpseeking behaviour)	
####	Muthulingam (2013) (ID:87849055)	Disparities in engagement in care and viral suppression among persons with HIV.	-EXCLUDE on intervention (service engagement/helpseeking behaviour)	
####	Mutter (2019) (ID:87848454)	Factors associated with completion of alcohol detoxification in residential settings.	-EXCLUDE on population (women)	
####	Muzanenhamo (2023) (ID:87857443)	A Critique of Vanishing Voice in Noncooperative Spaces: The Perspective of an Aspirant Black Female Intellectual Activist	-EXCLUDE on population (no housing precarity)	
####	Mwachofi (2012) (ID:87851214)	The covariates of mental health with special reference to household gender-structure.	-EXCLUDE on population (no housing precarity)	
####	Myhrvold (2017) (ID:87848686)	The mental healthcare needs of undocumented migrants: an exploratory analysis of psychological distress and living conditions among undocumented migrants in Norway.	-EXCLUDE on intervention (service engagement/helpseeking behaviour)	
####	Myhrvold (2019) (ID:87848463)	Undocumented migrants' life situations: An exploratory analysis of quality of life and living conditions in a sample of undocumented migrants living in Norway.	-EXCLUDE Duplicate	
####	MYRIE (2013) (ID:87857264)	'Should I really be here?' Exploring the relationship between black men's conceptions of well-being, subject positions and help-seeking behaviour	-EXCLUDE on population (women)	
####	Naeem (2019) (ID:87850741)	Cultural adaptations of CBT: A summary and discussion of the Special Issue on Cultural Adaptation of CBT.	-EXCLUDE on intervention (service engagement/helpseeking behaviour)	
####	Naganathan (2014) (ID:87857600)	The core features and perceived value of family support for ethnoracial homeless individuals with mental illness: Findings from the At Home/Chez Soi Project	-EXCLUDE on intervention (service engagement/helpseeking behaviour)	
####	Nagy (2021) (ID:87857567)	Castrating Superman: Rachel Pollack's Transgender Mutant Cyborg Superhero in Doom Patrol	-EXCLUDE on evidence and form (evidence not in written form or presented as research output)	
####	NAKONEZNY (2005) (ID:87856949)	Health services utilization between older and younger homeless adults	-EXCLUDE on date (2010)	

####	Namakhoma (2012) (ID:87851504)	Equity in antiretroviral therapy access : an assessment of patients' experiences in Lilongwe District, Malawi	-EXCLUDE on country (High-Income)	
####	Namuggala (2023) (ID:87851964)	Social and gender norms informing, voicing, and reporting against sexual harassment among domestic workers in Kampala's informal economy	-EXCLUDE on country (High-Income)	
####	Napoles (2019) (ID:87848417)	HCV treatment barriers among HIV/HCV co-infected patients in the US: a qualitative study to understand low uptake among marginalized populations in the DAA era.	-EXCLUDE on population (women)	
####	Naqashbandi (2023) (ID:87850552)	Gender equality in selected novels of Jane Austen : coping with anxiety to redefine home and gender roles	-EXCLUDE on intervention (service engagement/helpseeking behaviour)	
####	Naranbhai (2011) (ID:87849215)	Interventions to modify sexual risk behaviours for preventing HIV in homeless youth.	-EXCLUDE on intervention (service engagement/helpseeking behaviour)	
####	Narasimhan (2020) (ID:87963245)	Homelessness and women living with mental health issues: Lessons from the Banyan's experience in Chennai, Tamil Nadu	-EXCLUDE on country (High-Income)	
####	Narayan (2015) (ID:87853437)	Personal, dyadic, and contextual resilience in parents experiencing homelessness	-EXCLUDE on intervention (service engagement/helpseeking behaviour)	
####	Narayan (2018) (ID:87857555)	Phoolan Devi: Gendered Subaltern; Caste Warrior	-EXCLUDE on country (High-Income)	
####	Nardi (2021) (ID:87857814)	Mindful Women: A Culturally Adapted Mindfulness Program for Black Women	-EXCLUDE on population (no housing precarity)	
####	Narendorf (2016) (ID:87850925)	Parenting and homeless: Profiles of young adult mothers and fathers in unstable housing situations.	-EXCLUDE on population (no gender focus; women population <50)	
####	Narendorf (2017) (ID:87848688)	Symptoms, circumstances, and service systems: Pathways to psychiatric crisis service use among uninsured young adults.	-EXCLUDE on population (no housing precarity)	
####	Narendorf (2023) (ID:87852717)	Self-Identification of Mental Health Problems Among Young Adults Experiencing Homelessness	-EXCLUDE on intervention (service engagement/helpseeking behaviour)	
####	Nash (2022) (ID:87850585)	Management of alcohol and other drug issues in Special Health Accommodation during the COVID-19 Delta variant outbreak in Sydney, 2021.	-EXCLUDE on intervention (service engagement/helpseeking behaviour)	
####	Nash (2023) (ID:87850563)	Guided exploration of military servicewomen's healthcare experiences and related outcomes.	-EXCLUDE on population (no housing precarity)	
####	Nasir (2022) (ID:87857752)	Examining Social Relationships among Older Muslim Immigrants Living in Canada: A Narrative Inquiry	-EXCLUDE on population (no housing precarity)	
####	Nasraddin (2017) (ID:87963345)	Am I not a Woman and a Sister and an Immigrant?: Approaching Intimate Partner Violence in Black Immigrant Communities Within an Intersectional Framework	-INCLUDE on title & abstract	-EXCLUDE (IPV/DVA but little discussion on housing)
####	Nasrullah (2016) (ID:87848727)	The association of recent incarceration and health outcomes among HIV-infected adults receiving care in the United States.	-EXCLUDE on intervention (service engagement/helpseeking behaviour)	
####	Nasser (2021) (ID:87851521)	Oral rehydration salts therapy use among children under five years of age with diarrhea in Ethiopia	-EXCLUDE on country (High-Income)	
####	Nath (2016) (ID:87850916)	The impact of drop-in centers on the health of street children in New Delhi, India: A cross-sectional study.	-EXCLUDE on country (High-Income)	
####	Nathan (2013) (ID:87851671)	Making a Case for the Socioeconomic Determinacy of Survival in Osteosarcoma	-EXCLUDE on intervention (service engagement/helpseeking behaviour)	
####	National (2016) (ID:87851425)	Housing and the health of young children: policy and evidence briefing for the VCSE sector	-EXCLUDE on population (people aged under 18 years)	
####	NATIONAL (2017) (ID:87856973)	Homelessness and health for disadvantaged groups	-INCLUDE on title & abstract	-EXCLUDE on evidence and form (evidence not in written form or presented as research output)
####	National (2022) (ID:88019161)	Learning from organisations on supporting young women - Practice briefing for homelessness services	-EXCLUDE on evidence and form (evidence not in written form or presented as research output)<br>-EXCLUDE - but review for literature	
####	Nau (2023) (ID:87853407)	Assessment of Disruptive Life Events for Individuals Diagnosed With Schizophrenia or Bipolar I Disorder Using Data From a Consumer Credit Reporting Agency	-EXCLUDE on intervention (service engagement/helpseeking behaviour)	
####	Naudi (2018) (ID:87963036)	Full cooperation: zero violence barriers to help-seeking in gender-based violence against women: a research study	-EXCLUDE on intervention (service engagement/helpseeking behaviour)	
####	Navarro-Lashayas (2017) (ID:87852739)	Substance Use and Psychological Distress Is Related With Accommodation Status Among Homeless Immigrants	-EXCLUDE on intervention (service engagement/helpseeking behaviour)	
####	Navathe (2018) (ID:87848596)	Hospital Readmission and Social Risk Factors Identified from Physician Notes.	-EXCLUDE on intervention (service engagement/helpseeking behaviour)	

####	NAVED (2006) (ID:87857193)	Physical violence by husbands: magnitude, disclosure and help-seeking behavior of women in Bangladesh	-EXCLUDE on date (2010)	
####	Neaigus (2016) (ID:87848788)	Community Sexual Bridging Among Heterosexuals at High-Risk of HIV in New York City.	-EXCLUDE on intervention (service engagement/helpseeking behaviour)	
####	Neale (2014) (ID:87848933)	Homeless drug users and information technology: a qualitative study with potential implications for recovery from drug dependence.	-EXCLUDE on intervention (service engagement/helpseeking behaviour)	
####	Neale (2014) (ID:87857609)	Gender sameness and difference in recovery from heroin dependence: A qualitative exploration	-EXCLUDE on population (no housing precarity)<br>-EXCLUDE - but review for literature	
####	Neale (2015) (ID:87848879)	Social and recovery capital amongst homeless hostel residents who use drugs and alcohol.	-EXCLUDE on population (no gender focus; women population <50)<br>-EXCLUDE - but review for literature	
####	Neale (2016) (ID:87850908)	'We are always in some form of contact': Friendships among homeless drug and alcohol users living in hostels.	-EXCLUDE on population (women)	
####	Neale (2018) (ID:87848563)	Do women with complex alcohol and other drug use histories want women-only residential treatment?.	-INCLUDE on title & abstract	-EXCLUDE on intervention (intersectionality)
####	Nebelkopf (2011) (ID:87851308)	Holistic system of care: A ten-year perspective.	-EXCLUDE on intervention (service engagement/helpseeking behaviour)	
####	Negeri (2021) (ID:87853174)	Statistical Analysis of Determinants of Institutional Delivery Service among Reproductive Age Women in Ethiopia	-EXCLUDE on country (High-Income)	
####	Negron (2018) (ID:87851925)	Supporting asylum seekers and refugees who suffer chronic pain: an experience	-EXCLUDE on population (no housing precarity)	
####	NegurÄ (2019) (ID:87849314)	The state policy towards the homeless in Moldova between the 'left hand' and the 'right hand'. The case of chiÈ™u shelter	-EXCLUDE on country (High-Income)	
####	Negura (2020) (ID:87852944)	Homelessness in a Post-Soviet City: Weak Social Support and Institutional Alienation	-EXCLUDE on country (High-Income)	
####	Nehls (2021) (ID:87851448)	Building homes and hopes: the transformative service of YouthBuild Las Vegas	-EXCLUDE on intervention (service engagement/helpseeking behaviour)	
####	Neis (2018) (ID:87853395)	Fragile synchronicities: diverse, disruptive and constraining rhythms of employment-related geographical mobility, paid and unpaid work in the Canadian context	-EXCLUDE on population (no housing precarity)	
####	Neisler (2018) (ID:87848482)	The moderating effect of perceived social support on the relation between heaviness of smoking and quit attempts among adult homeless smokers.	-EXCLUDE on intervention (service engagement/helpseeking behaviour)	
####	Nejtek (2011) (ID:87853029)	Are There Racial/Ethnic Differences in Indigent Inner-City Clients With Dual Diagnoses?	-EXCLUDE on intervention (service engagement/helpseeking behaviour)	
####	Nelson (2012) (ID:87849132)	Moving ahead: evaluation of a work-skills training program for homeless adults.	-EXCLUDE on intervention (service engagement/helpseeking behaviour)	
####	Nelson (2015) (ID:87848860)	Life changes among homeless persons with mental illness: a longitudinal study of housing first and usual treatment.	-EXCLUDE on intervention (service engagement/helpseeking behaviour)	
####	Nelson (2018) (ID:87848502)	Costs Associated with Health Care Services Accessed through VA and in the Community through Medicare for Veterans Experiencing Homelessness.	-EXCLUDE on intervention (service engagement/helpseeking behaviour)	
####	Nelson (2018) (ID:87849347)	Storied realities a case study of homelessness, housing policy, and gender in Whitehorse, Yukon Territory	-EXCLUDE on intervention (service engagement/helpseeking behaviour)	
####	Nelson (2021) (ID:87963277)	Intersectional analysis of cannabis use, stigma and health among marginalized Nigerian women	-EXCLUDE on country (High-Income)	
####	Nelson (2023) (ID:87853052)	The Dynamics of Intimate Partner Violence and Its Impact on HIV Care: A Cross-Sectional Study of People of Mixed Gender and Sexual Preference in Lima, Peru	-EXCLUDE on country (High-Income)	
####	Nematy (2023) (ID:87857542)	LGBTQI + Refugees' and Asylum Seekers' Mental Health: A Qualitative Systematic Review	-EXCLUDE on intervention (service engagement/helpseeking behaviour)	
####	Nemiroff (2011) (ID:87851311)	From homelessness to community: Psychological integration of women who have experienced homelessness.	-EXCLUDE on intervention (service engagement/helpseeking behaviour)	
####	Nemiroff (2012) (ID:87851229)	Beyond rehousing: Community integration of women who have experienced homelessness.	-INCLUDE on title & abstract	-EXCLUDE on intervention (service engagement/helpseeking behaviour)
####	Neovius (2012) (ID:87851660)	Health Care Use During 20 Years Following Bariatric Surgery	-EXCLUDE on intervention (service engagement/helpseeking behaviour)	
####	Nestler (2018) (ID:87851735)	The Family Team at Boston Healthcare for the Homeless Program: an integrated approach to care in outreach settings.	-EXCLUDE on intervention (service engagement/helpseeking behaviour)	

####	Netto (2020) (ID:87857425)	Intersectionality, identity work and migrant progression from low-paid work: A critical realist approach	-EXCLUDE on intervention (service engagement/helpseeking behaviour)
####	Nevard (2021) (ID:87853346)	Conceptualising the social networks of vulnerable children and young people: a systematic review and narrative synthesis	-EXCLUDE on population (people aged under 18 years)
####	Newcomb (2016) (ID:87850932)	Parent perspectives about sexual minority adolescent participation in research and requirements of parental permission.	-EXCLUDE on intervention (service engagement/helpseeking behaviour)
####	NEWMAN (2013) (ID:87857215)	Domestic abuse against elder women and perceived barriers to help-seeking	-EXCLUDE on population (no housing precarity)
####	Newman (2018) (ID:87850845)	Factors associated with length of stay in psychiatric inpatient services in London, UK.	-EXCLUDE on intervention (service engagement/helpseeking behaviour)
####	Newman (2023) (ID:87857509)	Self-Employment or Selfless Employment? Exploration of Factors that Motivate, Facilitate, and Constrain Latina Entrepreneurship from a Family Embeddedness Perspective	-EXCLUDE on population (no housing precarity)
####	Ngo (2016) (ID:87848726)	Community Engagement Compared With Technical Assistance to Disseminate Depression Care Among Low-Income, Minority Women: A Randomized Controlled Effectiveness Study.	-EXCLUDE on intervention (service engagement/helpseeking behaviour)
####	Nguyen (2012) (ID:87851294)	Problem-solving appraisal, gender role conflict, help-seeking behavior, and psychological distress among men who are homeless.	-EXCLUDE on population (women)
####	Nguyen (2021) (ID:87857984)	Supporting Adolescents Curriculum for Mental Health Professionals	-EXCLUDE on intervention (service engagement/helpseeking behaviour)
####	NI (2019) (ID:87857359)	Clarifying the mechanisms and resources that enable the reciprocal involvement of seldom heard groups in health and social care research: a collaborative rapid realist review process	-EXCLUDE on intervention (service engagement/helpseeking behaviour)
####	NICHOLS (2011) (ID:87857009)	Homelessness and the mobile shelter system: public transportation as shelter	-EXCLUDE on country (High-Income)
####	Nichols (2016) (ID:87857839)	Crossing Jordan: An Exploration of Academic Optimism in the Schooling Experiences of Low- Income African American Female Students and Their Perceptions of Their Academic Lives	-EXCLUDE on population (no housing precarity)
####	Nichols (2016) (ID:87963297)	Sex trafficking in the United States: Theory, research, policy, and practice	-EXCLUDE on intervention (service engagement/helpseeking behaviour)
####	Nichols (2022) (ID:87857952)	A State-Level Analysis of Demographic Characteristics and Sex Trafficking Experiences of Survivors	-EXCLUDE on intervention (service engagement/helpseeking behaviour)
####	Nickels (2013) (ID:87857935)	The role of the social environment in non-suicidal self-injury among LGBTQ youth: A mixed methods study	-EXCLUDE on population (no housing precarity)
####	Niederhofer (2014) (ID:87851088)	Mentoring programs also required for children's homes.	-EXCLUDE on population (people aged under 18 years)
####	Nielssen (2020) (ID:87848341)	Comparison of homeless clinic attenders with and without psychotic illness.	-EXCLUDE on intervention (service engagement/helpseeking behaviour)
####	Nijdam-Jones (2017) (ID:87850887)	History of forensic mental health service use among homeless adults with mental illness.	-EXCLUDE on intervention (service engagement/helpseeking behaviour)
####	Nijhawan (2010) (ID:87851366)	Preventive healthcare for underserved women: Results of a prison survey.	-EXCLUDE on intervention (service engagement/helpseeking behaviour)
####	Nikendei (2019) (ID:87848397)	Asylum seekers' mental health and treatment utilization in a three months follow-up study after transfer from a state registration-and reception-center in Germany.	-EXCLUDE on intervention (service engagement/helpseeking behaviour)
####	Niksic (2016) (ID:87852743)	Is cancer survival associated with cancer symptom awareness and barriers to seeking medical help in England? An ecological study	-EXCLUDE on intervention (service engagement/helpseeking behaviour)
####	Nikupeteri (2017) (ID:87850861)	Professionals' critical positionings of women as help-seekers: Finnish women's narratives of help-seeking during post-separation stalking.	-EXCLUDE on population (no housing precarity)
####	Ning (2017) (ID:87857895)	Embodied Sites, Gendered Spaces: Implications of Ethnographic Work “At Home”	-EXCLUDE on intervention (service engagement/helpseeking behaviour)
####	Nipp (2018) (ID:87853299)	Disparities in cancer outcomes across age, sex, and race/ethnicity among patients with pancreatic cancer	-EXCLUDE on population (no housing precarity)

####	Njue (2020) (ID:87852709)	Models of maternal and child healthcare for African refugees: protocol for an exploratory, mixed-methods study	-EXCLUDE on intervention (service engagement/helpseeking behaviour)	
####	Nnawulezi (2019) (ID:87852813)	Understanding Formal Help-Seeking Among Women Whose Partners Are in Abuser Intervention Programs	-INCLUDE on title & abstract	-EXCLUDE (IPV/DVA but little discussion on housing)
####	No (2011) (ID:87851322)	Health Risks and Needs of Lesbian, Gay, Bisexual, Transgender, and Questioning Adolescents Position Statement.	-EXCLUDE on intervention (service engagement/helpseeking behaviour)	
####	Nobari (2021) (ID:87848236)	Severe Housing-Cost Burden and Low-Income Young Children's Exposure to Adverse Experiences: A Cross-Sectional Survey of WIC Participants in Los Angeles County.	-EXCLUDE on population (people aged under 18 years)	
####	Noel (2020) (ID:87848315)	Hearing loss and hearing needs in an adult homeless population: a prospective cross-sectional study.	-EXCLUDE on intervention (service engagement/helpseeking behaviour)	
####	Nomah (2021) (ID:87851636)	Sociodemographic, clinical, and immunological factors associated with SARS-CoV-2 diagnosis and severe COVID-19 outcomes in people living with HIV: a retrospective cohort study	-EXCLUDE on intervention (service engagement/helpseeking behaviour)	
####	Nomamiukor (2018) (ID:87850803)	Examining associations between sleep disturbance and distress tolerance in trauma-exposed psychiatric inpatients.	-EXCLUDE on intervention (service engagement/helpseeking behaviour)	
####	Nor (2016) (ID:87850923)	Mental health predicted by coping, social support, and resilience among young unwed pregnant Malaysian women and mothers living in shelter homes.	-EXCLUDE on country (High-Income)	
####	NORMAN (2010) (ID:87857370)	Improvisational international research: seeking to help children in Ukrainian orphanages sooner than later	-EXCLUDE on population (people aged under 18 years)	
####	Norquist (2023) (ID:87857760)	Illuminating the Black Male Athlete Experience at Community College	-EXCLUDE on population (women)	
####	Norredam (2014) (ID:87853343)	Cancer mortality does not differ between migrants and Danish-born patients	-EXCLUDE on intervention (service engagement/helpseeking behaviour)	
####	Norris (1977) (ID:87850545)	Measuring multiple realities : multidimensional research with a community work organisation concerned with deviance and homelessness	-EXCLUDE on date (2010)	
####	North (2010) (ID:87851379)	A tale of two studies of two disasters: Comparing psychosocial responses to disaster among Oklahoma City bombing survivors and Hurricane Katrina evacuees.	-EXCLUDE on intervention (service engagement/helpseeking behaviour)	
####	NORTH (2012) (ID:87857023)	Predictors of successful tracking over time in a homeless population	-EXCLUDE on intervention (service engagement/helpseeking behaviour)	
####	Northington (2018) (ID:87857974)	ArtThrust Teen Empowerment Program: Teaching Youth to Fly Against Resistance	-EXCLUDE on population (no housing precarity)	
####	Nossel (2016) (ID:87848778)	Use of Peer Staff in a Critical Time Intervention for Frequent Users of a Psychiatric Emergency Room.	-EXCLUDE on intervention (service engagement/helpseeking behaviour)	
####	NOTABLE LGBTQIA+... (2023) (ID:87858059)	NOTABLE LGBTQIA+ LEADERS	-EXCLUDE on intervention (service engagement/helpseeking behaviour)	
####	Nourie (2018) (ID:87963227)	An Intersectional Feminist Perspective on LGBTQ Youth in Foster Care: Implications for Service Providers.	-EXCLUDE on population (people aged under 18 years)	
####	Nousiainen (2021) (ID:87849310)	Frail paths home: former long-term homeless women and men narrate their experiences and meanings of home	-EXCLUDE on intervention (service engagement/helpseeking behaviour)	
####	Novak (2021) (ID:87853438)	Incidence and predictors of early and delayed renal function decline after aortic aneurysm repair in the Vascular Quality Initiative database	-EXCLUDE on intervention (service engagement/helpseeking behaviour)	
####	Novick (2012) (ID:87849115)	The intersection of everyday life and group prenatal care for women in two urban clinics.	-EXCLUDE on intervention (service engagement/helpseeking behaviour)	
####	NSPCC (2018) (ID:87857272)	Understanding the experiences of young people forced to move across borders	-EXCLUDE on intervention (service engagement/helpseeking behaviour)	
####	Nugent (2022) (ID:87963243)	Not Just a 'Youth Problem': LGBT+ Experiences of Homelessness Across the Life Course in Fife, Scotland	-EXCLUDE on intervention (service engagement/helpseeking behaviour)	
####	NURIUS (2011) (ID:87857205)	Intimate partner survivors' help-seeking and protection efforts: a person-oriented analysis	-INCLUDE on title & abstract	-EXCLUDE (IPV/DVA but little discussion on housing)
####	Nurmela (2018) (ID:87848578)	Identification of major depressive disorder among the long-term unemployed.	-EXCLUDE on intervention (service engagement/helpseeking behaviour)	

####	NUSKE (2013) (ID:87857109)	A narrative analysis of help-seeking behaviour and critical change points for recovering problem gamblers: the power of storytelling	-EXCLUDE on population (no housing precarity)	
####	Nuttbrock (2018) (ID:87850786)	Transgender sex work and society.	-EXCLUDE on intervention (service engagement/helpseeking behaviour)	
####	Nwaru (2014) (ID:87848982)	Muscular fitness and re-employment among unemployed job seekers in Finland: a three-year follow-up study.	-EXCLUDE on intervention (service engagement/helpseeking behaviour)	
####	Nyamathi (2012) (ID:87849120)	Characteristics of homeless youth who use cocaine and methamphetamine.	-EXCLUDE on intervention (service engagement/helpseeking behaviour)	
####	Nyamathi (2012) (ID:87851245)	Correlates of depressed mood among young stimulant-using homeless gay and bisexual men.	-EXCLUDE on population (women)	
####	Nyamathi (2014) (ID:87851101)	Predictors of high level of hostility among homeless men on parole.	-EXCLUDE on population (women)	
####	Nyamathi (2015) (ID:87848890)	Correlates of self-reported incarceration among homeless gay and bisexual stimulant-using young adults.	-EXCLUDE on intervention (service engagement/helpseeking behaviour)	
####	Nyamathi (2017) (ID:87848666)	Violent Crime in the Lives of Homeless Female Ex-Offenders.	-EXCLUDE on intervention (service engagement/helpseeking behaviour)	
####	Nyamathi (2017) (ID:87850889)	Impact of tailored interventions to reduce drug use and sexual risk behaviors among homeless gay and bisexual men.	-EXCLUDE on population (women)	
####	Nyamathi (2018) (ID:87850818)	Correlates of treatment readiness among formerly incarcerated homeless women.	-INCLUDE on title & abstract	-EXCLUDE on intervention (service engagement/helpseeking behaviour)
####	Nyamathi (2021) (ID:87850630)	Effect of a nurse-led community health worker intervention on latent tuberculosis medication completion among homeless adults.	-EXCLUDE on intervention (service engagement/helpseeking behaviour)	
####	Nydegger (2020) (ID:87857759)	Exploring patterns of substance use among highly vulnerable Black women at-risk for HIV through a syndemics framework: A qualitative study	-EXCLUDE on intervention (service engagement/helpseeking behaviour)	
####	Nygaard (2018) (ID:87848522)	Frostbite vs Burns: Increased Cost of Care and Use of Hospital Resources.	-EXCLUDE on intervention (service engagement/helpseeking behaviour)	
####	O'BRIEN (2013) (ID:87851738)	The Sisters of Providence.	-EXCLUDE on intervention (service engagement/helpseeking behaviour)	
####	O'Brien (2015) (ID:87848834)	Health, perceived quality of life and health services use among homeless illicit drug users.	-EXCLUDE on intervention (service engagement/helpseeking behaviour)	
####	O'Brien (2016) (ID:87963168)	Intersectionality and adolescent domestic violence and abuse: addressing “classed sexism” and improving service provision	-EXCLUDE on population (women)	
####	O'Brien (2020) (ID:87851615)	Incidence, clinical features, and outcomes of COVID-19 in Canada: impact of sex and age	-EXCLUDE on population (no housing precarity)	
####	O'Brien (2022) (ID:87852676)	Development of a Shelter-based Health Empowerment Program for Pregnant and Parenting Youth Experiencing Homelessness	-EXCLUDE on intervention (service engagement/helpseeking behaviour)	
####	O'Campo (2016) (ID:87848719)	How did a Housing First intervention improve health and social outcomes among homeless adults with mental illness in Toronto? Two-year outcomes from a randomised trial.	-EXCLUDE on intervention (service engagement/helpseeking behaviour)	
####	O'Carroll (2019) (ID:87848398)	Making sense of street chaos: an ethnographic exploration of homeless people's health service utilization.	-EXCLUDE on population (no gender focus; women population <50)	
####	O'Connell (2010) (ID:87849265)	National dissemination of supported housing in the VA: model adherence versus model modification.	-EXCLUDE on intervention (service engagement/helpseeking behaviour)	
####	O'Connor (2019) (ID:87851910)	CV Housing Coalition helps build communities	-EXCLUDE on evidence and form (evidence not in written form or presented as research output)	
####	O'Donnell (2011) (ID:87849161)	Acceptance of interferon-gamma release assay by a high-risk urban cohort.	-EXCLUDE on intervention (service engagement/helpseeking behaviour)	
####	O'Donovan (2019) (ID:87848411)	A Place to Call Home: Hearing the Perspectives of People Living with Homelessness and Mental Illness Through Service Evaluation.	-EXCLUDE on intervention (service engagement/helpseeking behaviour)	
####	O'FLAHERTY (2008) (ID:87856979)	Homeless shelters for single adults: why does their population change?	-EXCLUDE on date (2010)	
####	O'Herlihy (2004) (ID:87853700)	Characteristics of the Residents of In-Patient Child and Adolescent Mental Health Services in England and Wales	-EXCLUDE on date (2010)	

####	O'Malley (2017) (ID:87850862)	Revealing the prevalence and consequences of food insecurity in children with epilepsy.	-EXCLUDE on population (no housing precarity)
####	O'Malley (2022) (ID:87857708)	An Exploration of the Involuntary Celibate (Incel) Subculture Online	-EXCLUDE on population (no housing precarity)
####	O'Mullan (2022) (ID:87852835)	Strengthening the service experiences of women impacted by gambling-related intimate partner violence	-EXCLUDE on intervention (service engagement/helpseeking behaviour)
####	O'Neill (2019) (ID:87857619)	Women's desire for the kaleidoscope of authenticity, balance and challenge: A multi-method study of female health workers' careers	-EXCLUDE on population (no housing precarity)
####	O'Toole (2011) (ID:87849168)	Building care systems to improve access for high-risk and vulnerable veteran populations.	-EXCLUDE on intervention (service engagement/helpseeking behaviour)
####	O'Toole (2015) (ID:87848823)	Needing Primary Care But Not Getting It: The Role of Trust, Stigma and Organizational Obstacles reported by Homeless Veterans.	-EXCLUDE on population (women)
####	O'Toole (2015) (ID:87848861)	Tailoring Outreach Efforts to Increase Primary Care Use Among Homeless Veterans: Results of a Randomized Controlled Trial.	-EXCLUDE on population (women)
####	O'Toole (2016) (ID:87848759)	Tailoring Care to Vulnerable Populations by Incorporating Social Determinants of Health: the Veterans Health Administration's "Homeless Patient Aligned Care Team" Program.	-EXCLUDE on population (women)
####	O'Toole (2018) (ID:87848557)	Population-Tailored Care for Homeless Veterans and Acute Care Use, Cost, and Satisfaction: A Prospective Quasi-Experimental Trial.	-EXCLUDE on intervention (service engagement/helpseeking behaviour)
####	O98 - Optimising antenatal... (Christie) (ID:87851472)	O98 - Optimising antenatal care for marginalised and at-risk women at the Kirketon Road Centre (KRC), Kings Cross, NSW	-EXCLUDE on intervention (service engagement/helpseeking behaviour)
####	Obakhume (2014) (ID:87851948)	An aftercare program for female survivors of domestic abuse: A grant proposal (California).	-EXCLUDE on evidence and form (evidence not in written form or presented as research output)
####	Obeng-odoom (2009) (ID:87853689)	Has the Habitat for Humanity Housing Scheme achieved its goals? A Ghanaian case study	-EXCLUDE on date (2010)
####	Ober (2012) (ID:87849106)	If you provide the test, they will take it: factors associated with HIV/STI Testing in a representative sample of homeless youth in Los Angeles.	-EXCLUDE on population (women)
####	Ober (2018) (ID:87848526)	Patient predictors of substance use disorder treatment initiation in primary care.	-EXCLUDE on intervention (service engagement/helpseeking behaviour)
####	Obimakinde (2023) (ID:87851870)	Physical, mental and healthcare issues of children on the street of Ibadan, Nigeria	-EXCLUDE on country (High-Income)
####	Ochsenreither (2019) (ID:87858125)	A Knitting Group for Survivors of Intimate Partner Violence: Untangling the Unraveling	-EXCLUDE on intervention (service engagement/helpseeking behaviour)
####	Odden (2019) (ID:87853285)	Trajectories of Nonagenarian Health: Sex, Age, and Period Effects	-EXCLUDE on population (no housing precarity)
####	Odhiambo (2023) (ID:87853230)	Structural violence and the uncertainty of viral undetectability for African, Caribbean and Black people living with HIV in Canada: an institutional ethnography	-EXCLUDE on intervention (service engagement/helpseeking behaviour)
####	Ogden (2021) (ID:87853269)	"You need money to get high, and that's the easiest and fastest way:" A typology of sex work and health behaviours among people who inject drugs	-EXCLUDE on intervention (service engagement/helpseeking behaviour)
####	Ogunyemi (2020) (ID:87963264)	Microaggressions in the learning environment: A systematic review.	-EXCLUDE on intervention (service engagement/helpseeking behaviour)
####	Okamura (2014) (ID:87848974)	Suicidal behavior among homeless people in Japan.	-EXCLUDE on intervention (service engagement/helpseeking behaviour)
####	Okamura (2015) (ID:87848827)	Characteristics of Individuals With Mental Illness in Tokyo Homeless Shelters.	-EXCLUDE on intervention (service engagement/helpseeking behaviour)
####	Okeowo (2019) (ID:87857985)	Radical Care	-EXCLUDE on intervention (service engagement/helpseeking behaviour)
####	Okoro (2020) (ID:87857633)	"We get double slammed!": Healthcare experiences of perceived discrimination among low-income African-American women	-EXCLUDE on population (no housing precarity)
####	Okunseri (2010) (ID:87849247)	Factors associated with reported need for dental care among people who are homeless using assistance programs.	-EXCLUDE on population (women)

####	Okuyemi (2013) (ID:87849044)	Motivational interviewing to enhance nicotine patch treatment for smoking cessation among homeless smokers: a randomized controlled trial.	-EXCLUDE on population (women)	
####	Olafiranye (2011) (ID:87852043)	Anxiety and Cardiovascular Risk: Review of Epidemiological and Clinical Evidence: The Journal of Psychiatry	-EXCLUDE on population (no housing precarity)	
####	Olaiya (2018) (ID:87848539)	Exchange sex among people receiving medical care for HIV in the United States - medical monitoring project 2009-2013.	-EXCLUDE on intervention (service engagement/helpseeking behaviour)	
####	Olesek (2016) (ID:87848739)	Cluster B Personality Disorder Traits as a Predictor of Therapeutic Alliance Over Time in Residential Treatment for Substance Use Disorders.	-EXCLUDE on intervention (service engagement/helpseeking behaviour)	
####	Oliffe (2020) (ID:87963377)	Help-seeking prior to male suicide: Bereaved men perspectives	-EXCLUDE on population (women)	
####	Oliffe (2021) (ID:87848207)	Segmenting or Summing the Parts? A Scoping Review of Male Suicide Research in Canada.	-EXCLUDE on population (women)	
####	Oliner (2022) (ID:87858011)	The Water We Were Swimming In: Transgender and Gender Nonconforming Students' Lived Experiences in Engineering	-EXCLUDE on population (no housing precarity)	
####	Oliva (2016) (ID:87848783)	Patient perspectives on an opioid overdose education and naloxone distribution program in the U.S. Department of Veterans Affairs.	-EXCLUDE on intervention (service engagement/helpseeking behaviour)	
####	OLIVER (2005) (ID:87857149)	Help-seeking behaviour in men and women with common mental health problems: cross-sectional study	-EXCLUDE on date (2010)	
####	**Oliver (2012) (ID:87849125)**	**Sexual health: the role of sexual health services among homeless young women living in Toronto, Canada.**	**-INCLUDE on title & abstract**	**-INCLUDE on full study**
####	Oliver (2012) (ID:87849321)	Healing home: Health and homelessness in the life stories of young women	-INCLUDE on title & abstract	-EXCLUDE on intervention (intersectionality)
####	Oliver (2015) (ID:87853399)	FAMILY MATTERS: A STRENGTHS-BASED FAMILY RESILIENCY PERSPECTIVE TOWARD IMPROVING THE HEALTH OF YOUNG WOMEN EXPERIENCING HOMELESSNESS	-EXCLUDE on intervention (service engagement/helpseeking behaviour)	
####	Olivet (2021) (ID:87963119)	Racial inequity and homelessness: findings from the SPARC study	-EXCLUDE on population (no gender focus; women population <50)<br>-EXCLUDE - but review for literature	
####	Olszewski (2015) (ID:87853281)	Race-Specific Features and Outcomes of Nodular Lymphocyte-Predominant Hodgkin Lymphoma: Analysis of the National Cancer Data Base	-EXCLUDE on population (no housing precarity)	
####	Olutoyin (2022) (ID:87851882)	Exposure to job-related violence among young female sex workers in urban slums of Southwest Nigeria	-EXCLUDE on country (High-Income)	
####	Omura (2014) (ID:87851097)	Incarceration among street-involved youth in a Canadian study: Implications for health and policy interventions.	-EXCLUDE on intervention (service engagement/helpseeking behaviour)	
####	Onder (2012) (ID:87849136)	Polypharmacy in nursing home in Europe: results from the SHELTER study.	-EXCLUDE on population (no housing precarity)	
####	Onishenko (2010) (ID:87858089)	A Theoretically Critical Gaze on the Canadian Equal Marriage Debate: Breaking the Binaries	-EXCLUDE on population (no housing precarity)	
####	Onyeka (2013) (ID:87849035)	Coexisting social conditions and health problems among clients seeking treatment for illicit drug use in Finland: the HUUTI study.	-EXCLUDE on intervention (service engagement/helpseeking behaviour)	
####	Opare-Henaku (2017) (ID:87848629)	Culturally prescribed beliefs about mental illness among the Akan of Ghana.	-EXCLUDE on country (High-Income)	
####	Oral history interview... (2015) (ID:87849373)	Oral history interview with Nazima Ali, 2015	-EXCLUDE on evidence and form (evidence not in written form or presented as research output)	
####	ORAL PRESENTATION. (2011) (ID:87851675)	ORAL PRESENTATION.	-EXCLUDE on evidence and form (evidence not in written form or presented as research output)	
####	Orchard (2016) (ID:87851643)	'You just have to be smart': spatial practices and subjectivity among women in sex work in London, Ontario.	-EXCLUDE on intervention (service engagement/helpseeking behaviour)	
####	Orchard (2021) (ID:87857867)	Money, agency, and self-care among cisgender and trans people in sex work	-EXCLUDE on intervention (service engagement/helpseeking behaviour)	
####	Orui (2017) (ID:87848657)	Practical Report on Long-term Disaster Mental Health Services Following the Great East Japan Earthquake: Psychological and Social Background of Evacuees in Sendai City in the Mid- to Long-term Post-disaster Period.	-EXCLUDE on intervention (service engagement/helpseeking behaviour)	

####	Orwat (2011) (ID:87849228)	Factors associated with attendance in 12-step groups (Alcoholics Anonymous/Narcotics Anonymous) among adults with alcohol problems living with HIV/AIDS.	-EXCLUDE on intervention (service engagement/helpseeking behaviour)	
####	Osborne (2015) (ID:87857447)	Intersectionality and kyriarchy: A framework for approaching power and social justice in planning and climate change adaptation	-EXCLUDE on population (no housing precarity)	
####	Oscar (2018) (ID:87851921)	Moving Community Foundation Dollars from Wall Street to Main Street: A growing movement sees community foundations invest in local financial institutions and development projects, empowering entrepreneurship in the underserved communities that need it most.	-EXCLUDE on evidence and form (evidence not in written form or presented as research output)	
####	Osei (2020) (ID:87848305)	Health problems and healthcare service utilisation amongst homeless adults in Africa- a scoping review.	-EXCLUDE on country (High-Income)	
####	Osei-Kwasi (2019) (ID:87848410)	"I cannot sit here and eat alone when I know a fellow Ghanaian is suffering": Perceptions of food insecurity among Ghanaian migrants.	-EXCLUDE on population (no housing precarity)	
####	Oser (2016) (ID:87852684)	African American Female Offender's Use of Alternative and Traditional Health Services After Re-Entry: Examining the Behavioral Model for Vulnerable Populations	-INCLUDE on title & abstract	-EXCLUDE on intervention (service engagement/helpseeking behaviour)
####	Osilla (2016) (ID:87848721)	Feasibility of a computer-assisted social network motivational interviewing intervention for substance use and HIV risk behaviors for housing first residents.	-EXCLUDE on population (women)	
####	Oskar (2021) (ID:87851480)	Reproductive Ethics and Family	-EXCLUDE on evidence and form (evidence not in written form or presented as research output)	
####	OSUJI (2015) (ID:87856931)	History of abuse and the experience of homelessness: a framework for assisting women overcome housing instability	-EXCLUDE on intervention (service engagement/helpseeking behaviour)	
####	Oswald (2022) (ID:87857549)	Intersectional expansiveness borne at the neuroqueer nexus	-EXCLUDE on population (no housing precarity)	
####	Otazu (2012) (ID:87853386)	Mortality and morbidity in very low birth weight infants in the Basque Country and Navarra (2001-2006): population-based study	-EXCLUDE on population (no housing precarity)	
####	Otiniano (2023) (ID:87963050)	Homelessness and discrimination among US adults: The role of intersectionality	-EXCLUDE on population (no gender focus; women population <50)<br>-EXCLUDE - but review for literature	
####	Ouchi (2019) (ID:87853408)	Association of Emergency Clinicians' Assessment of Mortality Risk With Actual 1-Month Mortality Among Older Adults Admitted to the Hospital	-EXCLUDE on population (no housing precarity)	
####	Oudshoorn (2021) (ID:87857887)	A critical narrative inquiry to understand the impacts of an overdose prevention site on the lives of site users	-EXCLUDE on intervention (service engagement/helpseeking behaviour)	
####	OWENS (2005) (ID:87857182)	A qualitative study of help seeking and primary care consultation prior to suicide	-EXCLUDE on date (2010)	
####	OYEWUWO-GASSIKIA (2020) (ID:87857102)	Black Muslim women’s domestic violence help-seeking strategies: types, motivations, and outcomes	-INCLUDE on title & abstract	-EXCLUDE (IPV/DVA but little discussion on housing)
####	Ozmete (2022) (ID:87852907)	Problems and issues concerning social integration of elderly refugees in Turkey	-EXCLUDE on country (High-Income)	
####	Pacheco (2014) (ID:87848931)	The voices of Brazilian women breaking free from intimate partner violence.	-INCLUDE on title & abstract	-EXCLUDE (IPV/DVA but little discussion on housing)
####	Padgett (2011) (ID:87849236)	Substance use outcomes among homeless clients with serious mental illness: comparing Housing First with Treatment First programs.	-EXCLUDE on intervention (service engagement/helpseeking behaviour)	
####	Padgett (2011) (ID:87851318)	Moving into the fourth decade of ACT.	-EXCLUDE on evidence and form (evidence not in written form or presented as research output)	
####	Padgett (2016) (ID:87848772)	Trajectories of Recovery Among Formerly Homeless Adults With Serious Mental Illness.	-EXCLUDE on intervention (service engagement/helpseeking behaviour)	
####	Padgett (2016) (ID:87963307)	Housing First: Ending homelessness, transforming systems, and changing lives	-EXCLUDE on population (women)	
####	Padgett (2019) (ID:87857420)	Gender, Everyday Resistance and Bodily Integrity: Women’s Lives on Delhi Streets	-EXCLUDE on country (High-Income)	
####	Padilla (2020) (ID:87848348)	Mental health, substance use and HIV risk behaviors among HIV-positive adults who experienced homelessness in the United States - Medical Monitoring Project, 2009-2015.	-EXCLUDE on intervention (service engagement/helpseeking behaviour)	

####	Pagano (2021) (ID:87848233)	Impacts of COVID-19 on residential treatment programs for substance use disorder.	-EXCLUDE on intervention (service engagement/helpseeking behaviour)	
####	Page (2012) (ID:87849131)	Characteristics of homeless adults with serious mental illnesses served by three street-level federally funded homelessness programs.	-EXCLUDE on intervention (service engagement/helpseeking behaviour)	
####	Page (2016) (ID:87963019)	Forgotten youth: Homeless LGBT youth of color and the Runaway and Homeless Youth Act	-EXCLUDE on intervention (service engagement/helpseeking behaviour)	
####	Page-Broughton (2020) (ID:87858032)	"I Know Who I Am Today": Experiences and Reflections of System-Involved Youth	-EXCLUDE on intervention (service engagement/helpseeking behaviour)	
####	Pahwa (2019) (ID:87848364)	The Ties That Bind and Unbound Ties: Experiences of Formerly Homeless Individuals in Recovery From Serious Mental Illness and Substance Use.	-EXCLUDE on population (no gender focus; women population <50)<br>-EXCLUDE - but review for literature	
####	Pahwa (2022) (ID:87857553)	Is It Safe? Community Integration for Individuals With Serious Mental Illnesses	-EXCLUDE on intervention (service engagement/helpseeking behaviour)	
####	Painter (2018) (ID:87848579)	High inpatient utilization among Veterans Health Administration patients with substance-use disorders and co-occurring mental health conditions.	-EXCLUDE on population (women)	
####	Paisi (2022) (ID:87853094)	Barriers and facilitators to hepatitis C screening and treatment for people with lived experience of homelessness: A mixed-methods systematic review	-EXCLUDE on population (women)	
####	Pakhale (2018) (ID:87848562)	Management and Point-of-Care for Tobacco Dependence (PROMPT): a feasibility mixed methods community-based participatory action research project in Ottawa, Canada.	-EXCLUDE on intervention (service engagement/helpseeking behaviour)	
####	Palaia (2022) (ID:87852777)	Evaluation of dental demands and needs of people experiencing homelessness in the city of Rome, Italy	-EXCLUDE on population (women)	
####	Palepu (2012) (ID:87849101)	Quality of life themes in Canadian adults and street youth who are homeless or hard-to-house: a multi-site focus group study.	-EXCLUDE on intervention (service engagement/helpseeking behaviour)	
####	Palepu (2013) (ID:87851160)	Substance use and access to health care and addiction treatment among homeless and vulnerably housed persons in three Canadian cities.	-EXCLUDE on population (women)	
####	Palepu (2013) (ID:87852710)	Housing First Improves Residential Stability in Homeless Adults With Concurrent Substance Dependence and Mental Disorders	-EXCLUDE on intervention (service engagement/helpseeking behaviour)	
####	Pan (2020) (ID:87848288)	The Relationship Between Housing Status and Substance Use and Sexual Risk Behaviors Among People Currently Seeking or Receiving Services in Substance Use Disorder Treatment Programs.	-EXCLUDE on intervention (service engagement/helpseeking behaviour)	
####	Pan (2021) (ID:87857532)	Sexual citizenship and Asian immigrants in post-marriage equality Australia	-EXCLUDE on population (no housing precarity)	
####	Panahpour (2020) (ID:87848270)	Factors associated with low-acuity hospital admissions in a public safety-net setting: a cross-sectional study.	-EXCLUDE on population (women)	
####	Panczak (2016) (ID:87848710)	Work activity in the process of recovery - an interpretive phenomenological analysis of the experiences of people with a schizophrenia spectrum diagnosis.	-EXCLUDE on intervention (service engagement/helpseeking behaviour)	
####	Pantell (2019) (ID:87853087)	Associations between unstable housing, obstetric outcomes, and perinatal health care utilization	-EXCLUDE on intervention (service engagement/helpseeking behaviour)	
####	Parashar (2011) (ID:87849176)	Sticking to it: the effect of maximally assisted therapy on antiretroviral treatment adherence among individuals living with HIV who are unstably housed.	-EXCLUDE on intervention (service engagement/helpseeking behaviour)	
####	PARDECK (2002) (ID:87856965)	An exploration of violence among homeless women with emotional disabilities: implications for practice and policy	-EXCLUDE on date (2010)	
####	Park (2010) (ID:87849260)	Behavioral health services use among heads of homeless and housed poor families.	-EXCLUDE on intervention (service engagement/helpseeking behaviour)	
####	Park (2011) (ID:87851314)	Physical and mental health, cognitive development, and health care use by housing status of low-income young children in 20 American cities: A prospective cohort study.	-EXCLUDE on population (people aged under 18 years)	
####	Park (2020) (ID:87853125)	Structural Barriers to Women's Sustained Engagement in HIV Care in Southern California	-INCLUDE on title & abstract	-EXCLUDE on intervention (intersectionality)

####	Park (2023) (ID:87857923)	Participant Experiences in Student Recovery Programs in Canada: An Interpretative Phenomenological Analysis	-EXCLUDE on intervention (service engagement/helpseeking behaviour)
####	Parker (2012) (ID:87849094)	Barriers to care and service needs among chronically homeless persons in a housing first program.	-EXCLUDE on population (women)
####	Parker (2013) (ID:87851184)	The reality of homeless mobility and implications for improving care.	-EXCLUDE on intervention (service engagement/helpseeking behaviour)
####	Parker (2014) (ID:87848980)	Differences in risk behaviors, care utilization, and comorbidities in homeless persons based on HIV status.	-EXCLUDE on intervention (service engagement/helpseeking behaviour)
####	Parker (2018) (ID:87848544)	Understanding consumers' initial expectations of community-based residential mental health rehabilitation in the context of past experiences of care: A mixed-methods pragmatic grounded theory analysis.	-EXCLUDE on intervention (service engagement/helpseeking behaviour)
####	Parker (2019) (ID:87848480)	Consumers' understanding and expectations of a community-based recovery-oriented mental health rehabilitation unit: a pragmatic grounded theory analysis.	-EXCLUDE on intervention (service engagement/helpseeking behaviour)
####	Parker (2020) (ID:87858042)	"Sing a Black Girl Song:" Black Womxn Survivors' Narratives of Interpersonal Violence, Social and Cultural Messages, and Healing Praxis	-EXCLUDE on population (no housing precarity)
####	Parker (2022) (ID:87963088)	Pandemic precarity and everyday disparity: gendered housing needs in North America	-EXCLUDE on intervention (service engagement/helpseeking behaviour)
####	Parks (2014) (ID:87858024)	A qualitative inquiry exploring out college students' experiences with queer content in secondary schools	-EXCLUDE on population (no housing precarity)
####	Parnell (2019) (ID:87850774)	Attitudinal and behavioural responses to increasing tobacco control regulation among high smoking prevalence groups: A qualitative study.	-EXCLUDE on intervention (service engagement/helpseeking behaviour)
####	Parnell (2020) (ID:87848323)	Factors influencing the willingness of community service organisation staff to provide smoking cessation support: a qualitative study.	-EXCLUDE on intervention (service engagement/helpseeking behaviour)
####	Parpouchi (2016) (ID:87848733)	Food Insecurity among Homeless Adults with Mental Illness.	-EXCLUDE on intervention (service engagement/helpseeking behaviour)
####	Parra (2023) (ID:87853017)	COVID-19-Related Household Job Loss and Mental Health in a Nationwide United States Sample of Sexual Minority Adolescents	-EXCLUDE on population (no housing precarity)
####	PARRY-CROOKE (1998) (ID:87856969)	Addressing the needs of homeless women with mental health problems	-EXCLUDE on date (2010)
####	Parsell (2018) (ID:87848595)	Self-management of health care: multimethod study of using integrated health care and supportive housing to address systematic barriers for people experiencing homelessness.	-EXCLUDE on intervention (service engagement/helpseeking behaviour)
####	Parsons (2015) (ID:87857679)	'Are we not human?' Stories of stigma, disability and HIV from Lusaka, Zambia and their implications for access to health services	-EXCLUDE on country (High-Income)
####	Patel (2010) (ID:87851363)	Women's right to mental health.	-EXCLUDE on intervention (service engagement/helpseeking behaviour)
####	Patel (2013) (ID:87853115)	How Do Social Factors Explain Outcomes in Non-Small-Cell Lung Cancer Among Hispanics in California? Explaining the Hispanic Paradox	-EXCLUDE on population (no housing precarity)
####	Patel (2014) (ID:87853241)	Race and sex disparities in the treatment of older patients with T1a renal cell carcinoma: A comorbidity-controlled competing-risks model	-EXCLUDE on population (no housing precarity)
####	Patel (2016) (ID:87853361)	Rising population of survivors of oral squamous cell cancer in the United States	-EXCLUDE on population (no housing precarity)
####	Patel (2021) (ID:87853313)	Determinants of infant mortality in Pakistan: evidence from Pakistan Demographic and Health Survey 2017-18	-EXCLUDE on country (High-Income)
####	PATTERSON (2009) (ID:87857247)	Understanding rape survivors' decisions not to seek help from formal social systems	-EXCLUDE on date (2010)
####	Patterson (2013) (ID:87849020)	Housing First improves subjective quality of life among homeless adults with mental illness: 12-month findings from a randomized controlled trial in Vancouver, British Columbia.	-EXCLUDE on population (women)

####	Patterson (2015) (ID:87848846)	History of foster care among homeless adults with mental illness in Vancouver, British Columbia: a precursor to trajectories of risk.	-EXCLUDE on population (no gender focus; women population <50)	
####	Patterson (2015) (ID:87848881)	Exiting homelessness: perceived changes, barriers, and facilitators among formerly homeless adults with mental disorders.	-EXCLUDE on population (women)	
####	Patterson (2022) (ID:88019127)	Seeking of help and support after experiencing sexual harm: considerations for cisgender women, cisgender men and gender-diverse people	-EXCLUDE on population (no housing precarity)	
####	Patton (2015) (ID:87852847)	Adolescent Health Globally: Issues and Challenges	-EXCLUDE on intervention (service engagement/helpseeking behaviour)	
####	Paudyal (2019) (ID:87848455)	Perceived roles and barriers in caring for the people who are homeless: a survey of UK community pharmacists.	-EXCLUDE on intervention (service engagement/helpseeking behaviour)	
####	Paudyal (2020) (ID:87848352)	'If I die, I die, I don't care about my health': Perspectives on self-care of people experiencing homelessness.	-EXCLUDE on population (women)	
####	Paul (2018) (ID:87857492)	Coping and resilience among ethnoracial individuals experiencing homelessness and mental illness	-EXCLUDE on population (women)	
####	Pauly (2019) (ID:87848369)	"There is a Place": impacts of managed alcohol programs for people experiencing severe alcohol dependence and homelessness.	-EXCLUDE on population (women)	
####	Payton (2012) (ID:87851438)	Isolation, prejudice and missed opportunities: middle eastern women and domestic violence.	-INCLUDE on title & abstract	-EXCLUDE on evidence and form (evidence not in written form or presented as research output)
####	Pease (2017) (ID:87963065)	Critical ethics of care in social work: Transforming the politics and practices of caring	-EXCLUDE on intervention (service engagement/helpseeking behaviour)	
####	PECKOVER (2003) (ID:87857332)	'I could have just done with a little more help': an analysis of women's help-seeking from health visitors in the context of domestic violence	-EXCLUDE on date (2010)	
####	Pecoraro (2013) (ID:87849051)	Factors contributing to dropping out from and returning to HIV treatment in an inner city primary care HIV clinic in the United States.	-EXCLUDE on population (women)	
####	Pedersen (2012) (ID:87849145)	Associations between deprived life circumstances, wellbeing and self-rated health in a socially marginalized population.	-EXCLUDE on population (no gender focus; women population <50)	
####	Pedersen (2018) (ID:87848555)	Homeless people's experiences of medical respite care following acute hospitalisation in Denmark.	-EXCLUDE on intervention (service engagement/helpseeking behaviour)	
####	Pedersen (2018) (ID:87848565)	Predictors of Retention in an Alcohol and Risky Sex Prevention Program for Homeless Young Adults.	-EXCLUDE on intervention (service engagement/helpseeking behaviour)	
####	Pedersen (2020) (ID:87850709)	Renaissance social services supportive housing outreach team program: An outcome evaluation.	-EXCLUDE on intervention (service engagement/helpseeking behaviour)	
####	Peja (2017) (ID:87857847)	Domestic Violence Among Asian Indian Immigrant Women in the United States	-INCLUDE on title & abstract	-EXCLUDE (IPV/DVA but little discussion on housing)
####	PEMBERTON (2010) (ID:87857191)	No shame in seeking help	-EXCLUDE on population (no housing precarity)	
####	Penna (2012) (ID:87853233)	Care of adolescents sheltered in maternity hospitals from the perspective of health professionals	-EXCLUDE on country (High-Income)	
####	Peretz (2020) (ID:87857536)	Impacts of Men's Gender-transformative Personal Narratives: A Qualitative Evaluation of the Men's Story Project	-EXCLUDE on population (women)	
####	Perez (2011) (ID:87849233)	"Couch surfing" of Latino foster care alumni: reliance on peers as social capital.	-EXCLUDE on intervention (service engagement/helpseeking behaviour)	
####	Perez-Merino (2014) (ID:87852017)	Strategies for enhancing the delivery of person-centred care	-EXCLUDE on population (no housing precarity)	
####	Perez-Vazquez (2022) (ID:87852842)	Women Refugee's Perceptions, Experiences and Coping Mechanisms in Situations of Sexual and Gender-Based Violence (SGBV): A Metasynthesis	-EXCLUDE on intervention (service engagement/helpseeking behaviour)	
####	Perkins (2020) (ID:87857697)	Gender-Specific Services for Women Released from Prison: An Appreciative Inquiry	-INCLUDE on title & abstract	-EXCLUDE on target group (no housing precarity)
####	Perry (2013) (ID:87849084)	Adults with intellectual disabilities and challenging behaviour: the costs and outcomes of in- and out-of-area placements.	-EXCLUDE on population (no housing precarity)	
####	Perry (2017) (ID:87853358)	Association Between Insurance Status at Diagnosis and Overall Survival in Chronic Myeloid Leukemia: A Population-Based Study	-EXCLUDE on population (no housing precarity)	

####	PERRY-YOUNG (2018) (ID:87857230)	How people come to recognise a problem and seek medical help for a person showing early signs of dementia: a systematic review and meta-ethnography	-EXCLUDE on population (no housing precarity)	
####	PERRYMAN (2011) (ID:87857207)	The perceived challenges facing alcohol treatment services in England: a qualitative study of service providers	-EXCLUDE on intervention (service engagement/helpseeking behaviour)	
####	Perumal (2019) (ID:87850773)	Gender, perceived health status, and social support amongst Oahu's homeless.	-EXCLUDE on intervention (service engagement/helpseeking behaviour)	
####	Pesce (2011) (ID:87849210)	Deliberation to enhance awareness of and prioritize socioeconomic interventions for health.	-EXCLUDE on intervention (service engagement/helpseeking behaviour)	
####	Petering (2021) (ID:87848249)	Can Better Emotion Regulation Protect Against Interpersonal Violence in Homeless Youth Social Networks?.	-EXCLUDE on intervention (service engagement/helpseeking behaviour)	
####	Peterkin (2023) (ID:87848136)	Pre-Paid Phone Distribution: A Tool for Improving Healthcare Engagement for People with Substance Use Disorder.	-EXCLUDE on intervention (service engagement/helpseeking behaviour)	
####	Peterman (2020) (ID:87963153)	Pandemics and violence against women and children	-EXCLUDE on intervention (service engagement/helpseeking behaviour)	
####	Peters (2021) (ID:87850649)	Shining a light on the experiences of staff working with young homeless people: A grounded theory study.	-EXCLUDE on intervention (service engagement/helpseeking behaviour)	
####	Petersen (2013) (ID:87851185)	Redevelopment of tertiary psychiatric services in British Columbia: A prospective study of clinical, social, and residential outcomes of former long-stay inpatients.	-EXCLUDE on intervention (service engagement/helpseeking behaviour)	
####	Peterson (2023) (ID:87858001)	UNIVERSITY STUDENT FOOD INSECURITY AS A FORM OF STRUCTURAL VIOLENCE	-EXCLUDE on population (no housing precarity)	
####	Petra (2017) (ID:87851602)	Descriptions of health by EU citizens begging abroad	-EXCLUDE on intervention (service engagement/helpseeking behaviour)	
####	Phan (2023) (ID:87857644)	Examining civic engagement in ethnic minority youth populations: A literature review and concept analysis	-EXCLUDE on intervention (service engagement/helpseeking behaviour)	
####	Phelan (2021) (ID:87857572)	Understanding the social and emotional wellbeing of aboriginal LGBTIQ(SB)+ youth in Victoria's youth detention	-EXCLUDE on population (no housing precarity)	
####	Phillimore (2022) (ID:87857673)	"We are Forgotten": Forced Migration, Sexual and Gender-Based Violence, and Coronavirus Disease-2019	-EXCLUDE on intervention (service engagement/helpseeking behaviour)	
####	Phillips (2010) (ID:87851385)	"Toward a healthier tomorrow: Competent health and HIV care for transgender persons.": Erratum.	-EXCLUDE on evidence and form (evidence not in written form or presented as research output)	
####	Phillips (2014) (ID:87851079)	Inability to access addiction treatment among street-involved youth in a Canadian setting.	-EXCLUDE on population (people aged under 18 years)	
####	Phillips (2015) (ID:87850980)	High-intensity drug use and health service access among street-involved youth in a Canadian setting.	-EXCLUDE on intervention (service engagement/helpseeking behaviour)	
####	Phillips (2021) (ID:87848186)	Barriers to help-seeking among intimate partner violence survivors with opioid use disorder.	-INCLUDE on title & abstract	-EXCLUDE (IPV/DVA but little discussion on housing)
####	PHILLIPSON (2015) (ID:87857176)	Dementia attitudes and help-seeking intentions: an investigation of responses to two scenarios of an experience of the early signs of dementia	-EXCLUDE on population (no housing precarity)	
####	PHILLIPSON (2019) (ID:87857227)	Knowledge, help-seeking and efficacy to find respite services: an exploratory study in help-seeking carers of people with dementia in the context of aged care reforms	-EXCLUDE on intervention (service engagement/helpseeking behaviour)	
####	Phippen (2012) (ID:87852032)	Welcoming the homeless; Self-Help and Recovery Exchange homes work by creating a family culture	-EXCLUDE on evidence and form (evidence not in written form or presented as research output)	
####	Phipps (2019) (ID:87852953)	Women and homelessness, a complex multidimensional issue: findings from a scoping review	-INCLUDE on title & abstract	-EXCLUDE on intervention (intersectionality)<br>-EXCLUDE (systematic review)* only use with other code
####	Phipps (2021) (ID:87852803)	Combining Self-Determination Theory and Photo-Elicitation to Understand the Experiences of Homeless Women	-EXCLUDE on intervention (service engagement/helpseeking behaviour)<br>-EXCLUDE - but review for literature	
####	Phipps (2021) (ID:88019139)	A qualitative exploration of women's resilience in the face of homelessness	-INCLUDE on title & abstract	-EXCLUDE on intervention (intersectionality)
####	PICKARD (2011) (ID:87857320)	The relationship of social support to African American caregivers' help-seeking for emotional problems	-EXCLUDE on population (no housing precarity)	
####	Piehler (2014) (ID:87851119)	Executive functioning as a mediator of conduct problems prevention in children of homeless families residing in temporary supportive housing: A parallel process latent growth modeling approach.	-EXCLUDE on intervention (service engagement/helpseeking behaviour)	

####	Pinckney (2020) (ID:87851983)	Community-Based Counselors' Experiences Counseling Female Adjudicated Youth: A Basic Qualitative Study	-EXCLUDE on intervention (service engagement/helpseeking behaviour)		
####	Pinder (2022) (ID:87857899)	Understanding Fathering and Fatherhood of African American Adult Fathers Who Were Young Adult Fathers: A Retrospective Study	-EXCLUDE on population (women)		
####	Pindi (2015) (ID:87857858)	Performing Black feminisms in diasporic contexts: Sub-Saharan women negotiating identity across cultures	-EXCLUDE on intervention (service engagement/helpseeking behaviour)		
####	Pinto (2014) (ID:87848927)	Prevalence of syphilis and associated factors in homeless people of Sao Paulo, Brazil, using a Rapid Test.	-EXCLUDE on country (High-Income)		
####	Pinto (2016) (ID:87848723)	Cortisol awakening response among women exposed to intimate partner violence.	-EXCLUDE on intervention (service engagement/helpseeking behaviour)		
####	Pinto (2019) (ID:87848479)	Psychological Distress and Posttraumatic Stress Symptoms: The Role of Maternal Satisfaction, Parenting Stress, and Social Support Among Mothers and Children Exposed to Intimate Partner Violence.	-EXCLUDE on intervention (service engagement/helpseeking behaviour)		
####	Piske (2020) (ID:87848335)	The cascade of care for opioid use disorder: a retrospective study in British Columbia, Canada.	-EXCLUDE on intervention (service engagement/helpseeking behaviour)		
####	PITT (2000) (ID:87857111)	Seeking relief	-EXCLUDE on date (2010)		
####	Place (2022) (ID:87963080)	Toward a framework for listening with consideration for intersectionality: Insights from public relations professionals in borderland spaces	-EXCLUDE on intervention (service engagement/helpseeking behaviour)		
####	PLANEY (2019) (ID:87857229)	Barriers and facilitators to mental health help-seeking among African American youth and their families: a systematic review study	-EXCLUDE on population (no housing precarity)		
####	Planning community mental... (1996) (ID:87856929)	Planning community mental health services for women: a multiprofessional handbook	-EXCLUDE on date (2010)<br>-EXCLUDE - but review for literature		
####	Pleace (2016) (ID:88019120)	Exclusion by Definition: The Under-Representation of Women in European Homelessness Statistics	-EXCLUDE on intervention (service engagement/helpseeking behaviour)		
####	Pleace (2016) (ID:88019121)	Long-term and Recurrent Homelessness Among Women	-EXCLUDE on intervention (service engagement/helpseeking behaviour)<br>-EXCLUDE - but review for literature		
####	Pleace (2018) (ID:87851417)	Using Housing First in integrated homelessness strategies: a review of the evidence	-EXCLUDE on population (women)		
####	PLOEG (2008) (ID:87857019)	A case study of a Canadian homelessness intervention programme for elderly people	-EXCLUDE on date (2010)		
####	Plowman (2013) (ID:87851153)	Factors accounting for change in effective parenting following homelessness.	-EXCLUDE on intervention (service engagement/helpseeking behaviour)		
####	Podium abstract... (2023) (ID:87857920)	Podium abstract presentations	-EXCLUDE on evidence and form (evidence not in written form or presented as research output)		
####	Poleshuck (2021) (ID:87853088)	A Biopsychosocial and Interprofessional Approach to the Treatment of Family and Intimate Partner Violence: It Takes a Village	-EXCLUDE on intervention (service engagement/helpseeking behaviour)		
####	Polimis (2017) (ID:87849375)	Developing computational approaches to investigate health inequalities	-EXCLUDE on intervention (service engagement/helpseeking behaviour)		
####	Polvere (2013) (ID:87849019)	Participant perspectives on housing first and recovery: early findings from the At Home/Chez Soi project.	-EXCLUDE on population (women)		
####	Ponce (2014) (ID:87848956)	Homelessness, behavioral health disorders and intimate partner violence: barriers to services for women.	-INCLUDE on title & abstract	-EXCLUDE on intervention (intersectionality)	
####	Poncet (2020) (ID:87851704)	Dysménorrhée et expérience migratoire : caractéristiques associées à la dysménorrhée dans un échantillon de femmes migrantes hébergées à l'hôtel en Île-de-France. (French)	-EXCLUDE on intervention (service engagement/helpseeking behaviour)		
####	Poppleton (2022) (ID:87851877)	The perceptions of general practice among Central and Eastern Europeans in the United Kingdom: A systematic scoping review	-EXCLUDE on intervention (service engagement/helpseeking behaviour)		
####	PORCHLIGHT (2019) (ID:87856936)	Seeing the unseen: meeting the needs of women sleeping rough in Kent	-INCLUDE on title & abstract	-INCLUDE on full study	-EXCLUDED (Quality appraisal)
####	Poremski (2016) (ID:87848801)	Building trust with people receiving supported employment and housing first services.	-EXCLUDE on intervention (service engagement/helpseeking behaviour)		
####	Porter (2021) (ID:87963132)	Understanding influences of development on Black women's success in US colleges: A synthesis of literature	-EXCLUDE on population (no housing precarity)		

####	Portz (2020) (ID:87851646)	Advance Care Planning Among Users of a Patient Portal During the COVID-19 Pandemic: Retrospective Observational Study.	-EXCLUDE on population (no housing precarity)	
####	Poster Abstracts (2023) (ID:87857943)	Poster Abstracts	-EXCLUDE on evidence and form (evidence not in written form or presented as research output)	
####	POSTER... (2011) (ID:87851525)	POSTER PRESENTATIONS.	-EXCLUDE on evidence and form (evidence not in written form or presented as research output)	
####	Poteat (2020) (ID:87851541)	Vulnerability to COVID-19-related Harms Among Transgender Women With and Without HIV Infection in the Eastern and Southern U.S.	-EXCLUDE on intervention (service engagement/helpseeking behaviour)	
####	Potter (2016) (ID:87848799)	Support of vulnerable patients throughout TB treatment in the UK.	-EXCLUDE on population (women)	
####	Potts (2023) (ID:87858007)	Super, social, medical: Person-first and identity-first representations of disabled people in Australian newspapers, 2000–2019	-EXCLUDE on intervention (service engagement/helpseeking behaviour)	
####	Poulos (2010) (ID:87849237)	Vaccination against hepatitis A and B in persons subject to homelessness in inner Sydney: vaccine acceptance, completion rates and immunogenicity.	-EXCLUDE on intervention (service engagement/helpseeking behaviour)	
####	Powell (2016) (ID:87850926)	iTEAM: Outcomes of an affirming system of care serving LGBTQ youth experiencing homelessness.	-EXCLUDE on intervention (service engagement/helpseeking behaviour)	
####	Powell (2021) (ID:87850645)	Addressing risk factors among parents with serious mental illness: Commentary on Ostrow et al.	-EXCLUDE on evidence and form (evidence not in written form or presented as research output)	
####	POWELL (2021) (ID:87857128)	Mental health help seeking in young people and carers in out of home care: a systematic review	-EXCLUDE on population (no housing precarity)	
####	Pozo (2019) (ID:87858056)	Modifying the Relational Suicide Assessment for Use with Latinx LGB Adolescents	-EXCLUDE on population (people aged under 18 years)	
####	Prank (2023) (ID:87851582)	Rearing system, socio-economic status and common diseases frequency of goats in the Northern part of Bangladesh.	-EXCLUDE on country (High-Income)	
####	Pratt (2012) (ID:87849095)	Characteristics of adults with serious mental illness in the United States household population in 2007.	-EXCLUDE on intervention (service engagement/helpseeking behaviour)	
####	Pratt (2013) (ID:87851954)	Experience of Wellness Recovery Action Planning in Self-Help and Mutual Support Groups for People with Lived Experience of Mental Health Difficulties	-EXCLUDE on population (no housing precarity)	
####	Prayogo (2018) (ID:87848576)	Who uses foodbanks and why? Exploring the impact of financial strain and adverse life events on food insecurity.	-EXCLUDE on intervention (service engagement/helpseeking behaviour)	
####	Preis (2020) (ID:87857653)	Contributions of Psychology to Research, Treatment, and Care of Pregnant Women With Opioid Use Disorder	-EXCLUDE on population (no housing precarity)	
####	Premkumar (2020) (ID:87848293)	Home Birth in the Era of COVID-19: Counseling and Preparation for Pregnant Persons Living with HIV.	-EXCLUDE on population (no housing precarity)	
####	Presentation... (2012) (ID:87858025)	Presentation Abstracts	-EXCLUDE on evidence and form (evidence not in written form or presented as research output)	
####	Priebe (2013) (ID:87849069)	Mental health-care provision for marginalized groups across Europe: findings from the PROMO study.	-EXCLUDE on population (no gender focus; women population <50)	
####	Priester (2017) (ID:87853080)	Are Discrimination and Social Capital Related to Housing Instability?	-EXCLUDE on population (women)	
####	Prior (2023) (ID:87963386)	Help-seeking and help-related experiences of commercially sexually exploited youth: a qualitative meta-synthesis	-INCLUDE on title & abstract	-EXCLUDE on intervention (intersectionality)<br>-EXCLUDE (systematic review)* only use with other code
####	Prock (2017) (ID:87850858)	Federally-funded transitional living programs and services for LGBTQ-identified homeless youth: A profile in unmet need.	-EXCLUDE on intervention (service engagement/helpseeking behaviour)	
####	Prock (2019) (ID:87850751)	The relationship between LGBTQ identity, service utilization, and mental health and substance use impairment over time among homeless youth.	-EXCLUDE on intervention (service engagement/helpseeking behaviour)	
####	Prock (2020) (ID:87850702)	Characteristics, experiences, and service utilization patterns of homeless youth in a transitional living program: Differences by LGBQ identity.	-INCLUDE on title & abstract	-EXCLUDE on intervention (service engagement/helpseeking behaviour)
####	Proehl (2007) (ID:87853693)	Social Justice, Respect, and Meaning-Making: Keys to Working with the Homeless Elderly Population	-EXCLUDE on date (2010)	

####	Pryce (2017) (ID:87963337)	Transition to adulthood of former foster youth: Multilevel challenges to the help-seeking process	-EXCLUDE on intervention (service engagement/helpseeking behaviour)	
####	Psychiatric Risk in... (Flentje) (ID:87851479)	Psychiatric Risk in Unstably Housed Sexual Minority Women: Relationship between Sexual and Racial Minority Status and Human Immunodeficiency Virus and Psychiatric Diagnoses	-EXCLUDE on intervention (service engagement/helpseeking behaviour)	
####	Ptacek (2021) (ID:87857671)	Hidden Dramas of Masculinity: Women's Perspectives on Intimate Violence in Different Social Classes	-EXCLUDE on intervention (service engagement/helpseeking behaviour)	
####	PUBLIC (2018) (ID:87857098)	Improving the health and wellbeing of lesbian and bisexual women and other women who have sex with women	-EXCLUDE on intervention (service engagement/helpseeking behaviour)	
####	Pullen (2014) (ID:87963373)	Social networks, drug use, and drug abuse help-seeking: a test of the network episode model among African American women	-EXCLUDE on population (no housing precarity)<br>-EXCLUDE - but review for literature	
####	Purba (2023) (ID:87848129)	An Exploration of Family Caregivers' Health Care Needs When Caring for Patients With Cancer in the Resource-Challenged Context of West Java, Indonesia.	-EXCLUDE on country (High-Income)	
####	Purkey (2019) (ID:87848374)	Experiences of Palliative Health Care for Homeless and Vulnerably Housed Individuals.	-EXCLUDE on population (women)	
####	Purkey (2020) (ID:87852749)	Experience of emergency department use among persons with a history of adverse childhood experiences	-EXCLUDE on population (no housing precarity)<br>-EXCLUDE - but review for literature	
####	Putt (2017) (ID:87963126)	Women's specialist domestic and family violence services: Their responses and practices with and for Aboriginal women	-INCLUDE on title & abstract	-EXCLUDE on intervention (too programme-specific; not generalisable)
####	PYBUS (2004) (ID:87857107)	Welcome to the UK	-EXCLUDE on date (2010)	
####	PYNE (2011) (ID:87857231)	Unsuitable bodies: trans people and cisnormativity in shelter services	-INCLUDE on title & abstract	-EXCLUDE on evidence and form (evidence not in written form or presented as research output)
####	Quality Relationships,... (Burns) (ID:87849367)	Quality Relationships, Not Quantity	-INCLUDE on title & abstract	-EXCLUDE on date (2010)
####	Queiroz (2022) (ID:87851544)	A população em situação de rua: As estratégias de planejamento intersetorial para o cuidado em saúde.	-EXCLUDE on intervention (service engagement/helpseeking behaviour)	
####	Quezada (2021) (ID:87858048)	Ponte Las Pilas: First-Generation Latina College Students Navigating Higher Education with Parental Encouragement	-EXCLUDE on population (no housing precarity)	
####	QUILGARS (2018) (ID:87856960)	The threshold Housing First pilot for women with an offending history: the first two years	-EXCLUDE on intervention (service engagement/helpseeking behaviour)<br>-EXCLUDE - but review for literature	
####	Quilty (2019) (ID:87848375)	Addressing Profound Disadvantages to Improve Indigenous Health and Reduce Hospitalisation: A Collaborative Community Program in Remote Northern Territory.	-EXCLUDE on intervention (service engagement/helpseeking behaviour)	
####	Quilty (2021) (ID:87857684)	Benefits of digital health resources for substance use concerns in women: Scoping review	-EXCLUDE on intervention (service engagement/helpseeking behaviour)	
####	**Quinn (2015) (ID:87852764)**	**The Role of Supportive Housing for HIV-Positive Mothers and Their Children**	**-INCLUDE on title & abstract**	**-INCLUDE on full study**
####	Quinn (2020) (ID:87850712)	Investigating possible syndemic relationships between structural and drug use factors, sexual HIV transmission and viral load among men of colour who have sex with men in Los Angeles County.	-EXCLUDE on population (women)	
####	Quinton (2021) (ID:87963272)	An evaluation of My Strengths Training for Life™ for improving resilience and well-being of young people experiencing homelessness	-EXCLUDE on intervention (service engagement/helpseeking behaviour)	
####	Qureshi (2021) (ID:87848220)	Establishing an Epidemiologic Profile of Hepatitis C Virus Infection at the Los Angeles County Jail.	-EXCLUDE on intervention (service engagement/helpseeking behaviour)	
####	R (2019) (ID:87848453)	Context for layering women's nutrition interventions on a large scale poverty alleviation program: Evidence from three eastern Indian states.	-EXCLUDE on country (High-Income)	
####	RACIAL-ETHNIC COLORECTAL... (Wassira) (ID:87851779)	RACIAL-ETHNIC COLORECTAL CANCER SURVIVAL DISPARITIES IN THE MOUNTAIN WEST REGION: THE CASE OF BLACKS COMPARED TO WHITES.	-EXCLUDE on population (no housing precarity)	
####	Rada (2022) (ID:87852859)	Forced Migration and Reproductive Rights: Pregnant Women Fleeing Venezuela	-EXCLUDE on country (High-Income)	
####	Radkiewicz (2020) (ID:87851635)	Sex Differences in Urothelial Bladder Cancer Survival	-EXCLUDE on population (no housing precarity)	
####	Radó (2022) (ID:87849339)	Digital Technology Access and Health-Related Internet Use Among People Experiencing Homelessness in Hungary: Quantitative Survey	-EXCLUDE on intervention (service engagement/helpseeking behaviour)	

####	Raedel (2020) (ID:87850674)	Clergy attitudes about ways to support the mental health of sexual and gender minorities.	-EXCLUDE on intervention (service engagement/helpseeking behaviour)
####	Rafael (2018) (ID:87848497)	Disparities in follow-up care for ballistic and non-ballistic long bone lower extremity fractures.	-EXCLUDE on intervention (service engagement/helpseeking behaviour)
####	Ragavan (2018) (ID:87857946)	The Influence of Healthy Relationship Formation and Teen Dating Violence: A Qualitative Analysis of South Asian Youth Residing in the United States	-EXCLUDE on intervention (service engagement/helpseeking behaviour)
####	RAGHALLAIGH (2011) (ID:87857345)	Religion in the lives of unaccompanied minors: an available and compelling coping resource	-EXCLUDE on population (people aged under 18 years)
####	Rahman (2022) (ID:87853104)	Rural-urban differentials in the influences of individual and geospatial preparedness on institutional childbirth: a cross-sectional study in Bangladesh	-EXCLUDE on country (High-Income)
####	Rai (2018) (ID:87963105)	Socio-cultural risk factors impacting domestic violence among South Asian immigrant women: A scoping review	-EXCLUDE on intervention (service engagement/helpseeking behaviour)<br>-EXCLUDE - but review for literature
####	Raiford (2016) (ID:87848746)	The Role of Structural Barriers in Risky Sexual Behavior, Victimization and Readiness to Change HIV/STI-Related Risk Behavior Among Transgender Women.	-EXCLUDE on intervention (service engagement/helpseeking behaviour)
####	Raines (2015) (ID:87857690)	Mental health treatment seeking disparities among African American women	-EXCLUDE on population (no housing precarity)
####	Raj (2014) (ID:87848987)	Pilot evaluation of the Making Employment Needs [MEN] count intervention: addressing behavioral and structural HIV risks in heterosexual black men.	-EXCLUDE on intervention (service engagement/helpseeking behaviour)
####	RAJABIUN (2011) (ID:87857334)	Keeping at-risk persons living with HIV/AIDS in care: a qualitative study of staff perspectives	-EXCLUDE on population (no gender focus; women population <50)
####	RAMAKERS (2009) (ID:87857381)	Characteristics of help-seeking behaviour in subjects with subjective memory complaints at a memory clinic: a case-control study	-EXCLUDE on date (2010)
####	Rambarran (2013) (ID:87849075)	Relocating from out-of-area treatments: service users' perspective.	-EXCLUDE on intervention (service engagement/helpseeking behaviour)
####	Rambliere (2023) (ID:87853086)	Major depressive disorder in post-secondary students attending foodbanks in France	-EXCLUDE on intervention (service engagement/helpseeking behaviour)
####	Ramon (2010) (ID:87849276)	Companion animal knowledge, attachment and pet cat care and their associations with household demographics for residents of a rural Texas town.	-EXCLUDE on population (women)
####	Ramos (2015) (ID:87848831)	Navigator Approach to Improve Quality of Care for Vulnerable Populations in Mexico.	-EXCLUDE on country (High-Income)
####	Ramos (2018) (ID:87850840)	Worthy of care? Medical inclusion from the Watts Riots to the building of King-Drew, Prisons, and Skid Row, 1965-1986.	-EXCLUDE on intervention (service engagement/helpseeking behaviour)
####	Ramos (2018) (ID:87857845)	Out of the Margins: Experiences of Low-Income, Single Mothers of Color in Community College	-EXCLUDE on population (no housing precarity)
####	Ramos-Ochoa (2022) (ID:87858020)	Gang of Brothers–Hermandad: The Construction of a Humanized and Healing Space for Latino and Black Males through Community, Culture and Educational Empowerment	-EXCLUDE on population (women)
####	Ramsay (2019) (ID:87848438)	Health Care While Homeless: Barriers, Facilitators, and the Lived Experiences of Homeless Individuals Accessing Health Care in a Canadian Regional Municipality.	-EXCLUDE on population (women)
####	Ramsundarsingh (2023) (ID:87857541)	Interpersonal or Institutional: Understanding Service User Oppression in Social Service Organizations Through Staff Interactions	-EXCLUDE on intervention (service engagement/helpseeking behaviour)
####	Ran (2015) (ID:87848867)	Gender differences in outcomes in people with schizophrenia in rural China: 14-year follow-up study.	-EXCLUDE on country (High-Income)
####	Rana (2016) (ID:87850536)	Changes following adversities : the role of religious coping in the lives of homeless women of Vrindavan (India)	-EXCLUDE on country (High-Income)
####	Ransdell (2021) (ID:87857909)	Mentoring new and early-stage investigators and underrepresented minority faculty for research success in health-related fields: An integrative literature review (2010-2020)	-EXCLUDE on population (no housing precarity)
####	RANZATO (2021) (ID:87857249)	A qualitative analysis of goals set by foster carers seeking support for their child’s emotional well-being	-EXCLUDE on population (no housing precarity)

####	Rao (2015) (ID:87857733)	Expanding Opportunities for Diversity in Positive Psychology: An Examination of Gender, Race, and Ethnicity	-EXCLUDE on intervention (service engagement/helpseeking behaviour)
####	Raschig (2023) (ID:87857489)	Cargas Coming down: Chronic stress, Chicana-Indigenous spiritual healing, and feminist fugitive potentiality	-EXCLUDE on intervention (service engagement/helpseeking behaviour)
####	RASHLEIGH (2007) (ID:87857014)	Fobbed off	-EXCLUDE on date (2010)
####	Ratajczak (2023) (ID:87857924)	Queering Title IX: Protecting Transgender and Gender Non-Conforming Students from Discrimination, Harassment, and Violence	-EXCLUDE on population (no housing precarity)
####	Ratliff (2022) (ID:87850605)	A social-ecological history of gender and violence in the lives of transgender and nonbinary young people.	-EXCLUDE on intervention (service engagement/helpseeking behaviour)<br>-EXCLUDE - but review for literature
####	Raven (2020) (ID:87848260)	A randomized trial of permanent supportive housing for chronically homeless persons with high use of publicly funded services.	-EXCLUDE on intervention (service engagement/helpseeking behaviour)
####	Ravenhill (2016) (ID:87849292)	The Culture of Homelessness	-EXCLUDE on intervention (service engagement/helpseeking behaviour)
####	Ravi (2022) (ID:87852840)	Facilitators of Formal Help-Seeking for Adult Survivors of IPV in the United States: A Systematic Review	-EXCLUDE on population (no housing precarity)
####	RAVIV (2000) (ID:87857134)	Adolescents' help-seeking behaviour: the difference between self- and other referral	-EXCLUDE on date (2010)
####	RAVIV (2009) (ID:87857122)	The personal service gap: factors affecting adolescents' willingness to seek help	-EXCLUDE on date (2010)
####	Ray (2016) (ID:87853164)	Prognosis after maternal placental events and revascularization: PAMPER study	-EXCLUDE on population (no housing precarity)
####	Ray (2023) (ID:87857576)	Conceptualizing Task Force Sustainability	-EXCLUDE on intervention (service engagement/helpseeking behaviour)
####	Rayment-Jones (2021) (ID:87848197)	Project20: Does continuity of care and community-based antenatal care improve maternal and neonatal birth outcomes for women with social risk factors? A prospective, observational study.	-EXCLUDE on intervention (service engagement/helpseeking behaviour)
####	Rayment-Jones (2019) (ID:87857615)	How do women with social risk factors experience United Kingdom maternity care? A realist synthesis	-EXCLUDE on population (no housing precarity)
####	Raynor (2021) (ID:87852713)	Implementing Prevention Plus with Underserved Families in an Integrated Primary Care Setting	-EXCLUDE on intervention (service engagement/helpseeking behaviour)
####	Razeq (2017) (ID:87851640)	The incidence, risk factors, and mortality of preterm neonates: A prospective study from Jordan (2012-2013).	-EXCLUDE on country (High-Income)
####	Read (2017) (ID:87848633)	Delivering direct acting antiviral therapy for hepatitis C to highly marginalised and current drug injecting populations in a targeted primary health care setting.	-EXCLUDE on intervention (service engagement/helpseeking behaviour)
####	Read (2019) (ID:87848402)	Treatment adherence and support for people who inject drugs taking direct-acting antiviral therapy for hepatitis C infection.	-EXCLUDE on intervention (service engagement/helpseeking behaviour)
####	Reavey (2010) (ID:87858029)	Spatial markings: Memory, agency and child sexual abuse	-EXCLUDE on population (people aged under 18 years)
####	Reback (2018) (ID:87848527)	Health Disparities, Risk Behaviors and Healthcare Utilization Among Transgender Women in Los Angeles County: A Comparison from 1998-1999 to 2015-2016.	-EXCLUDE on intervention (service engagement/helpseeking behaviour)
####	Reback (2019) (ID:87853290)	Text Messaging to Improve Linkage, Retention, and Health Outcomes Among HIV-Positive Young Transgender Women: Protocol for a Randomized Controlled Trial (Text Me, Girl!)	-EXCLUDE on population (no housing precarity)
####	Reback (2020) (ID:87853265)	Technology-Based Stepped Care to Stem Transgender Adolescent Risk Transmission: Protocol for a Randomized Controlled Trial (TechStep)	-EXCLUDE on intervention (service engagement/helpseeking behaviour)
####	Recent trends in the... (Reda) (ID:87851631)	Recent trends in the timing of first sex and marriage among young women in Ethiopia.	-EXCLUDE on country (High-Income)
####	Redmond (2020) (ID:87857589)	Exploring African-American womens' experiences with substance use treatment: A review of the literature	-EXCLUDE on population (no housing precarity)
####	REED (1993) (ID:87857291)	Review of health and social services for mentally disordered offenders and others requiring similar services: volume 5; special issues and differing needs; the report of the official working group on services for people with special needs	-EXCLUDE on date (2010)<br>-EXCLUDE - but review for literature

####	Reed (2017) (ID:87848660)	What Drives Country Differences in Cost of Alzheimer's Disease? An Explanation from Resource Use in the GERAS Study.	-EXCLUDE on population (no housing precarity)	
####	Reed (2020) (ID:87857892)	Risk and Protective Factor specific to African American Youth and Adolescents: A Systematic Review	-EXCLUDE on intervention (service engagement/helpseeking behaviour)	
####	Reed (2020) (ID:87858043)	The Perceived Impact of Austerity on Young People of Colour's (Ypoc) Psychological Wellbeing and Education in the Uk	-EXCLUDE on population (no housing precarity)	
####	REEDTZ (2011) (ID:87857343)	Parents seeking help in child rearing: who are they and how do their children behave?	-EXCLUDE on population (no housing precarity)	
####	REES (2009) (ID:87856984)	Mental ill health in the adult single homeless population: a review of the literature	-EXCLUDE on date (2010)	
####	REEVE (2006) (ID:87856927)	Homeless women: still being failed yet striving to survive: summary	-EXCLUDE on date (2010)	
####	REEVE (2020) (ID:87856995)	Forgotten mothers: the case for a policy focus on the experiences of motherhood and homelessness	-INCLUDE on title & abstract	-EXCLUDE on evidence and form (not empirical)
####	Reeves (2019) (ID:87851996)	Compassion Fatigue: Stories/Artworks of an Art Teacher with a Trauma-Informed Pedagogy	-EXCLUDE on intervention (service engagement/helpseeking behaviour)	
####	Reeves (2021) (ID:87963085)	Marginalized women, domestic and family violence reforms and their unintended consequences	-EXCLUDE on intervention (service engagement/helpseeking behaviour)<br>-EXCLUDE - but review for literature	
####	Refaeli (2019) (ID:87848481)	Personal and Environmental Predictors of Depression Among Victims of Intimate Partner Violence: Comparison of Immigrant and Israeli-Born Women.	-EXCLUDE on intervention (service engagement/helpseeking behaviour)	
####	Reid (2020) (ID:87848309)	Mechanisms of change and participant outcomes in a Recovery Education Centre for individuals transitioning from homelessness: a qualitative evaluation.	-EXCLUDE on population (women)	
####	Reilly (2004) (ID:87853702)	Can a Health Advocate for Homeless Families Reduce Workload for the Primary Healthcare Team? A Controlled Trial	-EXCLUDE on date (2010)	
####	Reilly (2019) (ID:87848461)	Characteristics of Mothers and Infants Living in Homeless Shelters and Public Housing in New York City.	-EXCLUDE on intervention (service engagement/helpseeking behaviour)	
####	Reist (2018) (ID:87857922)	'Christ the Redeemer Turns His Back on Us:' Urban Black Struggle in Rio's Baixada Fluminense	-EXCLUDE on country (High-Income)	
####	Reitmanova (2012) (ID:87851218)	Rethinking immigrant tuberculosis control in Canada: From medical surveillance to tackling social determinants of health.	-EXCLUDE on intervention (service engagement/helpseeking behaviour)	
####	Reitzel (2014) (ID:87848920)	Is smoking cessation associated with worse comorbid substance use outcomes among homeless adults?.	-EXCLUDE on intervention (service engagement/helpseeking behaviour)	
####	Renwick (2017) (ID:87848610)	Predictors of change in social networks, support and satisfaction following a first episode psychosis: A cohort study.	-EXCLUDE on intervention (service engagement/helpseeking behaviour)	
####	Renzetti (2013) (ID:87851148)	Editor's introduction.	-EXCLUDE on evidence and form (evidence not in written form or presented as research output)	
####	Researchers from University... (2010) (ID:87851780)	Researchers from University of Malmo discuss findings in family practice	-EXCLUDE on evidence and form (evidence not in written form or presented as research output)	
####	Resnick (2010) (ID:87849266)	Who attends Vet-to-Vet? Predictors of attendance in mental health mutual support.	-EXCLUDE on population (no housing precarity)	
####	Resnik (2017) (ID:87848690)	Which Homeless Veterans Benefit From a Peer Mentor and How?.	-EXCLUDE on intervention (service engagement/helpseeking behaviour)	
####	Retention in HIV care:... (Giordano) (ID:87851859)	Retention in HIV care: what the clinician needs to know.	-EXCLUDE on population (no housing precarity)	
####	Returned and Services... (2019) (ID:87851985)	Returned and Services League (Queensland Branch): ESO Corner - Kookaburra Kids	-EXCLUDE on population (people aged under 18 years)	
####	Reynish (2021) (ID:87857765)	Barriers and Enablers to Sex Workers' Uptake of Mental Healthcare: a Systematic Literature Review	-INCLUDE on title & abstract	-EXCLUDE on intervention (intersectionality)<br>-EXCLUDE (systematic review)* only use with other code
####	Reynoso (2022) (ID:87850584)	Description of audiologic, developmental, ophthalmologic, and neurologic diagnoses at a primary care clinic for children in foster care.	-EXCLUDE on population (people aged under 18 years)	
####	Rezansoff (2017) (ID:87848692)	Housing First Improves Adherence to Antipsychotic Medication Among Formerly Homeless Adults With Schizophrenia: Results of a Randomized Controlled Trial.	-EXCLUDE on intervention (service engagement/helpseeking behaviour)	

####	Rezazadeh (2018) (ID:87963203)	Women's experiences of immigration to Canada: A review of the literature.	-EXCLUDE on intervention (service engagement/helpseeking behaviour)
####	Rhead (2022) (ID:87857461)	A comparison of single and intersectional social identities associated with discrimination and mental health service use: data from the 2014 Adult Psychiatric Morbidity Survey in England	-EXCLUDE on population (no housing precarity)
####	Rhoades (2014) (ID:87848952)	Prescription drug misuse among homeless youth.	-EXCLUDE on intervention (service engagement/helpseeking behaviour)
####	Rhoades (2018) (ID:87848510)	Social networks and substance use after transitioning into permanentsupportive housing.	-EXCLUDE on population (women)
####	Rhoades (2019) (ID:87848413)	Changes in Self-Rated Physical Health After Moving Into Permanent Supportive Housing.	-EXCLUDE on intervention (service engagement/helpseeking behaviour)
####	Rice (2010) (ID:87849240)	Internet use, social networking, and HIV/AIDS risk for homeless adolescents.	-EXCLUDE on intervention (service engagement/helpseeking behaviour)
####	Rice (2010) (ID:87849248)	The positive role of social networks and social networking technology in the condom-using behaviors of homeless young people.	-EXCLUDE on population (women)
####	Rice (2011) (ID:87849163)	Cell phone use among homeless youth: potential for new health interventions and research.	-EXCLUDE on intervention (service engagement/helpseeking behaviour)
####	Rice (2011) (ID:87849219)	Social networking technology, social network composition, and reductions in substance use among homeless adolescents.	-EXCLUDE on population (women)
####	Rice (2012) (ID:87849135)	Mobilizing homeless youth for HIV prevention: a social network analysis of the acceptability of a face-to-face and online social networking intervention.	-EXCLUDE on intervention (service engagement/helpseeking behaviour)
####	Rice (2012) (ID:87849141)	Position-specific HIV risk in a large network of homeless youths.	-EXCLUDE on population (no gender focus; women population <50)
####	Rice (2021) (ID:87853108)	A Peer-Led, Artificial Intelligence-Augmented Social Network Intervention to Prevent HIV Among Youth Experiencing Homelessness	-EXCLUDE on intervention (service engagement/helpseeking behaviour)
####	Richard (2019) (ID:87848365)	Validation study of health administrative data algorithms to identify individuals experiencing homelessness and estimate population prevalence of homelessness in Ontario, Canada.	-EXCLUDE on intervention (service engagement/helpseeking behaviour)
####	Richards (2011) (ID:87849225)	Maternal health behaviors and infant health outcomes among homeless mothers: U.S. Special Supplemental Nutrition Program for Women, Infants, and Children (WIC) 2000-2007.	-EXCLUDE on intervention (service engagement/helpseeking behaviour)
####	Richards (2015) (ID:87848884)	Retention of Homeless Smokers in the Power to Quit Study.	-EXCLUDE on intervention (service engagement/helpseeking behaviour)
####	Richards (2021) (ID:87851894)	Examining Homeless Trajectories and Health Outcomes Among Young Adults in Los Angeles County	-EXCLUDE on population (no gender focus; women population <50)
####	Richards (2022) (ID:87850603)	Examining homeless trajectories and health outcomes among young adults in Los Angeles County.	-EXCLUDE Duplicate
####	Richardson (2021) (ID:87849355)	Drug-related harm coinciding with income assistance payments: results from a community-based cohort of people who use drugs	-EXCLUDE on intervention (service engagement/helpseeking behaviour)
####	Ricks (2022) (ID:87857993)	A Systematic Literature Review of Community-Based Participatory Health Research with Sexual and Gender Minority Communities	-EXCLUDE on intervention (service engagement/helpseeking behaviour)
####	Ridlen (1990) (ID:87853712)	Outreach and Engagement for Homeless Women at Risk of Alcoholism	-EXCLUDE on date (2010)
####	Rieke (2015) (ID:87848829)	Mental and nonmental health hospital admissions among chronically homeless adults before and after supportive housing placement.	-EXCLUDE on intervention (service engagement/helpseeking behaviour)
####	Riley (2010) (ID:87849254)	Access to sterile syringes through San Francisco pharmacies and the association with HIV risk behavior among injection drug users.	-EXCLUDE on population (women)
####	Riley (2011) (ID:87849180)	Basic subsistence needs and overall health among human immunodeficiency virus-infected homeless and unstably housed women.	-EXCLUDE on intervention (service engagement/helpseeking behaviour)

####	Riley (2011) (ID:87849205)	Population-level effects of uninterrupted health insurance on services use among HIV-positive unstably housed adults.	-EXCLUDE on intervention (service engagement/helpseeking behaviour)	
####	Riley (2014) (ID:87848921)	Recent violence in a community-based sample of homeless and unstably housed women with high levels of psychiatric comorbidity.	-EXCLUDE on intervention (service engagement/helpseeking behaviour)	
####	Riley (2017) (ID:87963210)	Empowering justice: An intersectional feminist perspective on restorative justice in the sex trade	-EXCLUDE on intervention (service engagement/helpseeking behaviour)	
####	Riley (2019) (ID:87852933)	Housing First: Unsuppressed Viral Load Among Women Living with HIV in San Francisco	-EXCLUDE on intervention (service engagement/helpseeking behaviour)	
####	Riordan (2022) (ID:87857761)	Seeking justice beyond the platform economy: migrant workers navigating precarious lives	-EXCLUDE on intervention (service engagement/helpseeking behaviour)	
####	Rishworth (2022) (ID:87857658)	Global Discourses and Local Disconnects: Gender, Aging, Health, and Well-Being in Uganda	-EXCLUDE on country (High-Income)	
####	RITCHIE (2003) (ID:87857250)	Children cut adrift	-EXCLUDE on date (2010)	
####	Ritchie (2015) (ID:87851944)	Prevent rough sleeping; create a psychologically informed environment	-EXCLUDE on intervention (service engagement/helpseeking behaviour)	
####	Rivas (2022) (ID:87858017)	Science as White Property: BIPOC Elementary Teachers' Science Experience and Its Impact on Their Pedagogy	-EXCLUDE on population (no housing precarity)	
####	Rivera (2018) (ID:87848499)	A modeling study exploring the impact of homelessness on rostered primary care utilization in Calgary, Canada.	-EXCLUDE on intervention (service engagement/helpseeking behaviour)	
####	Rivera-Rivera (2020) (ID:87850676)	Caribbean homeless women veterans: Military and psychosocial characteristics and the association of service-connected disabilities.	-EXCLUDE on intervention (service engagement/helpseeking behaviour)	
####	Rizzo (2022) (ID:87849329)	Barriers to Accessing Addiction Treatment for Women at Risk of Homelessness	-INCLUDE on title & abstract	-EXCLUDE on intervention (intersectionality)
####	Robards (2017) (ID:87851933)	CULTIVATING CONNECTIONS WITH MARGINALIZED YOUNG PEOPLE THOUGH HEALTH LITERACY: A SYSTEMATIC REVIEW	-EXCLUDE on intervention (service engagement/helpseeking behaviour)	
####	Robards (2018) (ID:87848558)	How Marginalized Young People Access, Engage With, and Navigate Health-Care Systems in the Digital Age: Systematic Review.	-EXCLUDE on population (no gender focus; women population <50)<br>-EXCLUDE - but review for literature	
####	Robards (2019) (ID:87857417)	Health care equity and access for marginalised young people: A longitudinal qualitative study exploring health system navigation in Australia	-EXCLUDE on population (people aged under 18 years)	
####	Robards (2020) (ID:87848255)	Intersectionality: Social Marginalisation and Self-Reported Health Status in Young People.	-EXCLUDE on population (people aged under 18 years)	
####	Robbins (2010) (ID:87849242)	Health and oral health care needs and health care-seeking behavior among homeless injection drug users in San Francisco.	-EXCLUDE on population (women)	
####	Robelski (2020) (ID:87848331)	(Un)bounded Social Work?-Analysis of Working Conditions in Refugee and Homeless Aid in Relation to Perceived Job Stress and Job Satisfaction.	-EXCLUDE on intervention (service engagement/helpseeking behaviour)	
####	Roberts (1990) (ID:87849376)	Substance Abuse Issues in Legal Services Practice: An Overview	-EXCLUDE on date (2010)	
####	ROBERTS (2002) (ID:87857369)	Disabled people in refugee and asylum-seeking communities	-EXCLUDE on date (2010)	
####	Roberts (2023) (ID:87963046)	An Exploration of Mental Health Help-Seeking Experiences Among Women Combat Veterans	-EXCLUDE on population (no housing precarity)	
####	Robertson (2015) (ID:87963269)	Evidence for a gender-based approach to mental health programmes: Identifying the key considerations associated with "being male"	-EXCLUDE on population (women)	
####	ROBINSON (2012) (ID:87857368)	Seeking respite: issues around the use of day respite care for the carers of people with dementia	-EXCLUDE on population (no housing precarity)	
####	ROBINSON (2018) (ID:87857112)	Immersion learning in social work education: a pedagogical tool for enriching knowledge and practice skills among BSW Students	-EXCLUDE on population (women)	
####	Robinson (2021) (ID:87853140)	"They peed on my shoes": foregrounding intersectional minority stress in understanding LGBTQ youth homelessness	-EXCLUDE on intervention (service engagement/helpseeking behaviour)	
####	Robinson (2021) (ID:87963215)	Disability and family violence prevention: a case study on participation in evidence making	-INCLUDE on title & abstract	-EXCLUDE on target group (no housing precarity)
####	Robinson (2023) (ID:87853138)	Housing instability: Exploring socioecological influences on the health of birthing people	-EXCLUDE on intervention (service engagement/helpseeking behaviour)	

####	Robson (2018) (ID:87858110)	Raising Awareness and Addressing Elder Abuse in the LGBT Community: An Intergenerational Arts Project	-EXCLUDE on population (no housing precarity)	
####	Robst (2011) (ID:87851312)	Factors related to criminal justice expenditure trajectories for adults with serious mental illness.	-EXCLUDE on population (women)	
####	Rocha-Jimenez (2022) (ID:87850579)	Intercepted journeys: Associations between migration and mobility experiences and depressive symptoms among substance using migrants at the Mexico-Guatemala border.	-EXCLUDE on country (High-Income)	
####	Roche (2020) (ID:87963127)	Valuing All Voices: refining a trauma-informed, intersectional and critical reflexive framework for patient engagement in health research using a qualitative descriptive approach	-EXCLUDE on population (no housing precarity)	
####	Rockett (2022) (ID:87848166)	Association of State Social and Environmental Factors With Rates of Self-injury Mortality and Suicide in the United States.	-EXCLUDE on intervention (service engagement/helpseeking behaviour)	
####	RODDY (2018) (ID:87857279)	Couples with intimate partner violence seeking relationship help: associations and implications for self-help and online interventions	-EXCLUDE on intervention (service engagement/helpseeking behaviour)	
####	Rodriguez (2012) (ID:87849138)	Prevalence of hoarding disorder in individuals at potential risk of eviction in New York City: a pilot study.	-EXCLUDE on intervention (service engagement/helpseeking behaviour)	
####	Rodriguez (2014) (ID:87851061)	Mentorship and foster care status: Understanding the transition to independent living.	-EXCLUDE on intervention (service engagement/helpseeking behaviour)	
####	Rodriguez (2014) (ID:87852021)	A psychoeducational support group for Hispanic adults with major depressive disorder due to trauma: A grant proposal (Los Angeles, California).	-EXCLUDE on evidence and form (evidence not in written form or presented as research output)	
####	Rodriguez-Moreno (2020) (ID:87848302)	Risk of mental ill-health among homeless women in Madrid (Spain).	-EXCLUDE on intervention (service engagement/helpseeking behaviour)	
####	Rodriguez-Moreno (2021) (ID:87848235)	The Role of Stressful Life Events among Women Experiencing Homelessness: An Intragroup Analysis.	-INCLUDE on title & abstract	-EXCLUDE on intervention (intersectionality)
####	Rodriguez-Moreno (2022) (ID:87850576)	Initial effectiveness evaluation of the Unified Protocol for Transdiagnostic Treatment of Emotional Disorders for Homeless Women.	-EXCLUDE on intervention (service engagement/helpseeking behaviour)	
####	Rodriguez-Moreno (2023) (ID:87850570)	Mediators and moderators of therapeutic change in the unified protocol for women experiencing homelessness.	-INCLUDE on title & abstract	-EXCLUDE on intervention (service engagement/helpseeking behaviour)
####	Rodriguez-Roldan (2021) (ID:87850609)	Like a candle flickering in the mist: Violence against the trans community.	-EXCLUDE on intervention (service engagement/helpseeking behaviour)	
####	Roesch-Knapp (2020) (ID:87858083)	The cyclical nature of poverty: Evicting the poor	-EXCLUDE on intervention (service engagement/helpseeking behaviour)	
####	Rogal (2017) (ID:87848637)	Primary Care and Hepatology Provider-Perceived Barriers to and Facilitators of Hepatitis C Treatment Candidacy and Adherence.	-EXCLUDE on intervention (service engagement/helpseeking behaviour)	
####	Rogers (2011) (ID:87857836)	Immigrant women's narrative reconstruction of their interactions with their children's schools: A collective qualitative case study	-EXCLUDE on population (no housing precarity)	
####	Rogers (2017) (ID:87848655)	Long-term abstinence and predictors of tobacco treatment uptake among hospitalized smokers with serious mental illness enrolled in a smoking cessation trial.	-EXCLUDE on intervention (service engagement/helpseeking behaviour)	
####	Rogers (2018) (ID:87857407)	Mutual benefits: The lessons learned from a community based participatory research project with unaccompanied asylum-seeking children and foster carers	-EXCLUDE on population (people aged under 18 years)	
####	Rogers (2019) (ID:87857488)	Social Services Professionals' Views of Barriers to Supporting Homeless Noncustodial Fathers	-EXCLUDE on population (women)	
####	Rogers (2021) (ID:87850639)	Trans men's pathways to incarceration.	-EXCLUDE on population (women)	
####	Rogers (2023) (ID:87850561)	A call for resources and protection: Trans lives in the Southeastern United States.	-EXCLUDE on intervention (service engagement/helpseeking behaviour)	
####	Roig-Palmer (2022) (ID:87853032)	Confronting Oppression: Reframing Need and Advancing Responsivity for LGBTQ+ Youth and Young Adults	-EXCLUDE on intervention (service engagement/helpseeking behaviour)	
####	Rojas (2015) (ID:87848849)	Longitudinal hyperlipidemia outcomes at three student-run free clinic sites.	-EXCLUDE on population (women)	
####	ROLLINS (2001) (ID:87857070)	Low-income women speak out about housing: a high-stakes game of musical chairs	-EXCLUDE on date (2010)	
####	Romanelli (2017) (ID:87857612)	Individual and Systemic Barriers to Health Care: Perspectives of Lesbian, Gay, Bisexual, and Transgender Adults	-EXCLUDE on population (no housing precarity)	

####	Romero (2016) (ID:87849309)	When care work goes global: Locating the social relations of domestic work	-EXCLUDE on intervention (service engagement/helpseeking behaviour)	
####	Roncarati (2018) (ID:87848512)	Mortality Among Unsheltered Homeless Adults in Boston, Massachusetts, 2000-2009.	-EXCLUDE on intervention (service engagement/helpseeking behaviour)	
####	Rondet (2013) (ID:87849005)	Depression prevalence and primary care among vulnerable patients at a free outpatient clinic in Paris, France, in 2010: results of a cross-sectional survey.	-EXCLUDE on population (no housing precarity)	
####	ROOS (2014) (ID:87857001)	A history in-care predicts unique characteristics in a homeless population with mental illness	-EXCLUDE on intervention (service engagement/helpseeking behaviour)	
####	Roos (2016) (ID:87848742)	A qualitative study of how people with severe mental illness experience living in sheltered housing with a private fully equipped apartment.	-EXCLUDE on intervention (service engagement/helpseeking behaviour)	
####	Roper (2019) (ID:87857639)	Exploring the Cultural Meanings of Health, Self-Care, and Help-Seeking among Young Black Men: A Focused Ethnography	-EXCLUDE on population (women)	
####	Rosario (2012) (ID:87849154)	Homelessness among lesbian, gay, and bisexual youth: implications for subsequent internalizing and externalizing symptoms.	-EXCLUDE on intervention (service engagement/helpseeking behaviour)	
####	Rose (2016) (ID:87853289)	Sex disparities in use of chemotherapy and survival in patients with advanced bladder cancer	-EXCLUDE on population (no housing precarity)	
####	Rose (2022) (ID:87857823)	From an aviatrix to a eugenicist: walking with Manchester's Modernist Heroines	-EXCLUDE on intervention (service engagement/helpseeking behaviour)	
####	Rosenberg (2013) (ID:87849073)	Outdoor built environment barriers and facilitators to activity among midlife and older adults with mobility disabilities.	-EXCLUDE on intervention (service engagement/helpseeking behaviour)	
####	Rosenberg (2013) (ID:87851194)	A shared struggle with tobacco addiction.	-EXCLUDE on population (no housing precarity)	
####	Rosenberg (2015) (ID:87851619)	Insurance status and risk of cancer mortality among adolescents and young adults	-EXCLUDE on population (no housing precarity)	
####	Rosendale (2015) (ID:87850990)	Acceptance of lesbian, gay, bisexual, and transgender people-Reply.	-EXCLUDE on evidence and form (evidence not in written form or presented as research output)	
####	Rosenwohl-mack (2022) (ID:87857660)	Building H.O.U.S.E (Healthy Outcomes Using a Supportive Environment): Exploring the Role of Affordable and Inclusive Housing for LGBTQIA+ Older Adults	-EXCLUDE on intervention (service engagement/helpseeking behaviour)	
####	ROSS (2010) (ID:87857371)	Selection tests and social justice: a profile of applicants seeking admission to the social work undergraduate degree at a South African university	-EXCLUDE on country (High-Income)	
####	Ross (2017) (ID:87963314)	Reproductive justice: An introduction	-EXCLUDE on intervention (service engagement/helpseeking behaviour)	
####	Ross (2018) (ID:87857780)	In spite of the system: A qualitatively-driven mixed methods analysis of the mental health services experiences of LGBTQ people living in poverty in Ontario, Canada	-INCLUDE on title & abstract	-EXCLUDE on target group (no housing precarity)
####	Roth (2021) (ID:87851495)	An unmet need for paediatric LGBTQ training.	-EXCLUDE on intervention (service engagement/helpseeking behaviour)	
####	Rothbaum (2022) (ID:87857929)	Transgender community resilience on YouTube: Constructing an informational, emotional, and sociorelational support exchange	-EXCLUDE on population (no housing precarity)	
####	Rotstein (2020) (ID:87851633)	Risk of Mortality in Immigrants with Multiple Sclerosis in Ontario, Canada	-EXCLUDE on population (no housing precarity)	
####	Rottenberg (2014) (ID:87853131)	Prediagnostic self-assessed health and extent of social networks predict survival in older individuals with cancer: A population based cohort study	-EXCLUDE on population (no housing precarity)	
####	Rouhani (2020) (ID:87850705)	High willingness to use overdose prevention sites among female sex workers in Baltimore, Maryland.	-INCLUDE on title & abstract	-EXCLUDE on intervention (intersectionality)
####	Roulleau-Berger (2016) (ID:87849304)	Post-Western revolution in sociology: From China to Europe	-EXCLUDE on intervention (service engagement/helpseeking behaviour)	
####	ROWLANDS (2020) (ID:87857008)	Designing residential aged care for people at risk of, or experiencing, homelessness: an exploratory Australian study	-EXCLUDE on intervention (service engagement/helpseeking behaviour)	
####	Rowlands (2021) (ID:87848188)	A cross-sectional study of factors associated with unstable housing among marginalized people who use drugs in Ottawa, Canada.	-EXCLUDE on intervention (service engagement/helpseeking behaviour)	
####	Roy (2011) (ID:87849197)	Residential trajectory and HIV high-risk behaviors among Montreal street youth--a reciprocal relationship.	-EXCLUDE on intervention (service engagement/helpseeking behaviour)	

####	Roy (2013) (ID:87849066)	Patterns of cocaine and opioid co-use and polyroutes of administration among street-based cocaine users in Montreal, Canada.	-EXCLUDE on intervention (service engagement/helpseeking behaviour)	
####	ROYAL (2020) (ID:87857077)	Supporting midwives to address the needs of women experiencing severe and multiple disadvantage	-INCLUDE on title & abstract	-EXCLUDE on target group (not focused on women's behaviour/outcomes for women)
####	Roze (2018) (ID:87848567)	Factors associated with depression among homeless mothers. Results of the ENFAMS survey.	-EXCLUDE on intervention (service engagement/helpseeking behaviour)	
####	Rubin (2013) (ID:87851133)	Break the cycle of environmental health disparities: Maternal and child health aspects.	-EXCLUDE on intervention (service engagement/helpseeking behaviour)	
####	Rubio (2019) (ID:87857973)	"We Need to Redefine What We Mean by Winning": NAKASEC's Immigrant Justice Activism and Thinking Citizenship Otherwise	-EXCLUDE on intervention (service engagement/helpseeking behaviour)	
####	Ruck (2016) (ID:87857960)	The United Nations Convention on the Rights of the Child: Its Relevance for Adolescents	-EXCLUDE on population (people aged under 18 years)	
####	Rudolph (2011) (ID:87849193)	Does respondent driven sampling alter the social network composition and health-seeking behaviors of illicit drug users followed prospectively?.	-EXCLUDE on intervention (service engagement/helpseeking behaviour)	
####	Rudolph (2013) (ID:87849043)	Individual and neighborhood correlates of membership in drug using networks with a higher prevalence of HIV in New York City (2006-2009).	-EXCLUDE on intervention (service engagement/helpseeking behaviour)	
####	RUDOWICZ (2001) (ID:87857118)	Help-seeking experiences of Hong Kong social work students: implications for professional training	-EXCLUDE on date (2010)	
####	Ruff (2012) (ID:87851233)	Fostering relationships with children who are "too much to handle".	-EXCLUDE on population (people aged under 18 years)	
####	Ruff (2019) (ID:87857793)	Hope, Courage, and Resilience in the Lives of Transgender Women of Color	-EXCLUDE on population (no housing precarity)	
####	Ruiz-Perez (2017) (ID:87852822)	Socio-economic factors linked with mental health during the recession: a multilevel analysis	-EXCLUDE on population (no housing precarity)	
####	Rural Communities Opioid... (2021) (ID:87851782)	Rural Communities Opioid Response Program–Implementation (HRSA).	-EXCLUDE on evidence and form (evidence not in written form or presented as research output)	
####	Russell (2013) (ID:87857497)	Pathways, race and gender responsive reform: Through an abolitionist lens	-EXCLUDE on intervention (service engagement/helpseeking behaviour)	
####	Russell (2022) (ID:87851573)	Improving access for the vulnerable: a mixed-methods feasibility study of a pop-up model of care in south-eastern Melbourne, Australia.	-EXCLUDE on intervention (service engagement/helpseeking behaviour)	
####	**RUTTAN (2012) (ID:87856939)**	**Does a baby help young women transition out of homelessness? Motivation, coping, and parenting**	**-INCLUDE on title & abstract**	**-INCLUDE on full study**
####	Ryan (2013) (ID:87849072)	Perspectives on housing among homeless emerging adults.	-EXCLUDE on population (women)	
####	Ryan (2022) (ID:87852996)	Validation of a Deficit-Accumulation Frailty Index in the ASPirin in Reducing Events in the Elderly Study and Its Predictive Capacity for Disability-Free Survival	-EXCLUDE on population (no housing precarity)	
####	Ryan-Finn (1994) (ID:87853711)	Discussion: Empowerment as Primary Prevention: A Commentary	-EXCLUDE on date (2010)	
####	Rylander (2016) (ID:87848776)	Risk Factors for Readmission on an Adult Inpatient Psychiatric Unit.	-EXCLUDE on intervention (service engagement/helpseeking behaviour)	
####	Ryu (2019) (ID:87858047)	(Trans)gender Diverse Mental Healthcare: Applying APA Guidelines to Patient Experiences	-EXCLUDE on population (no housing precarity)	
####	Saab (2022) (ID:87851883)	Primary healthcare professionals' perspectives on patient help-seeking for lung cancer warning signs and symptoms: a qualitative study	-EXCLUDE on population (no housing precarity)	
####	Saadeh (2022) (ID:87850600)	Practicing integrative healthcare in middle east, Lebanon: An autopsychography.	-EXCLUDE on country (High-Income)	
####	Saadi (2011) (ID:87852829)	PERSPECTIVES ON PREVENTIVE HEALTH CARE AND BARRIERS TO BREAST CANCER SCREENING AMONG IRAQI WOMEN REFUGEES	-INCLUDE on title & abstract	-EXCLUDE on target group (no housing precarity)
####	Saadi (2012) (ID:87849146)	Perspectives on preventive health care and barriers to breast cancer screening among Iraqi women refugees.	-EXCLUDE on population (no housing precarity)	
####	SABINA (2012) (ID:87857190)	Help-seeking in a national sample of victimized Latino women: the influence of victimization types	-EXCLUDE on population (no housing precarity)	

####	Sacamano (2018) (ID:87848541)	Emergency Department Visits in a Cohort of Persons with Substance Use: Incorporating the Role of Social Networks.	-EXCLUDE on intervention (service engagement/helpseeking behaviour)	
####	Sadika (2020) (ID:87857599)	Intersectional Microaggressions and Social Support for LGBTQ Persons of Color: A Systematic Review of the Canadian-Based Empirical Literature	-EXCLUDE on population (no housing precarity)	
####	Saeedi (2017) (ID:87848691)	Trends in Prevalence of Diagnosed Ocular Disease and Utilization of Eye Care Services in American Veterans.	-EXCLUDE on intervention (service engagement/helpseeking behaviour)	
####	Saewyc (2010) (ID:87851410)	Restoring healthy developmental trajectories for sexually exploited young runaway girls: Fostering protective factors and reducing risk behaviors.	-EXCLUDE on intervention (service engagement/helpseeking behaviour)	
####	Safdar (2022) (ID:87851554)	Clinical Profile and Sex-Specific Recovery With Cardiac Rehabilitation After Coronary Artery Bypass Grafting Surgery	-EXCLUDE on population (no housing precarity)	
####	SAFELIVES (2018) (ID:87856942)	Safe at home: homelessness and domestic abuse	-INCLUDE on title & abstract	-EXCLUDE on intervention (intersectionality)
####	Sagan (2020) (ID:87851901)	The lonely legacy: loss and testimonial injustice in the narratives of people diagnosed with personality disorder	-EXCLUDE on intervention (service engagement/helpseeking behaviour)	
####	Sahoo (2019) (ID:87853447)	Sustainable development goals and reproductive healthcare rights of internally displaced persons in India	-EXCLUDE on country (High-Income)	
####	Saine (2019) (ID:87858050)	Measuring Disease-Related Stigma Among Patients with Chronic Hepatitis C Virus Infection	-EXCLUDE on population (no housing precarity)	
####	Sajatovic (2017) (ID:87848609)	A 6-Month Prospective Trial of a Personalized Behavioral Intervention + Long-Acting Injectable Antipsychotic in Individuals With Schizophrenia at Risk of Treatment Nonadherence and Homelessness.	-EXCLUDE on intervention (service engagement/helpseeking behaviour)	
####	Sakai-Bizmark (2022) (ID:87853044)	Reduced rate of postpartum readmissions among homeless compared with non-homeless women in New York: a population-based study using serial, cross-sectional data	-INCLUDE on title & abstract	-EXCLUDE on intervention (service engagement/helpseeking behaviour)
####	Saksena (2020) (ID:87852918)	Through their Eyes: A Photovoice and Interview Exploration of Integration Experiences of Congolese Refugee Women in Indianapolis	-EXCLUDE on intervention (service engagement/helpseeking behaviour)	
####	Salem (2013) (ID:87849007)	At a crossroads: reentry challenges and healthcare needs among homeless female ex-offenders.	-INCLUDE on title & abstract	-EXCLUDE on intervention (intersectionality)
####	Salem (2013) (ID:87849025)	Correlates of frailty among homeless adults.	-EXCLUDE on intervention (service engagement/helpseeking behaviour)	
####	Salem (2013) (ID:87851749)	Characterizing Frailty among Homeless Adults.	-EXCLUDE on intervention (service engagement/helpseeking behaviour)	
####	Salem (2014) (ID:87848959)	Constructing and identifying predictors of frailty among homeless adults-a latent variable structural equations model approach.	-EXCLUDE on intervention (service engagement/helpseeking behaviour)	
####	Salem (2014) (ID:87852649)	Development of a Frailty Framework Among Vulnerable Populations	-EXCLUDE on intervention (service engagement/helpseeking behaviour)	
####	Salem (2015) (ID:87848819)	Unmet physical and mental healthcare needs among stimulant-using gay and bisexual homeless men.	-EXCLUDE on population (women)	
####	Salem (2018) (ID:87852727)	On the Frontlines: Perspectives of Providers Working With Homeless Women	-EXCLUDE on intervention (service engagement/helpseeking behaviour)	
####	Salem (2019) (ID:87848431)	Correlates of physical, psychological, and social frailty among formerly incarcerated, homeless women.	-EXCLUDE on intervention (service engagement/helpseeking behaviour)	
####	Salem (2020) (ID:87850665)	Correlates of posttraumatic stress symptoms among formerly incarcerated, homeless women.	-EXCLUDE on intervention (service engagement/helpseeking behaviour)	
####	Salem (2021) (ID:87851890)	Transitioning into the Community: Perceptions of Barriers and Facilitators Experienced By Formerly Incarcerated, Homeless Women During Reentry—A Qualitative Study	-INCLUDE on title & abstract	-EXCLUDE on intervention (intersectionality)
####	Sales (2017) (ID:88019165)	Homeless women. The invisibility of female housing exclusion	-INCLUDE on title & abstract	-EXCLUDE on intervention (intersectionality)
####	Sales (2021) (ID:87850652)	Predictors of mental health services use among survivors of domestic violence emotional abuse.	-EXCLUDE on intervention (service engagement/helpseeking behaviour)	
####	Salgado (2019) (ID:87851992)	“Tell Me When ‘Normal’ Stops”: How Parents Recognized Their Child’s Mental Illness	-EXCLUDE on population (no housing precarity)	
####	Salvador-Carulla (2014) (ID:87848912)	Cost of borderline personality disorder in Catalonia (Spain).	-EXCLUDE on intervention (service engagement/helpseeking behaviour)	

####	Salvalaggio (2022) (ID:87852762)	Impact of an addiction medicine consult team intervention in a Canadian inner city hospital on acute care utilization: a pragmatic quasi-experimental study	-EXCLUDE on intervention (service engagement/helpseeking behaviour)	
####	Samari (2023) (ID:87857527)	Potentially exploring homelessness among refugees: a systematic review and meta-analysis	-EXCLUDE on intervention (service engagement/helpseeking behaviour)	
####	Samers (2016) (ID:87849312)	Migration	-EXCLUDE on evidence and form (evidence not in written form or presented as research output)	
####	Samiun (2020) (ID:87851904)	Health, illness and healthcare-seeking behaviour of the street dwellers of Dhaka City, Bangladesh: qualitative exploratory study	-EXCLUDE on country (High-Income)	
####	SAMPLE (2012) (ID:87856922)	In from the storm	-EXCLUDE on evidence and form (evidence not in written form or presented as research output)	
####	Samudzi (2016) (ID:87857700)	Cisgender male and transgender female sex workers in South Africa: gender variant identities and narratives of exclusion	-EXCLUDE on country (High-Income)	
####	Samuel (2015) (ID:87851639)	The health profile of street children in Africa: a literature review	-EXCLUDE on country (High-Income)	
####	SAMUEL (2015) (ID:87857397)	Utilization of mental health services among African-American male adolescents released from juvenile detention: examining reasons for within-group disparities in help-seeking behaviors	-EXCLUDE on population (women)	
####	Samuel-Nakamura (2022) (ID:87852630)	Emergency Department Use by Women Experiencing Homelessness in Los Angeles, California, USA	-INCLUDE on title & abstract	-EXCLUDE on intervention (intersectionality)
####	Samuels (2018) (ID:87851998)	"Nothing is for free...": Youth Attitudes about Engaging Resources While Unstably Housed	-EXCLUDE on population (women)	
####	Samuels-Dennis (2016) (ID:87857746)	Health Advocacy Project: Evaluating the Benefits of Service Learning to Nursing Students and Low Income Individuals Involved in a Community-Based Mental Health Promotion Project	-EXCLUDE on intervention (service engagement/helpseeking behaviour)	
####	Sanchez (2019) (ID:87857927)	Use of Experiential Learning, Reflective Writing, and Metacognition to Develop Cultural Humility Among Undergraduate Students	-EXCLUDE on intervention (service engagement/helpseeking behaviour)	
####	Sanchez (2020) (ID:87848343)	The Assessment of Recovery Capital (ARC) predicts substance abuse treatment completion.	-EXCLUDE on intervention (service engagement/helpseeking behaviour)	
####	Sánchez (2020) (ID:87858053)	Factors Influencing the Retention and Graduation of Latino Male Students: Four-Year Hispanic-Serving Institutions	-EXCLUDE on population (women)	
####	Sanchez-Cao (2013) (ID:87851177)	Psychological distress and mental health service contact of unaccompanied asylum-seeking children.	-EXCLUDE on population (people aged under 18 years)	
####	Sandel (2018) (ID:87848564)	Unstable Housing and Caregiver and Child Health in Renter Families.	-EXCLUDE on population (women)	
####	Sanders (2018) (ID:87850787)	The way she moves: Examining the perspectives and experiences of physical activity among women who have survived intimate partner violence.	-INCLUDE on title & abstract	-EXCLUDE on intervention (intersectionality)
####	Sanders (2021) (ID:87850651)	Politics of care and reverberations of trauma: Syrian refugees in Izmir, Turkey.	-EXCLUDE on country (High-Income)	
####	Sanders (2022) (ID:87857458)	The Lack of Services Provided to African American Males Who Are Homeless and Mentally Ill	-EXCLUDE on population (women)	
####	Sandhu (2011) (ID:87853276)	Social Adaptability Index: application and outcomes in a dialysis population	-EXCLUDE on intervention (service engagement/helpseeking behaviour)	
####	Sandhu (2016) (ID:87848770)	Development and psychometric properties of the client's assessment of treatment scale for supported accommodation (CAT-SA).	-EXCLUDE on intervention (service engagement/helpseeking behaviour)	
####	Sandoval (2021) (ID:87857719)	Experiences of Transgender and Nonbinary People of Color in Psychotherapy	-EXCLUDE on population (no housing precarity)	
####	Sankofa (2016) (ID:87857846)	Mapping the Blank: Centering Black Women's Vulnerability to Police Sexual Violence to Upend Mainstream Police Reform	-EXCLUDE on population (no housing precarity)	
####	Sansfaçon (2020) (ID:87963095)	Digging beneath the surface: Results from stage one of a qualitative analysis of factors influencing the well-being of trans youth in Quebec	-EXCLUDE on population (no housing precarity)	
####	Santa (2015) (ID:87851942)	"Then You Fall Off": Youth Experiences and Responses to Transitioning to Homelessness	-EXCLUDE on population (no gender focus; women population <50)	

####	Santa (2020) (ID:87848337)	Gaps in Sexual Assault Health Care Among Homeless Young Adults.	-EXCLUDE on population (no gender focus; women population <50)	
####	Santos (2018) (ID:87858030)	Disclosure, Life Experiences, and Social Relations of Sexuality: A New Generation of US Latino Queer Men	-EXCLUDE on intervention (service engagement/helpseeking behaviour)	
####	Sara (2012) (ID:87849098)	Stimulant use disorders: characteristics and comorbidity in an Australian population sample.	-EXCLUDE on population (no housing precarity)	
####	Sarah (2022) (ID:87851446)	Barriers and facilitators to access mental health services among refugee women in high-income countries: a systematic review	-INCLUDE on title & abstract	-EXCLUDE on target group (no housing precarity)<br>-EXCLUDE (systematic review)* only use with other code
####	Sarajlija (2014) (ID:87848948)	Assessment of health status and quality of life of homeless persons in Belgrade, Serbia.	-EXCLUDE on country (High-Income)	
####	Sarpong (2015) (ID:87963340)	Partner violence, help-seeking, and coping: Perceptions of African immigrant women survivors	-INCLUDE on title & abstract	-EXCLUDE (IPV/DVA but little discussion on housing)
####	Sarpong (2016) (ID:87850940)	Partner violence, help-seeking, and coping: Perceptions of African immigrant women survivors.	-INCLUDE on title & abstract	-EXCLUDE (IPV/DVA but little discussion on housing)
####	Sartorius (2010) (ID:87851742)	Young and vulnerable: Spatial-temporal trends and risk factors for infant mortality in rural South Africa (Agincourt), 1992-2007	-EXCLUDE on country (High-Income)	
####	Sathiyasusuman (2011) (ID:87849172)	Mental health services in Ethiopia: emerging public health issue.	-EXCLUDE on country (High-Income)	
####	Sattler (2014) (ID:87848967)	Indian Ocean tsunami: relationships among posttraumatic stress, posttraumatic growth, resource loss, and coping at 3 and 15 months.	-EXCLUDE on country (High-Income)	
####	Satyanarayana (2015) (ID:87853423)	Mental health consequences of violence against women and girls	-EXCLUDE on intervention (service engagement/helpseeking behaviour)	
####	SATYEN (2021) (ID:87857148)	The Royal Commission into Family Violence: trends in the reporting of intimate partner violence and help-seeking behavior	-INCLUDE on title & abstract	-EXCLUDE on intervention (service engagement/helpseeking behaviour)
####	Sauer-Zavala (2019) (ID:87848466)	Evaluating transdiagnostic, evidence-based mental health care in a safety-net setting serving homeless individuals.	-EXCLUDE on intervention (service engagement/helpseeking behaviour)	
####	Sauvaire (2022) (ID:87853015)	Down-and-out in Paris: being LGBTQ and homeless, a compulsory performance of gender and sexuality	-EXCLUDE on intervention (service engagement/helpseeking behaviour)	
####	Savage (2016) (ID:87963114)	Gendering women's homelessness	-INCLUDE on title & abstract	-EXCLUDE on intervention (service engagement/helpseeking behaviour)
####	SAVAYA (2005) (ID:87857126)	Help-seeking among Muslim Arab divorcees in Israel	-EXCLUDE on date (2010)	
####	Sawicki (2019) (ID:87857641)	Culturally competent health care for sex workers: an examination of myths that stigmatize sex work and hinder access to care	-INCLUDE on title & abstract	-EXCLUDE on intervention (intersectionality)
####	SAYAL (2010) (ID:87857206)	Parental help-seeking in primary care for child and adolescent mental health concerns: qualitative study	-EXCLUDE on population (no housing precarity)	
####	SAYEM (2015) (ID:87857338)	Women's attitudes towards formal and informal support-seeking coping strategies against intimate partner violence	-EXCLUDE on country (High-Income)	
####	Sayers (2017) (ID:87850894)	Service and infrastructure needs to support recovery programmes for indigenous community mental health consumers.	-EXCLUDE on intervention (service engagement/helpseeking behaviour)	
####	Scanlan (2017) (ID:87850868)	Evaluation of a peer-delivered, transitional and post-discharge support program following psychiatric hospitalisation.	-EXCLUDE on population (no housing precarity)	
####	Scanlon (2018) (ID:87850817)	Depression, executive dysfunction, and prior economic and social vulnerability associations in incarcerated African American men.	-EXCLUDE on population (women)	
####	Scarlett (2021) (ID:87849315)	Depression during the COVID-19 pandemic amongst residents of homeless shelters in France	-EXCLUDE on intervention (service engagement/helpseeking behaviour)	
####	Scarlett (2022) (ID:87849307)	Substance Use Among Residents of Homeless Shelters During the COVID-19 Pandemic: Findings From France	-EXCLUDE on intervention (service engagement/helpseeking behaviour)	
####	Schaaf (2021) (ID:87857907)	Necessary but not sufficient: a scoping review of legal accountability for sexual and reproductive health in low-income and middle-income countries	-EXCLUDE on country (High-Income)	
####	Schaefer (2016) (ID:87857961)	International students' perceptions of social inequalities in the U.S	-EXCLUDE on population (no housing precarity)	

####	Schamp (2021) (ID:87963364)	Women's views on barriers and facilitators for seeking alcohol and drug treatment in Belgium	-EXCLUDE on population (no housing precarity)		
####	Scharrón-del (2020) (ID:87857727)	Latinx: Inclusive Language as Liberation Praxis	-EXCLUDE on population (no housing precarity)		
####	Scheepers (2020) (ID:87857546)	Caution! Feminists at work: building organisations from the inside out	-EXCLUDE on intervention (service engagement/helpseeking behaviour)		
####	Scheer (2022) (ID:87963196)	Toward cognitive-behavioral therapy for sexual minority women: Voices from stakeholders and community members	-EXCLUDE on population (no housing precarity)		
####	Scheidell (2022) (ID:87851469)	Miscarriage and Abortion Among Women Attending Harm Reduction Services in Philadelphia: Correlations With Individual, Interpersonal, and Structural Factors.	-INCLUDE on title & abstract	-EXCLUDE on target group (no housing precarity)	
####	SCHEIM (2014) (ID:87851433)	Barriers to well-being for Aboriginal gender-diverse people: results from the Trans PULSE Project in Ontario, Canada	-EXCLUDE on intervention (service engagement/helpseeking behaviour)		
####	Scheim (2017) (ID:87850866)	Disparities in access to family physicians among transgender people in Ontario, Canada.	-EXCLUDE on intervention (service engagement/helpseeking behaviour)		
####	Scheim (2017) (ID:87850877)	Drug use among transgender people in Ontario, Canada: Disparities and associations with social exclusion.	-EXCLUDE on intervention (service engagement/helpseeking behaviour)		
####	Schick (2020) (ID:87848310)	A Community-Academic Collaboration to Support Chronic Disease Self-Management among Individuals Living in Permanent Supportive Housing.	-EXCLUDE on intervention (service engagement/helpseeking behaviour)		
####	Schiefelbein (2014) (ID:87848958)	Patterns of health care utilization among vulnerable populations in Central Texas using data from a regional health information exchange.	-EXCLUDE on population (women)		
####	Schiffler (2023) (ID:87851875)	Characteristics and Effectiveness of Co-Designed Mental Health Interventions in Primary Care for People Experiencing Homelessness: A Systematic Review	-EXCLUDE on intervention (service engagement/helpseeking behaviour)		
####	Schimmel-Bristow (2023) (ID:87850589)	The development and validation of the Transgender Resilience Measure (TRM).	-EXCLUDE on intervention (service engagement/helpseeking behaviour)		
####	Schindel (2020) (ID:87853342)	Somatic diseases of homeless people in Germany. A narrative literature review for the years 2009-2019	-EXCLUDE on intervention (service engagement/helpseeking behaviour)		
####	Schlesinger (2017) (ID:87963035)	Intersectional HCI: Engaging identity through gender, race, and class	-EXCLUDE on population (no housing precarity)		
####	**Schmidt (2015) (ID:87848805)**	**Trajectories of women's homelessness in Canada's 3 northern territories.**	**-INCLUDE on title & abstract**	**-INCLUDE on full study**	
####	Schmitt (2022) (ID:87848172)	Adverse childhood experiences among previously homeless African American women.	-EXCLUDE on intervention (service engagement/helpseeking behaviour)		
####	Schmitz (2018) (ID:87848590)	The Complexity of Family Reactions to Identity among Homeless and College Lesbian, Gay, Bisexual, Transgender, and Queer Young Adults.	-EXCLUDE on intervention (service engagement/helpseeking behaviour)		
####	Schmitz (2018) (ID:87857423)	Complex Processes of Religion and Spirituality Among Midwestern LGBTQ Homeless Young Adults	-EXCLUDE on intervention (service engagement/helpseeking behaviour)		
####	Schneider (2021) (ID:87853249)	Conceptualizing overdose trauma: The relationships between experiencing and witnessing overdoses with PTSD symptoms among street-recruited female sex workers in Baltimore, Maryland	-EXCLUDE on intervention (service engagement/helpseeking behaviour)		
####	Schnitzer (2020) (ID:87848281)	Disparities in Care: The Role of Race on the Utilization of Physical Restraints in the Emergency Setting.	-EXCLUDE on intervention (service engagement/helpseeking behaviour)		
####	SCHOFIELD (2019) (ID:87856921)	"Stuck in limbo": experiences of women in Greater Manchester on surviving domestic abuse, homelessness and a housing system not working for them	-INCLUDE on title & abstract	-INCLUDE on full study	-EXCLUDED (Quality appraisal)
####	SCHOFIELD (2021) (ID:87856948)	Fobbed off: the barriers preventing women accessing housing and homelessness support, and the women-centred approach needed to overcome them	-INCLUDE on title & abstract	-EXCLUDE on intervention (intersectionality)	
####	Schorsch (2023) (ID:87858069)	Florida Politics: Sunburn — The morning read of what's hot in Florida politics — 2.10.23	-EXCLUDE on evidence and form (evidence not in written form or presented as research output)		
####	Schott (2017) (ID:87857699)	Race, Online Space and the Feminine: Unmapping 'Black Girl Thinspiration'	-EXCLUDE on population (no housing precarity)		
####	Schrag (2014) (ID:87851114)	Social support networks of single young mothers.	-INCLUDE on title & abstract	-EXCLUDE on target group (not focused on women's behaviour/outcomes for women)	
####	Schreiter (2019) (ID:87848368)	Housing situation and healthcare for patients in a psychiatric centre in Berlin, Germany: a cross-sectional patient survey.	-EXCLUDE on intervention (service engagement/helpseeking behaviour)		

####	Schreiter (2021) (ID:87850638)	Homelessness: Care for the most vulnerable-A narrative review of risk factors, health needs, stigma, and intervention strategies.	-EXCLUDE on intervention (service engagement/helpseeking behaviour)	
####	Schroeder (2023) (ID:87851874)	Does sexuality matter? A cross-sectional study of drug use, social injecting, and access to injection-specific care among men who inject drugs in Melbourne, Australia	-EXCLUDE on population (women)	
####	Schultz-Krohn (2021) (ID:87851972)	The Lived Experience of Mothers Who Are Homeless and Participated in an Occupational Therapy Leisure Craft Group	-EXCLUDE on intervention (service engagement/helpseeking behaviour)	
####	Schutte (2022) (ID:87857967)	"Try This Brick": A Transformative Narrative Inquiry Examining the Lived Experiences of Transgender and Gender Nonconforming Community College Students	-EXCLUDE on population (no housing precarity)	
####	Schutz (2019) (ID:87848429)	Living With Dual Diagnosis and Homelessness: Marginalized Within a Marginalized Group.	-EXCLUDE on intervention (service engagement/helpseeking behaviour)	
####	Schwan (2018) (ID:87963252)	"The one thing that actually helps": Art creation as a self-care and health-promoting practice amongst youth experiencing homelessness	-EXCLUDE on population (women)	
####	Schwan (2018) (ID:87963295)	What would it take? Youth across Canada speak out on youth homelessness prevention	-EXCLUDE on intervention (service engagement/helpseeking behaviour)	
####	Schwan (2020) (ID:87963024)	The state of women's housing need & homelessness in Canada	-INCLUDE on title & abstract	-EXCLUDE on evidence and form (literature review)
####	Schwan (2020) (ID:87963057)	Girls, homelessness, and COVID-19: The urgent need for research and action	-INCLUDE on title & abstract	-EXCLUDE on intervention (service engagement/helpseeking behaviour)
####	Schwan (2021) (ID:87963027)	The Pan-Canadian Women's Housing & Homelessness Survey	-EXCLUDE on intervention (service engagement/helpseeking behaviour)<br>-EXCLUDE - but review for literature	
####	Schwarz (2017) (ID:87857591)	Aligned Across Difference: Structural Injustice, Sex Work, and Human Trafficking	-EXCLUDE on intervention (service engagement/helpseeking behaviour)	
####	Schwarz (2018) (ID:87848580)	Induced abortion among women veterans: data from the ECUUN study.	-EXCLUDE on intervention (service engagement/helpseeking behaviour)	
####	Schweizer (2021) (ID:87853049)	How health care professionals address sexuality issues in oncology? A review of literature	-EXCLUDE on population (no housing precarity)	
####	Scorgie (2017) (ID:87848616)	From fear to resilience: adolescents' experiences of violence in inner-city Johannesburg, South Africa.	-EXCLUDE on country (High-Income)	
####	Scott (2013) (ID:87849042)	The prevalence of diabetes, pre-diabetes and the metabolic syndrome in an Irish regional homeless population.	-EXCLUDE on intervention (service engagement/helpseeking behaviour)	
####	Scott (2022) (ID:87857624)	African American Women: Maintaining Resilience and Persistence in Pursuit of Human Services Industry Professional Leadership Roles	-EXCLUDE on intervention (service engagement/helpseeking behaviour)	
####	SCOTTISH (1998) (ID:87856926)	Good practice checklist for homeless women	-EXCLUDE on date (2010)	
####	SEAMARK (2018) (ID:87857095)	Barriers to support: a qualitative exploration into the help-seeking and avoidance factors of young adults	-EXCLUDE on population (no housing precarity)	
####	Secombe (2013) (ID:87849054)	Functional outcomes in high risk ICU patients in Central Australia: a prospective case series.	-EXCLUDE on intervention (service engagement/helpseeking behaviour)	
####	Seelman (2017) (ID:87857906)	A comparison of health disparities among transgender adults in Colorado (USA) by race and income	-EXCLUDE on population (no housing precarity)	
####	Seeman (2015) (ID:87850997)	The role of mental health services in addressing HIV infection among women with serious mental illness.	-EXCLUDE on intervention (service engagement/helpseeking behaviour)	
####	Segal (1998) (ID:87853709)	Health status of homeless and marginally housed users of mental health self-help agencies	-EXCLUDE on date (2010)	
####	SEGAL (2005) (ID:87857197)	Beliefs about mental illness and willingness to seek help: a cross-sectional study	-EXCLUDE on date (2010)	
####	Seibel (2018) (ID:87850779)	The impact of the parental support on risk factors in the process of gender affirmation of transgender and gender diverse people.	-EXCLUDE on country (High-Income)	
####	Seifter (2014) (ID:87853440)	Analysis of the bereavement effect after the death of a spouse in the Amish: a population-based retrospective cohort study	-EXCLUDE on population (no housing precarity)	
####	Seiler (2012) (ID:87851270)	The experiences of nurse practitioners providing health care to the homeless.	-EXCLUDE on intervention (service engagement/helpseeking behaviour)	

####	Selemani (2014) (ID:87853254)	The effect of mother's age and other related factors on neonatal survival associated with first and second birth in rural, Tanzania: evidence from Ifakara health and demographic surveillance system in rural Tanzania	-EXCLUDE on country (High-Income)
####	Selnau (2019) (ID:87849377)	Medical-Legal Partnerships in the VA	-EXCLUDE on evidence and form (evidence not in written form or presented as research output)
####	Semborski (2020) (ID:87853380)	Gender Differences in Residents' Intention to Leave Permanent Supportive Housing	-EXCLUDE on intervention (service engagement/helpseeking behaviour)
####	Semborski (2022) (ID:87848173)	Burden, Belonging, and Homelessness: Disclosure and Social Network Differences among LGBTQ Youth Recruited from a Suicide Crisis Service Provider.	-EXCLUDE on population (people aged under 18 years)
####	Semenova (2018) (ID:87849374)	Power, architecture, transition: creating a safe space for victims of domestic violence	-EXCLUDE on intervention (service engagement/helpseeking behaviour)
####	Semple (2013) (ID:87849078)	Social and behavioral characteristics of gharwalis who operate brothels in the Ganga-Jamuna area of Nagpur, India.	-EXCLUDE on country (High-Income)
####	Sen (2017) (ID:87858009)	Diversity and Social Justice in Planning Education: A Synthesis of Topics, Pedagogical Approaches, and Educational Goals in Planning Syllabi	-EXCLUDE on intervention (service engagement/helpseeking behaviour)
####	SENGOELGE (2021) (ID:87857078)	Reaching out and seeking help from frontline professionals: experiences of men perpetrating partner violence	-EXCLUDE on population (women)
####	Sentell (2016) (ID:87848731)	Pathways to potentially preventable hospitalizations for diabetes and heart failure: a qualitative analysis of patient perspectives.	-EXCLUDE on intervention (service engagement/helpseeking behaviour)
####	Sered (2014) (ID:87851067)	Can't catch a break: Gender, jail, drugs, and the limits of personal responsibility.	-EXCLUDE on intervention (service engagement/helpseeking behaviour)
####	Sered (2019) (ID:87848459)	Women on the Institutional Circuit: A 9-Year Qualitative Study.	-EXCLUDE on intervention (service engagement/helpseeking behaviour)<br>-EXCLUDE - but review for literature
####	Serena (2020) (ID:87851522)	Pathways to housing stability and viral suppression for people living with HIV/AIDS: Findings from the Building a Medical Home for Multiply Diagnosed HIV-positive Homeless Populations initiative.	-EXCLUDE on intervention (service engagement/helpseeking behaviour)
####	Sergentanis (2013) (ID:87853218)	Socioeconomic status, area remoteness, and survival from childhood leukemia: results from the Nationwide Registry for Childhood Hematological Malignancies in Greece	-EXCLUDE on population (people aged under 18 years)
####	Sevelius (2013) (ID:87857698)	Gender Affirmation: A Framework for Conceptualizing Risk Behavior Among Transgender Women of Color	-EXCLUDE on population (no housing precarity)
####	Sevelius (2014) (ID:87857559)	Barriers and Facilitators to Engagement and Retention in Care among Transgender Women Living with Human Immunodeficiency Virus	-EXCLUDE on population (no housing precarity)
####	Sewell (2019) (ID:87858078)	Being Black, Male, Gay and Gifted: Reflections on Childhood and Teaching	-EXCLUDE on population (women)
####	Sexual morbidity, quality... (Zaleta) (ID:87851531)	Sexual morbidity, quality of life, and patient-provider communication among ovarian cancer patients and survivors.	-EXCLUDE on population (no housing precarity)
####	Sexuality, LGBTI (2016) (ID:87857987)	Sexuality, LGBTI	-EXCLUDE on intervention (service engagement/helpseeking behaviour)
####	Seyed (2013) (ID:87851547)	An Analysis of the Street-Children Phenomenon in the City of Isfahan	-EXCLUDE on country (High-Income)
####	Sfeatcu (2011) (ID:87849179)	Aspects of oral and general health among a community center for the underserved.	-EXCLUDE on intervention (service engagement/helpseeking behaviour)
####	Shadravan (2020) (ID:87850677)	Cross-sectional study of homeless high service utilizers in Los Angeles county jails: Race, marginalization and opportunities for diversion.	-EXCLUDE on population (women)
####	Shah (2023) (ID:87852921)	Disparities in access to health care in adults with sinusitis in the United States	-EXCLUDE on population (no housing precarity)
####	Shahram (2021) (ID:87857916)	Promoting “Zest for Life”: A Systematic Literature Review of Resiliency Factors to Prevent Youth Suicide	-EXCLUDE on population (no housing precarity)

####	Shaia (2019) (ID:87857883)	Socially-Engineered Trauma and a New Social Work Pedagogy: Socioeducation as a Critical Foundation of Social Work Practice	-EXCLUDE on population (no housing precarity)	
####	Shaked (2021) (ID:87848241)	Predicting changes in PTSD and depression among female intimate partner violence survivors during shelter residency: A longitudinal study.	-EXCLUDE on intervention (service engagement/helpseeking behaviour)	
####	Shakeri (2012) (ID:87851763)	Impact of Diabetes, Hyper-Lipidemia, Smoking and Other Risk Factors on Survival Time of The Elderly Participants in The Tehran Lipid and Glucose Study. A 10-Year Follow-Up.	-EXCLUDE on country (High-Income)	
####	Shanahan (2013) (ID:87857767)	Youth engaging in prostitution: An examination of race, gender, and their intersections	-EXCLUDE on intervention (service engagement/helpseeking behaviour)	
####	Shankar (2015) (ID:87852008)	Self-help groups to facilitate avenues for livelihood	-EXCLUDE on intervention (service engagement/helpseeking behaviour)	
####	SHANNON (2009) (ID:87856907)	Prevalence and structural correlates of gender based violence among a prospective cohort of female sex workers	-EXCLUDE on date (2010)	
####	Sharma (2017) (ID:87848608)	Meso level multi-disciplinary approach for reduction of pre-treatment loss to follow-up in Revised National Tuberculosis Control Program, Delhi, India.	-EXCLUDE on country (High-Income)	
####	Sharma (2018) (ID:87851920)	Assessment of financial inclusive policy in Indian economy	-EXCLUDE on country (High-Income)	
####	Sharp (2015) (ID:87857530)	Keeping it from the community	-EXCLUDE on population (people aged under 18 years)	
####	SHARPEN (2018) (ID:87857011)	Jumping through hoops: how are coordinated responses to multiple disadvantage meeting the needs of women?	-INCLUDE on title & abstract	-EXCLUDE on intervention (intersectionality)
####	Shavers (2019) (ID:87857674)	The Perpetual Outsider: Voices of Black Women Pursuing Doctoral Degrees at Predominantly White Institutions	-EXCLUDE on population (no housing precarity)	
####	Shaw (2015) (ID:87848815)	Risk environments facing potential users of a supervised injection site in Ottawa, Canada.	-EXCLUDE on intervention (service engagement/helpseeking behaviour)	
####	Shaw (2021) (ID:87857584)	Citizenship and COVID-19: Syndemic Effects	-EXCLUDE on intervention (service engagement/helpseeking behaviour)	
####	Shechory (2013) (ID:87852916)	Attachment Styles, Coping Strategies, and Romantic Feelings Among Battered Women in Shelters	-EXCLUDE on intervention (service engagement/helpseeking behaviour)	
####	Shechory-Bitton (2014) (ID:87848973)	A glimpse into the world of battered ultra-Orthodox Jewish women in Israel: a follow-up study on women who resided in a shelter.	-INCLUDE on title & abstract	-EXCLUDE on intervention (service engagement/helpseeking behaviour)
####	Sheets (2014) (ID:87851087)	Review of Transgender Tuesdays: A clinic in the tenderloin.	-EXCLUDE on population (no housing precarity)	
####	SHEK (1998) (ID:87857093)	Help-seeking patterns of Chinese parents in Hong Kong	-EXCLUDE on date (2010)	
####	Sheller (2018) (ID:87851999)	Family Care Curriculum: A Parenting Support Program for Families Experiencing Homelessness	-EXCLUDE on intervention (service engagement/helpseeking behaviour)	
####	Shelley (2005) (ID:87850548)	Survival of the dispossessed : a study of seven Athol Fugard plays	-EXCLUDE on date (2010)	
####	SHELTON (2017) (ID:87851424)	"It just never worked out": how transgender and gender expansive youth understand their pathways into homelessness	-EXCLUDE on intervention (service engagement/helpseeking behaviour)	
####	Shelton (2018) (ID:87857538)	Homelessness and Housing Experiences among LGBTQ Young Adults in Seven U.S. Cities	-EXCLUDE on intervention (service engagement/helpseeking behaviour)	
####	Shelton (2018) (ID:87857585)	I'm more driven now: Resilience and resistance among transgender and gender expansive youth and young adults experiencing homelessness	-INCLUDE on title & abstract	-EXCLUDE on intervention (service engagement/helpseeking behaviour)
####	Shelton (2018) (ID:87963262)	Reversing Erasure of Youth and Young Adults Who are LGBTQ and Access Homelessness Services	-EXCLUDE on intervention (service engagement/helpseeking behaviour)	
####	Shelton (2020) (ID:87963209)	I'm more driven now: Resilience and resistance among transgender and gender expansive youth and young adults experiencing homelessness	-EXCLUDE Duplicate	
####	Shen (2018) (ID:87851524)	Poorer prognosis in young female patients with non-metastatic colorectal cancer: a hospital-based analysis of 5,047 patients in China	-EXCLUDE on country (High-Income)	
####	Shenderovich (2018) (ID:87850812)	What affects attendance and engagement in a parenting program in South Africa?	-EXCLUDE on country (High-Income)	
####	Shepp (2023) (ID:87850572)	Seeking support under the state: Aex worker's experiences navigating gender-based violence services.	-INCLUDE on title & abstract	-EXCLUDE (IPV/DVA but little discussion on housing)

####	Sherman (2022) (ID:87857586)	Transgender and Gender Diverse Community Connection, Help-Seeking, and Mental Health Among Black Transgender Women Who Have Survived Violence: A Mixed-Methods Analysis	-EXCLUDE on population (no housing precarity)	
####	Sherman (2022) (ID:87857623)	Barriers to accessing and engaging in healthcare as potential modifiers in the association between polyvictimization and mental health among Black transgender women	-EXCLUDE on population (no housing precarity)	
####	Sherry (2013) (ID:87852023)	The vulnerable researcher: facing the challenges of sensitive research	-EXCLUDE on intervention (service engagement/helpseeking behaviour)	
####	Sherwin (2021) (ID:87850514)	Women's experiences of homeless services : one city in time	-INCLUDE on title & abstract	-EXCLUDE on intervention (intersectionality)
####	Shiels (2015) (ID:87853419)	Cancer stage at diagnosis in patients infected with the human immunodeficiency virus and transplant recipients	-EXCLUDE on population (no housing precarity)	
####	SHIER (2011) (ID:87856925)	Sociocultural factors to consider when addressing the vulnerability of social service users: insights from women experiencing homelessness	-EXCLUDE on intervention (service engagement/helpseeking behaviour)	
####	SHIER (2012) (ID:87856956)	Employment difficulties experienced by employed homeless people: labor market factors that contribute to and maintain homelessness	-EXCLUDE on intervention (service engagement/helpseeking behaviour)	
####	Shinn (2015) (ID:87848832)	Longitudinal Impact of a Family Critical Time Intervention on Children in High-Risk Families Experiencing Homelessness: A Randomized Trial.	-EXCLUDE on population (people aged under 18 years)	
####	Shirley-Beavan (2020) (ID:87963285)	Women and barriers to harm reduction services: a literature review and initial findings from a qualitative study in Barcelona, Spain	-EXCLUDE on population (no housing precarity)<br>-EXCLUDE - but review for literature	
####	Shoemaker (2020) (ID:87848307)	Establishing need and population priorities to improve the health of homeless and vulnerably housed women, youth, and men: A Delphi consensus study.	-EXCLUDE on population (no gender focus; women population <50)	
####	Shokoohi (2019) (ID:87851629)	Patterns of social determinants of health associated with drug use among women living with HIV in Canada: a latent class analysis	-EXCLUDE on intervention (service engagement/helpseeking behaviour)	
####	SHOR (2006) (ID:87857158)	When children have problems: comparing help-seeking approaches of Israeli-born parents and immigrants from the former Soviet Union	-EXCLUDE on date (2010)	
####	SHOREY (2015) (ID:87857405)	The relation between trait mindfulness and aggression in men seeking residential substance use treatment	-EXCLUDE on population (no housing precarity)	
####	Short (2018) (ID:87848494)	Offending behaviour, health and wellbeing of military veterans in the criminal justice system.	-EXCLUDE on intervention (service engagement/helpseeking behaviour)	
####	SHPIEGEL (2017) (ID:87857028)	The impact of early childbirth on socioeconomic outcomes and risk indicators of females transitioning out of foster care	-EXCLUDE on population (no housing precarity)	
####	Shrestha (2013) (ID:87853321)	Short Communication: Lack of Occult HIV Infection Among Non-AIDS-Defining Cancer Patients in Three Academic Oncology Clinics in the United States	-EXCLUDE on population (no housing precarity)	
####	SHTOMPE (2014) (ID:87857208)	Negative feelings and help seeking among older adults with chronic conditions	-EXCLUDE on population (no housing precarity)	
####	Shufutinsky (2022) (ID:87858097)	Dissecting Meaning, Scope, and Roles of Social Justice and Social Action	-EXCLUDE on evidence and form (evidence not in written form or presented as research output)	
####	Shukla (2021) (ID:87848218)	To study the impact of active case finding (ACF) among the TB patients detected in South Delhi.	-EXCLUDE on country (High-Income)	
####	SHWARTZ-ZIV (2022) (ID:87857044)	Activist mothers in need of public housing and the welfare services: negotiating othering, mothering and resistance	-EXCLUDE on intervention (service engagement/helpseeking behaviour)	
####	Sidebottom (2017) (ID:87850876)	Prenatal care: Associations with prenatal depressive symptoms and social support in low-income urban women.	-EXCLUDE on population (no housing precarity)	
####	SIEBERT (2005) (ID:87857140)	Help seeking for AOD misuse among social workers: patterns, barriers, and implications	-EXCLUDE on date (2010)	
####	SIEGEL (2014) (ID:87857007)	Early life circumstances as contributors to HIV infection	-EXCLUDE on intervention (service engagement/helpseeking behaviour)	
####	Siegfriedt (2019) (ID:87858008)	From Victim to Volunteer: A Life Course Perspective and the Transition to Adulthood for Individuals Who Have Sold Sex	-EXCLUDE on intervention (service engagement/helpseeking behaviour)<br>-EXCLUDE - but review for literature	

####	Silva (2015) (ID:87850986)	Powerlessness and service utilization by people without homes by chronic homelessness, age, gender, mental health, and substance use.	-EXCLUDE on evidence and form (evidence not in written form or presented as research output)	
####	Silvestrini (2017) (ID:87851570)	Healthcare utilization among urban homeless followed by an outpatient clinic: more intensive use by migrant groups	-EXCLUDE on population (no gender focus; women population <50)<br>-EXCLUDE - but review for literature	
####	Simmons (2012) (ID:87849104)	Barriers to drug treatment for IDU couples: the need for couple-based approaches.	-EXCLUDE on population (no housing precarity)	
####	Simmons (2023) (ID:87850571)	Examining African American women probationer and parolees' utilization of mental health services.	-EXCLUDE on intervention (service engagement/helpseeking behaviour)	
####	Simon (2017) (ID:87848607)	Linking patients with buprenorphine treatment in primary care: Predictors of engagement.	-EXCLUDE on population (no housing precarity)	
####	Simon-Kumar (2017) (ID:87857606)	Mobilising culture against domestic violence in migrant and ethnic communities: practitioner perspectives from Aotearoa/New Zealand	-EXCLUDE on intervention (service engagement/helpseeking behaviour)	
####	Simpson (2014) (ID:87857603)	Oppression and Barriers to Service for Black, Lesbian Survivors of Intimate Partner Violence	-INCLUDE on title & abstract	-EXCLUDE (IPV/DVA but little discussion on housing)
####	Simpson (2015) (ID:87851429)	Scotland: the best place in the world to bring up children? A collection of essays about parenting in Scotland	-EXCLUDE on intervention (service engagement/helpseeking behaviour)	
####	Singer (2020) (ID:87857587)	Trans Rights Are Not Just Human Rights: Legal Strategies for Trans Justice	-EXCLUDE on population (no housing precarity)	
####	Singh (2015) (ID:87850989)	A comparative study on the death of unclaimed homeless males and females based on autopsy and circumstantial findings in a large North Indian population.	-EXCLUDE on country (High-Income)	
####	Singh (2015) (ID:87851943)	Towards Food Autonomy: Connectivity and Self-Help Groups in Hisar, India	-EXCLUDE on country (High-Income)	
####	Singh (2018) (ID:87851924)	The Queer and Transgender Resilience Workbook: Skills for Navigating Sexual Orientation and Gender Expression	-EXCLUDE on evidence and form (evidence not in written form or presented as research output)	
####	Singhal (2016) (ID:87848794)	Parental perceptions of avoidability of their child's emergency department visit.	-EXCLUDE on population (people aged under 18 years)	
####	SIRRIYEH (2010) (ID:87857313)	Support for migrant children	-EXCLUDE on population (people aged under 18 years)	
####	Sisselman-Borgia (2018) (ID:87963299)	The association of racial and homelessness microaggressions and physical and mental health in a sample of homeless youth	-EXCLUDE on intervention (service engagement/helpseeking behaviour)	
####	Sisselman-Borgia (2021) (ID:87963273)	An adapted life skills empowerment program for homeless youth: Preliminary findings	-EXCLUDE on intervention (service engagement/helpseeking behaviour)	
####	SKEATE (2002) (ID:87857361)	Duration of untreated psychosis and pathways to care in first-episode psychosis: investigation of help-seeking behaviour in primary care	-EXCLUDE on date (2010)	
####	Skenazy (2014) (ID:87852019)	Self Help for Synagogues	-EXCLUDE on evidence and form (evidence not in written form or presented as research output)	
####	Skobba (2015) (ID:87852938)	Doubling up and the erosion of social capital among very low income households	-EXCLUDE on intervention (service engagement/helpseeking behaviour)	
####	Skosireva (2014) (ID:87857440)	Different faces of discrimination: Perceived discrimination among homeless adults with mental illness in healthcare settings	-EXCLUDE on intervention (service engagement/helpseeking behaviour)	
####	SKUBBY (2015) (ID:87857186)	The help-seeking experiences of parents of children with a first-episode of psychosis	-EXCLUDE on population (no housing precarity)	
####	Slakoff (2022) (ID:87963244)	Barriers to reporting, barriers to services: Challenges for Transgender survivors of intimate Partner violence and sexual victimization	-INCLUDE on title & abstract	-EXCLUDE (IPV/DVA but little discussion on housing)
####	SLEATH (2006) (ID:87856923)	Racial differences in the use of antidepressants and counseling for depression among homeless women	-EXCLUDE on date (2010)	
####	Sleed (2013) (ID:87849056)	A psychotherapeutic baby clinic in a hostel for homeless families: practice and evaluation.	-EXCLUDE on population (people aged under 18 years)	
####	Slesnick (2012) (ID:87851230)	Experiences associated with intervening with homeless, substance-abusing mothers: The importance of success.	-INCLUDE on title & abstract	-EXCLUDE on evidence and form (not empirical)
####	**Slesnick (2013) (ID:87849070)**	**Treatment desires and symptomatology among substance-abusing homeless mothers: what I want versus what I need.**	**-INCLUDE on title & abstract**	**-INCLUDE on full study**

####	Slesnick (2013) (ID:87851143)	Two-year predictors of runaway and homeless episodes following shelter services among substance abusing adolescents.	-EXCLUDE on intervention (service engagement/helpseeking behaviour)	
####	Small (2011) (ID:87849222)	Determinants of physician utilization, emergency room use, and hospitalizations among populations with multiple health vulnerabilities.	-EXCLUDE on population (women)	
####	Smelson (2013) (ID:87849064)	A wraparound treatment engagement intervention for homeless veterans with co-occurring disorders.	-EXCLUDE on population (women)	
####	Smelson (2016) (ID:87848755)	Integrating Permanent Supportive Housing and Co-Occurring Disorders Treatment for Individuals Who Are Homeless.	-EXCLUDE on population (women)	
####	Smelson (2018) (ID:87848485)	Permanent Supportive Housing and Specialized Co-Occurring Disorders Wraparound Services for Homeless Individuals.	-EXCLUDE on intervention (service engagement/helpseeking behaviour)	
####	SMITH (1995) (ID:87857059)	Social work and resettlement	-EXCLUDE on date (2010)	
####	SMITH (2005) (ID:87857243)	Feel the fear and do it anyway': meeting the occupational needs of refugees and people seeking asylum	-EXCLUDE on date (2010)	
####	Smith (2013) (ID:87851150)	The association between housing and risk behaviors among homeless and unstably housed African American women living with HIV/AIDS.	-EXCLUDE on intervention (service engagement/helpseeking behaviour)	
####	SMITH (2013) (ID:87856990)	Methodological problems of sampling young homeless people in four European societies with different levels of service provision and definitions of homelessness	-EXCLUDE on intervention (service engagement/helpseeking behaviour)	
####	Smith (2015) (ID:87852012)	A journey of hope and perseverance	-EXCLUDE on evidence and form (evidence not in written form or presented as research output)	
####	Smith (2016) (ID:87857864)	Facing the Dragon: Black Mothering, Sequelae, and Gendered Necropolitics in the Americas	-EXCLUDE on intervention (service engagement/helpseeking behaviour)	
####	Smith (2021) (ID:87858095)	The Lived Experience of Gay Black Men in the Black Church Through the Lens of Intra-Racism and Toxic Masculinty	-EXCLUDE on population (women)	
####	Smith-Maddox (2020) (ID:87857422)	Developing a Policy Advocacy Practice for Preventing and Ending Homelessness	-EXCLUDE on evidence and form (evidence not in written form or presented as research output)	
####	Smithson (2021) (ID:87857944)	'They Really Should Start Listening to You': The Benefits and Challenges of Co-Producing a Participatory Framework of Youth Justice Practice	-EXCLUDE on intervention (service engagement/helpseeking behaviour)	
####	Smye (2011) (ID:87963083)	Harm reduction, methadone maintenance treatment and the root causes of health and social inequities: An intersectional lens in the Canadian context	-EXCLUDE on intervention (service engagement/helpseeking behaviour)	
####	Smyth (2021) (ID:87963087)	COVID-19 lockdowns, intimate partner violence and coercive control	-EXCLUDE on intervention (service engagement/helpseeking behaviour)	
####	Snow (2018) (ID:87852756)	Heard and valued: the development of a model to meaningfully engage marginalized populations in health services planning	-EXCLUDE on intervention (service engagement/helpseeking behaviour)	
####	Sobey (2015) (ID:87853055)	Risk of Major Cardiovascular Events in People with Down Syndrome	-EXCLUDE on population (no housing precarity)	
####	social sciences (2021) (ID:87858075)	social sciences	-EXCLUDE on evidence and form (evidence not in written form or presented as research output)	
####	Sofia (2022) (ID:87851507)	Examining Barriers to Medication Adherence and Retention in Care among Women Living with HIV in the Face of Homelessness and Unstable Housing	-INCLUDE on title & abstract	-EXCLUDE on intervention (intersectionality)
####	Solar (2011) (ID:87851336)	Support workers for patients in borderline personality disorder.	-EXCLUDE on evidence and form (evidence not in written form or presented as research output)	
####	Soled (2021) (ID:87849352)	Distributive Justice as a Means of Combating Systemic Racism in Healthcare	-EXCLUDE on population (no housing precarity)	
####	Somers (2015) (ID:87848840)	Changes in daily substance use among people experiencing homelessness and mental illness: 24-month outcomes following randomization to Housing First or usual care.	-EXCLUDE on population (women)	
####	Somers (2016) (ID:87848769)	Accuracy of reported service use in a cohort of people who are chronically homeless and seriously mentally ill.	-EXCLUDE on intervention (service engagement/helpseeking behaviour)	
####	Somjet (2017) (ID:87851604)	Empirical lessons regarding contraception in a protracted refugee setting: A descriptive study from Maela camp on the Thai-Myanmar border 1996 - 2015.	-EXCLUDE on country (High-Income)	

####	SOMLAI (1998) (ID:87856954)	Patterns, predictors and situational contexts of HIV risk behaviors among homeless men and women	-EXCLUDE on date (2010)
####	Song (2015) (ID:87851033)	The association of social networks with substance use among homeless men in Los Angeles who have unprotected sex with women.	-EXCLUDE on population (women)
####	Soo (2022) (ID:87853040)	Characterizing Multisystem High Users of the Homeless Services, Jail, and Hospital Systems in Chicago, Illinois	-EXCLUDE on intervention (service engagement/helpseeking behaviour)
####	SOORKIA (2011) (ID:87857254)	Factors influencing attitudes towards seeking professional psychological help among South Asian students in Britain	-EXCLUDE on population (no housing precarity)
####	Sooy-Mossey (2022) (ID:87853229)	Health Disparities in the Treatment of Supraventricular Tachycardia in Pediatric Patients	-EXCLUDE on intervention (service engagement/helpseeking behaviour)
####	SORENSON (2021) (ID:87857248)	The endemic amid the pandemic: seeking help for violence against women in the initial phases of COVID-19	-EXCLUDE on intervention (service engagement/helpseeking behaviour)<br>-EXCLUDE - but review for literature
####	SORMANTI (2008) (ID:87857117)	Intimate partner violence among midlife and older women: a descriptive analysis of women seeking medical services	-EXCLUDE on date (2010)
####	Sorsa (2018) (ID:87857408)	Engagement in help-seeking of dual diagnosed mothers at a low-threshold service: grasping life through co-created opportunities	-EXCLUDE on population (no housing precarity)
####	SOS receives major... (2014) (ID:87852018)	SOS receives major recognition	-EXCLUDE on evidence and form (evidence not in written form or presented as research output)
####	Soskolne (2006) (ID:87857410)	Help seeking and barriers to utilisation of medical and health social work services among ageing immigrants to Israel from the former Soviet Union	-EXCLUDE on date (2010)
####	Souza (2011) (ID:87851313)	Outcomes for street children and youth under multidisciplinary care in a drop-in centre in Tegucigalpa, Honduras.	-EXCLUDE on country (High-Income)
####	Spalding (2023) (ID:87857951)	The Experiences of LGBQ Clients Accessing Substance Use Treatment: Understanding Barriers and Facilitators Through Minority Stress Theory	-EXCLUDE on population (no housing precarity)
####	Sparbanie (2020) (ID:87851979)	Understanding the Occupational Therapy Perspective of Working with the Homeless Population: Self-Care Interventions	-EXCLUDE on intervention (service engagement/helpseeking behaviour)
####	Spencer (2022) (ID:87857456)	Women's Lived Experiences with Temporary Assistance for Needy Families (TANF): How TANF Can Better Support Women's Wellbeing and Reduce Intimate Partner Violence	-EXCLUDE on intervention (service engagement/helpseeking behaviour)
####	Spicer (2010) (ID:87851369)	Healthcare needs of the transgender homeless population.	-EXCLUDE on intervention (service engagement/helpseeking behaviour)
####	Spinelli (2020) (ID:87848264)	Viral suppression rates in a safety-net HIV clinic in San Francisco destabilized during COVID-19.	-EXCLUDE on intervention (service engagement/helpseeking behaviour)
####	Spinner (2017) (ID:87851620)	Issues in Specific Patient Populations	-EXCLUDE on intervention (service engagement/helpseeking behaviour)
####	Spinola (2021) (ID:87850657)	A psychosocial mediational model of homelessness among U.S. Male and female veterans who served in Iraq and Afghanistan.	-EXCLUDE on intervention (service engagement/helpseeking behaviour)
####	Spitzer (2016) (ID:87849366)	Alone at home: post-9/11 military veterans and American housing and homelessness policy	-EXCLUDE on intervention (service engagement/helpseeking behaviour)
####	Spivak (2019) (ID:87848376)	Financial hardship among individuals with serious mental illness.	-EXCLUDE on intervention (service engagement/helpseeking behaviour)
####	Spradling (2021) (ID:87848204)	Psychosocial Obstacles to Hepatitis C Treatment Initiation Among Patients in Care: A Hitch in the Cascade of Cure.	-EXCLUDE on intervention (service engagement/helpseeking behaviour)
####	Spragg (2020) (ID:87851903)	Understanding the Experiences of Military-Related Sexual Violence Among Women in the Army National Guard	-EXCLUDE on population (no housing precarity)
####	Spriggs (2013) (ID:87852028)	An analysis of North Carolina homeless shelter policies: Potential for fracturing the integrity of help-seeking homeless families	-EXCLUDE on intervention (service engagement/helpseeking behaviour)
####	Springgate (2018) (ID:87848505)	Comparative Effectiveness of Coalitions Versus Technical Assistance for Depression Quality Improvement in Persons with Multiple Chronic Conditions.	-EXCLUDE on intervention (service engagement/helpseeking behaviour)

####	SRIPADA (2021) (ID:87857100)	Help-seeking behavior of South Asian women in domestic violence: a scoping review	-EXCLUDE - but review for literature<br>-EXCLUDE on intervention (service engagement/helpseeking behaviour)	
####	St (2012) (ID:87857773)	Exploring the relevance of feminist pedagogy to community psychology: continuing the dialogue	-EXCLUDE on population (no housing precarity)	
####	St Mungo (2019) (ID:88019159)	Women at St Mungo's - A three year strategy for 2019-2022	-EXCLUDE on intervention (service engagement/helpseeking behaviour)	
####	Stagg (2019) (ID:87848428)	Improving engagement with healthcare in hepatitis C: a randomised controlled trial of a peer support intervention.	-EXCLUDE on population (women)	
####	Stahl (2016) (ID:87858005)	Adolescence: The issue of lesbian, gay, bisexual, and transgender	-EXCLUDE on population (no housing precarity)	
####	STAINBROOK (2006) (ID:87857029)	Similarities in the characteristics and needs of women with children in homeless family and domestic shelter violence	-EXCLUDE on date (2010)	
####	Stajduhar (2020) (ID:87848303)	"Everybody in this community is at risk of dying": An ethnographic exploration on the potential of integrating a palliative approach to care among workers in inner-city settings.	-EXCLUDE on intervention (service engagement/helpseeking behaviour)	
####	Stancliff (2012) (ID:87849102)	Opioid maintenance treatment as a harm reduction tool for opioid-dependent individuals in New York City: the need to expand access to buprenorphine/naloxone in marginalized populations.	-EXCLUDE on intervention (service engagement/helpseeking behaviour)	
####	Stang (2016) (ID:87853416)	A novel method for identifying settings for well-motivated ecologic studies of cancer	-EXCLUDE on population (no housing precarity)	
####	Stanic (2021) (ID:87848247)	The impact of the housing status on clinical outcomes and health care utilization among individuals living with HIV.	-EXCLUDE on population (women)	
####	STEELE (2021) (ID:87856918)	Keeping us safer: an approach for supporting homeless women experiencing multiple disadvantage	-INCLUDE on title & abstract	-EXCLUDE on intervention (intersectionality)
####	Stefanovics (2023) (ID:87850569)	Sex-specific risk and resilience correlates of suicidal ideation in U.S. military veterans.	-EXCLUDE on population (no housing precarity)	
####	Stein (2010) (ID:87849257)	Completion and subject loss within an intensive hepatitis vaccination intervention among homeless adults: the role of risk factors, demographics, and psychosocial variables.	-EXCLUDE on intervention (service engagement/helpseeking behaviour)	
####	Stein (2012) (ID:87851295)	Impact of hepatitis B and C infection on health services utilization in homeless adults: A test of the Gelberg-Andersen behavioral model for vulnerable populations.	-EXCLUDE on population (women)	
####	Stein (2014) (ID:87848964)	Looking for the uninsured in Massachusetts? Check opioid dependent persons seeking detoxification.	-EXCLUDE on intervention (service engagement/helpseeking behaviour)	
####	Stein (2016) (ID:87850954)	Linkage to primary care for persons first receiving injectable naltrexone during inpatient opioid detoxification.	-EXCLUDE on population (women)	
####	Steingrimsdottir (2012) (ID:87853161)	Trends in life expectancy by education in Norway 1961-2009	-EXCLUDE on population (no housing precarity)	
####	Stenius-Ayoade (2017) (ID:87848627)	Mental disorders and the use of primary health care services among homeless shelter users in the Helsinki metropolitan area, Finland.	-EXCLUDE on population (women)	
####	Stephen (1998) (ID:87850523)	Understanding context, agency and process in the health of homeless young women in Glasgow : a qualitative study	-EXCLUDE on date (2010)	
####	Stephens (2019) (ID:87857725)	Training for Life and Healing : The Systemic Empowerment of Aboriginal and Torres Strait Islander Men and Women Through Vocational Education and Training	-EXCLUDE on population (no housing precarity)	
####	Stergiopoulos (2015) (ID:87848824)	The effectiveness of an integrated collaborative care model vs. a shifted outpatient collaborative care model on community functioning, residential stability, and health service use among homeless adults with mental illness: a quasi-experimental study.	-EXCLUDE on population (women)	
####	Stergiopoulos (2015) (ID:87848835)	Effectiveness of Housing First with Intensive Case Management in an Ethnically Diverse Sample of Homeless Adults with Mental Illness: A Randomized Controlled Trial.	-EXCLUDE on intervention (service engagement/helpseeking behaviour)	
####	Stergiopoulos (2015) (ID:87850998)	Is all psychiatry social?: Reply.	-EXCLUDE on evidence and form (evidence not in written form or presented as research output)	

####	Stergiopoulos (2016) (ID:87848714)	The effectiveness of a Housing First adaptation for ethnic minority groups: findings of a pragmatic randomized controlled trial.	-EXCLUDE on intervention (service engagement/helpseeking behaviour)	
####	Stergiopoulos (2016) (ID:87848752)	Racial-Ethnic Differences in Health Service Use in a Large Sample of Homeless Adults With Mental Illness From Five Canadian Cities.	-EXCLUDE on intervention (service engagement/helpseeking behaviour)	
####	Stergiopoulos (2018) (ID:87848535)	Bridging Hospital and Community Care for Homeless Adults with Mental Health Needs: Outcomes of a Brief Interdisciplinary Intervention.	-EXCLUDE on intervention (service engagement/helpseeking behaviour)	
####	STERMAC (2004) (ID:87857088)	Stranger and acquaintance sexual assault of adult males	-EXCLUDE on date (2010)	
####	Stevens (2013) (ID:87857800)	Social environment and sexual risk-taking among gay and transgender African American youth	-EXCLUDE on population (no housing precarity)	
####	Stevens (2016) (ID:87857903)	Social media in the sexual lives of African American and latino youth: Challenges and opportunities in the digital neighborhood	-EXCLUDE on intervention (service engagement/helpseeking behaviour)	
####	STEVENS (2021) (ID:87857346)	An exploration of why health professionals seek to hold statutory powers in mental health services in England: considerations of the approved mental health professional role	-EXCLUDE on population (no housing precarity)	
####	Stevenson (2012) (ID:87851274)	'We did more rough sleeping just to be together'-Homeless drug users' romantic relationships in hostel accommodation.	-EXCLUDE on population (no gender focus; women population <50)	
####	Stewart (2013) (ID:87858073)	Promoting gender equity through health research: impacts and insights from a Canadian initiative	-EXCLUDE on population (no housing precarity)	
####	Stewart (2018) (ID:87850791)	Lesbian, gay, bisexual, and transgender Americans at risk: Problems and solutions: The middle-aged, seniors, and current topics.	-EXCLUDE on population (no housing precarity)	
####	Stewart (2018) (ID:87852001)	Intrapersonal and Social-Contextual Factors Related to Psychological Well-Being among Youth Experiencing Homelessness	-EXCLUDE on intervention (service engagement/helpseeking behaviour)	
####	Stewart (2023) (ID:87963248)	Cultivated invisibility and migrants' experiences of homelessness during the COVID-19 pandemic	-EXCLUDE on population (no gender focus; women population <50)<br>-EXCLUDE - but review for literature	
####	Stiekema (2018) (ID:87848552)	Effects of a lifestyle intervention on psychosocial well-being of severe mentally ill residential patients: ELIPS, a cluster randomized controlled pragmatic trial.	-EXCLUDE on population (no housing precarity)	
####	Stirling (2015) (ID:87858064)	Occupying Identities: Hierarchal Divisions and Collective Identity in the Occupy Movement	-EXCLUDE on intervention (service engagement/helpseeking behaviour)	
####	Stockman (2015) (ID:87963152)	Intimate partner violence and its health impact on ethnic minority women	-INCLUDE on title & abstract	-EXCLUDE (IPV/DVA but little discussion on housing)
####	Stockwell (2018) (ID:87848582)	Does managing the consumption of people with severe alcohol dependence reduce harm? A comparison of participants in six Canadian managed alcohol programs with locally recruited controls.	-EXCLUDE on intervention (service engagement/helpseeking behaviour)	
####	Stockwell (2021) (ID:87848231)	Trajectories of Alcohol Use and Related Harms for Managed Alcohol Program Participants over 12 Months Compared with Local Controls: A Quasi-Experimental Study.	-EXCLUDE on population (women)	
####	Stoicescu (2017) (ID:87850544)	The syndemic effects of intimate partner violence, substance use, and depression on HIV risk among Indonesian women who inject drugs : findings from the Women Speak Out study	-EXCLUDE on country (High-Income)	
####	Stokes (2016) (ID:87848782)	Care Seeking Patterns Among Women Who Have Experienced Gender-Based Violence in Afghanistan.	-EXCLUDE on country (High-Income)	
####	Stolzenburg (2019) (ID:87851988)	Individuals with currently untreated mental illness: causal beliefs and readiness to seek help	-EXCLUDE on population (no housing precarity)	
####	Stone (2020) (ID:87857801)	Multiplicity, Race, and Resilience: Transgender and Non-Binary People Building Community	-EXCLUDE on population (no housing precarity)	
####	Storholm (2022) (ID:87857838)	Intimate Partner Violence and HIV Prevention Among Sexual Minority Men: Protocol for a Prospective Mixed Methods Cohort Study	-EXCLUDE on population (women)	
####	Stormon (2018) (ID:87848509)	Does a facilitated pathway improve access to dental services for homeless and disadvantaged adults?.	-EXCLUDE on population (no gender focus; women population <50)	

####	Stormon (2019) (ID:87848472)	Evaluation of a community based dental clinic for youth experiencing homelessness in Brisbane.	-EXCLUDE on intervention (service engagement/helpseeking behaviour)	
####	Story (2014) (ID:87848965)	Influenza vaccination, inverse care and homelessness: cross-sectional survey of eligibility and uptake during the 2011/12 season in London.	-EXCLUDE on intervention (service engagement/helpseeking behaviour)	
####	Stout (2011) (ID:87851963)	Collective Visioning: How Groups Can Work Together for a Just and Sustainable Future	-EXCLUDE on intervention (service engagement/helpseeking behaviour)	
####	Straiton (2017) (ID:87848696)	Mental Health of Refugees and Non-refugees from War-Conflict Countries: Data from Primary Healthcare Services and the Norwegian Prescription Database.	-EXCLUDE on intervention (service engagement/helpseeking behaviour)	
####	Strange (2023) (ID:87857710)	Communicating Research as a Public Discussion: The PHED Commission on the Future of Health Care Post-COVID 19	-EXCLUDE on population (no housing precarity)	
####	Strauss (2011) (ID:87849196)	Is military sexual trauma associated with trading sex among women veterans seeking outpatient mental health care?.	-EXCLUDE on intervention (service engagement/helpseeking behaviour)	
####	Strauss (2020) (ID:87848356)	Associations between negative life experiences and the mental health of trans and gender diverse young people in Australia: findings from Trans Pathways.	-EXCLUDE on intervention (service engagement/helpseeking behaviour)	
####	Strehlau (2012) (ID:87849097)	Mental health, concurrent disorders, and health care utilization in homeless women.	-EXCLUDE on intervention (service engagement/helpseeking behaviour)	
####	Stringfellow (2016) (ID:87848768)	Substance use among persons with homeless experience in primary care.	-EXCLUDE on intervention (service engagement/helpseeking behaviour)	
####	Stubbs (2020) (ID:87857795)	Authoritarian Neoliberalism, Radical Conservatism and Social Policy within the European Union: Croatia, Hungary and Poland	-EXCLUDE on intervention (service engagement/helpseeking behaviour)	
####	**Stylianou (2021) (ID:87857607)**	**“My Sister Is the One That Made Me Stay Above Water”: How Social Supports Are Maintained and Strained When Survivors of Intimate Partner Violence Reside in Emergency Shelter Programs**	**-INCLUDE on title & abstract**	**-INCLUDE on full study**
####	Suárez (2021) (ID:87857677)	Using photovoice as an arts-based method for grieving: LGBTQ + students and the pulse nightclub shooting	-EXCLUDE on population (no housing precarity)	
####	Subject Index (2016) (ID:87851939)	Subject Index	-EXCLUDE on evidence and form (evidence not in written form or presented as research output)	
####	Substance abuse: Case... (YF) (ID:87849348)	Substance abuse: Case management and dental treatment	-EXCLUDE on population (no housing precarity)	
####	Sudore (2018) (ID:87848532)	Advance Care Planning for Older Homeless-Experienced Adults: Results from the Health Outcomes of People Experiencing Homelessness in Older Middle Age Study.	-EXCLUDE on intervention (service engagement/helpseeking behaviour)	
####	Suen (2020) (ID:87851603)	Effects of general and sexual minority-specific COVID-19-related stressors on the mental health of lesbian, gay, and bisexual people in Hong Kong	-EXCLUDE on country (High-Income)	
####	Sulkowski (2020) (ID:87848250)	Impact of a digital medicine programme on hepatitis C treatment adherence and efficacy in adults at high risk for non-adherence.	-EXCLUDE on intervention (service engagement/helpseeking behaviour)	
####	Sulo (2020) (ID:87853022)	Socioeconomic Gradientsn Mortality Following HF Hospitalization in a Country With Universal Health Care Coverage	-EXCLUDE on population (no housing precarity)	
####	Sun (2012) (ID:87851961)	Helping Homeless Individuals with Co-occurring Disorders: The Four Components	-EXCLUDE on population (no gender focus; women population <50)	
####	Sun (2018) (ID:87963144)	Re-entry experiences of Black men living with HIV/AIDS after release from prison: Intersectionality and implications for care	-EXCLUDE on population (women)	
####	Suphanchaimat (2015) (ID:87852898)	Challenges in the provision of healthcare services for migrants: a systematic review through providers' lens	-EXCLUDE on population (no gender focus; women population <50)<br>-EXCLUDE - but review for literature	
####	SURRATT (2012) (ID:87857073)	HIV risk among female sex workers in Miami: the impact of violent victimization and untreated mental illness	-EXCLUDE on intervention (service engagement/helpseeking behaviour)	
####	Surratt (2015) (ID:87848883)	Medication adherence challenges among HIV positive substance abusers: the role of food and housing insecurity.	-EXCLUDE on intervention (service engagement/helpseeking behaviour)	
####	Sutter (2017) (ID:87857930)	An Integrated Behavioral Model of Healthcare Utilization Among Transgender and Gender-Nonconforming Adults	-EXCLUDE on population (no housing precarity)	

####	SUTTON (2006) (ID:87857358)	A qualitative study exploring refugee minors' personal accounts of post-traumatic growth and positive change processes in adapting to life in the UK	-EXCLUDE on date (2010)	
####	Sutton (2017) (ID:87848669)	Social Determinants of HIV Disparities in the Southern United States and in Counties with Historically Black Colleges and Universities (HBCUs), 2013-2014.	-EXCLUDE on population (no housing precarity)	
####	Suyemoto (2021) (ID:87857812)	"TAKING THE EMPATHY TO AN ACTIVIST STATE": ALLY DEVELOPMENT AS CONTINUOUS CYCLES OF CRITICAL UNDERSTANDING AND ACTION	-EXCLUDE on population (no housing precarity)	
####	Swabri (2019) (ID:87848470)	Health status of the homeless in Dublin: does the mobile health clinic improve access to primary healthcare for its users?.	-EXCLUDE on intervention (service engagement/helpseeking behaviour)	
####	Swahn (2014) (ID:87848896)	Demographic and psychosocial characteristics of mobile phone ownership and usage among youth living in the slums of Kampala, Uganda.	-EXCLUDE on country (High-Income)	
####	Sweeney (2023) (ID:87963294)	A critical review of intersectionality, social work education and sex work: an Irish perspective	-EXCLUDE on intervention (service engagement/helpseeking behaviour)	
####	Sweeney-Magee (2022) (ID:87853382)	Patterns and determinants of adherence to primary and secondary colorectal cancer prevention recommendations in the BC Generations Project	-EXCLUDE on population (no housing precarity)	
####	Sweet (2023) (ID:87857999)	Stronger together: Supporting Generation Z college students through relational-cultural therapy	-EXCLUDE on population (no housing precarity)	
####	Symes (2014) (ID:87848963)	Physical and sexual intimate partner violence, women's health and children's behavioural functioning: entry analysis of a seven-year prospective study.	-EXCLUDE on population (no housing precarity)	
####	Symes (2020) (ID:87848360)	Evaluating an Intergenerational Model to Explain the Path From Violence Against Mothers to Child Behavior and Academic Outcomes.	-EXCLUDE on population (people aged under 18 years)	
####	**Sznajder-Murray (2011) (ID:87851296)**	**"Don't leave me hanging": Homeless mothers' perceptions of service providers.**	**-INCLUDE on title & abstract**	**-INCLUDE on full study**
####	Szymkowiak (2017) (ID:87848614)	Persistent Super-Utilization of Acute Care Services Among Subgroups of Veterans Experiencing Homelessness.	-EXCLUDE on population (women)	
####	Tabong (2021) (ID:87853443)	Reasons for the utilization of the services of traditional birth attendants during childbirth: A qualitative study in Northern Ghana	-EXCLUDE on country (High-Income)	
####	Takada (2013) (ID:87851131)	Post-traumatic stress disorders and mental health care (lessons learned from the Hanshin-Awaji Earthquake, Kobe, 1995).	-EXCLUDE on country (High-Income)	
####	Takousi (2017) (ID:87851476)	Patients' health related quality of life after coronary revascularization : a longitudinal mixed method study	-EXCLUDE on population (no housing precarity)	
####	Talmet (2023) (ID:87850566)	Female adult.	-EXCLUDE on population (no housing precarity)	
####	Tan (2021) (ID:87848232)	Increases in social support co-occur with decreases in depressive symptoms and substance use problems among adults in permanent supportive housing: an 18-month longitudinal study.	-EXCLUDE on intervention (service engagement/helpseeking behaviour)	
####	Tan (2021) (ID:87857758)	The Impact of the Coronavirus Disease (COVID-19) on the Health and Social Needs of Sex Workers in Singapore	-EXCLUDE on intervention (service engagement/helpseeking behaviour)	
####	Tang (2022) (ID:87853012)	Response Patterns to Weekly Short Message Service Health Surveys Among Diverse Youth at High Risk for Acquiring HIV	-EXCLUDE on population (no gender focus; women population <50)	
####	Tangherlini (2010) (ID:87849249)	Frequent use of emergency medical services by the elderly: a case-control study using paramedic records.	-EXCLUDE on intervention (service engagement/helpseeking behaviour)	
####	Tannis (2022) (ID:87850568)	Differences in disease prevalence among homeless and non-homeless veterans at an urban VA hospital.	-EXCLUDE on intervention (service engagement/helpseeking behaviour)	
####	Tapp (2012) (ID:87852031)	Community guide	-EXCLUDE on evidence and form (evidence not in written form or presented as research output)	
####	Tarshis (2022) (ID:87850575)	Intimate partner violence and employment-seeking: A multilevel examination of barriers and facilitators.	-EXCLUDE on intervention (service engagement/helpseeking behaviour)	
####	Tarshis (2022) (ID:87857463)	Intersectional and Trauma-Informed Approaches to Employment Services: Insights From Intimate Partner Violence (IPV) Service Providers	-INCLUDE on title & abstract	-EXCLUDE (IPV/DVA but little discussion on housing)

####	Tartakovsky (2012) (ID:87851260)	Female immigrant victims of domestic violence: A comparison between immigrants from the former Soviet Union in Israel and Israeli-born women.	-EXCLUDE on intervention (service engagement/helpseeking behaviour)	
####	Tarui (2005) (ID:87850537)	A house of their own : women and houses in Henry James's late 1890s fiction	-EXCLUDE on date (2010)	
####	Tauseef (2018) (ID:87857805)	Barriers to Recruitment of Diverse Women with Suicidality in Clinical Research	-EXCLUDE on population (no housing precarity)	
####	Tayebi (2023) (ID:87851683)	Researching with Care – Participatory Health Research with Afghan Women Refugees in Germany During the Covid-19 Pandemic: A Case with Commentaries.	-EXCLUDE on intervention (service engagement/helpseeking behaviour)	
####	TAYLOR (2012) (ID:87857321)	Incremental validity of stressful life experiences in predicting psychiatric comorbidity among women in substance abuse treatment	-EXCLUDE on intervention (service engagement/helpseeking behaviour)	
####	Taylor (2017) (ID:87858086)	Organizing Prostitution Through Silence^Discourse: Unveiling Masks of a Masquerade	-EXCLUDE on intervention (service engagement/helpseeking behaviour)	
####	Taylor (2019) (ID:87848372)	Lifetime, 5-year and past-year prevalence of homelessness in Europe: a cross-national survey in eight European nations.	-EXCLUDE on intervention (service engagement/helpseeking behaviour)	
####	Taylor (2022) (ID:87857945)	Pathways into and Out of Confinement: A Life-Course Study of Girls and School Discipline	-EXCLUDE on population (no housing precarity)	
####	Taylor (2022) (ID:88019131)	A Systematic Review and Meta-Synthesis of Barriers and Facilitators of Help- Seeking Behaviors in South Asian Women Living in High-Income Countries who Have Experienced Domestic Violence: Perception of Domestic Violence Survivors and Service Providers	-INCLUDE on title & abstract	-EXCLUDE (IPV/DVA but little discussion on housing)<br>-EXCLUDE (systematic review)* only use with other code
####	Taylor (2022) (ID:88019152)	Trauma involving violation of trust and mental health help seeking among homeless adults	-EXCLUDE on population (no gender focus; women population <50)	
####	Taylor-Clark (2010) (ID:87849258)	Communication inequalities during Public Health disasters: Katrina's wake.	-EXCLUDE on intervention (service engagement/helpseeking behaviour)	
####	TEAHAN (2006) (ID:87857319)	Help-seeking attitudes and behaviours among young people in Northern Ireland	-EXCLUDE on date (2010)	
####	**Tegan (2018) (ID:87849334)**	**Needs Assessment for Creating a Patient-Centered, Community-Engaged Health Program for Homeless Pregnant Women**	**-INCLUDE on title & abstract**	**-INCLUDE on full study**
####	TEMPLE (1995) (ID:87857163)	Refugee children in Europe	-EXCLUDE on date (2010)	
####	TEMPLE (2005) (ID:87857398)	Learning to live together: developing communities with dispersed refugee people seeking asylum	-EXCLUDE on date (2010)	
####	Tepper (2021) (ID:87857611)	Visual Arts and the Exploration of Gender Identity: A Self-Study	-EXCLUDE on population (no housing precarity)	
####	Terefe (2022) (ID:87852910)	Unmet Need for Family Planning Service and Associated Factors Among Homeless Women of Reproductive Age Group in Jimma Zone Administrative Towns, Ethiopia	-EXCLUDE on country (High-Income)	
####	Termorshuizen (2014) (ID:87848985)	Mortality and psychiatric disorders among public mental health care clients in Utrecht: a register-based cohort study.	-EXCLUDE on intervention (service engagement/helpseeking behaviour)	
####	**Teruya (2010) (ID:87849238)**	**Health and health care disparities among homeless women.**	**-INCLUDE on title & abstract**	**-INCLUDE on full study**
####	Tervonen (2017) (ID:87851647)	After accounting for competing causes of death and more advanced stage, do Aboriginal and Torres Strait Islander peoples with cancer still have worse survival? A population-based cohort study in New South Wales	-EXCLUDE on population (no housing precarity)	
####	Tesch (2020) (ID:87850675)	Best practices in shelter provision.	-INCLUDE on title & abstract	-EXCLUDE on form and evidence (not empirical)
####	Tesema (2022) (ID:87851764)	Incidence of infant mortality and its predictors in East Africa using Gompertz gamma shared frailty model.	-EXCLUDE on country (High-Income)	
####	Thakarar (2015) (ID:87848845)	Predictors of Frequent Emergency Room Visits among a Homeless Population.	-EXCLUDE on intervention (service engagement/helpseeking behaviour)	
####	Thanh (2013) (ID:87849013)	Potential impacts of the Alberta fetal alcohol spectrum disorder service networks on secondary disabilities: a cost-benefit analysis.	-EXCLUDE on intervention (service engagement/helpseeking behaviour)	
####	Thara (2021) (ID:87851608)	Gender perspectives in migration	-EXCLUDE on intervention (service engagement/helpseeking behaviour)	
####	That Is So Queer: Building... (2015) (ID:87858060)	That Is So Queer: Building a Foundation for Working With African American Lesbian, Gay, and Transgender Individuals in the Community	-EXCLUDE on population (no housing precarity)	

####	THE (2015) (ID:87857384)	Age assessment guidance: guidance to assist social workers and their managers in undertaking age assessments in England	-EXCLUDE on intervention (service engagement/helpseeking behaviour)	
####	THE (2018) (ID:87857067)	Making places work for women: gender and systems change	-INCLUDE on title & abstract	-EXCLUDE on intervention (intersectionality)
####	The Feminist Ethic of... (McCloskey) (ID:87851537)	The Feminist Ethic of Care: Mothering Among Sex Workers in Mumbai	-EXCLUDE on country (High-Income)	
####	The future of Cochrane... (Soll) (ID:87851487)	The future of Cochrane Neonatal	-EXCLUDE on intervention (service engagement/helpseeking behaviour)	
####	The Impact of the Natural,... (Coughlin) (ID:87851863)	The Impact of the Natural, Social, Built, and Policy Environments on Breast Cancer.	-EXCLUDE on population (no housing precarity)	
####	The Migration Risk Environment:... (Correa-Salazar) (ID:87851488)	The Migration Risk Environment: Challenges to Human Security for Venezuelan Migrant and Refugee Women and Girls Pre- and Post-Migration to Colombia	-EXCLUDE on country (High-Income)	
####	The Relationship between... (Garry) (ID:87851690)	The Relationship between Anticoagulation and Mortality in Pulmonary Arterial Hypertension: The Pulmonary Hypertension Association Registry (PHAR).	-EXCLUDE on population (no housing precarity)	
####	THEOBALD (2021) (ID:87857072)	Women's refuges and critical social work: opportunities and challenges in advancing social justice	-EXCLUDE on intervention (service engagement/helpseeking behaviour)	
####	**Theobald (2022) (ID:88019140)**	**Supporting Pregnant Women Experiencing Homelessness**	**-INCLUDE on title & abstract**	**-INCLUDE on full study**
####	Theuri (2016) (ID:87857617)	Black African Students and the Art and Design Education Space: Narratives of Journeys to Higher Education Art and Design	-EXCLUDE on population (no housing precarity)	
####	Thiel (2010) (ID:87849264)	Birth control sabotage and forced sex: experiences reported by women in domestic violence shelters.	-INCLUDE on title & abstract	-EXCLUDE (IPV/DVA but little discussion on housing)
####	THOMAS (2012) (ID:87851436)	An exploration of subjective wellbeing among people experiencing homelessness: a strengths-based approach	-EXCLUDE on population (women)	
####	Thomas (2015) (ID:87963238)	"I have lost everything": Trade-offs of seeking safety from intimate partner violence.	-INCLUDE on title & abstract	-EXCLUDE (IPV/DVA but little discussion on housing)
####	Thomas (2020) (ID:87857868)	BlackGirlMagic: An Exploratory Factor Analysis of the Stressors Related to Black Women's Experience of Depression	-EXCLUDE on population (no housing precarity)	
####	THOMPSON (2003) (ID:87857169)	National and regional differences among runaway youth using federally-funded crisis services	-EXCLUDE on date (2010)	
####	Thompson (2018) (ID:87858107)	Resilient Voices of Success: Counter-Narratives of Foster Youth in Graduate School	-EXCLUDE on population (women)	
####	Thompson (2023) (ID:88019143)	A Socioecological Perspective to Contraceptive Access for Women Experiencing Homelessness in the United States	-INCLUDE on title & abstract	-EXCLUDE on intervention (service engagement/helpseeking behaviour)
####	Thornhill (2010) (ID:87849271)	Creating environments of care with transgender communities.	-EXCLUDE on population (no housing precarity)	
####	Thornton (1988) (ID:87850525)	Homeless women and the law : the interpretation and implementation of Part III Housing Act 1985	-EXCLUDE on date (2010)	
####	Thulien (2022) (ID:87853445)	Effect of Portable Rent Subsidies and Mentorship on Socioeconomic Inclusion for Young People Exiting Homelessness A Community-Based Pilot Randomized Clinical Trial	-EXCLUDE on intervention (service engagement/helpseeking behaviour)	
####	Thungana (2022) (ID:87852643)	Comorbidity of mental ill-health in tuberculosis patients under treatment in a rural province of South Africa: a cross-sectional survey	-EXCLUDE on country (High-Income)	
####	TICKLE (2005) (ID:87857133)	Crossroads crisis	-EXCLUDE on date (2010)	
####	TIEU (2014) (ID:87857307)	Mental health help-seeking attitudes, utilization, and intentions among older Chinese immigrants in Canada	-EXCLUDE on population (no housing precarity)	
####	Tilahun (2020) (ID:87848280)	Common mental disorders and its associated factors and mental health care services for Ethiopian labour migrants returned from Middle East countries in Addis Ababa, Ethiopia.	-EXCLUDE on country (High-Income)	
####	Timko (2016) (ID:87848779)	Veterans' Service Utilization Patterns After Alcohol and Opioid Detoxification in VHA Care.	-EXCLUDE on population (women)	
####	TING (2009) (ID:87857299)	Barriers to help-seeking among immigrant African women survivors of partner abuse: listening to women's own voices	-EXCLUDE on date (2010)	
####	Tinland (2017) (ID:87849299)	Homeless women with schizophrenia reported lower adherence to their medication than men: results from the French Housing First experience	-EXCLUDE on population (no gender focus; women population <50)<br>-EXCLUDE - but review for literature	

####	Tinland (2018) (ID:87849293)	Victimization and posttraumatic stress disorder in homeless women with mental illness are associated with depression, suicide, and quality of life	-EXCLUDE on intervention (service engagement/helpseeking behaviour)	
####	Tinland (2020) (ID:87848259)	Effectiveness of a housing support team intervention with a recovery-oriented approach on hospital and emergency department use by homeless people with severe mental illness: a randomised controlled trial.	-EXCLUDE on population (women)	
####	Tinner (2023) (ID:87963137)	Use of Intersectionality Theory in Interventional Health Research in High-Income Countries: A Scoping Review	-EXCLUDE on intervention (service engagement/helpseeking behaviour)<br>-EXCLUDE - but review for literature	
####	TISCHLER (2008) (ID:87856945)	Resettlement and reintegration: single mothers' reflections after homelessness	-EXCLUDE on date (2010)	
####	TISCHLER (2009) (ID:87856961)	Working therapeutically with mothers who experience the trauma of homelessness: an opportunity for growth	-EXCLUDE on date (2010)	
####	Tisdale (2022) (ID:87853007)	Disparities in virtual cardiology visits among Veterans Health Administration patients during the COVID-19 pandemic	-EXCLUDE on intervention (service engagement/helpseeking behaviour)	
####	Titan (2018) (ID:87848531)	Homeless Status, Postdischarge Health Care Utilization, and Readmission After Surgery.	-EXCLUDE on population (no gender focus; women population <50)	
####	To (2015) (ID:87848891)	Healthcare Utilization, Legal Incidents, and Victimization Following Traumatic Brain Injury in Homeless and Vulnerably Housed Individuals: A Prospective Cohort Study.	-EXCLUDE on intervention (service engagement/helpseeking behaviour)	
####	Tobin (2012) (ID:87858074)	Widows and community based transitional justice in post genocide Rwanda	-EXCLUDE on country (High-Income)	
####	Tobin (2022) (ID:87852663)	Hospice care access inequalities: a systematic review and narrative synthesis	-EXCLUDE on population (no housing precarity)	
####	Tobin (2022) (ID:87852963)	Lesbian Gay Bisexual Transgender Questioning/Queer Two-Spirit Adolescents and Suicidality	-EXCLUDE on population (no housing precarity)	
####	Tohidi (2018) (ID:87853357)	Ten-Year Mortality and Revision After Total Knee Arthroplasty in Morbidly Obese Patients	-EXCLUDE on population (no housing precarity)	
####	Tomic (2022) (ID:87851577)	Lifetime risk, life expectancy, and years of life lost to type 2 diabetes in 23 high-income jurisdictions: a multinational, population-based study	-EXCLUDE on population (no housing precarity)	
####	Tomita (2012) (ID:87849107)	The impact of critical time intervention in reducing psychiatric rehospitalization after hospital discharge.	-EXCLUDE on intervention (service engagement/helpseeking behaviour)	
####	Tomita (2015) (ID:87848872)	The role of a critical time intervention on the experience of continuity of care among persons with severe mental illness after hospital discharge.	-EXCLUDE on intervention (service engagement/helpseeking behaviour)	
####	Tong (2021) (ID:87853411)	Persistent Homelessness and Violent Victimization Among Older Adults in the HOPE HOME Study	-EXCLUDE on intervention (service engagement/helpseeking behaviour)	
####	Topa (2023) (ID:87857739)	Trans Women's Body Self-Image and Health: Meanings and Impacts of Sex Work	-EXCLUDE on population (no housing precarity)	
####	Topolovec-Vranic (2013) (ID:87851158)	Clarifying the link between traumatic brain injury and homelessness: Workshop proceedings.	-EXCLUDE on intervention (service engagement/helpseeking behaviour)	
####	Topolovec-Vranic (2017) (ID:87848677)	The high burden of traumatic brain injury and comorbidities amongst homeless adults with mental illness.	-EXCLUDE on intervention (service engagement/helpseeking behaviour)<br>-EXCLUDE - but review for literature	
####	Torchalla (2011) (ID:87849190)	Smoking and predictors of nicotine dependence in a homeless population.	-EXCLUDE on population (women)	
####	Torchalla (2014) (ID:87852872)	Posttraumatic Stress Disorder and Substance Use Disorder Comorbidity in Homeless Adults: Prevalence, Correlates, and Sex Differences	-EXCLUDE on intervention (service engagement/helpseeking behaviour)	
####	Torchalla (2015) (ID:87848844)	"Like a lots happened with my whole childhood": violence, trauma, and addiction in pregnant and postpartum women from Vancouver's Downtown Eastside.	-EXCLUDE on intervention (service engagement/helpseeking behaviour)<br>-EXCLUDE - but review for literature	
####	Toro (2014) (ID:87848960)	Comparing the characteristics of homeless adults in Poland and the United States.	-EXCLUDE on intervention (service engagement/helpseeking behaviour)	

####	Tosas (2016) (ID:87848774)	Evidence for Community Transmission of Community-Associated but Not Health-Care-Associated Methicillin-Resistant Staphylococcus Aureus Strains Linked to Social and Material Deprivation: Spatial Analysis of Cross-sectional Data.	-EXCLUDE on population (women)	
####	Tosi (2018) (ID:87849319)	Homelessness and the housing factor: Learning from the debate on homelessness and poverty	-EXCLUDE on date (2010)	
####	Towns (2016) (ID:87848792)	"I Didn't Know Whether I Was Right or Wrong or Just Bewildered": Ambiguity, Responsibility, and Silencing Women's Talk of Men's Domestic Violence.	-INCLUDE on title & abstract	-EXCLUDE (IPV/DVA but little discussion on housing)
####	Tozer (2015) (ID:87848826)	Reorienting risk to resilience: street-involved youth perspectives on preventing the transition to injection drug use.	-EXCLUDE on intervention (service engagement/helpseeking behaviour)	
####	Trabut (2018) (ID:87848537)	Integrated Care for the Use of Direct-acting Antivirals in Patients With Chronic Hepatitis C and Substance Use Disorder.	-EXCLUDE on intervention (service engagement/helpseeking behaviour)	
####	Tran (2015) (ID:87848852)	Rebuilding lives and identities: The role of place in recovery among persons with complex needs.	-EXCLUDE on population (women)<br>-EXCLUDE - but review for literature	
####	Tran (2022) (ID:87850565)	"Knowing" your population: Who are we caring for at Tulane University School of Medicine's student-run free clinics?	-EXCLUDE on population (no housing precarity)	
####	Tran (2022) (ID:87857879)	Stigma experienced by people living with HIV who are on methadone maintenance treatment and have symptoms of common mental disorders in Hanoi, Vietnam: a qualitative study	-EXCLUDE on country (High-Income)	
####	TRANSGENDERING THE ACADEMY:Ensuring... (2016) (ID:87858066)	TRANSGENDERING THE ACADEMY:Ensuring Transgender Inclusion in Higher Education	-EXCLUDE on population (no housing precarity)	
####	Trapp (2015) (ID:87850971)	The relationship between dietary patterns, body mass index percentile, and household food security in young urban children.	-EXCLUDE on population (people aged under 18 years)	
####	Travelling Between The... (Pathak) (ID:87851540)	Travelling Between The Lines: Transgenders Study In Global Perspective.	-EXCLUDE on intervention (service engagement/helpseeking behaviour)	
####	Trejos (2022) (ID:87963192)	Homelessness in urban communities in the US: A Scoping Review utilizing the Socio-Ecological Model	-EXCLUDE on intervention (service engagement/helpseeking behaviour)	
####	Treloar (2016) (ID:87858104)	Multiple stigmas, shame and historical trauma compound the experience of Aboriginal Australians living with hepatitis C	-EXCLUDE on population (no housing precarity)	
####	Trepka (2013) (ID:87851684)	Rural AIDS Diagnoses in Florida: Changing Demographics and Factors Associated With Survival	-EXCLUDE on population (no housing precarity)	
####	Trickey (2017) (ID:87853209)	Survival of HIV-positive patients starting antiretroviral therapy between 1996 and 2013: a collaborative analysis of cohort studies	-EXCLUDE on population (no housing precarity)	
####	Tripathi (2018) (ID:87853081)	Association of Demographic and Socioeconomic Characteristics With Differences in Use of Outpatient Dermatology Services in the United States	-EXCLUDE on population (no housing precarity)	
####	Tripathi (2021) (ID:87851617)	Indian perspectives on homelessness and mental health	-EXCLUDE on country (High-Income)	
####	Tripathi (2022) (ID:87963233)	A systematic review of intimate partner violence interventions impacting South Asian women in the United States	-EXCLUDE on intervention (service engagement/helpseeking behaviour)<br>-EXCLUDE - but review for literature	
####	Trivedi (2020) (ID:87848253)	Evaluation of Changes in Veterans Affairs Medical Centers' Mortality Rates After Risk Adjustment for Socioeconomic Status.	-EXCLUDE on population (no housing precarity)	
####	Trivedi (2020) (ID:87853430)	Effects of Primary Sclerosing Cholangitis on Risks of Cancer and Death in People With Inflammatory Bowel Disease, Based on Sex, Race, and Age	-EXCLUDE on population (no housing precarity)	
####	Trochmann (2022) (ID:87963309)	Transforming power with Pose: Centering love in state-sponsored services for LGBTQ youth experiencing homelessness	-EXCLUDE on population (no housing precarity)	
####	Trout (2022) (ID:87851664)	In Our Own Voices: The Lived Experience of Sex Workers in Philadelphia who Identify as Women.	-INCLUDE on title & abstract	-EXCLUDE on intervention (intersectionality)

####	Trujillo (2022) (ID:87851650)	Disparities in Sexually Transmitted Infection Testing and the Need to Strengthen Comprehensive Sexual Health Services for Trans Women.	-EXCLUDE on intervention (service engagement/helpseeking behaviour)	
####	Tsai (2011) (ID:87849224)	A geographic analysis of chronically homeless adults before and after enrollment in a multi-site supported housing initiative: community characteristics and migration.	-EXCLUDE on population (women)	
####	Tsai (2012) (ID:87849099)	Outcomes of a group intensive peer-support model of case management for supported housing.	-EXCLUDE on intervention (service engagement/helpseeking behaviour)	
####	Tsai (2012) (ID:87849119)	Does housing chronically homeless adults lead to social integration?.	-EXCLUDE on population (women)	
####	Tsai (2012) (ID:87849122)	Smoking among chronically homeless adults: prevalence and correlates.	-EXCLUDE on intervention (service engagement/helpseeking behaviour)	
####	Tsai (2012) (ID:87849128)	Sobriety as an admission criterion for transitional housing: a multi-site comparison of programs with a sobriety requirement to programs with no sobriety requirement.	-EXCLUDE on intervention (service engagement/helpseeking behaviour)	
####	Tsai (2012) (ID:87849151)	Racial differences among supported housing clients in outcomes and therapeutic relationships.	-EXCLUDE on intervention (service engagement/helpseeking behaviour)	
####	Tsai (2013) (ID:87849000)	When health insurance is not a factor: national comparison of homeless and nonhomeless US veterans who use Veterans Affairs Emergency Departments.	-EXCLUDE on intervention (service engagement/helpseeking behaviour)	
####	Tsai (2013) (ID:87849062)	Obesity among chronically homeless adults: is it a problem?.	-EXCLUDE on population (no gender focus; women population <50)<br>-EXCLUDE - but review for literature	
####	Tsai (2013) (ID:87849081)	Conduct disorder behaviors, childhood family instability, and childhood abuse as predictors of severity of adult homelessness among American veterans.	-EXCLUDE on intervention (service engagement/helpseeking behaviour)	
####	Tsai (2014) (ID:87848900)	Homeless and nonhomeless VA service users likely eligible for Medicaid expansion.	-EXCLUDE on intervention (service engagement/helpseeking behaviour)	
####	Tsai (2014) (ID:87848945)	Predictors of attendance in a group-based model of case management for supported housing.	-EXCLUDE on intervention (service engagement/helpseeking behaviour)	
####	Tsai (2014) (ID:87848969)	National comparison of literally homeless male and female VA service users: entry characteristics, clinical needs, and service patterns.	-EXCLUDE on intervention (service engagement/helpseeking behaviour)	
####	Tsai (2014) (ID:87848990)	Alcohol and drug use disorders among homeless veterans: prevalence and association with supported housing outcomes.	-EXCLUDE on intervention (service engagement/helpseeking behaviour)	
####	Tsai (2014) (ID:87853035)	Homeless Female US Veterans in a National Supported Housing Program: Comparison of Individual Characteristics and Outcomes With Male Veterans	-EXCLUDE on population (women)	
####	Tsai (2015) (ID:87848851)	Violent Victimization, Mental Health, and Service Utilization Outcomes in a Cohort of Homeless and Unstably Housed Women Living With or at Risk of Becoming Infected With HIV.	-INCLUDE on title & abstract	-EXCLUDE on intervention (intersectionality)
####	Tsai (2015) (ID:87852792)	Risk Factors for Homelessness Among US Veterans	-EXCLUDE on intervention (service engagement/helpseeking behaviour)	
####	Tsai (2016) (ID:87850947)	Homelessness among a nationally representative sample of US veterans: Prevalence, service utilization, and correlates.	-EXCLUDE on population (women)	
####	Tsai (2017) (ID:87848603)	Medical-Legal Partnerships At Veterans Affairs Medical Centers Improved Housing And Psychosocial Outcomes For Vets.	-EXCLUDE on population (women)	
####	Tsai (2017) (ID:87848642)	One-year incidence and predictors of homelessness among 300,000 U.S. Veterans seen in specialty mental health care.	-EXCLUDE on intervention (service engagement/helpseeking behaviour)	
####	Tsai (2017) (ID:87848682)	Diversion of Veterans With Criminal Justice Involvement to Treatment Courts: Participant Characteristics and Outcomes.	-EXCLUDE on intervention (service engagement/helpseeking behaviour)	
####	Tsai (2017) (ID:87848683)	Partnerships Between Health Care and Legal Providers in the Veterans Health Administration.	-EXCLUDE on population (women)	
####	Tsai (2019) (ID:87850776)	Homelessness among U.S. Veterans: Critical perspectives.	-EXCLUDE on population (no gender focus; women population <50)<br>-EXCLUDE - but review for literature	
####	Tsai (2020) (ID:87848351)	Awareness of Suicide Prevention Programs Among U.S. Military Veterans.	-EXCLUDE on population (women)	

####	Tsai (2021) (ID:87848180)	Effects of Electroconvulsive Therapy on Suicidal Behavior and Emergency Department Use Among Homeless Veterans: A Propensity Score-Matched Study.	-EXCLUDE on intervention (service engagement/helpseeking behaviour)	
####	Tsai (2021) (ID:87848217)	Top 10 presenting diagnoses of homeless veterans seeking care at emergency departments.	-EXCLUDE on intervention (service engagement/helpseeking behaviour)	
####	Tsai (2021) (ID:87848222)	The Problem of Veteran Homelessness: An Update for the New Decade.	-EXCLUDE on population (women)	
####	Tsai (2021) (ID:87848224)	Mental Health and Psychosocial Characteristics Associated With COVID-19 Among U.S. Adults.	-EXCLUDE on intervention (service engagement/helpseeking behaviour)	
####	Tsai (2022) (ID:87850595)	Evictions and tenant-landlord relationships during the 2020-2021 eviction moratorium in the US.	-EXCLUDE on intervention (service engagement/helpseeking behaviour)	
####	Tsai (2022) (ID:87850619)	Psychopharmacoepidemiology of antidepressant medications among homeless and unstably housed service users in the Veterans Affairs healthcare system.	-EXCLUDE on intervention (service engagement/helpseeking behaviour)	
####	Tsai (2022) (ID:87850620)	Disentangling associations between military service, race, and incarceration in the U.S. population.	-EXCLUDE on intervention (service engagement/helpseeking behaviour)	
####	Tsai (2023) (ID:87850606)	Unmet needs of homeless U.S. veterans by gender and race/ethnicity: Data from five annual surveys.	-EXCLUDE on intervention (service engagement/helpseeking behaviour)	
####	TSANTEFSKI (2015) (ID:87857123)	A delicate balance: intervention with mothers with dual diagnosis and their infants	-INCLUDE on title & abstract	-EXCLUDE on target group (no housing precarity)
####	Tsemberis (2012) (ID:87849129)	Housing stability and recovery among chronically homeless persons with co-occuring disorders in Washington, DC.	-EXCLUDE on intervention (service engagement/helpseeking behaviour)	
####	Tsubota-Utsugi (2018) (ID:87848523)	Association between health risks and frailty in relation to the degree of housing damage among elderly survivors of the great East Japan earthquake.	-EXCLUDE on intervention (service engagement/helpseeking behaviour)	
####	Tubertini (2023) (ID:87851868)	Staff Members' Experience of Italian Shelters for LGBTQIA+ Homeless and Runaway People: An Exploratory Study	-EXCLUDE on intervention (service engagement/helpseeking behaviour)	
####	Tucker (2011) (ID:87849221)	Predictors of substance abuse treatment need and receipt among homeless women.	-INCLUDE on title & abstract	-EXCLUDE on intervention (intersectionality)
####	Tucker (2011) (ID:87849231)	Running away from home: a longitudinal study of adolescent risk factors and young adult outcomes.	-EXCLUDE on population (no gender focus; women population <50)	
####	Tucker (2018) (ID:87850811)	Factors associated with drop-in center utilization among unaccompanied youth experiencing homelessness.	-EXCLUDE on population (women)	
####	Tucker (2020) (ID:87848277)	Study protocol for a group-based motivational interviewing brief intervention to reduce substance use and sexual risk behavior among young adults experiencing homelessness.	-EXCLUDE on evidence and form (evidence not in written form or presented as research output)	
####	Tulloch (2012) (ID:87849152)	Timing, prevalence, determinants and outcomes of homelessness among patients admitted to acute psychiatric wards.	-EXCLUDE on intervention (service engagement/helpseeking behaviour)	
####	Tung (2019) (ID:87853130)	Population-Level Symptom Assessment Following Pancreaticoduodenectomy for Adenocarcinoma	-EXCLUDE on population (no housing precarity)	
####	Tung (2023) (ID:87963174)	10. No shelter from the storm: the growing challenges of housing precarity for older women during the COVID-19 pandemic	-EXCLUDE on intervention (service engagement/helpseeking behaviour)<br>-EXCLUDE - but review for literature	
####	Tunstall (2010) (ID:87849252)	Residential mobility in the UK during pregnancy and infancy: are pregnant women, new mothers and infants 'unhealthy migrants'?.	-EXCLUDE on intervention (service engagement/helpseeking behaviour)	
####	Turan (2016) (ID:87852848)	Mechanisms for the Negative Effects of Internalized HIV-Related Stigma on Antiretroviral Therapy Adherence in Women: The Mediating Roles of Social Isolation and Depression	-EXCLUDE on population (no housing precarity)	
####	TURELL (1999) (ID:87857374)	Seeking help for same-sex relationship abuses	-EXCLUDE on date (2010)	
####	Turell (2005) (ID:87853699)	Not all Alike: Within-Group Differences in Seeking Help for Same-Sex Relationship Abuses	-EXCLUDE on date (2010)	
####	Turesky (2022) (ID:87857537)	Aging Back Into the Closet: Community Planning for Older LGBTQ + Adults	-EXCLUDE on intervention (service engagement/helpseeking behaviour)	
####	Turner (2011) (ID:87849191)	The impact of needle and syringe provision and opiate substitution therapy on the incidence of hepatitis C virus in injecting drug users: pooling of UK evidence.	-EXCLUDE on intervention (service engagement/helpseeking behaviour)	

####	Turner (2011) (ID:87853154)	A Retrospective Cohort Study of the Potency of lipid-lowering therapy and Race-gender Differences in LDL cholesterol control	-EXCLUDE on population (no housing precarity)
####	Turner (2013) (ID:87849049)	Differences in HIV risk behavior of injection drug users in New York City by health care setting.	-EXCLUDE on population (women)
####	Turning off the tap:... (Walshe) (ID:87849359)	Turning off the tap: Can we do it?	-EXCLUDE on intervention (service engagement/helpseeking behaviour)
####	Twale (2016) (ID:87858012)	Conceptualizing Socialization of Graduate Students of Color: Revisiting the Weidman-Twale-Stein Framework	-EXCLUDE on intervention (service engagement/helpseeking behaviour)
####	Tweed (2022) (ID:87848143)	Premature mortality in people affected by co-occurring homelessness, justice involvement, opioid dependence, and psychosis: a retrospective cohort study using linked administrative data.	-EXCLUDE on intervention (service engagement/helpseeking behaviour)
####	TYLER (2002) (ID:87857091)	Perpetrators of early physical and sexual abuse among homeless and runaway adolescents	-EXCLUDE on date (2010)
####	Tyler (2010) (ID:87849241)	The effect of drug and sexual risk behaviours with social network and non-network members on homeless youths' sexually transmissible infections and HIV testing.	-EXCLUDE on population (no gender focus; women population <50)
####	Tyler (2013) (ID:87849036)	Homeless youths' HIV risk behaviors with strangers: Investigating the importance of social networks.	-EXCLUDE on population (people aged under 18 years)
####	Tyler (2018) (ID:87848556)	Role of Social Environmental Protective Factors on Anxiety and Depressive Symptoms Among Midwestern Homeless Youth.	-EXCLUDE on population (people aged under 18 years)
####	Tyler (2019) (ID:87853444)	Understanding Daily Depression, Drinking, and Marijuana Use Among Homeless Youth Using Short Message Service Surveying	-EXCLUDE on intervention (service engagement/helpseeking behaviour)
####	Uchimura (2013) (ID:87849012)	Characteristics and treatment outcomes of tuberculosis cases by risk groups, Japan, 2007-2010.	-EXCLUDE on intervention (service engagement/helpseeking behaviour)
####	Uddin (2012) (ID:87849112)	Strategies for providing healthcare services to street-dwellers in Dhaka city: evidence from an operations research.	-EXCLUDE on country (High-Income)
####	Uhlig (2019) (ID:87852780)	Intrahepatic Cholangiocarcinoma: Socioeconomic Discrepancies, Contemporary Treatment Approaches and Survival Trends from the National Cancer Database	-EXCLUDE on population (no housing precarity)
####	Ullah (2021) (ID:87857809)	Towards making an invisible diversity visible: A study of socially structured barriers for purple collar employees in the workplace	-EXCLUDE on population (no housing precarity)
####	Ullman (2020) (ID:87851902)	Correlates of African American sexual assault survivors' medical care seeking	-EXCLUDE on population (no housing precarity)
####	Um Estudo sobre Denúncias... (de Lima) (ID:87851592)	Um Estudo sobre Denúncias de Violência Registradas no Disque 100 - Pessoas com Deficiência.	-EXCLUDE on country (High-Income)
####	Underhill (2015) (ID:87857455)	A Qualitative Study of Medical Mistrust, Perceived Discrimination, and Risk Behavior Disclosure to Clinicians by U.S. Male Sex Workers and Other Men Who Have Sex with Men: Implications for Biomedical HIV Prevention	-EXCLUDE on population (women)
####	Ungpakorn (2020) (ID:87857483)	Health-related street outreach: Exploring the perceptions of homeless people with experience of sleeping rough	-EXCLUDE on population (women)
####	UNIVERSITY (2014) (ID:87857363)	Migrant children in foster care: seminar 4	-EXCLUDE on population (people aged under 18 years)
####	UNRAU (2005) (ID:87857373)	Exploring out-of-home placement as a moderator of help-seeking behavior among adolescents who are high at risk	-EXCLUDE on date (2010)
####	UNRAU (2006) (ID:87857223)	Connecting youth in foster care with needed mental health services lessons from research on help-seeking	-EXCLUDE on date (2010)
####	Up-to-date and projected... (Keogh) (ID:87851491)	Up-to-date and projected estimates of survival for people with cystic fibrosis using baseline characteristics: A longitudinal study using UK patient registry data	-EXCLUDE on population (no housing precarity)
####	Upadhyaya (2016) (ID:87857485)	Intersectionality and Marketplace as Utopia: Examining How Women Market Traders Manage Life Demands in Subsistence Marketplaces	-EXCLUDE on country (High-Income)
####	Upadhyaya (2021) (ID:87849362)	Evolving two-generation services to disrupt the intergenerational effects of poverty and promote family well-being	-EXCLUDE on population (women)

####	Upshur (2014) (ID:87848907)	Homeless women and hazardous drinking: screening results in a primary health care setting.	-EXCLUDE on intervention (service engagement/helpseeking behaviour)	
####	Upshur (2015) (ID:87848875)	A randomized control trial of a chronic care intervention for homeless women with alcohol use problems.	-INCLUDE on title & abstract	-EXCLUDE on intervention (too programme-specific; not generalisable)
####	Upshur (2017) (ID:87848625)	Prevalence and predictors of substance use disorders among homeless women seeking primary care: An 11 site survey.	-INCLUDE on title & abstract	-EXCLUDE on intervention (service engagement/helpseeking behaviour)
####	Upshur (2018) (ID:87848586)	Homeless women's service use, barriers, and motivation for participating in substance use treatment.	-INCLUDE on title & abstract	-EXCLUDE on intervention (intersectionality)
####	Urada (2018) (ID:87848530)	Sex Trade and Health Care Utilization Among People Living with HIV/AIDS.	-EXCLUDE on population (women)	
####	Ursula (2023) (ID:87851460)	Food insecurity in households with persons with disabilities in a situation of extreme vulnerability in Brazil: A secondary cross-sectional analysisResearch in context	-EXCLUDE on country (High-Income)	
####	Use of SERTS (Socio-Economic,... (Vercelli) (ID:87851492)	Use of SERTS (Socio-Economic, health Resources and Technologic Supplies) models to estimate cancer survival at provincial geographical level	-EXCLUDE on population (no housing precarity)	
####	Using Best-Worst Scaling... (Wittenberg) (ID:87851692)	Using Best-Worst Scaling to Understand Patient Priorities: A Case Example of Papanicolaou Tests for Homeless Women.	-EXCLUDE on intervention (service engagement/helpseeking behaviour)<br>-EXCLUDE - but review for literature	
####	Uwe (2016) (ID:87851559)	Dermatologic challenges of health care for displaced people. lessons from a German emergency refugee camp	-EXCLUDE on population (women)	
####	Vacchelli (2021) (ID:87963118)	Diversity as discourse and diversity as practice: critical reflections on migrant women's experiences of accessing mental health support in London	-EXCLUDE on intervention (service engagement/helpseeking behaviour)	
####	Vaitses (2019) (ID:87850759)	HIV-related care for transgender people: A systematic review of studies from around the world.	-EXCLUDE on intervention (service engagement/helpseeking behaviour)	
####	Vakkalanka (2021) (ID:87848183)	Mobile Crisis Outreach and Emergency Department Utilization: A Propensity Score-matched Analysis.	-EXCLUDE on intervention (service engagement/helpseeking behaviour)	
####	Valente (2013) (ID:87849016)	Gender differences in sexual risk and sexually transmitted infections correlate with gender differences in social networks among San Francisco homeless youth.	-EXCLUDE on intervention (service engagement/helpseeking behaviour)	
####	Valentich (2011) (ID:87858006)	On Being and Calling Oneself a Feminist Social Worker	-EXCLUDE on intervention (service engagement/helpseeking behaviour)	
####	Valentine (2020) (ID:87963177)	Inquiry into integrated housing support for vulnerable families	-EXCLUDE on intervention (service engagement/helpseeking behaviour)	
####	Valentine (2023) (ID:87857854)	Coaching to Completion: Impacts of Success Coaching on Community College Student Attainment in North Carolina	-EXCLUDE on population (no housing precarity)	
####	Valerio (2022) (ID:87849350)	Declining prevalence of current HCV infection and increased treatment uptake among people who inject drugs: The ETHOS Engage study	-EXCLUDE on intervention (service engagement/helpseeking behaviour)	
####	Vallance (2016) (ID:87848748)	Do managed alcohol programs change patterns of alcohol consumption and reduce related harm? A pilot study.	-EXCLUDE on intervention (service engagement/helpseeking behaviour)	
####	VALLE (2004) (ID:87857235)	Ethnic differences in social network help-seeking strategies among Latino and Euro-American dementia caregivers	-EXCLUDE on date (2010)	
####	Vallersnes (2018) (ID:87848498)	Factors associated with rapidly repeated acute poisoning by substances of abuse: a prospective observational cohort study.	-EXCLUDE on intervention (service engagement/helpseeking behaviour)	
####	Valles (2020) (ID:87857996)	The Predictable Inequities of COVID-19 in the US: Fundamental Causes and Broken Institutions	-EXCLUDE on population (no housing precarity)	
####	Van (1999) (ID:87857233)	Women's help-seeking patterns for depression	-EXCLUDE on date (2010)	
####	Van (2000) (ID:87857146)	Help seeking for violence: views of survivors	-EXCLUDE on date (2010)	
####	Van Berkum (2019) (ID:87963032)	'Where to now?'Understanding the landscape of health and social services for homeless women in London, Ontario, Canada	-INCLUDE on title & abstract	-EXCLUDE on intervention (intersectionality)
####	Van Dam (2018) (ID:87850827)	Does natural mentoring matter? A multilevel meta-analysis on the association between natural mentoring and youth outcomes.	-EXCLUDE on intervention (service engagement/helpseeking behaviour)	
####	van den Berk-Clark (2014) (ID:87848916)	Trust in health care providers: factors predicting trust among homeless veterans over time.	-EXCLUDE on population (women)	

####	van der Laan (2018) (ID:87848585)	Predicting homeless people's perceived health after entering the social relief system in The Netherlands.	-EXCLUDE on intervention (service engagement/helpseeking behaviour)	
####	van der Laan (2020) (ID:87850700)	Differences in housing transitions and changes in health and self-determination between formerly homeless individuals.	-EXCLUDE on population (no gender focus; women population <50)	
####	van der Plas (2012) (ID:87849148)	Perceptions of quality of life and disability in homeless persons with schizophrenia and persons with schizophrenia living in non-institutional housing.	-EXCLUDE on intervention (service engagement/helpseeking behaviour)	
####	van der Zee (2023) (ID:87851452)	Effect of the introduction of screening for cancer precursor lesions on anal cancer incidence over time in people living with HIV: a nationwide cohort study	-EXCLUDE on population (no housing precarity)	
####	van Dongen (2019) (ID:87848418)	Self-reported health, healthcare service use and health-related needs: A comparison of older and younger homeless people.	-EXCLUDE on population (no gender focus; women population <50)	
####	van Dongen (2022) (ID:87853188)	Individual-level income and out-of-hospital cardiac arrest survival in men and women	-EXCLUDE on population (no housing precarity)	
####	Van Patten (2020) (ID:87848299)	Accuracy of case managers in estimating intelligence quotients and functional status of people experiencing homelessness.	-EXCLUDE on intervention (service engagement/helpseeking behaviour)	
####	Van Straaten (2017) (ID:87848700)	Dutch homeless people 2.5 years after shelter admission: what are predictors of housing stability and housing satisfaction?.	-EXCLUDE on intervention (service engagement/helpseeking behaviour)	
####	Van Straaten (2017) (ID:87848704)	Self-reported care needs of Dutch homeless people with and without a suspected intellectual disability: a 1.5-year follow-up study.	-EXCLUDE on population (women)	
####	Van Straaten (2018) (ID:87853376)	Changes in Social Exclusion Indicators and Psychological Distress Among Homeless People Over a 2.5-Year Period	-EXCLUDE on population (women)	
####	van Vugt (2012) (ID:87849130)	Consumer-providers in assertive community treatment programs: associations with client outcomes.	-EXCLUDE on intervention (service engagement/helpseeking behaviour)	
####	Vandermeade (2020) (ID:87857942)	Using the Master's Tools to Dismantle the Master's House: White Women's Gendered and Racialized Citizenship, Pro-Immigrants' Rights Advocacy, and White Privilege in the Borderlands	-EXCLUDE on population (no housing precarity)	
####	Vandyk (2013) (ID:87851178)	Frequent emergency department use by individuals seeking mental healthcare: A systematic search and review.	-EXCLUDE on population (no housing precarity)	
####	Vardazaryan (2016) (ID:87857958)	Gender, migration, and practices of political participation in lethbridge	-EXCLUDE on intervention (service engagement/helpseeking behaviour)	
####	Varga (2014) (ID:87852672)	Predicting Health Care Utilization in Marginalized Populations: Black, Female, Street-Based Sex Workers	-INCLUDE on title & abstract	-EXCLUDE on intervention (service engagement/helpseeking behaviour)
####	Varma (2015) (ID:87850973)	Characteristics of child commercial sexual exploitation and sex trafficking victims presenting for medical care in the United States.	-EXCLUDE on population (people aged under 18 years)	
####	Vasil (2023) (ID:87963201)	"I Came Here, and it Got Worse Day by Day": Examining the Intersections Between Migrant Precarity and Family Violence Among Women with Insecure Migration Status in Australia	-INCLUDE on title & abstract	-EXCLUDE (IPV/DVA but little discussion on housing)
####	Vaughan (2015) (ID:87963028)	Promoting community-led responses to violence against immigrant and refugee women in metropolitan and regional Australia	-INCLUDE on title & abstract	-EXCLUDE (IPV/DVA but little discussion on housing)
####	Vaughan (2020) (ID:87963185)	Multicultural and Settlement services Supporting women experiencing violence	-INCLUDE on title & abstract	-EXCLUDE (IPV/DVA but little discussion on housing)
####	Vazquez (2017) (ID:87848703)	Actors, observers, and causal attributions of homelessness: Differences in attribution for the causes of homelessness among domiciled and homeless people in Madrid (Spain).	-EXCLUDE on intervention (service engagement/helpseeking behaviour)	
####	Vazquez (2019) (ID:87853185)	The Particularly Vulnerable Situation of Women Living Homeless in Madrid (Spain)	-INCLUDE on title & abstract	-EXCLUDE on intervention (intersectionality)
####	Vazquez (2022) (ID:87852962)	The vulnerability of women living homeless in Nicaragua: A comparison between homeless women and men in a low-income country	-EXCLUDE on country (High-Income)	
####	Vazquez (2022) (ID:87853075)	Role of stressful life events in homelessness in Nicaragua: an intragroup analysis in a low-income country	-EXCLUDE on country (High-Income)	

####	Vazquez (2022) (ID:87853396)	Access to employment and the labor market among women living homeless in Madrid, Spain	-INCLUDE on title & abstract	-EXCLUDE on intervention (intersectionality)
####	Vazquez (2022) (ID:87853428)	Characteristics and needs of people living homeless in Leon (Nicaragua): Similarities and differences with other groups in severe social exclusion	-EXCLUDE on country (High-Income)	
####	Vecho (2011) (ID:87853347)	Division of parental labor in french lesbian mother families created by anonymous donor insemination	-EXCLUDE on population (no housing precarity)	
####	Vedam (2019) (ID:87963311)	The Giving Voice to Mothers study: inequity and mistreatment during pregnancy and childbirth in the United States	-EXCLUDE on population (no housing precarity)	
####	Veen (2023) (ID:87852990)	Adherence to the physical activity guideline beyond the recommended minimum weekly amount: impacts on indicators of physical function in older adults	-EXCLUDE on population (no housing precarity)	
####	Veidemane (2021) (ID:87858109)	Inclusive Higher Education Access for Underrepresented Groups: It Matters, But How Can Universities Measure It?	-EXCLUDE on population (no housing precarity)	
####	Velazquez-Millings (2017) (ID:87857896)	A Quantitative Examination of Client Based Factors that Influence Completion Rates in Substance Abuse Treatment	-EXCLUDE on population (no housing precarity)	
####	Velez (2017) (ID:87848679)	"It's been an Experience, a Life Learning Experience": A Qualitative Study of Hospitalized Patients with Substance Use Disorders.	-EXCLUDE on population (women)	
####	Velez-Giraldo (2022) (ID:87857629)	The Unique Experiences of Latinx Female Veterans	-EXCLUDE on population (no housing precarity)	
####	Velonis (2018) (ID:87848554)	"One program that could improve health in this neighbourhood is ____?" using concept mapping to engage communities as part of a health and human services needs assessment.	-EXCLUDE on intervention (service engagement/helpseeking behaviour)	
####	Venables (2019) (ID:87853210)	Nationwide Incidence of Metastatic Cutaneous Squamous Cell Carcinoma in England	-EXCLUDE on population (no housing precarity)	
####	Ventriglio (2022) (ID:87850578)	New approaches for mental health of social minorities.	-EXCLUDE on population (no gender focus; women population <50)<br>-EXCLUDE - but review for literature	
####	Verhaeghe (2012) (ID:87849113)	Health promotion intervention in mental health care: design and baseline findings of a cluster preference randomized controlled trial.	-EXCLUDE on intervention (service engagement/helpseeking behaviour)	
####	Verhaeghe (2013) (ID:87849017)	Health promotion in individuals with mental disorders: a cluster preference randomized controlled trial.	-EXCLUDE on intervention (service engagement/helpseeking behaviour)	
####	**Versey (2022) (ID:87963040)**	**The impact of COVID-19 and housing insecurity on lower-income Black women**	**-INCLUDE on title & abstract**	**-INCLUDE on full study**
####	Vian (2017) (ID:87848649)	Willingness to Pay for a Maternity Waiting Home Stay in Zambia.	-EXCLUDE on country (High-Income)	
####	Vichta (2017) (ID:87963339)	Intimate partner violence and homelessness: Young women lost in the intersectionality	-EXCLUDE on intervention (service engagement/helpseeking behaviour)	
####	Vickery (2020) (ID:87848362)	Integrated, Accountable Care For Medicaid Expansion Enrollees: A Comparative Evaluation of Hennepin Health.	-EXCLUDE on intervention (service engagement/helpseeking behaviour)	
####	**Vidales (2010) (ID:87851383)**	**Arrested justice: The multifaceted plight of immigrant Latinas who faced domestic violence.**	**-INCLUDE on title & abstract**	**-INCLUDE on full study**
####	Viergever (2018) (ID:87848496)	Supporting ALL victims of violence, abuse, neglect or exploitation: guidance for health providers.	-EXCLUDE on population (no gender focus; women population <50)	
####	**Viergever (2019) (ID:87848452)**	**The recovery experience of people who were sex trafficked: the thwarted journey towards goal pursuit.**	**-INCLUDE on title & abstract**	**-INCLUDE on full study**
####	Vijayaraghavan (2012) (ID:87852661)	Health, Access to Health Care, and Health Care use Among Homeless Women with a History of Intimate Partner Violence	-EXCLUDE on intervention (service engagement/helpseeking behaviour)	
####	Vijayaraghavan (2013) (ID:87852683)	Housing Instability and Incident Hypertension in the CARDIA Cohort	-EXCLUDE on intervention (service engagement/helpseeking behaviour)	
####	Vijayaraghavan (2014) (ID:87848919)	Non-medical use of non-opioid psychotherapeutic medications in a community-based cohort of HIV-infected indigent adults.	-EXCLUDE on intervention (service engagement/helpseeking behaviour)	
####	Vila-Rodriguez (2013) (ID:87849010)	The hotel study: multimorbidity in a community sample living in marginal housing.	-EXCLUDE on intervention (service engagement/helpseeking behaviour)	
####	Village news (2017) (ID:87852005)	Village news	-EXCLUDE on evidence and form (evidence not in written form or presented as research output)	

####	Villalobos (2019) (ID:87858084)	Reintegration Experiences of OEF/OIF/OND Veterans in the U.S.-Mexico Border Region: An Interpretative Phenomenological Analysis	-EXCLUDE on intervention (service engagement/helpseeking behaviour)	
####	Villena (2010) (ID:87849269)	Challenges and struggles: lived experiences of individuals with co-occurring disorders.	-EXCLUDE on population (no gender focus; women population <50)	
####	Vipler (2018) (ID:87848517)	Use of withdrawal management services among people who use illicit drugs in Vancouver, Canada.	-EXCLUDE on intervention (service engagement/helpseeking behaviour)	
####	Viswanathan (2016) (ID:87857680)	A Bottom-Up Approach to Short-Term Immersion in Subsistence Marketplaces: Methodological and Substantive Lessons on Poverty and the Environment From Tanzania	-EXCLUDE on country (High-Income)	
####	Vivian (2018) (ID:87851526)	Predicting 3-Year Survival in Patients Receiving Maintenance Dialysis: An External Validation of iChoose Kidney in Ontario, Canada	-EXCLUDE on population (no gender focus; women population <50)	
####	Vognsgaard (2022) (ID:87851549)	Associations between smoking status and involvement of personal and professional relations among individuals reporting symptoms related to a diagnosis of lung cancer: a population-based study	-EXCLUDE on population (no housing precarity)	
####	von Aesch (2021) (ID:87848212)	Family medicine-directed hepatitis C care and barriers to treatment: a mixed-methods study.	-EXCLUDE on intervention (service engagement/helpseeking behaviour)	
####	Voon (2014) (ID:87848950)	Self-management of pain among people who inject drugs in Vancouver.	-EXCLUDE on population (no gender focus; women population <50)	
####	Voronka (2019) (ID:87963113)	The mental health peer worker as informant: Performing authenticity and the paradoxes of passing	-EXCLUDE on intervention (service engagement/helpseeking behaviour)	
####	Voss (2007) (ID:87853694)	Symptom Cluster of Fatigue and Depression in HIV/AIDS	-EXCLUDE on date (2010)	
####	Voth (2018) (ID:87857635)	Service Use and Needs Among Female Survivors of Intimate Partner Violence Attending Community College	-EXCLUDE on population (no housing precarity)	
####	Voth (2021) (ID:87963391)	Experiences with help seeking among non–service-engaged survivors of IPV: Survivors' recommendations for service providers	-INCLUDE on title & abstract	-EXCLUDE (IPV/DVA but little discussion on housing)
####	Vuillermoz (2016) (ID:87849291)	Mortality among homeless people in France, 2008-10	-EXCLUDE on intervention (service engagement/helpseeking behaviour)	
####	Vuillermoz (2017) (ID:87848612)	Unmet healthcare needs in homeless women with children in the Greater Paris area in France.	-INCLUDE on title & abstract	-EXCLUDE on intervention (service engagement/helpseeking behaviour)
####	Vuillermoz (2017) (ID:87852695)	Cervical cancer screening among homeless women in the Greater Paris Area (France): results of the ENFAMS survey	-EXCLUDE on intervention (service engagement/helpseeking behaviour)	
####	Vyas (2021) (ID:87857778)	A qualitative exploration of stigma experiences of second-generation British South-Asian people using an early intervention in psychosis service	-EXCLUDE on population (no housing precarity)	
####	WACHTER (2015) (ID:87856938)	Predictors of multiple arrests among homeless young adults: gender differences	-EXCLUDE on intervention (service engagement/helpseeking behaviour)<br>-EXCLUDE - but review for literature	
####	Wada (2019) (ID:87857787)	Affirmative Career Counselling with Transgender and Gender Nonconforming Clients: A Social Justice Perspective	-EXCLUDE on intervention (service engagement/helpseeking behaviour)	
####	WADE (2005) (ID:87857219)	Unaccompanied asylum seeking children: the response of social work services	-EXCLUDE on date (2010)	
####	WADE (2019) (ID:87857026)	Supporting unaccompanied asylum-seeking young people: the experience of foster care	-EXCLUDE on population (people aged under 18 years)	
####	Wadhera (2019) (ID:87852753)	Trends, Causes, and Outcomes of Hospitalizations for Homeless Individuals A Retrospective Cohort Study	-EXCLUDE on intervention (service engagement/helpseeking behaviour)	
####	Wadhera (2020) (ID:87848344)	Disparities in Care and Mortality Among Homeless Adults Hospitalized for Cardiovascular Conditions.	-EXCLUDE on population (no gender focus; women population <50)	
####	Wagaman (2013) (ID:87857928)	Exploring Intersections of Identity and Service Provision Among LGBTQ Young Adults: A Participatory Action Research Approach	-INCLUDE on title & abstract	-EXCLUDE on target group (no housing precarity)
####	**Wagaman (2014) (ID:87857816)**	**Understanding Service Experiences of LGBTQ Young People Through an Intersectional Lens**	**-INCLUDE on title & abstract**	**-INCLUDE on full study**
####	Wagaman (2016) (ID:87858081)	Self-definition as resistance: Understanding identities among LGBTQ emerging adults	-EXCLUDE on population (no housing precarity)	
####	Wagner (2015) (ID:87851008)	Good mom.	-EXCLUDE on evidence and form (evidence not in written form or presented as research output)	

####	Wagner (2020) (ID:87963190)	Addictive behaviors during emerging adulthood	-EXCLUDE on intervention (service engagement/helpseeking behaviour)	
####	Wagner (2021) (ID:87857855)	Student Perceptions of Institutional Care: Making Sense of Hardship Funding as a Retention Tool	-EXCLUDE on population (no housing precarity)	
####	Wakgari (2020) (ID:87848311)	Sexually transmitted disease among street dwellers in southern Ethiopia: a mixed methods study design.	-EXCLUDE on country (High-Income)	
####	Wald (1997) (ID:87850531)	Homelessness among young women leaving care : an exploratory study	-EXCLUDE on date (2010)	
####	Waldron (2020) (ID:87963379)	Black women's experiences with mental illness, help-seeking & coping in the halifax regional municipality: A study conducted to inform NSHA's Nova Scotia Sisterhood Initiative [Doctoral dissertation] Dalhousie University	-EXCLUDE on population (no housing precarity)	
####	Waldron (2021) (ID:87963038)	Hear my cry: Breaking the code of silence around intimate partner violence among Black women in and beyond midlife	-INCLUDE on title & abstract	-EXCLUDE (IPV/DVA but little discussion on housing)
####	WALES (2006) (ID:87857038)	Costs and benefits of the supporting people programme	-EXCLUDE on date (2010)	
####	WALES (2006) (ID:87857039)	Costs and benefits of the supporting people programme: executive summary	-EXCLUDE on date (2010)	
####	WALES (2011) (ID:87857209)	Safeguarding and promoting the welfare of unaccompanied asylum seeking children and young people: all Wales practice guidance	-EXCLUDE on population (people aged under 18 years)	
####	WALES (2017) (ID:87857135)	Exploring barriers to South Asian help-seeking for eating disorders	-EXCLUDE on population (no housing precarity)	
####	WALES (2017) (ID:87857401)	"I used to be someone": refugees and asylum seekers in Wales	-EXCLUDE on intervention (service engagement/helpseeking behaviour)	
####	WALKER (2017) (ID:87857362)	Addressing the challenges of aging how elders and their care partners seek information	-EXCLUDE on population (no housing precarity)	
####	Walker (2021) (ID:87857757)	Rape, inequality and the criminal justice response in England: The importance of age and gender	-EXCLUDE on population (no housing precarity)	
####	Walker (2022) (ID:87852789)	The Impact of an Educational Intervention on Nursing Staff Attitudes Toward Patients Experiencing Homelessness and Mental Illness	-EXCLUDE on intervention (service engagement/helpseeking behaviour)	
####	Wall (2021) (ID:87857453)	CATCHING BULLETS WITH HER ASS: Matrilineality and the Canadian Dub Poetry Tradition in the Work of d'bi.young anitafrika	-EXCLUDE on evidence and form (evidence not in written form or presented as research output)	
####	Wallace (2018) (ID:87848572)	Sheltering risks: Implementation of harm reduction in homeless shelters during an overdose emergency.	-EXCLUDE on intervention (service engagement/helpseeking behaviour)	
####	Waller (2018) (ID:87963394)	An examination of the help seeking behaviors of African American women with adverse childhood experiences	-EXCLUDE on population (no housing precarity)	
####	Waller (2022) (ID:87963389)	Caught in the crossroad: An intersectional examination of African American women intimate partner violence survivors' help seeking	-INCLUDE on title & abstract	-EXCLUDE on evidence and form (systematic review)
####	Waller (2022) (ID:88019145)	Sarah Waller's Help-Seeking Model: Understanding African American Women Intimate Partner Violence Survivors' Help-seeking Process	-INCLUDE on title & abstract	-EXCLUDE (IPV/DVA but little discussion on housing)
####	Waller (2023) (ID:87857424)	"I Am the One That Needs Help": The Theory of Help-Seeking Behavior for Survivors of Intimate Partner Violence	-INCLUDE on title & abstract	-EXCLUDE (IPV/DVA but little discussion on housing)
####	Waller (2023) (ID:87857590)	"I Have to Fight to Get Out": African American Women Intimate Partner Violence Survivors' Construction of Agency	-INCLUDE on title & abstract	-EXCLUDE (IPV/DVA but little discussion on housing)
####	Walley (2015) (ID:87848825)	Engagement and Substance Dependence in a Primary Care-Based Addiction Treatment Program for People Infected with HIV and People at High-Risk for HIV Infection.	-EXCLUDE on intervention (service engagement/helpseeking behaviour)	
####	WALLS (2008) (ID:87857184)	Suicidal ideation and attempts among sexual minority youths receiving social services	-EXCLUDE on date (2010)	
####	Walls (2010) (ID:87851368)	Correlates of cutting behavior among sexual minority youths and young adults.	-EXCLUDE on intervention (service engagement/helpseeking behaviour)	
####	Walsh (2009) (ID:87849341)	Characteristics of Home: Perspectives of Women Who Are Homeless	-EXCLUDE on date (2010)	
####	Walter (2016) (ID:87848793)	Two pathways through adversity: Predicting well-being and housing outcomes among homeless service users.	-EXCLUDE on intervention (service engagement/helpseeking behaviour)	

####	WALTERS (2008) (ID:87857251)	Help-seeking preferences for psychological distress in primary care: effect of current mental state	-EXCLUDE on date (2010)	
####	Walters (2010) (ID:87851370)	Invisible at every turn an examination of lesbian intimate partner violence.	-INCLUDE on title & abstract	-EXCLUDE (IPV/DVA but little discussion on housing)
####	Wang (2015) (ID:87848843)	Food insecurity and health: data from the Veterans Aging Cohort Study.	-EXCLUDE on intervention (service engagement/helpseeking behaviour)	
####	Wang (2017) (ID:87853333)	The Association Between Immigration Status and Office-based Medical Provider Visits for Cancer Patients in the United States	-EXCLUDE on intervention (service engagement/helpseeking behaviour)	
####	Wang (2017) (ID:87857726)	We Need Help in the Delta: Barriers to Health Promotion Among Older African American Men in the Mississippi Delta	-EXCLUDE on population (women)	
####	Wang (2020) (ID:87848286)	Correlates of Depressive Symptoms among Middle-Aged and Older Homeless Adults Using the 9-Item Patient Health Questionnaire.	-EXCLUDE on intervention (service engagement/helpseeking behaviour)	
####	Wang (2021) (ID:87848225)	Examining Health Care Mobility of Transgender Veterans Across the Veterans Health Administration.	-EXCLUDE on intervention (service engagement/helpseeking behaviour)	
####	Wang (2021) (ID:87853454)	The Homeless People in China During the COVID-19 Pandemic: Victims of the Strict Pandemic Control Measures of the Government	-EXCLUDE on country (High-Income)	
####	Warburton (2022) (ID:88019132)	Heterogeneity among Homeless Australian Women and Their Reasons for Homelessness Entry	-INCLUDE on title & abstract	-EXCLUDE on intervention (service engagement/helpseeking behaviour)
####	Ward (2013) (ID:87851953)	REFLECTIONS ON COMMUNITY JUSTICE IN MONTREAL	-EXCLUDE on evidence and form (evidence not in written form or presented as research output)	
####	Wardhaugh (2012) (ID:87849378)	Policies to Address Homelessness	-EXCLUDE on intervention (service engagement/helpseeking behaviour)	
####	Ware (2016) (ID:87848749)	Characteristics influencing attendance at a primary care health check for people with intellectual disability: An individual participant data meta-analysis.	-EXCLUDE on population (no housing precarity)	
####	Warren (2022) (ID:87857676)	Weaponizing COVID-19: How the Pandemic Influenced the Behavior of Those Who Use Violence in Domestic and Family Relationships	-EXCLUDE on intervention (service engagement/helpseeking behaviour)	
####	Washington (2010) (ID:87849274)	Risk factors for homelessness among women veterans.	-EXCLUDE on intervention (service engagement/helpseeking behaviour)	
####	Washington (2013) (ID:87849067)	Self-efficacy as a unifying construct in nursing-social work collaboration with vulnerable populations.	-EXCLUDE on intervention (service engagement/helpseeking behaviour)<br>-EXCLUDE - but review for literature	
####	Washington (2021) (ID:87963135)	Strength Under Pressure: Superwoman Schema (SWS) and Intimate Partner Violence (IPV) among Black Women	-INCLUDE on title & abstract	-EXCLUDE (IPV/DVA but little discussion on housing)
####	Washington-Brown (2021) (ID:87851546)	Advancing the health of homeless populations through vaccinations.	-EXCLUDE on population (no gender focus; women population <50)	
####	Wathen (2015) (ID:87851030)	What counts? A mixed-methods study to inform evaluation of shelters for abused women.	-EXCLUDE on intervention (service engagement/helpseeking behaviour)	
####	Watkins (2018) (ID:87848511)	Predictors of treatment initiation for alcohol use disorders in primary care.	-EXCLUDE on population (women)	
####	Watkins-Hayes (2013) (ID:87963384)	The micro dynamics of support seeking: The social and economic utility of institutional ties for HIV-positive women	-EXCLUDE on population (no housing precarity)<br>-EXCLUDE - but review for literature	
####	Watson (1983) (ID:87850516)	Homeless women : a social, economic and historical analysis	-EXCLUDE on date (2010)<br>-EXCLUDE - but review for literature	
####	Watson (2013) (ID:87851203)	Understanding the critical ingredients for facilitating consumer change in Housing First programming: A case study approach.	-EXCLUDE on intervention (service engagement/helpseeking behaviour)	
####	Watson (2015) (ID:87857802)	Therapists' integration of spirituality and religion into the counseling of African-American women clients	-EXCLUDE on population (no housing precarity)	
####	Watson (2016) (ID:87848740)	Social exclusion, health and hidden homelessness.	-EXCLUDE on population (no gender focus; women population <50)	
####	Watson (2017) (ID:87963156)	Youth homelessness and survival sex: Intimate relationships and gendered subjectivities	-EXCLUDE on intervention (service engagement/helpseeking behaviour)<br>-EXCLUDE - but review for literature	

####	Watson (2021) (ID:87963092)	AFRICAN SELF-CONSCIOUSNESS, CULTURAL MISTRUST, AND AFRICAN AMERICANS'HELP-SEEKING ATTITUDES TOWARD MENTAL HEALTHCARE UTILIZATION: AN EXPLORATION	-EXCLUDE on population (no housing precarity)	
####	Watt (2012) (ID:87852034)	Homeless find hope in Cambridge core	-EXCLUDE on evidence and form (evidence not in written form or presented as research output)	
####	Watters (2016) (ID:87848717)	Mental health and psychosocial interventions for children and adolescents in street situations in low- and middle-income countries: A systematic review.	-EXCLUDE on country (High-Income)	
####	Waxmonsky (2014) (ID:87848976)	Enhanced fidelity to treatment for bipolar disorder: results from a randomized controlled implementation trial.	-EXCLUDE on intervention (service engagement/helpseeking behaviour)	
####	Wearing (2011) (ID:87851351)	Strengthening youth citizenship and social inclusion practice-The Australian case: Towards rights based and inclusive practice in services for marginalized young people.	-EXCLUDE on intervention (service engagement/helpseeking behaviour)	
####	Weatherall (2021) (ID:87851885)	"I don't have an address": Housing instability and domestic violence in help-seeking calls to a support service	-INCLUDE on title & abstract	-EXCLUDE on intervention (service engagement/helpseeking behaviour)
####	Webb (2010) (ID:87849243)	Recruitment and retention of women in a large randomized control trial to reduce repeat preterm births: the Philadelphia Collaborative Preterm Prevention Project.	-EXCLUDE on intervention (service engagement/helpseeking behaviour)	
####	Webb (2023) (ID:87963184)	Absent and Problematic: The Representation of Fathers in the Program Policies of Organizations that Provide Family-Centred Services in Vancouver's Downtown Eastside	-EXCLUDE on population (no housing precarity)	
####	WEBBER (2015) (ID:87857252)	Engaging in cyberspace: seeking help for sexual assault	-EXCLUDE on intervention (service engagement/helpseeking behaviour)	
####	Webel (2013) (ID:87849034)	The impact of social context on self-management in women living with HIV.	-INCLUDE on title & abstract	-EXCLUDE on target group (no housing precarity)
####	Weber (2013) (ID:87849011)	Perception of access to health care by homeless individuals seeking services at a day shelter.	-EXCLUDE on population (no gender focus; women population <50)	
####	Weber (2019) (ID:87850758)	A systematic review of nurse-led interventions with populations experiencing homelessness.	-EXCLUDE on intervention (service engagement/helpseeking behaviour)	
####	Wei (2013) (ID:87849040)	Service utilization for mental problems in a metropolitan migrant population in china.	-EXCLUDE on country (High-Income)	
####	Weichsel (2005) (ID:87849363)	Risk factors for adult victimization among Florida's homeless women	-EXCLUDE on date (2010)	
####	Weinreb (2007) (ID:87853695)	Integrating Behavioral Health Services for Homeless Mothers and Children in Primary Care	-EXCLUDE on date (2010)	
####	Weinstein (2013) (ID:87849030)	A primary care-public health partnership addressing homelessness, serious mental illness, and health disparities.	-EXCLUDE on intervention (service engagement/helpseeking behaviour)	
####	Weinstein (2020) (ID:87848321)	Retention of Patients With Multiple Vulnerabilities in a Federally Qualified Health Center Buprenorphine Program: Pennsylvania, 2017-2018.	-EXCLUDE on intervention (service engagement/helpseeking behaviour)	
####	Weiser (2013) (ID:87851145)	Food insecurity and HIV clinical outcomes in a longitudinal study of urban homeless and marginally housed HIV-infected individuals.	-EXCLUDE on population (women)	
####	Weiser (2013) (ID:87851207)	Food insecurity is associated with greater acute care utilization among HIV-Infected homeless and marginally housed individuals in San Francisco.	-EXCLUDE on intervention (service engagement/helpseeking behaviour)	
####	WEISS-GRINSTEIN (2005) (ID:87857239)	Gender and ethnic differences in formal and informal help seeking among Israeli adolescents	-EXCLUDE on date (2010)	
####	Weisz (2018) (ID:87963280)	Stigmatized identities, psychological distress, and physical health: Intersections of homelessness and race.	-EXCLUDE on population (no gender focus; women population <50)	
####	Welch (2011) (ID:87851309)	What is the role of systematic reviews in tackling health inequity?	-EXCLUDE on intervention (service engagement/helpseeking behaviour)	
####	Wells (2013) (ID:87849031)	Community-partnered cluster-randomized comparative effectiveness trial of community engagement and planning or resources for services to address depression disparities.	-EXCLUDE on intervention (service engagement/helpseeking behaviour)	
####	Wemakor (2023) (ID:87852730)	Determinants of Household Food Insecurity and Depression in Mothers: Evidence from Ghana	-EXCLUDE on country (High-Income)	
####	Wen (2018) (ID:87853324)	Demographic and Urbanization Disparities of Liver Transplantation in Taiwan	-EXCLUDE on country (High-Income)	

####	WENDT (2016) (ID:87857183)	Gender and attitudes about mental health help seeking: results from National Data	-EXCLUDE on population (no housing precarity)
####	Wenham (2020) (ID:87850682)	"Women are most affected by pandemics -Lessons from past outbreaks": Correction.	-EXCLUDE on population (no housing precarity)
####	WENZEL (2001) (ID:87856940)	Risk factors for major violence among homeless women	-EXCLUDE on date (2010)
####	Wenzel (2010) (ID:87849246)	Personal network correlates of alcohol, cigarette, and marijuana use among homeless youth.	-EXCLUDE on population (people aged under 18 years)
####	Wenzel (2012) (ID:87849143)	Behavioral health and social normative influence: correlates of concurrent sexual partnering among heterosexually-active homeless men.	-EXCLUDE on population (women)
####	Wenzel (2012) (ID:87849149)	Social networks of homeless youth in emerging adulthood.	-EXCLUDE on population (women)
####	Wenzel (2016) (ID:87848797)	Pilot Test of an Adapted, Evidence-Based HIV Sexual Risk Reduction Intervention for Homeless Women.	-EXCLUDE on intervention (service engagement/helpseeking behaviour)
####	Wenzel (2017) (ID:87853273)	Provider perceptions on HIV risk and prevention services within permanent supportive housing	-EXCLUDE on intervention (service engagement/helpseeking behaviour)
####	WERNER (2003) (ID:87857303)	Knowledge about symptoms of Alzheimer's disease: correlates and relationship to help-seeking behaviour	-EXCLUDE on date (2010)
####	Wesp (2019) (ID:87857748)	It's a Process: A Qualitative Study about the Resistance and Resilience of Transgender Youth of Color Navigating Parent/Guardian Support and Societal Oppressions	-EXCLUDE on population (people aged under 18 years)
####	West (2012) (ID:87851267)	Clinically unintended medication switches and inability to prescribe preferred medications under Medicare Part D.	-EXCLUDE on intervention (service engagement/helpseeking behaviour)
####	West (2015) (ID:87851026)	Planning patient-centered health homes for Medicaid psychiatric patients at greatest risk for intensive service use.	-EXCLUDE on intervention (service engagement/helpseeking behaviour)
####	West (2020) (ID:87853247)	The Role of a Food Literacy Intervention in Promoting Food Security and Food Literacy-OzHarvest's NEST Program	-EXCLUDE on population (no housing precarity)
####	West (2021) (ID:87857949)	Authenticating and Legitimizing Transgender and Gender Non-conforming Identities Online: A Discourse Analysis	-EXCLUDE on intervention (service engagement/helpseeking behaviour)
####	WESTERHOF (2008) (ID:87857212)	Intentions to seek (preventive) psychological help among older adults: an application of the theory of planned behaviour	-EXCLUDE on date (2010)
####	Whaling (2020) (ID:87857893)	Examining the Relations Between Gender, Latent Classes of Adverse Childhood Experiences (ACEs), and Internalizing/Externalizing Symptoms Among Latinx Teens	-EXCLUDE on population (no housing precarity)
####	Whisler (2021) (ID:87848223)	The effect of a Housing First intervention on primary care retention among homeless individuals with mental illness.	-EXCLUDE on intervention (service engagement/helpseeking behaviour)
####	Whitacre (2022) (ID:87857888)	A Program Evaluation of the New Choices Workforce Development Program and Its Impact on Women's Ability to Obtain Employment and Overcome Personal Challenges	-EXCLUDE on population (no housing precarity)
####	White (2016) (ID:87848728)	Effect of the Economic Recession on Primary Care Access for the Homeless.	-EXCLUDE on intervention (service engagement/helpseeking behaviour)
####	White (2018) (ID:87848569)	The effect of the global financial crisis on preventable hospitalizations among the homeless in New York State.	-EXCLUDE on intervention (service engagement/helpseeking behaviour)
####	White (2018) (ID:87857672)	Victim Empowerment, Safety, and Perpetrator Accountability Through Collaboration: A Crisis to Transformation Conceptual Model	-EXCLUDE on population (no housing precarity)
####	White (2023) (ID:87963058)	Global prevalence and mental health outcomes of intimate partner violence among women: a systematic review and meta-analysis	-EXCLUDE on intervention (service engagement/helpseeking behaviour)
####	White (2023) (ID:88019146)	Trauma, Help-Seeking, and the Strong Black Woman	-EXCLUDE on population (no housing precarity)
####	Whitley (2013) (ID:87852883)	Fear and loathing in New England: examining the health-care perspectives of homeless people in rural areas	-EXCLUDE on intervention (service engagement/helpseeking behaviour)
####	Whitley (2014) (ID:87848953)	Stigma, agency and recovery amongst people with severe mental illness.	-EXCLUDE on population (no housing precarity)
####	Whitley (2022) (ID:87857588)	Narratives of trauma and resilience from Street Soccer players	-EXCLUDE on intervention (service engagement/helpseeking behaviour)
####	Whittaker (2017) (ID:87848668)	First examination of varying health outcomes of the chronically homeless according to Housing First configuration.	-EXCLUDE on intervention (service engagement/helpseeking behaviour)

####	Whittier (2016) (ID:87963068)	Carceral and intersectional feminism in congress: The violence against women act, discourse, and policy	-EXCLUDE on population (no housing precarity)	
####	Wickersham (2016) (ID:87848785)	Patterns of substance use and correlates of lifetime and active injection drug use among women in Malaysia.	-EXCLUDE on country (High-Income)	
####	Wiens (2021) (ID:87848191)	Factors associated with higher healthcare costs in a cohort of homeless adults with a mental illness and a general cohort of adults with a history of homelessness.	-EXCLUDE on intervention (service engagement/helpseeking behaviour)	
####	Wiersma (2010) (ID:87849281)	Episodic illness, chronic disease, and health care use among homeless persons in Metropolitan Atlanta, Georgia, 2007.	-EXCLUDE on intervention (service engagement/helpseeking behaviour)	
####	Wiewel (2020) (ID:87848317)	Housing Subsidies and Housing Stability are Associated with Better HIV Medical Outcomes Among Persons Who Experienced Homelessness and Live with HIV and Mental Illness or Substance Use Disorder.	-EXCLUDE on intervention (service engagement/helpseeking behaviour)	
####	Wiker (2019) (ID:87848414)	Supported accommodation for people with schizophrenia.	-EXCLUDE on population (no housing precarity)	
####	Wikstrom (2018) (ID:87852685)	Sexual and reproductive health and rights (SRHR) education with homeless people in Sweden	-EXCLUDE on intervention (service engagement/helpseeking behaviour)	
####	Wilbur (2022) (ID:87853442)	The inclusion of disability within efforts to address menstrual health during humanitarian emergencies: A systematized review	-EXCLUDE on intervention (service engagement/helpseeking behaviour)	
####	Wild (2020) (ID:87963178)	Domestic violence & abuse: prevention, intervention and the politics of gender	-EXCLUDE on evidence and form (evidence not in written form or presented as research output)	
####	WILKINS (2010) (ID:87857300)	Untold problems: a review of the essential issues in the mental health of men and boys	-EXCLUDE on population (women)	
####	Wilkins (2020) (ID:87851980)	Experiences of Compassion Fatigue in Case Managers Serving Homeless Youth While Maintaining Ethics: A Qualitative Study	-EXCLUDE on intervention (service engagement/helpseeking behaviour)	
####	Willey (2020) (ID:87851614)	'If you don't ask ... you don't tell': Refugee women's perspectives on perinatal mental health screening	-INCLUDE on title & abstract	-EXCLUDE on target group (no housing precarity)
####	Willey (2022) (ID:87857707)	Racism, healthcare access and health equity for people seeking asylum	-EXCLUDE on population (women)	
####	Willey (2023) (ID:87857668)	A Mother's Bond: A Relational Approach to Strengthening Mother-Child Attachment in Pregnant Women Who Are Addicted to Substances and Have a History of Interpersonal Trauma	-EXCLUDE on intervention (service engagement/helpseeking behaviour)	
####	Williams (2013) (ID:87849059)	Gender differences in baseline health, needs at release, and predictors of care engagement among HIV-positive clients leaving jail.	-INCLUDE on title & abstract	-EXCLUDE on intervention (service engagement/helpseeking behaviour)
####	Williams (2014) (ID:87848938)	Centralized care management support for "high utilizers" in primary care practices at an academic medical center.	-EXCLUDE on intervention (service engagement/helpseeking behaviour)	
####	Williams (2016) (ID:87857571)	Critical Thinking in Social Policy: The Challenges of Past, Present and Future	-EXCLUDE on intervention (service engagement/helpseeking behaviour)	
####	Williams (2016) (ID:87857849)	Spring into action for black women: Examining the Black Lives Matter organization's Twitter coverage of state-sanctioned violence against black women	-EXCLUDE on population (no housing precarity)	
####	Williams (2018) (ID:87857790)	Wonder Women: How Race and Gender Influenced News Coverage in the 2017 New Orleans and Atlanta Mayoral Elections	-EXCLUDE on population (no housing precarity)	
####	Williams (2018) (ID:87963117)	Examining the impact of race/ethnicity and gender intersectionality on preferences of social distance from individuals with mental health conditions	-EXCLUDE on population (no housing precarity)	
####	Williams (2019) (ID:87857818)	A Survey of Black Churches' Responses to Domestic Violence	-EXCLUDE on intervention (service engagement/helpseeking behaviour)	
####	Williams (2021) (ID:87857608)	Violence and Health Promotion Among First Nations, Métis, and Inuit Women: A Systematic Review of Qualitative Research	-EXCLUDE on population (no housing precarity)	
####	Williams (2023) (ID:87853203)	We get by with family: Maternal partnership transitions and extended kin coresidence	-EXCLUDE on population (no housing precarity)	
####	WILLIAMSON (2013) (ID:87851435)	The TARA project: a longitudinal study of the service needs of homeless women	-INCLUDE on title & abstract	-EXCLUDE on intervention (service engagement/helpseeking behaviour)
####	Willows (2011) (ID:87849177)	Associations between household food insecurity and health outcomes in the Aboriginal population (excluding reserves).	-EXCLUDE on population (women)	

####	**Wilson (2015) (ID:87851005)**	**House to house, shelter to shelter: Experiences of Black women seeking housing after leaving abusive relationships.**	**-INCLUDE on title & abstract**	**-INCLUDE on full study**
####	Wilson (2015) (ID:87858023)	Black educational activism for community empowerment: International leadership perspectives	-EXCLUDE on population (no housing precarity)	
####	Wilson (2019) (ID:87850754)	The social impact of musical engagement for young adults with learning difficulties: A qualitative study.	-EXCLUDE on population (no housing precarity)	
####	Wilson (2020) (ID:87853187)	Disparities in the PrEP continuum for trans women compared to MSM in San Francisco, California: results from population-based cross-sectional behavioural surveillance studies	-EXCLUDE on intervention (service engagement/helpseeking behaviour)	
####	Wilson-Forsberg (2015) (ID:87857711)	The Volunteering Dogma and Canadian Work Experience: Do Recent Immigrants Volunteer Voluntarily?	-EXCLUDE on intervention (service engagement/helpseeking behaviour)	
####	Wilson-Mitchell (2016) (ID:87857799)	Infusing Diversity and Equity Into Clinical Teaching: Training the Trainers	-EXCLUDE on population (no housing precarity)	
####	Windsor (2010) (ID:87857654)	What is Substance Use About? Assumptions in New York's Drug Policies and the Perceptions of African Americans Who are Low-Income and Using Drugs	-EXCLUDE on intervention (service engagement/helpseeking behaviour)	
####	Winetrobe (2017) (ID:87848665)	Differences in Health and Social Support between Homeless Men and Women Entering Permanent Supportive Housing.	-INCLUDE on title & abstract	-EXCLUDE on intervention (intersectionality)
####	Wingo (2023) (ID:88019134)	Improving Reproductive Health Communication Between Providers and Women Affected by Homelessness and Substance Use in San Francisco: Results from a Community-Informed Workshop	-EXCLUDE on intervention (service engagement/helpseeking behaviour)	
####	Wingood (2013) (ID:87851127)	Improving health outcomes for IPV-exposed women living with HIV.	-EXCLUDE on evidence and form (evidence not in written form or presented as research output)	
####	Winiarski (2020) (ID:87848324)	Assessing and treating complex mental health needs among homeless youth in a shelter-based clinic.	-EXCLUDE on population (people aged under 18 years)	
####	Winiker (2023) (ID:87857638)	"Through the Things That Have Happened to Me, They've Made Me Stronger": Individual and Interpersonal Sources of Violence and Resilience Among a Diverse Sample of Transgender Women in Los Angeles	-EXCLUDE on population (no housing precarity)	
####	Winker (2011) (ID:87963104)	Intersectionality as multi-level analysis: Dealing with social inequality	-EXCLUDE on intervention (service engagement/helpseeking behaviour)	
####	Winston (1999) (ID:87853708)	Self-Help for Grandmothers Parenting Again	-EXCLUDE on date (2010)	
####	Wirth (2019) (ID:87848384)	"This Isn't Just about Things, It's about People and Their Future": A Qualitative Analysis of the Working Conditions and Strains of Social Workers in Refugee and Homeless Aid.	-EXCLUDE on intervention (service engagement/helpseeking behaviour)	
####	Wise (2013) (ID:87849027)	Hearing the silent voices: narratives of health care and homelessness.	-EXCLUDE on population (women)	
####	Witten (2014) (ID:87852911)	It's Not All Darkness: Robustness, Resilience, and Successful Transgender Aging	-EXCLUDE on population (no housing precarity)	
####	Wittman (2017) (ID:87851932)	The architecture of recovery: two kinds of housing assistance for chronic homeless persons with substance use disorders	-EXCLUDE on intervention (service engagement/helpseeking behaviour)	
####	Wittwer (2022) (ID:87853264)	Sex Differences in Incidence and Outcome of Out-of-Hospital Cardiac Arrest Within a Local Health Network	-EXCLUDE on population (no housing precarity)	
####	Wohl (2017) (ID:87848664)	Project Engage: Snowball Sampling and Direct Recruitment to Identify and Link Hard-to-Reach HIV-Infected Persons Who Are Out of Care.	-EXCLUDE on intervention (service engagement/helpseeking behaviour)	
####	Woith (2017) (ID:87848680)	Lessons from the Homeless: Civil and Uncivil Interactions with Nurses, Self-Care Behaviors, and Barriers to Care.	-EXCLUDE on population (no gender focus; women population <50)	
####	Wolitski (2010) (ID:87849282)	Randomized trial of the effects of housing assistance on the health and risk behaviors of homeless and unstably housed people living with HIV.	-EXCLUDE on intervention (service engagement/helpseeking behaviour)	
####	Women and homelessness... (2001) (ID:87856908)	Women and homelessness in Europe: pathways, services and experiences	-EXCLUDE on date (2010)	
####	WONG (1998) (ID:87856947)	Residential transitions among homeless families and homeless single individuals: a comparison study	-EXCLUDE on date (2010)	
####	WONG (2007) (ID:87857138)	Crucial individuals in the help-seeking pathway of Chinese caregivers of relatives with early psychosis in Hong Kong	-EXCLUDE on date (2010)	
####	Wong (2010) (ID:87851853)	Marked variations in proximal colon cancer survival by race/ethnicity within the United States.	-EXCLUDE on population (no housing precarity)	

####	Wong (2013) (ID:87857969)	An exploratory study on the mental health of immigrants, refugees and non-status people living with HIV in Toronto	-EXCLUDE on population (no gender focus; women population <50)<br>-EXCLUDE - but review for literature	
####	Wong (2016) (ID:87848803)	The Impact of Specific and Complex Trauma on the Mental Health of Homeless Youth.	-EXCLUDE on population (women)	
####	Wong (2019) (ID:87852992)	Racial/Ethnic Disparities in Mortality Across the Veterans Health Administration	-EXCLUDE on population (no housing precarity)	
####	Wong (2020) (ID:87852766)	'I want the heart of fierceness to arise within us': maintaining public space to promote HIV-related health with House Ball Community members in an era of gentrification	-EXCLUDE on intervention (service engagement/helpseeking behaviour)	
####	Wood (2020) (ID:87851899)	Playing by the Rules: Agency Policy and Procedure in Service Experience of IPV Survivors	-EXCLUDE on intervention (service engagement/helpseeking behaviour)	
####	Wood (2020) (ID:87857574)	Child modern slavery, trafficking and health: A practical review of factors contributing to children's vulnerability and the potential impacts of severe exploitation on health	-EXCLUDE on population (people aged under 18 years)	
####	Woodcock (2021) (ID:87850642)	The mental health help seeking experiences of female victims of intimate partner volence.	-INCLUDE on title & abstract	-EXCLUDE (IPV/DVA but little discussion on housing)
####	Woodhall-Melnik (2022) (ID:88019128)	Getting Help: Findings from Two World Cafés with Youth who Experience Homelessness	-EXCLUDE on population (women)	
####	WOODWARD (2010) (ID:87857296)	Differences in professional and informal help seeking among older African Americans, black Caribbeans, and non-hispanic whites	-EXCLUDE on population (no gender focus; women population <50)	
####	Woolley (2013) (ID:87858065)	Identity and Difference: Negotiating Gender and Sexuality in High School Contexts	-EXCLUDE on population (no housing precarity)	
####	Wooten (2012) (ID:87853364)	Deployment Cycle Stressors and Post-Traumatic Stress Symptoms in Army National Guard Women: The Mediating Effect of Resilience	-EXCLUDE on population (no housing precarity)	
####	Working with unaccompanied... (2007) (ID:87857066)	Working with unaccompanied asylum seeking children: issues for policy and practice	-EXCLUDE on date (2010)	
####	Workman (2019) (ID:87963098)	Are Australian public discourses on intimate partner violence LGBTIQ inclusive?	-EXCLUDE on intervention (service engagement/helpseeking behaviour)	
####	Wozniak (2012) (ID:87851285)	Ritual and performance in domestic violence healing: From survivor to thriver through rites of passage.	-INCLUDE on title & abstract	-EXCLUDE on intervention (intersectionality)
####	Wray (2012) (ID:87853063)	Pilot Prognostic Model of Extremely Poor Survival Among High-Risk Hepatocellular Carcinoma Patients	-EXCLUDE on population (no housing precarity)	
####	Wrenn (2017) (ID:87857550)	Trump veganism: A political survey of american vegans in the era of identity politics	-EXCLUDE on population (no housing precarity)	
####	Wright (2012) (ID:87849091)	Encouraging legal help seeking for victims of intimate partner violence: the therapeutic effects of the civil protection order.	-INCLUDE on title & abstract	-EXCLUDE on target group (no housing precarity)
####	Wright (2012) (ID:87853322)	Implementation and evaluation of a harm-reduction model for clinical care of substance using pregnant women	-EXCLUDE on population (no housing precarity)	
####	WRIGHT (2021) (ID:87857089)	Help-seeking and barriers to care in intimate partner sexual violence: a systematic review	-INCLUDE on title & abstract	-EXCLUDE (IPV/DVA but little discussion on housing)<br>-EXCLUDE (systematic review)* only use with other code
####	Wright (2022) (ID:87850615)	Work-life-imbalance during the COVID-19 pandemic: Exploring social support and health outcomes in the United States.	-EXCLUDE on population (no housing precarity)	
####	Wu (2016) (ID:87848767)	Posttraumatic stress disorder and posttraumatic growth coexistence and the risk factors in Wenchuan earthquake survivors.	-EXCLUDE on country (High-Income)	
####	Wu (2018) (ID:87851616)	Increased survival of patients aged 0-29 years with osteosarcoma: A period analysis, 1984-2013.	-EXCLUDE on population (no housing precarity)	
####	Wu (2018) (ID:87851817)	Lifetime risks, loss of life expectancy, and health care expenditures for 19 types of cancer in Taiwan.	-EXCLUDE on country (High-Income)	
####	Wu (2022) (ID:87851881)	Building Emergency Response Capacity: Multi-Career-Stage Social Workers' Engagement with Homeless Sector during the First Two Waves of COVID-19 in Halifax, Nova Scotia, Canada	-EXCLUDE on intervention (service engagement/helpseeking behaviour)	
####	Wurie (2023) (ID:87850556)	Studies to inform the development and practical roll-out of a digital adherence intervention, Video-Observed Therapy (VOT)	-EXCLUDE on population (no housing precarity)	

####	**WYDALL (2017) (ID:87857217)**	**Domestic abuse and older people: factors influencing help-seeking**	**-INCLUDE on title & abstract**	**-INCLUDE on full study**
####	Wyss (2022) (ID:87857736)	Disentangling entangled mobilities: reflections on forms of knowledge production within migration studies	-EXCLUDE on intervention (service engagement/helpseeking behaviour)	
####	Xanthoudakis (2021) (ID:87857737)	A Content Analysis of Québec's K-11 Sexuality Education Program Using UNESCO Guidelines	-EXCLUDE on intervention (service engagement/helpseeking behaviour)	
####	Xiao (2016) (ID:87848764)	Relationship between Housing Status and Retention Rates among HIV-Positive African Americans Enrolled in a Comprehensive Care Program.	-EXCLUDE on population (women)	
####	Yahalom (2023) (ID:87857516)	Military Sexual Trauma Among Men: Gendered Experiences, Clinical Considerations, and Treatment Options	-EXCLUDE on population (women)	
####	Yakubovich (2022) (ID:87963154)	Preventing gender-based homelessness in Canada during the COVID-19 pandemic and beyond: the need to account for violence against women	-EXCLUDE on intervention (service engagement/helpseeking behaviour)	
####	Yamada (2016) (ID:87848800)	Dual Sensory Impairment and Cognitive Decline: The Results From the Shelter Study.	-EXCLUDE on intervention (service engagement/helpseeking behaviour)	
####	Yamamoto (2019) (ID:87852935)	Association between homelessness and opioid overdose and opioid-related hospital admissions/emergency department visits	-EXCLUDE on intervention (service engagement/helpseeking behaviour)	
####	Yamamoto (2021) (ID:87848174)	Comparison of Childbirth Delivery Outcomes and Costs of Care Between Women Experiencing vs Not Experiencing Homelessness.	-INCLUDE on title & abstract	-EXCLUDE on intervention (service engagement/helpseeking behaviour)
####	YAMASHIRO (1997) (ID:87857167)	Help-seeking among Asian and Pacific Americans: a multiperspective analysis	-EXCLUDE on date (2010)	
####	Yamashita (2013) (ID:87851176)	The Great East Japan Earthquake, Tsunami, and Fukushima Daiichi Nuclear Power Plant accident: A triple disaster affecting the mental health of the country.	-EXCLUDE on intervention (service engagement/helpseeking behaviour)	
####	YAN (2015) (ID:87857141)	Elder abuse and help-seeking behavior in elderly Chinese	-EXCLUDE on country (High-Income)	
####	Yang (2015) (ID:87848807)	Bidirectional Influence: A Longitudinal Analysis of Size of Drug Network and Depression Among Inner-City Residents in Baltimore, Maryland.	-EXCLUDE on intervention (service engagement/helpseeking behaviour)	
####	**Yarbrough (2023) (ID:87963163)**	**The carceral production of transgender poverty: How racialized gender policing deprives transgender women of housing and safety**	**-INCLUDE on title & abstract**	**-INCLUDE on full study**
####	Yarwood (2022) (ID:87857729)	LGBTQI + Migrants: A Systematic Review and Conceptual Framework of Health, Safety and Wellbeing during Migration	-EXCLUDE on population (no housing precarity)	
####	Yasin (2021) (ID:87852955)	Prostitution: a new dynamic of discrimination	-EXCLUDE on intervention (service engagement/helpseeking behaviour)	
####	YEUNG (2013) (ID:87857311)	Role of social networks in the help-seeking experiences among Chinese suffering from severe mental illness in England: a qualitative study	-EXCLUDE on population (no housing precarity)	
####	Yi (2022) (ID:87850547)	Housing poverty and housing choice : the experiences of young South Koreans	-EXCLUDE on population (no housing precarity)	
####	Yildiz (2010) (ID:87963278)	Empowering Women or Perpetuating Victimhood: Minority Ethnic and Roma Women's Experiences of Domestic Violence Policy and Service Provision	-INCLUDE on title & abstract	-EXCLUDE (IPV/DVA but little discussion on housing)
####	Yim (2015) (ID:87848816)	Prevalence of Mental Illness among Homeless People in Hong Kong.	-EXCLUDE on population (no housing precarity)	
####	Yimer (2014) (ID:87848905)	Prevalence and risk factors of louse-borne relapsing fever in high risk populations in Bahir Dar city Northwest, Ethiopia.	-EXCLUDE on country (High-Income)	
####	Yingwana (2022) (ID:87857884)	Queering Sex Work and Mobility	-EXCLUDE on country (High-Income)	
####	Yoo (2016) (ID:87850955)	Perceptions of disaster preparedness among older people in South Korea.	-EXCLUDE on intervention (service engagement/helpseeking behaviour)	
####	Yoo-Jeong (2020) (ID:87848354)	Correlates of loneliness in older persons living with HIV.	-EXCLUDE on intervention (service engagement/helpseeking behaviour)	
####	YORKE (2016) (ID:87857258)	Cultural factors influencing mental health help-seeking attitudes among Black English-Speaking Caribbean immigrants in the United States and Britain	-EXCLUDE on population (no housing precarity)	
####	Yoshioka-Maxwell (2017) (ID:87848698)	Exploring the impact of network characteristics on substance use outcomes among homeless former foster youth.	-EXCLUDE on population (women)	

####	Young (2011) (ID:87849227)	Online social networking technologies, HIV knowledge, and sexual risk and testing behaviors among homeless youth.	-EXCLUDE on intervention (service engagement/helpseeking behaviour)	
####	Young (2013) (ID:87858099)	An Exploration of the Experiences of African American Women's Use of Network Relationships to Access Resources and Information to Benefit Communities: A Phenomenological Study	-EXCLUDE on population (no housing precarity)	
####	Young (2014) (ID:87848968)	Six-month outcomes of an integrated assertive community treatment team serving adults with complex behavioral health and housing needs.	-EXCLUDE on intervention (service engagement/helpseeking behaviour)	
####	Young (2018) (ID:87850813)	The relationship between the strong Black woman archetype and attitudes towards seeking professional psychological help in intimate partner violence relationships among African American women.	-INCLUDE on title & abstract	-EXCLUDE (IPV/DVA but little discussion on housing)
####	YOUNG (2019) (ID:87856915)	Promising practice from the frontline	-INCLUDE on title & abstract	-EXCLUDE on intervention (intersectionality)
####	Young-Xu (2016) (ID:87848725)	Impact of Paliperidone Palmitate Versus Oral Atypical Antipsychotics on Health Care Resource Use and Costs in Veterans with Schizophrenia.	-EXCLUDE on intervention (service engagement/helpseeking behaviour)	
####	Younts (2021) (ID:87857819)	American Epidemic: The Societal and Multi-generational Impacts Caused by the Mass Incarceration of Women in the United States	-EXCLUDE on intervention (service engagement/helpseeking behaviour)	
####	YOUSSEFF (2006) (ID:87857180)	Factors influencing mental-health help-seeking in Arabic-speaking communities in Sydney, Australia	-EXCLUDE on date (2010)	
####	Yozgat (2023) (ID:87851505)	Evaluation of Leukemia and Solid Tumors in Refugee Children in Turkey: A Tertiary Center Experience.	-EXCLUDE on country (High-Income)	
####	Yue (2022) (ID:87851561)	Differential associations of homelessness with emergency department visits and hospitalizations by race, ethnicity, and gender	-EXCLUDE on intervention (service engagement/helpseeking behaviour)	
####	Yuval-Davis (2016) (ID:87963291)	Power, intersectionality and the politics of belonging	-EXCLUDE on intervention (service engagement/helpseeking behaviour)	
####	Zaat (2021) (ID:87851666)	Is home-based monitoring of ovulation to time frozen embryo transfer a cost-effective alternative for hospital-based monitoring of ovulation? Study protocol of the multicentre, non-inferiority Antarctica-2 randomised controlled trial.	-EXCLUDE on intervention (service engagement/helpseeking behaviour)	
####	Zain (2015) (ID:87848814)	FACTORS ASSOCIATED WITH PREGNANCY AMONG UNMARRIED WOMEN IN MALAYSIA.	-EXCLUDE on country (High-Income)	
####	Zehetmair (2021) (ID:87852927)	A Qualitative Evaluation of a Mother and Child Center Providing Psychosocial Support to Newly Arrived Female Refugees in a Registration and Reception Center in Germany	-INCLUDE on title & abstract	-EXCLUDE on target group (no housing precarity)
####	Zelenev (2013) (ID:87849029)	Patterns of homelessness and implications for HIV health after release from jail.	-EXCLUDE on population (no gender focus; women population <50)	
####	Zeluf-Andersson (2019) (ID:87848467)	Beyond viral suppression: the quality of life of people living with HIV in Sweden.	-EXCLUDE on intervention (service engagement/helpseeking behaviour)	
####	Zeneidi (2011) (ID:87849298)	The French-style Americanization of homelessness in Bordeaux	-EXCLUDE on intervention (service engagement/helpseeking behaviour)	
####	Zeng (2018) (ID:87853157)	Sex Differences in Genetic Associations With Longevity	-EXCLUDE on country (High-Income)	
####	Zerai (2011) (ID:87857568)	An Assessment of Afro Centricism, Color-Blind Ideology, and Intersectionality	-EXCLUDE on population (no housing precarity)	
####	Zerger (2012) (ID:87851283)	Housing: A fundamental component of drug policy.	-EXCLUDE on intervention (service engagement/helpseeking behaviour)	
####	Zerger (2014) (ID:87848922)	The role and meaning of interim housing in housing first programs for people experiencing homelessness and mental illness.	-EXCLUDE on population (women)	
####	Zerger (2014) (ID:87857471)	Differential experiences of discrimination among ethnoracially diverse persons experiencing mental illness and homelessness	-EXCLUDE on population (no gender focus; women population <50)<br>-EXCLUDE - but review for literature	
####	Zevallos (2012) (ID:87852941)	Gender Disparities in Puerto Ricans Hospitalized with an Initial Acute Myocardial Infarction: A Population-based Perspective	-EXCLUDE on intervention (service engagement/helpseeking behaviour)	
####	Zeyen (2023) (ID:87857965)	Disabled at Work: Body-Centric Cycles of Meaning-Making	-EXCLUDE on population (no housing precarity)	

####	Zhang (2018) (ID:87848553)	Concurrent Disorders and Health Care Utilization Among Homeless and Vulnerably Housed Persons in Canada.	-EXCLUDE on population (women)	
####	Zhang (2018) (ID:87848574)	Cost-effective way to reduce stimulant-abuse among gay/bisexual men and transgender women: a randomized clinical trial with a cost comparison.	-EXCLUDE on intervention (service engagement/helpseeking behaviour)	
####	Zhang (2019) (ID:87852679)	Demographics and Clinical Profiles of Patients Visiting a Free Clinic in Miami, Florida	-EXCLUDE on intervention (service engagement/helpseeking behaviour)	
####	Zhao (2018) (ID:87848486)	Incarceration history, social network composition, and substance use among homeless youth in Los Angeles.	-EXCLUDE on intervention (service engagement/helpseeking behaviour)	
####	Zhao (2021) (ID:87963257)	Housing precarity, correlates, and unmet health care and HIV care needs among women living with HIV in Metro Vancouver, Canada	-EXCLUDE on intervention (service engagement/helpseeking behaviour)	
####	Zhao (2022) (ID:87853117)	The prevalence and social-structural correlates of housing status among women living with HIV in Vancouver, Canada	-INCLUDE on title & abstract	-EXCLUDE on intervention (service engagement/helpseeking behaviour)
####	Zhao (2023) (ID:87852720)	Precarious Housing Associated with Unsuppressed Viral load, sub-optimal Access to HIV Treatment and Unmet Health care Needs, Among Women Living with HIV in Metro Vancouver, Canada	-EXCLUDE on intervention (service engagement/helpseeking behaviour)	
####	ZHOU (2009) (ID:87857375)	Help-seeking in a context of AIDS stigma: understanding the healthcare needs of people with HIV/AIDS in China	-EXCLUDE on country (High-Income)	
####	ZHU (2012) (ID:87857170)	Physical child abuses in urban China: victims' perceptions of the problem and impediments to help-seeking	-EXCLUDE on country (High-Income)	
####	Zoeckler (2014) (ID:87858040)	"I think that they knew I wasn't a normal boy" exploring parental support of LGBT youth during the coming out process as a predictor of success in higher education: Practice recommendations for educational institutions	-EXCLUDE on population (no housing precarity)	
####	Zubkova (2018) (ID:87853406)	THE 'SOVIET BEGGAR': RECONSTRUCTION OF THE COLLECTIVE PORTRAIT OF BEGGARS IN 1950S	-EXCLUDE on intervention (service engagement/helpseeking behaviour)	
####	Zucker (2012) (ID:87849137)	Mobile outreach strategies for screening hepatitis and HIV in high-risk populations.	-EXCLUDE on intervention (service engagement/helpseeking behaviour)	
####	Zufferey (2016) (ID:87963331)	Homelessness and social work: An intersectional approach	-EXCLUDE on intervention (service engagement/helpseeking behaviour)	
####	Zufferey (2019) (ID:87963344)	Family homelessness in regional and urban contexts: Service provider perspectives	-EXCLUDE on population (no gender focus; women population <50)<br>-EXCLUDE - but review for literature	
####	Zullig (2017) (ID:87853146)	Cancer Incidence Among Patients of the US Veterans Affairs Health Care System: 2010 Update	-EXCLUDE on intervention (service engagement/helpseeking behaviour)	
####	Zulman (2015) (ID:87848848)	Multimorbidity and healthcare utilisation among high-cost patients in the US Veterans Affairs Health Care System.	-EXCLUDE on intervention (service engagement/helpseeking behaviour)	
####	Zulman (2017) (ID:87848673)	Effect of an Intensive Outpatient Program to Augment Primary Care for High-Need Veterans Affairs Patients: A Randomized Clinical Trial.	-EXCLUDE on intervention (service engagement/helpseeking behaviour)	
####	Zulman (2022) (ID:87848165)	Outpatient care fragmentation in Veterans Affairs patients at high-risk for hospitalization.	-EXCLUDE on intervention (service engagement/helpseeking behaviour)	
####	Zur (2014) (ID:87848898)	Unmet need among homeless and non-homeless patients served at health care for the homeless programs.	-EXCLUDE on population (no gender focus; women population <50)	
####	Zvolensky (2017) (ID:87852954)	Subjective Social Status and Anxiety and Depressive Symptoms and Disorders among Low Income Latinos in Primary Care: The Role of Emotion Dysregulation	-EXCLUDE on population (no housing precarity)	
####	郭飞莹 (2014) (ID:87851946)	Beyond the Clinical Model of Recovery: Recovery of a Chinese Immigrant Woman with Bipolar Disorder	-EXCLUDE on population (no housing precarity)	

Studies identified via backward and forward citations (included on title)

No	First Author	Title	Screen on Full Text
1	Bodkin (2015)	Reducing stigma in healthcare and law enforcement: A novel approach to service provision for street level sex workers	-EXCLUDE on intervention (intersectionality)
2	Boudrassa (2004)	Racism, sexism, and colonialism: The impact on the health of aboriginal womeninCanada	-EXCLUDE on date (2010)

3	Deering (2011)	A peer-led mobile outreach program and increased utilization ofdetoxification and residential drug treatment among female sex workers who use drugs in a Canadian setting	-EXCLUDE on intervention (intersectionality)
4	Farley (2011)	Garden oftruth: The prostitution and trafficking ofnative women in Minneso	-EXCLUDE on target group (no discussion of housing precarity)
5	Hwang (2008)	The effect of traumatic brain injury on the health of homeless people	-EXCLUDE on date (2010)
6	Monahan (1999)	Head injury and battered women: An initial inquiry	-EXCLUDE on date (2010)
7	Orchard (2012)	Sex work in the forest city: Experiences of sex work beginnings, types and clientele among women in London	-EXCLUDE on intervention (service engagement/helpseeking behaviour)
8	Topolovec (2017)	The high burden oftraumatic brain injury and comorbidities amongst homeless adults with mental illness	- EXCLUDE (Duplicate)
9	Azarmehr (2018)	Nursing practice strategies for prenatal care of homeless pregnant women	-EXCLUDE on intervention (service engagement/helpseeking behaviour)
10	Benbow (2014)	Using a capabilities approach to understand poverty and social exclusion of psychiatric survivors	-EXCLUDE on intervention (service engagement/helpseeking behaviour)
11	Burnett (2012)	Examining the effects of policies on the delivery of shelter services to women who have experienced intimate partner violence	-EXCLUDE on evidence and form (dissertation)
12	Chambers (2014)	Factors associated with poor mental health status among homeless women with and without dependent children	- EXCLUDE (Duplicate)
13	Fazel (2014)	The health of homeless people in high-income countries: Descriptive epidemiology, health consequences, and clinical and policy recommendations	- EXCLUDE (Duplicate)
14	Walsh (2013)	Aboriginal women's voices: Breaking the cycle of homelessness and incarceration	-EXCLUDE on intervention (service engagement/helpseeking behaviour)
15	Benbow (2015)	Mothers experiencing homelessness: Social exclusion, resistance, and health.	- EXCLUDE (Duplicate)
16	Davies (2008)	Intersectionality as buzzword: A sociology of science perspective on what makes a feminist theory successful	-EXCLUDE on date (2010)
17	Fortin (2015)	I WAS HERE: young mothers who have experienced homelessness use Photovoice and participatory qualitative analysis to demonstrate strengths and assets	- EXCLUDE (Duplicate)
18	Mill (2012)	Women in the shadows: Prenatal care for street-involved women.	-EXCLUDE on intervention (programm evaluation)
19	Montgomery (2014)	Sheltering Aboriginal women with mental illness in Ontario, Canada: Being "kicked" and nurtured.	-EXCLUDE on intervention (intersectionality not sufficiently discussed)
20	**Kirkman (2015)**	**'I just wanted somewhere safe': Women who are homeless with their children.**	**-INCLUDE on full study**
21	Huey (2014)	They just asked me why I became homeless: "Failure to ask" as a barrier to homeless women's ability to access services postvictimization.	- EXCLUDE (Duplicate)
22	Little (2015)	Between the abuser and the street: An intersectional analysis of housing challenges for abused women.	-EXCLUDE on intervention (service engagement/helpseeking behaviour)
23	Milaney (2017)	Understanding mothers experiencing homelessness: A gendered approach to finding solutions for family homelessness.	-EXCLUDE (quality appraisal)
24	Osuji (2015)	History of abuse and the experience of homelessness: A framework for assisting women overcome housing instability.	-EXCLUDE on intervention (intersectionality)
25	Watson (2016)	Social exclusion, health and hidden homelessness	- EXCLUDE (Duplicate)
26	Bradley (2018)	How does homelessness affect parenting behaviour? A systematic critical review and thematic synthesis of qualitative research	-EXCLUDE on evidence and form (literature review)
27	Bywaters (2015)	Exploring inequities in child welfare and child protection services: Explaining the 'inverse intervention law'	-EXCLUDE on intervention (service engagement/helpseeking behaviour)
28	Healy (2020)	Recognising the caring capabilities of birth families of removed children: Towards a critical policy agenda	-EXCLUDE on target group (no discussion of housing precarity)
29	Lofstrand (2016)	Cultural images and definitions of homeless women: Implications for policy and practice at the European level	-EXCLUDE on intervention (service engagement/helpseeking behaviour)

30	Azim (2019)	Mothering in the margin: A narrative inquiry of women with children in a homeless shelter.	-EXCLUDE on intervention (service engagement/helpseeking behaviour)
31	Burlingam (2010)	A house is not a home: A qualitative assessment of the life experiences of alcoholic homeless women	-EXCLUDE on intervention (service engagement/helpseeking behaviour)
32	Ecker (2021)	Experiences of LGBTQ adults who have accessed emergency shelters in a large Urban City in Canada	-EXCLUDE on population (no gender focus; women population <50)
33	Lyons (2016)	Experiences of trans women and two-spirit persons accessing women-specific health and housing services in a Downtown Neighborhood of Vancouver, Canada	- EXCLUDE (Duplicate)
34	Olivet (2019)	The Intersection of homelessness, racism, and mental illness.	-EXCLUDE on intervention (service engagement/helpseeking behaviour)
35	Kofman (2020)	Home is not always a haven: The domestic violence crisis amid the COVID-19 pandemic.	-EXCLUDE on population (no housing precarity)
36	Windsor (2010)	Dimensions ofoppression in the lives ofimpoverished black women who use drugs.	-EXCLUDE on intervention (service engagement/helpseeking behaviour)
37	Gillis (2010)	Recovery and homeless services: New directions for the field.	-EXCLUDE on intervention (service engagement/helpseeking behaviour)
38	Johnson (2005)	Day Centres for Homeless People: Spaces of Care or Fear?	-EXCLUDE on date (2010)
39	May (2007)	Alternative Cartographies of Homelessness: Rendering Visible Women's Experiences of "Visible" Homelessness	-EXCLUDE on date (2010)
40	Swick (2010)	The voices of single parent mothers who are homeless: Implications for early childhood professionals	-EXCLUDE on intervention (service engagement/helpseeking behaviour)
41	Esen (2017)	The homeless pregnant woman	- EXCLUDE (Duplicate)
42	Adebowale (2018)	There is no excuse for homelessness in Britain in 2018	-EXCLUDE on intervention (service engagement/helpseeking behaviour)
43	Paterson (1990)	Obstetric outcome in homeless women.	-EXCLUDE on date (2010)
44	Cutts (2015)	Homelessness during pregnancy: a unique, time-dependent risk factor of birth outcomes.	-EXCLUDE on intervention (service engagement/helpseeking behaviour)
45	Canfield (2017)	Maternal substance use and child protection: a rapid evidence assessment of factors associated with loss of child care.	-EXCLUDE on intervention (service engagement/helpseeking behaviour)
46	Richter (2017)	Delivering care to women who are homeless: a narrative inquiry into the experience of health care providers in an obstetrical unit.	-EXCLUDE on intervention (intersectionality)
47	Richards (2011)	Health behaviors and infant health outcomes in homeless pregnant women in the United States.	-EXCLUDE on intervention (service engagement/helpseeking behaviour)
48	**Alessi (2020)**	**Victimization and resilience among sexual and gender minority homeless youth engaging in survival sex.**	**-INCLUDE on full study**
49	Choi (2015)	Serving our youth 2015: The needs and experiences of lesbian, gay, bisexual, transgender, and questioning youth experiencing homelessness.	-EXCLUDE on intervention (service engagement/helpseeking behaviour)
50	Urban Insititute	Surviving the streets of New York: Experiences of LGBTQ youth, YMSM, and YWSW engaged in survival sex	-EXCLUDE on intervention (service engagement/helpseeking behaviour)
51	Keuroghlian (2014)	Out on the street: a public health and policy agenda for lesbian, gay, bisexual, and transgender youth who are homeless	- EXCLUDE (Duplicate)
52	Shelton (2015)	Transgender youth homelessness: Understanding programmatic barriers through the lens of cisgenderism	-EXCLUDE on population (people aged under 18 years)
53	Broll (2017)	Every time I try to get out, I get pushed back": The role of violent victimization in women's experiences of multiple episodes of homelessness	-EXCLUDE on intervention (intersectionality)
54	Cameron (2016)	From pillar to post: Homeless women's experiences of social care.	- EXCLUDE (Duplicate)
55	Wacquant (2009)	Punishing the Poor: The Neoliberal Government of Social Insecurity	-EXCLUDE on date (2010)
56	Pyne (2011)	Unsuitable bodies: Trans people and cisnormativity in shelter services	- EXCLUDE (Duplicate)
57	Ruttan (2010)	"Home and native land": Aboriginal young women and homelessness in the city	-EXCLUDE on intervention (service engagement/helpseeking behaviour)

58	Paradis (2012)	We're not asking, we're telling: an inventory of practices promoting the dignity, autonomy, and self-determination of women and families facing homelessness	-EXCLUDE on intervention (intersectionality)
59	Ponce (2014)	Homelessness, behavioral health hisorders and intimate partner violence: barriers to services for women.	- EXCLUDE (Duplicate)
60	Baker (2010)	Domestic violence, housing instability, and homelessness: A review of housing policies and program practices for meeting the needs of survivors	- EXCLUDE (Duplicate)
61	Fisher (2019)	To stay or to leave: Factors influencing victims' decisions to stay or leave a domestic violence emergency shelter	-EXCLUDE on intervention (intersectionality)
62	Nnawulezi (2018)	The influence of low-barrier and voluntary service policies on survivor empowerment in a domestic violence housing organization.	-EXCLUDE on intervention (intersectionality)
63	Begun (2015)	The paradox of homeless youth pregnancy: A review of challenges and opportunities.	-EXCLUDE on evidence and form (literature review)
64	Clarke (2020)	Conditionality in the context of housing-led homelessness policy: Comparing Australia's housing first agenda to Scotland's "rights-based" approach	-EXCLUDE on intervention (service engagement/helpseeking behaviour)
65	**Smid (2010)**	**The challenge of pregnancy among homeless youth: Reclaiming a lost opportunity.**	**-INCLUDE on full study**
66	Berkowitz (2020)	Structurally vulnerable neighborhood environments and racial/ethnic COVID-19 inequities	-EXCLUDE on intervention (service engagement/helpseeking behaviour)
67	Femi-Ajao (2018)	A qualitative systematic review of published work on disclosure and help-seeking for domestic violence and abuse among women from ethnic minority populations	-EXCLUDE on evidence and form (literature review)
68	Beltran (2019)	Intersectional discrimination is associated with housing instability among trans women living in the San Francisco Bay area	- EXCLUDE (Duplicate)
69	Hanssmann (2020)	Trans homelessness. In: Stop the Revolving Door: A street-Level Framework for a New System.	-EXCLUDE on evidence and form

	**Studies identified via trackers (included on title)**		
1	Bjattacharya (2021)	Stakeholders facilitating hope and empowerment amidst social suffering: A qualitative documentary analysis exploring lives of homeless women with mental illness	-EXCLUDE on intervention (intersectionality)
2	Lopez (2023)	Substance misuse themes among women living in transitional housing: Effects on children, intimate partner relationships, and social	-EXCLUDE on intervention (service engagement/helpseeking behaviour)
3	Green (2023)	Influence of Cultural Norms on Formal Service Engagement Among Survivors of Intimate Partner Violence: A Qualitative Meta-synthesis	-EXCLUDE on evidence and form (literature review)
4	**Abramovich (2023)**	**Investigating the mental health outcomes among LGBTQ+ youth experiencing homelessness in York Region, Ontario**	**-INCLUDE on full study**
5	Theobald	Supporting Pregnant Women Experiencing Homelessness	- EXCLUDE (Duplicate)
