# Supplementary material for: A systematic narrative review of the research evidence of the impact of intersectionality on service engagement and help-seeking across different groups of women, trans women, and non-binary individuals experiencing homelessness and housing exclusion
Source: PLoS One. 2025 Apr 24;20(4):e0321300. doi: 10.1371/journal.pone.0321300 (PMC12021236; doi:10.1371/journal.pone.0321300)
Supplement: Attachment — Submitted filename: PlosOne response 9.1.25.docx [file pone.0321300.s008.docx]

**Manuscript** **PONE-D-24-34499**

**Response to Reviewers and Editor**

Date: 11/01/2025

Dear Dr. Chekuri,

We thank you and the reviewers for the thorough feedback on our systematic review “A systematic review of the research evidence of the impact of intersectionality on service engagement and help-seeking across different groups of women, trans women, and non-binary individuals experiencing homelessness and housing exclusion” and giving us the opportunity to submit a revised draft.

We appreciate the time and efforts dedicated to providing feedback to our paper which have greatly improved the quality of our manuscript. We hope we have adequately responded to the comments and addressed all concerns. Please see below for a point-by-point response to the editor’s and reviewers’ comments. Changes are highlighted in yellow within the manuscript.

Thank you again for your careful evaluation and we look forward to hearing from you.

**Point-by-point responses**

| **Editor** | |
| --- | --- |
| *PLOS ONE style* | We have ensured that the manuscript fitted PLOS ONE style, adjusting the title page, headers, and removing the funder from the acknowledgement section. |
| *Financial Disclosure* | Please add the financial disclose to our submission form “The funders had no role in study design, data collection and analysis, decision to publish, or preparation of the manuscript." References to the funder have been taken out of the acknowledgement section. |
| *Table identifying all screened studies* | We added a table of all studies identified in the literature search, including those excluded from the analyses and the reasons for exclusion in the supplementary material. The table was extracted from EPPI, including first author of the screened study, title, and year of publication. If required, we can also share the exported files from the different databases, which would make it easier to replicate the screening process. Unpublished studies that we were unable to access were excluded and appear as “Excluded on form” in the Prisma Flow Chart (p.10/11). |
| *Data extraction table* | The table with the primary data extracted is included in S2 Appendix. The first author (CH) extracted the data between Feb-April 2024 (line 276). Findings of included studies were imported to NVivo and thematically analysed (as noted on line 286). A copy of the themes analysed on NVivo can also be provided if required. |
| *Missing Data* | Data was qualitatively analysed using narrative methods. As we did not conduct any statistical analysis, there was no concern over missing data. |
| *Risk of bias/certainty assessments* | No risk of bias and quality/certainty assessments was conducted. |
| *Captions* | We have included captions on the Supporting Information files at the end of our manuscript, and updated any in-text citations to match accordingly. |

| **Reviewer #1** | |
| --- | --- |
| *Target outcomes and interventions are not clear* | We have reformulated the Research Question (RQ) (lines 78-79) to emphasise the review’s focus on the impact of intersectionality on service access and engagement. We have also included a brief explanation regarding the discrepancy between the review and the protocol (lines 93-95).  We have taken out references to “recovery” from title, RQ and methods, as we agree with the reviewers that analysed papers had not sufficiently addressed recovery (in terms of drug and alcohol dependency). We have also added a definition of support-trajectory, service engagement and help-seeking (lines 114-120).  Finally, we have made efforts to more clearly demonstrate how the results address the RQ, by a) clearer transitions to the findings when appropriate (e.g. 553-555) and b) restructuring the discussion to more directly respond to the original question (e.g. lines 699-707). |
| *Support services in question (i.e. focus on low-threshold services) are not clear* | We also provided an explanation for the lack of emphasis on low-threshold services (lines 344-350). Contrarily to what was expected and what we aimed for in the search strategy (line 178), included studies had a greater focus on statutory services. |
| *Other inconsistencies addressed* | We have also addressed the other inconsistencies with target setting, and typos. We mentioned criminal justice more specifically as a support service in question (line 83), and added ethnicity to ‘race’ (e.g. line 85). We also gave more detail about the advisory group (line 297) and removed the quote on line 465. |
| *Recommendations on specific intersectional identity groups based on review findings* | We felt that including specific recommendations for specific groups would not necessarily reflect our findings and the concept of intersectionality. The different dimensions were shown to be closely intertwined and will need to be examined in relation to each other rather than separately. However, we have emphasised this dilemma more clearly in the discussion and have given some recommendations on how intersectionality in general, inequalities and power dynamics could be addressed (e.g. lines 684-687; 699-704). |
| **Reviewer #2** | |
| *Justification of including ‘non-binary’ and* | Despite only a small proportion of included studies (and participants within them), where participants self-identified as ‘non-binary’, we chose not to remove references to this group. We felt that doing so may even further contribute to their invisibility and exclusion. Instead, we emphasised (line 310) that non-binary individuals made up of a very small proportion of participants in the included studies and limitation of this (line 767). We also provided participant summary statistics on gender (women, transgender, and non-binary identities) and a brief explanation on how gender was assessed and evaluated in the studies (lines 312-315). We also removed references to non-binary if they were not specifically invoked in the included studies, and sometimes replaced the construct of ‘women, trans women, and non-binary’ with ‘participants’/’people’ when we felt that the theme could more generally apply to the homelessness population. In a few places, we have added in barriers that were mentioned more explicitly to non-binary and gender-diverse individuals in the papers (e.g. line 434). |
| *operational definition of gender* | We have provided a better operational definition of how gender was defined (lines 131-135). |
