# Supplementary material for: A systematic narrative review of the research evidence of the impact of intersectionality on service engagement and help-seeking across different groups of women, trans women, and non-binary individuals experiencing homelessness and housing exclusion
Source: PLoS One. 2025 Apr 24;20(4):e0321300. doi: 10.1371/journal.pone.0321300 (PMC12021236; doi:10.1371/journal.pone.0321300)
Supplement: Attachment — Submitted filename: PlosOne response 05.02.docx [file pone.0321300.s010.docx]

**Manuscript** **PONE-D-24-34499**

**Response to Reviewers and Editor**

Date: 05/02/2025

Dear Dr. Chekuri,

We thank you and the reviewers for your response on the revision of our systematic review “A systematic review of the research evidence of the impact of intersectionality on service engagement and help-seeking across different groups of women, trans women, and non-binary individuals experiencing homelessness and housing exclusion” and giving us the opportunity to submit a revised draft.

Please see below for a point-by-point response to the editor’s comments. Changes are highlighted in yellow within the manuscript. We hope we have adequately responded to the comments and addressed all concerns.

Thank you again for your careful evaluation and we look forward to hearing from you.

**Point-by-point responses**

| **Editor** |  |
| --- | --- |
| Title: Please consider revising the phrase "A systematic review..." to "A systematic narrative review...." | We changed all mentions of “systematic review” to “systematic narrative review” |
| Line 17 and Line 46: Please consider revising the phrase "age of death" to "age at death" | We changed “age of death” to “age at death” at line 17 and line 46. |
| Line 17: Like other reviewer pointed out please delete the duplicate phrase "that of" from this Line. | We deleted the duplicate. |
| Line 47: Please consider revising the word "contexts" to "countries" | We replaced “contexts” to “countries.” |
| Line 60: Please consider revising the phrase: "barriers to access to services" to "barriers for accessing services" | We rephrased the phrase. |
| Line 68: Please consider deleting comma: "queer, questioning" | Instead of deleting comma, we deleted the “and” before “queer, questioning” |
| Page 10-11: Fig: 1: The math is not adding up. Please proofread the Prisma Flow chart and update the manuscript accordingly. For instance, after adding 14+16+5534-1425+4, the result is 4143 and not 4109 as depicted in the figure. Please explain. | Thank you for pointing this out. The numbers added up, but the way we had noted them down was indeed confusing. We have changed the way we have listed the different databases (which the 14 and 16 had referred to) and the numbers of studies we have screened (4109). The additional 4 studies were added after the first screening process (line 214/227) and thus not included in the 4109. |
