# Supplementary material for: A systematic narrative review of the research evidence of the impact of intersectionality on service engagement and help-seeking across different groups of women, trans women, and non-binary individuals experiencing homelessness and housing exclusion
Source: PLoS One. 2025 Apr 24;20(4):e0321300. doi: 10.1371/journal.pone.0321300 (PMC12021236; doi:10.1371/journal.pone.0321300)
Supplement: Attachment — Submitted filename: PlosOne response 27.02.docx [file pone.0321300.s011.docx]

**Manuscript** **PONE-D-24-34499**

**Response to Reviewers and Editor**

Date: 27/02/2025

Dear Dr. Chekuri,

We thank you on the revision of our systematic review “A systematic narrative review of the research evidence of the impact of intersectionality on service engagement and help-seeking across different groups of women, trans women, and non-binary individuals experiencing homelessness and housing exclusion” and giving us the opportunity to submit a revised draft.

Please see below for a point-by-point response. Changes are highlighted in yellow within the manuscript. We hope we have adequately responded to the comments and addressed all concerns.

**Point-by-point responses**

| **Editor** |  |
| --- | --- |
| Line 241 (Prisma) | We thank you for pointing this out and have changed the number to “3663” |
| Line 250 (Prisma) | We have changed the number to 395 |
| Line 298: Please describe how many people were part of the advisory group and a break up of number of people with lived experience of homelessness and number of researchers | We have added the number of people who are part of the advisory group. However, there is overlap between “people with lived experience” and practitioners/researchers. One of the researchers and one of the practitioners also have lived experience of homelessness.  We added a sentence that describes how many people are practitioners/researchers and how many of the advisory group have lived experience. |
| Line 390: Quotation | We brough (Nicola) into the quotation. |
| Line 633: Identify | We corrected the typo. |
